# Genome-wide identification and characterisation of bHLH transcription factors in *Artemisia annua*

**DOI:** 10.1186/s12870-023-04063-8

**Published:** 2023-02-01

**Authors:** Shuwei Chang, Qi Li, Baokang Huang, Wansheng Chen, Hexin Tan

**Affiliations:** 1 Department Chinese Medicine Authentication, College of Pharmacy, Naval Medical University (Second Military Medical University), Shanghai, China; 2 Department of Pharmacy, Shanghai Fourth People’s Hospital Affiliated to Tongji University School of Medicine, Shanghai, China; 3 Shanghai Key Laboratory for Pharmaceutical Metabolite Research, Shanghai, China

**Keywords:** *A. annua*, Artemisinin, bHLH, Myc, Regulation, Transcription factors, Trichome

## Abstract

**Background:**

*A. annua* (also named *Artemisia annua*, sweet wormwood) is the main source of the anti-malarial drug artemisinin, which is synthesised and stored in its trichomes. Members of the basic Helix-Loop-Helix (bHLH) family of transcription factors (TFs) have been implicated in artemisinin biosynthesis in *A. annua* and in trichome development in other plant species.

**Results:**

Here, we have systematically identified and characterised 226 putative bHLH TFs in *A. annua*. All of the proteins contain a HLH domain, 213 of which also contain the basic motif that mediates DNA binding of HLH dimers. Of these, 22 also contained a Myc domain that permits dimerisation with other families of TFs; only two proteins lacking the basic motif contained a Myc domain. Highly conserved GO annotations reflected the transcriptional regulatory role of the identified TFs, and suggested conserved roles in biological processes such as iron homeostasis, and guard cell and endosperm development. Expression analysis revealed that three genes (*AabHLH80*, *AabHLH96*, and *AaMyc-bHLH3*) exhibited spatiotemporal expression patterns similar to genes encoding key enzymes in artemisinin synthesis.

**Conclusions:**

This comprehensive analysis of bHLH TFs provides a new resource to direct further analysis into key molecular mechanisms underlying and regulating artemisinin biosynthesis and trichome development, as well as other biological processes, in the key medicinal plant *A. annua*.

**Supplementary Information:**

The online version contains supplementary material available at 10.1186/s12870-023-04063-8.

## Background

The bHLH (basic Helix-Loop-Helix) proteins are one of the most important transcription factor (TF) families present in all eukaryotes: from red algae and yeasts to higher plants and animals [1]. These proteins usually contain a highly conserved bHLH domain of 45–60 amino acids [2]. The HLH region comprises two generally hydrophobic helices linked by a loop region [3], and is critical for homo- or hetero-dimerisation of HLH proteins into functional TFs [4]. The basic motif, rich in basic amino acids (particularly arginine), mediates DNA recognition and binding to E-box or G-box hexanucleotide consensus sequences (5′-CANNTG-3′). Binding of bHLH TFs to E-box sequences has been shown to regulate gene expression in a wide range of biological processes, including cell differentiation, development and other processes, e.g., regulating flag angle, in rice [5]; determining lateral root initiation in *Arabidopsis thaliana* [6]; modulating multiple stress pathways [7, 8]; and controlling iron homeostasis [9] and hormone signalling [10]. HLH proteins lacking the basic motif can act as repressors by forming heterodimers to sequester bHLH proteins into inactive complexes unable to bind DNA [11].

Some bHLH TFs contain an additional N-terminal Myc domain. The Myc domain was first identified in oncogenes, and Myc-domain proteins promote proliferation and apoptosis and inhibit terminal differentiation in the genesis of an extraordinarily wide range of cancers [12]. Human c-Myc, a nuclear protein [13], was shown to interact with a bHLH protein Max to promote transcriptional activity [14, 15]; and Myc-bHLH proteins, encoding both Myc and bHLH domains, have also been reported [16, 17]. In plants, Myc-bHLH TFs contain an MYB interaction region (MIR), which can interact with an R2R3–MYB domain protein to affect transcription and downstream processes [18].

*A. annua* (Asteraceae) produces artemisinin, the powerful anti-malarial drug, mainly in its trichomes [19]. The key enzymes involved in artemisinin biosynthesis include ADS (amorpha4,11-diene synthase), DBR2 (artemisinic aldehyde delta-11 (13) reductase), CYP71AV1 (Cytochrome P450 monooxygenase), and ALDH1 (aldehyde dehydrogenase 1) [20–22]. Several bHLH TFs have been reported to be involved in artemisinin synthesis, e.g., AabHLH1(AaMyc-bHLH3, in the following naming of this study) [23]; bHLH112 (AabHLH65) that acts indirectly via ERF1 [24]; and AaPIF3 (AabHLH20), whose overexpression promotes artemisinin production [25].

In model plants Arabidopsis and rice, 162 [26] and 167 [27] bHLH, respectively, have been identified. As the genome sequences of more species are published and bioinformatic technologies become more refined, the identification of bHLH TFs in a larger number of species is being completed, e.g., potato [28], apple [29], maize [30], wheat [31]. Here, we have identified 226 putative bHLH TFs from *A. annua*, and analysed the bHLH domain structures, phylogeny, and gene ontology (GO) annotations of the TFs. Examination of their protein–protein interaction (PPI) network identified key hub genes, and transcriptomic analyses has identified potential genes involved in artemisinin biosynthesis and trichome development.

## Results

### Characterisation of bHLH TFs in *A. annua*

A total of 247 bHLH sequences were identified from the existing *A. annua* protein database [32] using a Hidden Markov Model search for the PF00010 (HLH) domain. A subsequent BlastP search using the amino acid sequences of 88 bHLH TFs from Arabidopsis identified 59 sequences. After combining the two sets of results and removing repeated entries, 226 sequences were identified (Table 1; cDNA sequences in Supplemental Material 2, gDNA sequences in Supplemental Material 3 and protein sequences in Supplemental Material 4). The presence of HLH domains in these sequences was confirmed by HMMscan and the NCBI Conserved Domains tool.

**Table 1:** AabHLH TFs identified in *A. annua*

Name	Protein Code	Name	Protein Code	Name	Protein Code	Name	Protein Code
**AabHLH with basic motifs**							
AabHLH1	PWA55990.1	AabHLH51	PWA70090.1	AabHLH101	PWA70681.1	AabHLH151	PWA77712.1
AabHLH2	PWA57731.1	AabHLH52	PWA38953.1	AabHLH102	PWA90530.1	AabHLH152	PWA56812.1
AabHLH3	PWA69418.1	AabHLH53	PWA55232.1	AabHLH103	PWA34988.1	AabHLH153	PWA58322.1
AabHLH4	PWA74304.1	AabHLH54	PWA81454.1	AabHLH104	PWA62058.1	AabHLH154	PWA81347.1
AabHLH5	PWA74945.1	AabHLH55	PWA78891.1	AabHLH105	PWA67558.1	AabHLH155	PWA60013.1
AabHLH6	PWA41055.1	AabHLH56	PWA72082.1	AabHLH106	PWA77835.1	AabHLH156	PWA48976.1
AabHLH7	PWA77304.1	AabHLH57	PWA55099.1	AabHLH107	PWA72171.1	AabHLH157	PWA66871.1
AabHLH8	PWA90906.1	AabHLH58	PWA74264.1	AabHLH108	PWA83828.1	AabHLH158	PWA97849.1
AabHLH9	PWA66892.1	AabHLH59	PWA95405.1	AabHLH109	PWA46034.1	AabHLH159	PWA92669.1
AabHLH10	PWA64011.1	AabHLH60	PWA78689.1	AabHLH110	PWA48940.1	AabHLH160	PWA87024.1
AabHLH11	PWA82746.1	AabHLH61	PWA53253.1	AabHLH111	PWA66928.1	AabHLH161	PWA76568.1
AabHLH12	PWA74544.1	AabHLH62	PWA66955.1	AabHLH112	PWA64110.1	AabHLH162	PWA67171.1
AabHLH13	PWA55110.1	AabHLH63	PWA98212.1	AabHLH113	PWA81915.1	AabHLH163	PWA77805.1
AabHLH14	PWA89112.1	AabHLH64	PWA35033.1	AabHLH114	PWA87169.1	AabHLH164	PWA70091.1
AabHLH15	PWA88446.1	AabHLH65	PWA98909.1	AabHLH115	PWA59093.1	AabHLH165	PWA74643.1
AabHLH16	PWA39370.1	AabHLH66	PWA88561.1	AabHLH116	PWA59095.1	AabHLH166	PWA98724.1
AabHLH17	PWA83315.1	AabHLH67	PWA94446.1	AabHLH117	PWA63660.1	AabHLH167	PWA77387.1
AabHLH18	PWA70682.1	AabHLH68	PWA99436.1	AabHLH118	PWA98080.1	AabHLH168	PWA34916.1
AabHLH19	PWA67204.1	AabHLH69	PWA45656.1	AabHLH119	PWA59562.1	AabHLH169	PWA93684.1
AabHLH20	PWA54178.1	AabHLH70	PWA36239.1	AabHLH120	PWA85499.1	AabHLH170	PWA79273.1
AabHLH21	PWA41219.1	AabHLH71	PWA83404.1	AabHLH121	PWA78892.1	AabHLH171	PWA92205.1
AabHLH22	PWA58889.1	AabHLH72	PWA97988.1	AabHLH122	PWA50798.1	AabHLH172	PWA59498.1
AabHLH23	PWA72748.1	AabHLH73	PWA82566.1	AabHLH123	PWA61984.1	AabHLH173	PWA42285.1
AabHLH24	PWA70680.1	AabHLH74	PWA96313.1	AabHLH124	PWA88991.1	AabHLH174	PWA52612.1
AabHLH25	PWA51550.1	AabHLH75	PWA61596.1	AabHLH125	PWA74675.1	AabHLH175	PWA56560.1
AabHLH26	PWA60184.1	AabHLH76	PWA91784.1	AabHLH126	PWA51864.1	AabHLH176	PWA74989.1
AabHLH27	PWA62541.1	AabHLH77	PWA92385.1	AabHLH127	PWA97564.1	AabHLH177	PWA91485.1
AabHLH28	PWA72494.1	AabHLH78	PWA56005.1	AabHLH128	PWA88733.1	AabHLH178	PWA80814.1
AabHLH29	PWA48286.1	AabHLH79	PWA88979.1	AabHLH129	PWA48176.1	AabHLH179	PWA75561.1
AabHLH30	PWA62059.1	AabHLH80	PWA38629.1	AabHLH130	PWA36675.1	AabHLH180	PWA75480.1
AabHLH31	PWA74043.1	AabHLH81	PWA94475.1	AabHLH131	PWA35238.1	AabHLH181	PWA75332.1
AabHLH32	PWA76291.1	AabHLH82	PWA39576.1	AabHLH132	PWA58321.1	AabHLH182	PWA71968.1
AabHLH33	PWA65870.1	AabHLH83	PWA85745.1	AabHLH133	PWA64232.1	AabHLH183	PWA66394.1
AabHLH34	PWA63780.1	AabHLH84	PWA61486.1	AabHLH134	PWA57685.1	AabHLH184	PWA65621.1
AabHLH35	PWA42437.1	AabHLH85	PWA81107.1	AabHLH135	PWA80753.1	AabHLH185	PWA60455.1
AabHLH36	PWA50122.1	AabHLH86	PWA87571.1	AabHLH136	PWA42451.1	AabHLH186	PWA60454.1
AabHLH37	PWA82054.1	AabHLH87	PWA72698.1	AabHLH137	PWA38987.1	AabHLH187	PWA54382.1
AabHLH38	PWA47106.1	AabHLH88	PWA79032.1	AabHLH138	PWA75302.1	AabHLH188	PWA54211.1
AabHLH39	PWA58665.1	AabHLH89	PWA37000.1	AabHLH139	PWA46789.1	AabHLH189	PWA49757.1
AabHLH40	PWA67154.1	AabHLH90	PWA96097.1	AabHLH140	PWA77102.1	AabHLH190	PWA44498.1
AabHLH41	PWA81252.1	AabHLH91	PWA96220.1	AabHLH141	PWA98866.1	AabHLH191	PWA43178.1
AabHLH42	PWA59408.1	AabHLH92	PWA77406.1	AabHLH142	PWA96898.1		
AabHLH43	PWA79337.1	AabHLH93	PWA61333.1	AabHLH143	PWA55728.1		
AabHLH44	PWA98349.1	AabHLH94	PWA69329.1	AabHLH144	PWA65904.1		
AabHLH45	PWA66229.1	AabHLH95	PWA65572.1	AabHLH145	PWA80754.1		
AabHLH46	PWA75111.1	AabHLH96	PWA81496.1	AabHLH146	PWA49330.1		
AabHLH47	PWA89490.1	AabHLH97	PWA53644.1	AabHLH147	PWA83667.1		
AabHLH48	PWA52643.1	AabHLH98	PWA99414.1	AabHLH148	PWA57582.1		
AabHLH49	PWA79335.1	AabHLH99	PWA59942.1	AabHLH149	PWA68771.1		
AabHLH50	PWA44100.1	AabHLH100	PWA57418.1	AabHLH150	PWA73613.1		
**AabHLH without a basic motifs**							
AaHLH1	PWA34245.1	AaHLH4	PWA66854.1	AaHLH7	PWA80110.1	AaHLH10	PWA51571.1
AaHLH2	PWA58072.1	AaHLH5	PWA60185.1	AaHLH8	PWA79663.1	AaHLH11	PWA48370.1
AaHLH3	PWA79264.1	AaHLH6	PWA97762.1	AaHLH9	PWA56300.1		
**AaMyc-bHLH with Myc and basic motifs**							
AaMyc-bHLH1	PWA49079.1	AaMyc-bHLH7	PWA55744.1	AaMyc-bHLH13	PWA89854.1	AaMyc-bHLH18	PWA38056.1
AaMyc-bHLH2	PWA57173.1	AaMyc-bHLH8	PWA44127.1	AaMyc-bHLH14	PWA35954.1	AaMyc-bHLH19	PWA64470.1
AaMyc-bHLH3	PWA45362.1	AaMyc-bHLH9	PWA45393.1	AaMyc-bHLH15	PWA50104.1	AaMyc-bHLH20	PWA83493.1
AaMyc-bHLH4	PWA52316.1	AaMyc-bHLH10	PWA63781.1	AaMyc-bHLH16	PWA42908.1	AaMyc-bHLH21	PWA51892.1
AaMyc-bHLH5	PWA67441.1	AaMyc-bHLH11	PWA38066.1	AaMyc-bHLH17	PWA90985.1	AaMyc-bHLH22	PWA34774.1
AaMyc-bHLH6	PWA97833.1	AaMyc-bHLH12	PWA69288.1				
**AaMyc-HLH with Myc but without a basic motifs**							
AaMyc-HLH1	PWA53552.1	AaMyc-HLH2	PWA53551.1				

### Analysis of the conserved domains of AabHLH TFs

**Fig. 1:**
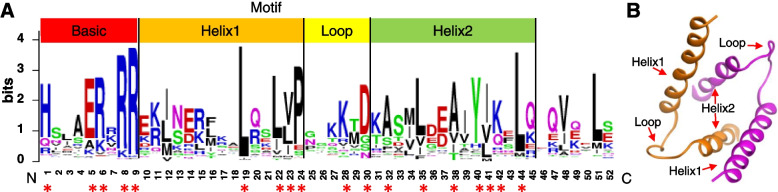
Sequence motifs and predicted structure of the bHLH domain. **A** amino acid sequences of *A. annua* bHLH domains. bHLH domains generally contain four conserved motifs: a basic, two helices, and one loop that connects the helices. Amino acids conserved over > 50% proteins are marked by red asterisks. **B** The three-dimensional structure of a bHLH homologous dimer showing orientations of loops and helices. The two monomers are shown in different colours

An alignment of the amino acid sequences of these 226 TFs was generated. Four conserved motifs are typically found in the bHLH domain, namely one basic motif, two helical motifs, and one loop that connected the two helices to form the helix-loop-helix (HLH) domain (Fig. 1A). The 9 aa basic motif of AabHLH TFs contained five highly conserved residues (His-1; Glu-5; Arg-6, 8, 9); the 14 aa helical motifs contained four (Leu-19, 22; Val-23; Pro-44) and seven (Ala-32, 38; Leu-35; Tyr-40; Ile-41; Lys-42; Leu-44) conserved residues in helix 1 and 2, respectively; while the 6 aa loop contained two conserved residues (Lys-28; Asp-30; Fig. 1A).

A predicted three-dimensional structure of the highest consensus sequence was generated, and confirmed the presence of two helices and intervening loop (Fig. 1B). The predicted structure easily forms homo- and hetero-dimers, consistent with the known requirement for bHLH TFs to form dimers to function and maintain stability (Fig. 1B).

The vast majority of AabHLH TFs (191/226) contained a basic motif and an HLH domain (AabHLH1–191), while eleven lacked the basic motif (AaHLH1–11). A further 24 TFs contained an additional Myc domain (PF00249), comprising three short repeated sequences upstream of the bHLH domain. Of these, 22 contained the basic motif (AaMyc-bHLH1–22), while the last two lacked the basic motif (AaMyc-HLH1–2; Table 1).

### Phylogenetic analysis of AabHLH TFs

**Fig. 2:**
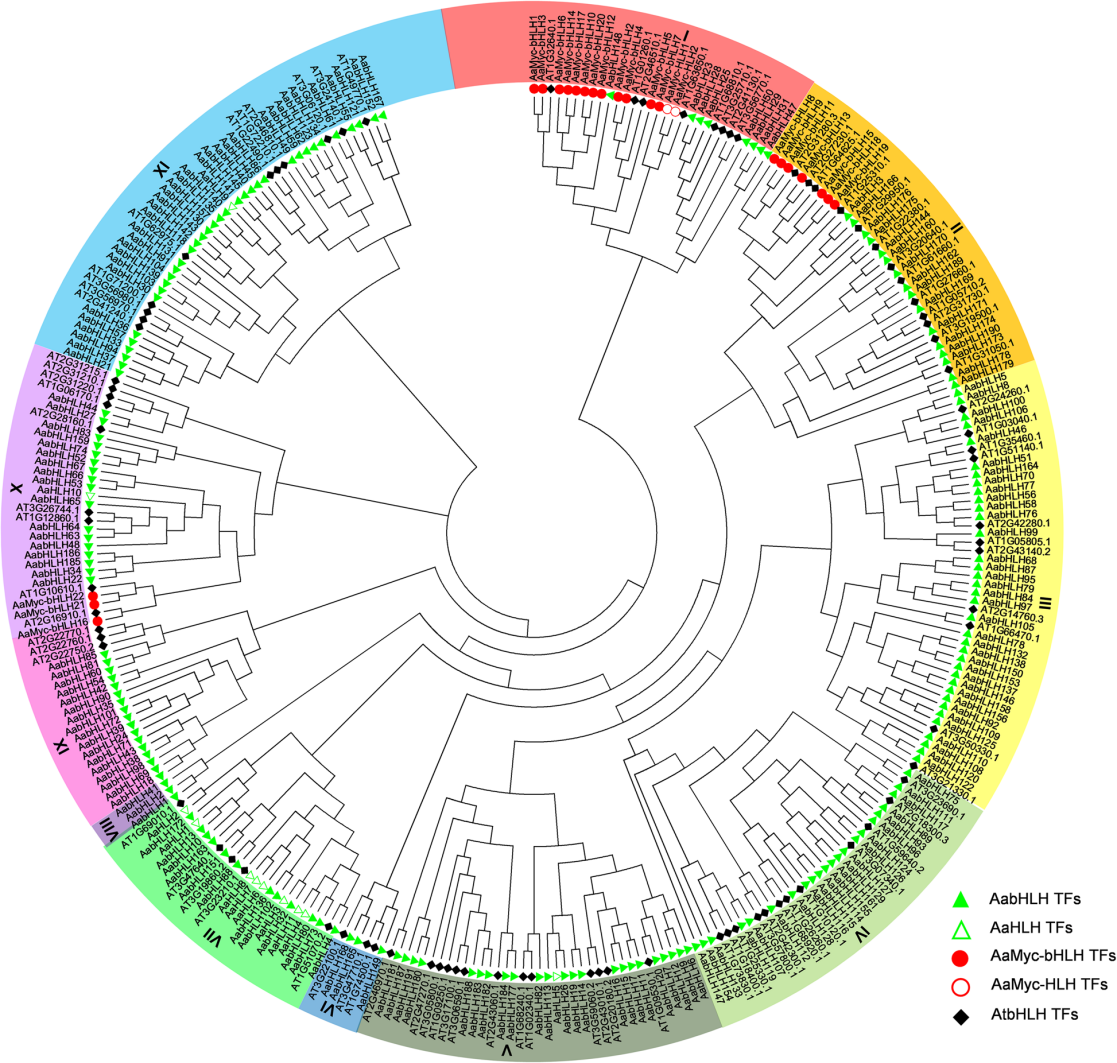
Phylogenetic tree of bHLH domain sequences from *Arabidopsis thaliana* (AT) and *A. annua* (Aa) proteins. All bHLH domains cluster into nine subclades (denoted by colour and numerals I–IX)

To classify the 226 bHLH TFs from *A. annua* and explore their evolutionary relationships with 88 Arabidopsis proteins, we constructed an unrooted phylogenetic tree based on their bHLH domains. The 314 TFs clustered into eleven subfamilies (Fig. 2). AaMyc-bHLH and AaMyc-HLH TFs were found in groups I, II, and X. AaHLH TFs mainly occurred in group VII, with a minor presence in groups V, X, and XI. AabHLH TFs were present in every group, while AtbHLH TFs were present in every group except VIII.

### Gene ontology classification of AabHLH TFs

Despite the sequences outside the bHLH domain being highly divergent, AabHLH TFs have highly conserved gene ontology (GO) annotations, especially with respect to Molecular Function (Table 2; Supplemental Material 1 Tables S1 and S2). Over 96% AabHLH TFs (217) possess dimerization activity; 86 have DNA binding activity; > 48% are involved in transcription processes; and 12 affect iron ion homeostasis. Several AabHLH TFs play a role in endosperm development and guard cell differentiation (Table 2). While there are only 11 AaHLH TFs (4.9% of the total), they are distributed across the more conserved GO annotations, including GO:0006355, GO:0046983, GO:0003700, GO:0055072, GO:0006357 and GO:0006351 (Supplemental Material 1 Table S2), indicating that a feedback regulation mechanism between AaHLH and AabHLH TFs may exist in *A. annua* biological processes.

**Table 2:** Gene ontology (GO) annotations of AabHLH TFs

GO	GO Term	Count	%	Description
Biological Process	GO:0006355	83	36.7	regulation of transcription, DNA-templated
	GO:0006357	22	9.7	regulation of transcription by RNA polymerase II
	GO:0055072	12	5.3	iron ion homeostasis
	GO:0006351	5	2.2	transcription, DNA-templated
	GO:0010052	5	2.2	guard cell differentiation
	GO:0009960	4	1.8	endosperm development
Molecular Function	GO:0046983	217	96	protein dimerization activity
	GO:0003700	86	38.1	DNA-binding transcription factor activity

### Protein–protein interaction network construction and hub gene identification

Protein interactions between the TFs were predicted with the STRING tool. A total of 227 nodes and 106 edges were identified in the protein-protein interaction (PPI) network; disconnected nodes in the network were hidden (Supplemental Material 1 Fig. S1). Nodes with higher degrees of connectivity tend to be more important for maintaining the stability of the entire network, so we focussed on identifying these hub genes, Cytoscape software was used to modify the PPI network.

**Fig. 3:**
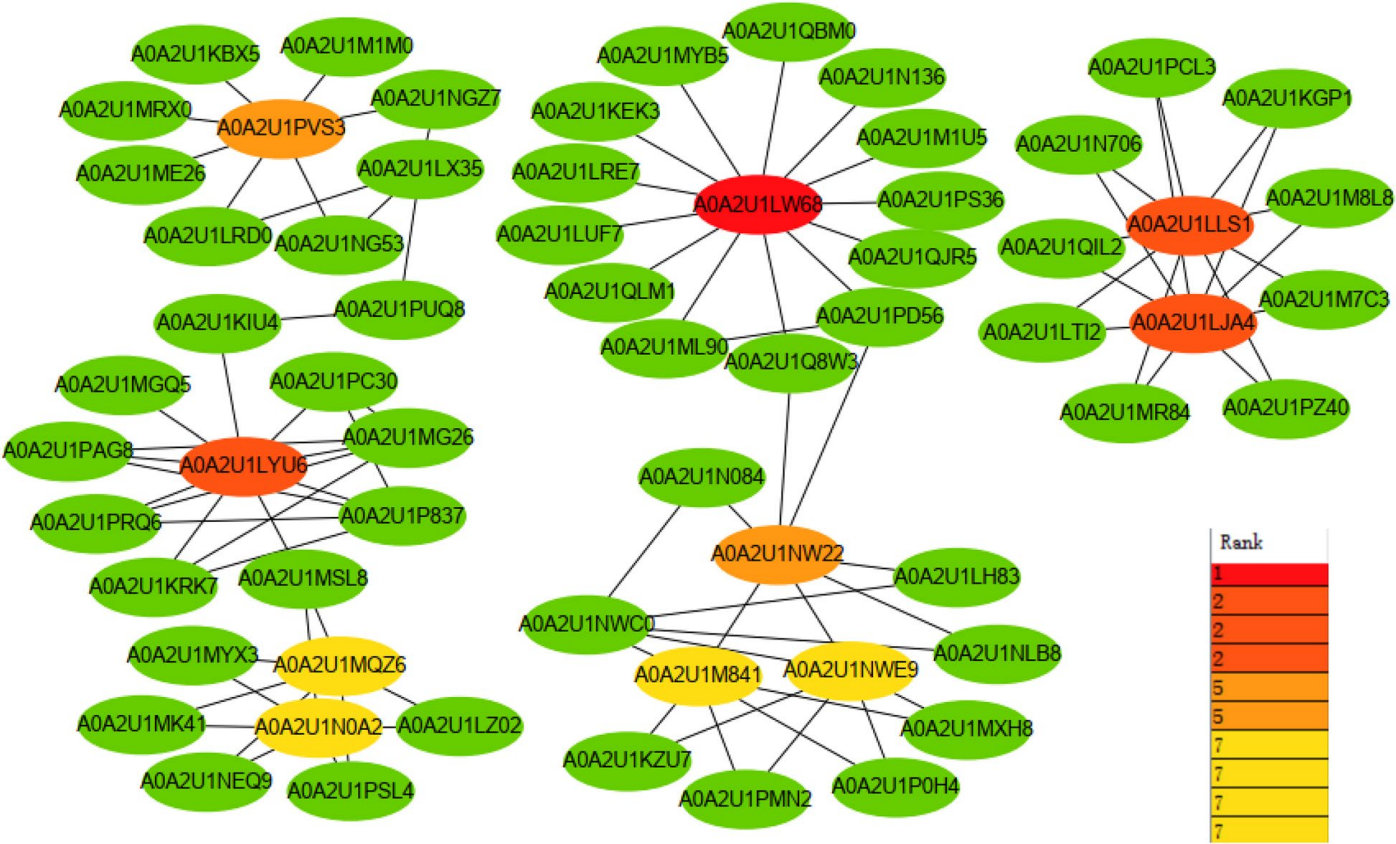
Modified protein–protein interaction (PPI) network based on *A. annua* bHLH proteins. The PPI network shows interaction relationship between bHLH proteins. Codes represent string names, and the non-green proteins are further described in Table 2

AabHLH61 had the highest degree of connectivity (26), followed by AabHLH20, AaMyc-bHLH3, and AaMyc-bHLH1, all with a degree of connectivity of 18 (Table 3; Fig. 3). The top ten proteins by connectivity in the PPI network were considered to be encoded by hub genes (Table 3).

**Table 3:** Top 10 hub proteins identified from the AabHLH TF PPI network

Gene	String Name	Annotation	Score
*AabHLH61*	A0A2U1LW68	Basic helix-loop-helix (BHLH) DNA-binding superfamily protein	26
*AabHLH20*	A0A2U1LYU6	Basic helix-loop-helix leucine zipper transcription factor	18
*AaMyc-bHLH3*	A0A2U1LLS1	Transcription factor MYC2	18
*AaMyc-bHLH1*	A0A2U1LJA4	MYC2 transcription factor	18
*AaMyc-bHLH9*	A0A2U1PVS3	Transcription factor	14
*AabHLH151*	A0A2U1NW22	Basic helix-loop-helix (BHLH) DNA-binding superfamily protein	14
*AabHLH100*	A0A2U1M841	Basic helix-loop-helix (BHLH) domain-containing protein	12
*AabHLH106*	A0A2U1NWE9	Basic helix-loop-helix (BHLH) DNA-binding superfamily protein	12
*AabHLH117*	A0A2U1MQZ6	Myc-type, basic helix-loop-helix (BHLH) domain-containing protein	12
*AabHLH111*	A0A2U1N0A2	Myc-type, basic helix-loop-helix (BHLH) domain-containing protein	12

**Fig. 4:**
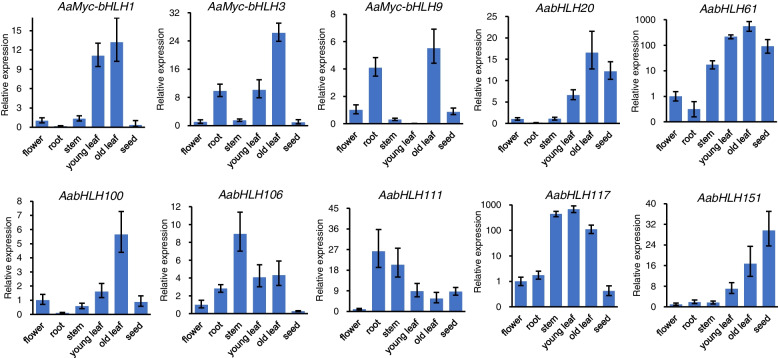
Expression levels of 10 *AabHLH* genes in *A. annua* vegetative and reproductive tissues. Results given as mean ± SD, *n* = 3. Gene expression relative to *actin* in the same tissue

The expression patterns of these genes were explored by quantitative reverse transcription (qRT-)PCR in flower, root, stem, young leaf, old leaf, and seed tissues (Fig. 4). All of these genes exhibited markedly different expression patterns in the six tissues analysed, suggest that these TFs play different functions in affecting various aspects of biological processes. *AaMyc-bHLH1* was highly expressed in young and old leaf, while *AaMyc-bHLH3* in old leaf. *AabHLH61* and *AabHLH117* expression were highest in leaf tissues, as well as seed for *AabHLH61* and stem for *AabHLH117*. *AaMyc-bHLH9* and *AabHLH100* expression also peaked in old leaf, it was at lower levels. Of the remaining hub genes, *AabHLH20* was highly expressed in old leaves and seeds; *AabHLH106* in the stem; *AabHLH111* in roots and stem; and *AabHLH151* in seeds.

### Differential expression of AabHLH TFs in various tissues

**Fig. 5:**
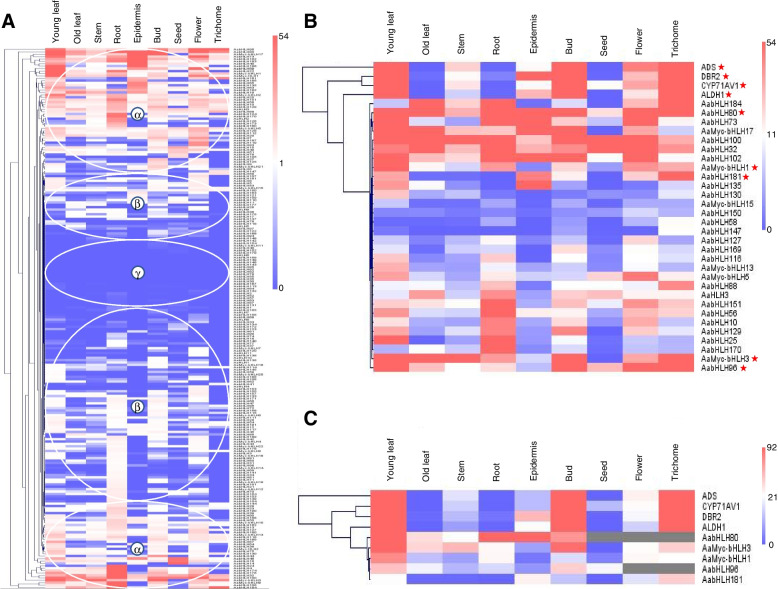
Expression of genes encoding AabHLH TFs across *A. annua* tissues and stages of development. **A** Hierarchical clustering of expression levels in different tissues of all *AabHLH* genes. α, highest expression level; β, low expression level; γ, almost no expression. **B** Hierarchical clustering of expression levels of *AabHLH* genes that encode key enzymes involved in artemisinin synthesis. Asterisks denote genes in B highly expressed in trichomes, shown in **C**

An existing RNA-sequencing (RNA-seq) database was used to further explore the expression patterns of 226 AabHLH TFs at different growth stages in different tissues and organs (young leaf, old leaf, stem, root, epidermis, bud, seed, flower and trichome) [32]. Three obvious clusters (labelled α, β, and γ) of expression were detected (Fig. 5A; Supplemental Material 1 Fig. S2). Expression of genes encoding AabHLH TFs was highest in the α clusters, with most genes exhibiting mid- to high-expression levels; in the β clusters, gene expression was generally lower. Across all four clusters, however, different patterns of tissue-specific expression were observed, e.g., in β, genes were generally most highly expressed in root, bud, and flower. The expression levels of AabHLH TFs from the γ cluster were generally very low across all tissues (Fig. 5A).

Trichomes (small protrusions of epidermal origin on stem, leaf, bud, and flower surfaces) of *A. annua* are the sites for production and storage of artemisinin [33, 34]. Genes encoding key enzymes in artemisinin synthesis are also highly expressed in trichomes, e.g., *ADS*, *DBR2*, CYP71AV1, and *ALDH1* (Fig. 5B). To define which AabHLH TFs might be involved in trichome formation and artemisinin synthesis, we identified AabHLH TF-encoding genes with relatively high expression in the trichome. The expression levels of *AaMyc-bHLH1*, *AaMyc-bHLH3*, *AabHLH184*, *AabHLH80*, *AabHLH181*, *AabHLH88*, and *AabHLH96* in trichome were comparable to those encoding key artemisinin synthetic enzymes (Fig. 5B). Moreover, *AabHLH80*, *AabHLH96*, *AabHLH181*, *AaMyc-bHLH1*, and *AaMyc-bHLH3* were also highly expressed in bud and young leaf (Fig. 5C), consistent with patterns exhibited by genes encoding artemisinin synthetic enzymes, suggesting that the encoded bHLH TFs may be involved in artemisinin synthesis.

## Discussion

### Comprehensive genome-wide detection of AabHLH TFs

Our research identified 226 AabHLH TFs in *A. annua* (Table 1), which slightly exceeds the 205 found in a previous study [24], likely due to differences in screening methods. Multiple sequence alignments of full-length AabHLH TF sequences showed that almost all TFs contained the classic bHLH domain, which is similar to domains in maize [30], tomato [35], and barley [36]. Some TFs lacked the N-terminal basic motif; these TFs cannot bind DNA, so play a negative regulatory role. For example, PAR1–PRE1 and PAR1–PIF4 heterodimers in Arabidopsis form a complex HLH/bHLH network regulating cell elongation and plant development in response to light and hormones [11]; bHLH TF GhFP2 and HLH TF GhACE1 antagonistically regulate fibre elongation in cotton [37]; and antagonistic HLH/bHLH TFs mediate brassinosteroid regulation of cell elongation and plant development in rice and Arabidopsis [38].

### GO annotation analysis

AaHLH TFs are annotated with 8 conserved GO terms (Table 2), particularly dimerisation, DNA-binding, and transcription processes, consistent with typical functions of bHLH TFs [39, 40]. This family of TFs have been reported to be involved in iron ion regulation in tomato [41] and Arabidopsis [42]; here, 12 *A. annua* bHLH TFs were annotated with a GO term implicating a role in iron homeostasis. Other roles for AabHLH TFs suggested by GO annotations, such as in endosperm development and guard cell differentiation, have been reported in other plants [43–46], indicating that the functions of bHLH TFs from different species are conserved.

Further, AaHLH TFs without basic motifs were distributed across the conserved GO annotations (Supplemental Material 1 Table S2), suggesting a potential role for these TFs in feedback mechanisms with AabHLH TFs across a broad range of biological processes.

### Potential function of AabHLH TFs in artemisinin biosynthesis and trichome development

AaMyc-bHLH3 has been reported to bind to the E-box motif of ADS and CYP71AV1 to positively regulate artemisinin biosynthesis in *A. annua* (annotated AabHLH1 in [23]). *AaMyc-bHLH3* is generally more highly expressed than other AabHLH TFs in young leaf, bud and trichome (Fig. 5B). Genes encoding AaMyc-bHLH1, AaMyc-bHLH3, AabHLH80, AabHLH181, and AabHLH96 showed similar expression patterns, suggesting that they may also be involved in trichome development and artemisinin regulation (Fig. 5B, C). Furthermore, AaMyc-bHLH1and AaMyc-bHLH3 also being hub genes, this also reflects the important role of both in the growth and development of *A. annua*.

In the well-studied model *Arabidopsis thaliana*, trichome initiation is regulated by two protein complexes. The first one, the activator–depletion multimer GL1/MYB23-GL3/eGL3-TTG1, forms a MYB-bHLH-WD40 complex that binds to the *GLABRA2* (*GL2*) promoter to positively regulate trichome development. The second one, the activator–inhibitor multimer MYB-bHLH-TTG, negatively regulates trichome formation by replacing the activator GL1/MYB23 with the inactive TRY/CPC-GL3 in a complex with eGL3-TTG1 [47, 48]. Previous studies in *A. annua* have identified a MYB23 homologue, *AaTAR2*, which encodes an R2R3 MYB TF expressed mainly in young leaves. Inhibition or overexpression of *AaTAR2* resulted in decreased or increased artemisinin content in glandular secretory trichomes (GSTs), respectively, as well as changes in GST morphology [34]. Another gene encoding an R2R3 MYB TF, *AaMIXTA1*, is mainly expressed in the basal cells of GSTs; again, its overexpression or inhibition resulted in an increase or decrease in GST numbers and artemisinin content in transgenic plants, respectively [49]. While these MYB TFs have been identified, no related bHLH TFs have been reported to regulate trichome initiation and development, as would be expected if the process is conserved with other plants. This new bHLH TF resource can be used to guide further research to uncover the molecular mechanisms underlying GST development in *A. annua*, and to identify specific bHLHs that may be involved in regulatory complexes.

## Conclusions

At last, this comprehensive analysis of bHLH TFs provides a new resource to direct further analysis into key molecular mechanisms underlying and regulating artemisinin biosynthesis and trichome development, as well as other biological processes, in the key medicinal plant *A. annua*.

## Methods

### Defining *A. annua* bHLH TF amino acid sequences

The *A. annua* genome, protein database, and annotation files were downloaded from NCBI (National Center for Biotechnology Information), ID: PRJNA416223 [32]. A local protein database was constructed with NCBI BLAST software (ncbi-blast-2.9.0 + −win64). An HMM (Hidden Markov Model) profile of the HLH conserved PF00010 domain was downloaded from http://Pfam.xfam.org/; this file was used as seed for Hmmer software [50] to run an HMMsearch in the local protein database (E-value 0.01). In parallel, 88 bHLH protein sequences from Arabidopsis were acquired from TAIR (The Arabidopsis Information Resource) database (https://www.arabidopsis.org/) [26]; these bHLH were also used as query sequences in a local BlastP search on the *A. annua* protein database (E-value 0.0001). The resulting sequences were combined, and redundant sequences removed with CD-HIT (http://www.bioinformiscs.org/CD-HIT/). The remaining 226 sequences were analysed with HMMscan (https://www.ebi.ac.uk/Tools/hmmer/search/hmmscan), and bHLH domains were determined with NCBI Conserved Domains (https://www.ncbi.nlm.nih.gov/Structure/cdd/wrpsb.cgi). Proteins containing Myc domains were identified by the presence of a PF00249 domain.

### Analysis of AabHLH domains

The AabHLH sequences were aligned with MEGA software 6.06 [51]. Conserved amino acids were identified and characterised with Weblogo (http://weblogo.berkeley.edu/), while Swiss-Model (https://swissmodel.expasy.org/) was used to predict the three-dimensional structure of the bHLH domain.

### Phylogenetic analysis

The neighbour-joining phylogenetic tree of bHLH domain sequences from Arabidopsis (88) and *A. annua* (226) was constructed using Clustal X2 [52] with a bootstrap test of 1000 replicates. MEGA 6.06 was used to modify the phylogenetic tree.

### GO analysis of AabHLH TFs

As *A. annua* is not included in the standard Gene Ontology (GO) Database for Annotation, Visualization, and Integrated Discovery (DAVID), we individually analysed 226 AabHLH TFs with InterPro (http://www.ebi.ac.uk/interpro/) to determine GO terms associated with each protein.

### PPI network construction and hub gene identification

To evaluate potential PPI relationships, the 226 AabHLH TFs were mapped to the STRING database (Search Tool for the Retrieval of Interacting Genes, http://string-db.org/), and PPI pairs with a combined score ≥ 0.4 were extracted. The PPI network was visualised with Cytoscape software (www.cytoscape.org/). CytoHubba, a Cytoscape plugin, was used to calculate the degree of connectivity for each protein node. The top ten genes were selected as hub genes.

### Gene expression analysis

The *A. annua* “Huhao 1” used in this article is a high artemisinin producer and was cultured at Naval Medical University for several years. The seeds of *A. annua* was stored at 4 °C, germinated on the Murashige and Skoog (MS) medium with 3% sucrose and 0.7% agar, then the plants with 2 leaves were transferred to soil (black soil: vermiculite: perlite about 10:10:1) and cultivated in a greenhouse with a relative humidity of 70%, a photoperiod of 16-h light (23 °C) /8-h dark (20 °C). Roots were obtained from 10 days old plant. Stem, leaves and bud were collected from 4 months old plants as previously described [32]. Total RNA was isolated with the TRIZOL Reagent (TRANS) from nine tissues collected from three independent plants: young leaf; old leaf; stem; root; epidermis; mature seed; flower; and trichomes. cDNA was synthesised from 4 mg of total RNA with Hifair® III reverse transcriptase (Hifair® III 1st Strand cDNA Synthesis Kit; YEASEN) according to manufacturer’s instructions.

Quantitative reverse transcription (qRT)-PCR was performed using QuanStudio 3 (Thermo Fisher Scientific) with the PerfectStart® Green qPCR SuperMix (TRANS). *Actin* (EU531837) was used as an internal control. For qRT-PCR assays, cDNA was denatured at 94 °C for 30 sec, followed by 45 cycles of 95 °C 5 s, 54 °C 15 s, and 72 °C 10s. Assays were performed in triplicate. Primers used for qRT-PCR are listed in Supplemental Material 1 Table S3.

### Analysis of *AabHLH* gene expression across tissues and stages of development

*A. annua* transcriptomics data was downloaded from NCBI (PRJNA416223) [32]. mRNA sequences were extracted with TBtools software [53], using Salmon software to build the index, and TPM (transcripts per million, normalised for gene length) values calculated [54]. Results were imported into MEV4.9.0 software [55] to generate heat maps and perform hierarchical clustering.

### Seeds access and culture

*A. annua* is a widely grown plant. The seeds of the “Huhao 1” cultivar line were obtained from Shanghai Jiao Tong University [32], deposited in our university seed bank and are freely accessible for research. The seeds were preserved, cultivated, and propagated in Naval Medical University (30^°^ N 121^°^ E) from April to November (the natural growing season) according to standard local practice. The seeds deposit information is as follows: ID: Huhao 1. Contact person: Prof Hexin Tan, department of pharmacy, Naval Medical University, 325 Guohe Road, Shanghai 200,433, China, Email: hexintan@163.com.

## Availability of data and materials

Genome sequences were from (https://www.ncbi.nlm.nih.gov/Traces/wgs/PKPP01?display=contigs&page=1); Local protein database was from (https://www.ncbi.nlm.nih.gov/Traces/wgs/PKPP01?display=proteins&page=1); *A. annua* transcriptomics data was downloaded from NCBI (PRJNA416223), for young leaf RNA-seq data from (https://trace.ncbi.nlm.nih.gov/Traces/?view=run_browser&acc=SRR6472941&display=download); old leaf (https://trace.ncbi.nlm.nih.gov/Traces/?view=run_browser&acc=SRR6472942&display=download); stem (https://trace.ncbi.nlm.nih.gov/Traces/?view=run_browser&acc=SRR6472943&display=download); root (https://trace.ncbi.nlm.nih.gov/Traces/?view=run_browser&acc=SRR6472944&display=download); epidermis (https://trace.ncbi.nlm.nih.gov/Traces/?view=run_browser&acc=SRR6472945&display=download); bud (https://trace.ncbi.nlm.nih.gov/Traces/?view=run_browser&acc=SRR6472946&display=download); seed (https://trace.ncbi.nlm.nih.gov/Traces/?view=run_browser&acc=SRR6472947&display=download); flower (https://trace.ncbi.nlm.nih.gov/Traces/?view=run_browser&acc=SRR6472948&display=download); trichome (https://trace.ncbi.nlm.nih.gov/Traces/?view=run_browser&acc=SRR6472949&display=download).

## Supplementary Information

**Additional file 1: Table S1.** All Gene ontology (GO) annotation of AabHLH TFs involved into. **Table S2.** All AabHLH TFs are divided into different categories. **Fig. S1.** Protein–protein interaction (PPI) network. **Fig. S2.** Expression of genes encoding total AabHLH TFs across *A. annua* tissues and stages of development. **Table S3.** qRT-PCR primers.

**Additional file 2: Supplemental Material 2.** cDNA sequences of 226 bHLH TFs.

**Additional file 3: Supplemental Material 3.** gDNA sequences of 226 bHLH TFs.

**Additional file 4: Supplemental Material 4.** 226 TFs protein sequences.

## Data Availability

Transcriptomic data of *A. annua* at various developmental stages and in different tissues was obtained from a previous study [32]. All other data generated or analysed during this study are included in this published article or its supplemental material.

## Abbreviations

bHLH: Basic helix-loop-helix
TF: Transcription factor
SD: Standard deviation
qRT-PCR: Quantitative reverse transcription polymerase chain reaction
