# Supplementary material for: Genome-wide identification and characterisation of bHLH transcription factors in Artemisia annua
Source: BMC Plant Biol. 2023 Feb 1;23:63. doi: 10.1186/s12870-023-04063-8 (PMC9890702; doi:10.1186/s12870-023-04063-8)
Supplement: Supplementary file 1 — Additional file 1: Table S1. All Gene ontology (GO) annotation of AabHLH TFs involved into. Table S2. All AabHLH TFs are divided into different categories. Fig. S1. Protein–protein interaction (PPI) network. Fig. S2. Expression of genes encoding total AabHLH TFs across A. annua tissues and stages of development. Table S3. qRT-PCR primers. [file 12870_2023_4063_MOESM1_ESM.docx]

**Table S1** All Gene ontology (GO) annotation of AabHLH TFs involved into.

| GO | GO-Term | Description |
| --- | --- | --- |
| BP | GO:0006355 | regulation of transcription, DNA-templated |
| MF | GO:0046983 | protein dimerization activity |
| MF | GO:0003700 | DNA-binding transcription factor activity |
| BP | GO:0048364 | root development |
| CC | GO:0005634 | nucleus |
| BP | GO:0006396 | RNA processing |
| MF | GO:0004525 | ribonuclease III activity |
| BP | GO:0055072 | iron ion homeostasis |
| BP | GO:0006351 | transcription, DNA-templated |
| BP | GO:0006357 | regulation of transcription by RNA polymerase II |
| BP | GO:0048658 | anther wall tapetum development |
| BP | GO:0006094 | gluconeogenesis |
| MF | GO:0005524 | ATP binding |
| MF | GO:0004612 | phosphoenolpyruvate carboxykinase (ATP) activity |
| MF | GO:0017076 | purine nucleotide binding |
| MF | GO: 0004611 | phosphoenolpyruvate carboxykinase activity |
| BP | GO:0009960 | endosperm development |
| BP | GO:0010052 | guard cell differentiation |
| BP | GO:0006508 | proteolysis |
| MF | GO:0008234 | cysteine-type peptidase activity |
| BP | GO:0006413 | translational initiation |
| MF | GO:0003743 | translational initiation factor activity |
| MF | GO:0003723 | RNA binding |
| MF | GO:0003824 | catalytic activity |
| MF | GO:0004252 | serine-type endopeptidase activity |
| CC | GO:0016021 | Integral component of membrane |
| MF | GO:0043565 | sequence-specific DNA binding |

BP: biological process, MF: molecular function, CC: cellular component.

**Table S2** All AabHLH TFs are divided into different categories.

| GO-term | AabHLH TFs | | |
| --- | --- | --- | --- |
| O:0006355 | AaMyc-bHLH (1, 2, 3, 4, 5, 6, 8, 9, 10, 11, 12, 15, 17, 18, 19, 20)  AabHLH(4, 20, 23, 25, 27, 28, 29, 31, 32, 44, 47, 50, 55, 73, 75, 78, 80, 89, 92, 93, 96, 102, 107, 108, 109, 110, 111, 112, 114, 115, 116, 117, 119, 120, 121, 122, 124, 125, 126, 127, 128, 129, 133, 136, 147, 148, 152, 155, 161, 165, 167, 168, 177, 178, 180, 181, 182, 183, 184, 187, 188, 191), AaHLH (3, 4, 6, 7, 11) | | |
| GO:0046983 | AaMyc-bHLH (1, 2, 3, 4, 5, 6, 7, 8, 9, 10, 11, 12, 13, 14, 15, 16, 17, 19, 20, 21, 22) *AaMyc-HLH (1, 2),*  AabHLH (1-191) except AabHLH (181, 184, 185, 186, 188), AaHLH (1, 2, 3, 4, 5, 6, 7, 11) | | |
| GO:0003700 | AaMyc-bHLH (1, 2, 3, 4, 5, 6, 8, 9, 10, 11, 12, 13, 14, 15, 17, 18, 19, 20)  AabHLH (4, 10, 13, 20, 21, 23, 25, 28, 29, 30, 31, 32, 33, 36, 37, 47, 50, 55, 57, 59, 61, 62, 73, 78, 80, 88, 91, 92, 94, 102, 103, 104, 108, 109, 110, 118, 120, 121, 122, 123, 125, 130, 131, 134, 135, 136, 139, 140, 141, 142, 143, 145, 148, 152, 157, 165, 167, 168, 172, 178)  AaHLH (1, 2, 3, 4, 6, 7, 9, 11) | | |
| GO:0006357 | AabHLH (21, 30, 33, 36, 37, 57, 91, 94, 103, 104, 118, 130, 131, 135, 139, 140, 141, 142, 143, 145, 157), AaHLH9, | | |
| GO:0055072 | AabHLH (4, 32, 73, 80, 88, 102, 136), AaHLH (3, 4, 6, 7, 11) | | |
| GO-term | AabHLH TFs | GO-term | AabHLH TFs |
| GO:0048364 | AaMyc-bHLH13 | GO:0009960 | AabHLH (55, 121, 152, 167) |
| GO:0005634 | AaMyc-bHLH13 | GO:0010052 | AabHLH (59, 61, 62, 123, 134) |
| GO:0006396 | AaMyc-bHLH14 | GO:0006508 | AabHLH72, |
| GO:0004525 | AaMyc-bHLH14 | GO:0008234 | AabHLH72, |
| GO:0006351 | AabHLH (10, 13, 172), AaHLH (1, 2) | GO:0006413 | AabHLH154, |
| GO:0048658 | AabHLH(27, 44) | GO:0003743 | AabHLH154, |
| GO:0006094 | AabHLH 49, | GO:0003723 | AabHLH154, |
| GO:0005524 | AabHLH (49, 155) | GO:0003824 | AabHLH156, |
| GO:0004612 | AabHLH49, | GO:0004252 | AabHLH158, |
| GO:0017076 | AabHLH49, | GO:0016021 | AabHLH158, |
| GO:0004611 | AabHLH49, | GO:0043565 | AabHLH178, |

Black font stands for AabHLH, font with dotted underline represents AaMyc-bHLH, italics font represents AaMyc-HLH, font with underlined represents AaHLH.


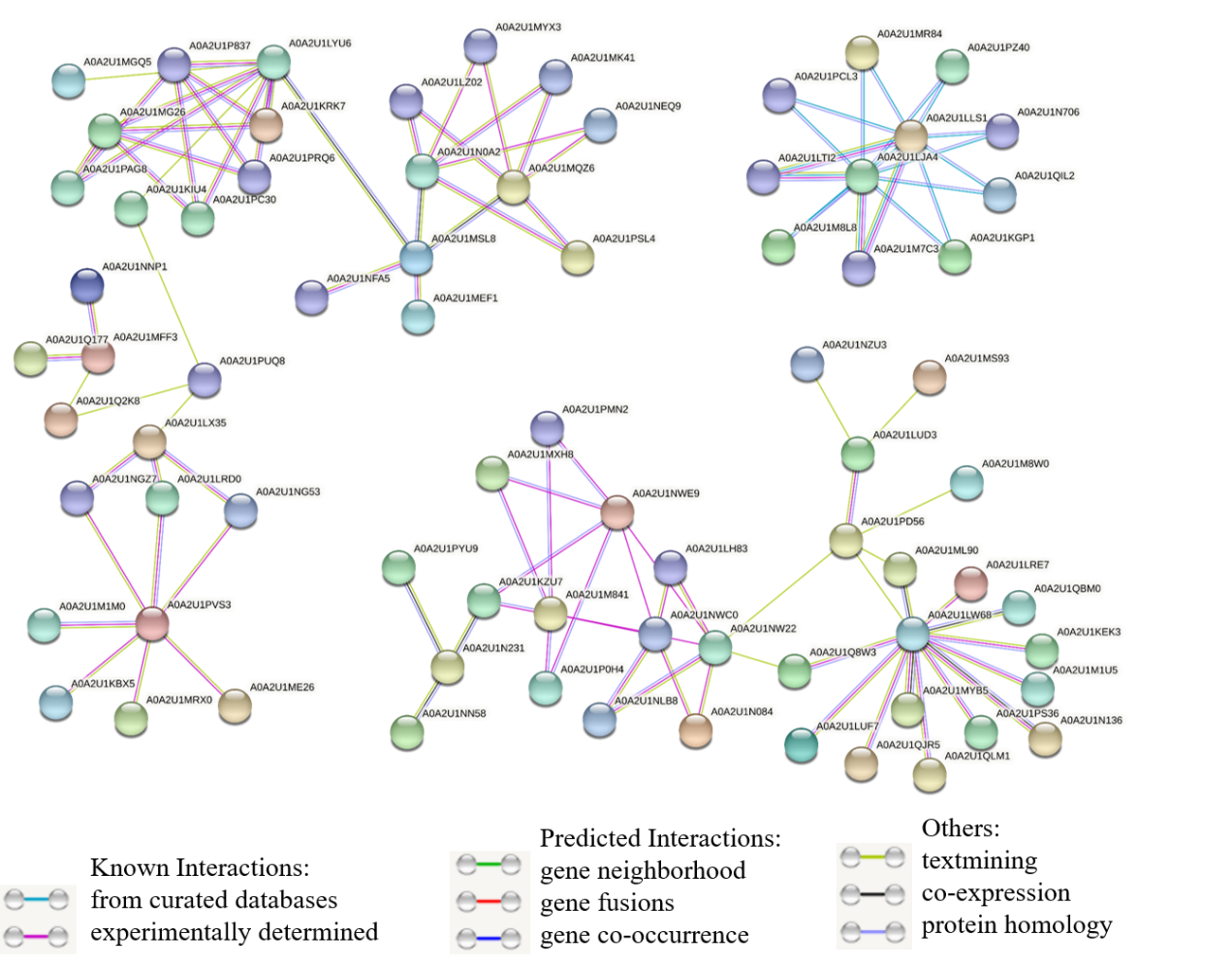


**Fig. S1** Protein–protein interaction (PPI) network.

PPI network was constructed with the AabHLH TFs by STRING tool, and PPI pairs with a combined score ≥0.4 were extracted.


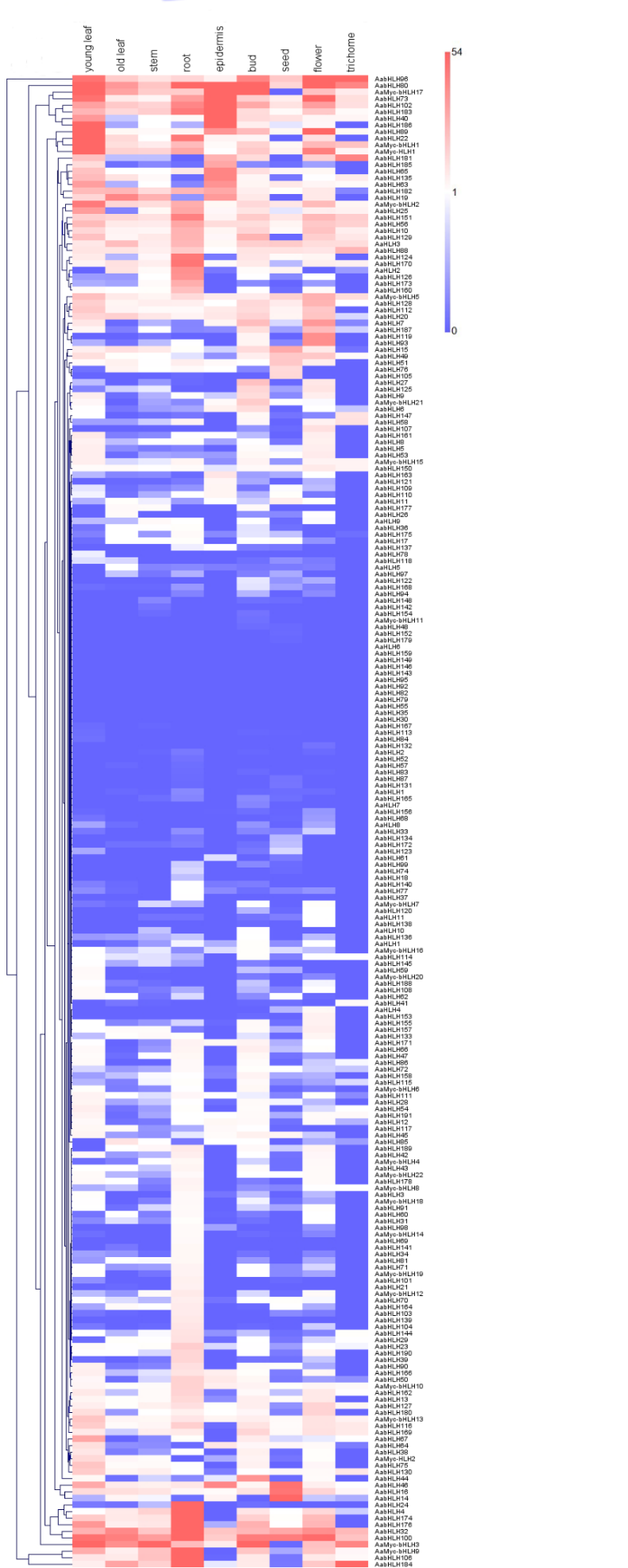


**Fig. S2** Expression of genes encoding total AabHLH TFs across A. annua tissues and stages of development.

Expression data of 226 AabHLH TFs were collected by MEV4.9.0 software to generate heat maps and perform hierarchical clustering.

**Table S3** qRT-PCR primers

| Primer Name | Sequences (5′-3′) |
| --- | --- |
| ActinS | CCAGGCTGTTCAGTCTCTGTAT |
| ActinA | CGCTCGGTAAGGATCTTCATCA |
| AaMyc-bHLH1S | GGTTAATACCGTGGAAATGAAA |
| AaMyc-bHLH1A | TTCACTAGCTCAACAGATTCCG |
| AaMyc-bHLH3S | GGGTTCGGGTTACAGACTATT |
| AaMyc-bHLH3A | CATCTCAGATCCAATAACTCCAA |
| AaMyc-bHLH9S | AAGCATTCAACTACAAGTATCAGG |
| AaMyc-bHLH9A | AATTGGACCGTTGAGTTAGTTG |
| RTbH20S | TGCTACTTCTTCTGTTTGCTCA |
| RTbH20A | TTTAGAACCTGTACCTCCACGA |
| RTbH61S | ATTGCCGCACTTGAAGATTT |
| RTbH61A | TTTGCTATGTCTTCTGCTGAGA |
| RTbH100S | CCCATCCACCTAGTATTCGTC |
| RTbH100A | CTGGGAACAAGTTCCTGTAAAG |
| RTbH106S | GTTCGTCCTACAAGGGTCAGA |
| RTbH106A | TGTAATGCTCTGATTCTCTCTGC |
| RTbH111S | TGTGTCACCTAAGAAAAGGAAAG |
| RTbH111A | CTGTTGCTTTGTCACTGTTGTTA |
| RTbH117S | TCAAAATCAAATGTTGGAGAAGA |
| RTbH117A | TCCTGCAAATATTTCATTCTCTC |
| RTbH151S | CTTTTTCTTGAGTTGGCTGGT |
| RTbH151A | GTGATTCGGACAGTAAAGCTG |
