# Supplementary material for: Genome-wide identification and characterisation of bHLH transcription factors in Artemisia annua
Source: BMC Plant Biol. 2023 Feb 1;23:63. doi: 10.1186/s12870-023-04063-8 (PMC9890702; doi:10.1186/s12870-023-04063-8)
Supplement: Supplementary file 2 — Additional file 2: Supplemental Material 2. cDNA sequences of 226 bHLH TFs. [file 12870_2023_4063_MOESM2_ESM.docx]

**Supplemental Material 2 cDNA sequences of 226 bHLH TFs**

AaMyc-bHLH1

ATGACGATGAATATATGGAATTCAGATGATAACGCTATGATGGATGCTTTTATGAGCTCCGATATGTTTTCTATATGGGGAACTCCAGCAACCCCAGCTGGAGCTCCAGCTGGGCCAGGACTAGTTGTTCCACCAGCTTCTTCCTCTGCTTCAACTTCTGCCGCGATTGGCAGCGAGTGGAATCAGGATACTTTGCAACAACGTCTCCAAGGATTGATTGATAACGCGCGTGAGTCTTGGACTTACGCGATATTCTGGCAGTCTTCTGGAGTGGATTACACAGCTCCTTCTGTGTTGGGGTGGGGTGACGGGTATTACAAAGGAGAGGTTAATAAACCCAAAACTGTAGAATCTGTTACCTCTTTGGCTGAGCAAGAGCATAGGAAGAAAGTGTTGAGGGAGCTGAATTCGTTGATTTCGGGTTCTCATAGCCAAGAAAATGAAACTATTGATGAGGAAGTTACTGATACAGAATGGTTTTTTCTTATTTCTATGACGCAGTCTTTTGTTAATGGACATGGGTTGCCAGGTCAAGCTATGTTTAGTAATCAGCCTGTTTGGATTACCGGGCGGGACAGATTGTCGGTGTCGCACTGCGAGCGTGCGAGACAAGGTCAGGTATTCGGAATACAGACTATTGTCTGTTTACCATCTAGTAATGGAGTTGTCGAACTAGGTTCGACGGAGTTAATCTTTCAAAGCTTAGATCTGATTAATCAGGTTAAGGTTTTGTTTAATTTTGGTAATAGTCCTCCAGATTTGGCGCCACTAGACGCGGATGATCAGGATCAGAATACTGATCCTTCGTCCATGTGGCTAAATGACCCACCTCCGGTGTCTTCTGGTACCACGGTTAATACCGTGGAAATGAAAGATACCGGAGATGTAAGGGCAGTTGTTCCTCCTAAGGAAACGAGTGTTGTCCCATTAAACAACTCGGTTCATACTGAAAATACTACTAAGTCTGTTGTTAATAATTCCAATCATCAAGGATTGTCTGGCAGCCGAGAATTGAATTTTTCTTCGGGGTTTGGGTGTAAGCCGGAATCTGTTGAGCTAGTGAATTTTGGTGAGAATAATAAGAAGAAGAAAGCCCCTGCTCTACGTGGTATTAATGAAGGGGGGATGATGTCATTTAGCTCTGGTATGGTTGTTCCTCCATCTGATACTGTTAAATCTGGGGGTGTAATGAGTGCAGCGGACTTTGACCAATCAGATATTGAAGCATCAATCGGAAGAGAGGTGGAGAGCCGATTAGTGGTGGAACCGGAGAAAAAGCCACGAAAACGAGGTAGGAAACCGGCAAATGGTCGAGAAGAGCCATTGAATCATGTGGAAGCCGAGAGGCAAAGAAGAGAAAAACTAAATCAGAAATTTTACGCTCTTCGTGCAGTTGTCCCAAATGTCTCGAAAATGGACAAAGCATCTCTTCTAGGAGATGCGATTTCTTACATCAACGACCTCAAATCAAAGCTTGACAGCACAACAACAGATAAAGAAGAACTGAAAGACCAACTCGACGCAATGAAGAAAGAGTTGTTATCAAAAGACTCTCATCAATCATCTTCCTCTACAGTCTCACCACCCGAAGATCTAATGACACCTAATCCAAGTAACCCGATATTAAATGATTTAGACATAGATGTCAAGATCATTGGGTGGGACGCCATGCTTAGGATCCAAAGTGGCAAGAAAAACCATCCTTCTGCACGCCTAATGGCGGCGTTAAAGGATCTTGATCTTGAAGTACACCATGCCAGTGTATCAGTCGTGAATGAATTGATGATGCAACAAGCTACAGTTAAAATGGGTAGTCGATTTTACACTGAAGATCAACTTCGAATAGCTTTGACAAACAGAATGTCAGATCCTAGGTAA

AaMyc-bHLH2

ATGAGTATTGAAAGTTTTAATGATGAGGATAAAGCAATAGTTGCATCAGTGTTAGGGACTAAGGCTTATGATTATTTAATATCGAGTTCGGTTACTAATGAATCTTTGTTAACTTCATTAGCTAGTAATGATGATAATTTGCAAAATAAGCTATCAGATCTAGTGGAGAATGTTAGTTTAGGTAACTTTAGTTGGAATTACGCCATTTTTTGGCAGATTTCGCGGTCGAAAACAGGGGAGTTGGTTTTGGTTTGGGGGGATGGGTGTTGTAGGGAGCCTAGAGAAGGGGAGGAGTTTGATATTGCGCGGATATTGAGTATTCGTCTAGAAGATGAGAATCAACAGAGGGTGAAGAAACGGGTTTTGCAGAAATTGCATGTTTTGTTTGGGGGGTTGGATGAGGATAATTATGCTTTTGGATTGGATAGAGTTACTGATACTGAGATGTTCTTTTTGATATCTATGTATTTTTCCTTTCCGCAAGGGCAAGGTGGTCCCGGGAAATGTTTTTCGTCCGGTAAGCATTTTTGGTATTCTGATGCTTTGAAGTCGAGTTCTGATTATTGTTTCCGGTCGAATTTGGCCAAGTCTGCTGGTATTCAGACGGTTGTTTTGGTTCCTACTGATGGTGGGGTAGTTGAGGTTGGTTCCATTCGTTCTATTCCTGAAAATATGGACCTTTTACATTCCGTGAGATCATCCTTTTCGTTAAAGCCAAATAATGGTTTAGTGGCTGCGGCTCCATTGATGGGTAATGCACAATTATCGAGTGCGTTGATTGGGGAGAGAAAAAACGAAAATGGCCATGGCGGGCATTTTTTGGATTTAGGTCTTGTTGATCATCAACTTAAGGCGTCTAAGGTTGTTCGACAAGATATGGGCTTGAGTTTGCGGCAGCCTCAATTTAGGGAAAAACTTGCAGTTAGGAAAGCAGAGGAGCCACGGTCTCCTTGGGAAGGGTATCCTGTTACTAATTCCCGACTCCTGGCTTCAAATACTAGAAACAGAATAACTGGTTCGAATTGGGGACAGTTCACTAGTCCCCAAGAGGAGTTCCAACTTAACAGTTTTAGGCCTCAAAAGTCACCAACAGAAATGCAAATTGACTTCACGGGTGCTGTTTCTCGTCCTTCAGTAGTTTCTCGGCCAGTTAGTGGTGACTCTGAGGCATCTGATGTGGAAGCTTCAGGCAGGGATGAAAGAGCTGTCCTAACTGGTTTAACAGATGATAAACGGCCTCGTAAAAGGGGCCGAAAGCCTGCTAACGGAAGGGAAGAGCCGCTGAATCATGTTGAAGCAGAGAGACAAAGAAGAGAGAAGCTGAACCAGAGGTTTTATGCTTTACGAGCGGTTGTTCCCAACATCTCAAAGATGGACAAAGCTTCACTGTTGGGAGATGCAATCACTTACATAACCGACCTTCAGAAGAAGCTCAAGGAGATGGAATCCGAAAGAAGTGGTTCACACGGAAGCACTTCTATGGAAACACCCAACAACAGTAACAACGGGTCAAGTTTAGAGAAAATCGAAATTGAAGCAGATAAAGATCAAGTAACTGTTAGAGTAAGCTGTCCGGTAGATACACACCCTATATCTAAGGTTATCCAAGCATTCAAAGAAGCTCAGATAAGAGTCGTTGATTCAAAAATGGCTGCAGCAAACGACAAAGTGTTTCACATATTCGTCATCAAGTCTCAAGGACCGGAACAACTGACAAAAGAGAAGTTGATGGCTGTGTTTTCAAAGGAATCAAGCTCCTCTTTAAACTCATTACCATAA

AaMyc-bHLH3

ATGACGGAGTACCGCATGAATCACTGGACTCCCGACGAGAACGCGTCCATGCTGGACGCGTTCGACATGTCATCTATCTGGGCTAATAACAGTAACCAAACTCAAACTGTCCCTCCTACTTCATCATCAGCTTCCACGTCAACTGTTCACCAAGAGTTTAACCAGGATACTTTACAACAACGTCTCCAAGGGCTGATTGATACAGCCCGTGAGTCATGGACTTACGCTATTTTCTGGCAGTCATCTGTCCTCGAATACTCAGGTCCTCCCATACTAGGGTGGGGTGACGGGTACTACAAAGGCGAACCAAATAAACCGAAAACAGTCATGTCAGCTACTTCCTTAGCTGAGCAACAGTATCGTAAAAAAGTGCTCCGTGAGCTTAACTCCATGATATCTGGAACACAAGCGCCAGAAAACGATGCAGTGGATGAGGAAGTCACTGATACTGAGTGGTTCTTTCTGATTTCTATGACGCAGTCTTTTGTTAACGGAGTTGGACTACCTGGACAAGCCATGGTAACGAACCAGCCCGTTTGGGTCGCGGGGCGTGAGCGTTTGATGACGTCACACTGCGAAAGAGCACGTCAGGGACAAGGGTTCGGGTTACAGACTATTGTTTGTATTCCTTCAGCTGATGGTGTGATAGAGTTGGGTTCGACTGAGTTGATGTATCAAAGTTCGGATATTATGAAGAAAGTTAGGGGTTCGTTTAATTTCAATCAGGCGCGTGATCCTATGCAAGATACAGTTGGAGTTATTGGATCTGAGATGATGACAAGTGTCATTCCGTCTGTTAATAAGTCACATGTCCCGAAACAATTACCGTTTGAAAACCCTAACACTTTATCTCAAAACCCGAGATCTGGACATAATTTTGGAAGCCGAGAGCTGAATTTTTCGGAATTCAGATCGATGGAGGGCGGATCCGGTGGCAAAAATGCAAATTCTTCGTATACGAAGCCCGAACCAGGAAAATTACTGAATTTCGGGGAGAGTAAACGGAGTATTACAAACAACGGGGCATTGTCTCCTACTTCTTGTGGTAGCAATGAAGATGGGATGCTTTCTTTTGTGTCCGGTACCGCGAAATCTGGTGCTGGCCCGTTTACAGGAGCTGACTCGGACCACTCGGACCTTGATGCGTCAATGATTAAAGAAGTAGAAAGCAGTAGAGTGGTGGAACCCGAGAAAAAACCAAGAAAACGTGGCCGAAAGCCGGCTAATGGAAGAGAAGAGCCATTGAATCATGTTGAAGCAGAGAGACAAAGAAGAGAGAAGCTAAATCAAAGGTTTTACGCTTTACGTGCTGTAGTCCCAAATGTTTCGAAAATGGACAAGGCATCACTTCTTGGAGATGCTATTTTGTACATCAAGGAGCTCAAGTCAAAGGTCGACAACACCCAATGTGATAAAGAGGAATTAAGAAACCAACTCGAGGCATTGAAGAAAGAATTATTAAGCAAAGATTCGCGACAATCTTCTTCCTCTGCTATCTCATTGCCAGACGAAATGAAAATGTCGACCCATTCACTTATTGCGGATTTGGATGTTGATGTTAAGGTTATCGGTTGGGACGCAATGATTAGGATTCAATGTAACAAGAAGAACCACCCTGCAGCTAGGCTAATGGCGGTGTTTAAGGAACTTGACTTTGAAGTTAATCATGCAAGTGTCTCAATTGTCAATGATTTGATGATCCAACAAGCGACTGTCAAGATGGGTAGTCGATTGTACTCTCAAGATCAGCTCCGAGTAGCCTTAACAAACGGATTTTCAGATCCATTATAA

AaMyc-bHLH4

ATGAAAAGCGAAGCTAGTATGGTTATTGGAGAAGAAAATTTATCAGAAGAAGATAAGTCCATTATTTCAAGTGTACTAGGATCAAAAGCATTTAATTATTTACTATCAGCATCTTTTGCTAATGAATGTTCATTTACTTCATTAGCTAATGATGAAAACTTGCAACATAAGTTATCACACCTAGTGGACAATCCAAACTCTGCTAGGTTTAGCTGGAACTATGCTATTTTCTGGCAAGTTTCTCGTTCGAAAAGTGGTGAGCTAGTGCTAGGATGGGGTGATGGGTCGTGTAGGGAGCCTAAAGAAGGTGAAGAATTCGATATAGGTCGAATTCTAAGGTTTAGGTTAGAAAATGATGAGAAGCAACAAATGATGAAAAAAAATGTTTTACAAAAAGTGCATGGGTTGTTCGGTGGTTTAGATGAAGATAATTATGCTTATGGGTTGGATAAAGTTACTGATACTGAAATGTTTTTTTTGTTGTCTATGTATTTTTTGTTTCGAAATGGTGAAGGTGGGCCCGGGAGATGTTTTGGGCAAGGGCGAGACGTGTGGATCTCGGATGCATTGAGTTCTGGTTCGGATTATTGTTTTAGGTCGAATCTTGCTAAAGCTGCTGGCATTCAGACAGTTGTTATGGTTCCTACTGACATTGGGGTTCTCGAGGTTGGATCAGTTAGATCCATACCTGAGAACCCCGGTGTTCTGCAGTCTATAAGAGCTTCTTTTTCGGGTAATTGTGTAGCCAATGGTTATGGGCATCCTGATCGTGTTATAAGTACGATCAAGATGTCCAAGATATTTGGTCAGGATTTTAGTTCGGGTATGAACCAACCACAGTTTCGCGAAAAAATCGCGGTTCGGAAACCGGAGGAGCCTGCAAGGGTGCCTTTTTCGAATTGGGCGAAAATACCAGGCCCACAGCTGCAAATAGATTTTACGGGGATCACCGAGTCGAAGGTTTCTGACGAGTGCTTGGTAAGAGATGATAGAGAAATGGTAACCACGATAATCGACGAAAAACGGCCTAGGAAACGTGGGCGTAAACCGGCTAATGGGCGTGAAGAGCCATTAAATCACGTCGAAGCCGAGAGACAAAGGCGCGAGAAGCTAAACCAACGATTCTACGCGTTACGAGCTGTAGTACCCAATATTTCGAAAATGGATAAAGCATCGTTGCTAGGTGATGCGATAACGTACATTACTGATCTTCAAAAAAAGCTTAAGGAAATGGAATCAGAAAGACAATCACCGAATTACAAAGTGTCTTTAGAGAAAATCGAAGTTCAAGCAACTGAGAACGAAGTTCTAGTACGCGTTACTAGTCCTTTAGAAGCACACCCTGTGTCGAAAGTAATTCAAACATTTGAAGAATCAAAGATAAAAGTAGTGGAATCGAAAATGAATGCGATAAATGATTTGGTGTTTCATACTTTTGTTGTCAAGTCTCAAGGACCCGAACAATTGACAAAAGAGTCGTTAATTTCTGCGTTTTCACGAGAAACCAGCTCGTCGTGA

AaMyc-bHLH5

ATGGGAGACAAATTTCGGTTAGAGGAAGAAGTTAAGGGTATGTTAGATAGTGTTTTGGGACGTGAAGCCCGCGAGTTCTTTGTTTGGTCAGCTTCAAATAAGGCTCCTGACGAGTTCAGATCAAAAACTAGTGATTTAGGTGTTCAAGAGGGTCTTCATAAGATCCTTGAAGGGTCAGATTGGAATTACGTCGTTTTTTGGCAAGTTTCAAATTCCAAATCAGGAAAATCCGCTTTGATTTGGGGTGATGGGCATCGTAAAGAATCAAAGGAACATGAAGAATATAATAATAATCGAGACGAAACTGTTAAAAGAATGAGGGTACTTCAGACGCTTCATTCGTGTTTTAACGGGTCCCACGAAGGAAATATTGCATCGAAAATGGATTCGGTTTCTGATTTGGATATGTTGTATTTAACATCTATGTACTATCTATTCCCGTTTGATAAACCTTCTAGCCCATCTCAGTCGTTCAATACAAGTCGGTCTGTCTGGGCTTCCGATGCCAAAAGTTGTGAAGAACATTACCACTCAAGATCGTTTTTAGCTAAGTTAGCTCGGGTTCAAACACTCGTTCTTGTACCTGTAAAACGAGGGGTCCTGGAGATCGGTTCTTTTAAGTCAATCCCAGAAGATCAAACCTTTGTTTCAACGGTCAAAACTTTATTTAACGGATACCATCCCCCGAAGGTATTACCGAAAATATTTGGTCAAGAACTTAGTCTAGGTGCAAAATCAGCTCCGAAATCAGGTCCAATAAGTATAAGTTTTTCCCCAAAAGTCGAAGACGATATGGAGTTTGGTAGCGAGTCCTATGAAATACAACCAAGTTTTGGTGGGAATTCTTCGAACGGTAATCAATCGATGGTATTGAATTCACAAGCGTTAGTATCTGGTCTCGATCAGTCGAATCAAGATTTATTAACAGATAGGAAACCGAGAAAACGGGGTCGAAAACCTGCAAACGGTCGCGAAGAACCGTTAAATCACGTGGAAGCCGAAAGACAAAGGCGTGAAAAGTTAAACCAAAGATTCTACGCGTTACGAGCAGTGGTCCCAAACATATCAAAAATGGATAAAGCATCACTTCTAGGTGACGCAATATCATACATAACCGATCTTCAATCAAAGATAAGAATATTAGAAGCTGAAAAAGAGGTAACGGGTGCACCCGAGGTTGACTTTATGGCTAGAAAAGATGATGCGGTTCTAAGAGTTAGTTGCCCGTTAGATGAGCATCCAGTTGCACGGGTTATAAAGACGTTTAGGGAACACCAGATCGTAACCCAGGACACAAACGTTACTGTTGAAGATGGGAAGGTGATCCATACGTTTTCGTTTCAGGGTCTTGCTGGCGGTGCGGCTGAAGAGCTCAAAGAGAAGCTGGATACAGTTTTATTGGATTGA

AaMyc-bHLH6

ATGAAATGTACTATATTTTTAACATCTCTATTTATTCTCCATCACTTATTGCTATATATCATCAGCATCAGATGTAACAAGATTCAAACCATTCGTTACGAGCTAATTGAAATGGATGATTTAATAGTCTCTTCATCTTCATCTTCTTCCATTGTTTCGATTCCTAGTACAACCATTAATCCAAATCAATGTGATACCCTCCAGCAAAAGCTCCAAACCCTACTACAAAACCAGCCCCAACCATGGGCTTACGCGATTTTTTGGCAAACCTTCAATGATGAATCAAATGGAAGCGTCTCCTTGTCGTGGGGAGATGGTCATTTTCTAAGCAGCAACGACATATTACCTGACAGTTTTCTCCCTGATTCTGACCTGGATTGTAGAAAGTCTGTTGTTAGAGAGATTAAGGCCCTTCTTGGACCTGATAACCGGGAAGATGTTGAATGGTTTTATGTGATCTCGTTAACTAGGTCTTTTATGCCTGGAGATGGTTCGGTTCCTGGTACCGCTTTGGGTTCGAATTCTATGATATGGTTAAGTGGGGTGGATCAACTTCAGTCTTTTAGCTGTGAGAGAGCAAAAGAGGCTCAGGTCCATGGATTGGAGACAATGGTTTGTATTCCAACTTGTAATGGTGTTGTGGAAATGGGTTCGTATCATTTCATCGAGGAGACATGGAATTTGGCGTATCAGGCTCAGTCATTGTTCGGCTTTGGTGGCGGTTCTACGAAGTTTAACGAGCTAAATGATGGCCATCATAATATTATTTCTTTTGCGGATATGGTACTTATGACTAGCGGATTGCATGACGAACAGGACGAGGGTATTAAAGTTTTGGGTTTTGAAGCAAACACGCCTGATGATGAGATGTCCAAGAATGGTGGAAAGTTACGCACGAACATGAACAATGTAGTCATTACCAATACCTACATAGAGACTGGGTCGGAACACTCAGATTCTGACTGCCAGTTGGTTCTTGCAACCTCAGAAAAGAGGTCGCAAAGAAAAAAAGGGAAAAACACTCGTGGACGGTGTCCACCTGTGAACCATGTGGAGGCAGAGCGACAGAGGCGTGAGAAGCTCAACCAACGCTTTTACGCCCTTCGCTCAGTCGTCCCAAATGTGTCTAGGATGGACAAAGCATCCCTCCTAGCTGACGCTGTCTGCTATATCAGTGAACTGAAACGAAAAGTTGAGTGTCTTGAATCTCAATTGCAGCATCGCAACAACCAAGGGAAAATCAAGAAAGTGAAGACAGAATTGCCGCACACCACGGACAACACTTGCAACTTGTATGTATCGACTAAACCAATACTCAAGAATAACAATAAGGCAAATTTAACCATGAACAAAATGACAAGCGGGTTCGGAGAAATAGAAGTGAAAATCGTTGGTGAGGACGCAATGATAAGGGTGCAATCAGGGAATGCTGACTTGCCCACGGCCAAATTAATGGATGCTCTTCGAGAAATGAAGGGACAAATCAAGCATGCAAGCATGTCGTGTGTGAACGACATAATGCTTCAAGATGTAGTGGTTCGAATCCCCGGTGCAACCGACTCGGATGAGCTAAAATCCGATCTCGTTAGGATTTTAGACCTCTAG

AaMyc-bHLH7

ATGGTTAGGGTTTTGGTATGGGGAGATGGATACTACAATGGATCTATAAAGACGAGGAAGACTGTTCAGTCGATTGAAGTTAGCACTGAGGAGGCTGCTTTAGGTAGAAGCGAGCAACTTAGAGAGCTCTATGATTCGTTGGTTGCTGGTGATCACCTGGTGACAGAGAACCCGCAAGCAACAACTATAAGGCGACCATCAATGGCACTGTCACCAGAAGATCTTACTGAATCCGAGTGGTTTTACCTCATGTGTGTCTCTTTTTCTTTTCCTCCTGGTGTCGGATTGGTTGGAGAGGCATACACGAAGCAGCAACATCTATGGCTCACAGGGGCAAATCAAGTCGATAGCAAAGTTTTTACAAGAGCTATTCTTGCTAAGAGTGCTAATATACAGACAGTGTTGTGCATCCCTCTACTAAATGGAGTCATAGAACTTGGAACAACAGACAAGGTGGAAGAGGCTATTGAATTAGTTCAACATGATAAAACTGGAATGGATGCAGTTATGGAGGCGAACGAATTATTGCCACTTGATATGTCAGAAGATATTAGATTTGGATCACTGAATGATGATCCCAATCACTTGGACTCTCATTGTAACTTGTTAGCAATGATCCACGACGATTCTTATAGAGCCGAGTCAATCCCCAAATGGTCAGATAATTTGGAGTTCAACGAGCCAACAAGCATTCAACTACAAGTATCAGGTGAATTATCACAAGGGGAGGACACACATTATTCTCACACAGTTTTAACCCTTCTCAACAACCAACAACTAACTCAACGGTCCAATTTTAGCACACCTTCCTGCCAAAACTCCATCCAATCAGTCTTCGCCACGTGGACACCAAATCACCTCTTTCCCGCCAAAACCACAAAAACATCCCAACGCGTCCTAAAATACATGCTATGCACCGTACCTTATCTCCACTCCACAGCCAGCCCCGGAGACACCTCCACAGTCGTAGGGGCAGCCGCTCGCCATGATGAGGTCAGCATGAACCATGTCATGGCGGAACGGCGTCGTAGAGAGAAGCTAAACGAGCGTTTCGTGACACTCCGGTCACTAGTCCCACTAGTGACAAAAATGGACAAAGCGTCAATACTAGGTGACACGATTGAGTACGTTAAACACCTGCGTAAGAAGGTTGCTGAGCTTGAGGCGCGTGGATGTCATGCGCCGCAGGGGAAGTTGCCAGAGAAGAGGAAGATTAGGGTTGTTGAAAGTGGAGTTACGGCGGTGGAGGTTTCGATAATCGAGAGTGACGCGTTGGTGGAGATTGAGTGTGTGCATAGAGAAGGGTTGTTGCTTGACGTGATGAAGAAGTTAAGGGAATTTGGTGTCGAGATCGTGACCGTTCAATCCTGTGTCGATGGTGGGATTTGTACGGCTGAGATGAGAGCTAAGGTGAAGATGAAGGGTATTAGGGGGAAGAAAATTAGCATAATGCAAGTAAAGAAAGCCATCAACCAAATAATATCTCCTTAG

AaMyc-bHLH8

ATGGGAACTAGAAGTCCGTTAAGGAGTTTCCTACAGAGTCTGTGCGAGAATTCTTGCTGGAATTATGCTGTTTTTTGGAAGCTTCAGCAACAAAACCAAATGATTTTGACATGGGAAGATGGATGCTTTGGCAACATGGAAGCTCAAGATTCCATTGAAAACATGTTTGCTGAGACTAGCTTCCAAGGCTTAGAAGAAACATCTTCATGTAACACATATCGTGGAACTTCGGGAGGAGAGGCGGTTGAACTAGCTGTGGCTTATATGTCCAACTTTCAGTATGCATTGGGTGATGGGGCTGTCGGTGATGTGGCATACACAGGGAACTCTCAGTGGGTTTCTGCAGGTTCAAAAACATCAGGGGAAATAAACACTACATCAATTTCTGAGCATGCAGATGAATGGGTATTTCAGTTCGCCGCAGGTGTGAAGACTATTCTGCTGGTGCCTGTAATTCCACATGGAGTTCTACAACTCGGCTCGTTGGACGTTGTTCCTGAAGATGCAAAAATGGCCAACTATATTAAAAGTGAATTTGTTGCGCATCAAGATCTCACAGCTTATTCTGATGCTTTTGCAACAAACCAACAATTTTCATCTCAATCACCATCATCACTCATGTCTATGAAAAGTTTGGATGAGCTCCTATATGATGATGTAGCAACCATTGATAATGTTAACTGGTCAAACCACAAACATTTGACCAATGGTATTGAAATACCAACTTGTGATAACTGGTATGCATCTCATTTAGCAGGAATAGATATGCATAACCCTTCTGCAGGCGGCCAGATGGGTGCTATTGAATTGACAGAGCCACTACATCAATTAACAACTTTAATCAATCCAGATGTCATAGAAAGCAGCAATTCCGGATGTCTAGAACCATTAGTTTGTGACGTGGACGCGTTGTTAAGCTTCCCTAAAGAGTGTGAGCTGCACAAAGCTCTTGTCCCTCCTTTCATGGGAAAAACAGATGATTATTCCCATCATCTTTCCATCGGGGACGATATGTACACCTCAAGCCACATTTTTAGTGAAGATCCTGTTGACTCATTACCTAAGAAAACAAATGGATACCTTTCATGCAAGGAGGATGTGCGAAATCCCTTAGGAAATGTAGTCACAAGTCTACATTTAGGTGAGAATTCTTCTATCCAAACCAGTGGCCAGAGCTCAATTATTAATTCGCATGGCCAATATTCCTCTATGGCAAAAAGAAAAAACATGCATGACAAAAGCGCATTTGAGGGAGAGAGTTCACTAATCAATAACCATGTAGCACCTGCGTTATGTTATAATACTGCCAAAAATTATAATGATAGTCCTTCACCTTCAGCTATTTCGTACGAGGATGTAGTTGATGAATTGGCCGTGGAAGAAGAGCGAAAAAATAGGTATGATGGTTTGCATCATAATGAGGGTTCAAAACCATCCATTGCCAGCAAAAGAAGGGGCAAACCTGGTGCTAAACAGAAGGCCAGACCTCGAGACAGGCAATTGATCCAGGACCGACTCAAAGACTTACGCGAACTTGTTCCAAACGGAGAAAAATGTAGCATTGATGGTCTTCTAGATCGAACTGTGAAACACATGCTGTTTTTTGAAAGGGTTGGTGTTCGTGCTATCAAATTGAGGCAGTGTTTGCAATCAGAGGGAATGGGAATGGGTCCAAAGAACAATAGAACAACTGAAGACAAAGGCAGTCAGAATGGAGCAAGTTGGGCATACGAATTGGGAGGTGATATTAACGTATGCCCCATACTTGTTGAGGATCTTCAATACCCTGGCCACATGATAATTGAGATGCTATGCAATGAGAGCATTTGTTTCCTTGAGATTGCGGAGGTGATACATGGCCTGGACTTGACAATTTTAAACGGTGCGATGGAGATGCGTTCCGGTGATACATGGGCTCGCTACATTGTGGAGGCTCCAAGGGGATTTCACAGATTGGATATATTTTGGCCATTAATGAAGCTTCTTCAGCAGCCCAAACAACATAGTCCAATATCCAGCAAGATTTGA

AaMyc-bHLH9

ATGATGGGTGGTGGTGCTCAATTACGCCAACTACTCAAGCATCTTTGTGTTAACACTGACTGGAAGTATGCTGTATTTTGGAAACTCGAGCATCAAGATCGAATGATGTTGACTTGCGAAGATTCTTACTGTGTTAATAATGAGAAAAACAATCGTTTAGAGAGCAACTGGTTTGATAACATGGGTGACAACTTGAAGGATGGGTTTTATGCAAAAGATCTTATTGGATTAGCAATTGCAAAGATGTCTTATCGTGTTTATTCTGTTGGGGAAGGGATTGTTGGACAGGTTGCAGATACCGGTAAACATCTGTGGATAAACGGGCATCAACTTGTAAATAGGGTGTGTTCTTTAGAAGAGCCTCTTGATGGATGGAAAACACAGTTTAGAGCCGGGATCAGGACTGTTGTTGTTGTTGACGTTGTTCCTTATGGGGTTATACAACTTGGCTCTTTAAAAACTGTCACTGAGGATTTGAAGCTGGTGAACAATATCAGAGAGATCTTTTTCGAGCTTCAAAATTCATTGATGTCATCTACGGACTGTTCTCCTTGTGTGACAGATGTGTCAACAAGGTCGGATAACCGTACGACAGTTGAAATGATCAACTATCATCAAGAAAGAGAAAACCCAATATTAGATTTCATCCAATCTACACTCTGCAATGAGCAAAATCGTGACTTGTTCATGCCGGGAACTTCAAATGATCAAATTAACTCCAACTACCATCATGATGTAGGAACTGAAACTAACGAGTCTTTCGTGTTTCCATCTGGGTGCGAGTTGTATGAAGCGTTAGGAACGGCTTTCTGTAAGCAACCATACAATTTTGATTGGGAGACAGCAACAACCGAGACCCTAAAAGTTGACAGGATCCCGGAAGAAACAAGTAGCAGTGTCTTGACACAAGTATCCGGCTCTGAGAATCTCCTGGAAGCAGTTGTAGCCAGGGTTTGTTGCAGCGATAGTGATGATAAAAGGCCAATGTCATTTGGTCAACCAGTGACTTGTTACTCAAATGAGCGATCTTTACAAGGCTTCTCATCAGCAGGCGTTAGTAAATGTAGTGAGCAGTTAGATAGGTCCCGAGAACCACCTAAGGTTGGAAAAAAACGAGCCAGACCTGGTGAAAGTCGTAAGCCTCGGCCAAGAGATAGACAACTTATCCAAGATCGTCTTAAGGAACTTAGGCAGCTTGTTCCTAATGGATCAAAGTGTAGTATCGATTCACTTTTGGAACAAACAATCAGGCATATGGTTTTTATGCAATCTGCCACGAAGCACGCTGCTAAAATGGATAGGTATGCCGAGTTCAAGTTGCTCGGCAAAGAAACAGGCATACAAGGTTCAAGCTGGGCAATGGAAGTAGGGACTGAAATAAAAGTATGTCCAATAATCGTGGAAAATATTGGCACTGATGGGCAAATGTTGGTGGAGATGATGTGTGACGAATATGTCCACTTTTTGGAGATCACTGAAGCAATCAGGAACTTGGGTTTGACGATTTTGAAGGGTGCAACAGAAGCCTGTGGTGACAAAAACTGGATGTGTTTTGTTGTTGAGGGAGATAATAACAAAAGCATACATCGGGTGGATATCTTGTGGTCCCTTATTCAGATATTGGAACTGAAGACAAAGACCTGA

AaMyc-bHLH10

ATGTTATTACTTAACTCAACCACTATGGCACAATCCTCTCTTCAAACCCGTCTCAAGTTCATCCTCCAAAACCGACCCGAAAGGTGGCTTTACACCATTTTCTGGCAAGCTTCCAAAAAAACAGACGACCATCTTGTTCTAGAATGGGCTGATGGTTATTTCCCTGAAACCAACTATGTGTTTGGACTAGATGATGTTTCTGATACCCAATGGTTGATTATGTCATCTCTAGGGATGTGTTTTCCGGCTGGACATGATGTTGTCGGCAAGTGTTTTGGTTCAAGATCTTGCGTATGGTTGGCAGGTGATATAGAATTAGGGAAATATGATAGTAAAAGATGTGAAGAAGTGAGAGTTCATGGGATTAAGTCTTTGGTTTGTATACCAACTAATAACGGTGTCGTTGAATTGGGTTGTTGTGATGTTGTGCAACAAGATTGTGGTTTGATTGAATTGACGAAATCAGTGTTTGATCCGAATAGCTTACCTAATATCAACTTTTATAATCTTGTGGATGAATCTGAATTCCCTAATCAAGGCCTTCAAAATCAAGTAAGTGAAGGTCAGGAAGAAGTGATGAGCACAAAGAAGATGAGGATGTCCTCTTCTGATTCAGATCCACTGGAAATCAATAGCTCATCATCAGTAACAACTAAGAACACATGTACACCGAAGAGGAAGGGTCGACGAGTAAAGGGCACAATCGCTCAGCCAGAAGTATTGGCACCTGGATACCATGTGGAGGCAGAGCGACAACGAAGAGAGAAGCTAAACCATCGCTTTTACGCACTCAGGAGCGTTGTTCCATACGTTTCAAAGATGGACAAGGCTTCTCTACTAGCAGATGCAGTTACTTACATCAATGAGCTCAAATCCAAAATCCAAACATTAGAAAATAAAAAGGGGTCGGAATCTTCTCTAATAAGACCTAGAAATGAAAACCAACTGAACGTCAATCAATGTAACCATGATCATGATAAAAGAACTATTGGTCATTCAACGGTTAATCAGCTTGAAGTCGAGGTGAAGTTACTTGAATCGGAAGCAATGATTAGGGTACAGTCTGCGGAAGTTAATCATCCGGCATGTAAGTTGATGGATGCACTTAGAAGCCTTAATCTAAAGGTACATTATGCAAGTGTCTCTTGTGTGAAGGATATGATGTTGCAAGATGTTATAGTGAACCTTCCTAACGGATTCGCAAGTGAGGAAGACACCTTACGACTTGCGATTCTCAACAAAATGTGCTTGGATTAA

AaMyc-bHLH11

ATGGAGGGTAGTGCATTGCATCAAGCACTCAAGACTCTTTGTCTTAACACCGATTGGAATTATGCTGTTTTTTGGAAACCGGATCCTCATGCTCGAATGATGTTGACTTGCGAAGATGCTTATTATGACCAAAATGATCCTTCAGGGAGCAAACCATTCAATAAGGGGATGGACAATTTACCTGATCAGCAGTACGCACAAGACATTCTTGACCTTGCTGTTGCAAAGATGTGTTCTCGTAGATATTCTCTCGGGGAAGGAATCGTTGGTCAGGTTGCAATCACAGATAAACACATGTGGATTTTCGGAGATCAATATGTTAATAATCCTGACTTGTCCTTTGAGGATTACGATGAATTGAAAATCCAGTTTGCCACTGGGATCACAACGATTGCTGTTGTTGGTGTTGTTCCTCATGGAGTTGTACAACTTGGTTCGTCAAATCATATACCCGAGGATCTTAAGATGGTGAATCACATCCGAGATATTATTCTTGATCTGCAAAATTCCTTAATGGGATGTACACCTAGCAATACTAGTTCATATAACGATACCATTACATCGCTAAATTATGGTGCCAACACAATATCAGATCTTGATCGAAATGTTGATTTATGTATGCCTGGTTCTTCGAATGTCCAAAGTGAAACCGACTCCATGAATACCTTAAAAACGTCTTTTAAGTTTCCTGCTGGATACGAGTTGTATGAAGCACTGGGACCTGCATTTTATACCCAAAACGATAACCATAGTTATCAGAAATTAGAGGAGATGCCGGGAACAAGTAGTAGCAATCTTCTGACACCAAATCCAGGATCAGAACATCTTCTAGAAGCAGTTGTAGCGAATGTTAGCCAAAGTGACAGTGAATTTAGTAACTCAGTGAAATGGGTTGAGACTCATTTTAATGATATGCAGACTAGTACTTCAGGTTGTTACTCGTTTGAAAGTTCATTAGGGTTCTCGTCAGCTAGTCATAGTAGATGTAGTGACCAACGAGTGATGTCACAAGAATCACCTGCTAATGTGACCAAGAAACGGGCTAAACCTGGTGAAAGTAGCAGGCCTCGGCCCCGTGATCGACAACTTATTCAAGATCGAATTAAGGAACTCCGTGAGCTAGTGCCTAATGGATCGAAGTGTAGCATTGATTCACTTCTTGAACGAACAATCAAGCACATGCTCTTCATGCAGTCTGTTACTAAGCATGCTGATAAAATTGATAAATATGCAGAATCAAAGTTGCTTAGCAAGGAAACAGGCATAAGAGGAACCTCAAGTCACGAGCAGGGTTCAAGTTGGGCAATGGAGGTCGGAAATGACATGAAAGTTTGTCCTGTGACTGTAGAAAACATTGGTACAAATGGCCAAATGCTGGTAGAGATGATGTGTGAGGAAGGTGTCCATTTTCTTGAGATAGCGGATGCCATTCGGAGCTTGGGACTCACCATTCTGAAAGGGGTTGCAGAACCTGACGGGGATAAAATGTGGATGTGTTTTGTTGTAGAGGGACAAAATAACAGAAATGTTCATAGGATGGATATACTTTGGTTACTTGTTCAGATATTGCAATCGAAGGCAAAGTCTTAA

AaMyc-bHLH12

ATGGCAGAATCCTCTCTTCAAACCCGTCTCAAGTTCATCCTCCAAAACCGACCAGAAAGGTGGCTTTACGCCATTTTCTGGCAAGCTTCCAAAAAAACAGACGACCATCTTGTTCTAGAATGGGCTGATGGTTATTTCCCTGAAACCAATTATGTGTTTGGACTAGATGATGTTTCTGATACCCAATGGTTGATTATTTCATCCCTAGGGATGTGTTTTACGGCTGGACATGATGTTGTGGGCCAATGTTTTGGTTCAAGATCTTGCATATGGTTGGCGGGTGATATAGAGTTAGGGAAATATGATAGTAAAAGATGTGAAGAAGTGAGAGTCCATGGGATTAAGTCTTTGGGTTGTATACCAACTAATAACGGTGTCGTTGAATTGGGTTGTTGTGATGTTGTGCAACAAGATTGTGGTTTGATTGAATTAACGAAATCAGTGTTTGATCCGAATAGCTCATCTAATATCAACTTTCATAATCTTGTTGATGAATGTGAATTTCCTAATCAAGGTGAAGGTCAGGAAGAAGTGATGAGCAGAAAGAAGATGAAGATGTCCTCTTCTGATTCAGATCAACTTGAAATCAATAGCTCACCATCATTAAGAACCAAGAACACATGTACACCAAAGAGGAAGGGTCGACGAGTAAAGGGCACAATCGCACAACCAGAAGTATTGGCACATGGGTACCATGTGGATGCCGAGCGACAACGAAGAGAGAAGCTAAATCGTCGCTTTTACGCACTTAGGAGCGTTGTTCCATACGTTTCAAAGATGGACAAGGCTTCTCTACTAGCAGACGCAGTTACTTACATCAATGAGCTCAAATCCAAAATCCAAACATTAGAAAATAATAAAGGGTCGGAATCTTCACTGATAAGATCTAGAAATGACAACCAACTGATCATCAATCAATGTAACCATGTTCATGATCAAAGAACTACTGGTCATTCAACGGCTAGTCAGGTTGAAGTCGAGGTGAAGTTACTTGACTCGGAAGCAATGATTAGGGTACAATCTGCGGAAGTTGATCATCCGGCATGTAAGTTGATGGATGCACTTAGAAGCCTTAATCTAAAGGATTTGATGTTGCAAGATGTTCTTGTGAAAGTTCCTAACGGATTCACAATTGAGGAAGACACCTTACGACTTGCGATTCTTAACAAAATAGCTTGTGAAGAAGTTCCTGGTGATTTGTATCCGAGTTTGGGTTACAATACGTGGGAGAAGAGCGTTCCTAACCTGTGA

AaMyc-bHLH13

ATGGGTTATTTGTTAAAAGAAGCACTTAAAACCCTTTGTGGTGTTAATAATCAATGGTCTTATGCTATTTTCTGGAAGTTTGGCTGTCAAAACCCTAAGCTTCTAATATGGGAAGAATGTTATTACGAACCTGTTATTTATTCTACTGGTGAGCATGGAATTCAAGCTACAAACAATATTCACTTGCTTGTAACCAAAATGATGAAAGACAACTACGTCAATCTTTTAGGGGAAGGATTAGTTGGGAGGGTTGCATTCACTAGAAATCTTCAGTGGATTACCTCGGATAACAAAATGCTAGATGTCCATCCACCTGAGGTATCAAATGAGCTTTGTACACAATTCTCAGCTGGCATTCAGACGATAGCAGTTATTCCGGTATATCCACATGGGGTTGTTCAACTTGGTTCATCCTCAATTATTATGGAGAATATGGGATTTGTGAATGATGTGAAGAGTTTGATTTCTCAGCTTGGATGTGTTCCAGGTGCCTTATTATCAGAGAACTTCATGAAAAGCGAAGGCACCTCGGACAGTAGTCCTAGTCAACGTGAATCCATCTGCCAAATGAATTCTGTATCTGCTCATTCATCAGGAAAATCTAAAGTGACAGACTATAACCAACAGGGCCACTCACCTCAGGCTTCTGGAAGTAGTGGACATACTTCTTTCTCTCTTAATAGGCATATTGGAGCTAACGCTGTGGCCAGCGGTTCAACACGTGTAATATCTGATGAAGATCAATGTCAAGCAAAAGTCATTCATATTATGAAGCAAAATCCCTCATTCAAAACCAATCAACAAATTGGAATCACCGGAGTAAAATCAATCACTGGGGCTAGTTTGTACAATCAGAATTCCAAAAAAGAGCAACAAAATGCTTTTGGCTCATGCTTATTAAAGTATGATCCTTTAACATTGATGGAACAGGATAACTTATCAGATGCATTTTTGGGAGATCATGCGAATGACACCATGATGTCCCAGATTGCTCAAACTTCTATTCCAGATGCTCACTTCTCTTGCACCCATGTTGATAAGGAAAATCTAGATAATGAACTATTTCAAGCTCTTTCAGTGTCACCAGAAGAAGCATGTTTAAAACCTTCCACTAGGGATGACTTGTTTGATATCGTGGGTATGGATTTTAAAAGCATATTATTTGGTGGAACTAGGACTAACTTTGTTAATAATGGAGTAGAAGCAAGCCCGCTAAATTTAAAGAGGAATGATAGCGTGGTTTTAGAGCCATTCTCTGTGTACGAAGGAGAATCTGATAGTGGTATATATTCGTCAACACCTAGTGACCATCTTTTAGATGCAGTCGTCTCTAAGGTTTACTCATCTTCGGGACAGATTGCAGATGATAATATGTCTTGTTCATCGTTGACAAAAACCAACAGCTCATCATCATCTATGTGGGCTAATAAGGGGATAAATTTGATGCAAAAAGAGTTGACTGGTGTTCCCAAATCTTTGAAAAATGAAGGGCTAACAAATTCTTGTTCATTTAAATCTGTGATCTTTGAGGATGGGGGAAACTTTTCGGATACTAGTTCTATCATTTCATCCCAAATCAGTTCTTGGAACGAAAAATTGAGAAAGGACAGCGGTGGTGCATCCCAGTATGGTAAGAGGGCTGATGAAACTAGCAAATCAAACCGTAAGAGACTTAAACCTGGAGAAAATCCTAGACCAAGACCAAAAGATCGCCAGATGATTCAGGATCGTGTAAAAGAATTGAGAGAAATCATCCCGAATGGTGCCAAGTGTAGCATCGATACTTTACTAGAACGGACCATCAAGCACATGCTTTTCTTGCAAAGTGTTACAAAGCATGCAGACAAGTTAAAACAAACGGGGGAATCTAAGATTGCCAGCAAAGACGGTGGCTTATTGTTGAGGGACAACTTTGAAGGAGGAGCAACATGGGCATACGAAGTTGGTTCACAATCAATGGTTTGCCCCATTATTGTTGAGGATCTTAATTCGCCTCGTCAAATGCTTGTCGAGATGCTTTGTGAAGAAAGAGGTTCGTTTTTAGAGATAGCCGATGTTGTTAAAGGATTGGGATTGACAATCTTGAAGGGGGTGATGGAGTCTCGTAATGGGAAGATATGGGCACACTTTACTGTAGAGGCTAACAGGGATGTGACACGAATGGAAATATTTATCTCACTAATTAGTCTCTTCGACCCAGCTGCAAAAAATGATGCATCAGCAGCTGGATGTGATGCGAATGGGAATATTTCAGTCCACGAACAATCTTTCAACCAATTAGCGACTAATCCCGCAACTGTTGGACCACATAATATTATACAGTAA

AaMyc-bHLH14

ATGATTTACCAAAATCTAGTACGTAGTATATATTCTCCCTCCCGTATTTCTATTATCTTTGCCTCACACACCTTTTGTTTTCATAACCAAACCATACGCATTACTATGGATGATGAAATCTTAATTCTTTCACCTTCTTCTTGTTCTTCAATCATCCACCAAAACACACACAATTCACACCATAAACTACAATTCCTATTACAAACACAACCCTACCCATGGGCCTTCGCCATTTTTTGGAAAACCACGTATAACCATGACTATGGTCGACCATGGACCTTAACATGGGCCGATGGTTACTTTCTTCAAAACCCAAACAAACCCGCCTTTAAAGAAATCCAAACACTAGTTGGACCGGACAACACAGACGGCGCAGAGTGGTTCTACGTCGTCTCATTGGCCCGATCCTTCGGTATTGGGGACGAGTCTGCCCCATCAAACTCCTTCACTTCTAATAGTGTCATTTGGTTAACGGGCGCTCATAGTCTTATATCTTTTAATTGTGAAAGAGCTAAAGAAGCTTATATTCATGGGCTTGAGACACTTGTGTATATTCCTACAACCAATGGGGTTGTGGAATTGGGGTCTTATCATGTGATTAATCATACCGAATCTGATTTAGCTCATCGAGTTAAATCACTCTTCAGTGCTAGTTCTTCCTCTTCGTCATCATTTTTGTCGCCATCTAGCAATTTGATTAACCAACCCAATGACTATTTGATCTCCTTCGGAGAAATGGTCTCTAGGTTGCCACAAGATGAAGAAAGTAAGGACATATTCGATTTAGGAACAACCACGTTTGGCCAACAATCCAAAAAACTTGGAAAAGTAGTTGAAAACACAAACAATAAAGGACAAAAAAAGAGTCAAAAAAAGAAAGGACATGATCCACCTCTAAACCACGTAGAGGCGGAGCGTAAGAGACTTACGAACCTTAACCAACGCTTCTACGCGTTGCGTTCAGTGGTCCCATATGTTTCAAAGATGGACAAGACATCCTTACTGGAGGACGCCGTTTGTTACATCAATGAATTAAAAAATAAAGTTGAGGAACTTGAAGCTCAACTCCAAGCCATGAACAACCAGCCCAAGTTGAAGAAAATAAAAGTCAAAGCACCAGATGTGACCTTTGTTCGAAATTCACAAGGTGCAAATATATATAATAACGGGAATCGAACCAAAACGAACGGAACCTTGGAAGTGGAGGTGAGAATGGTGGGTGATAATGCAATGATTAGAATACAGTCTGGTAACGCGGATTGGCCCTCTGCGAAACTGATGGATGCGTTACGAGAAATGGAAGCTAAAGTGCACCAAGCGAGCATGTCCTGTATTAATGACGTAACGCTTCAAGATGTAGTCGCTAGGGTACAAGAACTTATGGAAGGAATCAAACAGTATCCATTGCACTCTTATGGTTTAATCAATTCACCTAAGATACTTGCGGACATTGTGGAGTCTTTAGTTGGTGCAGTCTATGTCGACACCAATTTATCGGTCGATGCTACATGGGAGATCGGGATCAAGTTAGAATACAAAAATTTATGGGTCGAGTCGGGGGAGATTGAGATATATAATGGTAATGAGTTAATCGGGAAAGGAAACTACAAGAAAAAGAAAACAACGATGAAGAACAAAGCAGCAGCAGATGCATACGTGAATCTTGTCAAACAATTGGGTCTCAAAGATGATGCACAATTTGATGAATTTTTACACTAA

AaMyc-bHLH15

ATGGATACAGCAACTACACACAAAGATTCAGCTGTTAAGAATGTGCTCAAGAATCTGTGCACCTCTTATGGATGGTCTTATGCTGTTTTTTGGAACTTTGACCAACCAAATTCTATATTGTTGACTATGCAAGATGCTTACTTTGAGGTGGAAATCCAAGGTCTAATTAGTGATTTGCTTCAGCAAACACCTATGCTTGGAGGAGGGTTAATTGGACAGGCTGCTTTGACTAAGAAGCACATTTGGATGTCCTCTGAAGATAATTACATAGGACAGAGTTCCTCGGGTTCGATCTGGGATATGTTTCAGGATGATTCTAAATTTTTTCATCAATTTTCATCAGGTGTAAAGACAATTGCAGCAATCCCTGTGGAACCACAAGGAGTGGTACAATTTGGGTCCATTGAGAAGATTCTCGAGAAGACAGAATTTATACATCAAACAAAAAGAATGTTTGGTGAAATTGTAAATGGAAATGTACCAGCAATTTCATCTTGTACGTCAAACGGATTGTTTGCGTCCTTGATATCATCTGATGATTCCTATTTTGGAGGCGCTGCTCAATATTCAGTTAATCAAACTTCTTTAGATTTGCCACCCCAGTCTTTCGCTTTTGGTTCACAATCACAACCCATGTTTCTTGATAATATTCAGTCTACAGCACATTTACAACCGAACACTGTTCCATGGAGTACTTCTTCTAGTTTGACTTCATTCAACGAACCATTACAGTCGACAAACACTCATCAAGATTTGTTTGACTTACCAATGGATTTTGGAATCTTAGATGAAATGTTTCAAACAGGAGATTTCAACATTTCTCAGTTGTTACCTCAGTCCCCTGGTCAAAGTAATGTCACAGGATTACTTCCCATAAGTTCCAATGAAAAATTCAACTCCTTGACTATTTCTGGTGTAGATGTTGACCTTATCGGAAGTACCGGAGACTTAGGAGATATTGTAACACCAGTCGTAAAGGAAAATCAATTCGGTTTTGATTCGTACAAGTCTCAAAATATTGATAATGACTCTGCTCCGAAAAAAGGCCTGTTCTCGAATCTTGGGATCAAGGAGCTTTTTGAGGGTATTTCTGGTACTTCAAGCTCTTGTATTGAGGATCAAGTGTCGAAAAGAAGGAAAACAGGTAGTTCCTTTTCCGAAATGAGTTCCTTACAGCCTGGTGTATATAACAAAAGTTTTGGGCAGAAGAGCGAGCCCATTCTTAAATCAGAGCCATGGATGGGAAGTGCTTATAGCATGGATGGTTCAAGCACTGTTTTGCAAGCCAAGACGCAAGTCGAGCCTGCAAAGCCCACCAAGAAAAAAGCTAAACCAGGTACTCGACCCCGGCCCAAAGACCGGCAGATGATATTGGATCGTATGGCAGAGTTGAGAGAACTCATCCCCAATGGTGAAAAGATGAGCATTGATTGTTTGTTGGATCGGACAATCAAGCACATGCTTTTCTTGCAAAGTGTAACAAAACATGCTGACAGAATTAAACAGGCTGATGAACCAAAGCATAATGGTGTCATTCCGAATAATTTATCCAATGATCCAAACAACAATGGTGTGACATGGGCATGTGAAGTGGGAAATCAGTCGATGATTTGTCCATTAATAGTGGAAGACCTTAGTACCCCTGGACAAATGCTCATAGAGATGATCTGTGAAGAACACGGGTTCTTTCTTGAGATAGTAGATATAATCCGTGGTTTTGGCTTGACCATATTAAAGGGAATCATGGAATCTCGTGAGGAAAAAATATGGGCGCGCTTTATAGTTGAAGCTGAGGCAAAAAGGCATGTAACGAGGCATGAGATATTTGCAGCACTTGTTCAACTTCTACAAACAATGGGGTCTAACGATAAACATCTTGAAAAAAAGATTATGCAAACGGGGAACTCTTTTCATAACGGTTTCCAGCATTCTGGGATACAGCTTCCTGTAAGCTTGGCGGACACAGTGTATGGCATGAACTAA

AaMyc-bHLH16

ATGGAGGTGTTGCAAGAAATGATGGATAGGCTAAGATCGATTGTCGGTCCAGAGAGTTGGGATTACTGTGTGCTTTGGAAACCATGTAAAGAGCAAAGGGTGATTGAATGGATAGATTGCTGTTGTTCTGGGAGCAATGCTGCAAGGCATGGGATTGGAAATGATGATCAACAACAACAGTTGGTGTTCCAATGCAAAGATGTCGCATTTCATCACCCAACTACTGATACTTGCAACCTCCTTTCACTTTTACCTTCGTCGATGCCTTTTGACTCTGGACTCTATGGACAAACAATGATATCTAATCAACCAAGGTGGCTAAACTTTTCTAATAGTTCCAATTCTGGGTATTCTGAGGAAAATCTTGGGACTAAAGTCCTTATTCCTGTTCCAATTGGACTCGTGGAACTCTTCGTATCTAAACAAATATCTGAAGATCAAAGCATCATCGATTTTGTGACCACTATGTTTAACATGTCACTGGAACAACCTATGTTGAACCCAAACAATAATATCGACTCGAGTTTCTCTGTTAACATGGATAGTCTGGACGATGGGGAGTCAAAAGATTACATAGCTCAAGTTCTCGATGATCAAAAGGATCAAAATAACCATTTCCAACCACCGATTTCTCCTGCTACCATGCTAGAAAACCTTAACCTCTCACCAAACAACATCTCTGACAACCATATGCATCCTATGAACTTCTTGCAGCAATTTAACTACGGGGAAAGCAGAAATACAAATAACATCTTCATGGAAGGCACAAGCGAGCCTATGATGAACCATGATGGCCCTTTTGACCCTAATTCTGAAGATAATGTTGGATTTGACCATGAGATTGATATGGCGTTGCAAGGGCAAATGATGAGCGAAAATATGGGCAAGGCTCATCTCATGGAGCCGTTAGAGAACACTCCAAAGAAGCAGGGAAATGACATGAACCGGTCAGATTCTGTATCTGACTGTAGTGATCAAAATGATGAGGATGATGACCCTAAATGTCGAAGGAAGAATGGCAAGGGACAGTCCAAAAACCTCATGGCGGAGCGCAAAAGACGAAAGAAGCTTAATGATCGGCTCTATACTCTGCGCTCTCTAGTACCCAAAATCACTAAGTTGGATAGAGCCTCAATTCTCAAGGATGCCATCGAGTATGTAATGGAGTTGAAAAGGCAAGTGGAAGATCTCCAAAATGAGTTGGAAGAAAATTCGGATGATGAGGGCACTACAAACAACCAAAGCACCATCGTTGAACAAGAAGTTATCCACGGCAATGGAAGTAACTCAAAGCGTAGATACAACCACGGCCCTGGCCTGTTCGTGAATGGACCTCAACTGGAAGCGTATTCGGGTGTTGGTAACATTGAAGTCTCAAAACAAAACCAAGATGTCGAAGGTGCTAGCGAGAAAGGCCAACAAATGGAGCCGCAAGTGGAAGTGGCATCGTTAGATGGAAATGAATTCTTCGTGAAGGTATTCAGTGAGCACAAACCAGGAGGATTTGTGAGATTGATGGAGGCCTTTAATGCTCTAGGACTTGAAGTAACAAATGTCAATGTTACTAGTTTCAGATGTTTAGTTTTGAATGTCTTCAAAGTTGAGAGGAAGGATAGTGAAATGGTGCAGGCTGATCACGTAAGGGAGTCGCTGCTCGAGATAACACGAAACCCATATAAGGGTTGGCCGGAAAACTCCATGAAAGCACAAGAAAATGGTCATGGCATGGCAAAAGTACTTTTGGAATCATTCAATGGTCTTCGGAATAAGCCCAACCCTGGCAACTCATCTTCTCCACCCTATGATCTTTGTTTCTTTTCACATCCATCAGTAATTCTTCAACAATCGTTGCTCCACCACCAAGATATTCAGATGACGAGATCACCATTTGCCCAACAAAAAAAACGAAGGATACAAGCTGCCATGATAGCTGCCACAGGAAATTTCAACATAATAACAACTATGGTTCGTCGGTTTATGTTGGGTTGTTTGATCATCAATCATTTAATCGAGTCTAAAACGCTTCGTAGAAAATATATGGTGAGTTTGAGTAGTAGACGTGACAATATGTGGAGCATGGTGTATCAAAGTGATATTGCGAGTGTGGTTAATATTCGAATGAATATACTTGCGTTTTCGAAACTTTGTAAATTGCTTGAAACAAGAGGAGGACTGCATAGCAGCAAACACATGCTAGTTGATGAGCAGTTAGTTTTAGATGCCATATGCCGACTTCACAAAGAATTCTACAAGACACCTGTGCGAGTGCCCGACAATGAAACTGATGAGAGGTGGAAATGGTTTAAGGGTTGTCTCGGAGCATTGGATGGTACTTATATTAAAGTCAGGGTTCCAACATGTGATCGAAAACCTTATCGGACACGAAAGGGTGAGATTTGTACCAATGTTCTTGGGGTGTGCACAAGAGACCTTATGTTTTCTTATGTACTTGCGGGATGGGAAGGGTCGGCAGCAGACAGTCGTGTGCTTCGAGATGCTATTAGTAGGCCAAATGGTCTTAAGATAACCCGTGTAACATACATGTATCATGAGGAAACAATGTCACCTTTTCGGGTTGTGCTTTCAAGCACAGCCTAA

AaMyc-bHLH17

ATGGGAAAGTCCCTTCTTGGACCGGATAACTCAGACGACGCAGAGTGGTTCTACGTCGTCTCATTGACCCGGTCCTTTAGTGCTGGGGACGGTTCTGCCCCAGCAAACTCATTTGCTTCTAATAGTGTCATTTGGTTAACGGGTGCTCATAGTCTTTTGTCTTTTGATTGTGTACGAGCTAAAGAAGCTCATATTCATGGGCTAGAGACACTTGTTTATATTCCCACGACTAATGGAGTTGTGGAAATGGGGTCTTTTCATGTGATTAATCATACCGAATCTGATTTAGCTCATCGAGCTAAATCACTATTCGAAATGGGTGCTAGGTTGCCACAAGAAGAAGAAAGTATGAACATTATCGATTTTGGAACAATCACGTTCGACCAACAGCCCAAGAAACTTGGAAAGATAGTCAAGAACATGAACATGAAAGGCAATGAAGTCTCGGAAACAGGATCCGAGGAGTCGGATTCTGATTGCCAGCTAGTTGTGGCAACCAGTAAAAAAGTGGGTCAAAAAAAGAAAGGACGCGATCCGCCTATAAACCATGTAGAGGCGGAGCGTCAGAGGCGTGAGAAGCTTAACCAACGCTTCTACACGCTACGTTCCGTGGTCCCAAATGTTTCAAAGATGGACAAGGCGTCCTTACTAGCGGACGCCGTTTGTTACATCAATGAATTAAAAGGTAAAGTAGAGGAACTCGAAGCTCAACTCCAAGCCACGAATAACCAACCCAAGTTAAAGAAAATAAAAATAGAAATACCGGATGTCACCATTGTACCGAAATCAAATGGGGCAAAAATATATAACAAAAAGACTCAAACCAAAATGATCGAAAATTTGGAAGTGGAGGTGAAAATGGTGGGTGAGGATGCGATGATTAGAGTACAATCTGGTAACGGGGATTGGCCCGCTGCGAAACTGATGGATGCATTGCGAGAAATGGAAGTTAAAGTGCACCACGCTAGCATGTCGTGTGTCAACGACATTATGCTTCAAGATGTAGTCGCTAGGATTACTGGTTCTACTGAAGATGAAGTTAAATCTCATCTTCTAGCTAGATTAAATCAGTAA

AaMyc-bHLH18

ATGGGTACATTACAACAAGATTCAATTGTTAAAAATGTTATCAAGAATCTTTGTTGCTCTTATGGATGGTCTTATGGTGCATTTTGGAGCTATGATCAACCAAATTCCATATTATTGACACTGCAAGATGCTTACTTTGAGGAAAAATGCGGATCATTGATCGATAATTTGGTTCGACAAGTACCACTTGGAGGAGGGATAATTGGACAAGCTGCATACAACAACAAACACACATGGATGTCCTCAGAAGATCATTACAATGAGCAGAATTTTTCGGGCTCGATATGGGATATGTTTCTGGATGATTATGAATTTCGTCGGCAGTTTTCTGGCGGTATGAAGACAATTGCAGTCATCCCTGTGGAGCCACGAGGAGTGGTACAATTTGGGTCCATTGATAAGATTCTTGAGACGGTGGAATTTGTTAATCAAACAAAAATTATGTTTCAAGAAATTTTAAATCTTGGAGGGTCTGAAGTTGGTTTGACTTCTTTGGATGGTCAAACATGTTATCAGAATGAAGCGTTCGCTTCCTTGATATCACCCCAAGAATCTTTCTTTACTGATTTTGGTATCCCAGATGAGTTCTTTCAAAATGGAACTTTCCCTCCACTGAATCCCATCGATTCTGATGGAAAACACGAGTCTTTGACTATTTCTGGTACTGATGTTGACCTGCTCAGAAACAGTGGTGATTTGGGACACATTTTAGCTCCGTTTATAGATGGAAGTCACTCGGGTTTTCATTCTTACAGCTCAGAATGTATGTCCATGTCAAAGTCAGCACAGCGTGTTGACTGTACTACCCGTGGTCCCAAAGAAAGATTGTTCTCAAAGCTTGGTATCGAAGAGCTTTTGGAGGGTGTTTCTGGTATTTCAAATGCTGATTCATTGTCTTGCATTGATGGTCAAATTTCAGCAAAAAGAAGAAAAACAGGAAATTCAAAGTGGGAAGAGGTTATGCCTATATCACAGCCTTGTTTACACATGGTTGATGGTTATAGTGTGAGTGATTCGAGCACAGTTATGCAAGCTAAGAAGCAAGTTGAACCATTAAAGCCCATCAAGAAAAAAGCCAAACCAGGGACTCGTCCCCGACCCAAAGATCGCCAACAAATTTTGGATCGGATGGCTGAGTTAAGGCTACTTATTCCCAATGGTGAGAAGATGGATGCTAATGTAGCTGCTGAAGTATCTGCTGATGTTCCATTGGTTAATACTTCAGTGGAAACACCTGAGTCAGCAGATGTCTCTGCTACTGAACCTTCTTCTCATCATTCGATATATATTCCTGGTAGAAGGGCTAAAAGAATGGCAAGGATGAAAGTCTCGTCTTCTTCTGTACATAGGGAAGTGGATCTAGATGCTGCTGAATCTTCATTTANAAGTATCTGCTGA

AaMyc-bHLH19

ATGGGTACATTACAACAAGATTCAATTGTTAAAAATGTTATCAAGAATCTTTGTTGCTCTTATGGATGGTCTTATGGTGCATTTTGGAGCTATGATCAACCAAATTCCATATTATTGACCCTACAAGATGCTTACTTTGAGGAAAAATGCGGATCATTGATCGATAATTTGGTTCGGCAAGTACCACTTGGAGGAGGGATAATTGGACAAGCTGCTTACAACAACAAACACACATGGATGTCCTCAGAAGATCATTACAATGAGCAGAATTTTTCGGGTTCGATATGGGATATGTTTCTGGATGATTATGAATTTCGTCGGCAGTTTTCTGGCGGTATGAAGACAATTGCAGTAATCCCTGTGGAGCCACAAGGAGTGGTACAATTTGGGTCCATTGATAAGATTCTTGAGACGGTGGAATTTGTTAATCAAACAAAAATTATGTTTCAAGAAATTTCAAATCTTGGAGGGTCTGAAGTTGGTTTGACTTCTTTGGATGGTCAAACATGTTATCAGAATGAAGCGTTCGCTTCCTTGATATCACCCCAAGAATCTTTCTTTACTGATTTTGGTATCCCAGATGAGTTCTTTCAAAATGGAACTTTCCCTCCACTGAATCCCATCGATTCTGATGGAAAACACGAGTCTTTGACTATTTCTGGTACTGATGTTGACCTGCTCAGAAACAGTGGTGATTTGGGACACATTTTAGCTCCGTTTATAGATGGAAGTCACTCGGGTTTTCATTCTTACAGCTCAGAATGTATGTCCATGTCAAAGTCAGCACAGGGTGTTGACTGTACTACCCGTGTTCCCAAAGAAAGATTGTTCTCAAAGCTTGGTATCGAAGAGCTTTTGGAGGGTGTTTCTGGTATTTCAAATGCTGATTCATTGTCTTGCATTGATGGTCAAATTTCAGCAAAAAGAAGAAAAACAGGAAATTCTAAGTGGGAAGAGGTTATGCCTATATCACAGCCTTGTTTACACATGGTTGATGGTTATAGTGTGAGTGATTCGAGCACAGTTATGCAAGCTAAGAAGCAAGTTGAACCATTAAAGCCCATCAAGAAAAAAGCCAAACCAGGGACTCGTCCCCGACCCAAAGATCGCCAACAAATTTTGGATCGGATGGCTGAGTTAAGGCTACTTATTCCCAATGGTGAGAAGATGAGTATCGACTGTTTGTTGGATCGGACCATCAAGCACATGATTTTCATGCAAAATTTAGCGAAACAAGCTGACAAAATAAAACAGGCGGAAGAACGAAAGCATAACAGGATTGACTCCAATGACACGAGCACAAATGGAGTGACATGGGCTTGCGAATTGGGAAATCAGACAATGGTTTGTCCACTAATGGTTGAGGACCTTGATGAACCTGGTCAAATGCTTATAGAGATGATTTGCGAAGAACAAGGATTCTTTCTTGAGATTGTAGACATAATTCGGCGTTTTGGGTTGATAATCTTGAAAGGGGTTATGGAAACTCGCGGTGATAAAATATGGGCACGCTTCATAGTTGAACCCGAGGTGAACAAACACATAACAAGACACGAGATATTTTCAGCACTTGTTAAGTTTCTACAAGAGAATGCGCACATTGTTGATGAAAAATGCACACGACAAGGGAATTCTCTACTTGGCGATTTTCAACAAGCTGGGATACAAAATATTGGGATACAAAATCTTGTTAACTTAGCAGATATGCAATATTTTGTAAACTTGTAA

AaMyc-bHLH20

ATGGCACAATCCTCTCATCAAACCCGTCTCAAGTTCATCCTCCAAAACCGACCCGAAAGGTGGCTTTACGCCATTTTCTGGATAGCTTCCAAAAAAACAGACGACCATCTTGTTCTAGAATGGGCTGATGGTTATTTCCCTGAAACCAACTATGTGTTTGGACTAGATGATGTTTCTGATACCCAATGGTTGATTATGTCATCCCTAGGGATGTGTTTTACGGCTGGACATGATGTTGTCGGCCAGTGTTTTGGTTCAAGATCTTGCGTGTGGTTGGCAGGTGATATAGAGTTAGGGAAATATGATAGTAAAAGATGTGAAGAAGTGAGAGTTCATGGGATTAAGTCTTTGTTTTGCCTAACAAAAGCTAGATCATTTTTAGGCCTTCAAAATCAAGTAGGTGATGGTCAGGAAGAAGTGATGAGCACAAAGAAGATGAAGATGTCCTCTTCTGATTCAGATCCACTGGAAATGAATAGCTCATCATCATTAACAACCAAGAAAACATGTACACCGAAGAGGAAGGGTCGACGAGTAAAGGGCACAATCGCACAGCCAGAAGTATTGGTACCTGGGTACCATGTGGAGGCAGAGCGACAACGAAGAGAGAAGCTAAATCATCGCTTTTACGCACTTAGAAGTGTTGTTCCATACGTTTCAAAGATGGACAAGGCTTCTCTACTAGCAGATGCAGTTACTTACATCAATGAGCTCAAATCCAAAATCCAAAGGTTAGAAAATAATAAAGAGTCTGAATCTTCTCTAATAAGACCTAGAAATGGCAACCAGCTGAACATCAATCAATGTAACCATGTTCATGATCAAAGAACTACTGGTCATTCAATGTCTAATAAGGTTGAAGTCGAGGTGAAGTTACTTGAATCGGAAGCAATAATTAGGGTACAATCTGCGGAAGTTAATCATCCGGCATGTAAGTTGATGGATGCACTTAGAAGCCTTAATCTAAAGGTTAATTATGCAAGTGTCTCTTGTGTGAAGGATTTGATGTTGCAAGATGTTATTGTGAAGGTTCCTAACGGATTCACAAGTGAGGAAGACACCTTACGACTTGCGATTCTTAACAAAATGTGCTTGGATTAA

AaMyc-bHLH21

ATGAGTGCATTAACTGAGCGGTTAAGACCTCTTGTAGAAACTAAGTCATGGGATTACTGCATTGTTTGGAAATTTGGTGATGACCCTTCTAGGTACATTGAGTGGTTTGGTTGTTGTTGTAATGGTAGTAGTAATCAAGATGTTTGTGGCAATGTCAAGAAGGAAATCGAAGAAACGAAACCGCGTTCTTCTCAAGTGTGTAGAGATACTTTTGTTGAACATGGTTTGGGTACGAAAGCTTGCGAGAAACTAGCTGATATGCCTTTTTATCTGCCACTGTACTCTGGGGTCCATGGTGAAGTTGCAATGTCTGGTCAACCATCTTGGAGTCATGATACTATTGGGACTCAAGTTTTGTTTCCGGTTAATGGTGGGTTGCTTGAGCTCTATATCTCAAAACAAGTTCCGAGAGATGAAGAGATGATAGAGACTCTTACGGCACAGTTTAATGCACTTTCTAAGGATGAATGGTTTTGTGACACGAAAACTGCTGCACAACACATGGTGTATCTGAAAACTGAAGGATCGCCAAATGGTGAAGGACTATGGAGTGAAAACTCGTCACTTGTTTCAGCTGGTTCGGCGCAAGTTTCACCCACTCAGTCCATTGATAATCCGATAAACGTGTTGGGTGACATGAAGAGTAGGCAGAAGAATGGCAAAGAACAATACCAATCTAAGAATCTTGTGACGGAAAGGAAAAGAAGAAACAGAATTAAAGAAAATCTCTATATTTTACGCTCTGTAGTCCCTAAGATTTCAAAGATGGATAAAGCTTCCATACTTGGAGACGCAATTGAGTATATAAAGGAGTTGCAAAATAACGTTCAAGAACTTCAAGATGAGCTTAAAAGATCGGAAGAAGATGAGATTAAGTCTCATGAGGAAGAAGTTGAGGTCTGCAAACCGAAAAGAAAACGAGCATATGAACACTCGCCTACAAAAGGACATAGTCTTGTTTCAACATCACCAGACAAAAAAATTGAGGTTACAGTGGAAGTGCATCAAATTGGGGCTAAAGATTTCTGGCTTAAGCTAGTTTGTGGCCAAGAGCGAGGGGTTTTTAAGAGGATTATGGAGACTCTCGATTCTTTGGAGCTTCAAGTAATTGATGTCAATGTCACTACTTGTTATGGCCATGTCTTGACAAATCTCAAAGTAGAGGCAAAAGGGAAGGAGGTTGTTGCAGCCAAGAGTTTGAAAGACTTGTTACTAAATTGTTGGATGCCTGGTATACATGACGAGAACCAAAGGCGCGGTTGA

AaMyc-bHLH22

ATGGCTTTGTTAAATACATTAGACTTGCTAAGACCACTTATATCAACCAAATCATGGGACTATTGCATTGTTTGGAAATTTACTAATGACCCTTTAATGTGTATTGAGTGGGTTGGGAGTTGTTGCAGTGGTAGTCAAGGTGTTTGTGGAAATGTTAAATGTGAAAATGAGGCTTATTTATGTAAAGATAGTTGTGTGAAGCATTTGATACGAACCGATGCTTGTGTAAAACTTGCTATGGTTCCGTCTTCTTTGCCGTTTTATCCGGGGATTCATGGGGAAGTCGCAGTGTCTAAACAACCATTCTGGCAAACTACTGATTTGAGTGGAAGTCAACTTGTTGTGCCTGTTGATGGCGGTTTGATTGAGCTCTATAGATCAAAACATGTTCCCACTGATGAAAGGACAATAGAAGCACTTATTGCACGCCTAAGCAACATTGTTGATCCTAAGTTAGACGAAGAAGACTCGAAAACTCGTCTGAATTCATATCTTTTCGATCACATTGGTCCTAACCTACAGCTCCTTATCCCGCTTCCAGAACTCATTAGTCCGCTTCCTGAACTCGTTAGTCATCCAACCACCCCAGGGTTGACATCAGGGAATGATAACGAAGTTATGGTGGCAAAGCAAAAGAAAGTGAAAAAACAATTCAAATCAAAGAATCTTGAAGCTGAAAGAAACCGAAGGAAACGAATTAAGGATAATCTCTTAATTCTACGTTCTTTAGTCCCCAAGGTTTCAAAGATGGATACAGTTTCGATAGTTGGAGACGCAATCAAGTACATACAAGAATTGCAAGAGAATGCTAAAGAACTTGAAGACGAGCTTAAGGCACTTGAAGAACAAGATTGCATGGTTAATGGTCATGAAGTCAAGGATGTGGAAGTGGAAGTACACAAAATCGGAGCTCGAAAGTACTTGCTTAAAGTGTTTTGCAGCCACAAACCGGATGGATTTTCAAGGTTAATAGAGGCTGTTCAGTCTCTTGGACTTCAAGTAATCAATGTCAGTATGACCACTTGTATTGGTCTTGTTTTAAACACACTTGCTGTCGAGGCTAAAGAGGAGATCGATGCCAAGAGTTTGAAAGACTCGTTGCTTAGCAGCTGGACTTCTTCAGAACTAGATCAAAAGCTCTGA

AaMyc-HLH1

ATGGAAAACTTGAGGCAAAGATTGGCTATGGCTGTCAAAAGCATCCAATGGAGCTATGCTATCTTTTGGTCAACCTCTTCAACAGAACAAGGGGTATTGACATGGTGTGATGGGTACTACAATGGAGATATCAAGACAAGGAAAACGATACAAGCAGAGGGAATGAACGAGGACGATGATGATGGTCAAGTGGGATTGCAAAGGACTGAGCAATTAAAACAACTTTATGAATCACTTTCAGCAGCCGAAACACATCATTATGAACCACAAGCAAGAAGGCCGTCGGCTGCATTATCCCCTGAAGATCTCACCGACACCGAGTGGTATTTCTTGGTTTGCATGACGTTTGAGTTCGGTTACGGTCAAGGATTGCCAGGAAGAACACTGGCCAAGAATACAACTAGTTGGTTGAGTGATGCCCACTTAGCCGATAGCAAGGTCTTCACTCGCTCCCTTCTAGCAAAAAGTGCATCAATTCAGACTGTTGTGTGCATTCCATATTTAGAAGGTATAGTTGAGTTTGGCATAACTGAGAGGGTTTTAGAAGAACAGAACATTATTCAGCGGATTAAATCTTTAATCTTTATAGCTCCGCCACAGAAGATTCATGAAATCCCTTTAGAAAGTTGTTCCGCTATGCTTGATCATGATCTTATCCACAACAATCTTAGCACTAGTCGCGCGGGTGATCTTTGGAGTGATGATGATAGTCGCTATCAGTGTGTTCTTTCCAAAATATTTAAGAACACCCAGAGATCGGCTATGGGCCCTGATTATAGAAACAGTGATTCTGAGAAATCCGCTTTCGTTAGCTGGAAAAATTATGATGGAATGGAATGGAAAGGAAGTTCTTCACAGATGTTGCTAAAGAATGTGCTTTATGAAGTCCCGAAAATGCACGAGAATCATTTAAGTCGGTACTATGACAAGAATGGAAATCTTGATCGGATGCAGGAAGTTGCGGTTGATGATGTGAACGACGTCAACCATAGGTTTTCGGTTCTAAGCTCCATCGTCCCTTCTAGAGGCAAGGTTGACAAGGTGTCTCTACTCGATGACACCATCAATTACTTAAAAACACTCGAGAGAAAGGTGGAAGTGTTACAATCCAGAAAGAAATCTCACGACGTTCGAGAAAGAACATCTGATAACTACGCCAACAAAAGAAAAGCTTCTTGTGCTCTTGAAGATATACAAGAAGAATGTTCTTCAGATTGTATTACAGTTAGCGCAATAGAAAAAGATGTTACAATCGAGATACGTTGCAAATGGCGAGAAAATATGATGGTGCAAGTATTTGATGCAATGAGCAGCCTAAACTTGGAATCCCACTCAGTTTGTTCTTCTACCGTTGACGGGATTCTCACATTAAGCATTGAAACTAAGTTAAAAAAATTTACGACGTCGACAGCAAAGATGATCAGGCAGGCACTTCAGAGAGTAATCGGTAGGTATTAA

AaMyc-HLH2

ATGAACGAGGACGATGATGATGGTCAAGTGGGATTGCAAAGGACTGAGCAATTAAAACAACTTTATGAATCACTTTCAGCAGCCGAAACACATCATTATGAACCACAAGCAAGAAGGCCGTCGGCTGCATTATCCCCTGAAGATCTCACCGACACCGAGTGGTATTTCTTGGTTTGCATGACGTTTGAGTTCGGTTACGGTCAAGGATTGCCAGGAAGAACACTGGCCAAGAATACAACTAGTTGGTTGAGTGATGCCCACTTAGCCGATAGCAAGGTCTTCACTCGCTCCCTTCTAGCAAAAAGTGCATCAATTCAGACTGTTGTGTGCATTCCATATTTAGAAGGTATAGTTGAGTTTGGCATAACTGAGAGGGTTTTAGAAGAACAGAACATTATTCAGCGGATTAAATCTTTAATCTTTATAGCTCCGCCACAGAAGATTCATGAAATCCCTTTAGAAAGTTGTTCCGCTATGCTTGATCATGATCTTATCCACAACAATCTTAGCAGTATGCTTGAATATGATCAACCCCTAGTAAGAAAGCCACATAATTCCCCAAAAAATAGTGTTGGTGCATTTCAGCCACATGAACAAAGTTGGCAATTTGTAGACGATGATGGTGATGATGATGAAGATGAAGAAGAAGAAGAAGGCGAGGTTAGTTATTACCATAACAATTCCATGGGTTCGAGTGATTGTGTATCTCAAAACTTAGCTAGTCGCGCGGGTGATCTTTGGAGTGATGATGATAGTCGCTATCAGTGTGTTCTTTCCAAAATATTTAAGAACACCCAGAGATCGGCTATGGGCCCTGATTATAGAAACAGTGATTCTGAGAAATCCGCTTTCGTTAGCTGGAAAAATTATGATGGAATGGAATGGAAAGGAAGTTCTTCACAGATGTTGCTAAAGAATGTGCTTTATGAAGTCCCGAAAATGCACGAGAATCATTTAAGTCGGTACTATGACAAGAATGGAAATCTTGATCGGATGCAGGAAGTTGCGGTTGATGATGTGAACGACGTCAACCATAGGTTTTCGGTTCTAAGCTCCATCGTCCCTTCTAGAGGCAAGGTTGACAAGGTGTCTCTACTCGATGACACCATCAATTACTTAAAAACACTCGAGAGAAAGGTGGAAGTGTTACAATCCAGAAAGAAATCTCACGACGTTCGAGAAAGAACATCTGATAACTACGCCAACAAAAGAAAAGCTTCTTGTGCTCTTGAAGATATACAAGAAGAATGTTCTTCAGATTGTATTACAGTTAGCGCAATAGAAAAAGATGTTACAATCGAGATACGTTGCAAATGGCGAGAAAATATGATGGTGCAAGTATTTGATGCAATGAGCAGCCTAAACTTGGAATCCCACTCAGTTTGTTCTTCTACCGTTGACGGGATTCTCACATTAAGCATTGAAACTAAGTTAAAAAATTTTACGACGTCGACAGCAAAGATGATCAGGCAGGCACTTCAGAGAGTAATTGGTAGGTATTAA

AabHLH1

ATGGACTCCATATTCGATCTTGAAGAAACAGAAAGATCTCGTTTGCTTCGGCAAATCATGGACTCCTTTGGTTTCACTTACATTTGCTTATGGTCTCACTTTACTCAGCCTTCCAATTGTTTGATCTGCATTGACGGGGTATACAAAGAAGGAAACAATCAAGCAAGTTCGTCATCTGGAAGTCTAACCATGACGTCTTTTCTTGACTACAAAAAGTTGATGTTCTTTATTGATAATTATACTGGAGGAGTTCCAGGGTTTGCATTCATGCACAATATTACATACATGGAGCGGAAAAAGTTGGAGCTCTTAACTTTGGCATCAAATTCTGCACAGCTACAATTTTATCAAGAAGCAGGGATTAAGACTGCAATATTTATGGGGAGCAGCAATGGAGAGATTGAGCTTGGCATGACCAATGACTCCTCTCAAATAAACTTTGAAATCGAGTTGAAGAAGTTGTTTCCAGGTGATTTCCGGGAAGGTGTGCTTCTTCAACGACTTGAGGAAGCTCGGGCTTCATCTTCTTCTTCATCTTTAAGATCATTATCAATGGACAATAGCGTGGAGAACTCGCCATTTCTCTTCAACATGTTTCATTCCACTCCTTATATGTCCGAAATGTTTGCTCTCACAGAACGCCAACCCCCTCGAGTTGCTAGTGCTTTTAAGAGATATGGATCAAGTAATTTAGGACCTATTAAACAACGAGTTCCAAATCGACAAAATCTTCACAGAAGGTCACTTTCATTCTTACGAAATCTAAGTGAGGCTCGTGCTCAAAGAGATCAAATGGTCCAAACAACTAGACCTACTAGCAATCAACTTCATCACATGATAGCGGAGCGAAAGCGGCGAGAAAAACTGAATGAAAGCTTTCAAACTTTGAGATCATTGTTTCCTCCAGGGTCCAAGAAGGACAAAGCATCAGTGTTATCAAACACAATGGAGTACATATCTTCTTTAAAGTCTCAAGTCGAGGAGCTTAACAAAAGAAATCAGATTTTGGAGGCTGATCAGCGTGCCAGGAAAGAACCACCTAATCAAGGTTCCAGTCGTTTTTCCGGAGAGGGTCCTGTAGTTGGCATCACTGACATTGGTGAATCAACTTCAGATTCACGAGTCGTTGACTTGGAAGTGAATGCAAGAGGGAATGTGATACTGGTGGATTTGGTGATGAGCGTGTTGGAGTTCATTAAACAGTCCGAGAATGTCAGTGTCATGTCTATAGATGCTGGGACTCGAATGTTGGAGACAGAAGCTATCGCAAATCGAGTAATTTTTAGATTAAGAATTCAGGGAAATGAATGGGACAGATCAAGTTTCGAAGAAGCAGTGAGGAGGCTTCTTGGTGACCTGGCACAATGA

AabHLH2

ATGTCGTTCATTGGTACATTGTCGTCGTCTACACATGAAGAGGTGGCACTGGAAAAAGGTTCATCTGGCGTGATCTCAGCTTCAAATTTAGAATCCAACTGTGGAAGAGGATTTTTATCTCTTCAGAATTTAGAATCCAACTGTGGAAGAGGATTTTTATCTCTTCAGGGTTTCTGTAAGCAAAGTTTCTTGAACATGGACTCCATATTCGATCTTGAAGAAACAGAAAGATCTCGTTTGCTTCGGCAAATCATGGACTCCTTTGGTTTCACTTACATTTGCTTATGGTCTCACTTTTCTCAGCCTTCCAATTGTTTGATCTGCATTGACGGGGTATACAAAGAAGGAAACAATCAAGCAAGTTCGTCATCTGGAAGTCTAACCATGACGTCTTTTCTTGACTACAAAAAGTTGATGTTCTTTATTGATAATTATACTGGAGGAGTTCCAGGATTTGCATTCATGCACAATATTACATACATGGAGCGGAAAAAGTTGGAGCTCTTAAATTTGGCATCAAATCCTGCACAGCTACAATTTTATCAAGAAGCAGGGATTAAGACTGCAATATTTATGGGGAGCAGCAATGGAGAGATTGAGCTTGGCATGACCAATGACTCCTCTCAAATAAACTTTGAAATCGAGTTGAAGAAGTTGTTTCCAGGTGATTTCCGGGAAGGTGTGCTTCTTCAACGACTTGAGGAAGCTCGGGCTTCATCTTCTTCTTCATCTTTAAGATCATTATCAATGGACAATAGCGTGGAGAACTCGCCATTTCTCTTCAACATGTTTCATTCCACTCCTTATATGTCCGAAATGTTTGCTCTCACAGAACCACATATGGACCAACAAGCACCAAATCAAACACCACAGTCAACAACAATATTGAGACCAGAAGATCCTCTGCGGCAAGCTTTAGAACAAATTAGAACCTCTCAATATGGATCAAGCAATTTAGGACCTATTAAACAACGAGTTCCAAATCGACAAAATCTTCACAGAAGGTCACTTTCATTCTTACGAAATCTAAGTGAGGCTCGTGCTCAAAGAGATCAAATGGTCCAAACAACGAGACCTACTAGCAATCAACTTCATCACATGATAGCGGAGCGAAAGCGGCGAGAAAAACTGAATGAAAGCTTTCAAACTTTGAGATCATTGTTTCCTCCAGGGTCCAAGAAGGACAAAGCATCAGTGTTATCAAACACAATGGAGTACATATCTTCTTTAAAGTCTCAAGTCGAGGAGCTTAACAAAAGAAATCAGATTTTGGAGGCTGATCAGCGTGCCAGGAAAGAACCACCTAATCAAGGTTCCAGTCGTTTTTCCGGAGAGGGTCCTGTAGTTGGCATCACCGACATTGGTGAATCAACTTCAGATTCACGAGTCGTTGACTTGGAAGTGAATGCAAGAGGGAATGTGATACTGGTGGATTTGGTGATGAGCGTGTTGGAGTTCATTAAACAGTCCGAGAATGTCAGTGTCATGTCTATAGATGCTGGGACTCGAATGTTGGAGACAGAAGCTATCGCAAATCGAGTAATTTTTAGATTAAGAATTCAGGGAAATGAATGGGACAGATCAAGTTTCGAAGAAGCAGTGAGGAGGCTTCTTGGTGACCTGGCACAATGA

AabHLH3

ATGGGATGGTGGGTTGAGTTGTTGTTGGTCTTTGGTATTTGTTTTTATCCGGCTATTAAATATGGGATGGTGGGTATATATTGTGTTTGGATGTACCTTATTTTTTGGATTTATTCGAGTTGGAGTCGCCGAAATCTGGGTTGGGGTCGTCGGAATCTGGGTTGGGTTGGTAGTGGTGTTGGACGGAGATTGTTTTCCGGAGAAGTTCAAGAATATGAAGAGGACGAACTTAGCACAGCACGGACTCATAGAAATGATTTAAAAGATACTGCAGATTCTTGTTCTTCAAGGCTCGTAAAGAGCAGGATGGCTTTATCGTCATTTTCCCATAAGTCAAGTGGTAGTAGTGAAAGAAAACGTGAAAAGATGAGGAAGATGGTGAATACAATAAGAGAAATTGTGTCTAGTGGAAAGCAAATGAATTCAGTTCCTGTTATAGATGAGGTTGTTAAGTATCTCAAATCTCTAAAAATAGAACTGCAGGAAGTAGGTGTCGGAATTTGA

AabHLH4

ATGGAAATTGATGAAAACTCAAACTGGCTTTTGGATTATGGATTGATGGATGATATTTCTACTGTTGTTGATTATGTTGCTCCACCTGCTGTTGTTGTTGGGTTTTCCTGGCCTTCTGACATACCTGCTGTCAGTTCTGAGATTGAAAGCTCATTTATCGACTTTGAAGGCCTTAAGGGGGCAGGATCACGGAAACGGTTGAAGTCTGAATGCAGCAATGTATGTGGGTCCAAAGCTGGCCGTGAGAAACAGCGTAGAGATAGAATGAATGAGAGGTTCATGGAATTGGGTTCGATTCTGGAGCATGGAAAACCACCAAAAACAGATAAGACTGCCATCCTGAGTGATGCTATTCGGATGATAATGCAACTACGAAGTGAAGCAGAGAGACTCAACGAGTCAAATGTTGATTTGCAAGAAAAAATCAAGGAATTAAAGGCTGAGAAGAATGAACTGCGTGATGAAAAGCAGAGGCTAAAAGTCGAGAAAGAAAAGCTGGAACAACAAGTCAAAACCATGAACATTGGTCAACCAAGCTATCTGGCACATCCCACTGCAATGAGGGGTGCATTTGCTGCACAAGAACAAGCTGCTGGAAACAAGCTTATGCCTTTTGTCGGTTACCCCAGTGTTGCCATGTGGCAATTTATGCCATCTTCTGTGGTTGACACATCACTGGATCACGTCCTTCGACCTCCAGTCGCGTTGAAGTCTGAATGCAGCAATGTATGTGGGTCCAAAGCTGGCCGTGAGAAACAGCGTAGAGATAGAATGAATGAGAGGTTCATGGAATTGGGTTCGATTCTGGAGCATGGAAAACCACCAAAAACAGATAAGACTGCCATCCTGAGTGATGCTATTCGGATGATAATGCAACTACGAAGTGAAGCAGAGAGACTCAACGAGTCAAATGTTGATTTGCAAGAAAAAATCAAGGAATTAAAGGCTGAGAAGAATGAACTGCGTGATGAAAAGCAGAGGCTAAAAGTCGAGAAAGAAAAGCTGGAACAACAAGTCAAAACCATGAACATTGGTCAACCAAGCTATCTGGCACATCCCACTGCAATGAGGGGTGCATTTGCTGCACAAGAACAAGCTGCTGGAAACAAGCTTATGCCTTTTGTCGGTTACCCCAGTGTTGCCATGTGGCAATTTATGCCATCTTCTGTGGTTGACACATCACTGGATCACGTCCTTCGACCTCCAGTCGCGTAA

AabHLH5

ATGTCACTCCAAAACGCCACAAACTCGTTCGATCCCGCTTCGTCTCACGATGATTTTCTCGATCAAATGCTTTCGGGCCTCCAATCTGGTGCTTCTTGGCCCGAAATATCAACCGGTGGTGGTGGTGGTAATGGTTGGGATGTAGATCAGTTTGATGATCAATCTAATTTTCTAGCAACAAAGCTTCGCCAACATCAGATCAGCTCTGGCCCGTCATCCGCTGCGAAATCGCTGATTCTCCAACAACAGATGATGATCTCTAGAGGCTTGGCTGGTGCTGGAGAGTTGGGGAATTTTCAAAATGATATGGTTGATGCTTCGTCTTTTAAATCTCCGAGAGGAGATAATTCGATTCAAACTCTTTTTAATGGGTTTGCCGGATCTCTCCAATCCCATCCATCCCAAGAGTTTCCATTTCCTCCGACCCAGAGCTTTGGATCTCCTGGGACCACATCAGCTTTGGTGAACCAAGGACAAACTAATGGTGGACCTCCCGCCACAACTGGTGGCGGGAGTGGACCACCTGCTCAGCCTAGGCAACGAGTCAGAGCTCGAAGAGGACAAGCAACCGATCCACATAGTATCGCGGAACGACTCCGGCGAGAAAGGATTGCCGAGAGGATGAAATCCCTTCAAGAACTCGTCCCAAATGCTAACAAGATATGTTTTCATGTGATTGCTATTACAAGTCCAAGAAAATTTAGTAGAATGAACTCCAAGGTTTACTCAACAAAGCTTCGCCAACATCAGATCAGCTCTGGCCCGTCATCCGCTGCGAAATCGCTGATTCTCCAACAACAGATGATGATCTCTAGAGGCTTGGCTGGTGCTGGAGAGTTGGGAAATTTTCAAAATGATATGGTTGATGCTTCGTCTTTTAAATCTCCGAGAGGAGACAATTCGATTCAAACTCTTTTTAATGGGTTTGCCGGATCTCTCCAATCCCATCAATCCCAAGAGTTTCCATTTCCTCCGACCCAGAGCTTTGGATCTCCTGGGACCGCATCGGCCTTGGTGAACCAAGGACAAACTAATGGTGGACCTCCCGCCACAACTGGTGGCGTGAGTGGACCACCTGCTCAGCCTAGACAACGAGTCAGAGCTCGAAGAGGACAAGCAACCGATCCACATAGTATCGCGGAACGACTCCGACGAGAAAGGATTGCCGAGAGGATGAAATCCCTTCAAGAACTCGTCCCAAATGCTAACAAGACAGACAAGGCTTCAATGCTAGATGAGATCATAGATTATGTCAAATTCCTCCAGCTTCAAGTCAAAGTCCTGAGCATGAGTAGATTAGGAGGTGCTGGTGCTGTTGCTCCTCTAGTTACTGATATCTCACCTGAGGGAGCCCGTGACCGTGTGCAAGTAGCCAGTGGTGGTGTCGCAGGACGGACCACTAATGGAACAACGTCCTCTTCAAACAATGAAACAATGAGTATGGCCGAGAACCAAGTTGTGAAACTAATGGAAGAAGATATGGGATCAGCCATGCAATACTTGCAAGGAAAAGGCCTTTGTCTAATGCCGATTTCACTAGCTACTGCTATCTCCACCGCCACTTGCAACCCTTCTTCCGCTAGGAACAACCACCCACTACTTGGAGTTGCGGATGGTAATAGCCCTTCATCTCCCAATCTGTCCGCATTGACTGTGCAATCGGCGAATGGAGGATTGCAAACGGAAGGAGTTTCCATTAAAGATACTACCTCTATATCCAGGGGTGTTTGGACATTAACAAATGGTTCAGGAACAGACAAATGGGGTGTTTGGGTACTGACAGAAGACGATGCAGAAGTAGAATCAAAGTCATCACATATATTTGAAGACTCAAAATGA

AabHLH6

ATGGCCAACATGTTTGATAATATTTGTTCTTCTTCATATTCTCCTATGTCGCATGAGCCCAGTGACGATATTTCGGTTCTCCTACGTCAAATACTTTCTAAATCATCTTCTTCTTCTTCCTCGTCTTCGTTGCTGACAAAACAACAACAACAACAACAGCCGCATTCTGCCGATGTGGCTAATGTAGCGGTGAATGTTGGTGCTATTGATTATGATCATTCTGATGAATATGATTGTGAAAGCCAGGAGGGGTTCGAAAACTTGATGGAAGAAATGGATGGAAAACCAAACCCTCCTCGTAACCCTTCAAAGCGAATGCGAGCTGCAGAGGTCCATAATATGTCCGAAAAGAGGAGGAGGAGCAAGATAAATGAGAAAATGAAAGCCTTGCAAAAACTTATCCCTAATTCAAATAAGACGGATAAGGCTTCTATGCTGGATGAAGCCATTGAGTATCTAAAGCAACTTCAACTTCAAGTGCAGATGTTAACAATGAGGAATGGGATAAATTTGTACTCTATGTATGCACCACATGGTAGTGTTCAGCCGAACAATAGACCATCCAATGTGAACCAAGGAAACCATTCCATCAACATGGCACCGAACCAAGAAAGGCTTGTGAACCCGATGCTTGGCCAACCTATTCAATGTATGAGCCAGAACAAGCAATCAATACTTGACTTTTCGTGCACAATCAATCAAGAGCCTCCATTTGGAACTCAGCTTGGTTCGTCCTAG

AabHLH7

ATGGCGGATCTATACGGAAATGATCATCGTTCATTTTCATCATCTTCTTCACTTGAATCTGAAGATATGTCTTCTTTTCTACAAACTTTTATTAATAATAATAATGGTTCTTCAGCTAGTAACAAATATGGAGGGGGAGGACCTTTGATCCCTTCTCCTGTGCCTGAGTTTCATGATTCTGACATCCGGTTCTCCGATCTCAGCTCGTTTTATTCGCCCGAGCCGAATCAGGTGCAAAAAGTGTCGGATGTGCGTCAAAGTTCGAACCCTACTCGTTCCTCAAAGAGGACTAGAGCTGCTGAGATTCATAATTTGTCTGAGAAGAGGAGGAGGAGTAGGATTAATGAGAAGCTGAAAGCATTGCAGACGTTGGTTCCGAACTCTAATAAGACTGATAAGGCTTCAATGTTGGATGAGGCAATTGAATATCTGAAGCAGCTTCAACTGAAAGTGCAGACACTTGCTATGAGAAATGGGTTTGGATTACAACCTGTATTCTCACAAGAACAAGAAATGCAAAGGGGAATATGCTACGATGAGGGATACAAATTTGTAAGTTCATCTGATGGGGCTGCTGGTACATCTTCTCAAACCCAAGGATTCTCAATGCGAAGGGAATTTGGTATGCCAAACCACCAAATCGGCATGATGATGAAACCAAATCCTGTTTTTGGTTCAGAACTTGCAAATGAGAATCAATATGGATTTACCAATCATTCCACGTCAGTGAAGGACACAAGCAGATCATTCTCAGGAAAAGGAGTCTCGTCATAG

AabHLH8

ATGCTTTCTGGTCTCCAAACAAACGTATCTTGGCCAGACATTTCCAACGGTGGTGGTCAAAACAAACCGTCACTTCCATGGGACGTTGACCATTTTGACGATCAGTCTGCTTTCTTATCATCTAAGCTCCGTCAGCACCAGATCACCGGTGGTGCTCGTACTCCTGTCTCACCGTGCATGTCGAAACCAGTACTAAAAGCTATCGAGGCCATTTTTCGTAAACTAAACAAGACATGTAACATAAATATTTTTGACATGATTGGAGAGAATAACAATAATAATGCTATTCAGAATTTGTTTCATGGGTTTACTGGATCTCTAGCAACGAATCAAGGCCAACAATTCCGTAATCTGCCGGCCCAAAATTTTGGATCTCCGGCAGCGGCAGTTGCTGCAGCGGTTATGAACCAAGTGCAGGCTGGAAGTGTGACCGCAGCTGGCGGCGGTGGTGGTGGTGCGCCATCGCAGCCACGACAAAGAGTTCGAGCCAGACGCGGACAAGCAACTGACCCACACAGCATTGCTGAAAGATTACGACGTGAGAGGATTGCCGAGAGGATGAAAGCGCTACAAGAACTGGTCCCAAATGCTAACAAAGAATCACAAATGTCTCTTCAAGATCTCCAAAACAACGAAACCAGCAACAACAACAACACGTTTGATCCAACTGCCGCTCACGATGATTTCCTCGATCAAATGCTTTCTGGTCTCCAAACAAACGTATCTTGGCCAGACATTTCCAACGGTGGTGGTCAAAACAAACCGTCACTTCCATGGGACGTTGACCATTTTGACGATCAGTCTGCTTTCTTATCATCTAAGCTCCGTCAGCACCAGATCACCGGTGGTGCTCGTACTCCTGTCTCACCGTGCATGTCGAAACCAGTACTAAAAGCTATCGAGGCCATTTTTCGTAAACTAAACAAGACATGTAACATAAATATTTTTGACATGATTGGAGAGAATAACAATAATAATGCTATTCAGAATTTGTTTCATGGGTTTACTGGATCTCTAGCAACGAATCAAGGCCAACAATTCCGTAATCTGCCGGCCCAAAATTTTGGATCTCCGGCAGCGGCAGTTGCTGCAGCGGTTATGAACCAAGTGCAGGCTGGAAGTGTGACCGCAGCTGGCGGCGGTGGTGGTGGTGCGCCATCGCAGCCACGACAAAGAGTTCGAGCCAGACGCGGACAAGCAACTGACCCACACAGCATTGCTGAAAGATTACGACGTGAGAGGATTGCCGAGAGGATGAAAGCGCTACAAGAACTGGTCCCAAATGCTAACAAAACAGACAAGGCCTCAATGCTTGATGAGATCATAGACTATGTCAAATTCCTCCAGCTCCAAGTCAAGGTTTTGAGCATGAGCAGATTGGGAGGTGCCGGTGCTGCTGCAGGCGCGGCTAATCCCATGGCTGCTGAGGGTGGTGGCGATTGTGGACAAAGAGGTGGACCGGGAACAGGACGGAGTAGCAACGGAACAACGTCATCATCTAACAATGAGACAATGACAGTAACGGAGAACCAAGTGGTGAAACTAATGGAAGAAGATATGGGTTCCGCAATGCAATATTTGCAAGGGAAAGGTCTTTGTCTTATGCCTATTTCACTTGCTACGGCTATTTCCACCGCTACTTGCCACCCTTCTGGCACCCGAACTAATCATTTGGCAGGTGGAGAGGGTGGACCAACGTCACCGAACATGTCTGTGTTGACTGTTCAGTCGGCGAATGGGGTGAAAGATTCACCTTAG

AabHLH9

ATGTACAACACACAAGCAACTTCTTCTTTACCAGAAACAACAGACGACATTTCGTTGTTCCTACGTCAAATCCTTCTTAAATCATCATCCTTAAGTTCAGCAACAGCAAGCACAACAACAAAACCAACAATGTTGTGTTCTTCTGTTGTTGGTGCTAAGCAAATGCAATGTGAAATGCCACGTGTACAACCTGTTTATTCGTCTGGTTTAGTATCGGTTCCGGAGTGGGGTTGTTCTGATCTGGTTGGTGGTTATGCTCCGGTGACATATGTGTCTTCTTCTTCTGTGGGGACCTTGGATAATGAAGTTGATGAATTTGATTGTGAAAGTGAGGAGGGTGGTTTTGAAAATTTGTTGGAAGATATGGCGACAAAATCAAACCAAACTCCTCGAAATCCTTCGAAACGAACTAGAGCTGCAGAGGTTCATAATATGTCAGAAAAGAGAAGGAGAAGCCGCATAAATGAGAAAATGAAAGCGTTGCAGAAACTGATTCCTAATTCAAATAAGACGGACAAGGCTTCGATGTTGGATGAAGCCATTGAATATCTGAAGCAGCTTCAGCTCCGAGTACAGATGTTGACAATGAGGAATGGAATAAATTTATATTCCATGTCAGTACCACCTGGACTTCTACAGCCTCAACAGCTCCCATATTCACGAACCGGCTTCAATGAAGGAAATGATCCCCCAAACATGACCCGACTGAACCAGGAACCACACATGAATCCCATGCTCAACCTCCCAATTCAATCCACCAATCGAAGCCAACCATCAATCCCTAATCTTTCACATATAATGAATCAAGGGCCGTCATTGTCTTTCAGACCTCCACTTGGACCCTTTAAGGAATGCTTGATAAATAGCAGTGTATCTGATGGTACTCAACTGATGAATTCGGAGCAGAAATTGGTCCTTCCCGATAATTTAAACGGGGGTTTGCACCAGAAATGA

AabHLH10

ATGGAGCATCCTCAACAACGTTTGTTAGGTACTCAAGGTAGAAAACCAACGCATGATTTTCTTTCGCTTTATTCACCTGCTCAGCAAGATCCATCTGCTGCTACTACTACTCCTTCAGGTAGCTACCTCGAAACTCATGACTTCTTGCAACCATTGGAGCGGGTCGGGAAAAATGTTGCTAAAGAAGAAAATAAAGTTGAAGAATCATCCATCGGAAAGCCCCTACCACCAACTCCCCCAACTACAGTGGAGCATATTCTTCCAGGTGGGATCGGGACATATAGTATTAGTCACATTTCTGTAATCAATCAAAGCCAAAGGATGCCAAAGCCTGAAGGGGTCGGGGTCGTGATCACGGGTGCACAATCTAGTGGTAGTGATAAAAACGATGAAAATTCAAATTGTAGTTCGTACACCGGAAGTGGCTTTTCACTATGGGAAGAATCTGCAGTCAAGAAGGGAAAGACAGGGAAGGAGAATATTGCTGGAAATCGGCATGTGATTAGAGAAGGGGGCATGAAGATGGGAGGAATGCCATGGATGACATCTATGGAGCGGCCATCACAGTCGTCATCTGCCCATAACCATCCAACCGCAACCATCAGTTCTCTATCGTCTTCTCGTCCATCATCAGCCCAGAAGAATCCGAGTTTTGTTGATATGTTAAAGTCTGCCAAGAGTGTGCAAGAAGACGAAGATGAAGAAGTAGAAGAGTTTGTTATCAAGAAAGAACCATCCACACACTACAAAGGTGTTTTGTCTGTTAAAGTTGACACAGCACATCATGATCAAAAGCCCAACACCCCGCGATCTAAGCATTCTGCAACAGAGCAGCGTAGAAGAAGCAAAATCAATGACAGATTTTCAATGCTAAGAGAACTCATCCCTCACGGTGATCAAAAGAGAGATAAGGCCTCGTTTTTACTAGAGGTTATTGAATACATTCAGTTTCTACAAGAGAAGGTACACAAGTTCGAGGACTCATGCCGGGGATGGCCTAATGAACAACCTGCAATGACCCCATGGAACAACAACCAAAGACCAACAGAAGGGTTTATTGATCAACCACGAGTCCAAAACGGTGTGTCTGGCTCAGCATTGCCATATGCTTCAAAACTGAACGAGAACAAGCACAGTGTTGCCTTAAACCTGCCCAAAAAAGATCACCAAAACATATTAGATTCTGATTTAAGTTCCCTCGAAACCATGAAGGACATTGGTCAACATCCTCGTTTGACCAATTTGGCATCTCCTTTCCCATCACCCTTGCAACCAAACGTGTATTCACCTGGTTGTGGAAGCACGAGTGTCGCTGCACCGCCTCCTTCAACTCTGCCATCTGATACAACAAACACACCATCCCAATTATGGCAAAGCAGATCATGCACAACTGACTGCACCGTTGCTGGTGATAAATCAAAAGACCAGGACCTGACGATTGAAAGTGGCACAATTAGTATATCAACCATCTACTCTCAAGGGTTGTTGAGTACGCTAACACAAGCACTACAGAGCTCGGGCGTGGACTTATCAGAAGCCAATATCTCAGTACAAATCGACCTTGGGAAAAGAGCAAATACTAGTAGACGCGATTCCTCAACACCCATTTTTAAGAAAAACGAAGCTCCGGTCATAGATGAAAACCTGACACGTTCGCGACTTGCTAGCACAAGGGAGGAGAAGAACGACAATGATTTGAAGAGGTTTAAAACGAGCAGAAATTAA

AabHLH11

ATGTCCTCCTGGCTTCATTACCCGGTAGAATATCCTGCTAACGAAAATTCATTAGAAGGATATTTGTACAATAACGATCTTTTGTTTCCAATACCACCACCAAATCCGGTTACTACAACGCCTATGGCGCCTACTACTTTACCGCCACCTCCTCCTCCTTCGGTTGTGATCTCTTCTCCTCGTCCACCAGTTGCGCCTATACGGCGCAACCATGTGGACATACAACCTAGACAACAACCGAAGTACCCTAACTTTTTGCACTTTTCTAGGCCTAATAAAGTCACAACTCTAGAGTCAGGTGCTTCAGCGCCAGTGACAGAAGCACCTGAATCTAGAGCTTCGCGTGTGTCAGAAAAACCACCGCCTACTTCTGTAGGCGGTGAGAGCGTTAGTGGTGTAGGTTTAGTAGGTACGTCATCTATGGGTAGGGAAGTTGAGACATGTGATACAAGCATGATGTCATCGCCCGATGGGTCGGGAGCAAGTGGAAGTATCGAGCCATCGACTCAAATGCCACCTCCTTTGACGAATGACCGTAAGCGCAAGGGTCGCGACACCGAAGACACCGAATGTCATAGTGAGGACGTCGAGTGTGAGTATCCTGAAGCCAAGAAACAATCTCATGGATCAACTTCTACAAAGAGGTCGCGTGCTGCAGAAGTCCACAATCTCTCGGAGAGGAGACGTCGAGATAGGATTAATGAAAAGATGAAGGCCTTGCAAGAACTAATACCTCGCTGCAACAAGTCCGACAAAGCTTCAATGTTGGATGAGGCAATTGAGTACTTGAAGTCGCTTCAAATGCAAGTTCAGATGATGTCCATGGGATGCGGCATGGTTCCTATGATGTTCCCAGGTGTCCAACAATATGTTTCACCAATGGCAATGGGGATGGGAATGGGTATGGGTATGGGCATGGACATGGGAATGAATCGACCCATGGTTCCTTATCCCGCCATTCTTCCAGGCCCATCAATGCCCAATCCAGCTGCATCTGCTGCAGCAGCCGCTGCTCAGCTTGGTCAACGGTTTCCTGTTCCAGGATTCAACATGTCACCCGTTGCCGTTGCGGGTCAAGCGGCTAATATGTCAGCTCCAATGATGAGTTCATTTCCGCTACAAAATCAAAATCAACCACGAGTTCCCAATTTTGCAGATCCATATCAACAATACCTTGGCCTCCATCAGACACAAGTACCGCTTCCACAGAACCAAGGAGGCATTCCCCCAACTGCAACTAAACCGGGCAGTAGCAAAGATGCTACCAACCCTGATCATCACCAAAATGGTTGA

AabHLH12

ATGGCATGTGAGTCTGTTTTAATGGCGTATGGGCCTGATTGCATTGGAGCCTGGTGGCGTTTGAGCCTGATGGCGTTTATCATGTATGATTTGTACTTCAAAAGTTTAGAAGTGATTGCAATGAAACCTGATGAAATAGCCTTTGAGCTTGACTGGATGTTCTTGACCGAGGGGTTGTGTTGTTTATGTGAGGGGAATGTCCAGGTTGAGGTGTATGTGTTAGTTGTTGTGATTTGTTTTGTTGTATGTGAGATTTTCGGGTTTATGTCATGGGATCAGGTTCCAAGGGCTAGTTTTGTGAGGCGTTTCAATGTGATCTCAGGCTCCTGTGAGGAGGATTTTTTCATGTTTTTTAAACGAAAAACAGGTTTCGGCTTGTTGCTTGGAAACAAGTGTTTCATGGTGAAACCTTCGTTTTCTGGGAAGCAGAAACTGTCATTGTTTCGCTTGTTTTCCGGCGATGAATATATCGAAAACAAAGTTCTCAATTGTCGGAAAATCAAAAACGGTGAGGGTGGTTTTGAAAATTTGTTGGAAGATATGGCGACAAAATCAAACCAAACTCCTCGAAATCCTTCGAAACGAACTAGAGCTGCAGAGGTTCATAATATGTCAGAAAAGAGAAGGAGAAGCCGCATAAATGAGAAAATGAAAGCGTTGCAGAAACTGATTCCTAATTCAAATAAGACGGACAAGGCTTCGATGTTGGATGAAGCCATTGAATATCTGAAGCAGCTTCAGCTCCGAGTACAGATGTTAACAATGAGGAATGGAATAAATTTATATTCCATGTCGGTACCACCTGGACTTCTACAGCCTCAACAGCTCCCATATTCACGACCCGGCTTCAATGAAGGAAATGAACTCCCAAACATGACCCGACTGAACCAAGAACCACACATGAATCCCATGCTCAACCTCCCAATTCAATCCACCAATCGAAGCCAACCATCAATCCCTAATCTTTCACATATAATGAATCAAGGGCCGTCATTGTCGTCTTTCAGACCTCCGCTTGGACCCTTTAAGGAATGCTTGATAAATAGCAGTGGATCTGATGGTACTCAACTGATGAATTCGGAGCAGAAATTGGTCCTTCCCGATAATCTAAACGGGGGTTTGCACCAGAAATGA

AabHLH13

ATGGAGCTTTCGCAACCCCGTCCCCGCGGAGAGCCAGGTGCGAAACCCACGCATGACTTTCTTTCGCTTTATTCACCTGCTCACCAAGATCCGTCCCCTACCATTCCAGGTAGTTACCTTAAAACGCACAACTTCTTGCAACCACTAGAACAAGTTGGAAAGACTGTTTGCGAAGAAGTGGAATACATTAAAAAGTCTTTCCCACCATCTCCACCAACTGTTGGGGAACACATTCTACCCGGTGGGATGGGAACTTACAGCATTAGTCACATTCCGCATATTAATCAAACCCAAAAGGTATCAAAGCCTGAAGGGATTGTGATATCGGCTGCACAGTCAAGTAGTAGCAATAACAATGATGAAAATTCAAACTGCAGTTCTTACACAGGGAGTGGTTTTACGTTGTGGGAAGAATCTAATGTAAACAAGGGAAAGACGAGGAAGGAGAATAATATTGCCGCAAATAGGCATACAATGAGAGATGGGGGTATGAAGTTTGGAGTTCCATGGATGACATCGATTGAGCAGCCATCAAAGTCATCATCCATAAATAATCATCCGAGCACAACGTTTAGTTCCCTCTCATCCTCTCATCGATCATCAGCCCCTAAGAGTCAGTACTTCGTCGATATGAAGTCTGGTAGGAATTTCCAAGAAGTCGAAGACATTAATGAAGGGTTTGTTATTAAGAAAGAACCATCCTACCACCATAAAGGTGGTACGTCGACTAAAGCTGACACTTCAAATTCTGATAAGAAGCCTAACAGCCCACGCTCTAAGCACTCTGCTACAGAACAGCGTAGAAGAAGCAAAATTAATGACAGATTTTCAATGTTGAGAGGGATCATTCCACATGGAGATCAAAAAAGAGATAAGGCGTCGTTTTTGCTAGAGGTTATTGAATACATTCAGTTTCTACAAGAGAAGGTGCACAAGTATGAGGACTCATCTCAAGGATGGACCAATGAACCACCAAAAACAATTCCAATCAACGACTTCATTCATCAACCCCAAATCCCAACAAATCAAAACCTATTAGATTCTAACATGATCTCTCATGATGCTACTAACGAAACCAGTCAACACCCACAATCAACAAATAAACCATCTGCTTCTGCTGCAGCCTCTTATGAACAGATTTCCACTCCTGATACAGCAACCACAGCCTCACAGGCTCAAGAACTGATAATTGAAGGTGGCACAATTAGCATTTCAACCATCTACTCTCAAGGATTAGCTAGTGCTCTAACGCAAGCACTAAATAGCTCGGGTGTGGATTTGTCACATGCTAATATCTCGGTACAAATTGATCTTGGAAAGCGATCAAATGCAAATACAAATACACTTGAATCCTCACCACACGATCTCAAGGAGAATGAGACTCGTTCCAATGATCAGTCAATAGCACATTCGAGACTTGTAAGCACATGGGAAGACGAGAATGACCAAGGTTTCAAAAGGTTGAAGACAAGCAGAAATTGA

AabHLH14

ATGAATCAATGTGTACCGAGTTGGGATTTGGAAGACTTCAATTCTAATCTCGACATTTTCAAATTAGGTTATGAAGTAGCAGAATTGACATGGGAAAATGGCCAAGTCGCCATGCACGAATTAGGTCACCGGCGCGTGCCATCTAAATCCCAACCAGAAACCTCGTGGGACAAGCCACGCGCCGGTGAAACCCTTGAAGCTATTGTAACCCAAGCCACCTACCAACCCTACTGCAAGACCCATGTGGTAGTTAACGACAACGAGCTGGTCCCATCTGCCACAATGGCTTCAGACGCATTGGTACCAAGTGCTCGTGATGCTGGTTGTTCAACACATGTCGGTTCATGCAGTAATGACCCATCAGCGTTTTTAAACGAGAGGGTGGCTTGTGGTGGTGATGATGGTGGTTGTGCGACGGTTAGATGTCATGATATGACCATGAGTGGATGTGGGACTTATGAGACGTTTGATACTTATGATGGCGATACGGGTGGTCAACGGTTGATTGAGACTTCAATGGGGTCGCCAGAAAATACTAGCTCCGGTGGAGATTGTTTAAAGTCAAGGTCTCCTGATGACTCAGCTTGTCATTGTAGAACTAAGGAGGTGAACGTAGTTACGGAAAAGAAAAGGGGGAAATCGGAATCTTCGACATCAACTAAGAGAAGAAGGACAGCTGCAAATCATAATCAATCTGAACGGAAACGAAGAGACAAGATTAATCAAAAAATGAAGACTCTACAGAAGATGGTTCCGAACTCAAACAAGACAGATAAAGCGTCTATGTTGGATGAAGTAATAGAGTACCTAAAACAAATGCAAGCACAAGTTCATATGGTGAATAGAATGAACATGCCACCAATGATGATGCCATTAGTCATGCAACAACAACAACAACAGCAACAACAAATGCAAATGTCTATGATGAACTCCATGGGCCTCGGCATGGGGATGGGAATGGGAATGGGAATGGGGTTCGGAGGTATGGATATGAATACCATGGCCCTGTCTCACCTTCCAACCGGTTTTCACCCAACCACTTTCATGCATATGCCTTGGAACAACCACATCACTGATCGGGTTGTCAATTACGGCCCAATGGCTGGTGATCCCATGTCTGCATTTCGTCTGAGCAGGTCTCAGCCAATGAACATGGATGCTTATAGTAGAATGGCAGCCTTGTACCAATATATGCAAAATCAATCGAGTGGGTCACATCCAAAGAACTAA

AabHLH15

ATGGCTGAAAGATTAAGGGGAGATGAAGATATAATGGAATTACTATGGCAAAACGGACAAGTTGTAATGCAAAGCCAAAACCAAAGATCCAACGACGAGGATACGGCGCCGTGTAATCTCTATATGCACGAAGATGAAATGGTGTCATGGCTTCATTACCCTAGCGATGACAATAATAATCTAGACTTATATTTACATAACAATGATATTTTATATCCAGTTCTCCCTGCAGCGAATGTCGCGCATTCGTTGCAGGGAGTTTCGGCTATTTCTATTCCTCCGCCGCGCGCGTCATCAGTCGTGACGCGCGTGGGAGAGGAACAGAGTCAGAATAAGTTTGGAAACTTTTCGCATTTTTCTAAACCTAGTTTTGTAAAGAATAATCCCACAGCTGGATCAGCTCCGACAAGTTCTAACAAGATGTCGGAGGTGACTACAGTTGTGGAATCTAATAATCAGCCAATGGAAATGCAAAAGTTACGAGGAGGGATGATGACGTCATCTTCGGGGAGGGAAGTTGAGACCTATGATGTCAGCGCGTCGACCTCGTCGCCAGGTACAGGTGGCTCTGGTGCGAGTGCGAACGCTGAACCGGCGGCGGGAAAGAAATCGCCGCCGGTGGCTGAGGATAGGAAAAGGAAGGGGAGAGATGTTGCCGAGGAGACTGAATGTTACAGCGAGGATGTCGAGTTTGACTATCCTGAAGCAAAGAAGCAATCACGTGGATCAACGTCTACCAAGAGGTCTCGTGCTGCAGAAGTCCATAATCTCTCAGAAAGGAGACGTCGAGATAGAATAAATGAAAAGATGAAGGCCTTACAAGAACTGATACCTCGTTGCAACAAGTCTGACAAAGCTTCAATGCTTGATGAGGCAATCGAGTATTTGAAATCACTCCAAATGCAAGTGCAGATGATGTCCATGGGATACGGCATGGTTCCTATGATGCTCCCAGGTGTCCAACGTCAACGATTTCCAGTTCCACGGTTTCCAATGCCACAGATTCCCGCCATGGGACCGGCAAGAAGTCAAGACCCGATGATGAACTCGCTTCCACCACAAAACGCAAACCAGCCAAGGGTTCAATTTGCAGATCCATATCAGCAATACATTAGTCTTCCCCAAACACAACTGCCACAACCGCAGAATCAAGCAGGAACGCGAACGCCTCCAGTTACAAGTATGCCAAGCAGTAGCAAAGATGTTAGGGATCCAGGGCATCAGCCAACAGGCTGA

AabHLH16

ATGAATCATTGTGTTCCGGATTTCGAAAACGAAGAAGACTACTTGCTTCCAGCTTCCTTAAACTCGAAAAGGCACAAGAAGTCAACAATGGGAGATGAAGACATAATGGAATTATTATGGCAAAACGGACAAGTTGTAATGCAAAGCCAAAACCAAAGATCCAACGGTAACAACAACAACAATAAGAAGATAGAAACACAACCACCATTGTCAACAACCACCGCCGTAAACCACCATAACAACAACAACCGATCCACCGTACTAGACGACGAAACGACGCCGTGTAACCTCTATATGCACGAAGATGAAATGGTGTCATGGCTTCATTACCCTAGCGATGACAATAATAATCTAGACTTATATTTACATAACAATGATATTTTGTATCCAGTTCTCCCAGCAGCGAATGTTGCACCACATTCGCTGCAGGGAGTTTCGGCTATTTCTGTTCCTCCGCCGCGCGCGTCATCAGTCGTGACGCGCGTGGGAGAGGAACAGAATCAGAATAAGTTCGGAAACTTTTTGCATTTTTCTAAACCTAGTTTTGTAAAGAATAATCCCACAACTGGATCAGCTCCGACAAGCTCTAACAAGATGTCGGAGGTGACTACAGTTGTGGAATCTAATAATCAGCCAATGGAAAATATGAAAAAGTTGCGAGGAGGCATGATGACGTCATCTTCGGGGAGGGAAGTTGAGACGTATGATGTCAGCGCGTCGACCTCGTCGCCAGGTACAGGTGGTTCTGGCGCGAGTGCGAGCGCTGAACCGGTGGCGGGGAAGAAATCCCCGCCGGTTGCTGAAGATAAGAAAAGGAAGGGGAGGGATGATGTCGAGTTTGACTATCCTGAAGCAAAGAAGCAATCACGTGGTTCGACGTCTACCAAGAGGTCTCGTGCTGCAGAAGTCCATAATCTCTCAGAAAGGAGACGTCGAGATAGAATAAATGAAAAGATGAAGGCCTTACAAGAACTGATACCTCGTTGCAACAAGTCCGACAAAGCTTCAATGCTTGATGAAGCAATCGAGTATTTGAAATCACTCCAAATGCAAGTGCAGATGATGTCCATGGGATATGGCATGGTTCCTATGATGCTCCCAGGTGTCCAACGGTACATGCCACCAATGGCTGCTATGGGTATGGGAATGGGAATGGGAATGGAACATGTAGGAATGAACCGACCCATGGTTCCATATCCAGGAGTTCTTCCAGGTCCACCCATGCCAAATCCAGCAGCAGCGGCTGCAGCAGCCGCTCATCTCAGTCAACGTTTTCCAGTTCCACGGTTTCCAATGCCACAGATTCCAACCATGGGACCGACAAGAAGTCAAGATACGATGATGAACTCGCTTCCACCACAAAACGCAAACCAGCCAAGGGTTCCATTTGCAGATCCATATCAGCAATACATTGGCCTTCCCCAAACACAAATGCCACAACCACAGAATCAAGCAGGAATGCGAACGCCTCCCGTTACAAGTACGCCAAGCACTAGCAAAGATGTTAGGGATCCAGGGCATCAGCCAACAGGCTGA

AabHLH17

ATGCCGGGGACATATGTAGCAAAACCAGATGTCGTAAAATTTTCAACAAAACCTATACTCCCAAGTCCCAACGCATTATATCGGAAACTTGATTTGGATAAAGCTCACGGCCACATATTGCGTTATGGGTTTTTACCCGGGTATACAGAATGGACTGTACATGGAGAGCATACTATATCTTTAGCACCATCTCAATCTAGTTATGTTAATGTTGAGGAAACTTCTTTAGGTCAAGAGGACATAATAGGTTTGGTTCGTGATGCCTTAGGTATTAATTCTTTGCCTTCTGATAACACACAATTAGGAGATACAACGATGGAAGGAGATACGGGAGAATCCACCAAAGCTGATGATCATGGTGATGAGGGTGTTTCATATAAGAAGTTGTTAGAAGAATGTGACAAAGAATTATCACCGAATGATTTAGAGTCATTTGATATCTGCTATAAAACGACTGATGACACATATATTCAAGAAGCAACAGCAGAAATGATGGTTATAGCAAATCAAGAAATATCAAGGAAGAAATTAGAGCTAGTAGGTCCCGAAGGTAATATTGAACCTGCCTTAGAGGCAGAAATTGCTAGGGAGGTTCTTAACAAACTGTTTGGAAATGAAGAACCTCGATGTTTTGGGGCCGGTGTGACAAAATCCCAAATAACTAAGTTTTGTTGTGATCTAAGAATGATGAGGGGTGAAGTACTAGCAAATGAAAACCGTTTTCTGTTGGAGAAGGTAGACAATCAAAGTAAGGAAATAGCGACTCAAAAAAAGCAATTAGAGACTCAAAAGAACAAAGTTGAGTCTTATAGTAAGCAAGTAAACACTCTAGTTTCGCAACTGAATAATATGGGACAACAGCTGAATGAAGTTTATGGAATGTTGAAAGTTTTTCAAACCGCGTTTCCAGATCTATATAATACTGCTTCTACTTCTGCTGCTGCAAGTACATGTGACAAACAACCCTCTTCTAGCGCATCACCCATCATGGATCATTACTCACCTGTCATGGATCACTACCCGGAACTGATCTTCATTAAGATTAAGGCTATTAAAAATTGGTTGACCTCTTGGTTAGTCTTTATGCCAATATGTAGCTGTAGAAATGAACGGTTATATCTGTTACGAAGAAAAACAGGCCATCAAATGACCAAACTTCTGATAACAGCCTTTAGCCATGACTGGGGAGGAGATGAAGATATCATGGAGTTACTATGGCATAATGGTCAAGTTGTTATGCAAAGTCAAAACCAAAGATCTAGCGGGAGCAAGAAGCTAGAAACAAAGCCAGCGGTCCGATCAGCTGAGCAAACTGCTCATCAAACCGGACCTTCAGACTTGTTCATGCAAGAGGACGAAATTTCCTCTTGGCTTCATTACCCTATTGAATATCCTGCTAACGAAAATTCGTTAGAAGGATATATATACAATAATGATCTTTTGTTTCCAACACCACCACCAAATCCAGTTACTACAGCGCCTATCACGCCTACAACTTTACTGCCGCCTCCTCCTCCTTCGGTTGTAGTCCCTTCTCCTCGTCCCCCAGTTGCGCCTATATGGCGCAACCGGGTGGACATACAACCACAATCTAGACAACAACCGAAGTATCCTAATTTTTTGCATTTTTCTAGGCCTAATAAAGCCAGAACTCTAGAGTCAGGTCCTTCTGCACCAGTAACAGAAGCACCTGAATCTAGAGCTTCGCGTGTGTCAGAAAAACCACCGCCTATTTCTGCAGGCGGTGAGAGCGTTAGTGGTGTAGGTTTAGTAGGTACGTCATCTATGGGTAGGGAAGTAGAGACATGTGATACAAGCATGATGTCATCGCCCGATGGGTCAGGAGCAAGTGGAAGTATCGAGCCATCGACTCAAATGCCACCGCCTTCGACTAATGACCGTAAGCGCAAGGGTCGCGACACCGAAGACACCGAATGTCATAGTGAGGACGTCGAGTGTGAGTATCCTGATGCCAAGAAACAATCTCATGGATCAACTTCTACGAAGAGGTCACGTGCTGCAGAAGTCCATAATCTCTCAGAGAGGAGACGTCGAGATAGGATTAATGAAAAGATGAAGGCCTTGCAAGAACTAATACCTCGCTGCAACAAGTCCGACAAAGCTTCAATGTTGGATGAGGCCATTGAGTACTTGAAGTCGCTTCAAATGCAAGTTCAGGTAAATAAACCTTAA

AabHLH18

ATGGAAGATGGTGGGTTCATGACTCAATATGATAACATGTGCAAGCCATATGATATGGTTGATAAACTAAGTGTTGATTCTATTTCCTCAGAAAACATTCTGGAAAAAGAATCTTCCATCGATAGATTTTTTCAGACTCCAAGTAGGTTTGAAGAACCAACCGAAATAAATTTGCTTAGTTATCAAAAAGCCAGTAACATTAATCGAAGGTCTAGTACCCCAAACACTATTGCTGCTACTACTCATTCTTCCTTCAACACTTTCACCATATCCTTTAGAGATACAAAGGCTAAAGAAGAGATCCATCCATCTGATGATTCACTCGGTTATGAATCTGCTGGTACTGGAAAGGCTCCAATCATTGCCAGGACTCCACTTCAGGCTCAAGATCATGTGTTGGCTGAGAGGAAAAGAAGAGAAAAGTTAAATCGGCAATTCATTTCTATGTCTGCCCTCCTTCCAAACCTTAAAAAGATGGACAAGGCATCTGTGTTGGAAGATGCAACAAACTACATAAGAGAACTTCAAGATCGCGTGAAGGAACTCGAGGCATTATCAGACCTTATGAGAAAAGATACTAAAGATATTCTAGTTGCTTTAAAGAGATATAGGCTTAGTAGGGATGAGGAAGACGATTCATCTCTGAATGAAACAAACTCTGGAGATCATAGTGCGGGTGTCCCTTCTGAATCATCTGCTGAAATTGAAGTGCGGATATCAGGAGGCAGCGTGCTAGTAAGAATCTATTCTCATAAAACCTATTCATTGGCTGTGAAAGTGCTCAGCCAGATGCAGAGTCTTGGGATTAACATCATCAGTAGCAGCACGATGCCTTTTGCTAATACTATCACTGTTATTACCATTGTTGCACAGATGGACAAGGCATCTGTGTTGGAAGATGCAACAAACTACATAAGAGAACTTCAAGATCGCGTGAAGGAACTCGAGGCATTATCAGACCTTATGAGAAAAGATACTAAAGATATTCTAGTTGCTTTAAAGAGATATAGGCTTAGTAGGGATGAGGAAGACGATTCATCTCTGAATGAAACAAACTCTGGAGATCATAGTGCGGGTGTCCCTTCTGAATCATCTGCTGAAATTGAAGTGCGGATATCAGGAGGCAGCGTGCTAGTAAGAATCTATTCTCATAAAACCTATTCATTGGCTGTGAAAGTGCTCAGCCAGATGCAGAGTCTTGGGATTAACATCATCAGTAGCAGCACGATGCCTTTTGCTAATACTATCACTGTTATTACCATTGTTGCACAGATTGAGGAAGATTTCGTTATGACAGCAGCAGATCTTGTGAGTAAGCTTCAACTAGCTTAG

AabHLH19

ATGAGTCAGTGTGTACCGAGCTGGGATGTTGATCATGAGAATAACTCTCGCAATAATTCGAACCTTGCTCGAAACAAAGTCTCCTTACGTGCTCCTTCCGGTTCCATTTCTTCAACTCTTGATGTTCCAACACTAGATTATGAAGTAGCGGAATTAACATGGAAAAACGGACAACTAGCCATGCACGGTTTAGGACCACCGCGTGTGGTTAACAAACCCCATGCGAACACCGCTAATCTCACCAAATACACTTGGGACAAGCCACACGCGGCCGAGACTCTGGAGGCCATTGTCAACCAAGCCACCCTCCAACCAAAACAAAAATCTCATATCAATATTTACTCTGACGACCTTGTCCCATGGCTAGACCACCATCACAATTCTGGTGTCACAGCTGGTACTGTTAGTGCGTCCGGCACAGTGACAATGGACGCGCTGGTCCCTAGCTCTAATACACAACCCCACGCGCTATCTGGTACAAATGGTGCCCCAACAAATTGTTCTACACGCGTGGGGTCATGTAATGGTGACCAGTCTTGGTATGGGGACCACATGACGGCCCAAGGTGGCGCAGCCACACATGAGTGGAGCAGTTGTAGGGACCACAGTGGGAGTGGCAGTGCTACTTTTGGAATGGAAAGTAGCCGACAGCTGACTGTTGAAACATGTGAGAGGGAATTGGGTCCGAAAGGATTCACCTCAACTTCCACGGGCTCTCCAGAAAATACTGTTTCCGGTAAACAACAGTCCACTAAATCGACATCTCCTGATGAACATGATTCTGTTTGTCATAGTAGACCTCAGAAGAATGAAGTGGATGAAAAGAAGAAAGGTAAAGGAAAATCATCGATCTCGACTAAGAGAAGTAGAGCGGCTGCAGTCCACAATCAATCTGAGCGGAAACGAAGAGACAAGATCAATCAGAAGATGAAAACGTTACAAAAACTCGTTCCAAATGCTAATAAGACAGACAAAGCCTCGATGCTTGATGAAGTGATCGAATACCTCAAACAATTACAAGGACAAATTAATATGATTAATAGAATGAACATGTCTCCTATGATGATGCCATTAGCTATGCAACAACAACAATTTCAAATGGCCATGATGAACCCCATGGGTTTGGGAATGGGTATGGGGATGGGAATGGGGATGCCAGGAGTCATGGACTTGAATTCCATCAGCGCCAATCGCCCCAACATCCATGGGATGCCGCCAGTCTTCCACCCCTCCAACTTTATGCAGCCGACGATGGCTTCATGGGATATGAACACCACAAGCGATCAAGTTCCGAACCATAATGATCAAATGGCTGCATTTCTTGCATGCCAATCACAGCCCATGACGATGGAAGGTTATAGTCGAATGGCTGCAATGTTTCAGCAGATGCAAAATCAACCTACCTACCCTGGTCTCAAGAATTGA

AabHLH20

ATGCCACTTTCAGAGCTGTATAAGAACGAATCGTCTCAGCAGAAATTGACCGATATATCGTATATACCGAATGACGAGTTTGTGGAGTTGATATGGGAAAAGGGTCAGGTTATGATGCAGGGTCAGTCTAGTAAAGCTAGAAAGACGCCGGTTTCTAGTAACTTTCAGTTTCATGCGCCAAAAGTTCAAGGAAAAGATGGTATGTTGAATGTACCTATGGCGGAAATAGGATTGGATCAAGATGATGATATGGTGCCATGGTTGAATTATCCGCTTGATGATTATTGTGCTGATTTGTTACCCGAGATATCTGGTGTGACGGTTCATGAGCCGACTATGCATAATGGTATGAGTGTTATTGATAAGAGAGGTAATAAGGATACTAGTGTGTTTAATGGTTTAGACCAAGCGAACACATCTAAGGGTTTAAAAGTTAGCAACTTGTTTTCTTGGCCAGATCCGATGGTCAGATCAGGAATTACGGATATAGGTAGTAGTAATTGTAGAAGTAAAGTCGATAATGTGGTTCATAGAGATCCAATACAGATTCAAGGTTCAGCAGGCAGGGTTGAGAAAATTGCGCAAAAACAAGATTCATCATCTACCTTGTTGAACTTTTCTCATTTTTCAAGACCTGCTGCTATGGCTAAAACTAATCTTCAGAACACTGCAGTTAATGTTGTGAATCATGGATCACAGAAAGAAATTGGGTTCGTTAGTCAACCGAATTTGGATTCAGTTGGAGTAGGTTCGAACCCTTTTGTGAGTAAGCCACTTAATGAGCCACATTCTGTTGAAAAGTCATCAAATGTGGTTCTTGATGTGAATGGAGCGAAAGGAGTTCAAGAAACTGTAAAGAGCAATGAGCCAGTTGTTGCTACTTCTTCTGTTTGCTCAGGGAATAGTGCTGAGAGAGCTTCTAATGACTTTTCCAAGAATTCTAAGAGGAAATCTCGTGATACGGAGGAGTTTGAGTGCCAAAGCCAAGATGTTGATGAAGAGTCGCTGGGTACTAAAACAGCCAGCGCGTCTCGTGGAGGTACAGGTTCTAAAAGAAGCCGGGCTGCTGAAGTTCATAATTTGTCTGAAAGGAGGCGAAGGGATAGGATAAATGAAAAGATGCGTGCACTACAAGAACTCATTCCAAATTGCAACAAGGTGGATAAAGCATCAATGCTTGACGAGGCAATCGAGTATTTGAAGACACTTCAACTTCAAGTTCAGATTATGTCAATGGGAAGTGGATTATGTATGTCCCCAATGATGTTCCCAGGTGGAATGCATCCTCATTTCTCCCCCATGGGAATTGGCATGGGAATGGGCTATGGAATCGGAATGGGAATGGAGATGAATCAGCATGGACACCCAAATATGTTTCAATTTCCTCCAAATGCACAAGGGTCACGGCACCCTCTCCCTTCCCCAACTGTTTATGGGCATCCTAGCCAAGGAATGCCAATGTTATTTCCACAACCGCCTATGCCCAGGGTTCCTATGCATCCTGCTGCTCGACCGGTAGATGTAGCTCCAAGCTCAAAGGACCCGATGCAAACTAAGAACTCTCATGCAATGACCCAATTATCTAATCAGTCCCAAGATGCACACAAGACGCTTAACCAGTCTACTTTGGTTGACAAAAAAGATCAAGGTTTAGAAGCCGGATGTAGTACAGCTGGTATTGATTGA

AabHLH21

ATGCAACATTTTGAACATGAAAACTCCACTTCTTCTGGCATAGCCGCAAATGGTAGTACTGGAGATGATACTGTAGTGGCAAAAAAGATCAATCATAATGCAGGTGAAAGAGATCGCCGAAAGCGCGTTAATGACTTATATTCGTATCTTCGTTCACTGCTACCCATATCAAGTTATCACAAGAAAAAAGTAAGCATTCCCGAAACTGTATCGAGTGCAGTGAAATATATACCCGAACTACAAAAGGAAGTGGAGAGACTAAAGCATAAAAAGGAAAAAGTTCAGTGGTCTTCATCACCGACCATTAATGCCAAGCAAGAGCATCTTGCCATCAAGAAGAAAAGTTGCAAAACAAAAACAAATTCATTTTTAGTTTCTTCAGTGAATGTTTTGGGTGATAAAGAGGTTGTTATCCAGTTGATTTCCTCAACTGATCATACGAGCACGAACAAAGAGATAGGCTTCTTGTCGAAGGTTCTGGAAAACTTAGAACACGATGAAGATGGATTTGTATTGCTGAATGCGACGACCATGAAATGTTCCGGAGAAGGGATGGTTTTAAACACTCTTCATCTCCAGGTACAAGGGGATCACAAGATAGGCGGTGAAAAGTTGAAGGAACAAATGTGCTCTTTCTACCAAAATATATATGGAACTTTACTCTAA

AabHLH22

ATGGAAGACATTGGTGATGAATACAAGCACTACTGGGAAACCAATATGTTTCTCCAAAACGAAGAATTCGATAGTTGGGGAGGATTAGAAGAAACATTTTCAGGATACTATGACTCGAGCTCGCCCGATGGAGCGCAGTCGTCGGCAGCCTCAAAAAACATTATGTCCGAACGGAATAGGAGAAAGAAGCTCAATGATAGGCTGTTTGCACTTAGAGCCGTGGTCCCCAACATTAGTAAGATGGATAAAGCGTCTATAATTAAAGACGCAATTGATTATATACAACATTTGCATGATCAAGAGAGGGTTATTCAAGCGGAAATAATGGAACTCGAGTCGCGAAAATTGGAATCCGGGGTGCTTGAATATGATCAAGAGATGGCGTTTATGTCGACGGAAAATTCTAAGAAGAAAAAGATTGAACAATCTTTTGATTCTAGTGAGTCGAGGGCATATCCTGTCGAAATACTTGAGTTAAAAGTGTCGTATGTTGGTGAAAAGACGGTTTTGGTGAGCTTGAAGTGTAGGAAAAGCAGAGACACAATGGTTAAGATTTGTGAGGTTTTCGAAACATTGAAACTCAATGTTGTAACGGCTAACGTTACTACTTTTTCTGATACCCTTTTCAAGACACTCTTCATTCAGGCTGATGAGGAAGAAATTGATCTCCTGAAGATACAAATCCATACTGCCATATCAGCTCTAAATGACCCTCCGAGTCCAATGAGCACCTAG

AabHLH23

ATGCATCCTCATCATCATAATCACCAATCTTCATGGACAACTCTATCTCAACCCTTCCAACAATACACCTTCAATCATGTTCTTCCTCAATCATCACTACCTAATGGTGGTTTGTTTAAAAGGGTTGTTGATGGTTTGCAGTTTGCCTATGAGGGTAGTAGTAGTAGTACTACATCTCCATCGGCTGACCATTATCTAGGGTTTCAACCTGGATCCAATACCCTTGGGATCCAAACTGATACGACTAAGATGACAACTCAAGAGATGGCGGATGCTAAAGCCATAGCTGCCTCGAAAAGCCACAGTGAAGCAGAGCGAAGACGCCGAGAACGAATCAATAATCATCTTGCGAAGCTTCGTAGCTTACTTCCCAACACCACCAAAACAGACAAGGCTTCATTACTTGCTGAAGTGATACAACACGTGAAGGAGCTCAAGCATCAAACTTCCATTATAGCGGCACAAATTCCAGTCCCTAGTGAGATAGATGAGCTGGTCATTGATAACACATCGGATGAAGAAGGAAGAATTGTAATCAAAGCATCGTTATGTTGTGATGACCGGTCAGATCTCTTGCCTGACCTAATCAAGACTTTGAAATTGCTTCAATTGCGTACCCTAAAAGCCGAGATCACAACAATTGGAAGCCGTGTGAAGAACGTTTTATTTTTAACAGGAGAAGATCATATGAATAGCAACGAGGATGAAAAGGTTGTAAATCACTGGGTATACTCTATTACAGAGGCGATTAAGGCAGTAGTTGAGAAAACAAATGACGGAAATGATTGTTCTTTTGCAAGTATTAAGAGGCAAAGAACAAATAACATTGATATATTTAATCATCGTAGGCGTCTTTAG

AabHLH24

ATGTCACCGGCATGGCTATCAGAAATGGAAATAGAAGATCCAGGGTTCATGAATTATGATCAGATGAGCAGACTTTGTGATACAAGCCTTGATTCATTCTCCCCAGAAAGCTACGCCGAAAACATGACGTTTATCGATCAATCTTTCCAGGCTCCACAGCTCGAGATCGAAATTCCTAACTATCAAGAAAAGAGTAGTAGCATTGATAAATGTTCTCCAAGTCCAGATACCCTTGTTGCTACTACTCTTCCCTCATCCAATACTTTTACTATATCTTTTGGAGATCTAAAACCTAAAAGCGAGATCCTCCAATTTTCTGATCCGCTTCCTGGTGCCATAAAGGTTCCAACCATCCTCAGGAATCCACTTCAGGCACAAGATCATGTTTTGGCAGAGAGGAAGAGAAGAGAAAAGTTGAACCGACACTTCATTTCTTTGTCTGCCATCATTCCTAACCTAACGAAGATGGATAAGGCATCTGTGTTGGAAGATGCAACTAAATATATAAAAGAACTTCAAGATCGGGTCAAGGAGCTCGAGGGATCTCCAAGTACTAAGAGAAAACATGTACAAGAGTCTGTTATTTATGTAAAGAGATCTAGGCGTAGTGCTAGTGATGATCAATATTATTCGTCGGATGACACAAACTCTGAAGAGAGCACTGCACCTTACAAGACATCTCCTGAAATCGAAGTGCGGATGTCAGGAAGCAGTGTTCTAGTGTCTATTAAGTGCCATGATAACATTTCTTCATTGACAAAAGCACTAGATCATATGCAGAAACTTGGGTTGTCCATCATCAGTTGCAGTTCCATGCCCTTTGCTAAGACCACCCTTCTTATCGGTATAACTGCTCAGATTGAGGATGACTTCTGTATGACAACAACAGAACTTGTAAAAAACCTTCAACTAGCTTTATGA

AabHLH25

ATGTGTTCTAAGAGAGAAGAACAAGAACTAGTAGAAGAACAACAACTACAACAAGAAGGTGATCATCAAGAACATCCATCACTTATTATTCCACAAACTCAACATCAAAATATGGGTATTGTTGATTATTCATACACATCATCATCAGAAGTCTCACCTATCTTGCACCCACAACAACAACCATGGATCATACCTCAAGTATTTAACCATCACAATACCGCTTGCACTGATCTTCCATTTACTGACCACGGAAACTATATATTCCCTCCACCACCTCCTCCATCATCACTAACTTCGTCCTATGGTGGTTTGGTTAATAGGAGAGTCCCTAGTGGCTTGCAGTTTGCATATGATGGTAGTACATCCTCATCAGACCATCATTTGAGGCTCATATCCGAGACACTCGGCCAAATGGTTCAGCCAGGGTCTATGCCGTTTGGGCTACAAGCAGAAATGGGCAAAATGACTGCTCAAGAGATCATGGATGCTAAAGCTTTAGCTGCTTCAAAGAGCCATAGCGAAGCAGAAAGACGACGTAGAGAGCGAATCAACAATCATCTAGCTAAACTCCGTAGCATACTTCCTAGCACCACCAAAACGGACAAAGCTTCGTTGCTAGCTGAAGTAATACAACACTTGAAAGAGCTCAAGCGTCAAACTTCAATAATAGCTGAACAAAGTCCGGTCCCTACAGAGACTGACGAGTTGATCATTGACAACACGTCGGATGAAGATGGAAATTTAGTCATCCGAGCATCGTTGTGTTGTGAGGACCGCTCAGATCTTTTACCAGACCTCATCAAAACCCTAAAAGCACTTCACCTAAGAACCCTAAAAGCCGAGATCACAACACTTGGAGGACGTGTGAAGAATGTATTGTTCATTACAGGAGATCAAGATCCGAACAATAACGATAGTAATACAAATTACTCGATAAACATGATTCAAGAAGCATTTAAAGCAGTAATGGAGAAAACAAATGGGGATCATGAATCAACTTCTGGGAGTTCTAAGAGACAAAGAACAAACAGCATTCATGTTGTTGATCATCGTCGGACATGA

AabHLH26

ATGCATCAACAGCAGCCTACCCCTCCTCTTGTCCCCATGCTTGCTGACTATGGAGTAGCAGAAATAACATGGGAAAATGGCCAACCAGCCATGCATGGGCTAGGAAGAGCAAATGAGACACTAGAATCAATTGTTCATCAAGCTACAACATGTTACAACCAAACTCAATATCCAGAAATCGACTTACAACAAAGTCAAAGTCTTCCAAGAGCTCGCAACTTAAGCTCGAATGTTGCATCATCGAGTCGCCCGACTTACCTAAGGAAACGGCCTAGAGAATCTGTCATTATCCATGATCAATGTGTAGGAAATTTGGGTAACGCAAGTTTGCAAGAAGATAATGTTAGCAATAGTGGGACGGTTAATTCTAAAGATAATGATACTACAATGATGACATGGCCTTCATTCGACTCGCCTAATCAAAGCATGAAGAGCCAAAAAACAGATGATGATTCTGCCTGCCAATATGGATCGGAAAATCAAGAAGAAGAATGTAGGACTGAGGGTGAAACAATTCGATCTCAATCAAGTCGACGAAGCAGAGCAGCTGCTATTCATAACCAGTCCGAACGGCGACGAAGAGAAAGGATCAACCAGAAAATGAAAGCTCTACAGAAGCTTGTGCCTAATGCTAATAAGACGGATAAAGCATCGCTGTTGGATGAAGTGATTGATTACTTAAAAAAGCTACAATCACAAGTACAATTGATGAAGAACATGCCGTTTACACCACAACAAATGATGATGTCAATGCCCCTACAATTGCAGCAACAACAGCATCAACATCAGCAGCAGCAGCAGCTTCAAATGTCGATGCTAGCGCGAATGGGAATGGGATTCGGCCTTCAGATGGGAATGCCTGGAGTTATACCTCAGCCAGTTCATAATCCATTCATGGTCCCACAAACCATGCTTAGCCCAGCCCATGTAGGCACCACGTCACAAACTATTCATAGTCGCCCATCTACCAACACCCCAGTTCCTTTCAACGATCCACACAGCACGTTTCTAGCACAACAAATGAACATGGATATGTACAACAACATGGCAGCTTTCTATCGGCAACAGGTCAACCAGGGAAAATCGATGAGCGTTGACTCATCTCAACTAGACCATGTTCGGGGAGAGTGA

AabHLH27

ATGCTAGTTGATCCTGAAATGTATGAAGGAAGTGCTTGCTATGATCCAACTCATCTCGAATCGCTTATCGACCATCAAGATGACAACAATATGTCCCAAGCACATCTCCATAACTACCATCAAGCTCAAAATTTTAACTCACTTGATCAACAACAAGATTATCATATGATCAACATGGAAATGGAACACCAAAACCAAATCATGCAAGACCACCTTAATTGGTCCACTACTCATTCCCATGAACAAATACATATAGAAAACAATAATACTAGTAGCATACCAAACATGCCTTTAATCACACCTAATCCAACCCCACCTGATCTTTTGAACTTGTTTCAATTACCAAGATGCTCGAACTCATCTATATCGTTTTCTAACCCTACCCATATGGACCAAACTTCGTATGATCCTTTGTTGCCTTTAAATTTACCTCCACAACCGCCTTTTTTTCGCGAGTTGCTTCATTCGCTCCCTAACGGGTATAATTTAACGGGTGGTGGGTCGATTTTCGGAGAAATGGACATGGAAAGAGATCACCAATTGTATCATCATGAAGGTAATGGAATTTTGAAGTTTAGTGGTGCTGATATTAGTGGGATTGTTGGAAAAGGTAGAGATGTTAAGGATACTAAACATTTTGCTACTGAAAAACATAGGAGGCAACAATTGAATGATAAATTTGATGCTTTGAAGAATTTGGTTCCTAATCCTACCAAGGCGGATCGAGCATCAGTGGTAGGAGATGCAATTGACTACATAAATGAGCTCAAAAGAACCGTCGAGGAACTCACAATTCTTGTGGAGAGGAAAAGATGCAGCAGAGGAAGGATGAAAAAGCACAAAACCGAAGATGACTCGACACTAGATGTTGAAAGCATCAACACTAGGCCGAATGGTGGTGGTGATCAAGACCAACAAGGTTATAATGGTAATTCCACTTCTACGTTGAGGAGTTCATGGCTTCAACGAAAATCAAAGAACACTGAGGTCGATGTTCGTATCATTGATGATGAAGTTACCATCAAATTTGTCCAACAAAAGAGAATCAATTGTTTGTTGTTTGTTTCCAAAGTTCTTGATGAACTTCAATTGGATCTTAACCATGTTGCTGGTGGACTAATTGGTGACTTCTATAGTTACCTTTTCAACACCAAGATTTGTGAAGGGTCATCAGTTTATGCAAGTGCTATAGCAAATAAGCTAATTGAGGTTGTCGACAGGCACTATGCAAGTATCCCAACACCTAGTGGCTACTAG

AabHLH28

ATGGAAGAACAGGCTAGGAAGAAGAAGAATGATAGTAATAAGATGCTGAATACATATGTTTTAGAGAAGGATAACAGTCTTAATGTGGGGGGTAAGGATAGAATGGAGGGTCATGTTGTGAGTAATGATGTTAATAAAACGGATAGTGTGAATGGTACTAATGGTAAGGTGGATAATGATCTAGCTACTGGTTCAGATGTGAATAAGAGTAAGGTTGCTGTTGTTGACAAGAGCAAGGAGCAAGGTAATATGGAGAAGTGTGAATCAGTTGATGAACATGATGAGATTAAGAAAGATGATAGGAAGACTTATGCTAGTGCTACTTATGATACTAAACTAGATATGTCTAGGAAGTTGTTTGAAGTTCCTACTGAGGTGGATGAGAATGGATATGAGTTGGGAGTTGGTAGAGTGGGTTATGCTAGGGTGATGGTTGAAGTATCAGCAAAGAAATGTTTGCCTGATATTATAGAAATGATTTACAGGAATAAGAATGGAGGTGAGATATGTAGGAAAACAGTGAATGTGGTATATGATTGGACTCCTCCTAGATGCTCACATTGTTGTGTATTTGGTCATAGTGATAAGATGTGCAAGGTTTGTGAGAGTAATGAGGAGCCAAAAGATGCTAATAATACAGTAGAAGTGTCTGCTGAAAAAGAGAACAAGTCAGAGGAAGGAAAAGAGAATGTTGAGAAGAAGAATGATGGTTTTGAAGAGGTTAGATATAAAAAGAACAATGGTGGTAATAAAGGAAAAGGGCATAACAATAATAATGGTCAGAGGAAAAATGATGGTAAGCAGAATGTGCAACAAAATCAGGGTGTATATCAGAAGAAGGTGAATAAGGAGCAAGGTGAATCTAGTAATAGTAATAAAACAACAAAGTCTCCTGTGAAATCTCCAGTGAAAGCACCACTTAATAATAACAATGTCCCGGGTACACCTAATAGTAGGAAGGCTTGGAAAGTTCAGGGTGAGATTCTGGAAGAATTAAAGAGGTCTGCTAATAAGTTTGCTGGATTGGAAGTACCTGATGATACTGGGTGTTTGGGAAACAATGAGTGTTCTATGGATCTGAAAATTGGATGTTGGAACATCAGGGGTTTAAGTACCACTGATAAACAAAATGAGGTTAGAAAGTATATTGATGATGAAAGGTTGCATATGTGTGGTATTATAGAAACTCAGTTAAAGACAAAAAAGTTACAAAAAATTGGAGATTCTGTCTTCAAAAATTGGTCTTGGGTTAATAATATGAGGATGTGTGATAAGGGATGCAGAATCATGTTAGGATGGAATAGTGATATTGTCAATGTAAATGTCATTCATTACAGCAAGCAGTCTATTTTGTGCAAGGTTGAAATTGTAACTGGGAATATGGCTCTGTTTTGTACAATCATTTATGCTGCTAATAGTGGTAATGAGAGGAAGGATCTATGTTTGCTGGTTCTGTTTGATAGTATGAATGCACAAGGGTCTGTTATGGATGCTGACTTTTGCAGTCCTGAAAGTATTTTGTCATCCAGTGGAATATGTTTTGGATGTGCAGAGTTTCTCATCCCACAACCACCACCTCCTCCACCTTTATCATGCTATGGTGGCTTGTTTAACCGAAGGTTACCTTTGCAATTTGCATATGAAGGTAATCCCTCAGCTGATCATCACTTAAGACTTTTGTCAGAGACACTTGGGCATGTGGTTCAACCTGGATCCGGACCGTTTGGGCTCCAAGCTGAAATGAGTAAGATGACTGCTCAAGAGATCATGGATGCTAAAGCTTTAGCAGCTTCGAAAAGCCATAGTGAAGCTGAAAGACGTCGTAGGGAACGAATCAACAATCATCTAGCCAAACTTCGAAGCTTACTCCCTAGCACAACCAAAACTGACAAGGCTTCATTGCTAGCCGAAGTAATACAACATGTTAAAGAGTTAAAGCGTCAAACTTCGATAATAGCTGAACAATGTCCAGTCCCCACAGAGACTGACGAGTTAACTGTGGACAACGCGTCTGACAAAGATGGTAAACTTCTGATCAAAGCTTCATTATGTTGTGAGGACCGTTCAGACCTCTTGCCGGACCTCATCAAGACATTAAAGGCACTTCGACTAAGAACCCTAAAAGCTGAGATCACAACCCTTGGTGGACGTGTAAAGAATGTTTTGTTTATCACGGCAGACGAAGATCATTTAAATGGAAATGATGATCAGCAAATGGTAAATTACTCGATAAACACAATCCAAGAAGCACTTAAACAAGTGATGGAGAAAACAAATGGTGATGATTCAGGGAGTGTAAAGAGACAAAGAACAAACAACATTAACATTTTGGAGCATCATAGGTCTCTTTAA

AabHLH29

ATGGATTTCCCTCCAAATTGTTTCAAAGGATTCACTTCATCAGAAGAACATGTGCTAAAGGAAATGATGATGATGAGTCGTGTTAGAACGACTTCGTCATCATCTTCCTTAGTATTAGACAATGAAAAAGGTGAAATAGTGAGAGCTTTAGTGACACCGGGCAATATTCGCCATACTTTTCATGATGATCATGATCATGCAAATCTTCAAAAGGGTGCGAAAGGTGAGAAAGCTTTAATGGCGTTGAGAAATCACAGCGAGGCGGAAAGAAGAAGGAGGGAGAGGATTAATGGACATCTTTCTATGCTTCGTAGTCTCGTCCCGGGCACTACTAAGATGGACAAAGCCTCATTACTTGCTGAAGTTATCTCCCATTTGAAGCATATGAGGATGACCACCACTGAAGCCACTAAAGGTGTTCTTATACCAATGGATATAGATGAAGTAAAAGTTGAACAACAAGATGAAAACAGTTTGGATGGATCCTCATATTCCATAAGAGCATCTCTATGTTGTGAGTACAAACATGAGGTTTTATTAGATTTAAAAGAAGCTCTTGATGGCCTCCAATTAAAAACAATTCGTGCAGAAATAGCGACCTTAGGAAGCAGGATGATGAACCTTTTTGTAATTACGGGTAGCAAAGATGAAGTAAATATCAAAGATATCGTGAGTTCTATACGCCAAGCACTCAAATCTGTACTTGATAAATTCTACGCTTCACAAGAGTTCTCAGAAAGTAATTCACTTTCAAACAAACGACGGAGGGTGTCTTTTTTTACACCCTCAAGCTCGTCTTCGTTAGGAGACTTCTGGTGA

AabHLH30

ATGGGCATTAAAGATGAGCAAAGTGATGAGTTGGTATTTCATCAGATTCCTTCCTTAATTTGCTTCCAACAACCGGCAAGAAATCAACAAGATCTTATAGTGGAGGATAAGGATCATGTTACGATGGAGGGGAATACAAATAAGTCGGCCAGCAAGACCCGAAAAAAGCAAGAAGATCAGTCGCCTTCCTCGTCTAAACCTAATTCGGCAACTCCGGAAGATAGTACTAAAGATGAGCATACACAAAGAAAATTGGTACATAGAGAGATCGAAAGACAGCGTAGGCAAGATATGGCTAAGCTCTATGCTTCACTTAGGGGTCTACTTCCACTCGAATTCGTCAAGGGAAAGCGGTCTACATCGGATCATATGCATCAGGCAGTGAACTATATCAAACACATGCAAGAAAACATCAAAGAAATGACTGTCAAGCGAGACCAGCTCAAGAAATTCGTGGAGATGAGTGTTAGTGGGCTTGGGACAAATTCTAATGAAAAAAACTTAACGAACCTGCTTCCTAACACAGTTTCACTCCACTCTTCTAATGGGGGAATTCAGATTTCCATCAATAGTTGTCCTTTCGAAGAGGGGTTCCCTCTTTCAAGAATACTAAAAGCGATTTCCAAAGAAGGTTTTAAGGTTATAACCTGCACATGTACTAAAGTAAATGATCGGTTGATTCATTCTATCCAAGCCGAGGCAAATGATCCCGTTTTGACTGATCTCTCCATGTTGCAACAAAGGTTAACTGTGGCAGCGAATAACTACTGA

AabHLH31

ATGGAATTATATGGTTGGACTAATCCAGGGTTCTCAAACTTGGGACCAAATGGATTTGATGGATCTTTGATGAACTCAAGTGCGTTTAACGAGGACTATGGAAATGTTGATGGTTTGTTCATGAAGTCTAGTGAATCTTTGGTTTTGGACAACGAAAAAAGTGAGCTTGTTAAGGGTCAAAGTAAAGTGATTGGAAAGAAAATAGGATCAATATCTGATGAGAAAGCTGTGGCTGCTTTGAAGAGTCATAGCGAGGCTGAGAGAAGGAGAAGAGAAAGGATTAATGCTCATCTTGATACTCTTCGAGGCCTTGTACCATGCAACAATAAGATGGACAAAGCCACATTACTCGCCGAAGTTATCCGCCAAATAAAACAACTGAAAGTAAATGCAACACAAGCTAGCACAGGGTTACTCATGCCAGAAGACGTCGATGAAGTAAAAATTGAAAAGCTTGACCAACTTTCAGTCAATGGAAGTATCATTTTTCACGCGTCTTTCGGTTGCAAGCACCGTCCCGAGCTCCTAACAGATGTAAGAAAAGCCCTAGTTGACCTCAAAGTCCACATGGAAAGGGCACAACTGTCATATCTAGGAGACCATGTTAAAATAATTTTTGATTTCATGGCGAATGAAGATCTAGTTACTTCTGTTCGTGAAGCTCTTACAGCTGTTATAGAAAAGGGGTCAATCTCACCCGAATACTCCCTACGAACACTGCCTAACAAGAGGAGGAGGTATTGTCTATAA

AabHLH32

ATGGTGAAGAGTTTGATCGACCACTTTCCGGTTCCTGGCGGTGAACTTCCTTCTCTTGAACCGGGTTTTCACTGGTCTGCTAACTCCTTTCCCGGTTCAACCAGCGTTGTTAGTAAAGGATTTTCTGATTCTTATGGACAATCGGATGCTACCAAGGAGGACGAGTCACGAAAGAGGGCTCATCCTGGATCGTGCTCTAGTACAAAAGCATGTCGAGAGAAGAAAAGGAGGGACAAACTAAACGAGAGGTTTCAAGAATTGAATGAAATACTGGACCCTGGAAGGTCAGCTAAGACGGATAAGACGGTTATTTTGGCGGATGCTATTCGAATGATCACACACTTGAGAAATGAAGCCACAAATCTTAAGGACTCATCTCAAGATTTGCTCGTTAAAATCAATGAGCTGAAGGTTGAGAAGAACGAGCTGAGAGACGAGAAGCAAAAGTTGAAGACAGACAAAGAGAGACTAGAACAACAGCTGAAATCCACCTTTTGTGGTCCTCCAACGGCATTCTATCCTCCTGCACATCCCGTAATGCCGGTCCCATGTCCAGGCCCCACCCCCGTTGGTGGAAACAAGTTCATGCCATACATGGGATTCCAAGGGGTTCCCATGTGGCAATTTGCTCCCCCAGCTGCGGTTGATACTTCCAAAGATCATGTTCATCGATCACCACTTGCATAG

AabHLH33

ATGCTAGCATTATCTCCTACATTGTTTTCCACAAATTATGAATGGCCCTTGGGGGATAACCTTGACAGAAATCACCAACAAGATTGCAATAATATTTCTATGGACGTTGAAGCAAATTCATATGATTCAGTTCTTGATTTTCCAACATATGATCAAATCCGGCAGGATTTTACACCCGAGAGTTCAAATTCATTTGGAGGAGCTATAAATGGAAATACTGGTAATCCCATGAAGGTGTCCAAAAAGCTTAATCATAATGCTAGTGAAAGAGATCGGCGTAAGAGGGTTAACGATTTATATGCATATCTTCGTTCATTGCTGCCCATATCAGCTGATCAAAAGAAAAAAGTAAGTATTCCAGGGACAGTATCACGGGCACTCGAATACATACCAGAACCACAAAAGGAAGTAGAGACATTAATACGTAAAAAGGAAAAACTTTCATCGTACTCAACATCATCCGCTAGTACAAGCCAAAAGAATATCGGCATCGAGGGACAAAGTTCTAAAGATGTTACAGCTAATACAAATTCATCGGTAGTTTCATCTGTGAGCATTTTAGGTGAGAAAGAAGTGGTGATACAGCTGATTTTCTCTATGGATCGTATGAACAAGAATAAGGAGATTGGCTCCTTGTCTAGGGTTTTGGCATACCTAGAGTCGGAAGAAAATGGATTTGTTTTGCTAAATTCAACAACCTTCAAATGTTCGGGAGAACATATGTTATTAAACACTCTGCATCTTCAGGTGCAAGGAGATAAGAAAGTGGACGTTGAAATGTTGAAGGAGAAACTCTGCCATTTCAGCCAATAA

AabHLH34

ATGGAAAACATCACTGATGTCAACTGGGAATCCATCCTTCAACAAGAAGATTATAGTTGGGGTGGATTAGAGGAGACTTTTTCGGGCTACTATGATTCGAGTTCTCCAGACGGAGGACAGTCATCGTCGCCAGCATCGAAGAACATAGTTTCTGAGAGGAATAGAAGAAAGAAGCTGAATGATAGGTTGTTTGCACTTCGAGCAGTTGTTCCTAATATTAGCAAGATGGATAAGGCGTCAATAATTAAAGACGCGATCGAGTATATCCAATTATTGCACGATCAAGAGAGAACAATCCAAGCAGAACTAATGGAACTCGAGTCTCAAAAGCTAGAGCCCGAGAATTTCGATTTCAATCAAGAGACAAAGTCGTCTATGTCAATGGAAAAATCAAAGAAGATAAGAGTTGAGCAAGCTTTTGATTCGAGTGGATCAAGCTCGTATCCCATTGAAGTTCAAGATTTACATGTGTCATATATGGGAGAGAAGACAGTGTTGGTGAACCTAACATGTAACAAAAGAAGAGATACAATTGTGAAGCTTTGTGAGGTTTTTGAATCTTTGAAGCTCAAGATTGTCACAGCCAACATCTATGCTTTTTCAGAAAGGCTTTCAAACACTCTCTTTATTCAGGCTGACGAGGAAGATACAGACGTGTTGAAGATACAATTAGAAACAGCCATATCGACCTTAAATGACCCTCATAGTCCAATGAGAATCAAGAAGGGAGCAAAAGGAAGGCTTGTAATAATTTCAAGTAGGAATTATAGTTGGTGTAATTGA

AabHLH35

ATGCTCAGGAATCCACTTCAGGCACAAGATCATGTTTTGGCAGAGAGGAAGAGAAGAGAAAAGTTGAACCGACACTTCATTTCTTTGTCTGCCATCATTCCTAACCTAACGAAGATGGATAAGGCATCTGTGTTGGAAGATGCATCTAAATATATAAAAGAACTTCAAGATCGGGTCAAGGAGCTCGAGGAATCTCCAAGTATTAAGAGAAACCATGTACACGAATCTGTTATATCTGTAAAGAGATCTAGGCTTAGTGCTAGTGATGATGAATATTATTCGTCGAATGACACAAACTCTGAAGAGAGCACTGCACCTTACATGACATCTCCTGAAATCGAAGTGCGGATGTCAGGAAGCAGTGTTCTAGTGTCAATTAAGTGCCAGAATAACATTTCTTCTTTCACAAAAGCACTAGACCATATGGAGAAACTTGGGTTGTCCATCATCAGTTGCAGTTCCATGCCCTTTGCTAAGACCAACCTTCTTATCGGCATAACTGCTCAGGTGGGTGGCCAGGTCCGCCCGCCCCGGGGGCGGCGGCCCTGCCGGCCCGCCGGGGGCGCGGGCCGGCCGCGCGGCCGCGGGCGCCGATGCGCGGCGGGCGGCGGCGCCCCCGCCGCCGCGGTGACCCGCCGCCGCGCCCGCCGCCCCTACTTATTATCTTTTCGAGATTCTAAACCTAAAAGCCGAGATCCCTCCAGTTTCCTGGATCTGGCTTGCTTGGCTGCCATAAAGGGTTCAACCCATGCTCAGGAATCCACTTCAGGCACAAGGATCATGTTTTTGGGCAGGAGAGGAAGGAGAAGAGAAAAAGTTGAGCCATTTCTTGGCTCTGCCATCATTCCTAACCTAACGAAGATTGAGGATGACTTCTGTATGACAACAACAGAACTTGTAAAAAGCCTTCAACTAGCTTTATGA

AabHLH36

ATGCACTTTTCTAATGCAAGAGGATCACCTAGTTACCAATGTATAAATAGACCAACACATCGTCCTCATAATTTTCATTTAACCTTTCAACAACTATTCCCTCTAATAAATAAAAAACAAAAAATGCTAGCAGTACCCAGTACATTATTTTCCACATCTTATGGATGGCCCTTGGAGGATAACATCGCCCCAAATCACCAACAAGATTGCAATGATGTTTCTATCGACATTGAAGCAAACTCGTATATCTCCCTTCTTGATTTTCCATTATATGATCAAAGCCAGCATGATTGTGCACCCGAGAGTTGTTCTTCTGGAGGAGCTATAAATGGAAATATTGGTGATCCTAAGAAGGTGTCCAAAAAGCTTAATCATAACGCAAGTGAAAGAGATCGGCGTAAGAGGGTTAATGATTTATATTCATATCTTCGTTCATTGCTGCCCATATCAGCCGATCAAAAGAAAAAAGTAAGTATTCCAGGGACGGTATCGCGTGCACTAAAATACATACCTGAACTACGAAAGGAAGTAGAGACATTAATGCGTAAAAAGGAAAATCTTTCATCGTACTCAACATCCACTATTGCAAGACAAAAGAATCTCGACATTGGGAGACAAAGTACTAAAGATGCTATAATTAATACAAATTCATCAGTAGTTTCTTCTGTGAGCGTTTTAAGTGAAAAAGAAGTGGTGATACAACTGAGTTACTCTACTGATCATATGAGCAATAATAAGGAGATTGGCTCCTTGTCTAGGGTTTTAGAATACTTAGAGTCCGAAGAAAACGGATTTGTTTTGCTAAATTTAACGACCTTTAAATGCCCGGGTGAAGAGACGTTACTGAACACTCTACATCTTCAGGTCAAAGGTGATTATAAAATTGAGGCTGAAAAGATGAAGGAGAATCTATGCTCTCTCTACCAACAATCATATGCCTAA

AabHLH37

ATGCAAGATTTTGCAACTGAAAACTCTATGTCTTCTGGCGTAGATGCAAATGGTGGTACCGGAGACGACACAAAGGTAGCAAAAAAGCTCAATCATAATGCAGGTGAAAGAGATCGTCGCAAGCGGGCTAATGATTTGTATTCATATCTCCGTTCACTGCTACCCATATCAAGTGATCAAAAAAAAAAAGTAAGCATTCCTGGGATAGTATCGCATGCACTGAAATATATACCTGAGCTACAAAAGGAAGTGGTGACATTAAAGCGTAAAAAAGAGAAGGCTCAGTCATCTTCATCACAAACCATGAATAGCAGCTGGCAAGAGCATCGTGCCATTAAGAAGGAAAATTGTAATGGTACTACAACAAATAGAGATTCTTGGTTAGTTTCTTCGGTGAATGTTTTAAGTGACAGAGAGGTTGTCATCCAGCTGATTTCCTCGACTGATCATATGGGCACAAATAAGAAGAATGGTTTTTTGTCTAAGGTTCTGGACAACTTAGAGCGTGACGAAAATGAATTTGTTTTGCTAAATGCGACAACAATGAAATGTTCTGGAGAAGGGATGGTACTAAACACTCTTCATCTTCAGGTGCAAGGGGATCACAAGATAGCGAGCGAAAAGTTGAAGGAACATATGTACTCTTTCTATCAAAAAGTATATGAGACTTTACTCTAA

AabHLH38

ATGGATATGCCGTCAGCTTGGCTACCAGAACTGGAAATGCAGGAACAAGGGTTCATAAATCAGTATCAGATGAACAAACCTTATCATCCTCTAATGGATGACTTCAGTGTTGATTCGTTTTCCTCAGAAAGCTACACAGAAAACCCATCTTTTATCGATCAATCTTTTCAAACTCGTAAGGGAGTTGAAGAACAAGCTGATATTAAGCAGCCGTCTAGCTACAAAAAGGCCAATAGTATCAACAATAAATTTACTCCAATCAATCAAATACCGAAACCAAAGCGTGTTTCTGATCCTCCTAACACTTTCACTATATCTTTTGGAGATATAAAACCAAAAGATGAAATACTTTCTTTTAGTGATTCATATGACCTCACAGGAGATGGTGCCAACAAGGTCCCTGCGATGATTAGGAATCCAATTCAGGTTCAAGATCACGTGCTAGCCGAGAGGAAGAGAAGAGAAAAGTTGGCTCAGCGGTTTATTTCTTTGTCTTCTCTCCTTCCAGACCTGAAGAAGATGGATAAGGCAACTGTATTAGAAGATGCAGCTAATTACATTCAAGAACTTCAAGGTCGAGTGAAGGAACTCGAGGAATTATCAGGCTTGAAGCGAAAGAACATGCAAGAATCAGTTATATCTGCAAAGAGATCAAGGCTTAGTTGTAGTGACGATGATGGTTCTTCCTCTAACACAGCAAACTTGGAAGAGAGTAGCAGTCCAATAAATCCAGAAATTGAAGTTAAGATGTCTGGATCCTGTATGCTAATCGAAATCTATAGCCACAAAAACTGCACATCACTAATGAAAGTGTTGAGTGAGATGCAGAGGCTTGGTTTATCTGTTACCAGTAGCAGCACCATGCCGTTTGCTGATACCACTCTTCTTATCACCATTGTTGCTCAGAAGAGTGATGATTTCATTATGTCATCAACAGATCTTGTGAAGAACCTAAAACTAGTTATTTGA

AabHLH39

ATGGATATGTCACAGGCATGGTTGGCAGAAATGGAAATGGAAGATCCAGGGTTCATGAGTTATGATCAAATGAGCATACTTAGCGATGTTGTTAATAACTTCAGCGTTGACTCATTCTACTCAGAAATATATGCAGAAACGACTACGTGTGTTGATAAAACTTTTCAGACTCAGCAGCCCGAAATAAGACAAGAGACGAGTAGTAGTATTAGCAAAAGCTCTGCACCTCTGGATCCACTTGTAACCAATAGTCTTCCCTCATCCAAAACATTTACTATATCATTTGGAGATCTAGAACCGAAAGAGGAGACCCTCCAGTTTGATGACTCCCTCGGGTATGAAGATGCTCGTACCACAAAGGTGTCGATCACTCTCAGGAACCCGATACAGGCTCAGGATCATGTTTTTGCTGAGAGGAGGAGAAGAGAAAAGTTGAATCAAAACTTCATATCTTTGTCCACCGTCCTTCCTAGCCTTAAAAAGATGGATAAGGCGTCTATGTTAGAAGATGCTTTCAATTACATAAAAGAACTTCAAGGTCGTGTGAAGGAACTCGAAGGAACATTAAAACCGGACAACAAAAGAGAGAATGTTGATCAAGAATCTGATATTTCCTTGAAGAGATATAAGCTTAGTAATCCGTGCGATAAAACAAGATCGGAAAAGAGTACTTCCCCTTGCAACACATCTCCTGAGATCAAAGTGTCGATCTCAGGAAGCAGTGTGACAGTTACAATTCAGTGCCAGAATAACTCTTCTTCCTTTGTTAAAGCACTAACTCAGATGCAGAAGCTTGGACTATCTATCATCTACAGCAGTGCCATGCCTTTTGTTACGACCATTCTTCTTATCACCATTGTTGCTCAGATTGTGGATGACTTCTCAATGACACCAACAGAACTTGCAAAGAGCCTTCAACTAGCTATATGA

AabHLH40

ATGGCTTTAGAAACACTTTCTTCCAATGAACTCCTCAACTTCATAATCTATGACACCATTTCTGCATCCCCTTTCACCTTCAATGACTCATCTTCTCAAAACACCAACAACTCAAATACCTTCTTCTACAATCTCCATAATCAAAACCCTAACCCTAACCCTAATCCTCTATCTCAAGAACTTGAAGGTGCTGACATGGAAATTATGAGTTCTAATTCTTCTTTAGCTACAACCACAGAAAAGATGTCTCTGGCTGTTCAAGCTTATTGTGGAGGCAAAAACCCTGAAAAGAATTATTGTGATAACAACAACAACAATAAAAGTATTCATAATAATTTAGGGGTTCAAAAGAAGAAAAGAAGAAGGAGGCCTAGGGTTTGTAAGAACAAAGAAGAAGCTGAATCTCAAAGAATGACTCACATTGCTGTCGAAAGAAATCGTAGGAAACAGATGAATGAACATCTTGCCGTCTTACGGTCCCTCATGCCGGAATCTTACGTCCAAAGGGGTGACCAAGCTTCCATAGTTGGTGGTGCTATAGAATTTGTCAAGGAGCTTGAACATCTTTTGCAATCTCTTGAAGCTAAAAAATTTGTCATGACTCAACAACCACAAGAAGATGACGATAACGGAGGCCATGATAGTAATTTCACCAAATTATCATCAGCTGCCCCTCCATTTTCACAGTTTTTTTCATACCCGCAATACACTTGCTCTCAAATTCCCAACAAGTACACTTCTAAAAGCAAGGCAGCTATTGCGGACATTGAAGTTACTTTGATCGAAACGCATGCTAATCTTCGGATTTTATCGCAAAAACGGCTTGCTCAGCTGTCTAAAATGGTGGCTTGTTTCCAAACACTTTATTTATCAGTGCTACACCTCAATGTCACCACAATGGAACCTTTGGTTCTGTACTCCATAAGTGTTAAGGTGGAAGAAGGATGCAGACTCAATTCAGCAGACGAGATAGCAGGTGCAGTCCACCAGATGCTCAGAATTATTGAGGAGGAAGCTACCCTATGTGTTGTTGATTCTAGATAG

AabHLH41

ATGGAGCGGAATGAGTTGGAGCTCTTAAATTTGGCATCAAATTCAGCACAGCTAGAATTTTATAAAGAAGCAGGGATTAAGACTGCAATATTTATGGGGAGCAGCAATGGAGAGATTGAGCTTGGCATAACCTATGACTCCTCTCAAAAGTTGTTTCCTGGTGATTTCCCGCAAGGAGTGCTTCCTCAACGACTTGAGCAAGCTCGGGCTTCATCATTTTTAAGATCATTATCAATGGAAAATAGCGTGGAGAACTCGCCATTTCTCTTCAACATGCTTCATTCCACTCCTTATATGTCCGAAATGTTTGCTCTAACAGAACCATTAATGGACCAACAAGCACCAAATCAAACACCACAGTCAACAACAATATTGAGATCAGAAGATCCTCTGCAGCAAGCTTTAGAACAAATTAGAATCTCTCGATACGGATCAAGTTTAGGACCTAATAAACGAGTTCAAAATCGACAAAATCTTCACCGAAGGTCACTTTCATTCTTCCGAAATCTAAGTGAGGCTCGTGCTCAAAGAGATCAAATGGTCCAAACAACTAGACCTACTAGCAATCAATTTCTTCATGTGATAGCGGAGCGAAAGCGAAGAGGAAAACTCAATGAAAGCTTTCAAACTTTGAGATCACTGCTTCCTCCAGGGTTCAAGAAGGATAAGGCATCAGTGTTACTAAACACAATGAAGTACATATCTTCTTTAAAGTCTCAAGTCAAGGAGCTTAACAAAAGAAATCAGATTTTGGAGGCTGATCGGCGTGCCGGGAAAGAACCTCCTAATCAAGGTTCCAGTCGTTTTTCTGGAGAGGGGCCTGTAGTTGGCATCACCGACATTGGTGAATCAACTTCAGAATCACGAGTGGTTGATTTGGATGTGAATGCAAGAGGGAATGTGGTACTGGTGGATTTGGTGATGAGCGTGTTGGAGTTCATTAAACAGACCGAGAATATAAGTGTCATGTCTATAGATGCTGGGATTCGAATGTTGGATACAGAACCTATCGCAAATCGAGTTATTTTGAGATTAAGAATTCAGGGAAATGAATGGGACAAATCAAGCTTCGAAGAAGCAGTGAGGAGGCTTCTTGATGACCTGACACAATGA

AabHLH42

ATGGATATGCCATCCGGCTTATGGTTGCCTGAGCTGGAACTGGAAGATCCTCGTTACTTTATGAATCAGCATCAGCATACATGCACGTATAGTGAGCTAGTAGATTCATTTTCTTCGCAAGGATTCAAAGGTTACATAGAATCTAATCGTGCAACCACGGTTAGCGAAAATTTGGACAAACAAGAAAGTTACAAGGCAAGTAGAACTAACCCTTTCACTCCTATTGGCGGATCCACAGCTAATACATTCACTATATCCTTTGGAGACCACAACGAGATAAACAAAAATTCATTGCATGGAGGATTTCAGCTGAAATACAATGATGCAATAAAACCTAAAGTGGAGATGAGTCTCAATGAACTTCTTGACTCCATAGAGATTCCTAAAAGAGTACCCAGCACCAGAAGAAATCATAGACAAGCTCAAGAGCATGTCTTGGCTGAGAGGAAGAGAAGGGAGAAGCTGACTCGACGCTTCATTTCCTTGTCAACTCTCCTTCCTGAAATAAAAAAGATGGACAAGGCTACGGTGCTGGAAGATGCAAGTAAGTATATTAAACACCTTCAAAACCGAGTGAAGGAACTTGAACAAACATCAGTTGGTGAAAAACACATCATCCTGGAATCAATAACTTCAACGAGGAGCAATTTTCGCAGTAGTCATGAAGACAATGCATCTTCTTATGATAAGATTAACTCATTACCTTTTAGCACCGATAATGATCCTGGGATTAAAGTAAGAATTTCAGGTAGCCACACACTTGTAAGAATTTACTGTCAACGAAATTCTTCGCTTGCATTAAAAGCCCTCATCGAAATGGAGAGACTTCAATTTATCATCATGTGTAACAACGTTCTTCCCATCTCTGGAAATGCTGCTCTAATAACCATAATTGCTCAGATGAGTGAAGAGTTCGAAATGACAGCGACGGATCTTGTAAACTGCTTGAAATCGTCTCTTTAA

AabHLH43

ATGGAACCATCGTCATGGCTACCGGAACTAGAAATGCAGGATCAAGGGTTCATAAATCAGTATCAGATGAACAAAGCTTATCATCCTCTAATGGACGACTTCAGTGTCGATTCATTTTCATCAGAAAGCTACACAGAAAACCCATCTTTTATTGATCAATCTTTTCAAACTCGTAAGGGAGTTGAAGAACAAGCTGATATTAAGCAGCCGACTAGTTACAAAAGGGCCAATAGTATTAACAAGAAATTTATTCCTATCGATCAAAAACCGAAACCAAAGCTTGTTTCTGATCCTCCCAACGCTTTCACTATATCTTTTGGAGATATAAAACCTAAAGACGAAATACTTTCTTTTGGTGATTCATATGGCTTCACAGGAGCTGGTAGCAAAAAGGTACCCGCAATGATTAGGAATCCGATTCAAGTTCAAGATCACGTGCTGGCCGAGAGGAAGAGAAGAGAAAAGTTGGCTCAGCGGTTTATTTCTTTGTCTTCCCTCCTTCCTGACCTGAAGAAGATGGATAAGGCAACTGTATTAGAAGATGCAGCTAATTACATTCAAGAACTTCAAGGTCGAGTGAAGGAACTGGAGGGATTATCAGGCTTGAAGCGAAACAACATGCAAGAATCAGTTATATCTGCTAAGAGATCAAGGCTTAGTTGCAGTGATAATGATGGTTCTTCTAGTAATGAAACCAACTTTGAAGAGAGTAGTAGTCCGTCCACTCCCGAAATTGAAATTAGGACATCGGGATGCAGTCTACTAATCGAAATATATAGCCGAAAAAACTGCATATCACTAGTGAAAGTGCTAAGCGAGATGCAGACGCTTGGTTTATCTGTTATCAGTAGCAGCACCATGCCGTTTGCTGATACCTCTCTTCTTATCACCGTTGTTGCTCAGGTATATTCATAG

AabHLH44

ATGCTTGATAATTCAGACATGTTTGAACCCAACACATACAACCTTGATCCAAACACATTCTCCCATCTCATTGACCATAACCATAACCTTAATAACCAAGAACAACAACTCAACTGGACCAACAACCTTAATAATCTCGACGTCCAAACGAACCAACCCGACTTGCTAAACCTTTTTCAGCTGCCAAAATGTTCATCTTCTATATGTTTTTCCAACCCAAGTCACATGGACCAACAAGTAATGTACCATGATCATCATAGTACATTACTAGGAATGCATAATAGTAATAGTACTAATGTAGGTGTTACACAAAATGTTCCGTATATTCGTGAGCTTTTGCATAACGGGTTTAGCCTAAATGGATGTAACTCGTCGATTTTTGGCGAAATGGATATGGAACATAATGGTGGTTGTGATGGTATTTTGGAATTTGGTAAAAATGGTAATGGTAATAAAGATAATGTGAAGCATTTTGCAACTGAAAAACATAGGAGGCAACAATTGAATGGAAAGTTTGATGCTTTGAAGAGTTTGGTGCCTAATCCTAGTAAGCCTGATCGCGCATCAGTGGTTGGCGACGCGATTCAATACATACATGAGCTCAAAGGGACTGTGGAAGAACTCAAGCATTTGGTGGACAGGAAAAGATGCAACAGAGGCAGGATGAAAAAACACAAAACTGAGGACGACTCAACTCTAGATGTCGAAAGTATTTACACAATGAGCAATGGCGTAGTTGGTGATACTACTCATGATCAGCAGGCCTATAACGGCAACTCATCTTCATCAATGAGGAGTTCAATGGTACAAAGGAAATCAAAGCACACTGAAATCGATGTCCGTATCATTGACGATGAAGTTACGATCAAACTAGTGCAACAAAAAAAGATCAACTGTTTGTTACTTGTTTCAAAAGTCCTTGATGAGCTTCAGTTGGACTTTCATCATGTTGCTGGTGGACTTATTGGTGATTTCTATAGCTACTTGTTCAATACCAAGATATGTGAAGGGTCCTCTGTTTATGCAACTGCTATAGCCAATAAGCTAATTGAGGTGGTGGACAAACAATATGCAGCAATGCCAGTCACTTCTAGCTACTAG

AabHLH45

ATGTCTATGGCTGTTCAAGCTTATTGTGGAGGCAAAAACCCTGAAAAGAATTATTGTGATAACAACAACAACAATAAAAGTATTCATAATAATTTAGGGGTTCAAAAGAAGAAAAGAAGAAGGAGGCCTAGGGTTTGTAAGAACAAAGAAGAAGCTGAATCTCAAAGAATGACTCACATTGCTGTCGAAAGAAATCGTAGGAAACAGATGAATGAACATCTTGCCGTCTTACGATCCCTCATGCCGGAATCTTACGTCCAACGGGGTGACCAAGCTTCCATTGTTGGTGGTGCTATAGAATTTGTCAAGGAGCTTGAACATCTTTTGCAATCTCTTGAAGCTAAAAAATTTGTCATGACTCAACAACCACAAGAAGATGACGATAACGGAGGCCATGATAGTAATTTCACCAAATTATCATCAGCTGCCCCTCCATTTTCACAGTTTTTTTCATACCCGCAATACACTTGCTCTCAAATTCCCAACAAGTACACTTCTAAAAGCAAGGCAGCTATTGCGGACATTGAAGTGGAAGAAGGATGCAGACTCAATTCAGCAGACGAGATAGCAGGTGCAGTCCACCAGATGCTCAGAATTATTGAGGAGGAAGCTACCCTATGTGTTGTTGATTCTAGATAG

AabHLH46

ATGCAACCGACAACCGGTGGTGGTGGTGGTATTGGTCTCTCACGCTTCCGGTCAGCACCAGCAACATGGCTAGAAGCACTCCTGGAATCTGAAGAAGAAGATGTCATCATTGATCCACCCAAACCACCTTTAACACCACCACCTCATACCCATCCTTTTCATCAACAACATAGTACTGGGCCCACTTCTAGACCTCCTGCTACTTACGTGGATCCCAGTTCTACTATGTTGTTACCACCTACTAGTAGTGGTGGTATTAGTGTTGCTAACAGGCAGAATAGTTTGCCTGCTGAGTTCTTTTCTCAGATCAATGCTCCTGGAGATGGTACTTTTGTTTCGGGGTACTCGAATTCTGGTTATGATGATTATGTTTCGCCTTCTGGGTTAGATGGTCAACGAGCTAAGTTTACTACACAGCTGAGTGGAGACCAGAGTGCATTGTTGGATGCTGAGATGGACAAGCTGCTAGGGGAGTCAGTGCCGTGTCGAGTTCGAGCAAAGCGCGGATGTGCTACTCATCCTCGGAGTATTGCCGAGAGGGTTAGGAGGACTAGAATTAGTGACAGAATAAGGAAGCTACAAGATCTGGTTCCAAATATGGATAAGCAAACCAACACAGCGGACATGCTAGAAGAAGCTGTTGAGTACGTCAAGTTTCTGCAGAGACAGATTCAGGAACTTAGGGAGCATCGAGACAAATGCACATGCTTGGTTAAAGCTTGA

AabHLH47

ATGGACAATGTTGTTTGGTCTACTTCTTGGACACAAGATGGTGGTGTTTTTGATCAATTCAACAACTGGGATCGTGCTAATTTACTAGTTCCAAATCATAAAGGGGCTGGATCAAGTGCTTCCAAGAACCATAGTGAAGCCGAAAAGCGGCGTAGAGATAGGATTAATGCACATCTTGCTACTCTAAGGAGACTCGTCTCCAACTCGGATAAAATGGACAAGGCAACATTATTAGGGAAGGTCGTTGAGCGTGTAAAAGATTTAAAGTTGGAAGCAGTGGAATTAGGCAAAGTGTTTACAAATCGAGTAGAACTATTTTCGGAGATCAAGGACGCCCTCAATAGCCTCGGATTAACCATGGTTCAAGCCGATATGACTTGTTTGGGTGGCAGGATTATTTGCAACTTCATCCTTTGTTTGACGAATAATACAAGTGAGAATGAAGTTACATCAATCAAACACTCTCTCAAGATATTACTAAATAGAATTGTTTCGACGTCTTCTTGGACAATGTCATCAAACTATCGCATCAAAAGCAAGCGGCAAAGGTTCTTTTGTTCTTCTAGTTATGACACCAATGGCTGTGAATAG

AabHLH48

ATGAACAACAATCATGAATATGAAACCAACAATATGAACTCTTTTAACTCGCTACATGAACTCGAAGAAAATAGCAACAATTGGTACTTTAATCACCATGAAAACAATCTCTCTTTTGCGCCGAGTTTACCTTCAACTTACCAAAATTTCGATACAAAAACTAGATTTGATTCACAACCCCACATGGATGTTCCAAATATAATCTCAAACCATCATTTTCCAACGAACCATTTGGAAAACGCGTTTGGTGCATCAGGTTTTCAGGGTCTTGGACTCATGGGTAGTTCAAGTAATACAAAAAGTGGCGATGGACCAAATACTTTGGTTATGGATGATGTTGATGACATCAGTTTTGATGAGTCCGTTTTGAATTATGAGTCAGATGACGTCGCTAGAAAGATGGATTCTGGTGTAGGAAACATAGGAGGTTATAGGAAAGGTAAAAGAAAAGGGTTTCCTGCAAAGAATTTGATGGCAGAAAGGCGTCGTAGAAAGAAGCTGAATGATAGGCTTTTCTTGCTTAGGTCTATTGTGCCAAATATCAGCAAGATGGATAGGGCTTCGATTCTTGGAGATGCCATTGAGTATGTCAAAAAGCTTTTGCAGAATGTAAATGACCTTAACCTTGAACTGGAATCAACCCCTTCAACCTCTTTGATGCCCCCTGTTGTCGCCCCTACTACCACCACTGGATTATATCAGCTAACCCCGACTGCCACCATTGTCCCTTCATGTATCAAGGAAGAGGTTTTTCAAACTGGACAGCCTATCAAGATTGAGGTGAGGCAAAGAGAAGGAGGGGTGTTGAATATACACATGTTTTGTAGTCAGAAACAAGGCCTTTTGTATTCTGTCATGAGGACTCTTGAAGATCTCGGTTTAGACATCCAACAAGCCGTCATCAGCAGTTTTAATGGTTTTGCACTCGATGTTTTCCGAGCTAAGTATTCGGTTTTTTGCAGCATTACCTTGAAGGCCAAGATATTAACAGTGATCACATCAAGGCGGTGCTGCGTGAAACAGCTGAATATCATGGGGTCATGTAGATGTGGTTCACTGCCATCAGTGGAAGCAAGTACTTTAGGAGTTTAG

AabHLH49

ATGATTAGTGAGTGTACTTTTTTACCTGATTTAATCCATACATATGTCGATCAGTTTGGGGAAACCAACTTCTTGTCACCTGAGACAGTGTCTGCTGACGATGTATTTAGGGGTTTCGTGGCTGATTTGGAGGGTGGTTCTGACAAATTTGCGGTTATGACATCCGTTGGGCGCTTACACGAGGCTGTGGAGGGGCGTAAGAGGAAGAAAATGAAGATGACTGCGGTGGAGCAGGATGGCGTTGGTTGTGGTAATTCAGATGGGCAGCAAAAGGTGTCACATATCGCGGTAGAGAGGAATAGGAGGAAGCAGATGAATGAGCATTTGAGTGTCCTTCGTTCGCTGATGCCTTGCTTTTATGTCAAGAGGGGTGATCAAGCATCAATAATTGGAGGTGTAGTTGATTACATCACTGAATTGCAACAAGTTCTACAATCCCTAGAGGCAAAGAAGCAAAGAAAATCTTACAATGAACTAGTCATGAGTCCGAGGCTACTTGCGAGCCCCAGAACCTTACCTATCAGCCCACAAAAATCGCAATTTAGCCCTAGACCAATTAGCTTGCCCATTAGTCCGAGAACCCCGCAACCGTTGAGTCCCTACAGACCCATGCCACACCTTCCATTCATATCACCATCGCTTGATTATCCTTCACCTTCTAATTCTTTTACAACTTCAGAGAGTGGTAATGAGCTCATGGCAAATTCAAGGTCAACGGGTGCTGAAGTTGAAGTGAAATTTGTGGGTGGTAATCTTCTTTTAAAGACTTGCTCGAATAGGATACCAGGCCAAGCAACAAAAGTAGTAGCAATTCTTGAAAATCTATCTCTTGAAGTTATTCAAGCTAACATTAGTGTCGTTGATGATACCATGTTTAACTCATTTACCATCAAGGTTGGAGTTGCATGCAAACTAAGTGCTGATGAACTTGCTCAACACATTCAAGAGACATTCTGTACCGGGAAGACAACTTTGTCTACTGATCACAACCGATACTTGATTGGAGATGATGAGCATTGTTGGAGTGACAAGGGTGTGTCAAATATTGAAGGTGGTTGTTATGCAAAGTGTATTGATCTCTCAAGGGAGAAAGAACCTGATATCTGGAATGCCATTAAATTCGGGACCGTGTTGGAAAATGTTGTCTTTGACGAGCATACTAGGGAGGTGGATTATCTAGACAAATCTGTCACAGAGAATACTCGTGCAGCATACCCCATTGAGTACATCCCCAATGCCAAGATTCCGTGTGTGGGTCCTCACCCGAAGAATGTTATTCTGTTGGCGTGTGACGCTTTTGGTGTGCTTCCACCAGTGAGCAAGCTAAACCTTGCTCAAACGATGTATCACTTCATCAGTGGCTATACAGCTTTGGTGGCTGGAACTGAGGAGGGTGTAAAGGAGCCACGTGCAACGTTTTCAGCCTGTTTTGGTGCAGCGTTTATCATGCTACATCCCACAAAATATGCAGCAATGCTTGCTACCAAAATGGAGAAACATGGTGCTACTGGATGGCTTGTTAACACTGGTTGGTCAGGTGGCAGTTATGGTTCTGGGAGTCGTATGAAGTTGGCATACACACGAAAAATCATAGACGCCATTCATTCTGGACAGCTTTTGAATGCTAACTACAAGAAAACCGAAGTGTTTGGGCTTGAGATCCCAACTGAGGTCGAGGGTGTGCCTTCTGAGATTCTTGATCCAGTGAACACCTGGTCAGACAAGAATGCGTATAAGGAGACATTGCTGAAGTTGGGTGGGCTGTTCAAGAGCAACTTTGAAGTGTTTTTGGACCACAAGATTGGGAAGGATGGTAAGCTGACTGAGGAAATTCTTGCTGCTGGTCCCAACTTTTAA

AabHLH50

ATGATGCAATATTTACCAAGTCATGGCTATGAGTTTGCTAATTGTGCAAGTATTATAAGAAATTTAGTATATGATCATGGAGCGAAAGATTTGTCAAATGACACGGAGGCAAATGTAGCGGCAGAGGCAGAGGCGAAAGCGGCAGCTGCTTGTAATCGACATAGCGAAGCTGAAAGGAGACGTCGAAAGCGAATAAACGGCCACCTTGCCACCCTCCGGAGCATACTCCCCACTACCGTTAAGACAGACAAAGCATCATTGTTGGCGGAAGTAGTGAGACAGGTGAAGGAGTTAAAGAAAATGGCCGCGGAAATAGAATCCGCGGCTATTGATCAAGGTGATGACATTATACGAAACAACGAGTACTATATGATTCCAACTGAAAAGGACGAGCTCGAGTTGACATATGTTGGAGAAGATTCTAGTACCAAAAAGATTATGATAAAGGCACGATTGTGCTGCGATGATAGGGCCGAACTGATAGCTGAACTAACGAGGGCGTTGAGTTTAGTACATGCAAAATTGGTGCGGGCGGAGATAGGGACATTGGGCGGGAGAATCAAATGTTTGTTGTGGGTTCAAGTCTCAACGGTAACTATAGACCAAGGCATACATGAGCTACAACGATCTTTAAAGGTGGTTATGGATCGCGCAACGTTATTGGATATGCCACGAAACAAGAGACCACGTAGTTTCTCGGACTCTGTCGAATTTTAG

AabHLH51

ATGTATGGTGTTAACAATTCTTCAGATGCAATATCTAGAGACATGAACTCAATCTTGTACTCATCAACTTTCAAACATCCAGCAGACACTGAGTTTGCTAAGATTAAACAGCTGATATCCTTAGACAACAACAATAACTCCTATGAAAACCCTAGTACTCATCCACAACACCAAGAAAACAGCGAAAATCCACCGTTAGTAAGTTATCGGTCTACTCCTAGCTCTTTTTTCTCGAATCTATTAAACGAGAATGAAAATGATGCCTTCCAAGATCATGAACCTGAGGAAATATATTTTATGGACCAGCAACAACATAAGAAGAAGTCTGATCAATCTGAAGCATATAATAATATGAAACGTGAGAAACAAGAAATGGGTAGTAAGAATATTGAAGTTTTGGGTTATGGTTATTCGAAACAAAGTGATTTGGATTGTGGATCGTCTTTTAGAAGTGATTTGGTTAGGCAAAGCAGCTCTCCTGCTGGATTTTTGTCCTCGTTGACTGGTGAAAATGCTTTTGCTAAAGATCTGAGAAACGGAAGTTCATCGAGTTCATTTAATAGCCACATCAGTTTCTCGTTAGGGTCATCTTCTTCTTCGTCGAGATTTTTGCCCCAAATAGCTGAAAACGAGAATGAGTTGAATGATTCCACATTCCATAGCTTGAAAAGGAGCAGAGATGGTAGCTTAAAGATGTCACAGAATGGAGAGACTGTAAACCACACTCCTAATTTGGTCCACCATGTGAGCTTGCCAAAAACGTCTTCTGAAATGGCTGCTGTTGATAATTTCTTGCATTTCCAACAAGATTCATCGGTTCCTTGGAAAACACGTGCAAAAAGAGGATTTGCTACACATCCACGAAGTATTGCAGAGAGGGTCAGAAGAACTCGAATCAGCGAAAGAATCAAAAGGTTGCAGGAGCTTTTCCCTGATATGGACAAGCAAACTAATACTGCAGATATGTTAGATATGGCTGTTGAATACATCAAAGACCTTCAAAATGAACTTCAGACTTTGAATGATGCTCGAGCAAGGTGCAAGTGCTCAAAAGAGCAATTACAGTCCGGTTCGACCATGTAA

AabHLH52

ATGGACCCAAATTTGTATTTGATTTGGGACGAGGACGAGGATGATGTGAAGGAATCTGGAGATGGTGATTCCTCTGAGACGGTGACTACCCGTAATCCAAATACACAAAGGAGAGGTGGTGGTGTGAAAGGTGATCGTACAAAGTCTTTGATTTCCGAGAGGAAGAGGAGAATTGGGATGAAGGAGAAGCTATACACACTGCGCACAATGGTGCCCAACATCACTAAGATGGATAAAGCTTCGATAGTTGGGGATGCAACACGATACATTCAAGATCTTCAAACACAAGCAAGGAATTTAAGGTCAGAGATTGCAAAAATTGAAGTGAATAGTAACCATAAAAAGGCTTCACAAAATTCGAAGATGGCCAATGTATCAAACTCCCTCCCAATATTTAAGAAGATATCAAAGTTAGAAATGTTTCATGTGGAAGAAAAAGGATACTATGTGAAAGTAGTTTGCAACAAAGGCCGAGGTGTTGCGGTGGCCCTTCTTAAAGCACTTGAGTCGATCACAAGCTTTCAGGTTCAGAGCTCGAATTTGGCAACCCTTGGTGACACTTTTGAGTTAACATTCACGTTAAAAGTTGCTGCACGCGAATTCGATAAAAAACCACCAAATTTGAAGCTACGGCTTTCTGGGGCTTTCCTTAAACAGGGTTTCAAATTTAAGTAA

AabHLH53

ATGCTTGAAGATTATTCAACAATATTGGAACAAGTAGATGCTAAGGTTCATGAGGAACATCAAGTGGTTGTGTCGGATAATCAAATTCCTAATGTTTTCAATATTGGAGAAAAAAATAGTAAATCTAAGAATGTTCAAGGGCAACCATCTAAGAACCTAATGGCAGAAAGAAGGCGTAGAAAGCGGTTAAATGATCGTCTTTCCATGCTTAGATCAATTGTTCCTAGAATAAGCAAGATGGACAGAACATCTATACTTGGTGATACAATAGAGTATATGAAAGATCTAATGGAAAAGATCCGTAGCTTGAAAGAACAAGATACAGGATTGGATTCGAGTGGTCTGAATGAGTTAAAAGTGAATCAAACACAAGAAAGAAACTCCCCAAAGTTTGAAGTAGAGAGGAGAAACATTGACACTCGTATACAGATTTGTTGTGCACCGAAGCCAGGATTGTTATTTTCTACAGTGAACACTATTGAGTCCTTAGGCCTAGACATTCGACAATGTGTTATTAGTTCTTTTGGTGATTTTACCCTTCACGCTTCGTGTTATGAGGCACCGGAAAATCAAGTGCTTGCAAGTTCTGAGGAAATTAAGCAAATATTATTCAAAAATGCTGGTTATGGAGGTAGAGGTCTCTAA

AabHLH54

ATGGATATCCCATTTGGCGCTTGGTTGCCTGACCTGGAAATGGAAGATCCTTGTTACTTTATGAATCAACATCAACATACATATCCGTATAGTGAGTTAGTAGATTCATTTTCTTCCCAAGGCTTCAAAGGTTGCATGAATTTGGTCACAAGAAGTGAAGCTATTCACGCAGCAACCATTAGCAAAGAATTAGACAGTCAAGAAATTTATAAGGCAACTAGAAATAACTCGTTCACTCCTATTGGCGGATCTTCATCCAACACATTCACTATATCCTTTGGAGACCACAGTTCATCATCAGAGATTAACCAAACTACATTACCTGGAGGATTCAAGTTAAAATACGATGATGTAATGCAACCTAAATTAGAGATGAGTCTCAATGAACTTCTTGGATCCATAGAGCTCCCTAAAAGAGCTTCCACCACCAGAAGAAACCATAGACAAGCCCAGGAGCATGTCTTGGCTGAGAGGAAAAGAAGGGAGAAGTTAACTCGACGTTTCATTTCCTTGTCTGCTCTCCTTCCTGAAATAAAAAAGATGGACAAGGCTACAGTGCTGGAAGATGCAAGTAAGTACATCAAATACCTACAAAACCGAGTGAAGGAACTCGAAGAAACATCAGTTAGCGGAAAAAATGTCATCCAGGAATCAACAACTTCAATGAGGAGCAAGTTTTATGGTGGTCATGAAGACAATGCATCTTCTTTTGATGATACTAATTCTTTACCTATTAGCACCGCTAATGATCTTGGGATTAAAGTAAAAATATCAGGAAGCCACACACTTGTAAGAATTTACTGTCAACGAAATTCTTCTCTTGCATTACGAGCCATCACTGAAATGGAGAGCCTCCATTTCAGCATCATGTGTAACAACGTTCTTCCCATCTCTGGAAATACTGCTCTAATAACCATAATTGCTCAGATGAATGAAGAGATCGAAATGACAGCCATGAATCTTGTAAATTGTTTACAATCATCTCTTTCAAACTTTCTATGA

AabHLH55

ATGAAGCAGTCTGAGATGGGAGGTATAAAAGGTTCGGGTAGTGGTGAGCAAGAAGGGGTAGCTATAACTGTCACTAGTGGTAGCAAGAGGTCTAGTGATGCTTTTGAAGACGACGTCAAGATTAATCGTCCTCTTAGTGATCATGATTTGCATATATTGACAGAGAGGGAGCGAAGAAAGAAAATGAGGAATATGTTTCATCAGCTTCATGCCTTAGTACCTCATCTCCCTCATAAGACAGATAAGTCTACCATAGTTGATGAGGCTATAAGCTACATTCAAACCTTAGAAGAAACCCTCCAAAAGATTGAAACAAAGAAGCTAGAGAAGCTTTATGGTACTCAGTCTGCAGCCAACTCGACCACTGTTTCTCCAATTCAATCACCAAAACCAGCTTTGGACACTAGGGAATCCTTTTTAGCCGATCAAGGATCCTCAACAGTCTCGCCTTCTAGTTCAAGCACATTTTCCTTTCCAATATCTTCACCAACGGTTTTCCAAACATGGGCTTCTCCAAATGTCACTTTAAATGTGTGCGGCATGGATGCATTTTTTAACATTTGCTCCTTCCCGAAGCGTGGGCTATTTACTGCAATTTGCTTTGTGTTGGAGAAAAACAAGGTGGAGATGGTTTCTTCTGAAATTTATTCAGATCAATGTAAATGCTCATTCTTTATTCATGCCCATGTGAACGCTCGTGATCAAACTGTGAAGGATTTGTCTTTTGAAGAGATATACAAGCAGGCTGCAATGGAGATAATGCGTTGTGTTAGCACAAAATCGCCATAG

AabHLH56

ATGGATTCAGATCTTCATCATCAACTCAACCATCTTCCCCAACAACAAACCATGAACACTCCAGGGTTAACAAGATACAGATCTGCTCCTAGTTCATACTTTTCAAACCTGATCAACAGTGGAATATATGATGACACTGATCAGTTTTTCAATACTAGGGTTTCCTCAAGTAGTACTACTGATCAGATATTGTCAGGTTATGAGTCTAGGTTTATGATGGATTCTGTTAAGCAAGAACAAGAAGTTATATATTCAACACCACAACCAAGTACAATGAATTATCAACAACAACAACAACAGCAGCAACAGAATCAGTTTCAGTCACAAATGGATCAGCAGACTAATAATAGTAATACCTCAGGGATGGATAGTTCGAATAATATAAGGAGTAATAATTTGCTAAGGCAAAGTAGCTCACCTGCCGGGTTTTTCGATCAGCTCGATATGGATAATGGTTTTTCTGCGATGAGATCGTTGGATGATTTCAAAGTTGGTGGTAGGGGTATTGGAAACTCAATGCTCTCCTCGACAAAGAGAATGAAAAATGAAATGGGATTTACGTCTAGTTCACTAACGTCTTCTGGTATATTGCCGCGTATCCCTGAGAATGAGAGCAAAGTTATGGATATAAAAAGTGGTCGTGATGATGGAGGCTTTGGTTCACAATCATGGGATGATTCGGATATCTTATCTGATAGTTTCTTGAAAGACTTTGGAGAAATTGATCAAAGTAAGATATCGAGTTTAAAACCATCTGAAAATCAGAATGATGTTGGGAGAATACGTGCTCCCAATACGCTTGTGCATCACATGAGCTTGCCATCAAGTACAGCCGAGTTGGATAAACTGCTGCAATTTCAAGATTCTGTGCCTTTAAGAAGCAGAGCAAAAAGGGGTTGTGCAACTCACCCAAGAAGCATCGCTGAGAGGGTAAGACGGACCAGAATAAGTGAAAGAATGAGGAAGCTACAAGAGCTTGTCCCAAATATGGACAAGCAAACAAACACAGCAGACATGTTGGATTTAGCCGTTGACTATATTAAAGAACTTCAGAAAGAAGCCGAGGCTCTTTCAGACCATCACGCAAAATGTACATGTCCACATAAAGAAAAGCTGTAA

AabHLH57

ATGCAAGCATTATCTCCTACATTGTTTTCCACAACATATGGATGGCCTTCGGAGAATAACATCACCCAAAATCACCAACAAGATTTCAATGATATTTATATGGACGTTGAAGCAAATTCGTATAGTCCCCTTCTTGATATTCTATCATGTGATCAAAGCCAGCAAAATTGTGCACCCAATAGTTGTTCTTCTAGAGGAGCTATAGATGGAAATACTGCGGATCCCATGAAGGTGTCCAAAAAGCTTAATCATAACGCAAGTGAAAGAGATCGGCGTAAGAGGGTTAATGATTTATATGCATATCTTCGTTCGTTGCTGCCCATATCAGCTGATCAAAAGAAAAAAGTAAGCATTCCTCGGACAGTATCACGTGCACTGATATACATACCAGAACTACGAAAGGAAGTGGAAACATTAATACGTAAAAAGGAAACTCTATCATCGCACTCATTATCAACCACGAGTACAAGCCCAAAGAATCTCGGCATCAAGAGACAATGTGATAAAGATGTTAACATTAATATGAACTCATCGGTGGTTTCTTCTGTGAGCATTTTAGGTGAGAAAGAAGTGGTGATACAGTTGATTTCCTCTACAGATCAAATGAGCAAGAATAAGGATATTGGCTATTTGTCTAGGGTTTTGGCATACTTAGAGGCCGAAGAAAATGGATTTGTTTTGCTAAATTCAACGACTTTCAAATGTTCGGGAGATGAAATGTTATTAAACACTATACATCTTCAGGTGCAAGGTGATAATTATAAAGTAGAAGCTGAAATATTGAAGGAGAAGCTCTGCCGTTTGAGCCAACAAACAAATGAGCTTTCACCCTAA

AabHLH58

ATGTCTAATGGTGCCAGTGGTGGAGCTAGCTTGAGTTCTCATGAGAATTCAAGTCAAATTCGAGTAAAAGAATCGCGGTTTCTTGATCATATGAAACATGGACAACCTAGTTTTACAACTCAGCAACATGTGATGTATCAAAACCATGCTCAGTCTAAGCAAGGTGTTCCTAGCTCTAGCTCGGGTTTTTCGCCAATGAGTAACAATCTTATTCGTCAAAGTAGTTCTCCAGCTGGATTTTTTGAATATGTTAATATAGATGATGGTTACTCTATGATGAGAGCGATCGATAAGTATCGACATGCTAATGGGAGTGTTCAGGATTCACAGATGGCTAAAAGCAGGATGGTGTTTTCATCAGGTTCTCATTCGCTGTCTCGTATACCTGAAAATGAAGGGAAAGGGATAGCGAATAGTAATGGTGGTTATGTTGATAGCTTTGGTGGTGGTTCTTGGGATGCATCAGTTATGTTAAATGACGGGTTCATGAAAGAATTTGGAGAGAGTGATGGAATTAGCTCATTTGATAATCAGAATGACGAAGGGCAGATTCGGGTTTCTAATGGTTTAAGTCATCAGTTGAGTTTGCCAACGAGTTCGACTGAACTGTCTGAAATGGAAAAACTACTGCAGCTTCAAGATAACGTGCCTTTAAGAAGCAGGGCTAAAAGGGGTTGTGCTACTCATCCAAGAAGCATTGCTGAAAGGGTAAGACGAACACGAATAAGTGAAAGAATGAGGAAGCTACAAGATCTTGTCCCAAACATGGATAAGCAAGCAAACACAGCAGACATGTTGGATTTGGCAGTAGACTATATTAAAGAGCTTCAGAAACTATCTGAGAAACTTTCAGACCATCATGCAAAGTGTATGTGTCCACATAAGCGGAAACTATAA

AabHLH59

ATGAGTGGTGACTGCACTTCTTTAACTGATCTCTTTGATACTTATGTTGAGCAGTTTTCGGATACCAGCTTCCTATCACCCACTGCAGCATCCTCTGACGACATCTTCAGTATACTTGAAGTTCTTGAGGGTGTTTCTGATGATTTCAAATCCATGGCGCCTATGAAGAGTGGGCCATTTCATGTGAAAGAAATTAAGCATCCACTAGTCTCTCAGAAGTCTACTTCTTCATGTAGTGCTTTACAGGAACTTGTAGAGGAGAATATTGAGACACCTAACTCGTCAAAGAGGAAGCGACAGAAGCTTTCGTTGGTTGAAGAAGGCGGGGTTAACTCAGATGGACAGACAAAAGTGTCACATATTGCAGTGGAGAGGAACAGGAGGAAGCAGATGAATGAGCACTTAATAGTTCTTCGTTCACTAATGCCTTGCTTTTATGTCAAAAGGGGTGACCAAGCATCAATAATAGGAGGAGTAGTTGATTACATAACTGAATTGCAACAAGTTCTTCAATCACTAGAGGCAAAGAAGCAAAGAAAAGTTTACAGTGATGTTTTGAGCCCAAGACCAGCACTCAGCCCTCGTAAACCGCCTATTAGCCCTAGGCCACTCTTGCCGATTAGCCCTAGAACTCCACAGCCACTAAGTCCATATGGAGCTAGATCTCCAGCTGCATATAACTCCTCTTATATGTCATCATCACAATCATCAATGTCTAATAACATGATACCACATTCATTTGACCCTTCGCCATCTAGTTCTTCATCAACTTCAGACATCATTAATGAGCTAGTTGCAAATTCAAAGTCATCCATTGCTGATGTGGAAGTTAAGTTTTCATCGGGCTCTAATCTTATTTTGAAAACAACCTCACCACGGTTGCCAGGCCAAGCAACGAAAATAGTTTCGGTTCTTGAAGAGCTCTCTCTTGAAATCCTTAAAGCTGACATCAACACTGTCAATGAAACTATGGTGAATTCCTTCACCATTAAGATTGGAATTGAATGCCAGCTAAGTGCGGATGACCTGGTTCAACATGTCCAACAAACATTCTGCTAG

AabHLH60

ATGGAGATTACACAATTCAGAGGGTTTTCGGAATTGTCAGGAATTGAGGACCCTTGTTTTAATTCTCAGTGGCCATTTAACTCATTTGATGATCAACTAATGAACGTGGATGCATGTAACTCATTTGCTTATGCACCCATGTTTGATCATATTCATTATAAGCCTATTATGGAAGCATCACCAAGACCATCTAAGCAACTCAAAACCAGCAGCTGGAATTCGAGCATTAGCTCTGACCAGTCCATGATGAATCAGACTATACATTATGGTAGTGATCATTCGCATGTCGCAAATCAAGCTACCCTTATGAGTCCTAAAGAAGAAGCAACTCTATCTTCCAGCAGTGTATTCCAATCATCATCCCATGTTCAGTTTGGCAACCAAAACCACCATGCGAAAAAGATAAATTCTTCAAAATTCACTACACCTCAAGACCATATCATGGCTGAAAGAAAGAGAAGAGAGAAACTGAGCCAAAAGTTCATAGCCCTCTCTGCTTTGATCCCTGGCCTTAAAAAGATGGATAAAGCTTCTGTTCTTGGAGATGCAATCAAGTACTTGAAAAACCTTCAAGAAAAAGTGAAGACACTTGAGGAACAAACCAAAAAAAGATCAAATGTAGAATCAGTGGTTTTTGTTAAAAGATATGAGGTGTTAGGTGATGGTGGTGAAATCTCTTCATCCGGCGAAAACTCTCAGGAGCAACTGCCTGAAATCGAGGCACGATTCTCAGGCAAGGATGTTTTGATCCGAGTTCATTGTGAGAAAAAAGCCGGTATAGTAGAAGAAATACTAGCTGAAATTGAGAAACTTCATCTATCTGTTATCAATTCCACTGCCTTGACTTTTGCCAATTATGCACTCGATATAACCCTAGTAGCCACGATGGATCAAGAATTCACCATGACAATGAAGGATCTTGTCAAAAATCTACGTATCGGTCTTAAACGATTAATGTGA

AabHLH61

ATGTTGTGTTACAATGAATTTGAACTCTTTCAGGGTGGCTTTCTTGGAGTCGATTTTAGTGGTGAGCATGTGCAAGACCATGAACATCAGAATCAGCAACAGTTCATGGTGAAAAACACGTACGAAAATGGGAATCAAATGGTGGATTACTTGCTTAATAATCCCATACAAAATCTACCGTCTAGTGGTTTTTGTAGCTCGAATTCTTTCGATAAGTTGAGCTTTGCAGATGTGATGCAGTTTGCGGATCTTGGACCAAAGTTGGCTTTGAATCAAAACAAAACAAGTCATGATCATGAAGAAGAACAAGAAAACGGGATAGATCCCATTTACTTCTTAAAGTTCCCAGTGTTGAATGAGAGGTCACAAGAAGATCATGAGTCTTTATTGGCTCCTCTTGGAGACGAAGAAAACGAGCATAGATTAGTGATTGAAGGAGGTGAAAGAGATGAAGAAGCTAGGGTTTCCGAAGGTAATTCTGTTCGACTTCAGTTTATGGGTGAAGATGTTCATAAAACCCTAGGTACAGAAGGCAAGAATAAGAGAAAAAGACCCAGAACTATAAAGACTAGTGAAGAAGTTGAGAGCCAAAGAATGACTCATATTGCGGTCGAAAGAAACCGTAGAAAGCAAATGAATGAACATTTACGCGTTCTTAGGTCTCTCATGCCAGGCTCCTATGTTCAAAGGGGTGATCAAGCATCTATTATCGGTGGGGCAATTGAGTTCGTGAGGGAGTTAGAGCAACTCTTGCAATGTCTTGAGTCACAAAAACGACGAAGACTCTATGGAGACACTCCAAGGGTGGCCGGTGATTCATCGTCCCTTCCAGTTGTGCAGCAAGGGCCTCCAGCAGTTTTTTACCCTCCATCTGATGAGCAGATGAAGCTAGTGGAATACGACGGCGGACTGAAGGAGGAAATGGCAGAGAGTAAGTCCTGCTTGGCTGATGTCGAAGTGAGATTATTAGGGTTTGATGCCATGATCAAGATTTTATGTAGAAGAAGACCTGGTCAGCTTATTAAAATTATTGCCGCACTTGAAGATTTACAGTTTACTATCCTTCATACCAACATTACCACCATTGAACAAACTGTGCTCTATTCATTCAATGTTAAGGTTTCTAGTGAAGCGAGGTTCTCAGCAGAAGACATAGCAAATTCTGTACAACAAATTATTGGTTTTGTGCATGCAAATACTAGTTCTTGA

AabHLH62

ATGGTGAATGAGTCTACTTCTTTAACTGATCTATTTGACCCTAATGGATCATTAGCATATCCTGACGACATCTTTAATATTCTTGAGGCGTTAGAGGGTGTTTCTAATGAATTTACGGTGTCTACACCTTTAGCTGATTCCTCGAGTGGTGTACAAGAACTCGTGGAGGCGGAACATGAGGCGTTTTCACCAAGAAACAAGAGACAGAAGGTATCGGGGGATGAAGGTTGCGAAAACTTTGATGGACTGGTAAAGATGAATCATGTAACGGTGGAGAGGAACCGAAGGAAGCAGATGAATGAACACTTAACCGTTCTTCGGTCACTCATGCCTTGTTTTTACGTCAAAAGGGGTGATCAAGCATCAATAATTGGAGGTGTGATCGAGTACATCACCGAACTGCAACAAGTGCTACAATCTCTAGAGGCAAAGAAGCAAAGAAAAGTTTATAGTGATCATGTGTTGAGCCCAAGGCTTATTTCAAGCCCTAGAACAACCCTACCTCTCAGCCCTAGAAAACCGCCCTTGAGCCCGAGGCCAATTAGCTTGCCAATAAGCCCCAGGACACCACAGTCAGCTAGTCCATACCGGCATAGGTTACCATCTGCTACTTCTTACCTAAATACCAGACCAGCTTCACCTTGCAACTCTTCATCAAATTCTGACACCATTAACGAGCTTGTTGCAAATTCGAAGTCATCCATTGCGGATGTTGAGGTGAAGTTTTCAGGAGCAAATCTTCTTTTAAGGACTTTATCACCACGGTTACCAGGCCAGGTGACAAAGATAATATCAGTTCTTGAGGACTTATCTCTTGAAATCCTCCAAGCTAAAATCAACATTGTTAATGAAACTCTGGTTAACTCATTTACCATCAAGATTGGAATCGAATGCAAGCTTAGTGCTGAGGACCTTGCACAACACATTCAGCTCACATTTTGCTAG

AabHLH63

ATGCTACCGGAAAACGACACCGCTTGGATGGACAACCACCAAGATGAAACATCTTCATGGACCCCTACAACCACCCCAAACCACCACCAACCTCCATGCACCAACCAAAACACCTACACTTCACTAACAACCTTAAAAAACATGCTAGAAACAGAATGGTTCCAAAACAACAACAACAACAACCTTAACCTCACCTCAACTGACACTAATCCTATGTTCTTACCACTAGATTCTTCATCCTCATGTTCTCCTTCACAATCACACTCTCACTCACATTTCACTCAGTTTCCATTCATACCCACTAAATATAGCAACAACCATAGTACTAATGTCAACTTAACTTCTTTCGACTTGCCTTTCGAATTCGGGTGTGACCCTAGCTTCATTTCCAACAACCCTCCAACTAACTTTTTGAATTTCCCTACCCAGCAAATGGCAACTAGCTCCGAGTATACAGAGACTAGTTACCATGATGACTTAACCGGAACTGGCACAGGGTTCACTGGCTTTGAGCCAGTGAACCCTGTTTTTCCGGTTAGGTCCAAAGTTTTGAGACCTTTGGAAGTTTCTCCACCTGTTGGAGCGCAACCCACTTTGTTTCAGAAGCGGGCTGCGCTGAGACAGAGTGGAGAGAAGTTGGATGGGTTGGAAATTGTGAAAAAAAGGAAAAGGGGTAGTGGGAGTGGGAGTGGGCCCCATGAGGATGAGATTGATGAAATGGGTAATGGGGATGGTTCGGGGTTTAATTATGATTCGGATGAAGTTGAGCTTACTGGTGAGGTTAATGGGAATGGTAATGGGGGTAATTCGGGTTCGGTTATGGTTGGGGATAGTAAAGGGAAGAGGAAGGGTTTGCCTGCTAAGAATTTAATGGCGGAAAGGCGAAGGAGGAAGAAGTTGAATGATAGGTTGTATATGCTTAGATCTGTTGTCCCTAAAATTAGCAAGATGGATAGGGCGTCGATACTTGGTGATGCCATTGACTATTTGAAAGAGTTGTTGCAAAGAATTAATGATTTGCATAATGAACTTGAGGCAACACCACAAGGTTCTTTGATGCAAGCTTCGTCGAGTATCCATCCATTGACGCCTACACCACCAACGCTTCCGCAACATGTTAAGGAAGAGTTGTGTCCGAGTACTCTTCCTAGCCCAAAAAACCATCCTTCAAAGGTGGAGGTGCATGCAAGAGAAGGGAGGGGTGTGAACATCCACATGGTGTGTGGGCGCAGACCAGGTCTGTTGCTTTCAACGTTGAGGGCCCTAGAGAACCTTGGCCTGGACATTCAGCAAGCTGTCATAAGTTGTTTCAATGGGTTTGCTTTGGATGTATTTCGAGCCCAGCAATGTAGAGAAGGGCAGGAGATGTTACCGGAGCAGATAAAAGCAGTGCTTCTGGAGACTGCTGGTTACCATGGTGCCATTTAG

AabHLH64

ATGAGGGGTAAAACTGTAGTTTTGTGCAAAATGTTAGGGCAAAATTCTTCATCCTCATGTTCTCCTTCACAATCACACTCACACTCACATTTTACTCAGTTTCCATTCATACCCACTAAATATAACAACAACCATAGTACTAATGTCAACTTAACTTCTTTCGACTTGCCTTTCGAATTCGGGTGTGACCCTAGCTTCATTTCCAACAACCCTCCAACTAACTTTTTGAATTTCCCTACCCAGCAAATGGCAACTAGCTCCGAGTATACAGAGACTAGTTACCATGATGACTTAACCGGAACTGGCACAGGGTTCACTGGCTTTGAGCCAGTGAACCCTGTTTTTCCGCAAATGGCAACTAGCTCCGAGTACACGGAGACTAGTTACCATGATGACTTAACCGGAACTGGCACAGGGTTCACTGGCTTTGAGCCAGTGAACCCTGTTTTTCCGGTTAGGTCTAAAGTTTTGAGACCTTTGGAAGTTTCTCCACCTGTTGGAGCGCAGCCCACTTTGTTTCAGAAGCGGGCTGCGCTGAGACAGAGTGGAGAGAAGTTGGATGGGTTGGAAATTGTGAAAAAAAGGAAAAGGGTTGAGCTTACTAGTGAGGTTAATGGGAATGGGAATGGGGGTAATTCGGGTTCGGTTATGGTTGGGGATAGTAAAGGGAAGAGGAAGGGTTTGCCTGCTAAGAATTTAATGGCGGAAAGGCGAAGGAGGAAGAAGTTGAATGATAGGTTGTATATGCTTAGATCCGTTGTCCCTAAAATTAGCAAGATGGATAGGGCGTCGATACTTGGTGATGCTATTGACTATTTGAAGGAGTTGTTGCAAAGAATTAATGATTTGCATAATGAACTTGAGGCAACACCACAAGGTTCTTTGATGCAAGCTTCGTCGAGTATCCATCCATTGACGCCTACACCACCAACGCTTCCGCAACATGTTAAGGAAGAGTTGTGTCCGAGTACTCTTCCTAGCCCAAAAAACCATCCTTCAAAGGTGGAGGTGCATGCAAGAGAAGGGAGGGGTGTGAACATCCACATGGTGTGTGGGCGCAGACCAGGTCTGTTGCTTTCAACGTTGAGGGCCCTAGAGAACCTTGGCCTGGACATTCAGCAAGCTGTCATAAGTTGTTTCAATGGGTTTGCTTTGGATGTATTTCGAGCCCAGCAATGTAGAGAAGGGCAGGAGATGTTACCGGAGCAGATAAAAGCAGTGCTTCTGGAGACTGCTGGTTACCATGGTGCCATTTAG

AabHLH65

ATGTTGTCTAGAGTTAATAGTGTTTCTTGGATGAATGATGATGAAAATAACCAAACAAACAACAACAACAACAACAACAACAAGTCATTACTTGATGATGTTCAACTTGAAAGCAACAACAACAACACAAACATTAGTACTCAAAACAATTGGTTTTCAACATCATCTTCATGTTCACCAAATTCATCTTTTCAAAACCACCCATTTTACATACCACCTAAACCCAACAACACAATTTCATCACTTTTTAACCCAAACCACAACCCATTAGATGAAACCTTTAACATGCTACCAAATTTTTGTGACTTAGGCTCACAAACTCAAACCCATATGGGTATCAACAATTTGACCCCAAGTTTACCTTTTCCGGATACCCTTTTGGCTCAAGTGCCCGGAACCGGGTTCGGTTCACCGGGTTACCCGTTTTCTGATAACGGGTCAACCGCCGGTTTAATGCTGAACCGGTCAAAAATGCTTAAACCCTTGGAGGGTTTTACTCCTACTGGTAGCCCACCAACTTTGTTCCAGAAAAGGGCTTTGAAAAGAAACTCCGGCGAACTTTCCGGCGCCGGCGAGTTTTCCGGTACCGGGGTGTTGGGGAAGAGGAAGAGTGGAAGCTGGGATGAAGTGAGTTTGGATGGGTCTGGTTTGAATTATGATTCTGATGAGTTTACTGCTGTTGCTACTGGTGGTAATGGTGGTGGTGGTGGTAGCGCTGGTGGTGGTGGTGGTGGTAAAGGGAAGAAGAAAGGGTTGCCGGCGAAGAATTTGATGGCGGAGAGACGACGGAGGAAGAAGCTGAATGACAGGCTTTATATGCTTAGGTCAGTGGTGCCTAAAATAAGCAAGATGGATAGAGCTTCAATTCTTGGAGATGCCATTGAGTATTTGAAGGAGCTTCTTCAGAAGATCAATGATCTTAACCAGGAACTTGAAGCAACCCCGGCAAGCTCATCACAGACACCGACAGCAGCTGCCACAATTGCGGCTGGCCCCCCTGGGTTTTACCCTCTTACCCCGACTCCCACCAGCCTCCCTACGAGGATTAAGGAAGAAGTTTGCGCCACCATAATGCCTAGCCCAACAGGCCAACAACCTGCAAGAGTTGAAGTGAGGCAGAGCGAAGGAAGAGCGGTGAATATACATATGTTTTGCAGTAGGAGGCCCGGTTTGCTGTTGTCGACCATGAGGGCTCTTGATAATCTTGGTTTGGACATCCAACAAGCTGTTATAAGTTGCTTCAATGGTTTCGCTCTTGATATTTTTCGAGCTGAGCAATGCAAAGAAGGTCAAGATGTTCATCCGGATCAGGTTAAGGCAGTGTTGCTTGAATCTGCAGGTTACCATGGTGTCACTTGA

AabHLH66

ATGCACCAACCAAACAGTCAACAAGGTTTGTTGGAAGATCTAATGGCTCCAACCTCAGAAGCATGGTCAACATTTTCAAATTTACAAGAACTTAATACTTTTGACACTCAATTATTTCCAACTCAACTAGATGATGACTTACTAATGTTACCACCTTCTTCTTCATCATACTCAAACTCATCATTTTTAGAACTCCTTTCTTCATCCATACAGCCAAGTTTTCAGTCCCCTTATACCGATGAATCTATCTACTCTTCTACGCCATGTTTTGATAGTTTCTTGCCGGTTAATGGTTCGGTTTCTTCATCATATGATCAACAAATGTTTCCATTTATGGCGGAAGGAGAAGACAATGATGGTGATGAAGATCATAAGGATGTTGTTGTTTCTCATGATCAACTTCCAACTGTTTTTAACATGGGAGCGAATGGAGAAAAGAAGAGTAAATCGAAGAAAGTTGAAGGACAGCCTTCTAAGAACTTGATGGCCGAAAGAAGGAGGCGAAAAAGGTTAAATGATAGGCTTTCTATGCTTAGATCAATTGTTCCCAAGATAAGCAAGATGGATAGAACGTCAATACTTGGGGATACAATAGATTACATGAAAGAGCTTCTGGAAAAGATTCAAGATTTGAAAGAACAAGAAGCTGAATCTGATGTGGATCAATTAAAATTGGAGGGAAGTTTTCGAGAATTCAACATGAATGAATCACAAGTTATAAGGAATCCCCCTAAGTTTGATGTAGAGAGAGGAAATATCAATACTAGGATCCAGGTTTCTTGTTCAACGAAGCCCGGTTTATTGTTATCGACTATCGACACACTTGAAGCACTAGGCCTTGACATTCAACAATGTGTCATAAGTAGTTTTAATGATTTTTCCCTTCAAGCTTCTTGTTCTGAGGCACAGGATCATCGATCAATGATAAGTTGTGAAGAGATGAAGCAAATCCTATTTCGCAATGCAGGTTATGGAGGCAGATGCTTGTAA

AabHLH67

ATGGAGTTCCACCATGGATTCTTGGAAGAAATATATAATTCAAACTTTCCATTTGGATTAACCAAAGACATAGATGATCACTTTGATCAAAACACTCTCTTTTCTACCCCAAGCAATTTTCTTGAAACTTCACCTCTTTCAATCACCTCTCAAAACTCTTGCTCCAATTTTGAAGACTTTACTCTACCATGCAATTTTGACCCACAAACCCTAAATTCTTCTTCTTCATATGGTCAACATTTCATGAATCCTTTCATTGACTACACTCCAGACATTAAGCTTGAATCTTCACTATTGAATGATCAAGATTATTATAATGTGTTTTCAATGTTGGATGATGTACAAAACTGTCATTTTGTGCATGATATGAGCAATGTACCCAACCCTGAAATGCCCGGCCTACAAGGGGTTACAGTTGAATCTGAACTCCAGAGGCCGGGCAGTTTTAACATTGGAAAGAATGGTAAAAATAGTAAAGTTGAAGGACAACCTTCAAAAAATCTAATGGCGGAGAGAAGGAGAAGGAAGAGGTTAAATGATAGGCTTTCCATGCTTAGATCCATTGTGCCAAAGATCAGCAAGATGGATAGAACATCAATACTTGGAGACACAATAGACTACATGAAGGAGTTGATTGAGAAAATTAAACACATGCAAGAAGAAATGGCAATTTCCTCCAATGATCAATTAAACTCATTGAATGCAAAACCAAAAGAAACATATATCAGAAACTCACCAAAGTTTGATGTGGAGAGGAGAAATACCGATACTCAGGTTCAAGTAAGTTGTACAGGTAAACCCGAGCTATTGATATCAACGATGACAACACTTGAAACGTTTGGCCTAGAGATTCATCAATGTGTTATAAGTTGCTTCAATGATTTTGCGATGAAAGCTTCTTGTTCTGAGGAAATGGAGCATGGATTAATTATAGATTCGGATGATATAAAGCAAGCGTTATTTAGGAACGCAGGATACGGTGGAAAGTGTCTCTAA

AabHLH68

ATGGAGTCTTATGGAAATGTTTTTAATGAAGAATGGATGAATCTAAGCTCAATGTTCTCTTGTGATCAGAATTCGGATAATTTCATGGGCCATGAATTGTTCTCGAATGAGTATGAACATGGTTTAAATCCTTCTATTCTTTGGCAAAGTTCCAATGAATATAATAGCTCTAATTCTAGTGTCGTTGATGATCAGATTAATTTAGCCTATCCTTCTGATCATGATCTTAAATCTAATTTCCATAATTATTTTTCTCAAGAAAGTAGCAACTCTACAGACTCTCCTCCCAACCCATCCCATGATATTTTCCAATTTTCTGCCCCTTATAATACACTTCCGGATAATGTTTCTAACCAGTCTAATGATGTATCTGTCATGGAAGGTGACTATACCAACTTTTTCTTGCTAGCTCAAGTATTTTCTGATGAAGCAATGGAAGATATACTATGTTTAAAACAAGATGAGGTAGCTGAGAGGGGTAAAAAGGAAAATTCTGCCGGTCAGCATGTGCCTAATATTGGTGATGTGGGCAGGGAAACTTTTCTCAAGAGGAAGTATGAAATGCTCGAACTTCCCATTCTATTGAAAGAAGGAAACACTGGGAGAATGGATGAGAACCCCAAGAAGAAAAGCCGGGTTTCAAGAGATAACAAAAGCACGAAAAAATCGCCACCAAAGAAGAATCAGAAGATGACCACTACTATTAATAGAAATGAAGATGATCATGGTAGCAAAGAGAATGATCAGATTAATATTAATGGAAGAGGGGATGTACAAAGTTCAAGTTCTTGCAGCTCTGAGGATGATTCAAATATCTCTCAAAAATTGCAGGAAGAGACAAATAACTCAAGTGCGAAAACAAGAGCTAGTAGAGGCGCTGCAACTGACCCCCAAAGCATCTATGCCAGGAAAAGAAGAGAAAGAATTAATGAAAGACTTAAAACTCTTCAGACCCTTGTGCCAAATGGTACAAAAGTTGACATTAGCACAATGCTTGAAGAAGCAGTTCATTATGTGAAGTTCTTGCAGCTTCAAATCAAGTTACTGAGTTCTGATGATAAGTGGATGTATGCTCCGATAGCATACAACGGGATGGACATGGGTCTCTACCAGAATATTTCACAAAACGCACCGTTCCAAAATAGATCACATTCTAGTTTCCACATACGTTTACTTGGAGAAAAAGTAGCTATACACCATACGTGGAAGTGGTTCGGTCATTACAACCTCGATGTCTTTCTTTCTAATGGTCTTGGAAAAGGAATTTAA

AabHLH69

ATGGATTTGTCGTCAATGTGGTTACCGGAGCTGGAAATGGAAGATGGTGGGTTAACGACTCAATATGATAACATGTGCAAGCCATACGATATGGTTGATAAACTAAGTGCTGATTCTATTTCCTCAGAAAACATTCTGGAAAAAGAATCTTCCATCGATAGATTTTTTCAGACTCCAAGTAGGTTTGAAGAAGCAACCGAAATAAATTTGCTTAGTTATCAAAAAGCCAGTAACATTAATAGGTCTAGTACCCCAAATACTCTTGCTGCTACTCCTCATTCCTCCTTCAACGCTTTCACCATATCTTTTGGAGATATGAAATCTAAAGAAGAGATCCCTCCATCTGATGATTCACTCGGTTATGAATCTGCTGGTACTGGAAAGGCTCCGATCGTTGCCAGGACCCCACTTCAGGCTCAAGATCATGTGTTGGCTGAGAGGAAAAGAAGAGAAAAGTTAAATCGACAATTCATTTCTATGTCTGCCCTCCTTCCAAACCTTAAAAAGATGGATAAGGCATCTGTATTGGAAGATGCAACAAACCACATAAGAGAACTTCAAGATCGCGTGAAGGAACTTGAGGCATTAGCAGGCCTTAAGAGAAAGGATACTAAAGATATTCTTGTTGCTCTAAAGAGATATAGGCTTAGCAGGGATGAGGAAGACGATTCATCTTCGAATGAAACAAAATCTGGAGATCATAGTGCGGGTGTCCCTTCTGAATCATCTGCTGAGATTGAAGTGCGGATATCAGGAGGCAGTGTGCTAGTAAGAATCTATTCTCATAAAACCTATTCATTGACTGTGAAAGTGCTCAGCCAGATGCAGAGGCTTGGGATTAACATCATCAGTAGCAGCACGATGCCTTTTGCTAATACTATCACTGTTATTACCATTGTTGCACAGATTGAGGAAGATTTCGTTATGACCGCAGCAGATCTTGTGAGTAAGCTTCAACTAGCTTAG

AabHLH70

ATGGATAGTTCGTCAACATTTAGATCGATGAATTCGAATAATAATCTAATCAGACAAAGTAGCTCTCCAGCTGGATTTTTGTCTGCCTTGAATTCTGAAAATGGTTTCGCAAGCATGAGAGATGTGGATAAAAGTATTCCGTCGAGTAGGTTGAATAATCACATTAGTTTCTCATCAGATAAATCTTCGTCCTCGATGTTCTTGCCTCAAATTCCCGAAAACAGAAATAATATGAACGATTCCACATTCAGGAGCTTGAAAAGGACCAGAGACGATGATTCATTAACGTTGGACAAACAGAGTGGAGCATCTGGACAGTATACTCCTAGTTTGATTCACTATATGAGCTTACCAAAAACTTCTTCTGAGATGGCGGTAGCTGAAAAGCTCTTGCGATTTGACCAAGACTCGACTCCGTGCAAGACACGTGCAAAAAGAGGATGTGCCACACACCCACGAAGCATCGCAGAAAGGATGAGAAGAACCCGGATAAGCGATAGAATGAAGAAGCTACAAGAGCTTTTCCCTTCCATAGACAAGCAAACAAGCACATCCGACATGTTAGATATGGCTGTTCAGCACATTAAAGATCTCCAAAAGGATTTGCAGAATCTCAAGAATGCTCGGTCAAGGTGCACATGTTCATGTAAAGAATCATAG

AabHLH71

ATGCAAGATCAAAGCTTTATGAATCATGAGTACCAGATGATGAAACCTTATCACCTGGTACCTGGCTTCAGTACTATTGATTCATTTTGTTCAGGAAGCAACACCAATAATCCATCTTTTGTAGACCACGAGTTTCAAATTCCAAAAAGCATTAACGAAACATCCTTTATCAAAAGGCAGCCTAGTTACAAAATGACGAACAATATAGTGAAGAAACTTACCTCAGATGAACACCTAAAGCCGAAGCCAATGCCACTCTCCAGTAATACCAACACTTTCACTATATCTTTTGGCGATCTAAAACCAAAAGACGAGGTCATCACCTTATGCGATTCATTTGGTAACACACCCGGTATGAAAAAGGGGACTGTGATCAGGAATCGGATTCAGCTTCAAGATCATATGTTGGCTGAGAGAAAGAGAAGAGAAAAGCTGAGTCGAGGGTTTATTTCTTTGTCTTCCCTACTACCTGATCTAAAGAAGATGGACAAGGCAACTGTGTTAGAGGATGCAGCTAACTATATACAAGTGCTTGAGTGTCGGGTTAAGGAACTAGAGGGATTACCACACTTGAAAACAAGTGATATGGAAATGGATATGTCTGCAAAGAGACCAAAGCATAGTTTTAGTGATAACAATTGTTCCTTGTATGATGAAACAACAGTTGCAGCGAACCATATCCCTCACAACCCAGAGATTAAAGTGAGAATTTCTGGGAGCAGCGTTCTAGTACGAATCTTATGTCAGAAAGATTATGTGTGTTTAGTAAATGCAATTAGCGAGGTGGAGAAGCTAGGTCTTTCTGTCTCCAGTAGCAGTGTTTTGCCATTTGCCAATGTCGCTCTCCTTATCAACATTGTTGCGAAGCACCTTGTATGCGTTTGTGCCACATTTATGCTACAAAGATAA

AabHLH72

ATGGATATGTCACAAGGATGGCTAGAAGAACTGGAAATGGAGGATCCAGGATACATGAGCTGCGATGAGATGAACATACTCAACTACGATATTGATAACTTCAGTCTTGATTCATTTTACTCAGATATCTATACAGAAAAGACGAAGAATATTAATCAAATTTCTCAGACTCGCCAATCTGAAGTAAATAATCCTAACCATCAAGAAAAGAGTAGTAGTACAATTAACAAAATCTCCACAACTCCAGAACCCCTTATTCCTACCAATCTTCCCTCATCCAAAACTTTTACCATATCGTTTGGAGATCTAAAACCGAAAGATGAGCCTTTCCAGTTTCATGACTTGCCTGGGTATGAAGCTGCCCATACCACAAAGGTTTCAGTGGCTCTCAGGAACCCGATTCAGGCACAGGATCATGTTTTGGCAGAAAGGAAGAGAAGAGAAAAGTTGAACAAGAACTTCATTACTTTGTCCAACGTACTTCCTAAGCTTACGAAGATGGATAAGGCATCTATGTTGGAAGATGCATCAAATTACATAAAAGAACTTGAAGGTCGCGTGAAGGAACTCGAAGGAACACTAGAAAATAATAAAAGAGAGAATGTTGATCAAGAATCTGTTATTTCTGTGAAGAGATATAGACCTAGTTCTAGCAATGATGATTTGCGTGATGAACCAGCGACCTCTGGAGAGAGTACTGCACCTTGCAAGACGACTCCTGAGATCAAAGCGTGTAGATTAGGAAACACTCTGACAGTTAGTATTCAGTGCCAGATAAATCATTCTTCCTTTGTCAAAGCACTAACTCAGATGCAAAAGCTTGGACTCTCCATCATCAGCAGCAGTTCCATGCCATTTGCTAAGACCCACCTTCTTATCACCATTGTTGCTCAGGCGGTTCGTAGTGACAATAATGTGGCAGCCGTAGCTTCTACTACTTTAGGAGAAACGGATTATGGTTTCATTGAGGTAAAGAATCGTAAGGCTAAAGGTAAGAAGGTTTGGGTTCAAATGCTTCAAAGAAAGCCGTTTTGTGGTATTGATGTGGGAAAGGTGGCTCAAGAAACAATACCATTGTTGCCGGCTCTACAAACACCAGCATTATTGGTGAACCAACAAACACAAGCATCATCAGCAGTGGAGGATGCTAGAATACCTAAAGAAGAGCTAGAAAAAAGACTTGATAACTTTGAACATGTTGTTTTTAGGAAACAACCATGTGTGAGAGCTGCCTATACTCGTTGGATATCCCGTGACGATGTAACTACTAACCACATTATTGAAGTTCCTGGCGGGATGATTGCAGGGCATGAAGATAGGTTTCATGTTTCAATTTCTACCGACGACATAATGAGTTTATGGAAATTAGATGGGCTGAATAGGAACTTATGTCTTCAACCGGGTTCGGCTGTTAAAGATTACCTGACACGCACATTAAGCGTCTCGGACTATGATTTTATGCTAGCACCTTATGCTCAAGAGGGCCATTGGGTGTTGTTTGTTATATGCCCAAAACAACGCACGGGGTACATCCTGGATTCAAAAAATAGGAAGCAAACAAAGACGGAGCGCACTTACTGGTTAACAAGCCATTTGCAAGATGCTGTTGGTAGCTACACATGGACAATGGCTAAGGAAACTTCTTTCACCTGCAATCAACAACCACGCGACTCAGATTCCGGTTATTACATCATGAGATGGATGTACGACTTAGTGAACACTGATGAGCTTCACTTCCCTTCAAAAGACCGTAGAACAACTTAA

AabHLH73

ATGGAGAAAAAAGATTATGAGAGGCATTGTGATGGTGACAATTTGGGTCATATGGAAAACAAGGAATGCAAAGATGAAGATGTGCAAGTAGGTAAAGGGAAATCTAAAGCCGATAAAAAGAATTGGACGGCGATAGAAGAGGTTGCTTTGGCTAAAGCGTGGATTCATATCTCGACATGTAAAAAAGTCGGGAATGAACAAGGTAGAGATAAGATGTGGCAAAGAATTTTGGAACATTTCGCGACCACCATCACCGATACTAAGCGAACTCATCATAGCTTGAACACAAAGTGGAAAAACATGAATCACGCAATGGGTGTCTTTAACGGGCTATACATCCAACAAGCTAATGAACAAAACATGAACCATGTCATCATTTATCCATTTTACTTGTTCTGCGTGTTACCGGAAAATCACCAATTTCCGACTATGGTTTCACCGGAGACTACTAATTGGATCTACTATCCTACAAGTGTGGAACAAGATGGCTCACTTGCGGATTCAGATGGGCATAAGGAAACTGGTTCAAAAAAGCGGGGGAGGGCTGAACCTTGCAGTGGTACGAGTTCCAAAGCATGCCGAGAAAAATTGAGGAGGGATAAGCTAAACGATAAGTTTGTTGAATTGGCTTCGATCCTTGAACCTGGGAAGCCCCCTAAAATTGACAAGGCTGCTATATTGGTTGATGCTGTGCGGAAGCTTGCTCAGTTAAGAAACGAAGCCCAGAAGCTGAAAAACTCAAATACAGAAATTCAGGAGAATATTAAAGAGATGAAGAATGAGAAGACCGAATTGCGGGATGAAAAGCAGAGGCTAAAGGCTAAGAAAGAGGATCTCGAACAAAAAGTCAAATCAATGAATACCCAACCGAACTTTATGATTCCTCCACCTGGAATCCAGGCTGCATATCCTGCTGCCATTGCAGCAGCTCAAGGCCAAACAATGGGCAATAAATTTGTTCCTGTCGTCAGTTACCCGGGAATGGCAATGTGGCAGTTCATGCCACCAGCTGCTGTTGACACCTCTCAAGATCATGTGCTCCGCCCTCCGGTCGCTTAA

AabHLH74

ATGGATCCGAATTTGAACTTAATGTGGAACCAAGACGATGACGATGTGAAGGTATCTAGAGATGATGATTCCTCTGAGACGGAGACTACCCGTAATCCAGATACACAAAGGAGTGGTGGTGGCGTGAAAGTTGATCGTACAAGGACCTTGATTTCTGAGAGGAAGAGGAGAAGCGGGATGAAGGAGAAGCTTTACGCGTTGCGTGCATTGGTACCCTACATCACTAAGATGGATAAAGCTTCCATAGTTGGGGATGCAGCACGGTACATTCAGGATCTTCAAACACAAGCAAGGAATTTAAGGTCTGAGATCGCAACAATTGAAGCAACAAAAAATCAAAAATTGTCATCCCGTAATATGAATAAGAAAAATGTTCCAAACTCTCTTCCGGTATTGAAGAAGATATCAAAGATGGACATGTTTAATGTGGAAGAAAAAGGATATTATGTGAGATTAGTTTGCAACAAAGGTCGAGGTGTTGCTGTGGCCCTTCATAAAGCACTTGAGTCGATCACAAGCTTTCAGATTCAGAGCTCGAATTTGGCAACTGTCGGTGACAATTTTGTGTTGACATTCACCTTAATTGTTACGGCGTGCGAATTTGATATAAACCTACAAAATATGAAGTTATGGCTTTCTGGGGCTTTTCTTGGTCAGGGTTTCGAATTTAACACATTTCCATCACCCTAA

AabHLH75

ATGAGCGAAATCTTACAGATATTCGACCATTCTCCACCGTCGCACTATCAAATGCATCATCATGGTCACTTTCAACATGGTTTAGAAAATGTTGGGTCACAACTTAACGGGGTATGGGAATCTGAGTCTCCATTGTCCAGCATATTTAACTTTGGATGTTCTGAGGTAGCCGAAAATTGTTTCATGGGCTTTTCGGTTGATGGATTATCAAAGGTGACCACGGAAGTTGAGCAAACTCAGAGCTCGAGGAAGAGAAAACTTGAGCTTGATGAAGTAGAAGTGGACTCTAAAAAGCAAAGCGTTGAATGTGATTCGGAATTAGTTGAACCACCCAAAGCAAAGAGAAACACCCGTAATGGAAATTCAAAGCCAAAGGTGGATCCAAAGACTGATTATATTCATGTGAGGGCTCGTCGTGGACAAGCTACTGATAGCCATAGCATAGCTGAAAGAGCCAGAAGGGAGAAAATAAAAAGGAAAATGCAGTTTTTGCAAGATTTGGTTCCGGGATGCAGCAAAATCACCAATAAAGCTGCAATTCTAGATGAAATAATCACTTATGTTCAGCGCCTTCAAATGGAAGTTGAGGTTCTAACAATGGAACTCGCTGCTTCAACTACAAGTGTAGACTTGGAGATGAACAACTCATTACCCCAAGAGGCAATGAATACATTTTAA

AabHLH76

ATGAATTTTGAAATGAATAATCAGAACAATAATGGGTTGCTAAGATTCAGGTCTGCTCCTAGTTCTGTTCTTCAAAGTTATGTTAATGATATTGAGAAAAATAATAAAGATTTAAATGATGGGTTGAATTATAATTTTATTTCTAATACTTTTCAAGATCTACAAGAGGAAGTGGATCTTAAACCAAACTTAGTTAATGAGTTTCATTATCCAAGGCAAAGTACAACTCAAGGAAGCATGGATAGTAATGGTTATAGCATGGTGCAACAACCTAAAATGAGCTCAAGTCTTTTGAGACAAAATAGTTCTCCTGCTGGATTATTCACTCACATCAATCAACCTAACGGTTATGGTGGAATGAGTGGTTATAAATTGGTAAATGCTGGCAACAATGGTGAAGTAAATCCGTCTTCAAGCAGATTGAAGAGGCAAATGAGTTCCTCATCTGGTGTTAAAATGCTGCCAAGGATCGCAGAGGTCGAGCCTGACACCATGGATCCAGGAGTACTCAACGAGTCATCTGATTACCCTTTTGGTTCGTGGGAACAGGAGTCTTCACAATTCACTGACAGCTTTACTGGTCTCAAAAGAGAGCTAGATCTTCAGAATGGCAATACTCTGGGAAATCAACCACCTATGCTGGCACACCACTTGAGTTTGCCAAAAACATCAGGAGAAATGGCTGCTATTGAAAAGTTGCTGCATTGTCAAGATTCTGTCCCGTGTAAAATTCGTGCAAAACGAGGCTGTGCTACACATCCGAGAAGCATTGCCGAGAGGGTTCGAAGAACACGAATAAGTGAAAGAATGAGGAAACTTCAAGAACTAGTCCCACACATGGACAAGCAAACAAACACATCAGACATGTTGGATTTTGCTGTTGATTACATCAAAGATCTTCAAGAACAGTACAAGGCGTTGAAGGATTGTCGTGCCAATTGCAGATGTTCTGCAATGTCGGGTCTGACCTAA

AabHLH77

ATGGTTTTAGAAATCAACAATACATTCTTGCGACACAAGCAGCACAAGTGTTCTACCTTGAAGACCCTGCTAGACGACCACCTCATTGGAAGGTCGTCGAAGATGTTCATCATCGGAAGATCTGTCATCGGGATGTGTGAATTTCCTAACAATACCGTGGAAACAAGTTTTCCTCCCTGGTTCAATCACAAGATCCGTGAAAAGAGTGTGGCAAAAGACCCAAGCTGTAGTCCCGAGTTACTCTCTTTGGCATGTGGACCTTCATCTAGTGCAAGTACATACCCCGCTTGCATAGTTAACGGTGTGAAGTTTATGGTGCATGAGCGCGATATACTTCATACCACTCAAGGCAGTGGAGTTTCAACACCTGGGCTTGATGGAGATATGTATTATGGTCAACTGGAAGAAATTCTGGAGCTCACTTATATGGGCAACCGCAAGGTTGTGTTGTTCCGATGTAAATGGTTCGACACCCGTAACCCTAATAACCCTACGACACGCTCTAGACGTTCTTACAGTGAACGAGGCATACGTCATATTTTGACTGATAAGGATGGTTTTAGAAATCAACAATACATTCTTGCGACACAAGCAGCACAAGTCTTCTACCTTGAAGACCCTGCTAGACGACCACCTCATTGGAAGGTCGTCGAAGATGTTCATCATCGGAAGATCTGGCATCGGGATGTGCCCTCATTTAATTTGCCGGGCTATTGCGCAGGTAATATGGTCACGGGTGCGACATCAAATCGGGCCATAAAGAGGGCGCTTAGAGGGAATAATAATAAACCTTTGCCTATCGGCTTTGATCACGATGATCAAGGTACATTCTCACCCATAGGCACGTATGCACCGCAGCTAGCTAGTCTTATCGGGGAGCAAGTTAGGCCACTTCCCCTTGACTGCAAATGGGAAGAAATTCCAGATGTTTATAAGGCGCATATATTTCCCGCACTTCGGACCTATTTTGAGATCGAGCCTTGGTTAAATGACAATCGCCAAGTGAGGTATGAGGGCCAAGTATATAGAGTGGGGGACAGGGTATGTGAAGGGCTCAAACTTCAGATGCAGTTATATTATCGCAAAAATAAAAACAGGATCAAGAAAAAGTACTTTGATATCCATAGCACGCCCGCGGTGGCAAGGGAACATCCGCCGCCGCCGAACAAGTGGGTTAACAGAACCCGACAGGAATGGGAGCTTTTGGTCGACTGGTGGTCCGATCCGGCACGGATGGAAAGATCCGCCAAAAATGCAGAAAATCGAGCAAGGAGCAAGACACTAGCGATGAAGTCCCTTTATGATCAAGTTAAAGCCGGTACAGCCCCGTCGATGACCGAGCGAGAAATATTGCATAAAGTAGTTCCGAGTGACAGCCGGCAAAATATATCGGGCGTTGGCAGGAGGATGACGGGCAGCACTAGCAAGTCCACACATCAGCCGTTTGAACAAGATTACATTACTCGGCAGCATATGACGGAGGTGTTGAGGCGAGAGCAGCAACGGGCTGATTTGGCTGAACAACGGGCTCGCGTGGCTGAGCAAGAGGCTAGATTGGCTAGGGAAACTGCTAATGCGACTAATGCTAGAATGGGCACCTTTGAATCTTTCCTTGGGCAGTTTTTTACCTTCTACAACAACCAAGGTAATCCATTTCCCGTTCCTTTTCCCCCACCTAATTCTGTTCCCGGCCCTTTTTCCTTTCCCGGCCCTAATCCTGTTCCCGGCCCTAATCCTGTTCCCGGCCCTATTCCCTTTCCCGGCCCTCCTACTTTCCAAGCTCCCTTTCCCGACCCTAATAATGCTTTTAACAATTGTTATCGTCCCTCCTTTCCCGACTCTACTACATGCCCAGCATCTGGACAGTATACTCCTAGTTTGATTCACCATATGAGCTTACCAAAAACTTCTTCTGAGATGGCGGTAGCTGAAAAGCTCTTGCGATTTGACCAAGACTCGACTCCGTGCAAGACACGTGCAAAAAGAGGATGTGCCACACACCCACGAAGCATCGCAGAAAGGATGAGAAGAACCCGGATAAGCGATAGAATGAAGAAGCTACAAGAGCTTTTCCCTTCCATAGACAAGCAAACAAGCACATCCGACATGTTAGATATGGCTGTTCAGCACATTAAAGATCTCCAAAAGGATTTGCAGAATCTCAAGAATGCTCGGTCAAGGTGCACATGTTCATGTAAAGAATCATAG

AabHLH78

ATGGACTTCTTTTTACAATGCAGAAACCCTAATTTCCATTCTTCATCAGTAGTTGCAACAAACAACAAAAGTAGCATTACATTTCATGGAAAACAAGGGGAGAATAAGAGGAACAGAAGCAAAGACAAGATCAATGCCAAAGTGAAGCTCTCAACAGACCCACAAAGTGTTGCTGCAAGAGAAAGAAGACACAAAATCAGTGAGAAGTTCAAGATTCTAAGAAGCTTGATCCCAGGTGCTGACACCAGGAACATGGACACAGTTTCCATGCTAGAAGAGGCTATTCAATATGTCAAGTTCTTAAAATCACAAATTTGGCTTCACCAAACAATGATAAATTTTGATGATTATGATAAGGCTAATACAAATTCTCATCATCAAGATCTTCTCTCATTGTATCAACCTTATGTTGAAAATAACCACCTCTCCAGCTTGCCTCAGATAGAACATGAAATCTTACAACAACTAGGGTTTCCACATGGATCTTGCTTTAAAGTTGAAGGTGTGAGTGAAATGGAATCTTTCTATCATGATCATCATCATGTGATTTATCCATGA

AabHLH79

ATGGAGTCTGTCAGTACCCTTTTAGGAGAATGGAACTCCTTTGGCGTAAACGTTTCTGAAGAGGCATTTTTTATGTCTCAATTGCTTGAGAATTTCCCTTTTTCCAACGATTCAGAAACTAACTTACCCTTTGAAGTTCCTTCTACGTTTTGGCTGCAGCATGAACTAACCATGGGCGTTGATGAAGTTCATGAAACTTCTGTTTATTTGTCACATAACACCATTAGCAAGCCGCATTGTCTGTCACAAGATCATAATTCTTCTGATGATAGTAGCCTCCTCTTTTCCAATTCAAGTGGCACAGGCTACCCTCTAATCGATCCAATGAGGGAAGGAAGTGATAATTTGGTTCCAAAAAAATCAAAATATGACAAGTCCGTTGCAAGTTCTAGGAAACGCTCTGCTAGCATGTCAGATGTCCACGAGAACAAGGAAAAGATAAAGTGTAGAAAGATTCAAAAACTAGTTTCTGAAATCTACGAAGTTGAAACTGAGGGTGAGGTGGTTGTAGGACAAACTATGAAAGTTTATGCTTCCGATGATGACTCAAATTGGTCTCACGAGTCAAGTATTAGTCCGAGACCCGAAGCGGCACTGATTACAAACTCGAATGGCAAGACAAAAGCAAGTAGGGGGTCAGCAACTGATCCACAAAGTGTCTATGCGCGGAAGAGAAGAGAACGGATCAATGAACGGCTAAGAATACTACAAAAACTTGTTCCCAATGGTACAAAGGTTAATATAGGCACAATGCTTGAAGAGGCTGTTCAGTATGTGAAGTTCTTGCAGCTTCAAATAAAGTTGTTGAGCTCTGATGATATGTGGATGTATGCTCCCATTGCATACAATGGAATGGACATAGGGCTTGACTTTATTGTAAACCCATGCTTGGCACAAAAAAGGGGCAAAATCTAA

AabHLH80

ATGGTATCACCAGAGAACACAAATTGGATCTATGAATATGGTTTAATCGAAGATGTTTCCGCCTTCAATTGCTGGCCACCTACTTCCTTTAATAATAACAATGGTTCCTCTTCAAACCCTCCTCCTAGTGCGGAACTTGATGGCTCAATTGTGGATTCAGATGGACATAATGATTCTAGATCAAAAAAGCGGGGAAGGGCTGAATCCTGCAGTGGTACAAGTAACAAAGCTTGCCGAGAGAAAATGAGGAGGGATAAGCTGAATGAAAAGTTTGTTGAATTGGCTTCAATTCTTGAGCCCGGGAGACCCCCTAAAATTGATAAGGCTGCTATTTTGGTTGATGCTGTACGAACGGTGACTCAATTAAGAAAAGAAGCCCAGAAGCTGAAAGACTCTAGTTCAGAACTTCAAGAGAAAATCAAGGAGCTGAAGGCTGAGAAGAACGAGCTTCGTGATGAGAAACAAAGGTTGAAGATGGAAAAGGAAAAGCTTGAGCAACAAGTAAACACAATGAACGTGCAACCCACCTTCATGGCTCCTCCACCTGCAATCCCTGGTGCATATGCTGCTGCCCAAGGCCAAGCACTGGGCAACAAATTGGTTCCAGTTATCAGTTACCCAGGAATGGCCATGTGGCAATTCATGCCACCTGCCGCTGTCGATACCTCACAGGATCATGTGCTCCACCCGCCAGTTGCTTAA

AabHLH81

ATGGCGATTTCAGATTTTAAAGGCTTTTCGGAACTTATAGCAATGGAGGATCCTCCTTCATTCAATTTCCAGGATCTAGTTAACTCATATGATGATCATTTGGATCCTATGGGGATAGCGGTCAACTCGTTTGATGCTAATGTTGGTGTGTTTAACTACTATAAACCTGTTATGGAACCATCTTCAAGACCAACCAAGCAGATCAAAACAAGCAGCTGGAATTCTTCATGTGTAACAAATGAACATTCTTTGATGAATGTAAATATGTCTCAACAAGCGAATCTTGTTACACCTAAAGAAGAAACAACTGTTTCTTCCAAGATTAATGGTTTCCAATTTGGTCCGTGTATGAAATCTGGTTTTGATAATGGTTATCGTGGCGGCGTCGTTGATGGTGCTAAAAATAGCACAAAAGTCTCTCATGCTCAAGATCATATCTTAGCTGAAAGAAAAAGAAGAGAGAAACTTAGTCAGAGGTTCATCGCTCTTTCGGCTCTTGTCCCCGGTCTTAAAAAGATGGATAAAGCTTCGGTTCTTGGAGATGCAATCAAGCACATGAAAACCCTCCAAGAGAAAGTGAAGACACTTGAAGATCAGATCAAGAAAAGACCAAATACAGAATCAGTTGTATTTGTTAAACGGTACGAGGTTTTAGCTGATAATGGTGAAAGTTCTTCATCAAATGATGGCCCAATTAATGAGCAGCTTCCTGAAATCGAGGCAAGATTTTTTGGTAATGATGTTCTCATAAGAATTCATTGTGAGAAAAAGAAGGGTGTTCTTGAAAAGATTCTAGCCGAAATAGAGAAGCTTCATTTATCAGTTCTTAATAGCACTTGCATGACATTTGCCAATTATGCACTTGATATAACGGTTCTAGCTCAGCTTGATAAAGAATTTGCCATGACAATGAAGGATCTCGTGAAGAACCTACGTTCAGCTATCAAGCAATTTGTGTGA

AabHLH82

ATGCAGAAAGGTGATGATGATCAAAATGAGCACATCCCAACATCACAAGGTATATCAGAACATCATGCAGCTAGTTCACAGTTTCATGAAGCACCTTATACTTCAGTCTTTTATACCAATGATGTTACAAACTTCGCGGATATCGTCAAGGTTTTATCGCAAGTAGAGCTTCCTTCTCCATCGCCAGTCCTAGCAACATGTTGTACTTCAACCAGAGAAGATAATTTGATTACTTTCCATGAGGGTCAATCGAGTGGCACAAAGAAAGATGATGAGACTAAAGTAAATGCTGAACAACGAGAAACAGAACTAAAAGATAAGCATGATAATCTCCAAGAGCCTCATGAAACAGATTTTTTTGCTTCCCGGACGCAAAATAAAGAGCGGAGGCGAAGGAGTCAAATACACGAAAAAATACGAGTTCTCAAGGAGCTCGTTCCCAACTGCAACAAGAGAGACCAAGCATCAATTCTAGATGATACCATTGCGCATATCAAATCATTGCAAATGCAATTGCAGATGATGCAATGCATGGGAGCTGGTGCTATGTCACAAGGTCCTTACTTCACTATGAGACCAATGCACGGAATGGGATACATGAGTGACGGACACTATGGATCCTATTTTACTTCAAGTTATCCTATATTTCCACCTTTTGCAAGTGGATTCGGTCCTTCAGTGCCAACAATGGAGAATTTCGTAGGATCGCCAAGATTGCTTCAAATTCCTTATCAAGAGTTTCTATCACAGACAATTGAACCTGCTTGTTCTGGTACATTATCTTCTGACACCATTATTCCAACAGCAGGTGACCAAGTTGGATCTGGGATATCTTCTCACTATACATATCATATGCCAGTGACGAATCAAGTCTATGAAGTCCAAGACGATTAA

AabHLH83

ATGAATTCCTTTGAGTACTCAATTGGCCATTTCAATAATAGAAACTTTAACATGTTCCAACCCGATTTCATACCAGAAACCGACTTTGAAAACTTACTATCCACCATTCGATGTGAGACCACAGACCCTATTGAAAAATTCTGTCCAGACTACAATTGCCATCACTTTACAAATGCATGCACGGGAATGCAGTTGTTGCCTCAGTTGTATGGCCAGAATGAAAACCAAGAAGATGTGAAGGTTTCTGGAGACGATGATTCCTATGAAACGGTGACTACCGATAATCCAGATACACAAAGAAGGAGTGGAGGTGGCGTGAAAGGTGATCGTACAAGGACTTTGATTTCTGAGAGGAAGAGGAGAAGCGGGATGAAGGAGAAGCTTTACGCATTGCGTGCATTGGTACCCTACATCACTAAGATGGATAAAGCTTCCATAGTTGGAGATGCAGCACGGTACATTCAGGATCTTCAAACACAAGCAAGGAATTTAAGGTCTGAGATCGCAAAATTGAAGCATCAAAAAACCAACAAATGTCGAGGTGTTGCAGTTGCCCTTCATAAAGCACTTGAGTCGATCACAAGCTTTCAGGTTCAGAGCTCGAATTTGGCAACCGTTGGTGATGAATTTGTATTGATATTCACCTTGAATGATCTTGAAAAGATTTTATGCGATTCTCCCTGGAATGTGATGGGTTTTTACCTATCCATGGCACCATGGGATCCCAACAAAAGCTTTGCAGAAATTGATTTTTCGCAAGGTTCTTTTTGGGTGCAAGCACATAACCTGCCGCTGGGAAAATTAACAAAGAGTTTTGCTACCGATTTGGCCAAACGTATCGGTATCTTACTTGACATTGATTGCGAATCTTACCAAAAGGACTTGAACTTTGATCAACAAGCTGATGGACAGTCAATCCAAGGTAAACAGACTCCTTCTAGTTTGATACGTTTACCAACCATGCCTGAAAACTCAACACCTACAACACATTCAAATAATCCAAAAACAATTGTTTCGACTACCACTCAAACAATCCCCACCCAACCCCAAATCATTATAACAAATCTTGTTAAAACACATATTCCCACCAATCTTTTTGAGCCTCGATCCCTAAATCGTAAACCAAACACCGAACAGCCTGTGTATTTCATCACTTCCCCCACTGAGAGTCCGGTATGGTGGCTGCGCAAAATCAGCCACAAGACAAATGTTAATATGCTATCTTGGAATTGTCAGGGAGTTGGACGACCCCTGACAGTTTCTCACTTGCGAGAGCTTTGTCAAACTCATCGTGCAGAAGTAGTTTTCTTGATGGAAACTAAGAACAAAGAAAGAAGAATGGAATCTATTCGACGGTCGTTGCATTTTTCTGGTTACTACTACATTCACCCGGCTGGTCTATCAGGGATCTATCTGATAGGGGATCTATCTGATAAACAAGGTAGCTCAACAAATATATCTCACCAAATTGAAGAGTTTCAAGATTTTATCTCTGCATCTATGCTTTTTGACATTCCTTTCAAAGGTCTAAGCAATATGTGGGATAACAATCGCAAAGATGGTGCAAGTATTCGTGAGAGAATTGATTGTGCACTTGCTAATGATGATCTCGTTGAAGCTTTTCCGCACCACATGCTAACTCACCATCCCCTCATAGCTGAAATAAAAAGTATTCAATCTCTTCCGCCAACTTCAGAAAATTGTGCACGTCAAAACTTTCTTAAACTAAAACTCGAAGAAATATGGCTCAAAGAGGAGATGTTTTGGCATCGACGTTCACGGAATGAATGGATTGATGACCCGAAGGCCTTGAATCAACTCATCCTTAACCACTTCAAAGCTGTTTACAGTTCATCAAGGGCCCGTGACTTTTCTGATGTATTGAAACCTATTGATGTAGTCGTTTATGAGAGTATGAACTTGTCTTTAGAAGCTCCCGTTTCTGATTCTGAAATTCACAAAGCAGTTATGCAACTTGGGGCTCTAAAAGCTCCAAGAAAAGATGGTTTTCCTGGGCTCTTTTTTCAGCGATATTGGCATATTGTGAGTAACTCAGTTATCAAGGTTGTTAGACAAGTCTTTGAGAATGGTGTAATGCCTTCAAGCTTAAATAAAACTGTTATTGTCTTGGTTCCGAAAGTTCCATCCCCGGAGAAGGTCGGGTTCAAACCTTTCATTCATAAAATTATTTCTCCACAACAGTCTACGTTTATCCCAAGCCGCCTTATTCAAGATAGCAAGGTCAAAGCATCAATTTCTCAAAGTCAGCTGCCTATTTCTCTCCAAATACCCCAGAAACAAATTATGAACCCAAAAGCTCGTTATCTCTGTATGCCATCAATACATGGTCGGAACAAGAGTGAACTGTTCTCTTTCATTTTGGAACGAGTGCTCAACAAGATGCAAGGTTTTCTAAATAAACTTTTAAGTTATGTGAAACGCTTCTTTTGGGGTGGCTATGCTCATGGGAGTCACATTCATTGGGTTAGCAGGGACCACATTAGTAAACCGAAAGATGAAGGAGGCCTTGGTTTCAGAGATCTCAAAGCTTTCAATCTTGCTCTTCTCGCTAAACAAGGGTATTCGATGGTTCATCTCTTTCATAATTCTCAGCTTCTAATCCTTTGGTTCTTCATAGCTCCTCTCAAGAAAGTTCACGTTCATCTCTTTCATAATTCTCAGCTTCTAATCCTTTGGTTCTTCATAGCTCCTCTCAAGAAAGTTCACCTTAATTGTGATGGAGCATTTAAATTGAACCAAGGTGTTGTTGGTATTGCGACTCGAAATTGTGAAGGGTCTTTGCTTTTATGTCTTGGAGAAAGATGGCATGCTTCTTCAGTAATTGCTACCGAAGTTATAACATTACGTAGTGAGTGTAGTCTTGCAATGATGAAAAGGTGGCACAACGTGATCATTGAGTCTGATTCACAGCTTGCTATCTCTCTTGCTTCATCCGAGTCTGACCCTCCTTGGTCACTTGATGCTATTGTTGGCGACATTAAGGATTAG

AabHLH84

ATGGAGTCCATGAGTACCCTTTTAGGAGAGTGGAACTCCTTCAGTGGCGTAAACGTTTCTGAAGAGGCCTATTTTATGTCTCAATTGCTTGAGAATTTTCCTCGGTCAGATGAATCAGAAAATATTTCACCATTTGAAGCTCCTCCTTCGACATTTTGGCCAAGCCATGAACTAACCATGAGCCTTGATGAGGTCGAGGAAACTTCTGTTTATCTGTCAGATAACACCAATAGCAACCCGCATTGTCTGTCACAAGATCATAATTCTTCTGATGGTAGTAGCCTTATTATTCCCACTTCAAGTGGTGCAGGCTACCCCCCAATCGATTGCTTGAGGGAAGGAAGTGATAATATGGTTCCAAAACATACAAAAAATAATAAATTGTTTGCAAGTTCTAGGAAACGTTCTGCTAGCATGTCTGATGTCCACGAGAACAAGGAAATGATAAAGTTTTATGCATCCGATGATGACTCAAATTGGTCTCGGGAGTCAAGTATTAGTCCAAGAGAAAAAGAGGTAGCAATTCCAAATTCAAATGGCAAGACAAAAGCAAGTAGGGGGTCAGCAACTGATCCGCAGAGTGTCTATGCACGGAAGAGAAGAGAACGAATTAATGAACGGTTAAGAATATTACAGAAACTTGTTCCTAATGGTACAAAGGTTGATATAAGCACAATGCTTGAAGAGGCTGTTCAGTATGTGAAATTTTTGCAGCTTCAGATTAAGCTATTGAGCTCTGATGATATGTGGATGTATGCTCCGATTGCATACAACGGAATGGACATAGGGCTCGACATAACAATCCCATCACCAAGATGA

AabHLH85

ATGGATTGCCACGTCAGGAACCTGTTGACGAGGGCTATATTTGTGAAGGTATTCGTGGATGGTAAACAAGATGACGCGAAAGTTGTGAAAGTGGTGGGTGTGGTCGATGAGCAAAACAATGACGAGCGAAACGTGTTGGAAGGTAAAGGGGATTTAGGTGTTGAGAAGTGGATTGGTGCTTTTTCGTCGCAATTTGTTAGAATGGAAGATTCGAGTTTTAGTTTTCATTGGCCTGTCAACTCCATTGATGATCAACTTAGTTCAATAGTTGCAGTTGGAGAGAACATGCAACGCGTTAATGCTGGCGTCCCTTTGTTCGAGTACCATGAACCTGTTATGGAGCCATCTCCACGAGCCAGTAAGCAAATCAAAACTCACAGCATGAATCAGAATTTGATACATGGAAGTGATTTTGATATTGTGAATCGAGGTATCCTTGTGAACCCTAAAGAAGAAGCTTTTGCTTCTTCTTCTTCTTACAATAGCATACCATCGCCCAGTTCTGGCAATCAAAACTATTATGGCTTTACGGAAGGTTTTGACGGTGACGGTAGTGGTGCAAAAGTGGTACCCACAAGCAACAGAAGTAGAATTAGTCCATATCAAGATCATATATTGGCTGAGAGAAAAAGGAGAGAGATACTTAGCCAGAGGTTCATCGCTCTCTCGGCTCTACTCCCTAACCTCAGAAAGTTGGATAAGGCTTCTGTTCTTGGAGATGCAATTGAGTACATGAAAACCCTCCAAGAAAAGGTGAGAACACTTGAGGAGCAGACCCCGGAGACAAACAGGAAATCTGTAAGCTTCGAGATGGTGGGTGATGATGTTGAAAAGTCGTCATCAGATGAGAAATTGTCACGTCTTTCAAACCAACTACCTGAAATTGAAGCACGGTTTTCTGGCAAGGATGTCCTAATAAGAGTCTATTGTGTTAAAAAGGCTGGTATTGTAGAAAAAACACTAGCTGAGATAGAGAAGCTTCATGTATCTGTGATCAACAGCACGGCCATCATCTTTGCCAATTCTGCGCTTCATATCACCGTTATTGCTCAGATGGATAAGGATTTAGCCATGACAATGAAGGATGTAGTAAAGAATCTGCGGTTTAGTCTAACGCAATTCCTGTGA

AabHLH86

ATGGCTTTAGAAACTATAATATATCCAAAAGGAGAGATTGGTAATCTTGGATATGATTACTTTATGCAAGAAGAACATGCTTTTGATGAGTTTTTGGAGTGTGCTAATAGTAATATTACTCATTACCAAGAATCATCAAATAATGTCCATGCATATTGGGGTGATTACTATTCTTCACCAGAAAATTGTATTGGTGCTCATGACATGAAGGTCGAATTATCACCAACTGTTGTGGTGGCAACAAGTACGGCCACTACAACAGTTGGTGCTCGGAAAAAACGAAGGCGGACTAAGAGTGGTAAGAACAAAGAAGAGTTGGAGAATCAAAGGATGACTCATATTACAGTGGAGCGCAACCGCCGCAAACAAATGAATGAATATCTAGCCGTGATCCGGGGTCTCATGCCATCTTCATATGCTCAAAGGGGTGATCAAGCATCAATAGTAGGAGGAGCCATAAATTTTGTGAAAGAATTAGAACAACAACTACAAACTCTAGAGGCTAGAAAAAGGTCCAACAACAATAATGTTTTCTCACCATCACCACAACCCTTTAGTGATTTCTTCTCATTCCCACAATACTCTATTCGTCCCGATGCCAATGATGGTAGTTCAACCTCCACGACCAAGAGCAGACTACCCGCTATGGCGGAAATTGAGGTGACAATGGTCGAGAGCCATGCAAACATTAAGATACTTTCGAAAAAACGGCAGAGACAACTTTTGAAAATGGTTGCTGGTCTACAATGTTTATGGATCACTATCCTTCATCTTAATGTTACTACTATTGAGCAAATGGTTCTCTATACTCTTAGTGTCAAGCTTGAGGATGGGTGCCAACTGAGTACAGTAGATGAGATTGCGGATGCTGTAAATTGCTTGTTGTTCACAATTGAAGAAGAATCACTTTCATTCTCAAATATTTATATTATACCGATGGGATGGCGCGTCAACTCGTCAAGTGCACCTCTTGCATGCTTGACTCAAGATATCAAAGCATATCCAGATGAACCTCAGAGCATCCTTGAATTTTCATCAATATGCCCCTCTCGTGGTGAGCTCGTGGTCACAAGGCTCACTATTACACGAACCGGTTTTTTTCACAGATTTTTTTCCACGGAAACAAGACTGTGTGAAATTTCACATGGAATACGGATAAGTTGCACACCAAATTGTTGA

AabHLH87

ATGGAGTCTTTTGGAAGCTTTTTCGATGAAGAATGGGAAAATTTGAGCAAAATGTTTTCTTGTGATCAATATTCCGACCACGGTTTATATTCAAGTGAACAAGATCATGGTTTGAACTTTGAAATTCCTTCCTTTGTTTCAACTCTTATTACCGAAGCTAACAATGCTAACTCGTCGTTCATTGACCATAATGATTTTCACTATACTTCTGAAAATGTTAACTCTTACCATCATTACTCTCAAGAAACTAGCAACAATGCCAATGAATGTGTTGCTTATGATGGATCAGCCTCTCTTTCCTACCCATCATCTAATACTATTCCTTTACCAACAAATGGTGTGTATGAACACGAGCCCATGAATCTTTATAACGAAAATAATAACATCTCATCGTTACAAGCCCCAGTTTTTTCTGATGATTCAACGGAACCGATGGTGCCAAATGCTGACCAGCGCACACATATGATCGTCAAGAGGAAGATCGAAATGCCAGAATCGCCATTGGAAGACAAAGTCAACGAAGATAAACCAGATGAGAAACCAAAGAAGAGAGCTAGGGTCACCAAAGATAATAAAAATAAAAAGAAAGCGCAGCCAAAGAAGAAACAAAAGGTCATTGATTCTGCGTCAAATGAAAATGTGATTGATGGAGAAGATACAAACAACAACAAAGGAGGGAATGCACCAATCGCGAGTTCAAGCTCATGTAGCTCTGAAGATGATTTAAATGGTGGTGGAGATAACGTGAACTGGAAAACAAGAGCAGGTCGAGGTGCAGCCACTGATCCACAAAGCCTCTATGCAAGGAAAAGAAGAGAAAGGATCAATGAGAGATTGAAAGTACTTCAAAACTTGGTACCAAATGGGACAAAAGTTGACATTAGCACAATGCTTGAAGAAGCTGTAACATATGTGAAGTTCTTGCAACTTCAAATTAAGTTGTTGAGCTCTGATGAAATGTGGATGTATGCTCCAATTGCTTACAATGGAATGGACATGGGTCTTTATCAGAGGCTTTCACTAAACATGAGCTAA

AabHLH88

ATGGATGATAATAAATTTCATCTTGTTCCATTTGATAACAACAACAACAACAACACTATTATTCAATCAAGGCCTGGACCAGAACCGAAAGACAGAAAGCTTCAAAAAGCTGACAGAGAGAAACAAAGAAGAGATCGTCTAAATGAGCAATTTACCGAATTGGGAAAGACACTTGATCCTGAAAGACCAAAATTTGACAAGGCAACTATACTTGGTGATACAATCCAGATGCTAAATGATTTGACTGCTCAAGTCAGCAGACTAAAGTCTGAGTATACGACACTCACTGAAGAATCTCGCGAGTTGACTCAAGAGAAGCATGATTTACGAGAAGAAAAAGCATCCCTTAAATCAGATATTGAGAGTCTTAATCTTCAATACCAGCAAAGGGTTAGGGCTATGTACCCATGGGGTCATATGGACCAATCAGTTGTAATGCACCCCACTTCATACCCGTACCCGGTCCCAATGCAAATGCCGATGCCACCTGGATCTATTCCAATGCATCCATCCATACAACCTTACCCCTTTTTTGGAAGCCAAAATCCCGGGGTTGTTTCCAATCCTGGTTCCGCTTTCTTTCCACCTGTTCAATACGTATCTCCAGTTGTGCAGACAAGTACTAGGTCACAGGTCTCTAGCAGACAAGGTTCTAGAAACAAATCATCAGAGCAAGGGGAGAACGGAAGTGGAAAAGATGGGGATTCTAATGATGTTGCAACCGAATTAGAGCTCAAGACACCCGGATCTACAGGAGATCAGGATGGTTCTCCATGTCATTCAAAACCCAAAAAGTTACATAGGAAGGAGACTAGCTTTTCAGATGAGAACTCTGCAAGTGGGTGTGCGTCATCAGGTACTGTACAGGCTAGCTCTTCAACTAGTGTCATTGGCGGTGCAAATGGTGATCACCAAAGATCAGCATCACCACCCTGA

AabHLH89

ATGGATCCACCAATAATCAATGAGAGTTCATTTTCAACAGCTAACCCATCTTCTTACAGCTTAACTGAAATATGGCCGTTTAATGAGGGTGGTTTGGGGTTGAAGATTGGTGGTGGGTTTGGAGATTCCGGTGAGTCGTCTGTGACGGAGCAGAGTGGTAATGGTGGGAGGAATAAAAAAAGAGATGGGAATTATGAAGATGAGACTTCTTTACTTGTTTCTACTAGCAGTGCTAATGATTTGAGCAACTTGAATGGGAAAAGAATGAAGATATCAGGATTGAAAGACGAATCAGGTGGTTTGAAAGTTGACGGAGAAGGGAGTTCAGGATCGGGTAGTAAGGCTGCGGCAGCCGAGCAAAGCAGCAAACAGTCTGAACCACCTAAAGACTACATTCATGTTAGAGCTAGAAGGGGTCAAGCTACTGATAGCCATAGTCTTGCTGAGAGAGCTAGGAGAGAAAAGATCAGTGAAAGGATGAAAATTCTTCAAGATTTGGTCCCGGGGTGTAACAAGGTTATAGGAAAGGCACTTGTACTTGATGAAATAATCAACTACATCCAATCACTACAACATCAAGTCGAGTTCTTGTCAATGAAGCTTGAAGCTGTAAATACAAGAATGAACTCTCCACATTGA

AabHLH90

ATGGGAAAGGTATCGAACACGGTTCATGATCAAGATCATGTCTCGGATGAGAGAGACAGAAGAGAAAAGTTAGCCCAACAGTTCATTTCTTTGTCAACGCTACTTCCTAATCCCACCAAGACGGATATGGCATCTGTGTTGGAAGATGCATGTAACTACATTAAAGAACTTGAGAGTCATGTGAAGGAACTGGAAGAATCATCAGCTGGCTGCAACTCAAAGGATGTTCGAGAATCTAATGACGACGAAGCTAGTTCTTCTCACAAGACAAAGCGTGGAGAGGATATTGAAGTGCAAATGTCAGGGAAGAGTGTTTTAGTACAATTTCAATGCAAGAGAGATTCATCTTCCTATGTCAACGTATTAGGGGAGATGCTCAATATCGGTCTGTCAATTATCAGTACCAACGCTGTGTCTTTCACGAGTACCACTCTTTTTATCAACGTCGTTGCTGAGATGGCGGATGACTTCTGCATGACGCCAGCAGACCTATCGAAGAATCTGCAACAAGTTCTTTAG

AabHLH91

ATGTTGCCCTATTTCCAACATAACAGTTGTTCAGAGGTCTCTAATTTCCCATTTCCGTCAACCCTCTATCAACAAGATCTTCCTAATCCTGATAATCAAAAACCGGCTGGCCATAAGAGATCATGCTATAATTTGCAACCTCAGAAAGATCACCTCGTTATCATGGATAACGGCGTAGACAAGGAAAAGAAGAAGAAAAAGATGGTCCATCGAGAGACCGAAAAGAAAAGACGACAAGAGATGTCTAATCTATCCTCGTCTCTTCGATCACTCCTTCCTCTTCACTTCATTAAGGGTACACGGTCAATGTGTGAACACGTGAACCAGGCTGTGAATTACATAAAACATTTGGAAGAAAAGACCAAAGAACTCAGTATAAAAAGAGATAAACTCAAGAAAATGTGCAATGTAGTTACGGATGCAGAAGTTGTACTAAAGAAGAATGATAAGAACGAAAGGGTAATTGTCGAATGTAGTCAGAATCCTATTACAGTGACAGTCAGTTGTAGCGAAGGTGGAATTGAGATTTTGGTCAAGTCTTTTATTAATGAAAACAAGGGTCTTCAAATTTCTAGAGTGTTGAAAACACTTGTTCATGAAGGGATTGACGTTATAAGCTGCAATTCAACCAAAATCAATGACAGCTTGTTAATCTTTACCATCCATTCTAAGGTTATTGATGAGAAGACAAGCATTCAAGTGTCCATGTTACAGCAAAAGTTGGCAGAATTAGTTAATAGTGCTTCATGA

AabHLH92

ATGGATCTCGACAAACTTCCCAACCCATTCTCGCTCTCGTTCTCAAGCACACCACCACCGCAATTCAGAACCTCGTTTAAACCAGATTCAATGGAAGCCATGAGGGAAATGATATTCCAAATGGCAGCAATGCAACCAGTTCAGATTGATCCAGAAGCCGTTAGGCCACCAAAAAGACGAAATGTTAAGATATCGAAAGATCCACAAAGTGTGGCTGCAAGGCATAGGAGGGAAAGGATTAGTGAGAGGATACGGATACTTCAAAGATTAGTTCCTGGTGGTACGAAAATGGACACGGCTTCGATGTTGGAAGAGGCTGTTCATTATATGAAATACTTGAAAAAACAAGTGCAATCTTTAGAACAAGGTGCTGCTGGTGGTATGGCAATGGTGCCAATGAACAAAGAGGTGAACGTTGGTGGTGGTTATTTGTTGCACAACCAGTATGAAATGTCGTGGTAA

AabHLH93

ATGGATCCTCCAATTATCAATGAAACATCATTCTCAACAGCTAATCCTTCATCTTATACTTTAGCTGGAATCTGGCCCTTTAGTTCTGTTAGAATGGGTGGCAGAAATGTTAGTACCGGGTATGATGAGTCCATGGTGACAGAGTTTGGTGCTGGTGGTCGGAGAAAGAGGGAGGTGAATTTTGAAGATGAGTGTTCCAAGATTGTTTGTACTAGTAGTGGCAATGATTTGAACAAAGTGAATGGTAAAAGAATGAAGATATCAGGATCCAAAGACGAATTGGGTGGTTCCAAAATAGATCAAGAAGGGAATTCCGGGTCGGGTAGCAAGGCCGCGGTTGGTCAAACCCATAAACAACCAGAACCACCTAAAGACTACATTCATGTCAGAGCGAGAAGAGGTCAAGCTACCGATAGCCATAGTCTCGCTGAAAGAGCTAGGAGAGAAAAGATCAGTGAAAGAATGAAAATATTGCAAGATTTGGTCCCAGGATGCAACAAGGTTATAGGAAAAGCACTTGTTCTCGATGAAATAATTAATTACATCCAATCATTGCAACATCAAGTCGAGTTCTTGTCTATGAAACTTGAAGCTGTAAATACAAGGATGAACTCGCCGGTAGAAGGGTTTCCTACCAAAGATCTTGGTCCACCACCCTTTGATGCAGCTGGACTCTTATTTGGCTCACAAGTAGCAAGACAGTTCGCACACGGATCCCAACCCGAATGGTTACATATGCAGTTAGATCCGCGGATTTATGTCATGGGGGCCAAAATAATCTGCTAA

AabHLH94

ATGTCTTCGGGCATATCCGCGAATAGCGGTGGAGATGGGAACTTAGTGGTGAAAAAGTACGATCATAACGCAGGTGAAAGAGATCGTCGAAAGCGTGTTAATAACTTGTATCAATGTCTTGGTTCACTGCTACCCGTAACAGGTGATCAAAAGAAGAAACAAGTAAGCATTCCTGGGATAGTATCACGTGCCGTGAAATATTTACCGGAACTACAAAAGGAAGTTGAGGCATTAAAGCTAAAAAAGGAAAATATTCTGCCATTTTCATCACCAATCATAAATAGCAGGCAAGAGGGTCTTGCCATCAAGAATCAAAGTGGTGAAGGTGCTATAACAAAAAGAAATTCATGGTTAGTTTCTTCCGTGAATGTTTTGGGTGACAAAGAGGTTGTCATCCAGCTGACTACCTTAACTGATCGTATGAGCACGAATAAGGAGAATTGCTTTTTGTCTAAGGTTCTGGAAAACTTAGAGAATGGCGAATATGGATTTGTTTTGCTTAATGCGACAACCATGAAATGTTCCGGAGAAGGGATGGTATTAAGCACTCTTCATCTTCAGGTACATATATTTATTTACTTTTATGAATGTCATGTTACATTACACACCAACAAGGTGCTAAAAAAAGCTAACTAA

AabHLH95

ATGGAGTCTTTTGGAAACTTCTTTGATGAAGAATGGAGTAGTTTGAGCACAATGTTCCACGGCGACGACGCAGGTTCTGATCTCTTTGCCAGCCAAGGGTTAATGTCAAGTCAATATGATATTGGTGTAAATATTGAAATTCCTAACTTGTTTATGCATTCTAGTGATGAATCTAGCAATTCTGACTCGTTCGTTGTTGATGACGAGCATTTAGTCAATGTTTCCAATAATGTTAACCCTAATTTCTATCAGTTTTTTGCTCAAGAAAACATTTGTAGTAGTCGTGCTAGTAATGACACGGCCTCCCTTTCATACCCGTGTCATGAGAGCTTCCCGCTATACCATTCTAATATTGTTCCTTCACCATCAAACGATGTGTGTGATCAGTCCAACGAATTTTGTATGATGGACGAGATCAACAATTTGTCGCTACCAATCCAAGTTTTTTCTGATGGAAGTCTCTACGTGAGACAAGGAGTAGCGACGGAGAACGTTGGAATGGAGAATTCACTTGTTCGGGACAAGGAAACCCCTCTCAAGAGGAAGCATGAAGTGGCTGATGCAATTGATGATGAGGTCAACAATGAGAAAACCAATAAGAATCCCAAGAAAAGAATTCGAGTTTCAAGAGAGAATAAAAATAAGAAGAATGTGCAACCAAAGAAGAACCAAAAGATAGATGAGACAGAAGTTGGGAACAACAACAATAACGGGGAAAGGAATAATGGACAAAGTTCAAGCTCTTGCAGCTCCAATGATGAACTAAATGAATCTCAAGACGTGGATGCAACAAACCCGAACGGAAAAACAAGAGCAAGTCGAGGCACTGCCACTGATCCCCAAAGTCTCTACGCAAGGAAAAGAAGAGAGAGGATCAATGAGAGACTAAGAATCCTACAAAACCTTGTACCGAATGGTACTAAGGTTGACATTAGCACAATGCTTGAAGAAGCCGTAGAATATGTGAAGTTCTTGAAGCTTCAAATTCAGGTAGTAGCAACTAGCAAGTGCCGAGATTATTACGACGATACCGGATGCTACCGCTGCAATGACTATGGCAATGCCATAGCTTTTTCCATTCTTTTCTTCTACTATTATCTTAAGGGGTACCACCTCAACCTCTGGAGGCGATTGGGTTTAATTGAATTGGGTTTTAATCTTTTTGAGCTAAAGTATGATTATGATAATAATTTAATTCTGGATGCAAAAAAAGCAGCGCCATCAGGTGTATCAGGTTTGGATACAAATGGTGGAAAGCCAAATGTTTTTTCACAAGGGTGTCGATGTGTGACGCAGCAGTATCTCAGCCGATTCAAGCGTTTGTGCCGACTATATCCGCCCATCATTTATAGGCAGGTCGCTATCAAGAAATTGCATAGTTCTCGCCAGAACTCAGCTGATACATTGTGTCTGCAGACTACCACGGGGACCGGTTATTCTGGATTTCCAGTCGGAAACATTCGTCACTTTGACCATGTTACCAGTGGTGCGTTACATGAACTACCACGAGCACCAGCTATTCTTGATTTACAGTCACAAATCATTCATCATCTCAAAGTTGTTTCTTATTCACCAAATAGTACCGGAGGTGTTGATAACCCCGTAGCTGGCGTTCTTTGGTTTGACCACGTCATCAACGGTGTCTTTCCTGGTGAGTGTTTCCCACTCCTTCAGCAATATGACGACTGCTTGACTAATACAAATATTATGCTCCTCATTACTGTAAACACTATACTTATTGGCGGCTATAATGTTGTTGATTTATTGCACGCGCAGAAACTGAAACCCATGGTGGTTCGGAGCAACAGACGAAACATGCAACAGGCTCTTCACATGGATGACAGCGATGGACGCAGAAGTAGGAATAGTTTGCCGAGTGTAACTAGCAGAAAAGAGCGACTTGAAGCCCGCCATCCACCACCACCATCATGCAAACAACCCACCACCACCAAAAAACAGCTCGCCATCCACCATTATAAATGTTCGCCATCCACCACCACCATCATAAACAGCCCGCCATCCATCACCATAACTGCTCGTTATCCACCACTACCATCATGCAAACAACCCACCACCACCATAAAAAACAGCCCATCATCTCCACTACCATAG

AabHLH96

ATGGATCCACCGGTGATGATGAACGGAGTTGGTGGCGGCGGCGGTGGTTTCCGGTCAGGTAACATGTGTAATTTGGCTGAGATCTGGCCTTTTCAGATGAACTTGACCGGTAATGGTGGTGGTGGTGCTTACGTGGAAAATGATGATCCTATGGTGGTTGATAATAGTAATAATGGTAATAGTAATAGTAAGAAAAGACGTGATGATGATGAGTTGTCTAAAGGTGTTTCTACTAGTAGCAATAGCATGCTTGATAGTGACGGTAAACGCTTAAAGACGTTGGCTCTTGAAAACGAAAACTCGGAATCGAAACCGGAAGGTGAAAGAAGTTCTGGAAAAAAAGCGGAAAAACCGGCTGAGCCGTCTAAACAAGATTATATTCATGTTCGAGCACGAAGAGGTCAGGCAACTGATAGCCATAGTCTAGCGGAAAGGGCTAGAAGAGAGAAGATAAGTGAACGGATGAAAATTCTACAAGATCTCGTTCCTGGTTGTAATAAGGTAATCGGAAAAGCCCTAGTCCTTGATGAGATAATCAATTACATCCAATCGCTACAGCAACAGGTCGAGTTCCTATCGATGAAGCTTGAAGCTGTCACTTCAAGATCACAACCAAGCCCCCAAGGATATCCTTCAAAAGATTATGGTCAACAAACATTCGAAATGGCTGGGGTGCCGTTTGGCTCACAACCCCCAAGAGAATTCAGCAGAGGATCGTCACCAGAATGGTTGCACATGCAAATAGGTGGCAGCTTTGAAAGAACAACATAA

AabHLH97

ATGGAACCTATCTCTACCCTTTTTGGAGAAGAAAGGAACTTCTTTAGTGGTATAAACTCGAATGAAGAGGCTGATTTTATGTCCAATTTGTTTAGCAACTTTTCAACCAAAGTATCAAATGTTTCCACATATCAAGACGCTTCTGCATTTTGGCCACACCATGAACAAGCAATGAACACCGATGAGGCCAATGAAGTTTCAGTATCGATCTCAGATAATACGCATGCTACCATGCCTCTTCTATTTCAAGAAGATAGTTATCCGGAGAGTAATAGTATTTTTTTTCCGACTTCAAGTGGTGAAAGCAGCTACCTCTCAGTCTCATGCTCCAGGAGCATCCGAAAGGGCAATTCTGATGCTCCTTCAGAATATAATAATTTGTCGCCAAATACTAGGAAACATTCTTGTGATGAAGCAGATGTCCATCACACGAGCTGGAAAAAGCCAAAGATCAGTAGGAGTCAGAGTACAAATACGCTTGACTCGGATAATGACTCAAAAAATGTTGCAATGCTGAACACAAATGGCAAGAAAAGAGCGAGTAGTGGGTCAGCAGTCGATAGTCAAAGTGCATACGCAAAGAAAAGAAGAGAAAAAATCAATGAAAGGTTAAGGATACTACAAAACCTTGTTCCTAATGGTACAAAGGTTGATATAAGCACAATGCTTGAAGAAGCTGTTCAATATGTGAAGTTTTTGAAGCTCCAAATAAAGCTGTTGAGCTCTGATGACATGTGGATGTATGCTCCTATTGCATACAACGGGATGGACCTTGGGCTTGACGACGTGAAGCTCCCTTCCCCAAGATGA

AabHLH98

ATGAAGATGGCCATGGATTGGTCGTCGTCAATGTGGTTACCGGAACTGGAAATGGATGATGGAGGATTCATGAACCAATATGCTACGATGAGCAAACCATACGACACAACTGATAGCTTAAGTTACGGTTCTTTTAACGCTGAGAGCTACACCGGAAATGGATGTTTTATTGATAGATGTTATCAAAATCCATCTAGATTTGAAGAAAAAACTGAAATGATACTGCCTGGTTATCAAAAGGCTAGTAACATTAGCACAAAGACCCAAACTCCAAAGACTCCGGGTGCTACTATATCCTCCTCCAACACTTTCACTATATCTTTTGGGGATCTAAAGCCTGAAGACAAGATCATGCCTCCGTTTGATTATTCGATCTGTTATGAAGATGCTGGTACTGGAAAGGTTCCAACCGTAGCAAGGACTCCGGTTCAAGTTCAAGATCATGTGTTGGCTGAGCGGAAGAGAAGAGAAAAGTTGAATCGACAATTTATTTCCATGTCTGCCCTCCTTCCTAACCTTAAAAAGATGGATAAGATATCTGTGTTGGAAGAGGCAACAAATCACATAATAGAACTACATGATCGTGTCAAGAAACTCCAGGGATTATCGGTCGTTGAGCAGAAGGATGCTAAAGAATATACCATAGCATTGAAGAGGTCTAGGCCTAGCGATGATGATAACGAAGACTCCTCATACCAAGGAATAAACTTTGAAGATGATAGTGCGGATGTCCGTAGCAAATCATCTGCTGAGATCGAAGTGCGTATCTCAGGAGGCAGTGTGCTAGTGAGAATCTATTCGCAGAAAGTCCCTTCTTTGCTGGGGAAAATGCTCAGAAAAATGCAGGAGCTTGGGCTGTCCGTCATCAGTAGCAGCTCCATGCCGGTTGCTAATACTACTACTCTTATCATTATTCTTGCTCAGATCGAGGATGACTTCCTTATGACAGCAACAGACCTTGTGAATAACCTACAGCTAGCTCTATAA

AabHLH99

ATGTATCCATCTTCCAATTCCTCAACATCTAGAGATCCCAACAATATTAACAACAACACCAATGGCGATACTAACGTTAACCAACAAGGAATAGGTCTTGCTAGATACCGGTCTGCACCGGTATCTTTTTTAACAACAACCGTAGACTCTGTCATTAACGGACAAAGTCAACAACAAAGTACGGTTGGAAATCACATGTCTGGTGGTGGTGGGACCCCTATTAGGTTCTTCTCACCACCAGACACGACGTCGTCTCAACTATCCTCGGTATCTAATAATACTAATACTGGGGATAGGGTGCAGACTGGGACGTCATTTCGTTTAAATGAGTTTGCAACCGCTTTTAATGGTATGAAAAGTACGAGTAGTCAGACTCAAAATCCTTCGCCTTTGTTTCGACATGGTAGCTCACCAGCTGGTTTTCTCAATACTCTTGTTTCATCTACGCCAACTGATGGAAGGGGCTCGAGATTAGGTTCTCAACTCAGTTTCACAGGAACAAACTCTTATTCTCGACTATCTGAAGAACCTGATATTGGCAATTCCCTTATGTTTTCCTCTTCCTCATCGCATAACAAAAGAGCAAAGATTGACGTTAATGGTCTTAATATCATGGAATCTGAGCTCAATTTTGGGTTGTCAGAATCTGCTTTAGAGGCAGCCGCAATGGAGAAGATAATGGACCTCCCTCATGACTCCGTCCCTTGTAAAATTCGTGCCAAACGTGGTTGTGCAACTCATCCTCGAAGCATTGCAGAAAGGAATTCATCAATTCCTTATAAATCATCAATTCCTTTAATTTTAGATCATGCAGAAAAGGAAAGAAGAACAAGGATAAGTGGGAAGTTGAAAAAGTTGCAAGATCTTGTTCCGAATATGGATAAGCAAACAAGCTACTCAGACATGTTGGACCTTGCAGTACAACACATAAAAGGCCTTCAAACTCATGTTCAGAATAACTGCTTGGAGTATCTGGGTGCAAGTGATCTGCAGAGCATGATCACCCTTGAATCATACATTACTACTTCTTCCGCTGTTTTGTCAAAATTACCTCCAGAATTCAACAACTTCACCATCCCAACGAACATCATACATCGCTTATCAGGAAGATTTTCAAGATGGACACTTGGCTGCTAA

AabHLH100

ATGAATATGAGTGGTTTGTTTCCTTCGGCTCATCATCAGTTTGAAAATAATATGCACCCGTCTCAACAACACTCTTTGCTACATACTGTTCCTCAGGCTTTTCAAGGGCAACCAACTACCAGTACAGCTGTCACAGTTGCCCATCCACCTAGTATTCGTCCTAGGGTACGGGCTCGGCGAGGACAAGCCACAGATCCTCATAGCATAGCTGAGCGTCTGCGTCGAGAAAGAATTGCAGAAAGAATGAGGGCTTTACAGGAACTTGTTCCCAGTTGCAATAAGACCGACAAGGCAGCTATGCTTGATGAAATTCTTGATTACGTGAAATTCTTACGACTTCAGGTCAAGGTTCTTAGCATGAGTAGGCTTGGTGGAGCTGGTGCAGTGGCACAACTTGTATCTGATGTCCCGCTGCAATCTGTTGAGGGGGATGCGAACGAAAACGGATATAATCAGCCAGCATGGGAGAATTGGTCAAATGATGACACAGAACGTGAAGTAGCAAAACTCATGGAAGAGGATGTTGGAGCTGCAATGCAATTTCTCCAGTCAAAAGCACTATGCATCATGCCCATATCACTTGCTTCACTAATCTACCCTACCCAACAACCCGACACCAGTTCTCTCGTTAAGCCCGAACCATCTGCTCCTTCTTAG

AabHLH101

ATGGATATGTCACAGGGATGGCTAGCAGAACTGGAAATGGAGGATCCAGGATACATGAGCTATGATCAGATGAACATACTCTACGAAGATATTGATAACTTTATTCTTGATTCATTTTACTCACAGATCTATACAGAAAAGACGACGACTATCAATCAAACTTCTCAGACTCAAAAGTCTGAAGTAGATAATCCTAGCCATCAGGAAAATAGTAGTAGTACTATTAACAAAATCTCCACAACTCTAGAACCCCTTATTCCTACCAATCTTCCATCATCCAATACTTTTACCATATCGTTTGGAGATCTAAAACCGAAAGATGAGCCTTTCCAGTTTCATGACTTGCTTGGGTATAAAGCCGCCCATAACACAAAGGTTTCAATCAGTCTCAGGAACCCAATTCAGGCACAGGATCATGTGTTGGCGGAAAGGAAGAGAAGAGAAAAGTTGAACCGGAACTTCATTACTTTGTCCAACGTACTTCCTAACCTTAAGAAGGCATCTATGTTGGAAGATGCAACAAATTACATAAAAGAACTTGAAGGTCGCGTGAAGGAACTCGAAGGAACACTAGAAAATAATAAAAGAAAGAATGTTGATCAAGAATATGTTATTTCCATGAAGAGATCTAGACCTAGTTCTAGCGACGATGAATATTATTTGTGCGATGAACCAATGATCTCTGGAGAGAGTAGTGCACCTTGCAAGACAACTCCTGAGATCGAAGCGTGTATATTAGGAAACACTCTGACAGTTAGTATTCAGTGCCATAAAAATCATTCTTCCTTTATGAAAGCACTAACTCAGATGCAAAAGCTTGGACTATCCATCGTCAGCAGCAGTTCCATGCCATTTGCTAATACCCACCTTCTTATCACCATTGTTGCTCAGATTGCGGATGACTTCTCTATGACAACTACAGAACTTGTGAAGAACCTTCAACAATCATATGAATTGAAGCTAGATAATTATCTTTCCTTTCTCCAAGCATTTAAAAATCGTCAACTATTCAGTTTTCATGCCATTTACTTAGTTCAGAGGGAGGAAAGTTGA

AabHLH102

ATGGATCCTTTTGAGAATACGAATTGGGATCTGATTGATTATAATTGTTTAATTAATGATGTTGCATCTACCGATCTTTATTGGCCTGAACAAAGTCCGGCGGTCCAAGTTGACGCTTCACTTGCTAGCCTCACACCACCGGAGGATGGTGTGGAGAAAGAATGCCCCAGAAAGAGGGGCCGCAGTAACTCATGCAGCAGGGCTGAGAATAAAGCATGCCGTGAGAGGCAAAGAAGGGAAAAGTTGAATGAGAGATTTGTAGAATTGAGCTCTACTTTGGAACCCGACCGGCCTGCAACCACCGATAAGCTGGCTATTCTTGGAGATGCTATTCGAGTTCTGAATCAACTAAAATCTGAATCTCAAGAGTGCAAAGAGATGAACGAGAAACTCTTGGAAGAGATTAAAACATTGAAGGCAGAAAAGATTGAACTTCGTGAAGAAAAACTTGCATTGAAGGCCGAAAAAGCAAAAATGGAGCAGCAGGTCAAAGCCATGACCAATAGTAATCTTCCACCACCTGGATTTATGGCACCACATCCAGCTGCATATCAGGCTGGAGCAAACAAGATGCCCGTTTTCCCAGGCTACGGTTACATTCCAATGTGGCAGTATCTGCCACAAGCTACGTGTGATACATCTCATGATCACGAGCTGAGGCCGCCTGCCGCTTAA

AabHLH103

ATGTTTTCTTTAGATCAAAATGAGGATTTGGTGGATCACGATGAATCTTGTTTTATTTCTTTTCAACAACAGCAAGATCGTGTACCCAATCTTGATGAACATATTTCCAAGGTGGATGGCATGGCTATTGATAGAAGTAGTTTAAATCACCCTGAAAGCAGCACCAAGAAACTAGGCGGAAGCCGCTCACTGAATAAGCTTAATTTGGTTCGTGGTAGTGGTGATGCGGGAGGAGATGAGCAAGCGCAGAGGAAAATGCTTCATCGAGAGATCGAAAGACAACGGAGGCAAGATATGGCTAAACTTCATGCTTCGCTTAGAGAAATACTTCCTATCGAATTCGTCAAGGGAAACCGTTCGATATCAGATCATATGCATCAAGCAGTGTATTACATCAAACAAACGGAGGAAAATGTTAAACGGTTAGGTATGAGGAGAGATCAGCTCAAAAATTCTTTAGATACTGAAGGAAGCTTGATGAATCATCTCCTTAATACGGTCTCAGTCAACTATTCCAATGGGGGAGTCGAGATTTTGATCAATAGTTGCACTATAGAGGAGGGGTTCCACCTTTCACGAGTAGTAAAGGCTCTTGTTGATGAGAGTCTTAATGTTACAAGTTGCACTTCAACCAAAGTAAATGACCGGTTTCTTCACTCTATTCAATCTGAGGGAAACCGTTCGATATCAGATCATATGCATCAAGCAGTGTATTACATCAAACAAACGGAGGAAAATGTTAAACGGTTAGGTATGAGGAGAGATCAGCTCAAAAATTCTTTAGATACTGAAGGAAGCTTGATGAATCATCTCCTTAATACGGTCTCAGTCAACTATTCCAATGGGGGAGTCGAGATTTTGATCAATAGTTGCACTATAGAGGAGGGGTTCCACCTTTCACGAGTAGTAAAGGCTCTTGTTGAAGAGAGTCTTAATGTTACAAGTTGCACTTCAACCAAAGTAAATGACCGGTTTCTTCACTCTATTCAATCTGAGGCAAGTGATCTGGCATTACTTGATCTATCAATGTTGCAGCAAAAACTAGCAATTGATCAACAACATCTTATCAAAATATGGTGCGATTACAAGCTTACAGTCACAACTGAAATCTAG

AabHLH104

ATGATCTCTTTTCAGCAAAGTGGTGAGCTGGTATATCATGAGATTCCTTCTACAATATCCTTCAGAAATCAGCAAGATCTTACGGTGAATCTTCATGAACGTGTTACCATAGATGGGAATACAAATCTTGCTGGCAGCAACAAGAGGAAAAGGGGCAGAGATCACTCGTCGTATAAGGCTAATTCAGGTATTATTGGTCATGGAGTACGAGACAATAAAGATGAACATATGCTGAGAAAATTGGTTCACAAAGAGATTGAAAGGGAGCGCAGGAAAAATATAACGAAGCTTTATGCTTCACTCGGTGCTCTACTCCCTTACGAATTCATCAAGGGGAAACGTTCAATATCGGATCGCACTCTCCAGGTAGTGAACTACATCAAGCACATGCAAGGAAAGATTGAAGCTATAAGTGTCAAGAGAGATCAGCTCAAGAAACTCGTGGGCAAGAGTTTTAAAGAAAACACAATGAATAAAGTCTCGATTAGCTCCTGCAATGGGAGAATAGTTGAGATTGAGATCAATAGTTGCTCAATAGAAGATGGATTCCATCTTTCAAAAGTACTAAAAGCACTTGTTGAGGAAGGCCTTCACATTATAAACTATACTTTCACAAAAAGAAACAAAAGGTTACTCCACTCTATCCAATCTGAGGCATGTGATACAACGTTAACTGATCTTTCCAGATTGCAACAAAGATTAGTTTCCATAACCAATACCTGGCAGATTTTCACTTAG

AabHLH105

ATGGCACTAGCTAAAGAAAGAAACCCTAATGATCACTCTTCACACATGCCTGGTTTAGTCCATCAATCGTATAAATTTTATGGAGATAGTGAAAGCCCGGAAAAGAAAGGGAATTTCACTGGAAGTTCATCGAATTCTTCTTCACTTTCTAGTCCGGGTAGTGCAACAAATTCAAGTGGGCTTCTTTTCCGTGCTAGTGCAAGTCATCAACAACAGTCGCAACCAGAAGAAGGTCATTCAGTTATAAGTTTCAAGCCGGGCTACTACGATAATAATTTTGTGCAAGGAGGAAGTGCTTCGTGTTTCCTTAGCTTTGAAGAAAATGATGAACAACCGTACTCGAGTAATTTAAGCCCTGATCAGCTGATGAATCTTGGGTCCGGTTCGTTTGATAGCATACGTTTGCTAGAAACTATGAGTTGTATTCAAAGTGGTAGTATTAAAGAGAATAATCACCATGAGGAATTAGAATCATTTGCATGGCCAAATCCTTCATCTTCTGATAATTACCTTGCAACACAAGAACAACCATCTTTCCATAAGAGAACTCATTCGGGAGAGAGTGAGCAAGCATTCAAAAAACAATGTACCACCAACACTGCAACAAAGAAAACTAAGCAAAAGGCAACTCAACCCAAAGATCCGCAAAGTATTGCTGCCAAGAATCGTAGAGAACGAATCAGCGAGCGGCTAAAAGTTCTTCAAGATCTTGTTCCAAACGGTTCCAAGGTTGATTTGGTTACCATGTTAGAGAAAGCCATTAGTTACGTAAAGTTCCTACAGTTGCAAGTGAAGGTGTTGGCAACTGATGAGTTCTGGCCAGTGCAAGGTGGAAAAGCACCTGAACTTTCACAAGTGAAAGATGCAATTGATGCTATACTATCATCTTCCCAGAGAGACAGAAGTTCAAGCTCTAAATGA

AabHLH106

ATGGCAAACAGCAATCCTTCAGACGGTTCAGCTGACGACTTTTTCGAACAAATACTCGGGTTTCCTTTTGCTGGTGGTGTTGGTGGAGGTGGTGGTTCAAATGATCCAAATATGGCGGGAAATGATCAAGGTCATGTAATGGCTGGAGCAGGTGTTACTAATAATCCAAGGATGTTGCAGCTGAATTCTGGTATTGGTGTTGGTGGTGGTGGTGTTGGTGGTGGTGGAGGGTATCATTTTCCTTTAGGGTTGAGTTTGGATGCTTCTGGCAGTGGTAAAAGGTTTCGTGATGATGTCGTTGATAGTCGAGTTTCAGCTTCCGGGTTCCAGGGCCAGCCGATGCCTAACACAGTGCAAACGGCTCCAAATCCACCTGCCGTTCGTCCTACAAGGGTCAGAGCAAGACGAGGGCAAGCCACAGACCCACATAGTATTGCTGAGAGGCTTCGTCGTGAAAGAATAGCAGAGAGAATCAGAGCATTACAAGAACTAGTTCCTAGCGTCAATAAGACAGATAGAGCTGCCATGCTTGATGAAATTGTGGACTATGTCAAGTTCCTGAGGCTGCAAGTTAAGGTATTGAGCATGAGTAGATTGGGTGGAGCGAGTGCTGTGGCACCACTAGTCACTGACATCCCTATATCGTCCGTTGAGGATGAAGGTGGTGAAGGAGGTAGAAACCAGCCCGCATGGGAAAAATGGTCTAACGATGGCACAGAACGACAAGTAGCTAAGCTAATGGAAGAAAACGTTGGCGCTGCAATGCAATTTCTACAATCTAAGGCACTTTGCATCATGCCCATATCACTTGCATCAGCAATATATCACACACAACCACCCGACTCAACCTCACTTATCAAACCTGAATCCGAACCGCCTTTGTAA

AabHLH107

ATGGAACTACTAGAAAACAACATCAATAATTCACTTGGGAATTTCCAACCCAATGTTAGTCTTCCATTCATGCCTTTACTAGATGGTACCAACAACAATAATCTTAGCTTTAATATCAGTGGTGATCATCAAACTCGTTCCTTTTATAATCCAATGACTCGTCTCCCAACAACGACAGATGATATGGACAAGACAATATCAGGCGAAAGCTTGGGGAATTCAAATCTCCCCAACTTTAGCTCTGGGGTTACTGGACAGAATAAGGCTAACAGAAATTATAGCTATGGTGGTAGAAAGAGAAAGAAAAACCATGAAGCCGAAGTGGAGAAACCGCGAGAAGTTGTTCATGTAAGAGCAAGGAGAGGAGAAGCTACTGATAGTCACAGTCTAGCCGAAAGGTTAAGAAGGGAGAAAATAAATGGAAAGTTACGAAGCTTGCAAGAACTAGTTCCAGGATGTTATAAGACCTTGGGAATGTCAGTAATGTTGGACGTAACAGTCAATTATATTCGTTCTTTGCAAAATCAGATTGAGTTTCTCTCTATGAAGCTTTCAGCAGCAAGTATGTATTATGACTTCAACTCACCTGAAATGGATGCTTTGGATACAATAAAGGGAGCAAATGGGTATGAAGCCCAAGTTATGGACAGGATGGGTGCAGAAGGGTATGGAGACCTTCTACAGTTTCAACCAACATGGCCTCTTTGA

AabHLH108

ATGGAAGATTTAAGTTGGGAAAGAAACATATCATTCCCTTGGAGTAACATCAATCATCATCAACATCAAGATATCGAAGAAACCTTCATTTTCGGCTCGGAAAATATCTTCTTAAACCCAATCCAAGACCTTCGAAAACCCGATCCACCAACATGTTCTAACCCATTGCCCTCAAGTAATGGTTCAGCTAGTGAATATTGGTCTCAAACAACGATAGCTAAGGGCAAAACCGTAATTCCCATCACTACTCCAACACATGCGGATAGGGTCACCTCTCTAGAGTCACTAAACTGCTTATTTTCCGACAAAAATAGTAATGTGGACACGTCATTAGATGGCATTTCCGTTATTTTCGCGGATTACAATAATTTGTGTAATAACATTACTAATATCATTAGTGACGGTTCATCTACTTCCGTCACTAAAGATACCGACGACTGCATTGTGTCACAAAGCTCAAATGAAAGAAGACTAAAGCGACCAAGATCGGATCCGGGTCGACCCGTTTCCTCAAATATAAATTTCCGGCAAACTTCAGAATCTGACGAGACAGATTCTGAAGCAATTGCACAAATGAAGGAGATGATATACCGGGCGGCAGCTTTCCGCCCGGTAAGTTTTGCTGAAGATGAGGTCGCAGAGAAGCGTCGTAGAAAAAACGTGAAGATATCTAGCGATCCTCAAACTGTAGCTGCCCGACAACGAAGGGAGAGAATTAGCGACAGAATTCGGGCGTTGCAAAAGCTAGTTCCGGGTGGAAATAAAATGGATACAGCTTCAATGCTAGATGAAGCTGCTAATTACTTAAAGTTTTTGAGGTCACAAGTTAAAGAATTGGAACATATGGGTCAGAAATATGACTTTATGAGCTGTGGTACAACCACTAGTGCACAAAGTACAAGTCAAATTATGCAAAATATTAACCCTAATGTTACATTAGGTGTACCATTTCCCATGCAGACACATTTTTTACTGCCACATCAACAGCTTTACCCTAATCCACCACATGCATGA

AabHLH109

ATGGACATGATGACAATGATGATGGATCTTGATAAGCTTCCAGACCCATTTTCAATCCCATTTCAAGACCTTTCAGATTCATCCATCATGGATTTAAATTTCTCTAGCGAAAATTCCACCACCAACCATAATGGACCATCATCACTCATGTACCCTAGCCAAGACCATGTTGCGATACCACCATCACACTTCCCTCGTGCAAGTTGGTTGCACCAAAAGCCGGACTCAGTTGAGGCCATGCGGGAGATGATCTTCCGCATGGCAGCTCTGCAGCCGGTTCAGATAGATCCGGAAGCAGTTAAACCACCAAAGAGACGTAACGTGAAGATATCAAAAGACCCTCAAAGCGTGGCGGCTAGACATAGGCGTGAAAGGATTAGCGAAAGGATTAGGATATTGCAAAGATTAGTACCGGGTGGTACTAAGATGGATACAGCTTCAATGTTGGATGAAGCTGTTCATTACATGAAATTCTTGAAAAAACAAGTTGAGACATTGGAACAGGCGGCTGTGTCTGGTACAGCCGCGTGTTCCAACGTGACAACAACAGGGATGAATACTGTTGTCAGTTTCAGTCCCACCAACATGAACCACCATGGAGGTAATAATAATTATATGATTAGAGCATGTGGTGAGTCTGCTGGTCATATGGTGGGCTCTATGTAG

AabHLH110

ATGGACATTGACTTCCTCAAGTCCTTTTCCGATGACCAAACCGAGATGATGATGATGATGCAACTTGAAAAGCTACCTGATTTCTGCGGTATATACGGCGAAGATCATAACCCTTCAACCGAGTTCACAGATCAAGGAAGTAGCTCATCTACAAACCCTAATAACAACCCTAACATGTCTCATTTCCTCGAAGACTCGCATTTTGCGAACCCTTCGACTACCATTTGCTTTGGTTCTCCAACACAAACAATCCCAACTGCGATGCAACCTATTCACATTGACCCCGAGTCGGTTAAGCCACCAAAACGTAGGAATGTGAAGATATCGAAAGATCCACAAAGTGTCGCGGCTAGGCATCGAAGAGAGAGGATTAGTGAGAGGATAAGGATACTTCAAAGACTTGTTCCGGGTGGTACGAAGATGGATACGGCTTCTATGCTAGATGAAGCCATTCATTATGTGAAGTTTTTGAAAAATCAAGTGCAGACACTCGAGAAAGCTGGCGAAAATCGCCAGCCTTCTGGATCGGCTGGGATTGGCTTTCCTGTGCCGATGACGAGTGGTAATCACATTCACATGGGAACATCATCGAAAGGTTATCATCAGCAGTCGGGGCAACATCACTTTCTAGGTGCTTAA

AabHLH111

ATGAGTCACGAATTGCGAAATGACCCGAGTTTGCTCGAGAGACAACAAGCTAGAATGAAGTGGCAACAACAAAAACAACAAAGTTTTTTCAATGCGAGTGATCATACCATGCAAAATATGTTTAGTACTAGCATGCCACTAGCTCAAACTAATGAAACGTTTACGGGTTTGTTGAGTGGCCATAACGGGCTAGATATCGTTAAGCCGGACCCTGGATTAATGGAAGACTGGGCTGGTTTTGGTGATCATTTGAGCTATGGGTATATGAATCAAAATAGTATTATGCCATTAGGGGCAGATCAGTCATTTGTACATGGGAATTCTGTTTCAGTTTCAACTTCAGTTACAGTTTCTGTGTCACCTAAGAAAAGGAAAGCTGATAAAGGTCAAAGCCTAGAGGTAGTTACTGAAAAAGAAAAGAAGCTAAAAGGGTGTGCTGAAGAAGGTGATTCCAAAATCACACACCAAAATAGTAACAACAGTGACAAAGCAACAGGTGACAACAAGAGTAGTAACAGCAAAGGAGCTTCTACAAACACTTCATCTAAGGATAAATCTAAGGTTTCCGAGGTTCAAAAACCGGATTATATTCATGTTCGTGCACGTCGTGGTCAAGCCACTGACAGTCATAGTTTAGCTGAAAGGGTAAGAAGGGAGAAAATTAGTGAGAGAATGAAGTACCTTCAGGATTTAGTACCGGGGTGTAACAAGATCACAGGGAAAGCTGGAATGCTTGATGAAATTATTAATTATGTCCAATCCCTTCAAAAGCAAGTAGAGTTCTTGTCCATGAAGCTTGCTACTGTCAATCCGGAGCTAGATTTCAACATCGATAATGTATTCGCGAAAGAGATGTTTCAACCGTCCACAAGCGAATTTCAAGGACTCGGGTGTACTTCAGAAACACCTAATCCTGCATATTTTCAATTAAACTCATTGGATCAAGTATATTGTGGACTAGACATGGGAATTAACTCAGCAGAAATGGCATTAAGAAGAAGTCTTACTGCTCCAATGTCGATCCCTGAAACATTTATGGATTCATCTTGTTTCAATCAAATCCTACCAAGTGCTATGTGGGATGGTGATTTACAGAATCTGTGCAAAATGGAATTTGAACAAGGAACATTGATACCAATTCAATCCCATCAATATACAGGTTCAAATGAAGGAAGCAATTTGAAGATGGAAATGTGA

AabHLH112

ATGATGGAGAAGCGTGAAGAAATGTGTTACCAGTTTGTAGAAGATGAAATTGAGAGCTTGATTTCGCCGCCGGAAGGTGGGAGTTCTTTTACGGCGTTGTTGGAGCTTCCGGCGAACCAGGTGGTCAAGCTTTTAGATTCTCCGGTGGGTTTTCCGGTACCGGAAGGTGGTAACAATGATGTGAATTTGGTGAAAGAAGAACCTGTTGAGTTGCGTAATTCCGAAGATTCGGATCCGGTTGTGTCGAAATCTGTTAAAAGAAAGGAGCGTGAGAAGAAGGTTAAGTTACCGGTGAAGAAGAGTAAAAATGTGGTGAATGAGAGTGATGGTGATAAGGAGAAGTTGCCGTATGTTCATGTTAGAGCACGGCGTGGTCAAGCAACGGATAGTCATAGCTTAGCTGAAAGAGCACGAAGAGAGAAGATTAATGCTAGGATGAAGCTTCTTCAAGAGCTAGTTCCTGGTTGCAACAAGATTTCAGGTACGGCAATGGTGCTTGATGAGATTATCAATCATGTTCAATCCCTGCAACGTCAAGTGGAGTTTTTATCAATGAGATTGGCTGCTGTGCACCCTAGCATCGATATCAACATAGACAACATGTTCTCTGCAGAGAGTGGGTCCCTTATGGACTGTAATTTCCATGGCATGGTTTCACCACCATTGTCGCTTGATGGACAAATCAATGGACATAGCCAGCAGCTGTGGCAACCTGATGGACTACCTCCACAGCCGCTATGGGGTGCGGAAGAGGTCAACCCTAACTTCATCACGCCAGAAAATTCGCTTTTAAGTTACGACTCTTCTGGCAATTCAGGTGAATCCCAAATACTTAATTGGGCTGTTTGGATGTTTGTTTTGAAACTGAATATCTGA

AabHLH113

ATGGTGCTTTGTAAAATTCGGGTGTTGACTTGTATCGTAACTTTCAACGTGGGTCATGCACTTATGGCTCTCGTTCCAAGTCGTGACTCCAACAACCACAAACCCGCTGGTTTCAATACTGAACGTACACCTACTGCTCGGGATAATAAAAATACACCCAAAACTGCTCCAACACAAACCCATCCTCAATTGCCGCATTCAGTATTTGGAGTGGCTGGAATGCAATTTAGCCCATTTGGTTTTTGTCCGCCAGGTTATGTACCTAATCAGATTAGCGGCCCAATGCTACCACAGGCCCAACACCAACTTAACTCACATGGTTTTGTTAATAGTCTCATTGGTATTGGTCAACAAGCTGGAGCCCAACAGATACCACATGCACACTTAGCTACTAACAGTGTTTCTTCATCCCAACAGGCCCAACCATTGGGTCAACCCATTTACGGAAGTCAGACTATGCAACCAGGTGATAAGTGGTTTCATGAGGGTAATAGTCAAGATTACGATTCCATCGACAGTGAATATGTGGCCGCTATAGCAGCAGCCACATTCGCTGTAGATTCCTTAGAAGAAAAATCGAGTTCTCAGCACCATAGAAGAGAAAAAACTAGAGAAGAAGATTCATTGAGAACCAGAACGAGTCAGATTGATAGAGCTCTATCGCTTGCAAGACCAAGTAGGCCTAGAGATCCATATGTCAATAGGAATTTAAGCATACGTGGAAGTGGAAACACCAATGTTGATACTTGGGAGAGAAACCAATTGCTAAAGATTCAAAAACGATACGAGAAGAATAACCTTACAATTCTCGAATGGGAGAATGAGAAGAAAACAAGAGCTAAACATCGAATGGAAGAGAAAAAGGCAAGGTGTAGACACCATGAGCCGATCGAGGGCATGGTAGAAGGACAACATGTCATACAGTTGAATATAACAATTCTGATTAATGGTTCGGGTTCAAGGCTTGCTGACCATGGAGTAGCAGAAATAACATGGGAAAATGGCCAACCAGCCATGCATGGGCTAGGAAGAGCAAATGAGACACTAGAATCAATTGTTCATCAAGCTACAACATGTTACAACCAAACTCAATATCCAGAAATCGACTTACAACAAAGTCAAAGTCTTCCAAGAGCTCGCAACTTAAGCTCGAATGTTGCATCATCGAGTCGCCCGACTTACCTAAGGAAACGGCCTAGAGAATCTGTCATTATCCATGATCAATGTGTAGGAAATTTGGGTAACGCAAGTTTGCAAGAAGATAATGTTAGCAATAGTGGGACGGTTAATTCTAAAGATAATGATACTACAATGATGACATGGCCTTCATTCGACTCGCCTAATCAAAGCATGAAGAGCCAAAAAACAGATGATGATTCTGCCTGCCAATATGGATCGGAAAATCAAGAAGAAGAATGTAGGACTGAGGGTGAAACAATTCGATCTCAATCAAGTCGACGAAGCAGAGCAGCTGCTATTCATAACCAGTCCGAACGGCGACGAAGAGAAAGGATCAACCAGAAAATGAAAGCTCTACAGAAGCTTGTACCTAATGCTAATAAGATGGGAATGCCTGGAGTTATACCCCAGCCAGTTCATCATCCATTCATGGTCCCACAAACCATGCTTAGCCCAGCCCATGTAGGCGCCACGTCACAAACTATTCATAGTCGCCCATCTACCAGCACCACAGTTCCTTTCAACGATCCACACAGCACGTTTCTAGCACGACAAATGAACATGGATATGTACAACAACATGGCAGCTTTCTATCGGCAACAGGTCAACCAGGGAAAATCGATGAGCGTTGACTCATCTCAACTGGACCATGTTCGGGGAGAGTGA

AabHLH114

ATGGGTTCTTCAGAAGGTGATACTATTGCTAATAATAATGATAGTGAAATGGGTTATCAATATAGAGGTGAAATGAGTTCAGGAACCATTTTTAATAATAAGTCTTCAAGTGGGTCTGGTAATCCATTTGGGTCAAGCTGGGATCCATTAGAGAACTTTGGTGCTTACCCTCTTATGAATCAGTATCAATCTGGTGGTGAATTAGTTCCTAAAATTGGGTCTTTTGGAAGTGGAAATTTTTCAGAAATGGTGAATCCTTTTGTGCCTAATTCTTCTCCTAATAGTGGAGAAAAAAGAAGAGCTCAGTTTGATCCCAATATGAATGGTGAAAGAAATGATGGTTCTGGTGATAATACTTGTGAGAATGATGAAAAGAAACAAAGGATTGATTCAAGAAGCAAGCAAATGGGAAAACAAGTGAAAGATAATTCTGATAGTGGAGGGGCTGCTGCTAAAGATAGCTACATTCATGTGAGGGCAAAAAGAGGTCAAGCTACTAATAGTCATAGTCTTGCAGAGAGGGTTAGGAGAGAAAGAATTAGTGAGAGAATGAAATTGCTTCAAGAACTTGTTCCTGGCTGTAATAAGATCACTGGAAAAGCAGTAATGCTTGATGAGATTATCAACTATGTTCAGTCCCTTCAACAACAAGTGGAGTTTTTATCAATGAAACTAGCTACTGTGAATCCAGAAGTAAACATTGACATTGATCGCCTTGTGTTTAAAGATATTCATCATTCACGGGGCAGCAGTTCGAATCCACCTTTTGGATTTGGCCCAACATTAAGCTCGTCCCATTCTTATCCACACAGGAGCTTGCCTGGTATTTCACCTACAACTACCCCTTTGCATCCTATCCATCCTCAGCCGGTATGGGATAGCGATCTTCACAACCTTCTCCAAATGGGATTTGATGCTAACCCTGGCATTAACAATCTTGGACCAAATGCAGGACGAGCAAAGATGGATCTTTAG

AabHLH115

ATGGGTTCTTCAGAAGGTGATACTAATACTAATAATAATGATAGTGAAATGGGTTATCAATATAGAGGTGAAATGAGTTCAGGAACCATTTTTAATAATAAGTCTTCAAGTGGGTCTGGTAATCCATTTGGGTCAAGCTGGGATCCATTAGAGAACTTTGGTGCTTACCCTCTTATGAATCAGTATCAATCTGGTGGTGAATTAGTTCCTAAAATTGGGTCTTTTGGAAGTGGAAATTTTTCAGAAATGGTGAATCCTTTTGTGCCTAATTCTTCTCCTAATAGTGGAGAAAAAAGAAGAGCTCAGTTTGATCCCAATATGAATGGTGAAAGAAATGATGGTTCTGGTGATAATACTTGTGAGAATGATGAAAAGAAACAAAGGATTGATTCAAGAAGCAAGCAAATGGGAAAACAAGTGAAAGATAATTCTGATAGTGGAGGGGCTGCTGCTAAAGATAGCTACATTCATGTGAGGGCCAAAAGAGGTCAAGCTACTAATAGTCATAGTCTTGCAGAGAGGGTTAGGAGAGAAAGAATTAGTGAGAGAATGAAGTTGCTTCAAGAACTTGTTCCTGGCTGTAATAAGATCACTGGAAAAGCAGTAATGCTTGATGAGATTATCAACTATGTTCAGTCCCTTCAACAACAAGTGGAGTTTTTATCAATGAAACTAGCTACCGTGAATCCAGAAGTAAACATTGACATTGATCGCCTTGTGTTTAAAGATATTCATCATTCACGGGGCGGTAGTTCGAATCCACCTTTTGGATTTGGCCCAACATTAAGCTCGTCCCATTCTTATCCACACGGGAGCTTGCCTGTTATTTCAGCTATAACTACCCCTTTGCATCCTATCCATCCTCAGATGGATGTTGCGAAATATGATCGTTTCCACATCTACGTTAGATTTAATGGTTACTTGCGTCAAGTTAAAGAGTTACCCAAACCGGTATGGGATAACGATCTTCACAACCTTCTCCAAATGGGATTTGATGCTAACCCTGGCATTAACAATCTTGGACCAAATGGACGAGCAAAGATGGATCTTTAG

AabHLH116

ATGGGTTCTTCTGAAAATGATGATAGTGAAATGGGTTATCAATATAGAGGTGAAATGAGTTCAGGATCCATGTTTAATAACAAATCTTCAAGTGGGTCTGGTAATCTTTTTGGCCCAGGTTGGGATCCATTAGAGAACTTTGGTACTTACCCTTTAATGAATCAGTATCAATCTGGTGAATTAGTGCCTAAAATGATGGTCAATCCTAGTGCATCACCTAATAGTAATAGTAACAATAGTAGCAGGGAGAAAAGAAGATTGAACCCGAATATGAATGGTGATAATGTTAGTGAGAATGATGAAAAGAAACTTAGAGTTGATTCGAAAGGAAAACAAGTAAGTGAGGGTTCGGACCGTAAAGAGGATTATGTTCATATGAGAGCGAAAAGAGGTCAAGCTACTAATAGTCATAGTCTTGCAGAAAGGGTTAGGAGAGAAAAAATCAGTGAGAGAATGAAATTGCTTCAAGATCTTGTTCCTGGTTGCAATAAGATCACTGGAAAAGCTGTAATGCTTGATGAGATTATCAACTACGTTCAATCGCTCCAACAACAAGTCGAGTTTTTGTCAATGAAACTAGCGACGGTGAATCCAGAAGTGAACATTGACATTGATCGCCTTGTCTTTAAAGATGTTCTTCATTCACGAGGCAGCATTTCGAATCCAGCTTTTGGATTTAGCCACACATTAAACCCATCACATTCTTATTCACACGGAAGCTTGCCCGGTGTTCCAGCTACAACTGCACAATTGCATGCTATCCATCCTCAGCCTGTGTGGGATAACGATCTCCACAATCTTCTTCAAATGGGATATGATGTTAATCCTGGCACCAACAATCTTGGTCCAAATGGACGAGAAAAGATGGATTTATAG

AabHLH117

ATGAATGTAGTTTCACCAGAGATGTTAATGAGTGTATATGAAAGACAACAAGCAAGAATGAAGTTGCAACAACAACAAAGTTTTGTTAATGAGAATGAAAATGATCATTTTTCGGCTTTGCTCCAAGATATTAAACCCGTCCCGTGTATGCAAAATGGGTGGCCAGATTTAAGTAATGATCAGTTTCCATCATTGATGGTTGATGAGAAGATTAACAAAAGAAAAACCCATGAAGATCATAAGCTAGAGGTGGAATGTAAAGAAAATGGTGTTAAAGAAAAGAAGGTAAAAGTGTGTTCTCAAGAGGAATCAACAAAAACTTCATCAAAATCAAATGTTGGAGAAGATAAAAAACAAGATTTTATTCATGTACGAGCTAGGCGAGGCCAAGCTACCGATAGCCATAGTTTGGCTGAACGAGTAAGAAGGGAGAAGATCAGTGAGAGAATGAAATATTTGCAGGAATTAGTACCTGGCTGCAGCAAGATCACTGGAAAAGCAGGAATGCTTGATGAAATCATCAATTATGTTCAATCTCTTCAAAAACAAGTTGAATTTTTGTCCATGAAGCTTGCTAGTCTTCATCCAAGATTCGATTCCGACATTGACAACTTAATCACAAAAGAGATGTTTGAACTTTCAGCAGTCGGATACTCGTCTGAGATCGCTAGTTCTGCTTATTTTCAGTTAAACTCGTTACTAGAAATGGGAAACAGCCCGATTGACATGATGTTAAGAAGAAGCATGGGTGCTCCAGTTTCAATCCCTGAGACATCCACTGTTCCATCTTGTTATAACCAAATTCAGCCAAGTGTGACATGGGATGGTGATCAACAGATTTTAGATAAAATGGGATTTCAACCAACAATTCCATTTCAAGCCCATAAATTTACAGGTGCATACGAAGGAAGCAATTTAAAGATGGAGATGTAA

AabHLH118

ATGAGACGTAAAAGCTCAGTTTCTCAGCGGCCACCGGCAGCCAAAGAAAAGGAACGCCGAGACCGTATGAAGGACCTATACTCCACTCTTGCTACACTATTACAACTTCAACCTCACGAGAAAATGTCACCGCCGGATTTATTGGAACGAGCTACAGAAGAGCTGAAACAATGGAAGGAAAGAGTAGAACGATTGAAGAGTAGAAGAATGGAGCTAGAGAAAGATTCTAAGGGCGATTCAAGCAACAACATACAGAAGTTACAGCAGTTTGTTCAAGTAAGGGAAGTAGTGGACTTGCAACTAGAGGCAAATCTAAAAATCTTGGTTAATAACAAGAACGTGGCGCCTTTTGATATTTTGAGAGTTTTGGAGGAGAGTGGTACTCATGTCACGAGCTCTAATTTCTGTATCGTCAGCCACCACCTTTTTTGCACCATTCATGCAGAGGCTTCTAATGCTCGAATAGGGTTCGATGCTGAACAAATAGAGTCACGATTGTTGGAACTGGTTTACTAG

AabHLH119

ATGGTGGATGGGTTTACCCTAGGAATAAGCTTGGAGCTCCTAGACAACAATATGAACACGGAACTCGAAAACTTCAACTCAGCTGAAGTTAATCTTCATGGATTCATCCCTATGTCTGGTGACAATTTCTCGGATTATCATCACCGTAACCAAAGGTCTCATCCTCTTTCATTTATGTCTTTGCAAGATGGTACTAATGCCATTCAAAACTTCAATTTCTTTGGTGATCATCAACCTGGTTCGGTCTATGATCATATGAACCATTTCCCTATAACATTTGGCGCTCAACGTGATGGTACGGAGACGAGGGTGGAAAGTTTCATGAATTCGGATCCGCCAACATTTGGTCCTGCTCTCATCGGAGAAACTAAGGGTCGTGGTGGTAAAAAGAGAAAAAAGAACGACGTCGGAGATGAAAAACCAAGAGAAGTTGTTCATGTTAGAGCAAAGAGAGGTGAAGCAACTGATAGTCACAGTTTGGCTGAAAGGATGAGAAGAGAGAAGATAAATGAAAAATTACGGCGCTTGCAAGATCTTGTTCCAGGATGCTACAAGACGATGGGAATGTCAGTGATGTTGGATGTAATAATCAATTATGTACGATCATTGCAAAATCAAATCGAGTTTCTCTCAATGAAGCTTTCAGCAGCAAGCATGTTCTATGACTTCAACTCCCCGGAAATGGATGTTATGGAGACTATGAAGGGTACAAATGGGCACGAGGCACAAGTGATGGAGAGGATGGTTGAAGGGTATGGAGACCCTCCTTGCTTTCAATCAACGTGGTCTCTTTGA

AabHLH120

ATGGATGATTTGCAATTTTCGTGGTACAACATGATTCATGCTCATGATCATCAACAACAAACCTTCGATTTCAATTCAGATAATATTTTCTTGAATCAACTACCCCATTTATCACCTACCGATTGGACCGATCAAGCCATTGAAAATGACTTGATTTGCACTAATGGCTTCCAAGAGATCAAGAATGTGATTTCTTCCAACAATAACGCAGCTGCCTCATTGGAACGTTTGCTTTCTGGCCCAAATAGTAGCAATGATGCATCAGATGATGGTGTCTCCATTATTTTCCCGGATGGCAAGAGTTCTATATGGTGTAACAACGTTAATGTGACTAGAAATAATAACGGTATTAGTGGAGTTTCTTCTGCTGATTCTGTCACGGATGATGGTGTTGTTTCGGAAACGCTGCAACAGCTTGAAACTGATACTGAAGCGATTGCACAAATGAAGGAAATGATATATAGAGAGGCAGCCTTTCGGCCAGTGAGTTTCGCAGCTGAGACAGTGGTAGAAAAACCTAAAAGAAAAAATGTGAGAATATCAAGTGACCCTCAAACCGCGGCAGCTCGACAACGACGTGAGAGGATTAGTGAAAGAATTAGGGTTTTGCAAAAATTGGTGCCTGGTGGAAACAAGATGGATACAGCATCAATGCTTGATGAAGCTGCAAATTATCTTAAGTTCTTGCGGTCACAAGTGAAAGCCTTGGAGCAATTAGGGCATAATAGTAAAACTATTAATGCACATTCTTATGCTTCATTGGTTGCACCTTTTACTCAAACTCCATTTTCCATGCAAACCCAATATTCATTTCCTTGTGAAAATTTGTACCCTAATCCACCATCATCAGTTCATCATTAG

AabHLH121

ATGGGACTCGTGGAGGGTTCAGACGGTGGCGCCAAAGGCACGACGCTGGTAGCTGTTGGGGGTGGTATGAAGAGAAACAAGCGTACTTTGGTGACAATGAATGGCGACATGAAAAAGGAAAGTGGGGTTGAAGATAAGAAAGAAATAAAAGATTTGCAATTATGTGGTCATTCTAAACATTTGTGGACAGAACGACAAAGGAGGAAGAAGATGCAGATCATGTTTCAAGACCTTCAGGCTTTACTCCCTTGTCATTCATCACCTAAGGAACACTTGGCTACTATAGTTGATGAGGCTATAGCCTATGTCAAGACACTGGAAGAGACCATCCAAGAGCTTGAGAACCAGAAGCTAGAGAAGCTCTGTGTCAGTTCCTCAACCGCCGAATTGGATACAGCATCTCGTTCTCGGATCGCCCCATTTTGCTTCACAAGACTTTCACCGCCAGTCTTTCAAACTTGGACTTCCCCTAATGTTAGCTTAGATGTGTGCGGTGCGGATGCGTACATTACCATTTGCTCTTCCAAGAAGTCTCAACTCATTACTAGAATTTGCTTTGTGATGGAAAAGTACAAAATTAATATCATTTCTATTAAAATTTATTCGGATAAATACAGAAGCATGTTTATATTTCATGTTCAGGCAAATGCGCATGATGAGGTTTTAACGGCTTCTTGTTACGAAGAGCTATTAAAGAAAGCTGCACTTGAGATATTGTTGTTGGTTAACTCCAAATCATCGTAG

AabHLH122

ATGGATGATTTGCAATTTTCGTGGTACAACATGATTCATGCTCATGATCATCAACAACAAACCTTCGATTTCAATTCAGATAATATTTTCTTGAATCAACTACCCCATTTATCACCTACCGATTGGACCGATCAAGCCATTGAAAATGACTTGAATTGCACTAATGGCTTCCAAGAGATCAAGAATGTGATTTCTTCCAACAATAACGCAGCTGCCTCGTTGGAATGTTTGCTTTCTGGCTCAAATAGTAGCAATGATGCATCGGATGATGGCGTCTCCATCATTTTACCGGATTGCAAGAGTTCTTTATGGCGTAACAACGTTAATGCGGCTAGAAATAATAACGGTTGTGGAGTTTCTTCCGCTGATTCTGTCACGGATGATGGTGTTGTTTCGCAAACGCTGCAACAGATTCGTCAACTTGAATGCTCATCTGGAAAACGTACGAGAGAAACAATCGAGATTTCAGACAGCCAATCGAACCCAAAAAGATCCAGATCAAATCCAAGCCGACCCACTTCATCAAATATTAACTTCCAACAACCCGGGAATCCCGAAACCGATACTGAAGCAATTGCGCAAATGAAGGAAATGATATATAGAGAAGCAGCCTTTCGGCCAGTGAATTTCGCGGCTGAGGCAGTGGTAGAAAAACCTAAAAGAAAAAATGTGAGAATATCAAGTGACCCTCAAACCGCGGCCGCTCGACAACGACGTGAGAGGATTAGTGAAAGAATTAGGGTTTTGCAGAAATTGGTGCCTGGTGGAAACAAGATGGATACAGCATCAATGCTTGATGAAGCTGCAAATTATCTTAAGTTCTTGAGGTCACAAGTGAAAGCCTTGGAGCAATTAGGGCATAATAGTAAAACTATTAATGCACTTGGTACAAGTCCAATAATCTCTCATGCCTCATTGGTTACACCTTTTACTCAAACCCCATTTTCCATGCAAACCCAATTTTCATTTCCTTGTGAAAACTTGTACCCTAATCCACCATCATCAATTCATCATTAG

AabHLH123

ATGTCTCATATAGCTGTGGAGAGAAATAGAAGAAGGCAGATGAATGAACACCTCAAGGTCTTGCGATCTATGACACCATGCTTCTATATCAAAAGGGTCGGGTTTTATCACGGAATTGTTTGTCCATCCTTGTCTCAACTTGCTTTGGCCTACAAGTCTGTGTTTTTAAAGCCGGAAACAGCTAGCCCTAGGGTTCGTCAAGATCCAAGTAATTCACAAGTGTTAGCGTCCTATGGAAGTGAAACAAATACAATTGCTATAATCAGACGTTCTACCGGAGATCAAGCATCAATCATAGGAGGTGTGATAGAATTCATCAAGGAAATGCAACTAGTTTTACAGTCATTGGAATCGAAGAAAAGGCGAAGGAGCATAAGTCCTAGTCCCGGTCCAAGCCCAAAGCCATTACTGCAACCTGAGACCCCACAGTCGGAGCGCTCTATAATTTCACATGAAAATATCAAAGAACTGGGAGCATCCTGTAACTCACCAGTTGCGGACGTGGAAGCAAAGATATCAGGGTCAAACGTTATCTTGAGAACCGTATCACGAAGAATTCCAGGTCAAGTTGTGAAGATAGTCAGTTTATTGGAAAAACTTTCTCTTGAGATCCTGCATCTGAATATCAGCAGCATGGAGGACACTGTCCTTTACTCCTTTGTCATTAAGATTGGACTGGAATGTCAGCTTAGTGTTGAAGAGCTTGCTGTTGAAGTGCAGAAGAGCTTCTCCCTAAATCATACTTAG

AabHLH124

ATGGATCGCGATTTCTTCCCCTTCGAAAACTCATCCCAAATGCCATCTTGGAAGGTAATGTCACAAGTGCCTGAGATCAATGACAGGTATAATAGTTTTGAATCTAATATGACTTCAATGACATCATCCCCTGTTTCCAATTCACCATCAAACTCACCTAAGGTAAATTGGCCAATTATGGACCATTTTGTGAAAGAAAACGCACCCAATATGCGAAATTTGATACCCGTGGCACCGGGCTTGCCTTCGCTTGTTTCGGATCCTGGATTCGCTGAACGGGCTGCGAAATTTTCGTGTTTTGGTAGTAGGAGTTTTAATGGGAGAACAAGCCAATTGGGGCTGAATTCTGTTGACCCGAATTCGCAATTTAGATCTAGTGTTCCTACATTGATTGGTAACTCGAAATTGCCTCGAGTTTCAAGTAGTCCTTTGTTGAAGATCAATAGATCAGGTCATGATCATAAAAGTACTAATTCTAATGAAGAACAAGAGTCCTCTGTTTCTGAGCATATCGAAATCGGGTTAAAGAATGATTCGAGTTCGAGAAAAAGAAAAGTTGCATCTTTGAAAGGAAAAGCTAAAGATATTGCATCACCCGGTGTAAAGGAAGAGGAAAATGATGAAATGAATACGAAGAAGCCGAAAATAACAGAGGAGGGTACTAATGGTGAAACTGAGAAACCAAAGGTTCCTGAACCACCAAAAGATTATATTCATGTTAGAGCAAGAAGAGGTCAAGCTACTGATAGTCATAGCTTAGCCGAAAGAGTTAGGAGAGAGAAAATAAGTGAAAGAATGAAGCTTCTTCAAGATCTTGTTCCGGGTTGCAATAAGGTGACAGGAAAAGCACTTATGTTAGATGAAATCATCAATTATGTTCAATCATTGCAACATCAAGTTGAGTTTCTATCCATGAAATTAGCAACCGTGAATCCACGACAAGATCTAGACATGAACGGTCACATCTCAAAAGATATGAATCAATTATACCCGATCGAACCGTCTACATCAGACTTCTACCAGCAAAACCCGCAACAATTGCTATTTATCGGGTCTACTCCAATGACCCAATCGCCCATGGACCCATTACCTACTGTTCATGGATTCTCCGAAACTTTCACTCAGTTTGCTGGATTTGAAGGGGATGATCTTCACAGCATTGTGAAAATGGGTTTTGGAGATAGCCGTAACCAGTCACCAGAGATGAAAATCGAGCTTTAA

AabHLH125

ATGGAAATGATGCCGATGATGATGGAACTTGATGAACTTCCTGAGCAATACCCTCTTCCATTCCACGATCTTTCGGACTCACCACTCGTCGAGTTCACAGGCGGCGCAAGCTCCATAACAAACCACCACACACCATCACAACATGGCCACCTTATGTACCGTACACCACCACCCGTAATCATCCCATCAGTGCATGTGAGAGCTTTACGTGAATTCCCACGTGGGAGTTGGTTTCAGCAGAAGCCAGACTCGGTTGAGGCCATGAGGGAGATGATATTTCGTATGGCGGCCTTGCAGCCCGTACAAATTGATCCTGAGTCGGTTAAGGCACCTAAGAGGCGGAACGTGAAGATATCCAAGGATCCTCAGAGTGTGGCGGCTAGACATAGGAGGGAGAGGATTAGTGAGAGGATTAGGATACTGCAAAGATTAGTTCCTGGTGGTACGAAGATGGATACGGCTTCTATGTTGGATGAGGCTGCTCATTATATGAAGTTCTTGAAAAAACAAGTGCAAACTCTAGAACAAGCTGCGGCTGCTAATGGTGGTGCTGGTGCTATGCCGACAAAAACATCAGCACCAGTACCACCACCAGGGTTTAATGTTGTTACTATGAACAACCATGGTACTAATAATCATTCTAATTTGGTCAGAGCTTGTGGTCAGCCTGCTAGTCATATGATAGGGTCTATGGAGTTGCTTAGGTGA

AabHLH126

ATGGATAGACATTTTTTCCTAAATAATGGGATGCATTCATATTCGCAATTCGACTCAGTTTTAAGCTCCCCTGTTTCAAATTCGACGATATCTAATGACATTGTGAAAGAGAATTTTCCAAGAATTCATAATTCAATGTCGTTGAGCCCGAATTTGCCATTGATGGCAGATGATCCTGGCTTCGCAGAACGAGCGGCGAAATTCTCTTGTTTTGGTAGTAGGAGTTTTAATAGTAGAAGTGATCAATTCGGGTTGAATTCGAATAAAGGAGAGTTTCCATTTGGATCTATTAATAATGTGTCTCCTGTTGCGAAATTCCCTCGTGTTTCGAGTGTTCCAATGCTGAAAATTGATTCATCTCCTGTTGGATTTGAGGAAAAGATGAATTTAGATGAAGTTAATATGAAATTGAGTAGATTTGATGGCTCTGCTGCTAATTCTAATGAAGAATCCTCTGTTTCTGAGCAAATTCCTGTTAAGAAAGATTTCAGTTCAAGAAAAAGAAAAGGCACATCAAGCGCGAAAAAGGAAGTAGAAGCAAATGATGATTCGAATTCGAAAAGATTGAAGAAACCGGAGCAAGAAAATGGTGGTAAAACGGAGGAAAACTCAAAGTTACCCGAAGCACCAAAGGATTATATTCATGTTAGAGCAAGAAGAGGACAAGCTACTGATAGTCATAGTTTAGCAGAAAGAGTTAGAAGAGAAAAGATTAGTGAAAGAATGAAGCTTCTTCAGAATCTTGTGCCAAATTGTAACAAGGTAACAGGAAAGGCACTTATGCTTGATGAAATCATCAATTACGTGCAATCGTTGCAAAGACAAGTTGAGTTCCTTTCGATGAAGTTAGCTACGGTGAACCCAAGTCTCGACTTTGACACCAATGACCTACTCTCGCAAAATGTTAATCAACAAAACACAAATCTACTACAACCATCGACTTTTTATCAACAAAACACACAAGGACTCTACAACGGGTCTGCCCCAATGACCCAACCCGTTCATGAATTTGTGGAACCTTTCCCTCAGTTTTCGGGATTTAGTCAAGATGATCTTCAAAGCATTGTTCGAATGGGTTTTGGTGAAAATCTTGATCTTGATAACTCATTTTTTCAAACGACTCATGATCAACCATCTAATATGAAAATCGAGCTCTGA

AabHLH127

ATGGAAAAAGAGAAGTATTTCAATAATGGGAATGGAATCCCACAAGCATGGAATTCCATCTTTGGAATGGGAATGGGAATGGGAATGGGAATGCAAACTAGTGAAGGCTTTTTTAACCAAAATTGTGAAAATTCAGTTGATCAAAGAGATATATTTGAGTCTGCTTTGAGCTCAATAGTTTCATCTCCAGCCAGTTCTCACCCGGGAATTGGGCCAGGAATCCCAATTCCCGGAGGAACTGGCGGAAATGAAAGCATAGTTGTAAGAGAATTAATAGGTAGACTAGGAAGTATATGTAACTCTGGTGATAATAATGGTAACAATAGTACTAATAACTCTTGCTACAACACACCTTTGAATTCACCACCAAAACTTAACCTTTCAATCCTAGATCACCCAATTCCCGGGAATTCAGTTCCGGAGAATACAATTCTCCGGAATCCAGTTCCCGGGAATCCTAGTTTACCATTAGCTCAATTCTCTAGTGATCCTGGATTTGTTGAAAGAGCTGCAAGATTGTCATGTTTTAGTGGTCAATCTTTTATGGGAATCAACAAACTTTCAACACAAGCATGTGAAGAATCTGTTTCGGTTTCCGCAGAAACCGAAACAGGAATGAAAGTACAAAGTACTAATAGTAATGTAAGAAAGAGAAAGATGGTTCCCAAAGGAAAAGGAAAAGAAACACAATCTAGTAGTACTAGTTTCTCTGATAAAGATGACAAGGTTGTGGCAGAAGCTGAAAAAGTGGAATCAGATGCAAAAAGAAGTAAGTCAAATGATGAGGAAAATGGGAATGAAAAAGATGGGAATGGGAATGGGAATGAGAAACAAACAAAGGAAAATGCAAAGGCAGAACCACCAAAAGATTATATTCATGTTAGAGCAAGAAGGGGTCAAGCAACTGATAGCCATAGTCTTGCAGAAAGAGTTAGAAGAGAGAAAATAAGCGAAAGGATGAAGTTTTTGCAAGATCTTGTTCCTGGTTGTAATAAGGTAACAGGAAAAGCTGTTATGCTTGATGAAATTATAAACTATGTGCAGTCATTGCAGAGACAAGTTGAGTTTTTGTCGATGAAATTGGCGACTGTTAATCCTAGGATGGATGTGAGCATTGAATCACTTTTATCTAAAGATATGTTTCGGCCTCGAGTGTCTATGCCAACAAACATGAACCCTTTTGATGCCTCAGCACAACCATTTCCTTATGCCTTCCAACCACAAAATAATGGCATCGTTCCTGATGTATCCGAAAATCAGTTCTCCGTGAACCCTTTGATGGCGGCAATGCACCGCAACTCACTCATGAAACCATCCCACATTGATGGTCTTGGTGAGGCTTCAACATTTTGGGAAAATGATCTTCAAAGTGTTGTCAAGATGGCATTAGGACAGGATCAGCCACAAGGATTCCATGGTCATATGAAAATTGAGCTATGA

AabHLH128

ATGGCTGGTGATGATGAGATTGAGGGGGGAAATAGAGACGAGGAACAAATTCATTACAATTCAGCTAATTTATCAGCTGATTGGCCTTTCAATGGTACTAATGAACTTACAAATACATCTATGAGTAATCCTATGAGCAATATTTGGGATCATCATCCAACAAGTTCACAAAATTTAGGTGCTTTTTGTGATGTGAATTTGCAAAATAATCCGACAACTTCATCGTCGTCTTTAGGGTTTAGAAAAGGTAATCTGCTTGTTCCTCAAAGAAGTCTTGATATGGGTTGGGCTCCACCAGATTCCGCCATTAAACGAGGTGGTATGTTCTTGCCACCGGCTGCTTCTGCCATGCTTCCTCATAGCTTATCTCAGTTTCCGGCTGATTCGGGTTTTATTGAGAGGGCAGCTAGGTTATCGTCGTTTAGTGCTGGTAATTTTGAAGATATGATAAACCCTTTTGGCAATGTGAATGATTCATCTTTAAGTCCTTATTCGAGTAGATCAGTTCAAGGGCAAGCACAAGAGGGGTTTGTTGGAAATGGGTTCAAATCGGCTTCTGGTGACGGGTCTAAAGATATGTCATTGCCTGTTGACTGTGGGGCTAATGATGGAAGTCAACCTAAACAGGGTGGTTCTGGTTTTTCGGGCAATAATGGGACTGGTGATGCTGAATACAGTGAAGGTGGTGGTCAGGATGGAAATTCTAATGAGGGTCTTGGCTCAAAGAAAAGACGAAGAAGTGGTCAGGATACAGAGTATAATCAAGCCAAGCGATCCCCACAAACACCTAGTGATACTACAAAGAACAACACTGAAGTTCAACAGAAGGCTGACAATACCCCGAGTTCACTTGTTAATAAATCGGGTGGGAAACATGGGAAGCAGGCTCAGTCATCGGATGCACAGAAGGAAGAGTACATTCATGTAAGGGCGCGAAGAGGCCAAGCTACCAACAGCCATAGTCTTGCAGAGAGGGTGAGGAGGGAGAAGATAAGTGAAAGAATGAAGTTCCTTCAAGACCTTGTTCCTGGATGCAGCAAGGTCACAGGCAAAGCGGTTATGTTGGATGAAATTATTAACTATGTACAGTCATTACAGCGACAGGTTGAGTTTTTGTCGATGAAACTCGCTACTGTAAATCCTCGGATGGATTTTAATATTGAAGGACTCCTGGCAAAAGATATACTTGAATCACGACTGGGACCTTCTGGTCCACTTGGTTTTGGTGGTGATATGGCGATGCCTTATACTCCTAATCAATCACAAATGGCTGTCATGCAAGCCGGGATTCCTGGTGTAGGAACCTCTTCAGATGCTGTAAGAAGAACGATCAATTCCCATTTGATGGCTCTTGGTGGAGGATATAAAGATCATACTTCCCAGGTACCGAGTTCTTGGGATGATGAGCTTCATAACATTGTCCAAATGGGATTAAATCCTGGTACCCCAGTTAGCAGCCAAAATTTAGGATCCACACCTCCAGGCCATCTGAAAGCTGAACCATAA

AabHLH129

ATGTCAAAAGATGGTTTCTTCAACACACTCCCACAATCTTGGAATTCCATGTTTGGAATGGAACTTGACTCCCAAGTTAATGAAATGAACCTTTTTAATCATAATTGGGAGAATTCAATGGATCAGAGCGATCCCTTTGAATCCGCATTAAGTTCTATAGTCTCATCGCCAGTAAACAGTCATCCTGGGACAGGTATCAGGACACCTGTCCCAGGATGCCATGCTGGCGGTGAGAGTGTAGTTTTGAAGGAACTTATTGGTAGATTAGGAAGTATATGTAATTCTGGCGAAATTTCGCCAGAATCATGTATACATGGAAATAATACTAATAATAACAGTACTACTACATCATGTTACACTACTCCGTTAAATTCGCCACCTAAGCTTAATCTTTCGATAAATCATAGGAGTCATCATCAGTTGCCTATGATTCCTAGTGATCCTGCATTTGTTGAAAGAGCTGCAAGGTTTTCGTGTTTTGGTGCTAAGGAAGGTGAGTTTCAACATATGGTGGAATCTGGAAAGATGTCTAGAGTGTCGAGTAATCAGTCTTTTATTAAGACGGGTGGATCGGTTAGTAGGTTGTCGATGAATTTGAATTCGGATAATAAGGAAGTGGATCATTCTGTTGAGGTATCATCTTTGTCTGAGCAGATTAATGGCTCGGAAACCGGAATTAAAGGTAGAAAGAGGAAGGTGATTTCTAAAGGGAAATCGAAAGAAACCCAAGTCGATAATAAGGCTGTTGCGGAATCTGAGAAAGAGGAATCAGATGCAAAAAGAAGCAAGTCAGATGAAGAAGGGAATGGAATAGAACCCGAAAAAGAAAAGGAAAAGGCAGAAGGGAATCAGAAACAAACAAAGGAAAATGCAAAATTACCCGAACCACCCAAAGACTACATTCATGTTAGAGCAAGAAGGGGTCAAGCTACCGATAGTCATAGTCTTGCAGAAAGGGTTAGACGAGAAAAGATCAGTGAGAGAATGAAGTTCCTTCAAGATCTTGTCCCTGGTTGTAACAAGGTGACTGGAAAGGCTGTTATGCTCGATGAGATTATAAACTACGTACAATCATTGCAGAGACAAGTTGAGTTCTTGTCTATGAAATTAGCAACTGTTAATCCTAGGACGGATGTGAACATGGAAGCACTTTTGTCTAAAGACATTTTCCATTCTCGGCCATCCATGCCAAATCCCATGAACCATATGGAAGCATCAGCTCAACCGTTCTATGGCATGGTTAATGACGGGCCAGAGAACTCATTAATGGCCATGATGCATCATGGTTCAAATATGAAGTCATCACAGATTGATGGTTTCAGTGAAGCTTCCGCTTTCTGGGAAAATGACCTTCAGAGTGTTGTACAGATGGGGTTTGTCCAAAATCAAGGACCGAGCTTCCACGGAACAATGGGCTCGGGTCAAATGAAAGTTGAGCTATGA

AabHLH130

ATGGAAGGCAACCCTAGTTCATCGTGTTCAAGGGTTGATAGAAGAACAATTGAGAAAAATAGAAGAATTCACATGAAGGCTCTATACTCCAAACTTCATTCTCTTGTTGCTCATGACTCTTCTAGGGAAATGACATCCCTACCAGATCAGCTACACGAAGCTGCAAATTATATAAAGAAACTCCAAATCAAACTTGAGAAAATGAATGAAGAAAAGAATAATTTAATGGGAATCAAGAAACTTGAAATTAATAATAACCATAAAATCAAATGCTTGAATATGATGGTGGGGCAAGCAAGAGCGCCTCAGATTGAGGTTCGTGAAACCGGCTCTTCTCTTGAAGCGGTTCTCATCACCGGAGTGGACTTTCAGTTCTTGTTCAGTGAAACAATCCGTGTGATTCATGAAGAAGGTTTTGATGTTGTTAACGCCGGATTTTCGATCCTTAACGATACTGTTTTCCATACCATACATGCTCAGAACAGTACGAGGTTCCATAATTAA

AabHLH131

ATGTTGCCCTATTTCCAACATAACAGTTGTTCAGAGGTCTCTAATTTTCTATTTCCATCAACCCTCTATCAACAAGATCTTCCTAATCCTGATAATCAAAAACCGGCTGGCCATAAGAGATCATGCTATAATTTCCAACCTCAGGAAGATCACCTCGTTATCATGGATAACGGCGTAGACAAGGAAAAGAAGAAGAAAAAGATGGTCCATCGAGAGACCGAAAAGAAAAGACGACAAGAGATGTCTAATCTATCCTCGTCTCTTCGATCACTCCTTCCTCTTCACTTGATTAAGGCTGTGAATTACATAAAACATTTGGAAGAAAAGACCAAAGAACTAAGTATAAAAAGAGATAAGCTCAAGAAAATGTGCAATGTAGTTACGGATGCAGAAGTTGTACTAAAGAAGAATGATAAGAGCGAAAGGGTAATGGTCAAATGTAGTCAGAATCCTATTACAGTGACAGTCAGTTGTAGCGAAGGTGGAATTGAGATTTTGGTCAAGTCTTTTATTAATGAAAACAAGGGTCTTCAAATTTCTAGAGTGTTGAAAACACTTGTTCATGAAGGGATTGACGTTATAAGCTGCAATTCAACCAAAATCAATGACACCTTGTTAATCTATACCATCCATTCTAAGGCACATAATTGCCGGGATATCAAGACCCCTATTATCCAATTCCACCCTTGTTGGGATGCGCTTTCTAGCAGCATGCCAAACAAAAAGACAAATTTTCAAGGGAACTTGATGGTTCCATCTGGTGGTGGTGGACAAGGTTCATACAGTGGTGGAAATGGGTCAAACAGTGGTGGAAATGGTTTTGGTGGACAAAATAGGTACAATTACAGACCAAGGAATGTGAATATGGTTGCTAGTCAAGGAACAACTCCAGATGGGGCATCTACAAGTGAAACATCTACAAGTGGGCAAGAGGATCAAGCAGATACAGATGAAACAAGACTACCAAGAAGTCAAGAACAAAACTTTTAA

AabHLH132

ATGGCTCTTAGCTTCTATACCAATTGGTCTAATTATGACTCAAGTGTCACCTCTCTTTCCTGGCCATCAGAAGCCTCACAAGAGCTGCCTTGTTTCCATGAAGCCTCTACATTTTACGATACCATTAACCCAAATTTTGACACAAACTACACCAACAATTTAGATTTTTTGGGTCTATATTCATCAAGGTATCCCGTTGAGCCAACCCCAAATGTGTTCATGCAGGAATTGCAAGATCCTAATTATCACACCTTTCCATACTCAAATACTATTCAACATGAGAATTTGCTTATGGAGTACACTATGGGGCCAGAGCTACCGTCTCTAGTGCCGCCATTTCTTGACAGTTTAACGTACCAAGGAAATGGTAGTGTTGCTGCATTGCCTCCTTGGTACAATTGTGGACTTCAGGGACAAACCCAGGTTGTAAGTGGCGTGAAAGTGAAGAAGCAAGATGCGAATAACGAAGAAAGGAGTTTAACTGCACAAAGTTTGGCGGCAAGGGCAAGGCGGAGGAAGATAAGCGAGAAGACGCAAGAGCTCGGGAAGCTAATTCCCGGTGGTCAAAAGATGAACACGGCCGAGATGTTTCAAGCTGCTTTTAAGTATGTTAAGTTCTTGCAGGCTCAAATTGACGTTCTCAAACATATGGCCTTACTTCCGGAAAGTGAAGAAGTACTGGGTAATGGAGATATGCAAGATTTGGTGACTTGTGCCTCGATTCAAGAGAAGTGCATTGGACCAAATACATTGCAAATATCGTCCAATGACCAACATTAA

AabHLH133

ATGGAGGGTTTTACATCAAGTTTCCATAGCTTAAAACCTTCATTTTCTTTCATAGACATTGAACAAAACATGGAACTATTCAACCAGTTTTCAATCCAATATGACAACTCAAGCATGAGTACTTCTCAAAGCTTCATGGGAATTTCAAATGATAATTTCATGTCTCAACAAGTATCACCATCACTTGATCAACAATTCGTGCAAAGCTTTCAACCAGTCTTTCAACATGAAAAGAAAAATGTTATGGTGATTCCAGAAGCCGCTCCTATGGGACCGGTTCTAAACGGCAAGAGAAAATCAATGGATGTATCATCAAGCAGTTCCGGAAATTCGTCATCTCATCTTGTCGCGGATTGTGAGATTGATGGCAAAAAATATCAGAGTTCAGGTAAGGGCAAAAAAGCAAAAGTGAGTGACAATGGAGAAGCACCAAAAGAAGTGGTACACGTTAGAGCTAGAAGGGGCCAAGCAACTGATAGTCACAGTATAGCAGAAAGGATTAGAAGAGGGAAAATTAACGAGCGTTTGAGATGCTTGCAAGATATCGTTCCCGGGTGCTACAAGTCAATGGGCATGGCGGTAATGTTGGATGAGATAATCAACTATGTACAATCCTTACAAAATCAGGTGGAGTTTCTTTCGTCGAAACTCAGTGAAGCAAGCAGATTTCAAAACTTCTATTCAGAGTCTATGCATATCGATGCATTTCAGATGGGAAATGTTATTGAAGGACTGAAGTTGCAAAGATTGGAAGAGAATGGTCCAGTTGACCAGAGTTTTGGCCCATATCCTTCATTGCCATACCATAGAACATAG

AabHLH134

ATGTCTCATATAGCTGTGGAGAGAAATAGAAGAAGGCAGATGAATGAACACCTCAAGGTCTTGCGATCTATGACACCATGCTTCTATATCAAAAGGGTCGGGTTTTATCACGGAATTGTTTGTCCATCCTTGTGTCAACTTGCTTTGGCCTACAAGTCTGTGTTTTTAAAGCCGGAAACAGCTAGCCCTAGGGTTCGTCAAGATCCAAGTAATTCACAAGTGTTAGCGTCCTATGGAAGTGAAACAAATACAATTGCTATAATCAGACGTTCTACCGGAGATCAAGCATCAATCATAGGAGAACTGGGAGCATCCTGTAACTCACCAGTTGCGGACGTGGAAGCAAAGATATCAGGGTCAAACGTTATTTTGAGAACCGTATCACGAAGAATTCCAGGTCAAGTTGTGAAGATAGTCAGTTTATTGGAAAAACTTTCTCTTGAGATCCTGCATCTGAATATCAGCAGCATGGAGGACACTGTCCTATATTCCTTTGTCATTAAGATTGGACTGGAATGTCCGCTTAGTGTTGAAGAGCTTGCTGTTGAAGTGCAGAAGAGCTTCTCCCTTAATCTTACTTAG

AabHLH135

ATGGAGGGAAGCATCAAGTGTTCAGCTTCAGATTTGAAGAAACCAGAAAGGAAGATTATTGAGAAGAATAGAAGAAACCAAATGAAGTCTCTTTACTCTAATCTCTTTTCACTCATTCCTCCTAATATCTTCTCCAAGGATGGTGATGTGTCGGATCGTGTGGATAGAGCCATAGAGTACATCCAAATGTCGAAAACTAACTTGGATATGCTCAAGAACAAGAAGGAGAAGTTGTCTAGTAGAAAGAGATCACACGAACACACAAAAATAATCAAGAATGTGTGCAAGCCGGTTGATATTCAAATCCATGAAATTAGTCATGATATTGATGCTGTTATGGTAACAGGATTGGATAACCATTCGAGCTTTTGTGACGTTGTTTGGTTACTAAACCGGTATAGTGCTGAGGTTACACTAGCAACTTTTTCAAGCAACGGGCATTCGACCTTTAACATCCGTCAAAAAAAGATCGAAGGAAAAGACATATGTAAGAGGCTCAAGACTTTGCTTGAAGGGTCTTTAAATGTGAAGGAGTTGGAGAATAATCATGCCTTGTTAACTCTTCCTCAACCAGGATTGGACACCGGAATTGAAAGTGGACCCCAAAGAATCGAAGAATCACAGATAAATTTACAGAAAGAACTTAATGAACTCGAGTATGATTCGAATTTAAGTATATGGGACCTTGATTTCCAGTCAAATGTATGGATTACTTGCAATGAACTCGATTATGAGTCCAATTTAAGTATATGGGACTTTGATTTCCATCCAAATGTATGGGGATCTGAGTTAGAGGTGTTCCAATAA

AabHLH136

ATGTACAATGAAACACAGTTTCGTATGTATCATGAACTAAAAACATTTCGTATGTATCATGAACTAAAAACTGTTGGTGGGTCAATCCAATATGAGCTTCATTATATTATACATTGTTCTCTTGATGTTGTTGATGAAAGAGGTATTATCATTAAGCCGTCGTATGACCGGAAGGAAGATGGTTTTGGCAATAGTGAGCCAAATGAGTCAAATCGATCTAATGATGGAATGAAGCAAGAGCATATGGTGTATGACCCTCAAGGCCCATATGGCCTAAAAACATTTCGTATGTATCATGAACTAAAAACTGTTGGTGGGTCAATCCAATATGAGCTTCATTATATTGTACATTGTTCTCTTGATGTTGTTGATGAAAGAGCAATTCGGTTCAGAAAAATTCAAAACAAAATCCCAGGCATTCAATTGTTTCGTCTTATCTGTCCTAATGTTTCAAGTAATGTTAAATTGCTCCATACTGTTCAATCACACAACAGGCGACGCAGTAACTCATGCAGCAGGGCAGAGAATAAAGCATGTCGTGAAAGGCAAAGAAGGGAAAAGTTGAATGAGAGATTTGTGGAATTAAGCTCTACTTTGGAACCTGACTGGTCTGCAACCACCGATAAGCTAGCTATTATTGGAGATGTTATTCGAGTTCTAAATCAATTAAAAGCTGAATCTCAGGAGTGCAAAAATATGAACGAGAAAATATTGGAAGAGATCAAAACATTTAGGCAGCAGGTCAAAACCATGACCAATAATCACCTTCCGCCACCTGGATTTATGTCACCACATCCGGCTGCATATCAGGCTGTAGCAAACAAGATGCCTATTTTTTCAGGTTATGGTTACATTCCAATGTGGGAGTATCTGCCACAAACTATGTGTGATACATCTCATGATCAAGAGCTGAGGCCACCTGCTGCTTAA

AabHLH137

ATGGACTTTTCACTTAGACTTCTACGATTGCAAGATAAGGTTGTGTGGCTAGAACACCAAGGAGTTCAAAATACCTCATTTAGAGACACCTATGAATCATATGGGCTTTCACCTAGGATAGATTCTTATGAACAACCTCCATGTTTAGGATCTCGTTTTAGTGAAGCTAATAGGAAAAGCGACCTAGTTTACCAAGGAATTGTGGATAGAGCAGTAGCAATGAACCATAAGTTGGATGAAGCACTAGAGCTATGTAGCCTGAAAGTAAAGAAACAAGTTGCGAGTAACGAAGAAAGAATTTTAGGAGCAAAAAGTTTCGCGGTAAGGGTGAGGCGAAGGAATATAAGCGAGAAGACACAAGAGCTTGGGAAGCTAATTCCTAGTGATCAAAAGATGAACACACCCGAGATGTTTCAAGCTACTTTTAAGTACATCAAGTTCATGCAGACTCAAATTGGTGTTCTCATTCCATATGGCCTTACTTCCGGTAATTTACAACAAGAAGCGGTTAGATAG

AabHLH138

ATGGCTCTTAGCTTCTACACCAATTGGTCTAATCATGACTCAAGTGTCACTTCTCTTTTCTGGCCATCAGAAGCCTCACAAGAGCTGCTTTGTTTCCAGGAAGACACTACATTTTACGATACCATTAACCCAATTTTCGACACAAACTACACCAACAATTTAGATTTTTCGGGTCTATTTTCGTCAAAGTATCCCGTTGAGCCGACCTCAAATGTGTTCAAGCTGGAATTGAAATATCCTAATTATCACACCTTTCCATACTCAAGTACTTTTCAACATGGGAATTTGCTTATGGAGTACACAATGGGGCCAGAGCTGCCGTCTCTACTGCCGCCATTTCTTGATAGTTTAACGTACCAAGGAAGTGGTAGTGTTGTTGCATTGCCTCCTTGGTACAACTGTGGACTTCAGGGACAAACCCAGGTTGAAGAGATAAGTAGCGTGAAAGTGAAGAAACAAGATGCGAGTAACGAAGAAAGGATTTTAACTGCACAAAGTTTGGCGGCAAGGGCGAGGCGGAGGAAGATAAGCAAGAAGACGCAAGAGCTCGGGAAGCTAATTCCCGGTGGTCAAAAGATGAACACGGCCGAGATGTTTCAAGCTGCTTTTAAGTACGTTAAGTTCTTGCAGGCTCAAATTGGTGTTCTCAAACATATGGCCTTACTTCAGGAAAGTGAAGAAGTATTGGGTAATGTAGAAATGCAAAATTTGGTGAACTCTGCCTTGATTCAAGAGAAGTTGTACACTGCAGAGAAGTGCATTGGACCAAATACTTTGCAAAAACCGTCGAATCATGACCAACATTAA

AabHLH139

ATGCTTCATCGAGAGATCGAAAGACAAAGGAGGCAAGATATGACTAAGCTTAATGGTTCACTTAGGAATTTACTTCCTATTGAATTCGTCAAAGGAAACCGTTCAATATCGGATCATAGGCATCAAGCAATGCAATACATCAAACAAATGGAGGAAAACGTCAAAGGGCTAAGTACGAGGAGAGATAAGCTCAAGAATAACAAAAGCTCAAGCTCAATGAATCACCTGCATAATACTGTCTCAGTCAACCTTTGCAATGGTGGAGTTGACATTTTGATCAATAGTTGCACTATAGAGGACGGGTTCCACCTTTCGCTAGTACTAAAGGCTCTTGATGAAGAGGGTCTTAATGTTACAAGTTGCACTTCAACCAAAGCAAATGATCGTTTACTTCACGCTATTCAGTCTGAGGAAAATCTGGCATCACTTGATCTATCAATGTTACAACAAAGACTAACGTTTGTAGCAAATACTCAGCCAAATTATTATTAA

AabHLH140

ATGGTGGGTAACAATAAGTGTTCAACTTCAAGATTGAAGAAACCAGAAAGAAAGATTATTGAGAGGAATAGAAGAAACCAAATGAAGTTTCTTTACTCTAATCTCTTTTCACTCATCCCTCTTAATCTCTTCTCCAAGGATGGTGATGTGTCGGATCGTGTGGATAGAGCCATAGAGTACATTCAAATGTCGAAAACTAAATTGGATTTGCTCAAGAACAAGAGGGAGAAGCTGTCTAGTAGGAAGAGATCACACGAACACACAAAAATGATCAACAATGTGTGCAAGCCGGTTGATATTCAAATCCATGAAATTAGTCATGATATTGATGCTGTTATGGTAACAGGATTGGATAACCATTCGAGCTTTTGTGACGTTGTTTGTTTACTAAACCGGTATAGTGCTGAGGTTACACAAGCAAATTTTTCAAGCTACGGGCATTCGACCTTTCATATCCGTAAAAAAAAGGTTGAAGGACAAGACATATGTAAGAGGCTCAAGAGTTTGCTTGAAGGGTCTTTAAATGTGAAGGAGTTGGAGAATAATCATGCATTGTTTTCTGTGCCCGTAACTCTTCCTCAACCAGGATTGGATACCGGAATTGAAAGTGGACCCCAAAGACACGAAGAATCACAGATAAATTTACAGAAAGAACTTAGTTCTCAATCATGCAATGAACTCGAGTATGATTCGAATTTAAGTATTTGGGACCTCGATTTCCAGTCAAATGTATGGATTACTTGCAATGAACTCGACTATGAGTCCAATTGA

AabHLH141

ATGGATGCAAGCCTCAAGTGTTCACCTTCAAAGTTGAAGAAGAAACCAGAAAGGAAGATTATTGAAAAGAATAGAAGAAACCAAATGAAGTTTCTCTACTCTCATCTCTTTTCTCTGATTCCTCCTAATTACCTCTCCAAGGTAGGTGACGTGTCGGATAGAGTAGATAGTGCCATAGAGTACATACAAGCATTGAAAACCAACTTGGACATAATCAAGAACAAGAAGGATAAGTTGTCGAGTCAGGAGAGATCACACGAACACACAAAAATGATCCATAATGTGTGCAACACAATTGATATACAAATCCACGAAATGATCAGTCATGATACTGATGCTGTTTTGGTAACAGGACTGAAAAATTATTCAAAATTTCGTGATGTTGTTTGGTTTCTAAATCAGTGTACCACTGAGGTCACGCTTGCAAATTTTTCATGCACCGGGCATTCAACCTTTCACATTCGTCAAAAAAAGGTCGGAGCAGAGGCTATTCGTAAGAGGGTCAAGAGTTTAATTGAAGGATCTTTAAATGTGAAGGAGTTGGAGAATAATTATGCATTGTTTTGCACCAACGTAACTTTTCCTCAGCCAAGATGGGGCAGGACTGGAAGTCAACTTGGAGAAATCGAAGAATTACAAATGAATTTTCAAAAAGAACCCAGTTCTGTTTCATTTTGCAATGAATTGGACTATGAGTCAAATATAAGTATATGGGACTTCGAATCTAATGTAACGTTACAGTGA

AabHLH142

ATGAAACGCCCTTCGGGTTCATCTTCTCACCGGCCACCCGCCGCCATGGAAAAACAACGCCGGGATCGAATGAAGAATCTCTACTCCAAACTTGCCTCCCTCCTTCGTCTTCAGTCCTATGAAAGGATGCCATTGCTTGGTTTATTGGAAAAAGCTACTGATTCGATAAAACGATGGAAAGAAATGGTGGAGCGATTGACAGCTAGAAAAAAAGAGCTAGAAAACGAGCTAAGAGGTGCGATGAGCAATGAAATCAACTTAAATGTCGTTCAAGTGAGTGAAATGGATTCAAACCTCGAGGTGAACTTAATCATTAAATCAAGTAACAAGAGAATCGAGCTTTTACGAGTTCTAAATATTATAGAGCAAGGTGGTGCTGAGATTATAAATTATAGTCTTTCAAGCATGGGGCAAAACACACATTACACCATTCATGCTCAGGCACTCTATTCACGGTTTGGGATTGATAGTTCATTGATAGAATACAATCTTAAGCAACTTGTTTCTTAG

AabHLH143

ATGCAGCGCCCTTCGGGTTCATCTTCTCTCCGGCCACCCGCCGCCATGGAAAAACAACGTCGGGATCAGATGAAAGATCTCTACTCCAAACTTGCCTCCCTCCTCCGTCTTCAGTCCTACGAAAGAATTACATTGCTTGGTTTATTGGAAAATGCTACTGATTCTATAAAACAATGGAAAGAAACAGTGGATCGATTGACAGCTAGGAAAAATGAGCTAGAAAACGACCTAAGAGGTGCAATGAGCAAGGAAATCAACTTACATGTCGTTCAAGTGAGTGAAATGGATTCAAACCTCGAGGTGAACTTAATCATTCGGGCAAGTAACAAGAAAATCGAGCATTCTCGAGTTCTAAGTATTATAGAGCAAGGTGGTGCCGAAATTAAAAATTATAGTCTCTCATGCATAGGACAAAACACACATTACACCATTCATGCTCAGGCACTGCATTCACGGTTAGGAATTGATAGTTCATTGATAGAATACAATCTAAAGCAACTTGTCTCTTAG

AabHLH144

ATGGCAGATAGTTGGTGGGATTCAAGAACGACAAGGCCTTGTTTGGATTCAGTTTCTGTTTCCATGAATCTTTTTCAAGAAACCACCACCACAACTACCACCAGTGGTGGCGGTAGTGATCATGGAGTGGTGGGCAACAATCCTAGCTTACAAATGATGGAATTAGGTCTTTCATCACAACCAACATCACAATCTTTGGATTGGAACCAAGCTTTATATCGTGGAGATCAAAGAAATGAACATCATCAAAGTGGTTACCAAACCCTAATCCAAGATGATCATGGTTTGAGCTCAAACACAAGTAATTTCCAAGAAACTCAATGGAAATCACATAAAATGTATTCGGATTCGCCATCAGAATACAAGCAAATCAATGCTAGAGGGTTTCGGCTAGAAGAACCGGTGCATTACAATGACGAAAGTGGATTAAATCCAAGCTTCCAAACGTTGGATCATTCATATGGAAGTAACTCTACGGTACTACAAAGTTTATTTGGTTCAGATAATAACACGAATCAGGATGTTGCTGATTCGTGTTATGATCAGAACCAAGGAATTAGTTATAATTCCTACCAATCTAGCTATGGTGGAATCACTATGTCAGGTGGTGGCGGCGGAGGAGAGTACCCTCCTCATCCACCTCAAGAATTCCCAGTGAATTCACCACCTAAAGTGCAACCACCGAACATTACACCATTACATTTCTCCAACAACGCACGCTTTTGGAATGCTTCAGCCGCCTCCATGAATGATGTTAGATCAAGTTTTTTTCCTTCGCAAATGCAATCAACTTCATCAACTATTGAGGATAAACCAAAGAATCCCATATCAGAAATTGTGAAAAAAACCATTAGCAAATCATCATCAACCAAAAGACCAAGAAATGAAAACCCACCATTGCCAGCTTTTAAGGTGAGAAAAGAGAAGATGGGGGACAGAATTACTGCACTCCAACAATTGGTTTCACCTTTCGGAAAAACTGATACGGCTTCCGTGTTGACTGAAGCTATAGAATACATCAAATTTCTCCACGAGCAAGTTAATGTTCTAAGTACTCCATACATGAAAAATGGAGCTGCTCCCATGCAACAACAACAACAACAGATTCCGGATAAACCTTTGGAAGGATCAAGACAAGATTTAAGAAGTCGAGGGCTATGTTTGGTACCGATATCAAGTACATTCCCAGTGACACACGAAACAACAGTTGATTTTTGGACACCATCTTTTGGAGGAACCTTTCGATGA

AabHLH145

ATGGATGCAAGCCTCAAGTGTTCACCTTCAAAGTTGAAGAAGAAACCAGAAAGGAAGATTATAGAAAAGAATAGAAGAAACCAAATGAAGTTTCTCTACTCTCATCTCTTCTCTCTAATCCCTCCTAATTACCTCCCCAAGGTAGGTGACATGTCGGATAGAGTAGATAGTGCCATAGAGTACATACAAACATTGAAAACCAACTTGGACATTATCAAGAACAAGAAGGATAAGTTGCCGAGTAGGAATGGATCGCACAAACACACAAAAATGATCAATAATGTGTGCAAGCCAATTGATATACAAATCCACGAAATGAGTCATGATACTGATGCTGTTTTGGTAACAGGACTGAAAATTCATTCAAAATTTCGTGATGTCGTTTGGTTTCTAAATCAGTGTACTACTGAGGTCACGCTTGCAAATTTTTCATGCTGCGGGCATTCGATCTTTCACATTCGTCAGAAAAAGGTCGGAGCAGAGGCTATTTGTAAGAGGGTCAAGAGTTTAATTGAAGGATCTTTAAATGTCAAGGAATTGGAGAATAATTATGCATTGTTTTGCACTAACGTAACTCTTCCTCAGCCAAGATGGGGTAGTACTGGAACTCAACCTCGAGAAATTGAAGAATTACAAATGAATTTTCAAAAAGAACTCAGTTTTGTTTCATCTTCCAATGAATTGGACTATGAGTCAAATATAAGTATATGGGACTTTGATTTCCAATCTAATCTTTACCATTTATGTGACATACTAAGATATGAGATTCACTCATATCCTATGCACTCAAATGTATATCACAATGTAAAAACCTTTTACCCAAAGAAAGTATATCACAATGTTGAGTCTTCTATCTATACTAGGAAAATTATAGGGCTATGCATAGCACAACCCATGGACGTTCATAATCATATTGATTTCTTGAGTGCCTTTGAGTGA

AabHLH146

ATGGTGGTGGCAGTCGTTATGTCTTATGTGATGTTTGATCCAACTTATGAACTTTCACAAATAAGAACAGTGATAGTGTTGCGACACTTATCTCACATGAATAAAAAAACAATTAAAATGAGCAAAAAGTTTCGCGGTAAGGGTGAGGCGAAGGAATATAAGCGAGAAGACACAAGAGCTTGGGAAGCTAATTCCTGGTGGTCAAAAGATGAACACACCCGANCAGAGGTATGTAGCCTGAAAGTAAAGAAACAAGTTGCGAGTAACGAAGAAAGGATTTTAGGAGCAAAAAGTTTCGCGGTAAGGGTGAGGCGAAGGAATATAAGCGAGAAGACACAAGAGCTTGGGAAGCTAATTCCTGGTGGTCAAAAGATGAACACACCCGAGATGTTTCAAGCTGCTTTTAAGTATGTCAAGTTCTTGCAGACTCAAATTGGTGTTCTCATTCCATATGGCCTTACTTCCGGTAATTTACAACAAGAAGCGGTTAGATAG

AabHLH147

ATGGATGCATCTCCAACTCACCACCAACACCAGCTTCTTCTTGCTGATCAATGTCTTTCTTCTCGTAACATCGCCACTGCCAACACGACGAGCGACAACAATGAGTTTATCCCTCATAGTTTCATTGAATTGAGTGCTCATGGCATTCTAGATTCCAGTGAAAATAACCTACAAAGCTCAAATTCTTTAATGTCTATACATGATGACAAACGTGGAAGGAAGATGCCAACAACCAACTCTACTTCTTTCAATCATGTTCAAACTAAGGTGAGAAGAGAAAAGATAAGTAAGAAGATGAAGACCTTGCAAGCCATTGTACCAGGGTGTGACAAGATTACAGGAAAGGCTCATATGCTGGATGAAGTAATTAACTATGTCCAGTCTCTACAAAATGAAATTCAGATACTTTCTTTGAAGCTTGCTTCTGTAAATCCCATATATGATTATGAAGCAGACTTGGAGGCATTCACGGTTAAACCTCATCAGATTATGACCACCAACCAACAGCAGCAGCTTTCATTTCATGAATATCAAATGCCATGCATGTTATCCCAGAATCATGAAGAAGCATTATGGGAGTTGGAAGAACAACGACAAGGACTTGACGATGATCTTTTTGCTATCATCAACTACAATTCATTGTATGAGCCATATTCCTAG

AabHLH148

ATGGCTTGTTCATTGTCGGATTTAGTATCACTTGATACCAGATTCATACTTTACAATATGTGGGTGACGTTAATGAAACAATTAGGTGGTGAAGATGGCGGCTATAGTGGAGAAGAAGGCGGCGTAGATGTGGAGAAGAAGGCGGCGGTGAGAGGTGGCCGGCCGAGGCTGGAGGTGGCTGGAGGCGGGGTGAAGGGTTTCCAACTCGAAGTGCCAGATAATCCAAAGGGATTACCCCACCTGTCGTTACCTGAATTGGATGCATCAAGAATGATTACAACAATTCAGGCGGGTGCAGAAGAGTATAAAACAGGAAATAAGTGGGCAAAATCAGTTAACCACGTGGAAGCAGAGAGCCGGCGACGTCTCAACCAACAAATCTATGCCCTCCGGTCTGTGGTACCAAACGTGTCTAAGATTGATAAAGCTTGTATCCTGGCTGACGCCATTTACAATATCAATGAATTGAAAGGCAAAGTGGAATGTCTTGAATACCAGTTGCATTCTGGTAAAAACTACCTTCGGAAAATGAGGAGAGTGAAGAAACAAATGGCGGGTTTATAG

AabHLH149

ATGTATAGTCCAAGGAAGAGAGCCTCAAAGCATCAAGCTCACTGTGATGACGAACTTGCCGATCTCGTGCTGAAGTTACAAGCATTGCTCCCAACTTCAAGTTCCAAAGGTGATGGACGGGAAAAGATGGCAACTTCAAAGATTATCCAAGAGACATGCAATTACATAAAGAGGCTGCAAAAAGAGGTGAATATAATAGGAGAAAGATTATCCCAACTATTGGACTCCATGGAAAACGACGATCTTGACATGGACATTCTCAGAAACCTTCTGCAACAATAA

AabHLH150

ATGGGGGATCAGATGTCTTTTGACACCCTCTTGCAATTCATCAACTCATTGAGTGGTCAAATCGACCTTGACACAGTTCTCCGTGATGCAGAAGCTTTATGCCTGTGTGCCAGTGAGGATGGTGCAGCTTCCATTCCACCAGGAACTCCACCTTCATTGCCCGTTGAGGATGAAGAGTCCATATACCAACAAGAGGATGAGGTGTGTGCTCAGAAAGAGGTCCTAATCCGTAATCAGATGTTCTTCATGCCAAAATACCCTGATCTTATGATACATCATTACCATTTCTTTGAAGAAACCCAAGTGAAAGATACATTAAAATATCAGTGTAAATCTTGTTTTGTCTTTGTTCTTCAACAAAAACTCTTTGGTTTGTCAATGGCAGCACTCAGCTTCTACTCTAACTGGTCTACATTCCCACAAAACCGCTACGAAACAGTCGTACCTCAGCCACCCGAACTCTCGCCATTTCAAGACAACTTAGCCCTTTTCGAGTCGAATAATAACCATTTCTTCACAAACAACCTTTACCCATGTCAAAACACCATCAAATACCCTACTTTATCATCTTCTTATAATCCAATCTCACATCAACATTACTCACATCCACTCGCTGTTCCGAGTGTGTTTGATCATGAAGAGGTTTTTCCCATGGACTACCAAATGGAGCCAGAATGTTACTTAAATCCACTTGTGGACTCGAATGTATTGTTTGATAATGAAGTGTTCTCTCCTACGGAGGGAATGCAGCCCGAATACTACTCTTGCAATTCGTATCATTCATATCCACTCGTTAGTGAAGTATGCGAACAAATGCAGCCAGAACTGCCACCATTGCCCGAAATATACCAATTTGGTGGGGGTTCTAGTGATGTAATGCCTATGTCACATGATTTTGATAATGGGTGTAATGTTGTTCAAGGAGAGAGTACTTTACAAGTGAAGGATAATGGTGATGGAGGGAGGAAATTATCGGCTCAAAGTATGGCCGCGAGGGTGAGGAGGAGGAAGATAAGTGAGAAGACAATGGAGCTCGGGAAGCTAGTTCCGGGCGGTCATAGGATGACCACGGCGGAGATGTTTCAAGCGGCTTTTAAGTACATTAAGTTCTTGCAAGCTCAAGCTAGTGTTTTGCAACACATGGACTCATCTCCGGCACTCGGTGAAGAACTTCAAGCTTTTGTCACAAACCCTTCCATACAAGAGAAGCTATACACTGCGGAGAAGTGCATCGTAACAGAAAAACTTGGGAAAACGTTAACCGGTGATTATGAAGGAACAAATAACTAG

AabHLH151

ATGGGAGCAGAAAAATCACCAACATGCTTGCAGCCTAGCAAGAAGAGCCCTGGTAAAGTTCCTAAACGAATTCACAAGGCTGAAAGGGAGAAATTGAAACGTGAGCATTTGAATGAGCTTTTTCTTGAGTTGGCTGGTGCACTTGAACTCTCAGAACAAAACAGTGGCAAAGCCTCTATATTAGGTGAGACTACTCGAGTTGTCAAGGATATGGTTGATCAGATCAAGTCCCTCAGAAAGGAGAATGCAGCTTTACTGTCCGAATCACAATATGTTACAATCGAAAGAAATGAACTACAAGATGAGGCATCTACTTTACAGAATCAGATCAGTGTACTAAAGAGCATGATAAAAGAACACACTGTCCAAACAAACCTCGACCTGAATGCACCCGCAATAGAAACTCAAGAGCCACAGTTGCCACAGTATTTTCCACAGGACATTATAAGGTTACCTTCTGGTGATCCAGTTTTAAATCCTGTTTTTGTTATTCCCCCATGTCAGAACATCCAAATTAACCCACAACCCGTGTCAAACGTGAGTAAACCAAATCCAAGGTATCCCACTCCATCCGATTCCTGGCCTTTTCAACTTCTTGAAAAGCCGTCTCAAGAAGTTGGGGAGATACAACACAGGGAACGAGTTTGA

AabHLH152

ATGATAGATCTGGAGGCCTGGTGGAAAAAATTATTGTTGTTTGCAGGTGATGAAAACTTGAGTGACGAAACTATGTTACTTGAAACTCATGAAAATATGGTTCATGAACCAAAAGGAAATACTCTAGGTGAGGAGACGTCACTTGGTGCAAACTTAGTGACTGGCGAAATTATGGTTCGTAAACCAAGTGAAAATACTCTAGGCGAGGAGAAGTCACTTGTTCCATCTATAGCGACTAACGAAAAGTACGCTAAAAAACTTGATATGGGACTAATTGGTGGCCATGATAGTGGTCCAAATGAAGTGGATGATGAGGCAGTAAGAGAACATCCAAATGAAGTGGATGATGGTAGTGTCCGCGCGAGAAAAAAAGGAAAATTCGTTGTCACTGAGAATGATGGGACGGGACAAAGAGATCATCGAGCATCCAAAAAAGCGATTCATGTAAAGGCAGTAAGAAAACATCGAAAACTTTTGAATGGTTTGTTTAAAAATCTTCGCGTCTTGCTTCCTCAACTTCCAACTAAGATCAGCACAGAAACCTTAGTGGAAGAGGCTGTGAGCTCAATCAAATCTTTGGAGGAAACCCGTGATAGCCTTGAAAAACATAAACTAGAGAGGCTATCAACTGATACTAGAATGGCACCTCCTGCGCCATCTCAAACTCGAGTAATTGAGAATGCTGATTCCATGATGAACAAGGACGTGATCTTAGGTCCTACATCTCACTTTTCGAAAAACTGGTGTTCGTCGAACATCTCTTTGAGTGTGTTTGGGGCTAATGCCTTGGTTAACATTTGCACTGTGAGAAATGCAAACTTTTACAGTAGCATTTCTTACATCTTAAAGAAACACAATGTGGATGTTCTCGCCACCAGTATCCATTCAGATCAGGCCAAGACCATGTATATGATGGCACTGCGCGTCAATGCTCCAACCGAGATTGCGCACATGTTTCTGTACGAAGACTTGTTCAAGCTTGCTATGAACGAGATCGACTATGCGTATCGTCAAATTTGA

AabHLH153

ATGGCGTCTCTCAGCTTTTGCTCCAACTGGCCAACATTGTCACAACTCCGCCACGAAACCATTGTCCCTCAGCCACTTGAAATCTCAGCATTTCACGACAACTTTAACCTTTTCGAACCCAATAATAACCACTTCTCAACCTTCAACAATAACATCACTAACTTCCCAATTCAAAACACCGTCAAATACCCTACCTTGTCCGCATCTTATAATCCAATATCACAACAGCATTACTCTAATCCACTAAGTCCAAATGTATTTGATCATGAAGAGTTCTTTCCTATGGAGCAAATGCAGCCAGAATATTACTCGAATCCACTCTTCATTCCTAATGTATTTGATAACCAAGAGTTCTCTCCTATGGAGCAAATGCAAATACAACCAGAATATTGTTCGTATAATTCGTTTGATTCATATCCACTTGTTCCTGATGTGTGTGAACAAATGCATCCAGAACATTGTTTGTATAATTCGTATGATTCATATCCAATTGTTCCTAATGTATGCGAACAAATGCAGCCAGAACTACTACCCTTGCCCGAAATATACCAAAATGGTAGTGGTTCTGAAGATATAATGGCTATGTCGTATGATACTGGACATGGATGTAATGTTGTAAATCTAGATGAGAGTTTACAAGTGGCGAAGAATGGTGAAGGAGAGGGGAGGAAGTTATCGTCTCAAAGTATGGCAGCAAGGGTGAGGCGAAGGAAGATAAGTGAGAAGACAATGGAGCTCGGGAAGCTGGTTCCTGGTGGTCATAGGATGACTACCGCCGAGATGTTTCAAGCGACTTTTAAGTACATCAAGTTCTTGCAAGCTCAAGTTGCTGTTTTGCAACACATGGGCTCTTCTCCGGGACTCGGGGAAGAACTTCAAGCTTTGATCACAAACTCTACCGTACAAGAGAAGTTATATACTGCAGGGAAGTGCATCGTAACTGAAGAACTTGGGAAATTTTTAGCCGATAATCACCAAGGAACGAAAAATAATATTAACAATTGA

AabHLH154

ATGATGAGCTTCAAGATCGATTACTTCCATCTGATAGTTGCGGCTGGAAAAGGGGATCCTATGGTGGTGATGGCGGATTGCGAGTTTAAGGTTGAGCCTTATTTGTCGCAAACTTTGAGAATCAAGAAGCAGGAGGTCGAGAAGATGAATGAGATGATGTTTTTTAGATTCGCAAGAGGGTTCAGGAAGTTGGTTATTTTACTACTGGATAAGGAGGAGGGTAATGATTATCATAAAGAAAAACTCCAAGAACCATCAAAGGACTATATTCATGTTAGAGCAAGAAGAGGACAAGCTACTGATAGTCATTGTTTAGCAGAAAGAGTTAGGAGAGAAAAGATTAGTGAAAGAATGAAGCTTCTTCAGAATCTTGCGCCAAATTGCAACAAGGTAACTGGAAAGGTACTTATGCTTTATGAAATCATCAACTACATGCAATCGTTGCAAAGACAAGTTGAGTTCCTTTCGATGAAGTTAGCTACGGTGAACCCAAGTCTCTCAACTTTGACACCAATGACCTACTCTCGCAAAATAGTGCATTGGTTTCAAGAAGTTCCAAATGGAATGTATCATTACTTTAGTAAGGTAGTACCAACCATATACACTAATATTAGAGGCCGCACTATTCAGTCGAATCAGTTCTCTGTGACCGAACACTATAAGAGCCCAGAAGTTGGCAGACAGTCTCTTCCCGGGGTCTTCTTTTTCTATGACCTTTCTCCAATAAAGGTGACATTTACGGAAACATATGCCTCATTCTTACACTTCATGACCAATGTTTGTGGCATTGTTGGAGGAGCAGACCTTCATTGTTTCAAAAACAGAATCGAGCTAAAATGGGAGGATCCTGTTTGTGCTCCTGGTGGAAAGTGGACTATGACTTTTCCTAAGTCAAAATCTGACACATGTTGCCTCTATACGTTGCTACCAATGATTGGAGAACAGTTTGATCATGGGGATGAAATATGCGGGGCAGTTGCAAATGTTAGGTCAAGGCAGGAAAAGATAGCTCTTTGGACTAAGAATGCTTCCAATGAGGCTCCTCAGACGAGCATTGGGAAACAATGGAAAGAATTACTTGATTACAACGACACCATAGGATTCATATTCCATGAGGATGCTAAGAAACTTGACAGAGGTGCCAAAAATAAATACACAGCATGCGACATTGTATATTTCTAG

AabHLH155

ATGATAAGAAACAAAACAAAGGAAAATGCAAAGGCAGAACCACCAAAAGATTATATTCATGTTAGAGCAAGAAGGGGTCAAGCAACTGATAGCCATAGTCTTGCAGAAAGAGTTAGAAGAGAGAAAATTAGTGAAAGGATGAAGTTTTTGCAAGATCTTGTTCCTAGTTGTAATAAGGTAACAGGAAAAGCTTTTATGCTTGATGAGAGTTACGTACAGTCATTGCAGAGACAAGTTGAGTTTTTGTCGATGAAATTGGGGACCGTTAATCCTAGGATGGATGTGAGCATTGAATCACTTTTATCTAAAGATATGTTTCTGCCTCGAGTGTCTATGCCAACAAACATGAACCCTTTTGATGCCTCAGCACAACCATTTCCTTATTCATTCCAACCACAAAATAATGGCATTGTTCCTGATGTATCCGAGAACCAATTTTCCATGAACCCTTTGATGGCGGCAATGCATCGCAACTCACTCATGAAACCATCCCACGTTGATGGTCTTGGTGAGTGTTCTCAAGATGGCATTAGGACAGGATCATTACTCAACTCATCTTTCTATTCCTTTGTGGGGAATCCAGGTCTCTGCATCGATTATGTCCTTAATAACTGTGATGCTAACCTCACTAGAAACCTGAGACGCTGTACAGGTGCTTCTTCAAAAAAACTGAAAGGTCTTAGCAAGTTCCAAACTGGAATGGTAGCCCTTGGGACATCAGCATTTTTGTTTGCAATTGTTGTTGGAACTGGTTTCTTCATCCTTCAATGTCGGCAAAGAGAAAAACATGATACCGAAGAGATGTATAATGATAAACGAGAAGAGGATGACATCTTGTTTCAAAAAGTAATGGAAGCTACAGAGGACTTGAACGACAGGTATATAATAGGGAGAGGAGCACATGGAACTGTGTACAAGGCTTCATTGGGTTCTCAAGATGGGGTGTATGCGGTTAAGAAACTTATGTTTGGAGGAAGCAACAAAGAAGGAAGCACAAGCATGATTAGAGAGATCGAGACTGTTGGGAAGGTATATGCATATATGGTCCTGAGCAAAGATTTGGTGGGATTGGTCCGTTGA

AabHLH156

ATGTCTTCTGGATGTGGCCACCAGCAGAGAAAAAGGCGATCCGTGGGCAAGGGACATGCTGGTCATAATCCTGCGATTGAAAACATTCGAGCTAGGAATGGTGGAACTGAAGTAAGCAAGTTCAGTGTTATTTGGGACTTCCATCAAGTGAAGACCAGTGCACATGAATGTTACCGAAACCAACACGGTGGAGGTGATCCGGGAATGTGTCCAATTGGTGTGAAGGAACACTTAGCACCATACTTGCCTTCACACCCTGTGACTCAGAAAGTTGTTGCTAAAACCAAAAGGAATGTTAACTCGAAAAAACAAGAGGCAACCAACACCAATAATGGAGAAACTATATACGTGGTCGAGGAGAAAAAGCCTGAAGAAGGTACATATCAGATTCAATCTTTCGATACAGGTTTTTGGGTTGTACTTTATGCAAGCATTGCAACTTCAAGACTCTCTTTCAAGTATACAAGGGACATGCCATTGGCCAGATCCATACATTCTTATATTCAATTGGAGTCACCAGATACGGGTTGGATTGGCTTTTTCCCAATTGTTATCAGGGTTGAAGAGGTAAGTAGCGTGAAAGCAACTAAACAAGATGCGAGTAACGAAGAAAGGAGTTTATCAGCACAAATTTTGGCGGCAAGGGTGAGGCGAAGGAAGATAAGCGAGAAGACACAAGAGCTTGCGAAGCTAATTCTTGGAGGTCAAAAGATGAACACGGCCGAGATATTTCATTATGCTTTTAAGTACGTCAAGTTTTTGCAAGATCAAATTGGTGTTCTCAAACATATGGCCTTACTTCCGGATTACTTTGATTCTCAACAAGTTAATGCAACGAATCAGGAACCTGGAATGAAAAAGATGGAATCTAGCCTGAGAACTTCGAAAGCTTATGGTTCTTTAAGGGTGTTCATCTCTGAAAATATGACTGTAGGATTGAATGATCCTATAGTTAGAACCGAAGTAGCTGTCATGATGAAGGCTCATGAAGGAATCCGAATGGATAATGGTGATATTAGCGAGCGGACTTTAGACCTTGCGGAGCGGAAAATAAGAAGTAAAGTAGATCTTTCAAATGATAAATCCCATGAAGGACACCATTTAGCAGCAAGGCTGATTTTCCTCGACTCCCCTTTGATGCTTTCATCTAAGGATATGAACTTTGATGGTTACACATACAAGAACATTGAAATCGTTAATTATTATCAAGTTCCAAGAATGGGAGATCAATGCTATCTTGAGCGATTCAATGATTCATCCCAACATATCTCAATATGTCAACTTTACAACGACTTGCACAAACTTGGGTGTAGGAATGCTGTCATTTTAGCTATTGAGTCTATGGATCAAGGGTCACAAAGATTGCTAGAACACCTGAGCATGAAGATCAGATTGGTGCCTTGCTTTGGTTCTCACTTCTTTACTGTTCTACCAGCTTTGGATATACACACTACAATATTTGCATCCTCCTTTACAATGGAGTACGAGCTGAAAGGCTTTAAGGTTTGGTCTACGGATTTATATTTTGTGCACGTGAAGACTCAGAAGGCTCAACTACAACTGGATGCACAACTGCAGAAAGACGCCCGCATCATTGAACCCTGCTTCATCAGCATATACATGTGTTTCAAGAAGGCCAAATTTACAGCATTTGCACAGTTGGGAAAGGCACATGGTACTGTTCTGGGAAGAGAGCAGCGTCAACACGGTGGCAATGAGGATTCAACAGAAGGCGCCGTGTTAATATTGGTGCTGAAAGGGACCGAGGCCAAGAATGTAGTTGATGAGTTTTTGTTTGGATTAGTTGAGGATTTAAATATGTGGAATCAGTTTCCGTGGGGGTCGTATGTATGGCCAACATTGTATAGTAATCTAAAGGACACAGCAATTAAGCGTAGCGAATTGCACTTTGCCGAGGGGAGGGATCCAAATAATCTTCCTAAGTACATGTTGAACGGCTTTATATGGGCTTTCAAGGTAAGACGACCTCCACCGAGGCTTAGACCGGATGAAGTTGAGTTATTGAGCGAGTGGTGGGTTCATAGTAAAGCGTTGTTTGATGGGAATCCTCTCCCACCTCCAGTGAGACAGCCACAAGTAAACTCATTGATGGACGATGAAATTCCGCCGGTAATTTTGCAAAGGTTTAAGGTGTACGATGCGATGTTGGAACGACATCAATTGGAGTTGAAAAAACTTGCCGAGCTAAATAATCAAGCAATCCATAAAGAAACAAGCTTTGTTGGTGACGAACCCGGTTTTAGTTACTTTAGGGTGACTCAAGACACACAGGCAGGTCCATCTTTCGCCCCGGACATGGCTGTTGACACGCGGGTAAACTATTAA

AabHLH157

ATGACTACTGTGGAGTGTCGTGCAAATGCAGTGGAACCCAAGCACGAATTTGACTCCGCTCTCGCATTGGATATCGATAGAATGATGCTAACATGTGAGGGTAGTTCCAAGAAGCCCGAAAGAAAAACATTGGAAAAGAACAGAAGAATTCACATGAAAGGTCTTTGCTACAAGCTCAATTCTCTCATTCCTTCTTTGTCCTCACAACCTTTTAAGTTAACTACACAAGAGAACCAATTCGATCAAGCGACTGCTTACATCAAGCAACTACGAAAGAGAATAGAATTACTGAAGGAAAAGAGGGATCAAGCATTAAGATTGGTTAACAACGGGAGAAACAATGATGCATCGTGTAGCATTGAAAGCAAACAAAAGGCAGTGAGTTCATGGTTGCCCACGGTTGAGGTAAAAGAATTCGAGGGGGCTTTGCAAGTGTTTTTGACGAGCAATTTCGAGAGGAAGTTTTCTTTACCTCAAGTTGTTAGGATAGTAGAGGATGGGGGAGGTGAGGTGGTGAAGGGTGGTTACACTATTGTAGGTGATAAAGTCATTTATACCATCCATGCAAAGGCGAGAGTTACAAGAATTGGTGTTGACGTGACAGGCGTACACAAGGAACTACAAGAGTTAATCAACGGAAGCTCGTGCCGATCTCGTTTGAGGTTTCAAGGTTCGGGTTCGTGGGAAGTAGCAGGTTTTTTTTAA

AabHLH158

ATGGTGGTGGCAGTCGTCATGTCTTATGTGATGTTTGATCCAACTTATGAACTTTCACAAATAAGAACAGTGATAGTGTTGCGACACTTATCTCAACAGTATATAATGGTTGAAGAGGTATGTAGCCTGAAAGTAAAGAAACAAGTTGCGAGTAACGAAGAAAGGATTTTAGGAGCAAAAAGTTTCGCGGTAAGGGTGAGGCGAAGGAATATAAGCGAGAAGACACAAGAGCTTGGGAAGCTAATTCCTGGTGGTCAAAAGATGAACACACCCGAGATGTTTCAAGCTGCTTTTAAGTACGTCAAGTTCTTGCAGACTCAAATTGGTGTTCTCATTCCATATGGCCTTACTTCCGGTAATTTACAACAAGAAGCGATACAATATGATAACATTAAAAGTGGGAGGTACCATACTCTGATAACTTACGCATTCAGTCATGTTAGCTTGTTACATATGGTCGCAAACATGGTGGGGCTTTACAGCTTTGGCAAGAGTCTTGGACGAATAATTACGCCTGGAAGTCTGCTGAGGTTGTACATAGCTGGCGCATTAGGTGGTGCGTTCGCCTACTTGGTTGATTGTAGTTATCGAGCATCTGAGCCAGCACCATCATCAGAGGGCTCAAAGCCTCACCCTACAAAACGTATAGCAGTGGGAGCAAGTGGTGCAGTGACTGCTATGGTGTGCCTTGATATCTTACTTTTCCCAAGGGAAAAAGTATTGCTTTATTTCATAATACCAGTCCCTTCTTTATTAGCGGGGATATATATGATTTGGTTAGATGCAAACAGAGTAAAGTCATCATCAAACAATGCAGAACCTATTATGCCTCAAATCAATACCCAAATACCCAACACAAGTTCTTTCACAAACCGATCAAACACACTTACTCATCGTGGCAATATCTCTACCTACACTACTTCACCACAAGCATTTTCGAACCCAATTCCTGGAAACCGGCTTTTGACTAAAGGAAACATTGGAAGCAAGTTGTTTGTGGGTCATGAGTTTAGGCCTTTAAGACATCAATTCAGAAGACATTTTAGTATCTACTCAAAAAGATGGGTTTCAAAAATTCAATTCTCATCTTTCAAATCACACACTATGCATAAATTATTCTCCCTTAAACTAATCACCAAAAACCCATCAAAATTTACTCCAAAACCCTTCAAATCAAACCCAGTTTCACACCCTGTGACCAACAATGTGACCCATAATTACCACCACCATCATGGGTTTTATAGTCTGCCACAAGCATTTCAAGGAACACCCACCAACTGTTTGATCAAAGTCCTTTCGAACCCGGTTCGGTTTAACGGGGTTTTAGCGAAATTTAAGGGTTTTGTGGGTCAGGGGGGATTTGGGTCTTTGAGAGATCAGTTTAGGAGACATGGGTTTCAGTTTAATCAGCCTGTTAGTTACCAACAAACATGGTTGGCACAGTTCAGAAGGAGACTCACAACTGATGGTGTGGTAATAGGCTTGATTGTAACAAATGTGGCCGTGTTTCTACTGTGGAGGGTTGCTGACCGTAGGTTCATGGTGCAGAATTTTATGATACAATTGGACAACTTTAAAAGTGGACGCTTCCATACTATGATAACTTCTGCCTTCAGTCATAATGATGTAGGACATATTGTCTCAAACATGATTGGGCTTTATTTCTTTGGCAAGAGTATTGGACATCAGTTTGGGCCTGAATTCTTATTAATGTTGTACCTGGCTGGGGCTTTTGTTGGTTCAGCATTCTACTTGGTGCACCGTGCTTTTTTGGTTCCGTCATCAAAGGACAGACGTTTGTTTGAACCAGACCCTTCAAAAGTTCCAGGGCTGGGAGCAAGCGGTGCAGTAAATGCTATTATGTTGCTTGATATCTTCCTTAACCCGACAAAGACTATATATCTTGAGTTTATAATACCCGTCCCTGCTATCTTACTGGGGATTTTTCTAGTTGGGCATGATATGATGAGAATATTGGAGGACAGACGTTTGTTTGAACCAGACCCTTCAAAAGTTCCAGGGCTGGGAGCAAGCGGTGCAGTAAATGCTATTATGTTGCTTGATATCTTCCTTAACCCGACAAAGACTATATATCTTGAGTTTATAATACCCGTCCCTGCTATCTTACTGGGGATTTTTCTAGTTGGGCATGATATGATGAGAATATTGGAGGGAGATAGTAAAATTTCAGGATCAGCTCACTTGGGTGGTGCTGTGGTTGCTGCTGTTGCTTGGGCACGTTTGAGGAAGGTTAGAATTTTGGCGAAAGGTCTGGTTCAAGTTTTCCAGCAAATAATTGATCAAATTGGACGAGTGCATCAGCTGGTCATGCTAGATGATTAA

AabHLH159

ATGGATCCAAATTTGTATTTGATTTGGGACGAGGATGAGGATGATGCAAAGGTATCTGGAGACGGTGATTCCTCTGAGACAGTGACTACCCGTAATCCAAAAACAAAAAGAAGAAGTGGTGGTGGCGTGAAAGGTGATCGTACAAAGTCTTTGATTTCCGAGAGGAAGAGGAGAAGCGGAATGAAGGAGAACCTGTACGCATTGCGTACATTGGTACCCAACATCTCTAAGGTTAATCACTTTTTGCCCCAAATTATTCTGATGGTCACTACACCACAAATGGCTTTTAGAGACGGATCGATTTGGGGACAGCAGAAGGTTGTCACTAAAGGTTAA

AabHLH160

ATGGCTGATAATTGGTGGGATTCATCTTCAAGAACAAGACCTTCTTTGGATTCACTTTCTGTCTCCAACTCTATTAATCTTTTCCAAGATTCCGAAACTGCCACAGCCGCCACCACGACCACCACAGCCGCCACTAGCATGGGGGGCGGCACCGGGGCAAGCACGAGCTTACATATGATGGGGTTGGGACTTTCATCACCATCACTTCCACAATCGTTGGATTGGAATCAAGCTTTAGTTAGGGGAGATCAAAAAGGTGATGGCGGATTTCGTAATCTACTTGAAGATCAAGATCATAGTTTGAGCTCAAGCACGGACAATTTTCCCCTCGAAAATAACCAATGGAGGCAACAAAAGATGTATTCGGCTAGTTCACAAGACTCATCATCGGATTTTAAGCAAATAAATGTTCGGGGTTTTCAGTTGGATCAACCGATGCATGATAGTGATAACGAAAGCATAATCACATGCCAAGGGCTAAATTCAAGCTTCCAAAGCATGGATTTATATGGAAGTCCTTCTACTATAATGCAAAGTTTATTTGGTTCAGATAACAATCAACAACAACTAGGATCACGTCTTGATCAAAACCAAGGAATGAGTTATTCTTTGTATCAATCGAGCTATGGTGGCATAAATATGCCAGGTGGTGGTAACGGAGGTGGAGGCGGAGGTGGCGCGGAGTTATCAACATCTAATTGGTCAAAATTTCCACCTCAACCTCAAGAGTTTGTGGTGAATTCACCTTCAAAAGTGCAGTTGGCTGACATGAGTGGTAGCCAACTGCACTTTGCTAACAACGCACGATTTTGGAACCCATCGGCAGGAGGTGTAAACGATATCCGGCCTCCCTTTTTCCCATCTTTGCAAATGCAACTACCAACTTCAACCTTTGAAGATAAACCTAAGATTGCACCTAAAGTAGTGAAAGAAAGCACTAGCGAATCATCATCATCATCAACCAAAAGACCGCGAACTGAAAACCAATCACCTTTACCCGGGGCTTTTAAGGTGAAAAAAGAGAAGATGGGGGACAGAATCACTGCACTCCAACAATTAGTTTCACCTTTCGGAAAAACGGATACGGCGTCTGTGTTGTCTGAAGCTATTGAATACATTAAATTCCTACATGAACAAGTTAGTGTTCTAAGTACTCCATATATGAAAAATGGAGCTGCCATGATGCAACAACAGGCGGCAACTGACAAACCACCAGATGGACCTAGGCAAGATTTAAGAAGTCGTGGGCTATGTTTAGTGCCCGTTTCAAGCACGTTTCCGGTGACACACGAAACAACGGTTGATTTCTGGACACCTACGTTTGGTGGAACGTTTCGATAG

AabHLH161

ATGTCAAAAGATGGTTTCTTCAACACACTCCCACAATCTTGGAATTCCATGTTTGGAATGGAACTTGACTCCCAAGTTAATGAAATGAACCTTTTTAATCATAATTGGGAGAATTCAATGGATCAGAGCGATCCCTTTGAATCCGCATTAAGTTCTATAGTCTCATCGCCAGTAAACAGTCATCCTGGGACAGGTATCAGGACACCTGTCCCAGGATGCCATGCTGGCGCTGCAAGGTTTTCGTGTTTTGGTGCTAAGGAAGGTGAGTTTCAACATATGGTGGAATCTGGAAAGATGTCTAGAGTGTCTAGTAATCAGTCTTTTATTAAGACGGGTGGATCGGTTAGTAGGTTGTCGATGAATTTGAATTCGGATAATAAGGAAGTGGATCATTCTGTTGAGGTATCATCTTTGTCTGAGCAGATTAATGGCTCGGAAACCGGAATTAAAGGTAGAAAGAGGAAGGTGATTTCTAAAGGGAAATCGAAAGAAACCCAAGTCGATAATAAGGTTAGACGAGAAAAGATCAGTGAGAGAATGAAGTTCCTTCAAGATCTTGTCCCTGGTTGTAACAAGGTGACTGGAAAGGCTGTTATGCTCGATGAGATTATAAACTACGTACAATCATTGCAGAGACAAGTTGAGTTCTTGTCGATGAAATTAGCAACTGTTAATCCTAGGACGGATGTGAACATGGAAGCACTTTTGTCTAAAGACATTTTCCATTCTCGGCCATCCATGCCAAATCCCATGAACCATATGGAAGCATCAGCTCAACCGTTCTATGGCATGGTTAATGACGGGCCAGAGAACTCATTAATGGCCATGATGCATCATGGTTCAAATATGAAGTCATCACAGATTGATGGTTTCAGTGAAGCTTCCGCTTTCTGGGAAAATGACCTTCAGAGTGTTGTACAGATGGGGTTTGTCCAAAATCAAGGACCGAGCTTCCACGGAACAATGGGCTCGGGTCAAATGAAAGTTGAGCTATGA

AabHLH162

ATGGAGTCTGCAAATTTTCATCAACAACAACATCAAGAGCATCAGCCTGTACTTGACTCTTCTTGCTATGGACGTTCTTGGTCCCAAAATCCTAGCTTGAATAATACAAGCAACACAAACTCGAGAGAAAATATACATCTTGTCCCTTGTCACAACGCTAGCCCTCCTATGTTGGGATTACCTTGGAATACCACTACCAGCAGCAATGTGACAAGCAATCCTATTGAAAACTTCATGACCCATGAATTGCAACGCCTCGCAAGGATCAAAGATGAATTTTCAGCCTCAGAATCTTATCCAAGGTTTTCAGAGATGATAAATACTAGCCCAACTTCGAGTGTTGAAGATTTGCACTTAAACCCTTCTCCTGGATACCATAACAATCAGGACTTCTTCCTTAGAACATTCTCTAACGAGTGTCAAATAAAAGGCAGTCTGGTTATGGATCAAGTCCCAGATGACACCCAAAATAATCTCTACCAAAATTGTTCTAGAGGGACTTTTAGCCAGATTTTTCCGACAATAAATGTATCAAATTTAAATCAATCTCGAGCAGCCTCAGTTTCTTCAAACTCTTTTGATATGAACTTGCCAGCTTTGGATCTATTTGGTTCACCAAGATTCAACAGAAATTTTAGCCATCCTTCTTCATTTAATCCTCACCAACTTGGAAGCTTTTTCAAAGACACTTGTTTGTCATATGGGCTTGATCAGATGCATCAATCAAATAATCGACCAGCAATTTTCCCTAGCAAAATATCATCAGCCTTCAATACTAGTTGTACAGAAGCAAAGAGACCAGCTACCAATTACATGGACACAAAGGCTCCACAAGCCACGGTACCGAAGAAATCAAAAGTGGAGCCGCGTGCTTCTTGTGCACCTTTTAAGGTTAGAAAGGAGAAACTGGGAGATAGAATTGCAGCCCTTCAGCAAATGGTGGCACCTTTTGGGAAGACAGATACAGCTTCTGTACTCATGGAGGCTATTGGATATATCAAATTCTTACAAAATCAAGTCGAGACGTTAAGTGTGCCATACATGAAGTCGACTCAAAAGAACAACAGATTACCAACACAAGGGGTTTCACTGGAGGAAGGAAATGAAGAACCAAAAAGAGATCTTCGAAGTCGAGGGTTGTGCCTAGTACCCTTGTCATGTTTGTCATATGTTACCGATGGGGGTGGAGGTGTTTGGCCAGGCCCCTGA

AabHLH163

ATGGAGGGTTCGGATGAAAATGTCGACTTGGCTAAGAAGAAATCGGTTGGCAGGTCTTCTTCGAGCAAGAAGAAAGATGGCGTAAAAGTTCCTAGGAAAATTCGTAAAGCGGAAAGGGAGAAGCTGAAACGTGACCATTTGAATGACTTATTTCTAGACCTTACTAATGCTCTAGAACCGGCCACTCAGAATATTGGGAAATCTTCTGCATTGACTGATACTATGCGAATACTGCGGGACCTTATTGCTCAAGTTGATTCTCTTAAGAAGGAGAATTCTACTCTTTTAGCCGAATCTCAATATATTGCGGTCGAGAGGGATGAACTAAAAGAGGAAAATAGTGCCATAGAAGCCCATATTAAAAAGCTTCAAAGCCAGATAGACGAGAGAATGAATCCTCAGTCTTCATGGAGTTCGGAATGCAACCCTGTTGTCGGCCCTGTATTCATGTTGCCCCTGCAGAATGACCCAAAGCTTTATGCAGAACCCAAGATTCCAGAATTTGTAACAAAATCGCTTGGGCCAAATGTGAGCAAACCACATGCTAGATACCCATCAGCCTCTGATTCATGGCCACTGAACATTCTTTCTGAACAGTCAAGAGCAGCTTAA

AabHLH164

ATGTATGGTGTTAACAATTCTTCAGATGCAATATCTAGAGACATGAACTCAATCTTGTACTCATCAACTTTCAAACATCCAGCAGACACTGAGTTTGCTAAGATTAAACAGCTGATATCCTTAGACAACAACAATAACTCCTATGAAAACCCTAGTACTCATCCACAACACCAAGAAAACAGCGAAAATCCACCGTTAGTAAGTTATCGGTCTACTCCTAGCTCTTTTTTCTCGAATCTATTAAACGAGAATGAAAATGATGCCTTCCAAGATCATGAACCTGAGGAAATATATTTTATGGACCAGCAACAACATAAGAAGAAGTCTGATCAATCTGAAGCATATAATAATATGAAACGTGAGAAACAAGAAATGGGTAGTAAGAATATTGAAGTTTTGGGTTATGGTTATTCGAAACAAAGTGATTTGGATTGTGGATCGTCTTTTAGAAGTGATTTGGTTAGGCAAAGCAGCTCTCCTGCTGGATTTTTGTCCTCGTTGACTGGTGAAAATGCTTTTGCTAAAGATCTGAGAAACGGAAGTTCATCGAGTTCATTTAATAGCCACATCAGTTTCTCGTTAGGGTCATCTTCTTCTTCGTCGAGATTTTTGCCCCAAATAGCTGAAAACGAGAATGAGTTGAATGATTCCACATTCCATAGCTTGAAAAGGAGCAGAGATGGTAGCTTAAAGATGTCACAGAATGGAGAGACTGTAAACCACACTCCTAATTTGGTCCACCATGTGAGCTTGCCAAAAACGTCTTCTGAAATGGCTGCTGTTGATAATTTCTTGCATTTCCAACAAGATTCATCGGTTCCTTGGAAAACACGTGCAAAAAGAGGATTTGCTACACATCCACGAAGTATTGCAGAGAGGGTCAGAAGAACTCGAATCAGCGAAAGAATCAAAAGGTTGCAGGAGCTTTTCCCTGATATGGACAAGGTAAAATTTTCAAGCCTAAAACGTAAATCTTGTATTGAATTACTAAAAACAAGAAATCAAATGATCCATTTCATCTACGTATGCTCAAAAAACTGTAAACACTTATCAGAGATGCGTTTCAAATAA

AabHLH165

ATGTTTCATCAGCTACCAGACCTCACTTCATTTGAAAACCTTAACTACCCACCTTCCCTACCTACATTCCTATCTCAGCCACGGAAAATACCAACATCCTCAGCGCGCGAAAAACAGCAACAAAAACGTCGCACACTAAGCGAAAAAACACGCGCATTGCAGAAAATACTCCCTTGGGACAAAAAAATGGACATGGGCACATTGCTAGAGGAGACTTATAAATATATAAAGTTTCTTCAAGCACAAGTTAAGGTTTTGGAGCTGATGCCTGTTGATTCGTCTACTACTGGTTCAAATTTTGGTTGTTGTGTTTATTCGACTGAACAGTTGGTTATGTTTAAGGATCTTGCTGAGAAGAATTATGCTTCAATGTTGGGAATATTGGGTATGGTAAAAGCTAAAGGTGAGGTTAAGGGTGGTTTAGTAATTTTGGAGATTGCAACTGTAGTGGTAAATGAAAGTATAGACATTGTAATATCACAGAATTTAGGAACACTCCTAAAATTGACATCAACTAGGTGGCAGGTCTTATATAATAGGTCCATAAAACTGTATGTTATATACAGCGAAAAGATACTTTACATACTATGTGTCAATCTGAAAGGCAACCTAGCAAAGGATTCACGATTTATGTACCAATATAAAGGGCAACCTGGCAAAGATTTTAGTTTTCAAAATAGAAGATATCATAAATTCACATCTTTAACCATAAGCTACATCAATCAACAAAGACATGGTATACAGGGTGATCAACAATGTACATATATTATTCATGAACATGAACCCTTAATTCTGTGTGCTCTTCAATCACTAAACGTTAACAAGATCATCAAACTACACACAGTAAAGACACAATGTATCCCAAGTAGCAACATAATCAGCTCAACAAAGCAAGATGCATTATTGGAGATTCCCAAGTGA

AabHLH166

ATGCATAGACGGACCCAATTAGGAGTGAATGACAGTTGGGGGAAGCTTAGACAAACTGCTAGGGATCATTTGGTTATGGAAATTGGAGATGGTAATAAAGCTTCTATGTGGTTTGATTTAGCTCAGATTAAGACTGTTAATCTTGACAATGGGAAGAGTGATGTTCTGAAGTGGAAGAATAGGAAAGGAAAATTGGGGAAGTTTACTGTTAGTCATGGTTATAATGATTTAAGAGAGGATGAGACCGATGCTAATTGGTACAAGCTTGTTTGGTTTTCACAAAATATTCCCCAACATGCTTTTGTGCTATGGTTAGCTGTCCAGAATAAGTTGACTACTCAAGATGTGATTAAGAGATGGGGTTCATATGATATGATGTTAAAAATGGGAATAAGGGAGATTAATTTGTCATGGGAAGGCATTGTGAATAAAATGGCTGATTGGGGTCGCCGGAATCTGGGTTGGGGTCATCGGAATCTGGGTTGGGTTGGTAGTGGTGTTGGACGGAGATTGTTTGCCGGAGAAGTTCAAGAATATGAAGAGGACGAACTTAGCACAGCACGGACTCATAGAAATGATTTAAAAGATACTTCAGATTCTTGTTCTTCAAGGCTCGTAAAGAGCAGGCTGGCTTTATCGTCATTTTCCCATAAGTCAAGTGGTAGTAGTGAAAGAAAACGTGAAAAGATGAGGAAGATGGTGAATACAATAAGAGAAATTGTGCCTAGTGGAAAGCAAATGAATTCAGTTGCTGTTATAGATGAGGCTGTTAAGTATCTCAAATCTCTAAAAGTAGAACTGCAGGAAGTAGGTGTCGGAATTTGA

AabHLH167

ATGGCCAAGGAAATGATAGATCTGGAGGCCTGGTGGAAAAAATTATTGTTGTTTGCAGGTGATGAAAACTTGAGTGACGAAACTATGTTACTTGAAACTCATGAAAATATGGTTCATGAACCAAAAGGAAATACTCTAGGTGAGGAGACGTCACTTGGTGCAAACTTAGTGACTGACGAAATTATGGTTCGTAAACCAAGTGAAAATACTCTAGGCGAGGAGAAGTCACTTGTTCCATCCATAGCGACTAAGGAAAAGTACGCTGAAATAATTGATTTGGGACTAATTGGTGGCCATGATAGTGGTCCAAATGAAGTGGATGATGGTAGTGTTCGAGCGAGAAGAAAAGGAAAATCCGTTATCACTGAGGATGATGGGACGGGACGAAGAGATCGTCGAACATCCAAAAAAGTGATTCATTTAGAGGCGGTAAGAGAACATCCAAATGAAGTGGATGATGGTAGTGTCCGTGCGAGAAAAAAAGGAAAATCCATTGTCACTGAGAATGATGGGACGGGACGAAGAGATCGTCAAACATCCAAAAAAGTGATTCATTTAGAGGCAGTAAGAGAACATCCAAATGAAGTGGATGATGGTAGTGTCCGCACAAGAAAAAAAGGAAAATTCGTTGTCACTAAAAATGATGGGACGGGACAAATAGATCATCGAACATCCAAAAAAGCGATTCATGTAAAGGCAGTAAGAAAACATCGAAAACTTTTGACTGGTTTGTTTAAAAATCTTCGCGTCTTGCTTCCTCAACTTCCAACTAAGATTAGCAGAGAAACCTTAGTGGAAGAGGCTGTGAGCTCAATCAAATCTTTGGAGGAAACCCGTGATAGCCTTGAAAAACATAAACTAGAGAGGCTATCAACTGATACTAGAATGGCACCTCCTGCGCCATCTAAAACTCGAGTATTTGAGAATGCTGATTCCATGATGAACAAGGACGAGATCTTAGGTCCTACATCTCACTTTTCGAAAAACTGGTGTTCGTCGAACATCTCTTTGAGTGTGTTTGGGGCTAATGCCTTGGTTAACATTTGCACTGTGAGAAATGCAAACTTTTACAGTAGCATTTCTTATATCTTAAAGAAACACAATGTGGATGTTCTCGCCACCAGTATCCATTCAGATCAGGCCAAGACCATGTATATGATGACACTGCGTGTCAATGCTCCAACTGAGATTGCGCACATGTTTCCGTACGAAGACTTGTTCAAGCTTGCTATGAACGAGATCGACTATGCGTATCGTCAAATATTTAAAGCTCCAAAAGAGGATAATATGATGTGA

AabHLH168

ATGATCATGTTTCATCAGCTACCAGACCTCACTTCATTTGAAAACCTTAACTACCCACCTTCCCTACCTACATTCCTATCTCAGCCACGGAAAATACCAACATCCTCAGCGCGCGAAAAACAGCAACAAAAACGTGGCACGCTAAGCGAAAAAACACGCGCATTGCAGAAAATACTCCCATGGGACAAAAAAATGGACATGGGCACATTGCTAGAGGAGACTTATAAATATATAAAGTTTCTTCAAGCACAAGTTAAGGTTTTGGAGTTGATGCCTGTTGATTCGTCTACTACTGGTTCAAATTTTGGTAGTAATGAAGATGATAATGTTGGTTATGCTGCTTATGGTTATGGTACTTATGGTTATGGTTGTTATGGTAATGGTTATGGTAATAATGTTTATGGTGGATTAGGGAGATTGAATAGGCAACAGCTGTTGGAAGTTATTGTGAATTCGCCTGTGGCTCAAGCGAATTTGTGTTCCAGAGGTTGTTGTGTTTATTCGACTGAACAGTTGGTTATGTTTAAGGATCTTGCTGAGAAAAATTATGCTTCAATGTACTATTAA

AabHLH169

ATGCTTGCGGTAATCGAGACGAAAAGAGTTGAACCAGCGACTAAGATGATGGACCATAAGCGAAGCCCAGTGTCTATTGAGCAGGGGAGTCTTACTTCTTTGACACCAAAAAGACAGAAGGCCGGTTTATCCATGTCCTCCAAGGAAAGAAAAGAGAAGGTTGGTGAAAGGGTAGCAGCCCTTCAGCAGCTCGTCTCACCATACGGCAAGACAGATACTGCTTCGGTACTTCTTGAGGCAATGGAGTACATACACTTTCTTCACGATCAAGTAAAGGTGCTGAGTGCGCCTTATCTTCAGAGCGATCCAACAGATCAGTATCAGGAGTTAGACTCATATAACTTGAGAAGCAAAGGCTTGTGTCTGGTTCCGACTTCATATACAATGGGAGTTGCTAGCAGCAATGGAGCTGATATCTGGGCCCCAATCAAAACGCAGTCGCCAACATGA

AabHLH170

ATGGCAGAAGAATTTCAGGCAGCAGAAGTGAGTTGGTGGAATTCACCAAGAACCAACTTTAATGGATCACCATTTATGAGCACCTATGCTGGTGGCTGCTGGCAAAATGATTTTATGAACATCAAAACAAGGTCAACTGATGAGTCTTGTGGTGGTTACACAGCTACAATCTCACCGGATTCCGCTTTTCAGATAACGGACTCTGGCTCGGGCTCATCACCATCTACAAACACCACTTGGCATCAATCTTTATTAGTTAATGGAAGAAATGAAGAGAGTTACAACCAAACCCTACCAGAAATAATCTTGAATAACTTACCAGTCAGCGGTCAAGAAATTTCATCGAATTTTGCTATGAACCAGCAAGAGACTTCAAATTTCATGACAAATTCGGGTGATTGCACCGAAAACTTGGTTACGACTTCATATGGTTATCCTTCTAGTTTGTTACAAACTTTATTCGAAAGCGCATCTCCACCGACAGCAGCAGCACCACAACAACAAGCTCTCTATGATTTTGAAGCGAATTTGAACAATTTTAATTCGGTCCCAAGTATGCCTAATGGATTCTCTTCTATAGTAAAGCCAAAGCAACAAGTACTTGGAGGGTTACACTTAGCTAACAAGACGCCCTACTGGAATTCATCTGTGTTGGATTTAAATGATAATCGAGGTGGTTATATCGCTTCTACGCAGCCTAGATTTGGCTCCTCGACATATGAAGAAAAAAACAGTTATCCTAACATGAAGTCTCAAAATGAAGAAATTCGGGACTTGGGATCTTCTGTAAAGAAATCTTCTGGTGAACCTACATTCAAACGACCTCGTCTTGAAACACCATCGCCACTGCCAACGTTTAAGGTTCGAAAAGAAAAACTAGGAGATCGAGTCACTGCGCTCCAACAATTAGTTTCACCATTTGGAAAGACTGATACAGCATCGGTTCTCCATGAAGCAATCGAGTATATTAAGCTACTTCACGATCAAGTCAATGTTCTAAGTACTCCTTATATGAAAAATGGAGCTACCATGCAGCGACAACAAATTCATGACAAAGTGAAAGATGACACGGAAGGAGCTAAACAAGACTTGAGGAGTCGAGGACTTTGCCTTGTGCCAGTATCAAGTACCTTCCCGGTTACTACCGAGACTGCACAAGATTATTGGACATCCAGTTTCGGAAGCACATTCAAATAG

AabHLH171

ATGCCGCAAAACGAGGCTACAACCATTGACCCTATCTCCGGTGGAACCTTTTCCCGGCTACTCTTCTCCGACGCCGACGTTGATGATGCCGTAACTCTCCTCAACAACAACAACACCTTCACATTTTCTAGTAATGATCAAAAGCCACCAAAAATGTTGTGCTTTGGACAAGATAAATATGTCACACAAAACAATCCAACAACTAATGATCATGATCATGACAAATCTTTGCTACCCTCTTCAACCACCACTTCTTTGTCATCTAGTTCCAATATGAAAAGAAATATGGGATGTGACTATTATCAACCGGTTGTTCTTTCTACTAGTGTTGTCACCACCACCACCGGCGCCACCGTAGCTCCGTCGGGGAGCAACCGCCGGAGTAATAAAAAGATGAAGGCAGAAAACACACCTCCGGCTGGCCATGCAAAGGTGAAGAAAGAGAAGCTTGGAGAAAGAATTGCAGCCCTACAACAGATTGTGTCACCTTATGGCAAGACAGACACGGCATCGGTGCTACACGAGGCATTGGGATACATAAAGTTCCTTCAAGAACAGGTGCAAGTCTTGTGCTCTCCGTATATGAAGCTTATTCCGGCCACCCACAATGAAGGACAAGAAGACGGAGGCGGGCAAAGAAAGAAGGATCTTCAGAGCCGAGGGCTATGCTTGGTCCCGGTTGAGTGCACCCTCCATGTGGCAGAAAGCAATGGTGCTGATATGTGGTCAGCTGCAATGGTCAATCACCATGGCTCATCAGCACTGCGTTGA

AabHLH172

ATGAGTTTCATTGCATTCGTAACGGGAAGTGGAATTGAAGGCAACGTGTGGTGGTTGAAGGTGAATAAGCGGCGGCGAGGATTTTCAATGTTAAGAGAACTCATCCCTCATGGTGGTCAAAAGAGAGATAAGGCCTCATTTTTACTAGAGGTATATCACGAGAGGCATGTTTACTTTGTTCACCCAAAACTTACATATAGGCATATAGCTAAGCCTAAAGTTCTGGTTTGTCAGATTATTGAATACATTCAGTTTCTACAAAAGAAGGTACACAAGTTCGAGGACTCATGCCGGGGATGGAATAATGAACAACCTGCGATGACCGCATGGGTAATAACCTGCCACAAGTAA

AabHLH173

ATGATTAATTCAAATAGACCTACAACACTCAATAAATTGTCCTGCCTTTATATTCCAAATACCCCACCCCCATCCCCTAATCAACATAAGTTTCAGAGCTTAATGTATAAGTTTATAATGGCTGAACAATGCAATCAAAACTCTTCTTCAACCGCAAAGTGGTGGCCAGATGTTCATGCGAGTTCGCTTTGTTCATGGACTGGTGGTGCTAATTATGCAAGTAATAATCTGTGCAATAACTCACAAACCCCTAACTCCAATTGCTCTAATGGTGAAGAAGACGTTTCTATCTCTACTTCTTTTACAACGAATGCTTCAAACAACTCTGGGCTCAGCATGGAATCCTCTCGACGGCTTGTAGAGAAAGCTTCCACCAACGATCCTTATGGAGAGGCAGTTTCCGATAATCATCATTTATGGAACCAAGTTTTACTAGGTGTGGGAACTACCGGGGAGCTGCAAAACACATCATCTCAAATGTTTGAACCAGCGTGTGATTATTTGAAGAAAATCGACAATGGATGGGACTTTTTAAGTTCAACACATCCTAACCAGTTCCAAAAGAATTTTAATGGTGTCAACAACGGTTTATACCAAAACAAGATTTCACAAGATGTTGTAATAAGCCCACAGCCTGATCAATATAATGGTCAATTCACAGCAATAAAAAGTGAACGCATGGAGAGTGATGTTCATCGTGAGGGGATATTTAGGAGAGGGTTAAGTTGTCATGCAAGTGAATATCAAGCCGGGACTAACGACGTTGTTTTAGAAGATAGTAACAAGTATTATGACAATGGTATGTCGTCGGATATGGAGTGTACGAATGGAAGGGCTTTTCTGGATTTGGTTGGTTACGGTAGCTTCTTAAACAAACCAGTATTGGATCTTGAGAGTAACGTGTTTAATAAACCCATGATGAGTACCATGAATCTACCAGACAGAATGAAGCAAGGCATCCATAACTCTTTTCAGCCAATAAGGCCAAGAGGCAACACACCAACTAAAAGTAATGAAAGAGGAAATGGAATTGCTAATGAAGGGAAGAGAAAGAAATCAGAGGACCAATCTGGGTCACTCAAAAAACCTAAGCTTGAGACTTCTACAGTCTCATCAACTAAGGCTCAGCTTCCTAAAGCAAAGCTCGGGGAGAAAATTACAGCCCTTCAACAAATTGTGTCACCGTTCGGCAAGACAGATACAGCTTCGGTATTAGGGGAAGCGATTTGTTATATCAAGTGTCTACAAGAGCAAGTACAGTTATTGAGCAATCCATACATGAAGACCAATATCATCAAGGACCCATGGGTGCGACTGGAAACAAAAGATAGAGGAGATATGAATCTTGATCTTAAGACTAGAGGTCTGTGTTTGGTTCCCTTATCGCGTACTCCTCAAGTGTACCATGAAAACAACGGATCAGATTACTGGACTCCAGCATATAGAGGATATATCTATAGATAG

AabHLH174

ATGAATAGAGGAAATGTGTTGCAAAGTTCACCGGTGCAACAAATGATTGCCGGCGGTCCTAACGGCTGGTGGAGCATGATGGCTACGACTAGGCCATCTACACCACCTCCTCCGCAACAACCGTTCTTTGCTAGCTCTGCACAACCCCACTTCTTTCCTCACCAATATGCACCACCACTAACAATACCATCTTCACAAGCTCCATGGCATAATAATCAAGAAAATCCGGATTCCTTAAGTCAATTACTCATGTCTGGATTAGTGAGTGAAGAAGACAAGTCAGCTTTGAGCCATATGCAACAAGTTAAGAAATTGGAGAACTGGGAACAACAGCTATTACTTCGTGATCATCATCGTCAGCAGCAGCAACACAACTCCTTGAACGCGCAATCCGTGGAAGAATCTACTATCAAGCAAGAAAACTCACTAAACTACGGATCATCGTATGGTCAAGGAAACGGCGAGTTTCATGGCACAAAGCCAAGTAATTGGTCTAATCAAATGATCCCCGTTTCATCTCCTACTTCTTGTGTCTCAAGTGTGAGCAACAGTATGCTCGATTTCTCTACCACTACTAGTAACAAGGCGGATGGAAGACACCCTCCACCTGATCGCTCATCTGAGTGCAATAGCACTGCTACAGGTGGGGCATTAAAGAAGGCTAAAATACAACCTTCTTCAAGTCAATCTACCTTCAAGGTGAGGAAAGAAAAATTAGGGGATAGAATAACAGCACTTCACCAGCTTGTTTCTCCATTTGGGAAGACTGACACAGCTTCGGTCCTGTTAGAAGCTATTGGTTACATAAGATTCCTTCAGAGTCAAATTGAGGCCTTAAGCTTGCCATACCTCGGTGGTGGATCTGCTGGAAACATGAGAAACCACCAACATTCCGTTCAACAAGGGGAAAGAAATTGTTTATTTCCTGAAGATCCTGGCCAGCTTGTGAATGATAGCTGCATGAAGATGAAAGGAGCTGCTCTTGATCAGGATTCTCATGAAGAACCAAAGAAGGATTTGAGGAGTAGAGGGTTGTGTCTAGTCCCTGTGTCATGCACAATGCAAGTAGGTAGTGACAATGGAGCAGATTATTGGGCTCCTGCACTTAATGGAGGGTTCCGGTAA

AabHLH175

ATGGATGATTTGAATGAACATGTTCCTCAAAATGATTCTGATTTGCAGCCGTACACATCTACTTTACCAGAAACTTTTGGTTATGTACCCCGATTAGCTGAAAATACTTTCTACCAGCAGCATATAGTCCAAGCAAAAGATTGTGATATCTATTCTGTTCCTCAATGGATTGGAGAGTATCTTGCAGGAAATGGGCTTTCTAATATGCAAGGACAAATACCATATGACAGCACTGGAATCGGATGCAGTTCTTTCACTGAGGTCCCTTTTCTCAGCATGAATGGTCATCTGGCAGAGCCAAGAGACATTAACTCACATGTAAATTTTCTGAGCCAGCAACATGAAGAAGTGCCCGTGAACAATTTTGATACTACACAATATGGAATAGGCTATCAGAAGCAAGGGCCAAATACAGGCTTAAATAATGCTGCTCAACATCTGGAAAACATGTCAGTGTCCACCTCAGGTAGCGGAACCAAATCAGGCAAAAAGCGTGTGAAATACCCTGAAACAAATTGTCGTCGAAAAACAAGGCTTACAGCAGCACTAGATGCTTTGGAAAATGTGCTTCCTCGGTCTAGAGAGGTAAACAAAGAAACTATTGTTGATGATTGTGAAGACTATATAAAGTCTTTGCAGCTTCACATGAAGGAATTGAGCCAGAACAGATTGAGATGTGAACCCACTTCTAATCACTTGCAGTACCTTGAGGGATATGGCCATTACCTTGTTCATGAAAACACCGCTACTGGACCTTTAGATGACATACTGGGAAAGTTGTTGAAGGAGAACCCAACCGCAGCAACCAAGCTACTGGAGAGTAAAGGTCTTTTCATGACGACTAGTACTCCTAATTAA

AabHLH176

ATGGTGTGTCAATCAGCCGGTCAAACAAGATTCCGGGCATTGAAACATGAAAATGGAATTGATGGGAGTGCCACCATAATAGTTAAAGTTATTGCATGCTTTCAACCTCTACAAGATTGTCAGCATTTTGAATCCTGGTTTCAAAACCAACAATCTGATCCAAATTTGTCGAGTGCTCTCTTCAGCTTTCAGCACCGACGCAACATACCATATTTGGGGAATACACTTTCACCTAACACGATCAACATAAATCTCCCCATGTTTGCGTTTTCAGCAAGCAATCCGGAAGAACCACGTGATTGGTTCAATGGCTTGGCCCCGATGGCCAAGTCTATCCCGAAACAGCAACTTCCGGAACCTCAAGCAAGCAAACCACAAGAACCATGTGATTGGTTCAATGGCTTGACCCCGATGGTCAAGACTATCCCGATACAGCAACTTCCGGAACCTCAAGCAAGCAAACCACAAGAATCATGTGATTGGTTCAATGGCTTAACCCGGATGGTTAACTCTATTCCGAAACAGCAACTTCCAGAACGTCAAGTCACACACGGGTTACAGAAGAAGTTTCTTGTGTTTGATCAATCAAATTATCGAACTACATTGATTTATAGCTCAACTCCAGCTCAGTACCAAATCCCCAAACCACAACTTTTCTTTAACGTGAAAAAGGAAAGTTCAGTAATTGAAAACCACTTGGATACCCATTTTTCAATCCCGTTTCCTGATGATGGAAACACTACGGCAAGTGAGATGCAAGAGGATAGCGAAGAGCTTAAGGCATTACTCTACTCTGATGACGAGAGTGATTATTCTGAAGATGAAGAGGAACAAAGTACAGGGCATTCCCCTGATTTGATACCGGGCCTCGATAGACATGAATGTAAGGATGATAATCTCGAAGAAGTGGCTAGTTCAACGGGTTGTAGGAAAAGGCCTAAACATGAACTAGAAGACACTGCAAGCTCAGGAAAATCAGGCCTTAATTGCTCTGGTGACGCAGATCTTAGTAGCTGTGGAAATAACTCTGGTGTCAGAAACGGAGTGTCTGATGTTGAATCAGAAACTTTCCCGCCAGTTAAGAGGGCGAGAAAAGAGAAACTGTGGGAAACGATAAACATTCTTCAGAATTTGGTTCCGAGTGGGAAAAGTGACAATAAGGATGCCATGGTCATTCTTGATGAAGCCATTGATTATTTGAGAATTTTGAAGGTGAAAGCTATAGCCTTAGGGCTTGATTCTCTTTAA

AabHLH177

ATGTCTCCATCAATAAACCCTACTGATCATTTCAAGAATCAATTCGCCACTAGATTCGTTCATGCTCTAAACAATATAAACGCCACAAAAAGTTCACCTCATGATGATCACAAAAACATGTTTCAAAGATCTCGTCGAGTCAAGATAGCAGCATACACCGCCATGGCTTCTGTCTCTGGATCAGAGAGAGCGTGGAGCCGTGCGTTGTTATGGAAGATCAGAAATCGATCAAGAAATCGCGGTTTGTTGCTAAGGAATAAAAAAAGGGCTGATCATGCTAAGGTGTCTTCAAAGAAAAGAAACCCTAACCCTAAAAGACAAGATGTTAATGCGCTTAGATACTCGGGTCAAGAATTGAAGCTGAGGAAAATTGTTCCTGGTGCTGAAACCATGGATTCACGTTCTTTGATGAGTGAAACAGCTGATTACATCAAGTGTCTTGTTGCACAGGTAGAGGTCATGAAATCCCTTGTGGATCTCTGCTACACAAAAGTTTAA

AabHLH178

ATGGAATTGCCTCCTCAAATTGGAGCACTGAAAAAACTCAAGTTGTTTGATCTTGAAGGAACCGAGCTCATGTATCTACCAAAGGAAATTGGCAAATTAGAGACATTGGAATGTTTAAGGGTCTCTTTCTCGACATATGCAGATGACCAAAAAGACCGAAGCGGCGTAGAGCACATAATCCCAAGGATGACAATTTCAAAGCTCACAAAGCTCAAAGAGTTAAGTATCAGTGTCAATGCGGACAATGAATGGTGGGAGGTTGAACTTTTAGAAGGAATTATGGGGGATCTTATTTTCTTACCCGACCTTAAAACTATAAAGCTGTACTTACCAACTGCTAAAGCATTGCAAGAGTTCTTAAGCCTTGAAAGGTATAAAGTACCAATATATTCAAATTTGTGGAATTTTAGGTTTATGATTGGTCGCTGTGAAGAGCTTCCATGTTCTGTACAACTTGATATCGAAGAAAATTTTCTGAAATTAGAAAAATGTGTAAAATTCATGAATGGTGATGGATACACGGATGAAACTGCGGAGTTAGTTAGAAATGCTAGAGCTCTATACTTGCGCCGTCATTGGACTATTGGGAAGCTCCCAATATTTGACATGAAGAGAGTGAAATATTGCTTGTTAATGGAGTGTAATGAGATGCAAACCCTTTTCGATCAAGAAGATGTATATGCACACCTAGATAAAGCTACTAACGATGAAGACGCAAGTCTAGCCTCCTTGCAGTATTTGGGGGTTCACTTCATGAAGAAGTTACAACGGCTTTCAAAGGGGCCAATTAGCAGTACAAGTCTGTCCCATTTAAGGATCTTAGCACTGCATAGCTGCCCTGAGATGAGTAGCATTTTTACTGGAAGTTTGCTTCAAAATATGCAAGGCTTGACTGAACTTGTAGTAGAAGATTGCCCCAAATTCAACTGCCTTGTCAATTTGGAGGATGGTACCCCTTGTAGTAGTGGTCCATTTCTTCAAAGTTTAAGGAGGGTATCACTTATTGACTTACCTGAGCTAGTGAGTATATCTGGTGGTGTCAGTATTGCGCCACAGCTTGATAGTTTACTTGTTTTTAATTGCCTAAAACTCGACTATCTTTCCATCATGGAATTAACTAGAGATGTAAAGGAGATCAAAGGAGAGATTGAATGGTGGGATGCATTAAAGTATGGAAAAATGACATGGAACAATGTCTTTGTGCCACTCAAAAGAGATGGAAATTTGTTGGATTTATTAGCACAAGATACAAACTCTCTCCAACATTTTCTTGAGCTTTTTACGGCCCCTTCACACGCTGGTAGCATTTTGCAAGTAGACCAAAACTCGTCAAGAGACCATGTTGATCAGCTTCAGATTGAACATGATGTGCTCCTTTCTAATGAAACTCAAAAAATGTCCTCTCAGGATATTTTCAACTCTAACAAGGCCACTCAGTCACTAGATGCTGGAACAGCGGTTAGAATGTGGGAGCCACAGTATCCAGTGATTGGAAAACTGGATAATGAAGATATGGACTGTGATTTTGATGTTCCTAATTACAAACCTGCCTCTATAAAGAATAAGCGCAGAAGTATCAGCTTCATCACGTCGAAGCACCAAGTAAAAGTTAACAAGGGGATAGAAGTTGAAAAAGCCCTTGATGATGGCTATACTTGGAGAAAATATGGCCAAAAGGAAATTCTTAATGCCAAATACCCCAGAGCATATTATAGATGCAGCTTCGGAAGTACACATGGTTGTTGTGCAAAGAAGAATGTTCAAAGATCTACTGAGGACCCATCTGTCTTTGAAGTCATGTACATTGGATATCACACCTGCCCAACGATATTAATGACTGCTACAGAATCATCTTGTAGAATTTTAAGAACAAAAGATTCATCAACTGCAACTGAGTCGGACTCTACGAATACTTATAGTCGTTTTGAACATCCAAAAAGACCTCAAATGCTGGAGTCTAACAAAATAAGTAAAAGTGGTAGGGGCGCTAGATTGACTCAGCACGTAGGAGTTAGCCCAAAGACTGCAGGGTTTGAAACACCACCTGATGATGGTTATAAATGGAGGAGGTATGGTCAACGTGAAATATTAGGAGCAAAGTATTCCAGTAGCTATTACAGATGCACAAGTTGCAGTACAAAGAAACGAGCCCAAAGATCTGATGATGATCCATCCATCTGGGAAATCACTTACAAAGGAACGCACACCTGTCTTGGACCTTCAATAGCCAGGAATTTTGCAAGTACAACTGAATCAGAAATAAGTATCATAAAAAACAGTGACTCTTCGACTATAACAAAATCAGCTTCTGTAATCACTGAGAACTCTGTTGGACTGGAGTTTACAAAGAACATGAACCTCCAATCATCCACGAAGGAACTCCAGAACTCAAAACGGAGGCCGTCACCATTTTCTAAGGCAAGGAAAGAGAAGTTGGGGGACAGAATTACTGCACTTCAGGTGTTAGTTTCACCTTTTGGAAAGACTGATACAGCATCAGTGCTGTTTGAAACTTGTGCCTATATCAGATTCCTACATGAGCAAGTCAGCGTTCTTATGACCCCATACACAACAAAAGGAGCTCCATTAGAGCTACAGCAGAAGGTTACAAAGCGAGACCTAAGAAATCAAGGGCTATGTTTGGTACCTGTTTCAACTTGCGAGCACCTTGCCGGTTGCTATAGTAACTATTCTGATTTCGCAATCTCTACTTCTGCTAAAAATCCCAACGAGTCACTCGCCCATGAACAACTTTTCCAGATGTAG

AabHLH179

ATGGGCATGGAAGCAGTGGCAGGAGATGGCGACAACGACGATGAAGAGATCATATTGAGTTGGGGCGAGAAATGTGGTAATGTTCCCTTTCTTCCTGATTATGGCCCCCTCTCCGTCACTGACTGGGGATTGCTTAATAGTTCTGGGATGAGCTACGGTGTCCAGCCATTCTTCTCATCTCAATTTTATAACTTTGGAAACATGAACGAAGCATCCTCCTCGTCTACCTTTCCTGAGGAGCTTGAGGACATGATACAGGAAGAGAAAGTAACTTTCTATACCAATGACTTCCGACTAGAACCAGAATTGTACAACTATTACCAGCAGATTGATCATAGAGCCGTTGATGTTCCTCTGCAAGGCCCAATGATAGACTATCAATCTTCAGGTGTAAACAATAATCATGGTGCTATATTACAGCAACCATTCACTCCCGATATGAAACATTCAGATTGGAGATATGAACAAAATTCCCGAGTAATGGAGAATCCTAAGAAACGTATGAACAATGTTCGTGATCAAAAGGTGGTGAACAAGAAACATGCACGCCATAACAAAATGCAAGGAATCGAAGGTGTAACAGAACGGGTGCCGATGAGAAGAAGCCAAAAGCTAGCTGACAAAATTACAGCCCTCCAGAAGCTTGTCTCTCCTTATGGCAAGACGGACACGGCTTCTGTGCTCCAAGAAGCTCATATATCTATCAATCTCCTTCATGGCCAAATTCAGAAGTTGCTGCAGAGCACACAGACTCCAGCGATCAACATTGGCCCAATCCAAAATAGGAATAACAAGGAGGCCGAGAGCAGCTTACGAGATAAAGGCCTATGTTTGGTTCCAGTGTCAACTCTTCAAGTCAACAGCATATACCACGGGGAACAGAATTTTATATCCGGAAACTACTAG

AabHLH180

ATGCACAACTCTAGCCAACTAAAGAAGGAATTTATCAAGAATTGGATGAAGGGTCTTCACATATGTTATTCATCAAATAAGCAAATGAAGCTCTTAGAAAGAAAGAAGAAAATAAAGATATCTGCAGACATTGCTTTAGCTTGTGCTAAAAATGCAACAACTTCTTGGAGTAAAGCTCTAATTGCCGAAGCGAAAAAAGATGAACAAAATAAGATACTTATTGACAATCTAGCATGCCCAGAATCCGAGATCAAGGGTTTTCACCAAAAGGTCATGACTTGCCACAAGAGAATTAGATGCAAGAAAATCTTGAAAAGGAGTCATGGTGTAGTTAAAAGAACGAAGAAGTTGAAGCCTCGTCGATCGGATTTAGCTATCCGTGTTGCTAAAAGATTGGTAAAGAAGAGAACCCAAGTGCTAAAAGGGCTAGTACCAGGTGGAGAATCAATGGATGAATTTTCGATTATCAAAGAAGCATTAGACTATATACTTTCTCTTAAGGTGCAAGTTGATGTAATGAAGAACCTTGTGAATGCCGCCAATGTCCTAAATTAA

AabHLH181

ATGGACAACAATGGTGGATTCACTAAAGAGAAATCTAGTTTGCGAACCGAAAGGAAAGTAAGAAGGACAAGTAGAAGAATCAATACGACTGTGCTTAGGAAGGTGAAGAAGTTGCAAAAGCTTATACCAGGAGGCAAAGGGTTGAATGCAGATCGACTTTTCGATCATACCGCGAGTTACATTATGCATCTCAAGCTACAGGTTGATGTGCTTCAAGCTTTGTCTGATGTTTATCGCCCCTAA

AabHLH182

ATGATGGCTTCATCTTCGGATCCGATATCAAACAATATCGAACGAGTACGTGATTCGTCGAAAAGAAGGAAAAAGAAGAAAATCAAAAGCAATAATGATATAGCAAAAAATAAAGATGAGATTACACAATGGAAATCAGAAACTCAACAACAAGTATACAGCTCAAAACTATTACAAGCTCTTCGTCACGTCCGGATCAGCTCAGGGGTGGCGCTGAAGTCAGCGCCACATCGCGGACGTGCAGTCCGCGAAGCAGCTGATCGAGTCCTAGCTGTAACAGCTAAAGGAAGGACTCGATGGAGCCGAGCAATTCTTACAAATAAACTTAAACTTAAGTTTATGAAAAAGAACAGAAGGCAAAAAGGGAGTATTGTTACACCAACAAGTAATAGCCGAATAAAAAAACCGCGAGTTAGTATTCTAAGGCTTAAGACGAAGAATTTACCGGCTGTACAAAGGAAAACACGCGTTTTAGGACGATTAGTTCCTGGTTGTAGGAAACAATCGTTACCCGTTGTATTGGAAGAAGCAAGTGATTATATAGCTGCACTTGAGATGCAAGTTAAAGCTATGGCTGCTTTAGCTCAACTTTTATCCGGTGGCTCCGGCTCAAATTCTGGAGCCACCGGTGATATGATTTCGAGTCATCGGCAGCCACCACCAAGCTTATGA

AabHLH183

ATGTCGTCGTCAACGGTATCTCAGATTCCATTACGCGATTCATCTAAACGAAGGAAACGAAAGAAGCTTCAACAGCTGCAATCGCATAACAACGATCAGATCACGAAACAAAATAATAAAATCCAGTGGAAATCAGATGCTCAGCAACAAATATACAGTTCAAAGCTCTTACAAGCTCTCCGTCACGTAAGGAGATCCACTGGCACCAGTACCGGCACCAGCAGTACTACTGCTGGTGCGGGTACTAGTGCCAGGAAACACGCGGTCCGTGAAACAGCAGACCGCGTGTTGGCAGTAACAGCAAAAGGCCGTACACGGTGGAGCCGTGCTATACTAACAAATAAATTAAAAATAAAAAATTTGAAGCGGAGTAGGAGAGAGAGAGGCTTAATAGTTTCTTCAGCCGGCTCTGCTAATAGCCGGCTGAAGAAACCTAGAGTAAGTATATTACGCTTAAAAACGAAGAATCTACCCGCGGTTCAAAGGAAAACACGTGTTTTAGGCGGTTTAGTGCCGGGTTGTAAGAAACAATCGTTACCTGTTGTTTTGGAAGAGGCTACTGATTATATACCTGCACTTGAGATGCAGGTTAAAGCTTTGGCTGCATTAGTTGAATTATTATCTGGTGGTTCGAGTTCGGGTTTGAGTTCGGGTTCGGGTGCTGGTGATGGTGGTGGTGGATTAAACTTGTTGAGTTACAGGCCACAAGGGAGATTGTGA

AabHLH184

ATGTCTCCACCTCATCAACCCACATCCATAAACCCTAATTCCCTCAAGATCCAACTTGCCTATCGATTTCTTCACAATCTAAACAACATAAACACAAAAAGATCCAATCTAGACCACACCCAAATCAATCGAAAGTCTCATCGTGTCAAGGTCGCAGCCTACGCATCGATGGCTTTTGTAACCGGATCAAGAAGAGCTTGGAGCCGCTCTATTTTACGCAAGATTAGAAACCGAGGCGTGTTAGCAAGAATCAAGAAAAGGGTTGATCATAAAGCTAGTCGTGTTCGTGTTCATCATCACCTTAATGCAAGGTCTACAAAGAGAAGAAACCCTAGTTGTTCAAACCCTAATGGAGACTATATCGACCCGAGTGGTAATTTGGGCCTTGAAGTGAAGCTGAGGAAGCTTGTACCAGGTGCTGTAATGATGGATGCATGTGGTTTGTTGGATGAAACTGCTGATTATATAAAGTGTCTTGCTACACAGGTAGAGGTTATGAGAACTTTGGTAGATCTTTACTCTACCATATGA

AabHLH185

ATGCCAAGAAAGAACTCAAAAATGGTGTCTAGGGAGAACAAGAAAGCTGCACTGCATGACAAGCTGCAACTTCTTCGATCTGTTACAAACTCTCATGCAAAGGAAGATTCATCAATCATAATTGATGCATCAAAGTACATACAAGAGCTCAAACAGAAAATAGATATGCTAAATCAAGATGTAGCTCAAAGCTCAAGCTATCAGAACGCATGGCCTATGGTTACGGTAGAAAACCTAGATAAGGGTATACAAGTGAATGTGTATTCAGAAAGAAGCTGTCCAGGTCTAGTAGTTTTTGTGCTGAAAGTGTTTGAAGACTTAAATCTTAATGTGTTGGAAGCAAGAGTTTCTTGTACAGGCAGCTTTCAACTTGAAGCCCTTGGAATTGAGGTAATATATGTTTTTTTGTATGTTATTGCCTTATTGATTGCATCAATGATATCAATAGCTTCGATTCTGGAAAAGAAAAAACAAAAATATAATTTAAAGTGGTTCAATAAATTGCTTAAGATTAAGGCGTTGAAACATATATAA

AabHLH186

ATGCCAAGAAAGAACTCAAAAATGGTGTCTAGGGAGAACAAGAAAGCTGCACTGCATGACAAGCTGCAACTTCTTCGATCTGTTACAAACTCTCATGCAAAGGAAGATTCATCAATCATAATTGATGCATCAAAGTACATACAAGAGCTCAAACAGAAAATAGATATGCTAAATCAAGATGTAGCTCAAAGCTCAAGCTATCAGAACGCATGGCCTATGGTTACGGTAGAAAACCTAGATAAGGGTATACAAGTGAATGTGTATTCAGAAAGAAGCTGTCCAGGTCTAGTAGTTTTTGTGCTGAAAGTGTTTGAAGACTTAAATCTTAATGTGTTGGAAGCAAGAGTTTCTTGTACAGGCAGCTTTCAACTTGAAGCCCTTGGAATTGAGAATGATGAAAATGGAGAATGCATTGACAGTCATTTGGTGAAACAGGCGGTCTTACAAGCAATTGAAGAATGGAGTGAAAGCAATGATCAAGAATGCTAA

AabHLH187

ATGCATACATCAAGCAAGCTAAAGAAAGAATTTTTTAAGAAATGGATGCAGGGTCTTCAAATATGTTGTTCATCGAAGAAGCAAATGAATATCATGGAAAGAAAGAAGAAAATAAAGCTTTCTTCAGATATTGCCATGGCTTCTGCTAAAATGACACCAACTTCTTGGAGCAATGCACTAATTTCCAACGCTAAAAAATGTGAGCAAGACACTGTTCTTGTGGACAAGTTAATAGGACCCCAATCTCAGTTGAAGCTACAGAAGACTAGCAATAAAATGATTAGTTTCCATAATAGAGTTCAATGCAAAAAAGTCTTGAAAAGGAGTTGCAATTATGCTGCAAAAAAGACAAAGAGGATGGGAGATTCTAGAAAGAATCTAGCAACTATCATTGCGAAAAGATTGGTGAAGAAGAGGACTAAAGTGCTTAAAAGACTTGTGCCTGGTGGAGAATCCCTGGATGAATTCTCACTCATTAAAGAAGCATTGGACTACATCCTGTCTCTTAAGGTACAAGTTGATGTGATGAGGAGTGTTGTTAATGCAACTGAGGTTGTTTTGAATGGTGATAATTTGATGAAGTCTGTTTGA

AabHLH188

ATGTCAACCACAAAGAAGAAAATGTCAAGGCAATATAGTTGTGTTAGACAACAAGCAATGCAAAACATAAGCAATAACAACGAGTCCACGTCATGGAAATCAAAATATCAACAAGAACTATACAGGTCGAAGCTCGTACAAGCTCTTCGTCAGATTCAGCTAAGCTCTAGTCCCCTTAGCGGACATGTTGTTCATGAAGCAGCTAACCAAGTTCTTGCAATGACGGCCAAAGGGCATACTCGATGGAGCCAAGCAATCCTTACAAATAAAGTGATACGTAAAGCTCGACGAGTTGTTGTTCCCACAATGGTAGCTAAGAGAAGATTTAAAAAGCGGAGAGTTGGTATTTTGAGGTTGAAATCTAAGAATTTATCGGCCGTGCAAAGGAAAGCACGCGATCTCAGACATTTAGTTCCAGGCTGCCGAAAACAACCTTTGTCTGTTGTGTTAGACGAAGTAACTGATTATATTTGTGCTGTTGAAATGCAAGTTAAAGCCATGGCTACTCTAGCTGACCTTTTCTCCAGTATTAACCAGCCACCATCGAGCTCATGA

AabHLH189

ATGGAGTCTGCTAATTTTTATCACCATCAACAACAAGATAATCAACTTGTGGATACTTCTTTTCATTGGTCCCAAAATCCCATCTTGAATGGAGCAAATAACAATATAAATTCAAGAAATCTTGACACTAGTATCAATTCCATGGTTGAAGACATGGGATTTCCCTACAATGGTATCGGATATCCGATAGACAACCTCATGACTCAAGAATTGCAACGTTTGGCACGAATCAAGGAAGAGTTCTCAGTTGCCGAATCATATCCAAAGTTTTTAGAACTTTTAAATGGCAGCCCAACAACCTCGAGTATTGAAGATTTACGCTCACAACCTTACAGTACTACGTACATGAAGACTGACCACCACGATCAACAAATTAGCTACTCAAATTATAATCAAGATTTCTTGCTAACAAATTTGCTTAACGGATGTCAAATCAAAGGTGGCCAGCTTGTAAATGATCAACGTAGTTCTAATGGAACACTTAGCCAGATTTTTCCAACTATAAGTATTTCAAGTTTGAAGCAATCCACCACCTCATCATCATCATCGTCATCCTCAGCAATTTCTTCAAGCTCTTTTGATATGAACTGCTTACCAGCTTTAGATCACTTTGGTTCTCCAAGGTTCGATGCAAGCTTTAGTCGTCCTTCCTCGCTTAACGCCAATAATCTTGGTGGCTTTCCGTATGGTCTTGACCGTATGAATCAACCAAATCACAAGCCAGCAGTTTGCCCTAGCAAGATATCATCGGTCTCCACCACTGGAAGCTGTACATATCAATCAGCAAAGAGGCCAGCTAGTAAGTATATCGACGAAAAAGTGACTCAACCCATAGCACAAAAGAAATCAAAATCGGAAACACGCGCTCCTTGTGCACCCTTTCAGGTCAGGAAGGAAAAATTAGGGGACAGAATTGCAGCTATTCAGCAGATGGTGGCACCTTTTGGCAAGACTGATACAGCTTCGGTATTAATGGAGGCTATTGGATATATCAAATTTCTTCACACCCAAGTCGAGACGCTGAGCGTTCCATACATGAAGTCAACAAACAAGATTTGCGGAATATCTTCACAAGGGGGACGAGTGGAGGATGGAGTTACGACAAAGGCAAAAAGAGATCTTCAAAGTCGAGGATTGTGCTTGGTGCCATTGTCATGTTTGTCGTACGTTACAGATGGATGCGAAGGCATTTGGCCACCACATTAA

AabHLH190

ATGACACTTTTTCTCTTTCAAAAGGTAGGCGTGATCCATTCAAACCACAAATGTGCCTCTAATCGAGTGGGACGAGACTCAGAACGTTTAGACCTGAGTGTCTTAAGGACTGAGGTGTCTGGTTCAAACCCTAATGCTTTGGCTGACCATCATCAACATGAAAACCAAGATTTCCCTGTCTCATGGAGCCAACTACTCATGACTGGATTAGCTAATGATCAAGAACAACACAACTTGGTTGCTGGACATGTTGGTGAAGTAAAGCATGGATATTTGGATAATCAACAAAGCAGACAACCTTTTTATAGTCATAAAAGTGATCATCAAGATCAAAACAATAGTATCGAAGATGATCAACAAGGTTGTAGCTCTACATGGTCATCGCAACTCAATCCGTATGATGATACATCTACTAGCAGTGGGGCATCTAAAAAGCCAAGATTTCAATCTTCTTCAACACAAGCTTCTCCTGTGGTTAGAAAGGAAAAATTGGGCGACAGGATATCGGCTTTGCACCAACTTGTTTCCCCATTCGGAAAGACTGACACAGCTTCTGTATTGTTTGAGGCCACAGCTCAGATCAGACATCTTGAGGGTCAAATTGAGACACTTACCTCACCGTACATGAACTTAGCCAATAATGTCTCAGGAGCCACGAGACACCAACATTCTTCTGATGGGAATGCATTGCAAAGGAATCTCACTAGTAGAGGACTATGCTTGGTTCCGGTGAATTGCATAGATCATATTGACACTTCGAATATGACCAACAATGGCACAGAATTTTGGACACCAGCACTTGGAGGTGGACTGTAA

AabHLH191

ATGTACACAAAATTAAGTATGCTTAACTTTTTTGTTGTAGGGAATATAAAAATGCATACTTCTAGCAAGCAGCTCAAGAAAGACTTTATCAAGAAATGGGTAAAGGGTCTTGAAATATGTTGTTCTTCAAATAAGAAAATGGATGTTATGGAAAGAAAGAAGAAAATAAAGCTTTGTGCAGACATTGCTTTGGCTTCTGCTAAAAATGCCACCACTTCTTGGAGCAATGCTCTCATTTACAATGCTAAAAAAGATGACGAAAACGCCATTCTTGTCGATAATTTATTAGGACCCGAGTCACGGTTCAACTCACAAAGGACTGCTCATCAAATGATCACTTTCCATAAGAGGGTTAGAAGCAAGAGGATCTTGAAAAAGAGTTGCACTGTTAGTCAAAGAATGAAGAAGGCAAGTCCTCCAGCGTCGAATTTAGCTACTTGTATCGCGAAAAGATTGGTAAAGAAGCGAACCCAAGTGCTTAAAAAACTTGTTCCTGGTGGAGAAGCAATGGATGAATACTCCCTCATCAAAGAAGCACTAGACTATATACTTTCACTTAGGGTGCAAGTTGATGTCATGAGAAGTCTCGCAAATGCCACCGAGGTTTTGGATTGA

AaHLH1

ATGGAGCTTTCGCAACCCCGTCCCCGCGGAGAGCCAGGTGCGAAACCCACGCATGACTTTCTTTCGCTTTATTCACCTGCTCACCAAGATCCGTCCCCTACCATTCCAGGTAGTTACCTTAAAACGCACAACTTCTTGCAACCACTAGAACAAGTTGGAAAGACTGTTTGCGAAGAAGTGGAATACATTAAAAAGTCTTTCCCACCATCTCCACCAACTGTTGGGGAACACATTCTACCCGGTGGGATGGGAACTTACAGCATTAGTCACATTCCGCATATTAATCAAACCCAAAAGGTATCAAAGCCTGAAGGGATTGTGATATCGGCTGCACAGTCAAGTAGTAGCAATAACAATGATGAAAATTCAAACTGCAGTTCTTACACAGGGAGTGGTTTTACGTTGTGGGAAGAATCTAATGTAAACAAGGGAAAGACGAGGAAGGAGAATAATATTGCCGCAAATAGGCATACAATGAGAGATGGGGGTATGAAGTTTGGAGTTCCATGGATGACATCGATTGAGCAGCCATCAAAGTCATCATCCATAAATAATCATCCGAGCACAACTCGATCATCAGCCCCTAAGAGTCAGTACTTCGTCGATATGAAGTCTGGTAGGAATTTCCAAGAAGTCGAAGACATTAATGAAGGATTTTCAATGTTGAGAGGGATCATTCCACATGGAGATCAAAAAAGAGATAAGGCGTCGTTTTTGCTAGAGGTTATTGAATACATTCAGTTTCTACAAGAGAAGGTGCACAAGTATGAGGACTCATCTCAAGGATGGACCAATGAACCACCAAAAACAATTCCAATCAACGACTTCATTCATCAACCCCAAATCCCAACAAATCAAAACCTATTAGATTCTAACATGATCTCTCATGATGCTACTAACGAAACCAGTCAACACCCACAATCAACAAATAAACCATCTGCTTCTGCTGCAGCCTCTTATGAACAGATTTCCACTCCTGATACAGCAACCACAGCCTCACAGGCTCAAGAACTGATAATTGAAGGTGGCACAATTAGCATTTCAACCATCTACTCTCAAGGATTAGCTAGTGCTCTAACGCAAGCACTAAATAGCTCGGGTGTGGATTTGTCACATGCTAATATCTCGGTACAAATTGATCTTGGAAAGCGATCAAATGCAAATACAAATACACTTGAATCCTCACCACACAATCTCAAGGAGAATGAGACTCGTTCCAATGATCAGTCAATAGCACATTCGAGACTTGTAAGCACATGGGAAGACGAGAAAGACCAAGGTTTCAAGAGGTTGAAGACAAGCAGAAATTGA

AaHLH2

ATGAGGAGATTCCAGATATTGAGAGATCTGATACCCAACAGTGATCAAAAGAGAGACACTGCATCATTTTTACTAGAGGTGATAGAATATGTTCAGTACTTACAGGAAAGAGTACAAAAGCATGAGGGATCATACCAGGGTTATAGCGCAGAGCCCACAAAGTTAATGCCATGGAGAAACAGTCATTGGCGTGTCCCAAATTTTGGTCATCCACCCGTCCTAAGGAATGATTCCGGGTCGGCCCCATCTTTGCCTGTTAGATTTGATGAAACTGTCTCGACCATCAACACGCCCCCACAAATTCCTGCTCGCTCTGACCCTAGTGGAGATCTGAACCGTAATTTGATTGATTCACAACCTGATTTACAAGCAAACATTCCTGTCCCTGATGAATATGTTCAGTACTTACAGGAAAGAGTACAAAAGCACGAGGGATCATACCAAGGTTATAGCGCAGAGCCCACAAAGTTAATGCCATGGAGAAACAGTCATTGGCGTGTCCCAAATTTTGGTCATCCACCCGTCCTAAGGAATGATTCCGGGTCGGCCCCATCTTTGCCTGTTAGATTTGATGAAACTGTCTCAACCATCAACACGCCCCCACAAATTCCTGCTCGCTCTGACCCTAGTGGAGATCTGAACCGTAATTTGATTGATTCACAACCTGATTTACAAGCAAACATTCCTGTCCCTGATGGTACACTCTCTCATTCTACCCTTGGGGGCTCAGTCTGTCACGTCAATAGGCAGTCAACCGATATCCCTGCTGCTGGTGATGGGAATCAGCAAGATGAGCTAACTGTTGAAGGTGGCACAATTAACATTTCAAGTGTTTATTCTGAAGGGTGA

AaHLH3

ATGGATATAGCAAGCAAGTATGAGGTGAAGGCAATGCCAACATTCTTGTTGATAAAAGAAGGTGTTGTAGTTGGGAGGCTAGTTGGAGCAAATCCTGAAGAGATCAAGAAAAGGATTGAGACACATCTTCAATCCAACACTCATTTGATCGACCACTTTCCGGTTCCTGGCGGTGAACTTCCTTCCCTTGAACCGGGTTTTCACTGGTCTGCTAACTCTTTTCCCGGTTCAACCAGCGTTGTTAGGTTTCAAGAATTGAATGAAATACTGGACCCTGGAAGGTCAGCTAAGACGGATAAGACGGTTATTTTGGCGGATGCTATTCGAATGATCACACACTTGAGAAATGAAGCCACAAATCTTAAGGACTCATCTCAAGATTTGCTCGTTAAAATCAATGAGCTGAAGGTTGAGAAGAACGAGCTGAGAGACGAGAAGCAAAAGTTGAAGACAGACAAAGAGAGACTAGAACAACATCTGAAATCCACCTTTTGTGGTCCTCCAACGGCATTCTATCCTCCTGCACATCCTGTAATGCCGGTCCCATGTCCAGGCCCCACCCCCGTTGGTGGAAACAAGTTCATGCCATACATGGGATTCCAAGGGGTTCCTATGTGGCAATTTGCTCCCCCAGCTGCGGTTGATACTTCCAAAGATCATGTTCATCGATCACCACTTGCATAG

AaHLH4

ATGACTACTTCTAAGTTTGTTGAATTGGCATTAATCCTTGAACCTGGGAAGCCCCCTAAAATTGATAAGGCTGCTATATTGGTTGATGCTGTGCGGAAGCTTGCTCAGTTAAGAAATGAAGTCCAGAAGCTGATAGACTCAAATACAGAAATTCAGGAGAATATTAAATAG

AaHLH5

ATGCATCAACAGCAGCCTACCCCTCCTCTTGTCCCCATGCTTGCTGACTATGGAGTAGCAGAAATAACATGGGAAAATGGCCAACCAGCCATGCATGGGCTAGGAAGAGCAAATGAGACACTAGAATCAATTGTTCATCAAGCTACAACATGTTACAACCAAACTCAATATCCAGAAATCGACTTACAACAAAGTCAAAGTCTTCCAAGAGCTCGCAACTTAAGCTCGAATGTTGCATCATCGAGTCGCCCGACTTACCTAAGGAAACGGCCTAGAGAATCTGTCATTATCCATGATCAATGTGTAGGAAATTTGGGTAACGCAAGTTTGCAAGAAGATAATGTTAGCAATAGTGGGACGGTTAATTCTAAAGATAATGATACTACAATGATGACATGGCCTTCATTCGACTCGCCTAATCAAAGCATGAAGAGCCAAAAAACAGATGATGATTCTGCCTGCCAATATGGATCGGAAAATCAAGAAGAAGAATGTAGGACTGAGGGTGAAACAATTCGATCTCAATCAAGTCGACGAAGCAGAGCAGCTGCTATTCATAACCAGTCCGAACGGACGGATAAAGCATCGCTGTTGGATGAAGTGATTGATTACTTAAAAAAGCTACAATCACAAGTACAATTGATGAAGAACATGCCGTTTACACCACAACAAATGATGATGTCAATGCCCCTACAATTGCAGCAACAACAGCATCAACATCAGCAGCAGCAGCAGCTTCAAATGTCGATGCTAGCGCGAATGGGAATGGGATTCGGCCTTCAGATGGGAATGCCTGGAGTTATACCTCAGCCAGTTCATAATCCATTCATGGTCCCACAAACCATGCTTAGCCCAGCCCATGTAGGCACCACGTCACAAACTATTCATAGTCGCCCATCTACCAACACCCCAGTTCCTTTCAACGATCCACACAGCACGTTTCTAGCACAACAAATGAACATGGATATGTACAACAACATGGCAGCTTTCTATCGGCAACAGGTCAACCAGGGAAAATCGATGAGCGTTGACTCATCTCAACTAGACCATGTTCGGGGAGAGTGA

AaHLH6

ATGAAAGAGGTAGAAGTTAAAGAGGTTGATACAGAGGAGGAAGGGAAACGGAAGAAAGAGAGAGCAGCAAAAGTAGACATTGAACCAGAGGCCGTGCCAGGGATAGGATTTGGGAAGGATAGCGGGACGGAGGTTATGTCCTTAGTGGCAAAATCTGATAGTGCTTATGAAGGTGGCCCACCTGCTGATTACTACTACTCAAAATCGTACTTTGCATCCAGATTTGTAGAATTAGGCTCTACTTTGGAACCTGACCAGTCTGCCACCACCGATAGGCTAGCTATTATTGGAGATGTTATTCGAGTTCTAAATCAATTAAAATCTGAATTTCAGGAGTGCAAAGAGATGAACGAGAAACTATTGGAAGAGATCAAAACATTTAGGGCAGAAATGGTTGAACTGCGTAAAGAATAA

AaHLH7

ATGGGGTCTTCAATAATGTTTAGCGAGTGTCTCAACTTTGAAGATTGTGGGGTATCCTTACTAAGATTTGTAGAATTAAGCTCTACTTTGGAACCTGGCCGGTCTGTAACCACCGATAAGCTAGCTATTATTGGAGATGTTATTCGAGTTCTAAATCAATTAAAATCTGAATCTCAGGAGTGCAAAGAGATGAACGAGAAACTATTGGAAGAGATCAAAACATTTAGGATCATTGAGTTCAGCTCTGTCCCGGTTACTGTTAGTAGTGATGCTGCTGAGAAGCAGTCTATTCCCCTTGTGGCCTTAAAGTGTATTTTAGAATTACTTTTTGAGTAA

AaHLH8

ATGGCTTTTGTTTCTTTCAAATTTGTAGAATTAGGCTCTACTTTGGAACCTGACCGGTCTGCCACCACCGATAAGCTAGCTATTATTGGAGATGTTATTCGAGTTCTAAATCAATTAAAATCTGAAATTCAGGAGTGCAAAGAGATGAACGAGAAACTATTGGAAGAGATCAAAATATTTAGGGGAGAAGATGAATTGATTATGAAAGAAGGGGCAAGTAATGACCAGAATGTTCATTACGGAAATTGTTTTGCTTTTTGTGTAGACAAGGCAGAAGTTGTAGGTTATTTTCATCTGTACGATCTGTTGGCAATGTGGGAAAATAAATTGGGTCGCCCTCCCACCAAGAAACTTTTAAGTCGCAAAGCCCCACATCAAAAGTATTCTACATGTACAACTACTACGGAGTTTCTTGGTACAGATGATGGGCATGAAGAGGTTTTGGCTGCAGTGAATGCTACTATTAAGCCTGGTGGTTGTGCATTGTCCAGTCCTTTCTGGAGGTTAATGGATCCACTTTTCGGTTTCGTATTTGATGCAGGTGTGGCTTACGTAAAGCAAGAGGTGATGGCCTATTAG

AaHLH9

ATGAAGTCTTTTTACTCTAATCTCTTTTCACTCATTCCTCCTAATGTCTTCTCCAAGGAAGGTGATGTGTCGGATCGTGTGGATAGAGCCATAGAATACATTCAAATGTCTAAAACTCACTTGGATATGCTCAAGAACAAGAAGGAGAAGTTGTCTGCTAGTAGGAAGAGATCACATGAACACACAAAAATGATCAACTATGTGTGCAAGCAGGTTGATGTTCAAATCCATGAAATGAGTCATGATATTGATGCTGTTTTGGTAACAGGATTTGAGAACCATTCGAGCTTTTGTGACGTTCTTTGGTTACTAAACCGGTATAGTGCTGAGGTTACACTAGCAATGTTTTCAAGCAACGGGTATTCGACCTTTCATATCCGTAAAAAAAAGATTGAAGCACAAGATATATGTAAGAGGCTCATGAGTTTGCTGGAAGGGTCTTTAAATGTGAAGGAGTTGGAGAATAATCATGCATTGTTTTGCGTGCCCGTAACACTTCCTCAACCAGCTGGATGGGACACCCGAATGGAAAGTGGACCCCAAAGAATCGAAGAATCACATATAAATTTACAGAACGAACTTAATTCTCTATCATGCAATGAACTCGAGTATGATTCGAATTTAAGTATATGGGACCTTGATTTCCAGTCAAATGTATGGATTACTTGCAATGAACTCGACTATGAGTCCAATTTAAGTATGTATGGGGATCTGAGTTAG

AaHLH10

ATGACGGCTGGCGCTCCCAAGTTTTACCCCCTTACCCCACCTCCCAGCAGTCTCCCCTCAAGAATTAATGAAGAACTTTGCGCCACCACAATGCCTAGCCCAACTGGCGCCTTATTGGATGTAGCTTCAGTTCTTGGAGATGCCATTAAGTATCTGAAGGAGCTTCTTCAGAAGATCAATGATCTTAATCATGAACTTAAAGAAACCCCGTAA

AaHLH11

ATGACTACTTCTAGGTTTGTTGAATTGGCATCGATCCTTGAACCTGAGAAGCCCCCTAAAATTGATAAGGCTGCTATATTTGTCGATGTTGTGCGGAAGCTTGCTCAGTTAAGAAACGAAGTCCAGAAGCTCATAGACTCAAACACAGAAATTCAGGAGAATATTAGAGATGAAGGTAATCCCATTGCCATGTTTTTTCTATACTATACATCTATTACAAATGAGAAGACCGACTTGCGGGATGAAAAGCAGAGGCTAAAGGCTAAGAAAGAGGATCTCGAACAAAAAGTCAAATCAATGAACACCCAACCGAACTTTATGATTCCTCCACCTGGAATCCAGGCAGCAAACAGTTTCACCAGGCATATATCAAGTTTTGTTGCACCTTTGTTGGCCTGTGATATAGCAAAGCCGACGGAACGCTTTGGTGTCTGCTGGATATTCACTTCTATATTTTATGACAGGAAGGAGGTGTTTGCGATTACGTCTTCGTTATACCATTCTTGGTTCTATGATGCCCGCAAAAAGTGCAACAAGAGTGCGAACCATGTCAAAAATGGTGCTTCTTCATCCGGTGCAACCAAGAACAAGAACGCCAAGTCTGCCAAACAGGCCAACACGTGTGATGAACATGGTATCATTTACCAAGTTGTGCCAAGGTTTTAG
