# Supplementary material for: Genome-wide identification and characterisation of bHLH transcription factors in Artemisia annua
Source: BMC Plant Biol. 2023 Feb 1;23:63. doi: 10.1186/s12870-023-04063-8 (PMC9890702; doi:10.1186/s12870-023-04063-8)
Supplement: Supplementary file 3 — Additional file 3: Supplemental Material 3. gDNA sequences of 226 bHLH TFs. [file 12870_2023_4063_MOESM3_ESM.docx]

**Supplemental Material 3 gDNA sequences of 226 bHLH TFs**

(Red letters represent exons)

AaMyc-bHLH1

AGCAACTAATTCCGCCCACTCTTACAATACCCGAGATATTTACTTGCTTTCCATAAAATATAAAAATACAAAAAGAATCATCCTTCACCTCTAGACCTAGTTATATTAGTTTCAATCCTAATGAACACTATTCCCTGAGAGAACGATCAATCCCCCGGTACTTGCCACTCACTATACTACATCGGTCAGGTACACTGTCTGGTGGGGATTGCAAGCTTCTCAAGATCCTCTGAGAAGTAAAACCTTGTGCTTAGATCGCAATCCACGCATCATCTTGATAGTTATACGCCGTATGTCAAAAGTGTAATGCGGCGTAAGGTAAAAGATGATGATAGTGATGATGTGTTGATTGATGATTTTACGTTGACGTGAAGACAAGAATACAATTAACCATTGTGTCAATTCTAATTAATGCTGACCATATATACACTCAGCTCTTCATACATAACGTTACCATAGGTTCGAAAGCAAAATCATTTCTAGTTACTATTTTGTGTAAAAAAAAAAAAAAAAAGGCCCCAATAATATGGCGTGAAAAACACACACACCTGATGTTCTCTCTCACCCACTAATAATATTCAGTTTTATTTCTCTCTTTCCTCATCTCTTCCTTGAATCTATTTCTTCCCTAACTACTCATCAGCCGCCATCCATCCACCCAACCTAACACTTTTCATACCTTGTTTGTACGGTTAATGGAATGACGATGAATATATGGAATTCAGATGATAACGCTATGATGGATGCTTTTATGAGCTCCGATATGTTTTCTATATGGGGAACTCCAGCAACCCCAGCTGGAGCTCCAGCTGGGCCAGGACTAGTTGTTCCACCAGCTTCTTCCTCTGCTTCAACTTCTGCCGCGATTGGCAGCGAGTGGAATCAGGATACTTTGCAACAACGTCTCCAAGGATTGATTGATAACGCGCGTGAGTCTTGGACTTACGCGATATTCTGGCAGTCTTCTGGAGTGGATTACACAGCTCCTTCTGTGTTGGGGTGGGGTGACGGGTATTACAAAGGAGAGGTTAATAAACCCAAAACTGTAGAATCTGTTACCTCTTTGGCTGAGCAAGAGCATAGGAAGAAAGTGTTGAGGGAGCTGAATTCGTTGATTTCGGGTTCTCATAGCCAAGAAAATGAAACTATTGATGAGGAAGTTACTGATACAGAATGGTTTTTTCTTATTTCTATGACGCAGTCTTTTGTTAATGGACATGGGTTGCCAGGTCAAGCTATGTTTAGTAATCAGCCTGTTTGGATTACCGGGCGGGACAGATTGTCGGTGTCGCACTGCGAGCGTGCGAGACAAGGTCAGGTATTCGGAATACAGACTATTGTCTGTTTACCATCTAGTAATGGAGTTGTCGAACTAGGTTCGACGGAGTTAATCTTTCAAAGCTTAGATCTGATTAATCAGGTTAAGGTTTTGTTTAATTTTGGTAATAGTCCTCCAGATTTGGCGCCACTAGACGCGGATGATCAGGATCAGAATACTGATCCTTCGTCCATGTGGCTAAATGACCCACCTCCGGTGTCTTCTGGTACCACGGTTAATACCGTGGAAATGAAAGATACCGGAGATGTAAGGGCAGTTGTTCCTCCTAAGGAAACGAGTGTTGTCCCATTAAACAACTCGGTTCATACTGAAAATACTACTAAGTCTGTTGTTAATAATTCCAATCATCAAGGATTGTCTGGCAGCCGAGAATTGAATTTTTCTTCGGGGTTTGGGTGTAAGCCGGAATCTGTTGAGCTAGTGAATTTTGGTGAGAATAATAAGAAGAAGAAAGCCCCTGCTCTACGTGGTATTAATGAAGGGGGGATGATGTCATTTAGCTCTGGTATGGTTGTTCCTCCATCTGATACTGTTAAATCTGGGGGTGTAATGAGTGCAGCGGACTTTGACCAATCAGATATTGAAGCATCAATCGGAAGAGAGGTGGAGAGCCGATTAGTGGTGGAACCGGAGAAAAAGCCACGAAAACGAGGTAGGAAACCGGCAAATGGTCGAGAAGAGCCATTGAATCATGTGGAAGCCGAGAGGCAAAGAAGAGAAAAACTAAATCAGAAATTTTACGCTCTTCGTGCAGTTGTCCCAAATGTCTCGAAAATGGACAAAGCATCTCTTCTAGGAGATGCGATTTCTTACATCAACGACCTCAAATCAAAGCTTGACAGCACAACAACAGATAAAGAAGAACTGAAAGACCAACTCGACGCAATGAAGAAAGAGTTGTTATCAAAAGACTCTCATCAATCATCTTCCTCTACAGTCTCACCACCCGAAGATCTAATGACACCTAATCCAAGTAACCCGATATTAAATGATTTAGACATAGATGTCAAGATCATTGGGTGGGACGCCATGCTTAGGATCCAAAGTGGCAAGAAAAACCATCCTTCTGCACGCCTAATGGCGGCGTTAAAGGATCTTGATCTTGAAGTACACCATGCCAGTGTATCAGTCGTGAATGAATTGATGATGCAACAAGCTACAGTTAAAATGGGTAGTCGATTTTACACTGAAGATCAACTTCGAATAGCTTTGACAAACAGAATGTCAGATCCTAGGTAAGCTCGTCTTTCTCATGGGTAGTTTTCTTAAGCTACCCAAAAAGCAGGATTTGGGTGTAACATAGTATAGTTGTGCTTGTTGCTAGAAGTTCTGTTTTTGGTGTTTGACATTCATCTAAGTATTTGTTCTACTTCTATTATTAGTACTCTTTTCGATTCGGGCACATCTTTGAGTGCATGTATATAAGTTTAGGACTGATTTACCCTCTTTTTTGTATGCGAAAATGAGAGACCGTTACCACTAAGAATGAAAAGTAATCTGTCACATATCACATCAATTTTTCTTAAATCTTTCTCTGCATATAACATGATCTGTAGATCAAACAAATAATTTGTATTGTGTCGTGTGAAAATGTATAGCGAGTAAAACATACAACAATCGAAATTATTGCTTTGAACGGCAAAGACTTGGAGGCTATGAAGTATCGGTTTTATTTGGGATTATCTTAACTTTGGATTCAGTTAAGTATCTCTATATTCCAACAGAGACATGATTTAAATTTTAAGTGTTTAACTGTCTCCATTTGTGGATCATGGCTATACATGTTTATATTAATGGATAGACCCATAAAACACGATGCAAGTTGTAGCAACATGATATCGTGTTTTTGGTAAAGAAATGAATATGGTTTTAATTGGTGCTTGTTGCCATGAACTTCATGT

AaMyc-bHLH2

TATTTTGAAACATACGCGTTTATATATGTTTATATATAAAGTTTTAAAATAACCCAGAAAAAAAAATTTTGGTTTATGAAATATATAAGAGGAAATCATATAAGCTAATAAGCCCAAAAATAAGCTGAAAAAAAGCCTTATTTTCACACTCTTATATTGTTTTTTTATGTTTTTGTTTATGCAGGTGTTTGGAGATCTGAAATTAATCAAATTATTTGATTAGTTTTATTAAATTGGGATATATGGATTTTTAGTGTTTGTTTGGGGATATTATTTAAAATTGAAATCAGGGTTTGCATGAAATTTATAAAAGTGTGAGTGAATATGAGTATTGAAAGTTTTAATGATGAGGATAAAGCAATAGTTGCATCAGTGTTAGGGACTAAGGCTTATGATTATTTAATATCGAGTTCGGTTACTAATGAATCTTTGTTAACTTCATTAGCTAGTAATGATGATAATTTGCAAAATAAGCTATCAGATCTAGTGGAGAATGTTAGTTTAGGTAACTTTAGTTGGAATTACGCCATTTTTTGGCAGATTTCGCGGTCGAAAACAGGGGAGTTGGTTTTGGTTTGGGGGGATGGGTGTTGTAGGGAGCCTAGAGAAGGGGAGGAGTTTGATATTGCGCGGATATTGAGTATTCGTCTAGAAGATGAGAATCAACAGAGGGTGAAGAAACGGGTTTTGCAGAAATTGCATGTTTTGTTTGGGGGGTTGGATGAGGATAATTATGCTTTTGGATTGGATAGAGTTACTGATACTGAGATGTTCTTTTTGATATCTATGTATTTTTCCTTTCCGCAAGGGCAAGGTGGTCCCGGGAAATGTTTTTCGTCCGGTAAGCATTTTTGGTATTCTGATGCTTTGAAGTCGAGTTCTGATTATTGTTTCCGGTCGAATTTGGCCAAGTCTGCTGGTATTCAGACGGTTGTTTTGGTTCCTACTGATGGTGGGGTAGTTGAGGTTGGTTCCATTCGTTCTATTCCTGAAAATATGGACCTTTTACATTCCGTGAGATCATCCTTTTCGTTAAAGCCAAATAATGGTTTAGTGGCTGCGGCTCCATTGATGGGTAATGCACAATTATCGAGTGCGTTGATTGGGGAGAGAAAAAACGAAAATGGCCATGGCGGGCATTTTTTGGATTTAGGTCTTGTTGATCATCAACTTAAGGCGTCTAAGGTTGTTCGACAAGATATGGGCTTGAGTTTGCGGCAGCCTCAATTTAGGGAAAAACTTGCAGTTAGGAAAGCAGAGGAGCCACGGTCTCCTTGGGAAGGGTATCCTGTTACTAATTCCCGACTCCTGGCTTCAAATACTAGAAACAGAATAACTGGTTCGAATTGGGGACAGTTCACTAGTCCCCAAGAGGAGTTCCAACTTAACAGTTTTAGGCCTCAAAAGTCACCAACAGAAATGCAAATTGACTTCACGGGTGCTGTTTCTCGTCCTTCAGTAGTTTCTCGGCCAGTTAGTGGTGACTCTGAGGCATCTGATGTGGAAGCTTCAGGCAGGGATGAAAGAGCTGTCCTAACTGGTTTAACAGATGATAAACGGCCTCGTAAAAGGGGCCGAAAGCCTGCTAACGGAAGGGAAGAGCCGCTGAATCATGTTGAAGCAGAGAGACAAAGAAGAGAGAAGCTGAACCAGAGGTTTTATGCTTTACGAGCGGTTGTTCCCAACATCTCAAAGATGGACAAAGCTTCACTGTTGGGAGATGCAATCACTTACATAACCGACCTTCAGAAGAAGCTCAAGGAGATGGAATCCGAAAGAAGTGGTTCACACGGAAGCACTTCTATGGAAACACCCAACAACAGTAACAACGGGTCAAGTTTAGAGAAAATCGAAATTGAAGCAGATAAAGATCAAGTAACTGTTAGAGTAAGCTGTCCGGTAGATACACACCCTATATCTAAGGTTATCCAAGCATTCAAAGAAGCTCAGATAAGAGTCGTTGATTCAAAAATGGCTGCAGCAAACGACAAAGTGTTTCACATATTCGTCATCAAGTCTCAAGGACCGGAACAACTGACAAAAGAGAAGTTGATGGCTGTGTTTTCAAAGGAATCAAGCTCCTCTTTAAACTCATTACCATAAATAATATCAAGGTATAAATAGAAAGTTTTGTATTAGATTAGTTCGGGTTCTTTTATTTACTCCCTAAGCTTGGAAAACAATGTAGGCTTATTCCGTTGCTTATTTTTTGTTTTCTTTTGGTATACATATATGTGTAGAAAATGACTATTACTTAGAAACTAGAGCAGCGAAATTTTTTCTATGTTTATTCTATAGAACATTATTATGAATTTATTCAGTTGAAGTCCGCATACTATGAATCATTGTTTGTTTTTATTGTTTTCCATTATGGATTTCTTTGTGTTTTAGGCTTGTGTGAATCTGAAGTGTACACAGGTTATATTATGTCATAAGTCAGAACTTTGAACATAAGTTATTGTAATTCTTAAAAAACTGAAACATGCTTCCACAAGTTCCTGCTTTATGTGTTAAGGTTCAGTTCTTATCTGCTTTTGGTCCTCTTTCACTTTCATGGATTGAAAAATGGGAAACTCATTAGCCTTAAGTAGTTAGTGATGGAGTAAAAAAGACTTTATAAGCTAGCCTACTTAAAAGCTTAAGCCTTATAAACCTCGCGCAAACACAACCTATATAAATGGCAAGATGGATGACGGGTCAAAACAGGTTTTGTTGAAATGGTTCTTAGTAGACTGTACTGGGTTGGGTTAAAAATTACCCTTTTTTTTTTTTTTTTTTTTTTTTTAAAAAAAAACA

AaMyc-bHLH3

CTCACTAATGTCCTTAGAACATGGTTATGACATTTGGGCCGGTACAATATAGCCAAGCGATAGGACAAAAAAAATGCTTATAATGGTGCCAAAACACACACTGCACCACTTCCTATCCCACAAAATAATTGATTACTTTCATGAGATAAATTACCGTGTTTCGGAGATACCAATATAACACAATTATTTCTAGTCAGGTAAAGTAAAAACATCATGTGAGTAAAAAACAAAACGTGTTTAATATTACTGGGACCCATTTTTATCCTTCCATGAAACCACACACTCCATTCATCACACGCTTCACCCACTAAAAATACACACCCATCACACCCAAACCCTTCATATCCACTCCCACGTTTTTCACACACACTCAAAACACACACACATTGTTCACCGCAACTAACCCATCTCATGACGGAGTACCGCATGAATCACTGGACTCCCGACGAGAACGCGTCCATGCTGGACGCGTTCGACATGTCATCTATCTGGGCTAATAACAGTAACCAAACTCAAACTGTCCCTCCTACTTCATCATCAGCTTCCACGTCAACTGTTCACCAAGAGTTTAACCAGGATACTTTACAACAACGTCTCCAAGGGCTGATTGATACAGCCCGTGAGTCATGGACTTACGCTATTTTCTGGCAGTCATCTGTCCTCGAATACTCAGGTCCTCCCATACTAGGGTGGGGTGACGGGTACTACAAAGGCGAACCAAATAAACCGAAAACAGTCATGTCAGCTACTTCCTTAGCTGAGCAACAGTATCGTAAAAAAGTGCTCCGTGAGCTTAACTCCATGATATCTGGAACACAAGCGCCAGAAAACGATGCAGTGGATGAGGAAGTCACTGATACTGAGTGGTTCTTTCTGATTTCTATGACGCAGTCTTTTGTTAACGGAGTTGGACTACCTGGACAAGCCATGGTAACGAACCAGCCCGTTTGGGTCGCGGGGCGTGAGCGTTTGATGACGTCACACTGCGAAAGAGCACGTCAGGGACAAGGGTTCGGGTTACAGACTATTGTTTGTATTCCTTCAGCTGATGGTGTGATAGAGTTGGGTTCGACTGAGTTGATGTATCAAAGTTCGGATATTATGAAGAAAGTTAGGGGTTCGTTTAATTTCAATCAGGCGCGTGATCCTATGCAAGTTAATGATAATAATAATAATGATGATACTGATCCGTCATCCATCTGGCTGACGGATCCAGTAGCTACTTCTGTATCTACCGTGACAACTGATGTCACGGTAATCAAGGATACAGTTGGAGTTATTGGATCTGAGATGATGACAAGTGTCATTCCGTCTGTTAATAAGTCACATGTCCCGAAACAATTACCGTTTGAAAACCCTAACACTTTATCTCAAAACCCGAGATCTGGACATAATTTTGGAAGCCGAGAGCTGAATTTTTCGGAATTCAGATCGATGGAGGGCGGATCCGGTGGCAAAAATGCAAATTCTTCGTATACGAAGCCCGAACCAGGAAAATTACTGAATTTCGGGGAGAGTAAACGGAGTATTACAAACAACGGGGCATTGTTCGTTGGAACGGATAATAACAATAATAATATTAATAGTAATAAGAAGAAGAGGTCTCCTACTTCTTGTGGTAGCAATGAAGATGGGATGCTTTCTTTTGTGTCCGGTACCGCGAAATCTGGTGCTGGCCCGTTTACAGGAGCTGACTCGGACCACTCGGACCTTGATGCGTCAATGATTAAAGAAGTAGAAAGCAGTAGAGTGGTGGAACCCGAGAAAAAACCAAGAAAACGTGGCCGAAAGCCGGCTAATGGAAGAGAAGAGCCATTGAATCATGTTGAAGCAGAGAGACAAAGAAGAGAGAAGCTAAATCAAAGGTTTTACGCTTTACGTGCTGTAGTCCCAAATGTTTCGAAAATGGACAAGGCATCACTTCTTGGAGATGCTATTTTGTACATCAAGGAGCTCAAGTCAAAGGTCGACAACACCCAATGTGATAAAGAGGAATTAAGAAACCAACTCGAGGCATTGAAGAAAGAATTATTAAGCAAAGATTCGCGACAATCTTCTTCCTCTGCTATCTCATTGCCAGACGAAATGAAAATGTCGACCCATTCACTTATTGCGGATTTGGATGTTGATGTTAAGGTTATCGGTTGGGACGCAATGATTAGGATTCAATGTAACAAGAAGAACCACCCTGCAGCTAGGCTAATGGCGGTGTTTAAGGAACTTGACTTTGAAGTTAATCATGCAAGTGTCTCAATTGTCAATGATTTGATGATCCAACAAGCGACTGTCAAGATGGGTAGTCGATTGTACTCTCAAGATCAGCTCCGAGTAGCCTTAACAAACGGATTTTCAGATCCATTATAAAGTTAAGGCAATTAGCGAAGCTGTAGCTAGCTTAATTTTTCTTAAAGCTATGCTTGTTGTAATGTAGTAACATATAGTAGCATACTTATGGTTAATTATGTGTATGTTTAACGAGTGTAAGCTATATGTAACTGTATGAAATCTCGAGCTTTAGATGCTCTCGAGCCTTGTCTATGTAAAATGAAATAATTAGTTATGACTTATTATGAGTCGTTCGTGGTATCCATGTTTTTTGAGATAACGCAGTCGATTATAGATCAGAAAACAATGGTGGTACGAAAAAGGCATCCAATATAGGATTCGAAACATGAATTTAAATGGATGGCCATTATACGACCATGCAATGTTCTGTGGTAAAGGAAACACAGTGCTTAAAAATGTATGTTACCATGGATGCCGACTTGTCGACATCTTTTTTTGTCATTTAATGTCACTTTAAAACACCAAACATTATCATTATAACCAACACTTTCGGGTTTTCTTGAATTATAGGGTGAGTAGTAACATAGTAAACCAGATTTGTATGCACATATTATCTATGCAATAAGCAAAATATATGAAGTTCAATAAACAAAATATATAAAGTTAACAGTAATGATTTATTATGGAATAGTGTCTGTCCACTTCAAAATATAATAAAACAAGACACGATGAGTTGTTCAATGAATATCGAATGAATTTGCAACACACAAAATA

AaMyc-bHLH4

GTATATATGTTATAATATATATAGTAATTAATAAATTTAATTTAGTTTTTAGAGCGGAAGTAAGTAGAAAGAAAAAGAAAAGAAAAAAACCACGAGTATTACATTCCGTGAATAAAATAATGTCACTATCAAAAGATCAATAATAAAAATTAAAAAATATTATAACCCAACCATGGTTAGTTTCATGAGTACCCCTATAGAACAACGTGCCATAGTTTATACACACGCAAGTATAGATTAAAAGGACCAACACACAAACAAACCACTTCCCCCTCTGTCTCTACTCTCTCTAATAAAAAAACATCCCAGCCTTTGCAACCATTTCAACAAACAATTCTCAATCTTCATGTCCCAAAACAACAACCTTAACTTAACTTACCATATAATTTGCAAAATTTTTTTGATATGAAAAGCGAAGCTAGTATGGTTATTGGAGAAGAAAATTTATCAGAAGAAGATAAGTCCATTATTTCAAGTGTACTAGGATCAAAAGCATTTAATTATTTACTATCAGCATCTTTTGCTAATGAATGTTCATTTACTTCATTAGCTAATGATGAAAACTTGCAACATAAGTTATCACACCTAGTGGACAATCCAAACTCTGCTAGGTTTAGCTGGAACTATGCTATTTTCTGGCAAGTTTCTCGTTCGAAAAGTGGTGAGCTAGTGCTAGGATGGGGTGATGGGTCGTGTAGGGAGCCTAAAGAAGGTGAAGAATTCGATATAGGTCGAATTCTAAGGTTTAGGTTAGAAAATGATGAGAAGCAACAAATGATGAAAAAAAATGTTTTACAAAAAGTGCATGGGTTGTTCGGTGGTTTAGATGAAGATAATTATGCTTATGGGTTGGATAAAGTTACTGATACTGAAATGTTTTTTTTGTTGTCTATGTATTTTTTGTTTCGAAATGGTGAAGGTGGGCCCGGGAGATGTTTTGGGCAAGGGCGAGACGTGTGGATCTCGGATGCATTGAGTTCTGGTTCGGATTATTGTTTTAGGTCGAATCTTGCTAAAGCTGCTGGCATTCAGACAGTTGTTATGGTTCCTACTGACATTGGGGTTCTCGAGGTTGGATCAGTTAGATCCATACCTGAGAACCCCGGTGTTCTGCAGTCTATAAGAGCTTCTTTTTCGGGTAATTGTGTAGCCAATGGTTATGGGCATCCTGATCGTGTTATAAGTACGATCAAGATGTCCAAGATATTTGGTCAGGATTTTAGTTCGGGTATGAACCAACCACAGTTTCGCGAAAAAATCGCGGTTCGGAAACCGGAGGAGCCTGCAAGGGTGCCTTTTTCGAATTGGGCGAAAATACCAGGCCCACAGCTGCAAATAGATTTTACGGGGATCACCGAGTCGAAGGTTTCTGACGAGTGCTTGGTAAGAGATGATAGAGAAATGGTAACCACGATAATCGACGAAAAACGGCCTAGGAAACGTGGGCGTAAACCGGCTAATGGGCGTGAAGAGCCATTAAATCACGTCGAAGCCGAGAGACAAAGGCGCGAGAAGCTAAACCAACGATTCTACGCGTTACGAGCTGTAGTACCCAATATTTCGAAAATGGATAAAGCATCGTTGCTAGGTGATGCGATAACGTACATTACTGATCTTCAAAAAAAGCTTAAGGAAATGGAATCAGAAAGACAATCACCGAATTACAAAGTGTCTTTAGAGAAAATCGAAGTTCAAGCAACTGAGAACGAAGTTCTAGTACGCGTTACTAGTCCTTTAGAAGCACACCCTGTGTCGAAAGTAATTCAAACATTTGAAGAATCAAAGATAAAAGTAGTGGAATCGAAAATGAATGCGATAAATGATTTGGTGTTTCATACTTTTGTTGTCAAGTCTCAAGGACCCGAACAATTGACAAAAGAGTCGTTAATTTCTGCGTTTTCACGAGAAACCAGCTCGTCGTGACGACAATCGACATAAAATTCTAGGTAAAACGAGCTAGTTTTATATGTGATTAATCGTTCTTATATATATAGAGCAGATCAGAAAGTTTATTTCGTTCTTTTTAAGGTTATATATATAATTTTAACAACTAAAATGTATTAAAAATAAGATCTTAGATGTGTATGTTCGTTTCTTATGATATATGATTTTGTCAATTGCTGGTTTAACTTTTATATCGTTATAGTGATTATGGGTTCAAGTCATGTCTCACGTCTCTCTAGTAATGATGTTACATGATTCATTTTTTGATGTTGGTTGTGCGACCAGCTAGATGATCATTTTTTTTTTTTTTCATATATATCGTGTCGTGCGACACGTCTGGTATGATATCGTTACACACGAGTTTTGGTGTACCCANGTAAACACGATAATCGACGAAAAACGGCCAAGGAAACGTGGGCGTAAACCAGCTAATGGGCGTGAAGAGCCATTAAATCATGTCGAAGCCGAGAGACAAAGGCGCGAAAAGCTAAACCAACGATTCTACGCGTTACGAGC

AaMyc-bHLH5

CATTTAACATATTAATCTTATAATTATTTAGAAGCCGCCAAACATGTTAAACATCTGATTAACAATAATCTCATTGTGTTATGGTCCTTATGGATTGCATAGAAACCACTTATCCTGCTATCTATACTGTATGCTGGTACCAACCTGCTTATTGTTAAGTGCTTTTTTGCTCTGCAATAAGCAGGATAAGCAGTTTTACCCAAACACAAACTAATAAGCAACCCAAAACACCCTTTATAATGAAGTGCTAAAAACGCCCCCAAAAGACTATTGATTGTACATCTAATATCGACCTTAGAAAAATGCTCATTTGAACACCTGTTTATAATATATATTTTTTCCGTATCTGAATTCTATGGCAAATTCATTTTATTTGCAGATATGGGAGACAAATTTCGGTTAGAGGAAGAAGTTAAGGGTATGTTAGATAGTGTTTTGGGACGTGAAGCCCGCGAGTTCTTTGTTTGGTCAGCTTCAAATAAGGCTCCTGACGAGTTCAGATCAAAAACTAGTGATTTAGGTGTTCAAGAGGGTCTTCATAAGATCCTTGAAGGGTCAGATTGGAATTACGTCGTTTTTTGGCAAGTTTCAAATTCCAAATCAGGAAAATCCGCTTTGATTTGGGGTGATGGGCATCGTAAAGAATCAAAGGAACATGAAGAATATAATAATAATCGAGACGAAACTGTTAAAAGAATGAGGGTACTTCAGACGCTTCATTCGTGTTTTAACGGGTCCCACGAAGGAAATATTGCATCGAAAATGGATTCGGTTTCTGATTTGGATATGTTGTATTTAACATCTATGTACTATCTATTCCCGTTTGATAAACCTTCTAGCCCATCTCAGTCGTTCAATACAAGTCGGTCTGTCTGGGCTTCCGATGCCAAAAGTTGTGAAGAACATTACCACTCAAGATCGTTTTTAGCTAAGTTAGCTCGGGTTCAAACACTCGTTCTTGTACCTGTAAAACGAGGGGTCCTGGAGATCGGTTCTTTTAAGTCAATCCCAGAAGATCAAACCTTTGTTTCAACGGTCAAAACTTTATTTAACGGATACCATCCCCCGAAGGTATTACCGAAAATATTTGGTCAAGAACTTAGTCTAGGTGCAAAATCAGCTCCGAAATCAGGTCCAATAAGTATAAGTTTTTCCCCAAAAGTCGAAGACGATATGGAGTTTGGTAGCGAGTCCTATGAAATACAACCAAGTTTTGGTGGGAATTCTTCGAACGGTAATCAATCGATGGTATTGAATTCACAAGCGTTAGTATCTGGTCTCGATCAGTCGAATCAAGATTTATTAACAGATAGGAAACCGAGAAAACGGGGTCGAAAACCTGCAAACGGTCGCGAAGAACCGTTAAATCACGTGGAAGCCGAAAGACAAAGGCGTGAAAAGTTAAACCAAAGATTCTACGCGTTACGAGCAGTGGTCCCAAACATATCAAAAATGGATAAAGCATCACTTCTAGGTGACGCAATATCATACATAACCGATCTTCAATCAAAGATAAGAATATTAGAAGCTGAAAAAGAGGTAACGGGTGCACCCGAGGTTGACTTTATGGCTAGAAAAGATGATGCGGTTCTAAGAGTTAGTTGCCCGTTAGATGAGCATCCAGTTGCACGGGTTATAAAGACGTTTAGGGAACACCAGATCGTAACCCAGGACACAAACGTTACTGTTGAAGATGGGAAGGTGATCCATACGTTTTCGTTTCAGGGTCTTGCTGGCGGTGCGGCTGAAGAGCTCAAAGAGAAGCTGGATACAGTTTTATTGGATTGATGTCCAGGTTTCTTGTTGTATGTAAGATAAATTTTTTTTATCTTCAAATAAATTATATGTTTTAGTTTTGAGTAGTTACATAGATCAATGTGTTGGTTCTTATATATATTCATAGAAATTGTTGGATTTTAGATTATACTTTTGTAACTTTTATGCTAAGTTTAGAGAAGAATATGCATTTTATGTTTTTGGTAGATACCGAAACTATGTTGCATTCTGTATTGTTGCAGTTTCATTATCTCGTGTTTAGGTACTGTATACTCTCGTGTAGCGGGTGAATCCGGTTACAACTGGACTAAAAAGATTCAAACTGGAAATTAAAACTGGAAATTCAAACTAGGGGTGTCAGTAGGGTCGGGTTGACTCGAACCCGATAAGGCTTATCGGTCGGGTTAAATCAGGTTAAAAAATCTTGTCGGGTTGTTTTGACCTGAGCCCCGATAGTGTTATCAGGTCGGGTTAGGGTTAGATAAATAATGTTATCGGGTCAACCCGATAAGCCCCGAAAATCATTATTATTATTTTTTTAATTTTATTTTTTTAATAATATTTTTACAAGCCCAATAACAGTTAAACAGAGACTGCAAATTGCATGGGACGTTTGGTTTACCCATTCCATTACCATTTACAGAGGTTACAGACTTGCAGTCTTTTATCCATGGCTACTAGAATGTTATACTACTGGTGGTATTGCAATTTAATTTTTCATTAGTATGTTGTGTCAAGAATGTTTGTGTCAAGTGTGAATGGCTGCTGCTACTATTAGTCCTTTTTATTTTTCATGTTATAAGATCATGGAATATGCTTATTGTCACAAGGGGTGTTTGGTTCTTGACGTATGGCTGCAACAAAGGGGAGTTTGGTGCTGCAAGAGGGGCATTTGGTATTCAGTTTATTGCTGCATATTTCAGC

AaMyc-bHLH6

TGGAACTCCAATCTCAGAGCAAAGAACAACAACAAACCAAAGAACTATCGTGTACATACACTTAAATGCTCCATAGAAAGTAAACTTTATAATCATACATAGAGTCTCATTTTTGTCCTCTGGACAACTGATTCCATGTACTTAAAGCCATTGATGACATTGCCTTGGAATATGTGGATGACAGTGCAATCAAATAAAGTGTTTGAAGCTTTAAATTAAAACATATAAAATGCATGGTAAAGCCAAGTTTTTTAATATTGATACCATATATTTATGATCGTTTTCGTCATTACAAGATCATGATAGTAAGATTGGATCAAGATTTTATGAGTTTACTATCGTGTCATTTTTCTGATAGGCGTTGTAAAATTATGCAAATCAACATAATATTATATATCATATATCATAAAAATGTATATCAGAATTATAATTATGAAGCGTTTAATACAATTAGCCTCTTATTTTTCATATTCTACAAAACTTAATACATAAATTTGCTATTTTTTAGCTTGTGGTTCTAGAAAGTTAAGATCGGATTGATCGAGATCTTAAGATCCTATAGTAAAGATCGGAATTCTATTAAAATTCTATCTCGCAAACATCTTACAGATGATCGGGATCGTTTTTATTTATAGTATCGTAAAATCGTAATATCGTAAGAATATTACAACCATGAATAAACCTGCCCAAGACTTTGGGAACGACATGTGGTTGACTGTCACAATATGGTGTATATGTTAAGTTTTCAGATGATCATGACCCACGAAAAATGCAAAAATAAATTGTATTATATATATATGGTACGATATAAATATGAAATGTACTATATTTTTAACATCTCTATTTATTCTCCATCACTTATTGCTATATATCATCAGCATCAGATGTAACAAGATTCAAACCATTCGTTACGAGCTAATTGAAATGGATGATTTAATAGTCTCTTCATCTTCATCTTCTTCCATTGTTTCGATTCCTAGTACAACCATTAATCCAAATCAATGTGATACCCTCCAGCAAAAGCTCCAAACCCTACTACAAAACCAGCCCCAACCATGGGCTTACGCGATTTTTTGGCAAACCTTCAATGATGAATCAAATGGAAGCGTCTCCTTGTCGTGGGGAGATGGTCATTTTCTAAGCAGCAACGACATATTACCTGACAGTTTTCTCCCTGATTCTGACCTGGATTGTAGAAAGTCTGTTGTTAGAGAGATTAAGGCCCTTCTTGGACCTGATAACCGGGAAGATGTTGAATGGTTTTATGTGATCTCGTTAACTAGGTCTTTTATGCCTGGAGATGGTTCGGTTCCTGGTACCGCTTTGGGTTCGAATTCTATGATATGGTTAAGTGGGGTGGATCAACTTCAGTCTTTTAGCTGTGAGAGAGCAAAAGAGGCTCAGGTCCATGGATTGGAGACAATGGTTTGTATTCCAACTTGTAATGGTGTTGTGGAAATGGGTTCGTATCATTTCATCGAGGAGACATGGAATTTGGCGTATCAGGCTCAGTCATTGTTCGGCTTTGGTGGCGGTTCTACGAAGTTTAACGAGCTAAATGATGGCCATCATAATATTATTTCTTTTGCGGATATGGTACTTATGACTAGCGGATTGCATGACGAACAGGACGAGGGTATTAAAGTTTTGGGTTTTGAAGCAAACACGCCTGATGATGAGATGTCCAAGAATGGTGGAAAGTTACGCACGAACATGAACAATGTAGTCATTACCAATACCTACATAGAGACTGGGTCGGAACACTCAGATTCTGACTGCCAGTTGGTTCTTGCAACCTCAGAAAAGAGGTCGCAAAGAAAAAAAGGGAAAAACACTCGTGGACGGTGTCCACCTGTGAACCATGTGGAGGCAGAGCGACAGAGGCGTGAGAAGCTCAACCAACGCTTTTACGCCCTTCGCTCAGTCGTCCCAAATGTGTCTAGGATGGACAAAGCATCCCTCCTAGCTGACGCTGTCTGCTATATCAGTGAACTGAAACGAAAAGTTGAGTGTCTTGAATCTCAATTGCAGCATCGCAACAACCAAGGGAAAATCAAGAAAGTGAAGACAGAATTGCCGCACACCACGGACAACACTTGCAACTTGTATGTATCGACTAAACCAATACTCAAGAATAACAATAAGGCAAATTTAACCATGAACAAAATGACAAGCGGGTTCGGAGAAATAGAAGTGAAAATCGTTGGTGAGGACGCAATGATAAGGGTGCAATCAGGGAATGCTGACTTGCCCACGGCCAAATTAATGGATGCTCTTCGAGAAATGAAGGGACAAATCAAGCATGCAAGCATGTCGTGTGTGAACGACATAATGCTTCAAGATGTAGTGGTTCGAATCCCCGGTGCAACCGACTCGGATGAGCTAAAATCCGATCTCGTTAGGATTTTAGACCTCTAGTTATATATATTAGAAGCGTCTATTAGGATTTTTAGACCGCTAGTTATATTAGAAGTGTCTATTAGGTTTAATATGTATACATGGTATTAATTCATAATGGTAATGATAATGATGTTGTATTAATTATTAGTAGTATATATATAATGGTGTAACATTTGGTAGGTAATGTACGCAGATGGCTAGGTTTTAGAATGCAGTAAATACTATTGGAAAGCCAACTGTGTGTTGTATGTGAGGGAGCAGAAGTAGCGTCAATGAGTGTGCGTTTACTTCGGGTGTACTGCTGTACCCTTCTCCAAAGATACAGCGATTCTTTTGACTCAGACTCACGTGATGTCCGATTAGTTTAAAAAGTGGCTTAACTTGGGTTTATATTTATGTGTGATAATTAAATAGCACACATGCATTATTGGTTTGAACACACCACCTTTGTTCATACAACATGGACACATAGTTTATATCCCACGTCTCCTATCAAATCTGAATTCATAATCTTTGGTTAACGAGACAACCTACAATTTTTCGATCTTTTTGATATAGCACACACTTGTGGCTATTTGTAATAGTTACTGCTGAATTATC

AaMyc-bHLH7

ATGGACCTACAGTATTTTCTGGCAACTTTGTCCTCAACAAGGGTAAGTAAGGTATCATATCAATAATAAAGAGTTATAGAGTACGTGTTACTTGAATATGAATTGATTGCGATAACCTTTATATACTAACTTACTGATTAAGCCAACTCTTTTAGAAAGATAAAAATGCCTGCGTGGGTCGATTTTAGGTACTTCATGATCAATAAAATCATAAAGGTTATAATAGGCCGGCATACTTATTTTAAGAAAAATAAAAGTAAAACAGGAGCACTTTTGTCGTTCTACAATGATTCGGTAATACTTATTTTGGTTGAAATTTGCATAATCGAAAGTAAAACGATACTTGATGCCTGGAATTTTATATTGAAAATTTGGTGGTCTTGTGTAACATGCATTCACTCTAGCTACACTTTGGGTAGTTTTTCATTTGCCTTGATTAGATTTTTTTTTTTTTTTTTTTTTTTTTTCTTATTTAATCTATGGTTAGGGTTTTGGTATGGGGAGATGGATACTACAATGGATCTATAAAGACGAGGAAGACTGTTCAGTCGATTGAAGTTAGCACTGAGGAGGCTGCTTTAGGTAGAAGCGAGCAACTTAGAGAGCTCTATGATTCGTTGGTTGCTGGTGATCACCTGGTGACAGAGAACCCGCAAACAGCAACTATAAGACGACCATCAATGGCACTGTCACCAGAAGATCTTACTGAATCCGAGTGGTTTTACCTCATGTGTGTCTCTTTTTCTTTTCCTCTTGGTATCGGGTACGTTACATTTAACCTAAAACTCGTGTTGTTGGTTGCTGCTAAACGGTAATTAAATTCTGTTAAACTTTGGGACCAAAAATTTATTCTTAGGAAAAAAAAAAAAAAAGTCATTTGGTGCAATGTTATATACTTATATAAGACTATCAATAAAATTACTAATGATCAGAATTCTGCTAAACGTAATTAATACGTTATGTTTACGTGCATTTGCCTATATATGAGAATACGTTATGTGTATATCATTGCAGATTGGTTGGAGAGGCATACACGAAGCAGCAACATCTATGGCTCACAGGGGCAAACCAAGTCGATAGCAAAGTTTTTACAAGAGCTATTCTTGCTAAGGTATGTAATGTAGTTCTTGGCCGGAATATTGAAAAAGACAAACAAATTACTCCATTGATTTGAGAATTGATGGTATAAAATGGAATGAATACGGATCACATAACTTTTGGATGATATATGTATAAAGTAGGTCACCAAATTAATGAAAATTCGAAACGTAAAACATTTGATTTCTCTTTAGTGTTGTTTATATCAAAATCAAGAAAATGACAACATCAAATAAAAAACAAAGTGTTTGACGTTCGCAAACATTGGTGGCCTTTGCATGTAGGATGCCCACATTTCATTTTCTAATTTAATAGACTAGTACCTCTCGAATAGGTCATTGGAACATATGTGGCATGAATATATACTATACACTCATGCACTGTGTTCTAATTTAATCAAAGTGTCAATTACTTTTAGTAAATCACTGTTACAAGAGGGCTTTTAGTCGACATGTATGTGAGTGACCCATCAACTAAGAGATTATTTATTCGAGTTTCGTTTGCAATACGTGTGAAATATTATGTGATATATGAGTGTGTGAACAATTTGCCTCTATAAAAAAAATTAAGAGTTATGAATTGGATAATCTAATTACATTCTAATTTTCATGTCCCTTGCCAACAAAGTAACAGGAATTAGTTCATCACTTGATGAAAACTACATTTGCTGCACTTGATTAAATGAACAATCTTCCCAATAAATTCTGATAGCATTCATTTCATTTCGACCTGTTGGTTGCATAATCGACAATAACATTCATAGTTGTGGTCCATAACAATTCCATTTCTTATTATTTACATGTTTCGTATGTTCCAGTGATGTTACACTATTTAGAAAGATCGAAATTGGAAAAATAATAAATGATTTAAACAAATCGATATATTTGTATTATTTACAAAAATAATTTCTAACGTTTTGATTATGTTAATGAATGTTGCTTTGCATCCTTGCTGGGGGTTGGTTGTTGCACCAGAGTGCTAATATACAGGCGAGTCGATTTTTACTCCCAAACTTTTATACCTATTATATTAGATATATATGTATATTCTGTCGTTGCATTCTATGCTTAACGTCAAGCTTCGATTATGCAGACAGTGTTGTGCATCCCTCTACTAAATGGAGTCATAGAACTTGGAACAACGGACAAGGTTAGTCCCATATACCTTACTTTATATTTATATGTTATTTGTCACATGGGATCAAGAAACATAGATACAATTTTCCGCATGGCTGGCTTGTCAATTTGCACAACCATTTTAGTTCAACACCATGGAATTTATATTAGACGTGACATGTGGGTGGGTCGTTAGTTTGCTTCAGAACGAATAATTTCTAGTTTTGGTTGGAACTCACCCGTCAACATATATGATTTCTCCTTCTTTCTTTGAAACTTATGAATGTTTAAATCTTGTTTAGCTATGAGCCTGTGATTACACATATTTTATTGGTTAAAATCACTCTCTTTAACTAAATGATTTAGGGTATTGCAAGCATTACACTTTAGTCGACTTTGAACAATACTATCTTAATTTGTAGGTGGAAGAGGCTATTGAATTAGTTCAACATGTAAAACTGTTCTTCATGGCCGGCAATGACAACCCAATTCTCATACCTCCAAAGCCCGCCCTTTCGGCCCATTCTTCAAACACCAAGCTTTCCTCAAATCAGAAGACAACCACAATCAAACCTCTCGACAACACATATTCAATGGATGAAGATGATGAAAGTGAAGAAGATGAGGAGGATGAGGAAGAATATGAAGAAGCAGAAGAAGAAAATGTGTCGGGCATTGTGGCAGATACATGTCACTATTCCGATTTTCAAAAACCTAGTCAGGATAATACTGGAATGGATGCAGTTATGGAGGTGAACGAATTATTGCCACTTGATATGTCAGAAGATATTAGATTTGGATCACTGAATGATGATCCCAATCACTTGGACTCTCATTGTAACTTGTTAGCAACAATCCACGACGATTCTTATAGAGCCGAGTCAATCCCCAAATGGTCAGATAATTTGGAGTTCAACGAAAGCATTCAACTACAAGTATCAGGTATTGGGTTGTTCTTGTCTAACTCCATCTAGTACTCTTTTTTATAACATCTATCTCGGAAGGTGAAAAAAAAAAAAAAATCATTTAATTTAACGTTCTCGAATGAACCGGACTCATGCTATACAAATCTAAAAATATGCATAATAATACTTGAACCATTTGACCCGATGAATTGCTGATGTAGGATATCTTTTACTCCTGGTAAATTTTGTTAAATCTGCCATATGTCTTGCGTCCTATTGCTCAGCTTATTATCTAACTTTTATAAGCGAAACGAAATGTTATTGTCGTGCTTGTATCTTTAACTTTAAGTCTACAAATTATTTTTATATAAACAGGTGAATTATCACAAGGGGAGGACACACATTATTCTCACACAGTTTTAACCCTTCTCAACAACCAACAACTAACTCAACGGTCCAATTTTAGCACACCTTCCCGCCAAAACTCCATCCAATCAGTCTTCGCCACGTGGACACCAAATCACCTCTTTCCCGCCAAAACCAAAAAAACATCCCAACGCATCCTAAAATACATGCTATGCACCGTACCTTATCTCCACTCCACAGCCAGCCCCGGAGACACCTCCACAGTCGTAGGAGCAGCCTCTCGCCATGACGAGGTCAGCGCAAACCATGTCATGGCGGAACGGCGTCGTAGAGAGAAGCTAAACGAGCGTTTCGTGACACTCCGGTCACTAGTCCCACTAGTGACCAAAATGGACAAAGCGTCAATACTAGGTGACACGATTGAGTATTTGAAACACTTGCGTAAGAAGGTTGCTGAGCTTGAGGCGCGTGGATGTAACGCGCCTCAGGGGAGGTTGCCAGAGAAGAGGAAGATTAGGGTTGTTGAAAGTGGAGTTACGGCGGTGGAGGTTTCGATAATCGAGAGTGACGCGTTGGTGGAGATTGAGTGTGTGCATAGAGATGGGTTGTTGCTTGACGTGATGAAGAAGTTAAGGGAATTTGGTGTCGAGATCGTGACTGTTCAATCCTGTGTCGATGGTGGGATTTGTACGGCTGAGATGAGAGCTAAGGTAAATTACTTGATCTTTTTTTCAGTTTCTAAGTTGATTGTACATTGGTTGCTCGAGCTTTAGCTTGAACGGCGAGCTCGAACTTGAATTATTTAGGCTAACTCGGAGATTGCAAGCCTCGACTTAAGAAAAACAAGCATGAATATCTTTTAAGAAAATCTCTTTCTGTAATCAAATTTATTTCAAACATAAGCAAAAGCTATACCATTCGATAACAAAACTTATATCAAATATATATGTAAATCACACCAAAATTTGACCACTAGCTTAGTCAGAAGCTTTTCAAAACAAACAACTCCAAAACTAATAAAAATCCGCAGACACTGAATTTTTATAAAAATAATTGCATATAAAAAGTATATGGGCTTGTGTTGAGCACTAAGCTCGAACTTGACTCATTATACTATTTTAAACAAAGAGCTTATAAAAAGAACAAATTTGTGTGTGAATCAGCTATTCTATTTTGTATACTTAGGTCTCCTCTTTATTGTCCTCATACTTTTTGCACTAACAAGTTTTTATTTGAAATAAATTTTCAGGTGAAGGTAAAGGGTATCAGGGGGAAGAAAATTAGCATAATGCAAGTAAAGAAAGCCATCAACCAAATAATATCTCCTTAGAAAAAACAGAGTAGAGGGCACATAAGTGCAATTTCTTTATTTAGTATCAAATGAAAGGGTGATGCTGATTACTGAATTAGATATATGGGATTTTTTTTTTTTTTTTTTTTTTTTTTTTTTTTTAATTAATTAATATCGGAATTATTTTTTCTCTTTGAAATAAGATCATGGTATGTGTAGGACATAGAGGACCCAACCACGCG

AaMyc-bHLH8

CTCATAAACTCATGTAGAGTGTTTGGATTAGCTTATGGTAAATGGGTAAAAAGAAAGTAGCTTATAAACCGGAATAAATAAGCCCACCAAATAACTGCTTATATTTTTGTGGATGCAGGTTTGCTGTTCATTTTTAATCCATAAAATTCGTCCCAAAGTGTGCTTTAATTTTTTCCATGGTTTTTAAGGTTAGAGGAATAGTGTTATAGGCTTCTGATAACGAGCTTTTTTCTGTGAATTCTTTTGGATGCACTTCTTTAGTTAATGGATGGGACCGTAACTAAGCTTCAAATAGAATTGGCTGCTTCTTTCTTTGACATCGTATCATCTTGAAACAGAGAATTTGGTACTGGTTATTGGTATTAGTCTCAAGTAGAGCAAAGTGCGTTTATTAGATATGCGTTGTGTCTTAATGGTTTCATCAGGACCTCCAATAAAGAAACGTTCAGGTTTGCGGATTAAACAAGCAGGACGTGGCTCATACAGGGGTAGTTAGCTGTACTTGTTGTTTTTGGGTAATTTGATCCAAGTTAATCTTTGCTGTACGTACTGGTTGAGTTTTTATATTGAAAAAGTAAGATGGGAACTAGAAGTCCGTTAAGGAGTTTCCTACAGAGTCTGTGCGAGAATTCTTGCTGGAATTATGCTGTTTTTTGGAAGCTTCAGCAACAAAACCAAATGTATGTATTGCTTTTCGTCTTTCATAGCCAACGATTTATATACATCTATAATTTTGCCTTCTAGAAAACAGGCAATTTCTTTATGTGCAAGTGTTATTTGTGTTGACAAATTAGTTATTAACATCCATCTCAGTCAATATAAGGTTAATCAATGGGTTCTTTGTGCCTTGGATGCCAAGTCAATTGAGCATTCACTCCATCCAGTAGAGTTGTGTATGGAATCATATGTTTTTTTAGCTGTAGCTATCATGATTCATGAACCCTTCTTTGTTTATGATAGGATATATGTGTCAAGTTCCGTGTTCATTTGTAGGTTGTTTCTTATGTAGTATTCTTCTTTTTTTATTATTATCATAAGACAACTACCCATGAACCTGCCCAAGAAGCGAAATCACGTTGACTTTTTAGATTCTATAAATTATGTGATTTGTCGGGCGGTGGAGATGTGATTATATTATCAAGTGAATTACACAAGTGGTCCCGTGAGTAGTTGATGACCTGCATTTTGGTGTCCGTTTAAGCTTCCATCACTTGATAGTTGGAAAAGGGTATAATAGTAAATTATAAATAATAGAAGTCAACTCTGTCATCCCTTCCTCCCTTCTCTAAAACAATTTATCTTGCCATCTTCTTTCACCCGGATCACATAACACATATAAATACATAACCAGCCTCCAACATACACACACATGCTAGCTAGAAGAGGGAGTTCTGAGAGAGAAAGGAAGAGTGGGATATAGAGACATAGGCTTTTTGTTTTTATGTTTTTAGTTTTTTTATGTGGATAATAAAAAATTTTGACCTTCAATAAGACATTTATATCTTCATTTATTGGCATGTGAGGTTTGTATGACACAATAAAAAAACCTCCTCAACTGTCTTTAAGTTTCTTGATACGCGAGGATAATAATCTACTGTTCCATAGATGATTTCTTCAGTTTCCTAAAAAGGAAACGGGTCAAATGGTTTAAAAGTACCCACACGACACTCATTGTTAACAACTTGTGCTTTATTCTGAAAGTTAGATCACTATCATAATGATATTTAGGTAAAAATATAAAAATATTAATTATTTATAAAACTGTGCCAGAAAAAAGAAGAGACTTGACGAGTGAACCTAAACTGACCTGACCTGTTTTTACCTTTCTTAAGATGTTTAGCTGCTTTCCGATCAAACCCTGGATCTCTTATCCAACTAGTCTGACCTGCCCTATTGCCATATGCCTAGTAAGGTATCAGTTTTTCTACTTTACTTGGTCTATCCAGTAAGGTACTTCTATCATCTGATATTAATAATAAGGTAGCATTTGCTGTGGCATAACTGCATTTAATCATTTATATGATCCCAGTGAGCTAGACTTTCTTTCTTAGGATTGGTAAGTGAGAGAAAGTCTATAATCATCAAAAATTATTTTCTAATGATGTTTTGCTATATCTGATTGTCTGCCAAAGTTATTGGGGGCACTTCGACTTGAATTTCAGTGTACCGGGCCACAAATGTATGAAAAATCTGCATTCTTCAATATGCCTGATAAAGTTCTAACTTATCAATCTACTTGTTAATCATACTTCTCATTTAGAGTTCTTTTTATGGGATGTGATCATTGATAGATTTTATGCTTTGTCTAGACTCGTGCTTTGGTTTGTTTCTTAATTAAAAAATTATCTATACATAATATGAATAATAATGTTCTATGTCTACCATTATGTTTTTCAGATTATGTGACTGCATACTCTTTTCTTACATTAATATACATGTTCATCTGGTATTTAGGATTTTGACATGGGAAGATGGATGCTTTGGCAACATGGAAGCTCAAGATTCCATTGAAAACATGTTTGCTGAGACTAGCTTCCGAGGCTTAGAAGAAACATCCTCATGTAACACATATCGTGGAACTTCGGGAGGAGAGGCGGTTGAACTAGCTGTGGCTTATATGTCCAACTTTCAGTATGCATTGGGTGATGGGTAAAGCCTTTCTTCTGCCCTTCTTTATGTTGTTTCCTGGTGATACTTTACTCTTTTCGTTACCTTTTATGTGTATAAAATTAATGAAAACAGTATGTTCATAGAGATAACGATACCGTTGGTTGTCAACATGATCTGCTTTGCAATGTAAATTTGTATGAAATTCATTAGGAAATCAACTTAATTTCCACATCGAAAAGAAAATAATGTCTAAAGTAACATCACATCGTGAGTCACCGTCATATAACCAAATCGTGTAATAACCCAGATTTTTTTGATCTAGAGACTTATTTTCTAGCTACATGAATATGCTCACTGTGTTCGTATTGTTATGAACAATGAAAGAGACACGACAAGACTTATTTTATGTATGCTTCTGTTACGCGGTTAGACAGTAAATGGGCATAGAAAAACTAAGTGAATGTTCCATTCACGAGTCATGAGATCTGTCTACTGTTTCGTGATCCATGTCATAAATTAACAATTAGTCCAGACCTGATTATAAATTGGTCATAACATTTTTAAATCATGTAACTGGTATTAAGGAATTGTGAATTTTACTTTGTAGGTTTCTCTAAAACCTCACAAAAGATGGAAGGTTATGGTAGTAACCCAAATATCAAATAGTTTAAATGATGAAAACTAATTTGTTTGGTCAATCACTTTAGGGCTGTCGGTGATGTGGCATACACAGGGAACTCTCAGTGGGTTTCTGCAGGTTCAAAAACATCAGGGGAAATAAACACTACACCAATTTCTGAGGTTAGTTTGGAATATCATCAAGTGATAATTCCTAATAGAAAACCTGCTTAAGTTATCTAGTTGTCTGAGTTAACAAAAAAGTTACCATCATATTTTTCCATTGGCAGCATGCTGATGAATGGGTATTTCAGTTCGCCGCAGGTGTGAAGGTATTGATTCGTGACTAGATTATCTTATTGGTTTCATTCTAAAGCTTGTGTGTATGTTTGTATGGGTTCTACGGTTATAAATGGGTTGATTTGGGTTGTGTTTATCTCATATGGCATAAATAATTAAAGCAGCTAAAAGGAGAACGTGTTGAAAGCCGGCCACTGTGTAATTGTAAGGTGTAGAACTTTCTAAATTATTTTCATCAAAAATTTGGATTATTACTTTATACTTATTGTTTTTCTTATCAAAGTTAACATACTACTTGATTAGTAAAAGGACAAAAATAGTGTTTGCATCAACCCAGCTTGTCACGGCCTGTACAGAAAATGACCTATTTCACCCGAAACCATTTTGATAATTTCTTGATCACTCCCATGCAGACTATTCTGCTGGTGCCTGTAATTCCACATGGAGTTCTGCAACTCGGTTCGTTGGACGTCGTATGTATCCCTTAATGTATATTGGAAGGGAAACTTCACTTTATTGTGAATGTTATTTTGTGTATTGATTTATTTTACCCTTAATGTTCCAGGTTCCTGAAGATGCAAAAATGGCCAACTATATTAAAAGTGAATTTGTTGCGCATCAAGATCTCATTGCTTATTCTGATGCTTTTGCTACAAACCAACAATTTTCATCTCAATCACCATCATCACTCATGCCTACAATGATGAAAAGTTTGGATGAGCTCCTATATGATGATGTAGCAACCATTGATGATGTTAACTTGTCAAACCCCAAACATTTGACCAATGGTATTGTAATACCAACTTGTGATAACTGGTATGCATCTCATTTAGCAGGAATAGATATGCATAACCCTTCTGCAGGCGGCCAGATGGGTGCTATTGAATTGACAGAGCCACTACATCAATTAACAACTTTAATCAATCCAGATGTCATAGAAAGCAGCAATTCTGGATGTCTAGAACCATTACTTTGTGATGTGGGCGCGTTGTTAAGCTTCCCTAAAGAGTGTGAGCTGCACAAAGCTCTTGTCCCTCCTTTCATGGGAAAAACAGATGATTATTCCCAGCATCTTTCCATCGGGGACGATATATACACCTCAAGCCATATTTTTAGTGAAGATCCTGTTGACTCATTATCTAAGAAAACAAATGGATGCCTTTCGTGCAAGGAGAATGTGCGAAATCCCTTAGAAAATGTAGTCACAAGTCTACATTTGGGTGAGAATTCTTCTATCCAAACCAGTGGCCAGAGCTCAATTACTAATTCGCTTGGCCAATATTCCTCTGTGGCAAAAAGAAAAAATATACATGACAAAAGCGCATTTGAGGGAGAGAGTTCACTAATCAATAACCATGTAGCACCTGCGTTATATAATAATACTGCCAAAAATTATAATGATAGTTCTTCACCTTCAGCTATTTCGTACGAGGATGTAGTTGATGAATTGGCCGTGGAAGAAGAGCGAAAAAAAAGGTATGATGGTATGCATCATAATGAGGGTTCAAAACTATCCATTGCCAGCAAAAGAAGGGGCAAACCTGGTGCTAAACAGAAGGCCAGACCTCGAGACAGGCAATTAATCCAGGACAGACTCCAGGACTTGCGCGAACTTGTTCCAGACGGAGCAAAAGTTAGTGCCTCCTGAACACCACTTTTTGCTTTTAATTTTTATTCTCGTATAATTGTTATATGAGTATATGACACCTTTTACCAGTGGCATACTTTTGTATCAACCCAGGGATGTACCCACCAAGTTTTCTTCAATAACTTTTGTTTTTTTCAGTGATTGACCTGAAACACATTTTTTTATATGTATTTTATGTTAAACTTCGCAAAAGGGTATCGAACTACGTAGTATAGAACGATTCATTGAACGTATCATACAACGAAAATGTTCAATAATGGTGTCATACTTTTGTTTTTATAAACAAAAGGTATCCAACTTTCTTGCACAAAAATCGAAGTACGATACCATTATTAAACATTTTAGTTGTATGATACCTTAAATAGGAATTGGCCTGTAGTTCGATACCCTCTTGTGCAGAAAACCTATATTTTTGGTAAACATTTATTCTGTCAAATAGGATTACAACAATGATCTTCTTCTTCCCCCCCCCCCCCCCCCCCCCCCTTTAGTTTAGTTTCCGAAAAAAGCATAAAGCCCGGAGGCGGGTATAGTCGAACTGGTTGATTCTAAGGTGCATTTTATTTCTTCCTCATAGATTTGGTGTCTAAATTTAATCCTAAAGGAGGCTTTATGCACTAAATATACATTGACTAATACGTAGCCCCTTTCGTTTTACGCTAATTTTAGTTGGTTTCGCCCACTAAAGATAGTTGGAATCTATCCATTTGAAAGTAAAATGCGTCTATATTTATTTTCTATATTTTTTTAGATTTATTATACATGCTGCTTTTCAAAATTTGTTATGGTTGTGGTAGTTCATCATCTGATGGAAATCCTGATCCGCCATTGCCTTTGGCCCAACTAGTTTACATGGACCATGCAAAATGTTGTTTCAAATGATTGGCGTATGCTCTGCCATTTGGCTAGTTAACGCGATTTTTATTGAGTTGTATCGTCTAGTGGGATTCATTAGGTATAACATTCTAGTAAATTAGCCCTTGGGTTCTTCAACGTCAAAATTTTATGTTGAGTTAAAAATTTAATTTCTCATGTGTATCATCCTAATTTGAAATGCAGTGTAGCATTGATGGTCTGCTGGATAGAACTGTGAAACACATGCTGTTTTTTGAAAGTGTCGGTGATCGTGCTATCAAATTGAGGCAGAGTTTGCAATCAGAGGCACGTGATTTCCGGATATTTTTATCTATATTTTCTTGCCTTTATATAACATCTTATGCCGGTGGACAAAATCGTATGCATGATTTTCTTATTAAAATATCTATTGGGAGAATTGTGCATTTCGAGTTCATATCTTATTCTAACAAGTTTCGTAGTGTCTCGATAGAGGTGGTTGTTGCGACCCATTTACTCATGAATGGGTTGATTTGGGTATCTTTAATCTCTAATGAGTCAAATATAATATCTTAAAAGGAACCGGTTGTGAGGAGCCAAAAATGTAGTTTTCGTCTAAAACCCCCTGCAATCGTTTTATTCTAAAATTTAGATATTTAAAAAATATATATATATATATATATATATATATATATATATATATATATATATATATATATATATATATATATATATATATATCTGCAATTATTTCATAAAAATGGTTATTTGACCCATAATCTACTGTCTGGTGTCTGAATCATCTGCAGGGAATGGGAATGGGTCCAAAGAACAATAGAACACCTGAAGTCAAAGGTATTCAGAATGGAGCAAGTTGGGCATACGAATTGGGAGGTGATATAGAAGTATGCCCCATACTTGTTGAAGATCTTCAATACCCTGGCCACATGATAATTGAGGTCTGTGTGTTCAAATTTGCCTACTTACGAATGTACATATATCTTGTGCGTAGATTGTGGATGTCACGGTACTCCCATTCATTATAGTTGCATATTTTGTACTTTCGTAATTCCCATTAAATAACATATGGATGCCAATGGTCAAATTGGAACAAAGAGGACGTCAGAGTTGCATTATTTATTCTGTTAGTAAAATGAGGAATGATGGTATGTTATTAAAGAAAAGTCGATGAATTTGGCAAATGAATTTATTTTTATGATTATTGTAAGCAACCAGATTACATCTATTATTAGAGACTCTGTAATAACAACTTATATACTTTTGTATGTGTATTCTGCATCATTGAGAAAACCTGCCTCCCCCTAGTAGGTCGAAGATGTGGGTGCCTAATAGATCTTGTATTATGTCAATTGTCCTGTTAGAGGAGGAGAGTACAATTTGGAGAAAATATTATCTGTCTCGATCATTGTTGATTATGGATATTAATAATTTGACTAACAGTTGACTAATAAGGATAATTTAATCATGATGGTAGTTAATCTTTTTGTCAAGACTTGAAGTTTGAACACATTCCGTTAACAATCGAAAGGGCAAACATGAATCTTATGTAACAAAGTTTATATACCACAAATAGCCTTACCGATTGTGGTGGCTTTCATAACAGATGCTATGCAATGAGAGCATTCGTTTCCTTGAGATTGCGGAGGTGATACATGGCCTGGACTTGACAATTTTAAACGGTGCGATGGAGATGCGTTCCGGTGATACATGGGCTCGCTACATTGTGGAGGTATGAACTTTTTTTATCTTTAATATCTATGGTTTATTCGTTTTCTCATTTGTTGGTATAAGACACATTTGATTGAATTAGCCTACGAGGTTTGTGGAAACCTTTATGATACATCGGATCACTAACAAATTAGAGACTAACTAACGTAATTTGACTAATTAGCTTTTATTGGCCAATGCATGACACTTTGGGTGAATTTAAACCCATACATTCATTTTACCTATTTTAGAATTTAATCCATTGGCTTCTTAGAGATAAATCATAACCCGAATCGACTCCTCTATAAGTAAACGGGTCAAACTGTCCACCTCTTTTAAGAAAAATGACAGTTTTTTTTTTTTTTTTTTTTTTTTTTTTTTTTTTTTTTTTTTTTTTTTTTCTCTCTCTCTCTCTCTCTCTCTCTTAATTACTAAGTGAATGTGTACTTTTGTATGCAGGCTCCAAGGGGATTTCACAGATTGGATATATTTTGGCCATTAATGAAGCTTCTTCAGCAGCCCAAACAACATAGTCCAATATCCAGCTGTTGGGCTAATGTCTGACCGAGCTAATGGACAGATAAACCGAACCAAGCATGTGGGATGAGTTACAAGAATAGTCTAATTAGGATTCCTTATTTTTAGCTTTAAGATAAGGATTATCTTATTTAATTAGAATTGTATTAGGATTAGGAGAGCTCATTCTTAAGTCCTATATAAACAACCCAACGAGCTTGTGTTGGGTGTACCATTCATTATCAGTTCTTGTAAGCAAATACTTTTCTGATTAATTAGAAAGCTTGTTCTAGTTATTCTCGTGGTGTTTTTGTTCCAGTTCTGTCGAGTTTTGTGTTGGGTCGAATCAGATTCAGGTCTTGAATTGGTATCAAGAGCAAGGTTCAAGCAGTCGATCAACAGGATAAACCAAGAATCAAGTCGATCTCAGAAGGGTTCCAAGCATAAGTCGACTAAAGGGATTCCAAGTCAGTCGATTCATAAGGGTTTACAAGGGTTTCTAAACCAAGGGTCGACTAAAGGAATTGTCGAACACAAGGGTTTCTAAACCAAGCCTAAACTCTAAAACAAGGGTTTCACCAAGAAAGTCGATAAAATTTCAAGCTTTATTTGATTTACTTAAGCAAGAAGTAAATCAAGCGTGATTAAGGGCCGGAAGTCGAACCAACTACACCGGTAACCTACAAAAGACGTTCAAGAAAAATGGTAGGCGAGACGAGTCAACCACATCCTATTGAACATTCCAAAACGGCTCTTCAATGTCCAATGTTAACAAGCACGAAT

AaMyc-bHLH9

GGTCCACCGATAAAAAAGCGAGCAGGACTTCGGAGGAAACAGGCCGGCAGAGGCTCATACAGGGGTAGTTAGACTGTTAGACTTTTGCAGTAAGGGTAAAAAGTCATTTTAGTTTGTGTTTTTGGGGGTGGGGTGTTCATAGTTTATTAACATAGTTTGGAAGAAGATGATGGGTGGTGGTGCTCAATTACGCCAACTACTCAAGCATCTTTGTGTTAACACTGACTGGAAGTATGCTGTATTTTGGAAACTCGAGCATCAAGATCGAATGTAAGATCATTTTCAACAACTTTAGTGTGTTACAGTTTGTTATATATCAACTCGTACTGCATACAATATTTGCCTAGATGTAGATTCATGAGGAGTATAATGATTGTTTTGCAAGAGCATGAGCAAATATGCCATTACAGCTTGTAAACTTACCAAAATAAGAAACGTTGCAGGATGTTGACTTGCGAAGATTCTTACTGTGTTAATAATGAGAAAAACAATCGTTTAGAGAGCAACTGGTTTGATAACATGGGTGACAACTTGAAGGATGGGTTTTACGCAAAAGATCTTATTGGATTAGCAATTGCAAAGATGTCTTATCGTGTTTATTCTATTGGGGAAGGGTATGTTGCTAAACTTTTACTAACGGTTGGATAATATATATTTAATCTTGCTCATAGTGCGTATGTTTATGAAATTTTTTATTTAGGATTGTTGGACAGGTTGCAGATACCGGTAAACATCTGTGGATAAACGGGCATCAACTTGTAAATAGGGTGTGTTCTTTAGAAGAGGTGAGAAATAGCTCTGATTTGAGAAATATTATACAATATTGTCTTTTAAGTTCTGTCATGCAGATTTATGTGGTCATTGGTTAATTTTCAGCCTCTTGATGGATGGAAAACACAGTTTACAGCCGGGATCAGGGTACGTCAAGTCACCTCTAGTCCTCTTCCCCAAAGATTAAGTTCTAGTTGATGAATAATAATACTTTGCTGTCTATTGTGATATGCAGACTGTTGCTGTTGTTGACGTTGTTCCTTATGGGGTTATACAACTTGGCTCTTTAAAAACTGTAAGATAACTTAAAGTTTTACTTCTAGCTATTGTACATGTAAATAGAAAAGCTGTAACTCTGATCTATAGACTAACCCTTGCCATTGATACGAAACATGCAGGTCACTGAGGATTTGAAGCTGGTGAACAATATCAGAGAGATCTTTTTCGAGCATCAAAATTCATTGATGTCATCTACGGACTGTTCTCCTTGTGTGGTATCTTTCTCTCCCACCACATCGCATTTAAGATAAGACTTGCATAAGATCTTACCAGTAATCCTTTTTTCCAGACAGATGTGTCAACAAGGTCGGATACGCGTACGCCAGTTGAAATGATCAACTATCATCAAGAAAAAGAAAACCCAATATTAGATTTCATCCAATCTACACTCTGCAATGAGCAAAATCGCGACTTGTTCATGCCGGGAACTTCAAATGATCAAATTAACTCCAACTACCATCATGATGTAGGAACTGAAACTAACGAGTCTTTCGTGTTTCCATCTGGGTGCGAGTTGTATGAAGCGTTAGGAACGGCTTTCTGTAAGCAACCATACAATTTTGATTGGGAGACAGCAACAACCGAGACCCTAAAAGTTGACAGGATCCCGGAAGAAACAAGTAGCAGTGTCTTGACACAAGTATCCGGCTCTGAGAATCTCCTGGAAGCAGTTGTAGCCAGGGTTTGTTGCAGCGATAGTGATGATAAAAGGCCAATGTCATTTGGTCAACCAGTGACTTGTTACTCAAATGAGCGATCTTTACAAGGCTTCTCATCAGCAGGCGTTAGTAAATGTAGTGAGCAGTTAGATAGGTCCCGAGAACCACCTAAGGTTGGAAAAAAACGAGCTAGACCTGGTGAAAGTCGTAAGCCTCGGCCAAGAGATAGACAACTGATCCAAGATCGTCTTAAGGAACTTAGGCAGCTTGTTCCTAATGGATCAAAGGTAATTCTAGTTTTAAGAGTACTATTGTTTATGGTTCTTTGTTAAGATTTTGAGTTGAAAGAAGTGATTTTGTGCCACAGTGTAGTATCGATTCACTTTTGGAACAAACAATCAGGCATATGGTTTTTATGCAATCTGCCACGAAGCATGCTGCTAAAATGGATAAATATGCCGAGTTCAAGGTATGGCTTAGCTTCTTGTACAGTGTTTTTTATATGTTTTATGTTAACTGGTGTAAGGCTCTTCGTCGGGCCTGAACCAGACCGGACCAAACAATTCAATAATTAAGTACAGCAGTCGATTCTGAAAAATACTCTATTCTGCAGTTGCTCGGCAAAGAAACAGGCATACAAGGTTCAAGCTGGGCAATGGAAGTAGGGACTGAAATAAAAGTATGTCCAATAATCGTGGAAAATATTGGCACTGATGGGCAAATGTTGGTGGAGGTAATATAAATGAATAGTAACACAAAAGAACCTGACATATGTTTGAATTTTGCTAACATTTAGATCTGGATATGCACAGATGATGTGTGATGAATATGTCCACTTTTTGGAGATCACTGAAGCAATCAGGAACTTGGGTTTGACGATTTTAAAGGGTGCAACAGAAGCATGTGGTGACAAAAACTGGATGTGTTTTGTTGTTGAGGTATAGCGTTAGAGCAAATTAATCATCAAGTATCCATTCTAGTATTTCCTGAATCTAGAGAGTTAGCTTAGGTCAAAACATGTCAGGTCATGCTTTCAACTTTTATGCATGATTAATATTGAAATAATATAAGTTTTTCTTAGACAAGTTATGTGGTTGTATACATTTAAAACACTACCGATGCGTTTTGACACGTTTCCTGTTTTGACCCGTTAGACACATGACACCCTAAATCGACCCTTTAATAGATAATTGGTCAAGGTTGCCAGTTGCCACTTCTACCTGAAACAAACATACATCTAATCTCACTAATATGGTGTTTCTTTCCTGTATGCAGGGAGATAATAACAAAAGCATACATCGGGTGGATATCTTGTGGTCCCTTATTCAGATATTGGAACTGAAGACAAAGACCTGAGAGTAATTGTTTTGTAGAGTGGCTGATCATTTAGGATCGGCAATTTGTGTTTGATAGCATTTGGTTTTGAGAGTGTTTGTTTTTCATGTTTTTATATTTCGCTGCAACATTGATTGGTGGAGATGAGTGAAGAATGCTTCTTTTTCATTTTTATTTGTCGTGATTTGGCTTGTAAAAATTGCAACTTATGTTTGTTGTGTGTTTTGGGAATACAAAGTCCTATAGTTTGCCAATAAAAACCTAGAACAAAATAGATTAAGTGATGGGTGTAGTTCTCTAATCTTTTAGCTTGCACTTTAAAATAAATTTATTTATGAATCACATACATATCACTTCAAATATTGAATGTCGTCTTCTTTCTCTAATTTATATTTAGTATAAACCTTCAACTACAAGTAAAATAACGGGACTGTCTAAGAACTAGTGCTACTCGTACAACCTAATGAGCGTTCCAATACCTCGAGTAGAACACAGATGCTAAGATAAGTCTATCAGCATAATGAGACAACTCTTATAGCCGTAAAATCTAAGGAATCAATAACACAAAGTAAGCAATTCAGCAGTAACCCTTTCATTGGATAATTGAAATTCCCAGTAAACTTTAATTATCATAGTCGTATTCAACAAAAGCAACTAGCAATATATTAGAAAACGAGTTTAAATACAAAGCATTGTTCTCGTTATTGATAGCAGAAAACCCCTAATCGTAAGACAGGTGATATAAAATGTGGTTAACTGAAAGTTACCAACATAAAGCTTTCTGTTAAACGAATCATTTCATCTTGGCTTCAGTGAAGAGGACGTGACGGTTGACACGAGGATCGTACTT

AaMyc-bHLH10

TGCTCTCCACCTTTTATTTTTTATTTTATTTTATTTTTTTGATTTTTTGATTTTTTATTTTTACAATTTTTTTGGTATCAATAAACTATAAAACTGAAAATTTAATAAATCCAAAGCTTAAAACCGACAAAAAACACGAAACAGTAATTAATAAAAAACTGTGTTGTACTGTAAACTGAGAGTCAAAGAGAATTTTTTGTACTTTTACCCCTTTTTGGCAAGGCCGGATACATATAACATAACCCTAAAAATATTTTTTCGTATATAAAGAATCCAAAACATATTTATGATATAATATATTCTCGTGACCTTATTACTAAACGTCACATAAGATGTCGCTTGCAAGCACTCCGTCGTCGTTATAAAACATGCTGCATTATTGCCTTAAGGTAGTATTTATCATACATCACCGGTGAAGATTGTTCGATGTTATTACTTAACTCAACCACCATGGCACAATCCTCTCTTCAAACCCGTCTCAAGTTCATCCTCCAAAACCGACCCGAAAGGTGGCTTTACACCATTTTCTGGCAAGCTTCCAAAAAAACAGACGACCATCTTGTTCTAGAATGGGCTGATGGTTATTTCCCTGAAACCAACTATGTGTTTGGACTAGATGATGTTTCTGATACCCAATGGTTGATTATGTCATCCCTAGGGATGTGTTTTCCGGCTGGACATGATGTTGTTGGCCAGTGTTTTGGTTCAAGATCTTGCGTGTGGTTGGCAGGTGATATAGAGTTAGGGAAATATGATAGTAAAAGATGTGAAGAAGTGAGAGTTCATGGGATTAAGTCTTTGGTTTGTATACCAACTAATAACGGTGTCGTTGAATTGGGTTGTTGTGATGTTGTGCAACAAGATTGTGGTTTGATTGAATTGACGAAATCAGTGTTTGATCCGAATAGCTTACCGAATATCAACTTTCATAATCTTGTTGATGAATGTGAATTCCCTAATCAAGGTGAGTATAATCTTTTGTTGTTCTTTTGTACGGAGTAATTTTTTTTTTTATGGTTTTACATGATAACCATGAAGCAAGATCAAAGTTACATATGACTAGTTTTAATTAAACAATGAAAAATCTAGAAAGAAGATAAGATATATTCTGTCTACTATAGTTGAAATGTTTAATATAATTTCTAGTAATTAGTTCATGAAAAAAGATTGAATTATAATTGTATTTTAGTAGGCAGGGTGCAATTGGACTCCGGCGCAGCTTGTTGCTCGTTTGCTTTGCCCCTCTATGTAAATCGAAAGAATTGTAATATTTAAGGCTTTAAAACCCAAAAATATAACAGCTCCAAGAAAAAAAATTCCTAGCAAAAATATAGGTAGCTAGAACATCTTTTTTATGTGTATAAACACTAGTTAATCATCAATCATTAAATCCCATGATGGACTAATTCACGACAGTTCTATGGCTATGTAGTCCTTTATTTTTCTTAGTTTTGCCTAACAAGAGCTAGATCATTTTTAGGCCATCAAAATCAAGTAAGTGAAGGTCAGGAAGAAGTGATGAGCACAAAGAAGATGAGGATGTCCTCTTCTGATTCAGATCCACTGGAAATCAATAGCTCATCATCAGTAACAACCAAGAACACATGTACACCGAAGAGGAAGGGTCGACGAGTAAAGGGCACAATCGCTCAGCCAGAAGTATTGGCACCTGGATACCATGTGGAGGCAGAGCGACAACGAAGAGAGAAGCTAAACCATCGCTTTTACGCACTCAGGAGCGTTGTTCCATACGTTTCAAAGATGGACAAGGCTTCTCTACTAGCAGATGCAGTTACTTACATCAATGAGCTCAAATCCAAAATCCAAACATTAGAAAATAAAAAGGGGTCGGAATCTTCTCTAATAAGACCTAGAAATGAAAACCAACTGAACATCAATCAATGTCACCATGATCATGATAAAAGAACTGCTGGTCATTCAACGGTTAATCAGCTTGAAGTCGAGGTGAAGTTACTTGAATCGGAAGCAATGATTAGGGTACAGTCTGCGGAAGTTAATCATCCGGCATGTAAGTTGATGGAAGCACTTAGAAGCCTTAATCTAAAGGTACATTATGCAACTGTCTCTTGTGTTAAGGATATGATGTTGCAAGATGTTATTGTGAACCTTCCTAATGGATTCGCAAGTGAGGAAGACACCTTACGACTTGCGATTCTCAACAAAATGTGCTTGGATTAACAAATAGTTTTATTGAATGCTTAGTTTCTTTGAGTTTTATTCCATCTATTGTATCAGCATCTAAGTGGAATGACGTAATAGTGATTTATTTCTAGATTCTCAAAACTTTAATGTAAATACAATAAAAAAAGGCTAATCACACCAAGATGTATCATATTTGTCACTTATTCATTGTATATGTATCGAACAAATTTTTTTGCTATTCTGTGTACTAAACTTGATCTTTTAGGCATTTTTATGTACGTTATGACTATTTTGCCCCTCATTTTATTGACATAGGAGGGAGGGGCAAAATGGTTAAAAAGTACATAAAATTGTCCAAAAAATCAAGTTTAGTACACATAACAGCAAAAAAAAAAAAAAAAAAAAAAAAAAAAAAAAAAAAAAAAA

AaMyc-bHLH11

TATAAAGTTGCTACATTTTTAATGGGTATTTTCTTAAATTGTGTAAAAAGTTATCTTTTGGAGTGAAGTTTATAAATTTGTTATTGGGTTGGTGAAATTTTTAAGTAAAATTAGTAAGTTTTGGTTTTTGGGTGTTGAAGAAAATTTGGGGGAAAGATAATGGGATTAGATAGTTTTCTGAATTTGACAGTCGACCCACCGATAAAACGAAGAGCCGGGCTACGGCGAAAACAAGCCGGGAGAGGTTCATACAGAGGGAGCTAGAGTTTGAAGACAGGATAAAGCAAAAAGTTTTTAGATTTATTTAAATTTGACGGTTTGAGGGTCCTAATTCATTAGCTTTATAGTAGAAAGTGTTTGGTAATTTTTTCGAAAGAAGATGGAGGGTAGTGCATTGCATCAAGCACTCAAGACTCTTTGTCTTAACACTGATTGGAATTATGCTGTTTTTTGGAAACCGGATCCTCATGCTCGAATGTAAGATCATTTGAAAAACTTCAGTTTTTATATGTTTGTTGATTGTGTTGTTTATCTATTGCATATGTTTGTTTGGTGTTTTGGTAATTGATTGTAGATATGTCGTAACCAGCTCATTAGTAAGTGGATATTCTGGTCGTAGTAAAAATGATTCCTGATGATCTGATTATCAAAGGGTTAAATTAACAGTTTAGGAGTAGTTGTAATAAGGCTGACTCTTCTTATCTGATTATTTTCGTTCATATCACATTCAACCCTGAAATAGTTAGGATCTCCCTGAAACTTAAACAGTGGATGATTTGATTATCACAACTTCAAAGTATCACATCTGGACACAGTCTCGCGCAACCTGTTTGTTATAGATAATTGTACGGTTTTGATTAGTACATATTACATAATTACCTTCAAAACGCACTACCAAACATTCTTAATTTTCTTTTGTTATTCTGATGTGGGATCGGTATTATTTAGCTTCTTGGTTTTAGACAACTTAAAGGCTCATAGCTAATGGTTGTGTTATGTATTTTTTAGATGGGGTTAATAGTATTAAGTTTCTTGATTGTAGATTTGTAAGTTTTTCTTTTTTATCATCTGGAGATCAAAATCAGAAACTTCAAGAATTATGCATATGCATACATGCCGATTTGTGTTTGAAAATAATACACCTTAGGTTGTTTACTATTGCCCATGATATGGACTTCTTGTAGGATGTTGACTTGCGAAGATGCTTATTATGACCAAAATGATCCTTCAGGGAGCAAACCATTCAATAAGGGGATGGACAATTTACCTGATCAGCAGTACGCACAAGACATTCTTGACCTCGCTGTTGCAAAGATGTGTTCTCGTAGATATTCTTTCGGGGAAGGGTATGTTAATACACTTTACCATTGTTTTCTCACTTGAATGGGTTGATTTGGCTTTTGTTTTATCAACGGGTCAAGTAAAAAAAAGGTTCAAGGGTAACTGGTTAAACAAGTTTTAAAAGTGTTCGCGGCTCAAGTGCTCAACCTTACTAGAGCCAGCCCATTTTGACCCGTGCAAAAAAGTGCATTTTAAACACGTCGGACCAGTTACCTATACACACCTGACCCACCCATCTTGGCACCTCTATTAATTATGTTTGCTCATCATTCTAATAAATCCTTTCGCTGGTAGAATCGTTGGTCAGGTTGCAATCACAGATAAACACATGTGGATTTTCGGAGATCAATATGTTAATAATCCCGACTTGTCCTTTGAGGTAAGGAATAATTCTAGTACCAATATTTTTGTGTACAAAATATTTGTTACCATTCTTCCTAGTGCTGTATACATTGTTATAATATAAATTGAAATTCGTAATTTTTTTTTCAGGATTACGATGAATGGAAAATTCAGTTTGCCACTGGGATCACAGTATGTAGTCTCTTTAGTATAAAAGTTAACTAGAAACTTGTAGATAGTTAGAATATCAGGATCAGGTTTTTAATTCATAAATTAAAGATGTTTCTTTTGTTATGCAGACGATTGCTGTTGTTGGTGTTGTTCCTCATGGAGTTGTACAACTTGGTTCGTCAAATCATGTAAGATGATTTCTTTTTCCTTATTTATATTCATCAATATACAAAATTAAAGACAAGACTTACAAAATAAAGATCGTATAAATGGATCACATGACCATAAAGCTAGAAACTTAAAAAATATTCCGTTTGAATTGAGCAGATACCCGAGGATCTAAAGATGGTGAATCACATCCGAGATATTATTCTTGATCTGCAAAATTCTTTAATGGGATGTACACCTAGCAATACTAGTTCATATAACGATACCATTACATCGCTAAATTATGGTGCCAACACAATATCAGATCTTGATCGAAATGTTGATTTATGTATGCCTGGTTCTTCGAATGTCCAAAGTGAAACCGACTCCATGAATACCTTAAAAACGTCTTTTAAGTTTCCTGCTGGATACGAGTTGTATGAAGCACTGGGACCTGCATTTTATACCCAAAACGATAACCATAGTTATCAGAAATTAGAGGAGATGCCGGGAACAAGTAGTAGCAATCTTCTGACACCAAATCCAGGATCAGAACATCTTCTAGAAGCAGTTGTAGCGAATGTTAGCCAAAGTGACAGTGAATTTAGTAACTCAGTGAAATGGGTTGAGACTCATTTTAATGATATGCAGACTAGTACTTCAGGTTGTTACTCGTTTGAAAGTTCATTAGGGTTCTCGTCAGCTAGTCATAGTAGATGTAGTGACCAACGAGTGATGTCACAAGAATCACCTGCTAATGTGACCAAGAAACGGGCTAAACCTGGTGAAAGTAGTAGGCCTCGGCCCCGTGACCGACAACTTATTCAAGATCGAATTAAGGAACTCCGTGAGCTAGTGCCTAATGGATCGAAGGTTACTTCTTTTGCATATATGATATGTATTTTTTATTTATTATTATTATTATTTTTTTTTTATTGTTTTTGGACTTTCTATGTTTACTGAAATGGGCATTTTATGGTACAGTGTAGCATTGATTCACTTCTTGAACGAACAATCAAGCACATGCTCTTCATGCAGTCTGTCACTAAGCATGCTGATAAAATTGATAAATATGCAGAATCAAAGGTATGGTTTTGCATTTTGTCTTTTAAATGAAATTACTTTCGGTTCGGATGCAAATAGTCATAGCCTTTTTTTATAATAGTCATTTGCATAAGATAACAATGTATTTTTTCATTTGTACATACGAGTCGTTAAAAGTTGGTAAGGTATGTCCCCATTTGAGTCATTTTGTAACCTGTAATCTACAAATTTTGACATTAAAAAAGTCATCAAGATTGTATATCAGTATAACATTTGTATACATAAATCTAACAAGTTGATATGAAAATATAACTTTTGGAAGTTGCTTTTGAGGGTTTTGGTAAATGACCCAAACGGGACACAAACTTATCTTATAATGAGTAAGATGTATAAAATTAAAACAGGACGAGAGAAAATTCTGGTGCTATTACGTTCCCAAAAATACCCTTTATAGTATGATTAAGATTAAAGGTGGCAATTTCAACCCAGTTTCGCTTATTAATAAGATGATTCTAGTGATGTTTTATTATCTCTACTGGGTTAAGTGAGTTAAACAATTTAAACGTTATTCACGAAAAGAAATGTGTCAAATGGGTTTAAGGGTTCGAAAGTGGTATTGCTGAACTCTTCTCAAGAAAATTGAATTATTATTGTAAAACTATATATGTATTCATATTAATAGGCTAATCAGTTATAGGATGGTTATGAGGTTATTTATTTAAGAGATGCATATCACGATTACTTAACCACCCTCAAGGTGGCCTAGTGGTCTCGCATCCTGATTTCCTCAAAAGAGGTCTCATGTTCAAACCTCACTAGGTAAACATCTTGGGGATGGCCAGGGAAAGGGTCGAAAACGGTCTCGGGGACACCCTGTTTAGGCTTCGTACAGCTGAGTCAAAATTTCACCCTCCCGGGGTATCCTGAACAGGTTTAAACCTCATACGTTTACCCATTTCACAGTTGCTTAGCAAGGAAACAGGCATACGAGGAACCTCAAGTCACGAGCAGGGTTCAAGTTGGGCAATGGAGGTCGGAAATGACATGAAAGTTTGTCCTGTGACTGTAGAAAACATTGGTACAAATGGCCAAATGCTGGTGGAGGTATAGCCGGTCTTCCAAAATGCAAATTCTGAACATTCAGTTTATAATTTCAGAGTACTAAAACTGATAAATTTATGTGATGCAGATGATATGTGAGGAAGGTGTCCATTTTCTTGAGATAGCGGATGCCATTCGGAGCTTGGGACTCACCATTCTGAAAGGGGTTGCAGAACCTGATGGGGATAAAATGTGGATGTGTTTTGTTGTAGAGGTATTGTTTCATGTGCAAAATGACTGGGTACTGCGGGTTGGGTATGAGTCTAAATGGGTCAGGTTTCATGTGCAAAATGACTGGGTACTGCGGGTTGGGTATGAGTCTAAATGGGTCAGGTTGGGTTGACTTGAAACACTTTTTGCTTACAAATATAAGTAATATTATTGTTGCTAGACACTAATCCATTCTTTAATGACTAATGCTTTAGGAAGTTAGATGAGGTAAAACAAATTTAGGTAACTTTCAACCCGATTTTTAGCTCATCAACCGATTGTCTTACCTTTTCTGATTTTAGCGGTTTGTCTCATTAGAGATGAAATATATCCTGAAATGAAATGTAACCTGAATCGATACATTACTTAGTACTGAATGAGTAGAATTGTCATAATATTTACTGTCTTGTGTTGATTTTAAGCCATCGATTCTATATGCAGGGACAAAATAACAGAAATGTTCATAGGATGGATATACTTTGGTTACTTGTTCAGATATTGCAATCGAAGGCAAAGTCTTAACTTGATTAGTCCTGTAATATTTTCAAGTTTTTGCTGATAATATATTAAAAAGATTCTTGTCCTCACTTTTGTATGCTGGTAATGGGTCGAGACGGGTTTTTGTTATTGTTATTTCTTGCCATTCCATAATGTAATTTGGCTTGTCTTTTATGTATCACTTTGAATTTAGGCCTCCCATGGGTCTGCAAAAACACATTGTGTATATTTGTTTAACATGTTCTTCATATTATCTTGTATAGTCGTCAACATGTATTGGGGACATGTGAATATGTGATAACC

AaMyc-bHLH12

TGTTTACAGGGTATCTATAGTACAGTGGTTTTTGGATGAATGGTGTGTGAACGTGTGGTGCTATTTTCTTGCACCTAGGAATAGTACCACTTTTTAAAAATATTTTTCGTTAATAAAGAATCCAAACCATAATTACTGGTATGACATAATTTATTCTCCTGACATTACTAATAAACGTCATGTAAGATGTCGCTAGCAAGCAAGAACTCCGTAGTCTTTATAAAGCATGCTGCATTATGATAGTATTTATAATACATCATCGGTGCAGTTTGTTCGTTGGGATTACTTAACTCAACCACCATGGCAGAATCCTCTCTTCAAACCCGTCTCAAGTTCATCCTCCAAAACCGACCAGAAAGGTGGCTTTACGCCATTTTCTGGCAAGCTTCCAAAAAAACAGACGACCATCTTGTTCTAGAATGGGCTGATGGTTATTTCCCTGAAACCAATTATGTGTTTGGACTAGATGATGTTTCTGATACCCAATGGTTGATTATTTCATCCCTAGGGATGTGTTTTACGGCTGGACATGATGTTGTGGGCCAATGTTTTGGTTCAAGATCTTGCATATGGTTGGCGGGTGATATAGAGTTAGGGAAATATGATAGTAAAAGATGTGAAGAAGTGAGAGTCCATGGGATTAAGTCTTTGGGTTGTATACCAACTAATAACGGTGTCGTTGAATTGGGTTGTTGTGATGTTGTGCAACAAGATTGTGGTTTGATTGAATTAACGAAATCAGTGTTTGATCCGAATAGCTCATCTAATATCAACTTTCATAATCTTGTTGATGAATGTGAATTTCCTAATCAAGGTGACTATAATATTTTGTTGTTCTTTTGTACGGAGTATTTTTTTATGGTTTTACATGATACCCAATACCCATGAAGCAAGATCAAAGTTATATATGACTAGTTTTAATTAAACAATGAAAAAAGCTAGTAATTAATTGAGCAAATAACATATTAAAGTACTATATTTGTCAAGAAAGTCATTTTTGGGTATCGAACAAATTTTTTTCCTCATATGGGTACTGAACTTTTATTTTTCGGCCTCTTTGAGGCACTTTGTGACATTTTTGCCTCTGGATTTTTAATAATTTGAGGGGTAAAAACGTCACAAAGTGCCTCAAAGAGGCCAAAAATTGAAAGTTCAGTACCCATATGAGGAAAAAAAAAATTGTTCGATACCCAAAAGTGTATTTTAGGACAAGTACGATGATTTTTTATAGTATTTGCCCTAATTAATTCATGAAAAAAGATTGAATGATAAGTAATTTAGTAGGCAGGGTGTGTGCAATTGGACTCCGGTATATTGTATTATTGTAGCTAGAAATTTTAGTTATAATATAGGTGGCTAGAACATCTTTTTTGTGTGTGTAGACACTAGTTAATCATCGATCATTCGATCGCATGATGGACTAATTCGTGCCAGTTCTATGGCTAAGTTGTCCTTTATTTTTCTTAGTTTTGCCTAACAAAAGCTAGATCATTTAAATAGGTGAAGGTCAGGAAGAAGTGATGAGCAGAAAGAAGATGAAGATGTCCTCTTCTGATTCAGATCAACTTGAAATCAATAGCTCACCATCATTAAGAACCAAGAACACATGTACACCAAAGAGGAAGGGTCGACGAGTAAAGGGCACAATCGCACAACCAGAAGTATTGGCACATGGGTACCATGTGGATGCCGAGCGACAACGAAGAGAGAAGCTAAATCGTCGCTTTTACGCACTTAGGAGCGTTGTTCCATACGTTTCAAAGATGGACAAGGCTTCTCTACTAGCAGACGCAGTTACTTACATCAATGAGCTCAAATCCAAAATCCAAACATTAGAAAATAATAAAGGGTCGGAATCTTCACTGATAAGATCTAGAAATGACAACCAACTGATCATCAATCAATGTAACCATGTTCATGATCAAAGAACTACTGGTCATTCAACGGCTAGTCAGGTTGAAGTCGAGGTGAAGTTACTTGACTCGGAAGCAATGATTAGGGTACAATCTGCGGAAGTTGATCATCCGGCATGTAAGTTGATGGATGCACTTAGAAGCCTTAATCTAAAGGTTCATTATGCAAGTGTCTCTTGAGTGAAGGATTTGATGTTGCAAGATGTTCTTGTGAAAGTTCCTAACGGATTCACAATTGAGGAAGACACCTTACGACTTGCGATTCTTAACAAAATGTGCTTGGATTAACAAATAGTTATATTTAATGCTTAATTAGTTTCTTTGAGTTTTCATTCCATCTATTGTATCAGCATAGTTAACTATCAAAAAGTACTCCGTACTGCCATGTGGGTATTTAATGTTGGGGAATTCCTGATCGAATGGATGTGGCTGTAAAGCGTGTTCCAGAATCAGGAAAGCTATCCTAGTTGGAGTAGAAGTCCAACAAGTTTACTTGACGTGCAAGTAGTTATCTTCCTGTAGGACTTAGATAATTATAAGGTTAAGTAAAGTGTAATCCTAGTTATAGTCTAATACAAATAGATTAGTCTATATATACCGTGGAGGGTAGGGATTCTCATTGTATCAAATTGGAGTTTTGAGTGAGTTTCTTCAATAAAACAAATCTGCGTGTTTAGGCAGTAACATACCTGTGTCGTGTTCTTGTTATGTGCTTCTGCTACCTGACCTTCTGATTACCACAATTGGTATCCAGAGCTTGTGAAGAAGTTCCTGGTGATTTGTATCCGAGTTTGGGTTACAATACGTGGGAGAAGAGCGTTCCTAACCTGTGATCCAGTGGTTAGACGTGAATTATTTTTAGGGTTACAGGAACCTTAAAAACAAACGACTTAAACCTGTGAACATGTTGGTTGGTCGTGATTTGCAGCCCACTGTGATTGAAGCCCACTTTACAGCCCACTTTTCTCTTCTTTTGTAAACGGATTACAGTTTCTTTTTTTTCTTTCTAGACAGTAAGTTGGAAGCAGTTTTTAGGACTGTTGATTGTCTCACTGTCTTAAACTAATTCTAGCCGTGATATAAGGGCTGGAAAATTCTCCATTACAAACCTATTCGCGATCCAGAAGAAGAACCAGGATGACTGGAGATCCATCAGGTTCTAACCCACCATCAACGCCAACAAAGGAATCCAATTTGACGTTCCAGTG

AaMyc-bHLH13

ACATAACTCAACAAGACACTTTACCAAAATCTCCCTTTCTCTCTCTTTCTCATCTTTTGAGCACTTGAAAAGCTGTAAAAAGTTTGACATTTACAAAAACATGAAAACCCTCTTAAGAAAGCTGTCTTGTTAACAAAACCCAGTTGAAAATGATGACATGGGTTTTGATTTTGGGTGGGAATTTGTTAAGAACTAAACAAGCTGGAAGAGGTTCTTGCAGAGCTAGTTAGGCTTTTTATTATTATTATTAGTATTGATTTTTTTTTTGTCGGATAATCAATGGGTTATTTGTTAAAAGAAGCACTTAAAACCCTTTGTGGTGTTAATAATCAATGGTCTTATGCTATTTTCTGGAAGTTTGGCTGTCAAAACCCTAAGTAAGTTTCAGATTTTTACTATTTCTTGTTATTAATCTTGTTTTGCTCCATGGAAATTGACCTTTGCTTTAGGGGTGTTGAGTTTTATCATCTCGGGCTCTCGTCTTTTAACTTTGTGTACATTATCTTTACATTGTTCCATACTATCTAATTCATGTCACTTGGAACTCAAAAGTATCTCCTATTAACCACTATGTCAAACCAAGGTGGCTAGATTAATCCTCTACTTTGGTTGATCTTGTAGTAGTATTGTGTATATAAGAGATCTGACATGTCATTCATTCTTTGGTGTTGTATTCATATGTTTAGATTGTTTTGTTTTGTCTTGGTCCATGTTAGTGAGGAGACAGGGCAGGTTTGTTGTGGTCTAGTGTTTTTATTCTTCCTCCTTAGGGCATACCTCCATCTATGTTTTCAAGGACATTAGTACAAGGTTACGGCTGCTTGATATGTCTATATCTGGATTCAGATATTCTGAGATAGTAAGGGTAGGATACTTTTATATTTGTAAGATAAATGCAGTAAAATCTAAGTTGGTGTTACTTTTACTTGTTGTATCCGTAGTGGTCCAAACTATCAGAGAAAGTAACGTGTCATGTCCTGGTTGAAGGGCATTTTGGATATTTGACTAACGATCCAACACTTGAATTATATTTGTAGGTTGAAATGTTATAGTACTCCAGGGATGGATATAATTGCAGAATATGCAGACTAATTAGTTTTTCTTCTTTATTGTAGGCTTCTAATATGGGAAGAATGTTATTACGAACCTGTTATTTATTCTACTGGTGAGCATGGAATTCAAGCTACAAACAATATTCACTTGCTTGTAACCAAAATGATGAAAGACAACTACGTCAATCTTTTAGGGGAAGGGTGTGTTTCTATGTTTAAATTTTGATTTTTATTATGGGTTTTTGTTGATTAAATAACGCAATATTTTTCAGATTAGTTGGGAGGGTTGCATTCACTAGAAATCTTCAGTGGATTACCTCGGATAACAAAATGCTAGATGTCCATCCACCTGAGGTATGAACTTTATCTTTGTAGATATTTACAGGGTGTTCTGGAACTGATTATCTGAAGTTGTAGTAAATAGTTTTTAGTTTATGGGTTAAGAGAGTTGGCTTTTAATTATTTCTATATTCTGTAGTAAAAATAGCAAGTTCCATTTCAGTTTCAAGAAACTTATCTGAGGGCATATTGACATTACTTATTTTCCCTTGCATGTGCTACAGGTATCAAATGAGCTTTGTACACAATTCTCAGCTGGCATTCAGGTAAACTATTTCTCTTATTATTCAAGTATAATTATAATATCTTGCTGTTCTATTGTAATGTTTCTCTATTTTTTGGCACCAGACGATAGCAGTTATTCCGGTATATCCACATGGGGTTGTTCAACTTGGTTCATCCTCAATTGTAAGTACATCTTTACCTTCCATTTTTACAAGTAATATGCTCGCATATAACTTGTGAGTATTTACGCATTCTTGAGGTTACTAATACCTTAGTATTATGCAAGGGTTGTCACCTTACATATAATGATGCAATCTGCTGTGTTCAAATGTGTGCAGATTATGGAGAATATGGGATTTGTGAATGATGTGAAGAGTTTGATTTCTCAGCTTGGATGTGTTCCAGGTGCCTTATTATCAGAGAACTTCATGAAAAGCGAAGGCACCTCGGACAGTAGTCCTAGTCAACGTGAATCCATCTGCCAAATGAATTCTGTATCTGCTCATTCATCAGGAAAATCTAAAGTGACAGACTATAACCAACAGGGCCACTCACCTCAGGCTTCTGGAAGTAGTGGACATACTTCTTTCTCTCTTAATAGGCATATTGGAGCTAACGCTGTGGCCAGCGGTTCAACACGTGTAATATCTGATGAAGATCAATGTCAAGCAAAAGTCATTCATATTATGAAGCAAAATCCCTCATTCAAAACCAATCAACAAATTGGAATCACCGGAGTAAAATCAATCACTGGGGCTAGTTTGTACAATCAGAATTCCAAAAAAGAGCAACAAAATGCTTTTGGCTCATGCTTATTAAAGTATGATCCTTTAACATTGATGGAACAGGATAACTTATCAGATGCATTTTTGGGAGATCATGCGAATGACACCATGATGTCCCAGATTGCTCAAACTTCTATTCCAGATGCTCACTTCTCTTGCACCCATGTTGGTAAAATTTCCAAACTTTTGATTTAACTAAAAACAAAATATTTATATGTTGTTCGTCATAGAGTTTGATGTTCATGAAGCCTCAGGTTTTATAGGCACAGAAATTTTGACCCCTTTGCTTATGTATGATTCGAATTTGATTATGTTTTATCTCAAGCGAGTCAAATCAAAATTATTTGACTAAGCAGCGAACATGTTGAAGTTTTCCCAAATTATTCTTTATGCATGACCTCTTGGATTGGCTAACCATTTTTATCACTATTCCATTAATGGACTTTTGCAATAATAAACACATTAGTTATTTATAAAAATAGCCATAAGATAGGAAATTACTGTTTGTCTGTCAACCCGATCAACTTTTGTTTGGACCCCTGCACAAAGAATAACTGTGGTGGCTGATCCTTGTGCTTTCTTGATTAATGGTGGCATTAACCATGTGCTTCATCATGCAGATAAGGAAAATCTAGATAATGAACTATTTCAAGCTCTTTCAGTGTCACCAGAAGAAGCATGTTTAAAACCTTCCACTAGGGATGACTTGTTTGATATCGTGGGTATGGATTTTAAAAGCATATTATTTGGTGGAACTAGGACTAACTTTGTTAATAATGGAGTAGAAGCAAGCCCGCTAAATTTAAAGAGGAATGATAGCGTGGTTTTAGAGCCATTCTCTGTGTACGAAGGAGAATCTGATAGTGGTATATATTCGTCAACACCTAGTGACCATCTTTTAGATGCAGTCGTCTCTAAGGTTTACTCATCTTCGGGACAGATTGCAGATGATAATATGTCTTGTTCATCGTTGACAAAAACCAACAGCTCATCATCATCTATGTGGGCTAATAAGGGGATAAATTTGATGCAAAAAGAGTTGACTGGTGTTCCCAAATCTTTGAAAAATGAAGGGCTAACAAATTCTTGTTCATTTAAATCTGTGATCTTTGAGGATGGGGGAAACTTTTCGGATACTAGTTCTATCATTTCATCCCAAATCAGTTCTTGGAACGAAAAATTGAGAAAGGACAGCGGTGGTGCATCCCAGTATGGTAAGAGGGCTGATGAAACTAGCAAATCAAACCGTAAGAGACTTAAACCTGGAGAAAATCCTAGACCAAGACCAAAAGATCGCCAGATGATTCAGGATCGTGTAAAAGAATTGAGAGAAATCATCCCGAATGGTGCCAAGGTAAAACACCTTTGACCTATATATAGATTCTTATTTTTATTCTATCTTTTTAACGTTTCAAGAAAAAAACTTGATACCAAAGGTGGCAATGCTGACCTATTTACTTCTAGATGGCTAGACTTGGGTTAAGATCTGTACCTAACAGGTTAAATAGAAAAGTTAGCTAAGGGAAACCGGTAGAAAGTAGCAAAAGTGTATTTTTTTCTAATGCTTATAGCCTCCTAAATTGTTTTATCAACAGTCCATTTCTACCCAAACCCTTTTGACCTGTTAGGCCATTTTGCCTCCTATAATTTCAAAACCCCAAGGGATTCCTAGTTGCCAAGTTATCAGCAGTTGTTCTTTAAATTTACCTAGGTCATGTGAATTGGCCGGTTTGCACGACGTATTAGGGAAAAAGAATTGACATTTTTCTGTTTTTTTTGTCGTTAAAACCAGTGTAGCATCGATACTTTACTAGAACGGACCATCAAGCACATGCTTTTCTTGCAAAGTGTTACAAAGCATGCAGACAAGTTAAAACAAACGGGGGAATCTAAGGTATGGATTTATATGTATATGTTACCATTTTGTTTTGATGTTGACTGACAAATCTGAGTAACATTATGTGTAAAAGAGATCATTCACTTTGATAAGTCATAATCTGATGTTTTTCAGTTTGTTTATTTCAAACTTCAAATGATTTTTCATAGTTATGAGACACGCATCTTGTAGAGACGTTAGAATTTGCTGATTCCTTGATTGTAGAATGAAACTTATACAAATGCGAAAATGGGATAAACGGAAGCAATTTGGTCTTTCTGGGGCAAAAAAAGGAGACGAAATCTGAAAAATTAGAGAATAAGGCTAAATGACTAAATATTGTGTTGAGATTTTGGAGTAATAGGAACACAAAAGGGAAATTCGAAGGACAAGGTTAGGTTAAACTTTAGTAGTATGGCTCATGATTAGCAATTTTGTTTTCATTGTTTCAGTTTACACTTGTGTATGTTTTAGAACCGTGAAATTAGCTAATCAAAATCTGATGATCAATTGTAATTCGGGTGCTTGGATGTGCACCTTCAAAGTATTTTTTGTGATATTGAATATAACTCAAAAAACGACCTTTGGTAGATAGTGCTTAGTCTGTTTGTACTTTGAAGTTGTAATAATCGGATAATCATGGTCACACATATATAAATCAATACCCATACACTATTACGCCACATCCCCTCTGCATTCTGTTGCAAGATAATGGCTACATATTATATTTTTTTGGGTAAAGGGTTTATACCCAGTGAATATAATGGCTACATATTATATGAGTAGCCACTCCCTCAAAAAAAATCCAGAATAGTTTACAGCTAAACGACCTCATTGATATCGAAAGTCAAGCTATAATTCTTATGCTGGAAGCCAAGAAGTATACCAATTTCTGTAGAGGTAACACACTTCTATGACTTATTTTACTTTTAGATTGCCAGCAAAGACGGTGGCTTATTGTTGAGGGACAACTTTGAAGGAGGAGCAACATGGGCATACGAAGTTGGTTCACAATCAATGGTTTGCCCCATTATTGTTGAGGATCTTAATTCGCCTCGTCAAATGCTTGTCGAGGTATGCACTGCTGGTGGTCATTTTATCACACAAAGGCATTGGCCCTTCCATAAACTTCATCATATATGTATATTTCCGTTAGAGATGACAAGATGGTCGGGTCGGTGGGTAATGGGTTGGGTAAAAGGTCAAAGCAGGTTAGGTTGACCCCAAAAACTTTCTTATATGTGGTTGATACACTAAATTACAAAATTATATTGTTACAATTAATTTGACAATTTGGGTGGCTTCAATTCTGTTATAAGCATTTGACCCGTTCTTTGGATTGGAATTTGATGAATCATAACCCAAGTCATATATTTAGTATCGACTTGAACCAGATGAATCATAACTCAAGTCTACAATGTTTCTACATATGTTGCTTATGTGTCCATGTGTCGGTTTGCAGATGCTTTGTGAAGAAAGAGGTTCGTTTTTAGAGATAGCCGATGTTGTTAAAGGATTGGGATTGACAATCTTGAAGGGGGTGATGGAGTCTCGTAATGGGAAGATATGGGCACACTTTACTGTAGAGGTATGTCCCGAATGTCTCTCTACAATCAAATCTACTAACTTCAGAAATTCTGACGATTTTTTAAAGCCTTTTATTGTGTAAACAGGCTAACAGGGATGTGACACGAATGGAAATATTTATCTCACTAATTAGTCTCTTCGACCCAGCTGCAAAAAATGATGCATCAGCAGCTGGATGTGATGCGAATGGGAATATTTCAGTCCACGAACAATCTTTCAACCAATTAGCGACTAATCCCGCAACTGTTGGACCACATAATATTATACAGTAATGCCACTGTGAGGCTTGGAGATGTTGAGAGCAGCTCTGCCATGACCGACATTGATCCCAGCCTGGTCTTCATGATCAACCAAGTGTTGCTTGTTATTAGCTCGTTGTTGGATTGACTAAAACATATTGCTTAGAGAATTCGTGTTAGTTTCTTGGAGCTTCAATGCCCATGTAGCTGAATCTTGGTGGGCATTGTCATGTATTTATTAATTGGTTGTGTTTTAAATTAGTTTGCCCTATGTTTTTTTTGCTACATTGAAGAGAGTGAACTGATGGGCCCGATACGTTAAAGACACTTTATGTGTCAATTGTATTGCAACATTTTCTATCATAGAAAAACAAGAGACCTGTTGATGATACAGTTGAGTTGCCTGAATACTTGAACTTAAAACTTGCACATGGACTTATCAGAATGTCACTTAAGTCACTTGTTTCCAGGTGTGTCATCTGAGTTAGACCATACATCTTTTAGCTAGCTTACAATGTTCATGACGACAAAGAGGGCTAACCTTGCTTAAAGTTCTATCAAACTTCTGATACTAAATAAAGAATCAGATTATAAACAAATAAAAAGCAACATGGCATGA

AaMyc-bHLH14

ATGTTCTAGTTCCGCCACTGTTGACACCCCTAGTAATTACTCCCTCCGTCCCAAAATTATTGTCCAGTTTGAATTTTCAAAGTCAAACTGGTTCAACTTTGACCATAAATATATTTTTTTATTATATAATACTTGATGAAAAATTATACCATTGAAAAGTATACTTAAAACCCAATCCATTTATATATTTTGCATCAAATATTATATAGCACCCACAAAAATATTTAAGGTCAAAGTTAAAGAAAAAAGACTAAAAAGTCAATAATGAACAATAAATTTGGGACGGAGGGAGTACAATTTAGAAACGGGAAAGAGTTTTTTCAAACAGTATATTAGATGTCTTTATTATCTTCATCATTGATATCAAAGAAATTAGAATAATGGTCATGAGCCCACGAGTTTAGATGTTATTCCATTTTATATATTTTGAGCGAAAGGTAGGGATTAAATCATTGAAACAACCCAGTTGTCTGAATAATATTATTAAATAAGTTGTGAATACTACTTAGGAGGATGATTTACCAAAATCTAGTACGTAGTATATATTCTCCCTCCCGTATTTCTATTATCTTTGCCTCACACACCTTTTGTTTTCATAACCAAACCATACGCATTACTATGGATGATGAAATCTTAATTCTTTCACCTTCTTCTTGTTCTTCAATCATCCACCAAAACACACACAATTCACACCATAAACTACAATTCCTATTACAAACACAACCCTACCCATGGGCCTTCGCCATTTTTTGGAAAACCACGTATAACCATGACTATGGTCGACCATGGACCTTAACATGGGCCGATGGTTACTTTCTTCAAAACCCAAACAAACCCGCCTTTAAAGAAATCCAAACACTAGTTGGACCGGACAACACAGACGGCGCAGAGTGGTTCTACGTCGTCTCATTGGCCCGATCCTTCGGTATTGGGGACGAGTCTGCCCCATCAAACTCCTTCACTTCTAATAGTGTCATTTGGTTAACGGGCGCTCATAGTCTTATATCTTTTAATTGTGAAAGAGCTAAAGAAGCTTATATTCATGGGCTTGAGACACTTGTGTATATTCCTACAACCAATGGGGTTGTGGAATTGGGGTCTTATCATGTGATTAATCATACCGAATCTGATTTAGCTCATCGAGTTAAATCACTCTTCAGTGCTAGTTCTTCCTCTTCGTCATCATTTTTGTCGCCATCTAGCAATTTGATTAACCAACCCAATGACTATTTGATCTCCTTCGGAGAAATGGTCTCTAGGTTGCCACAAGATGAAGAAAGTAAGGACATATTCGATTTAGGAACAACCACGTTTGGCCAACAATCCAAAAAACTTGGAAAAGTAGTTGAAAACACAAACAATAAAGGACAAAAAAAGAGTCAAAAAAAGAAAGGACATGATCCACCTCTAAACCACGTAGAGGCGGAGCGTAAGAGACTTACGAACCTTAACCAACGCTTCTACGCGTTGCGTTCAGTGGTCCCATATGTTTCAAAGATGGACAAGACATCCTTACTGGAGGACGCCGTTTGTTACATCAATGAATTAAAAAATAAAGTTGAGGAACTTGAAGCTCAACTCCAAGCCATGAACAACCAGCCCAAGTTGAAGAAAATAAAAGTCAAAGCACCAGATGTGACCTTTGTTCGAAATTCACAAGGTGCAAATATATATAATAACGGGAATCGAACCAAAACGAACGGAACCTTGGAAGTGGAGGTGAGAATGGTGGGTGATAATGCAATGATTAGAATACAGTCTGGTAACGCGGATTGGCCCTCTGCGAAACTGATGGATGCGTTACGAGAAATGGAAGCTAAAGTGCACCAAGCGAGCATGTCCTGTATTAATGACGTAACGCTTCAAGATGTAGTCGCTAGGGTCACTGGTTTTACAGGGGATGAAGTTAAAAANAGCTGACGTAACAGTGAAATTGGAATAGATTATCTCTCCTTACTGCATCCAGCCTTAGGCTGCCCCAGTAACGGATCCCAATCCCTCTCATTTTCTCTGCTATGTCAGCTTTCTCTCTCCAGCAACCTTCAGGCGATTGGATGCCCCAGTAAGGGTGACCATGATCAATTTTTTTTAAACATTTTTGACACAAACTCAAATTTCATTAGCAATTAAAACGTTACATATTAATTCAAATAAATTAAAAACATTACAACACTTATTAAAAATTACAACACTTATTAAAACTAATAATCTAGGTTATGGCATGCCTTGATCGAATCTTTGATTTCCTGCATTTTTTGTCGTTGTAATGCGGGCAAAGATGGGTCGATTGGTGTGAGATAGAATATGATGTCTTTTTTCCGTTTCTTTTCCTAGTCAAATTCAAATCTTTGTTCTTCTAGTTGAATTTTTCTTTCCTCCATTTCCGCTGCTTTCTTATCTCTTTTTGCTTGCAGCTCCAACTCCATTTGCTTTTGCTCCAACTCTTTTTTCTTCAACTCGTAGTATTCGTCTGGCAACTCCTTCTTCTTTCCTAATGCCATGGACTTAATGGTTTTTCATTTGTCGGCTACCCCATCCATATACGAAGACTTGGAACTCTCCGACGATTTCTTCTTATTTTTCTTAGCGGTATTACGAACAATTGGTCGCTCATTTCGAGTCTCTTCATTATCAGGCACATCCGAGCCGGTTTCAGAAGTCCTTGCTTTTTTTTTCGTGCCGCATCTTGGCTTAAAAAAGCCGGCATCTCATTATCCTTCCAAATCTCATGATCCCTCAAAATTTCCCAACAATGCTCCAATACAAACGCATGCATATACACTATTTGGTATTCTTCACACGCTCGTCTGTAAACAGTGAGATCATTCTCACCACTTCCAAAATTCTTATAAGCATTGTCGTAAATGGCACAAAATGCACCAACTCTAGGACGCATCCTATTTTTCCACTTGGTAATAATCGAGTTGTACGTTCTGTTTGCACCAACCTCTTTTTCAAAGTAAGTACTCACTTTCTGCTCCCTTAGAATTTTGGTTGGTTCCTATTATGCTATTATTGGACGAATCACACCAAGTATTGCACAACGCGATCTCTTCTTTGCGTGTCCAAATCGACGAATCATTCTTGGTTTCTTTCTCAACAGTCTTCTCCTTAGCCTTTGTAGCTCGTTTCTTAGGTTTGACCGGTGACATCTCTTCCAAAAGAGTGTTGTCATCGTCATCATCAGCAACATCATCATTTGGAACCGAATCGTGATACGATCCATGAGCCGAACCTTGACCCATAGAATACTCTTGACTCGGATAACCTTCACCCGGATAACCCGAACCAGTTCCTACATAAAAATCTGGATTGTTGTATAATTAATCCATAAAATCACAAGCATCAAGATTCATGCGGTTAACCGGAGAATAATGTTGATAATTTGAATAGGAAGACGTTGATGAATCCATTGTTGGATGATACACCGATTCGAGTGAGCCATACATAGATTGTTGGCTTCGACGGGAGTGGCTTTGTTGCTGTTGTTGGTTGTTTTGATACGGTGGAATAAATGGACGATTGATAGGACGATTTATAAAATATGGATTGTGAGTTTGTTGTTGTGGAGGTTGTTGCGATTGATAATCTAGAGTAGTGGGTCGGAAATTGACATTTAGGTGTGAGACACCGGTTAACCGAAAATTCGGATCCGATGTTAATTGCAGGATGTTTTGTGGCAATCGGGATTTTCGGCCTTTTGGTTGTGGTGTTTTCTTAGACATTTTGAATAGAGAGAATTGTGAGATTTTGTGGATTGAAGTGTGTAATATGTTGTGTTAATATGTGGTATTTATAGTGTGTTAAAAGATTTTTTTTTTCAAACTGTTGTGTAACGGTTAAAGGAGTAAATGCACAAGAAAATGAGAAATTCCAATCGATCGTGTATACGACCAAGATAAAAAAACATGGCCGATCGATTCGTCCACAGTGAGGAGATCGACTTGTAATCGATCACATATGTCGATCGCGCGACCCCTTACTGCGGGTAGCCTTATACTGTTTTGAACTTAACTCATTGATTAAGGTGTAGAGTAGACAATTGGACACATGAAGTAAGTCCCCTGGCTTTTTAAGTGTATGCTTCTGATGCATTTTTAGGTAACGTTTGGTTTGACTCGTGGAATCACGTTTGGTATTGGTTTAGAATTGAGTTGGTATAAGAATTAGTTTATGTTTCCAATTTGTTGTTTGATTTTAAAAACTTGGTTTTAATATCATCATTTACAGCCTCATCTTCAACAATAATTTTTCATCTGCTTATCCTATGTTCTTATTATATACCGTAGTTTGTATTCTTTACTTGACCTACAAAAGAGCTAGCACGCATATCTTGTTCCTTAGGAGATATGGTTAACATGTTGAATCATGTTTGTTTGCCAATATATGTGGTTGTTTTGGGCTTTTACTTATGCACGTTTCTTTTAACTATAGTATGCACATTAGTCTATTATCTAGGGTGTTTAGTGTAATATATTAGTTTTATTTTAGTCAAGTTAGGATAGTTATATTTGGTTAGTTTTCGCCTTGCATGGCAAGCTTATTATAACACCTTACTATCTTGTTTCTTTCTTTAGGATCTTTTTTTTCCCCTCATTGATATGTTTAAGACTCATATCTTCTTTTTCCTATCTCACTAAATTGTTTATTAGCTATGAAAACATCAACAAATTAAGTGTATTTCACAACTTTTATTGTCATTGATATGTTTAAGACTCATATCTTCTTTTTCCTATCTCACTAAATTGTTTATTAGCTATGAAAACATCAACAAATTAAGTGTATTTCACAACTTTTATTGTCATGATATTTGAACGTCAGAATTTTTCTTAGACTATCTTATGAATTCGTGTTTGATATTTAATAGATATGTATAAGTGTATAACGTTATGATCCATCAACGTCTGTTAGCAAGACAAGACACTTTGGTGAGATCTTGCGACCTCGTTCCGATCTTACGATCCCGATCTTGCAAAGATAAAAAAGATCTTGGGTAGAATCTAGGGGCGGAGCCAGAAAGCTTTGCTCAAGAGAGCACATTTTGATAAATTTTAGAAACTACAATACAAATTGATTCTTAGGGCCAATATAAAAAAGCCAAATATTTTAGAAAATTTACACTATAATTTTTTTCGTTAAAGGGGCGCCGATGGTAAGATCTCGATCTTAACATCCCTGCATTTAATTACTTAAGTAACAATTAACGGTTTTAGGAATTTTTTTTAACGACAACTAACAATCTCACAATACAAACCACAAACTATCTCAAATGTCCTAACCATTTTGATTGGGGAGCTTCTCACACAACTAGAGATACCATTGGGGCACAAGCCATTTGGCAAGAATTCTTTAATATTACTATAAAAACTAAAATAAATCCATAATAACGACTTTTAAATTAAAAGTCAATCTTGTATTCGATATTGACACTTCAAATAAAACAAGGGGCGTACTTAGATAACAAGGCATGGGAAGGTAGGAGTCTTGCCATTTAAAAATAATACTAACTAAACAAGCAACAAAGTCCTCATTATCGTTATTTAGAATATATGGTGTGCGTGTTTAGACCTACCAATGCAATCGACTTTGCCCTACCAAACGAAGTAAAAGACATTAAAGGTCCCAAAATTCTTGTATCTGTACAACAACTTGATTTACACGAGCTAAATGAACGAGTCGGTCTCATCATCATTTCCTCGTCTCAATGAGGTGGAGAAGATCATTGGATACGAATTCAAGAACAAAGAGTTATTGAAAGAAGCTTTTACTCATAATTCTTACAAAGATGACAATTGTCGATCTTACGATCGGCTTGAATACCTTGGAGACTCATTTTTGAACCATATATATAATTGCCAGAGAACACTTTTTTCAATATCCAAGGATGAAGTCTGGTGAGTTGACTCAGCTTCGTGCCGCCAATGTTGATACTGAAGCATCGGCTCGTGTTGCATTCAAACATGGCTTACATAGGTTTCTTAGGCACCAAGATCCTCTACTTGAAGGACGGGTATTATTTTATTTTACTTTTTTCATTTTAACTATTCTAACTAACTAAATATGTTTTTGTCTAAAATTGTTGAGTTGCAATTTTGAACTTTGGATGACATCTAAATGAATACACGTTTGTTTTTGTAAAAAGTCTCTTGGGTTGGGTGTAAATCTAATCTCACATTGATAAAAGATAAATTTCTAAGTCAATATATATGTTGGTGGAATACTTATTCTTCTGCCAATTCGTTTTAACATCAATACATAGTCGTGCATATATGTTGGACTTGTGAGATAAGAGATTGTGGCGAGGTCTTATTGATTTTTATTATTTATTTTGTTATTCCTGAATATGAAATCATAGAACATACAACTATGTGTAGGTACAAGAACTTATGGAAGGAATCAAACAGTATCCATTGCACTCTTATGGTTTAATCAATTCACCTAAGATACTTGCGGACATTGTGGAGTCTTTAGTTGGTGCAGTCTATGTCGACACCAATTTATCGGTCGATGCTACATGGGAGGTGAGATCCATACCCTGATCAACAAAATGCGAACATAACAAGTGCACTAACGATTCACATAAACTTTTAATTTTGGGGAATGACTTAATTTTGGATTTATAGGTGGTGAAAAGCTTGTTAAAACCTTTGACATGTTATATTTTTCACAGGTTCCATACTTGATATTTCCAGGATCGATATATGCACAAGATATACAAGAAGATGATTCATTTATAAACAACATAATAGTGGTTACATTGTTACAAAGACACTACTTTCTAAACGTTACATTGTATCCTATATATACTAGTCGAATGGGTATGACCCAAACAACATGTCGGTTCAAGTTGGTTGTTAACCAAACGACACAACCATTCAGAAATAACTAACTTCCTACAATAACTAACTTAACCGCTATTCGGTTATAAACTTTCTATCTAACATGTTTAGTCCTTTGTCTTTCAAGACTTATACAATTATAAGGTCCAACATTTTCCCCCCTAATTGTTTAAGTCTTGTTCTTCGAGTCTTCATACTCTTCTCCGTGTCTCTTTCTTTCCTTCTTCCTGTCTTGCCAACGTGTTGTTGTTTTTCGTTGTTTACTCCTTGTTACTAAACCAATTCGTAAAGAAAAACTCTTGTTCTCCTTTTGATGTTGTCGAATTGTCTCCTTCTCTTCAATAAATCAACGACTTCTTCTTGTTGCTGTATACTTCCACGTTGTCTTTCTCTTTCTCTTGTGTCGACTTGGTACAAGGCGTGTGTGTGCCCTAGACCCTTTTTGTCTCGGCTTCTTTCTCCAACACTGTTTTTGGCCAAAATCAAACTTTGTAGTTTGTTGTTGTTTCGTTCCAAGTTCTTCACAAATACCTCTTGGTATTTATTCTAAAACAAACAAATTGTTTGTTTTCTTTCTACTACTACGACGGTAGTATTCAAACAAGGAGAGCGTTTTTCTTAAATCCTTGTTGTCCTCGTTGCTTGTCCCTTTCCAATGTTGCACCACAATACTTTCTTGTTTCTTTCTTAATTCCACGCCGTATCTTCAACAACTCCTAATACCCGTCGGTATTCTTTTTAAACAAAGCAAGTGATCAGCCTGTTTTGTTATTGTTGTTTTTCCGTATTGCTCTAAACGTCTCTCCAAACAATCCTTGATTGTTTTGTTGTCTCGGTATACCTTATCACATCCACAAGCAACGCCTTTTTCTTGTGTTTATAACGCTTATTCCGTTTGTTTTCTCTTTTGTCAACCGGTTGTCTTAACACAAAGTATCCTTCTTCTTGACGTCGTATCCTTTCTTCTTGTCGCGTTGTATGGCAACAAGTCCCTTCTTCTTTGTTGTCGTGTGCAAGACACCAACAAAGGTATCTAACAATTGTTCTTCTATGTTTTCTCCCACCAATACGTTTGGTGTCAATGTTCGCTTCCTTTTGTGTCGGTTATCAAAACTTATCAAAACTTGCCGCTCCCACAAAAGTTTCCTTCAATCTTCATTGGATTGAAGTCGTTGCTATCTTGAAACTTCCGTTGGTTCTGATCATGCATTATCTTCCCCAACATCCTTGGTGTCTTCCCTTCTCGGTCTCTTAATCTTCTTAATCAAAAAACTTGAATCACAACTTGTAATCTTGCAAACCTTCGGCTCTGATGCCATGTTATATTTTTCACAGGTTCTATACTTGATATTTCCAGGATCGATATATGCACAAGATATACAAGAAGATGATTCATTTATAAACAACATAACAGTGGTTACATTGTTACAAAGACACTACTTTCTAAATGTTACATTGTATCCTATATATACTAGTCGAATGGGTATGACCCAAACAACATGTCGGTTCAAGTTGGTTGTTAACCAAACGACACAACCATTCAGAAATAACTAACTTCTTACAAATAACTAACTTAACCGCTATTCGGTTATAAACTTTCTATCTAACATGTTTAGTCCTTTGTCTTTCAAGACTTATACAATTATAAGGTCCAACATGACAACCCCAGAGAACCTCAAGCTAAACCCCGTGACAAAGTTGAATGAAACGTGCCAAAAGATCGGGATCAAGTTAGAATACAAAAATTTATGGGTCGAGTCGGGGGAGATTGAGATATATAATGGTAATGAGTTAATCGGGAAAGGAAACTACAAGAAAAAGAAAACAACGATGAAGAACAAAGCAGCAGCAGATGCATACGTGAATCTTGTCAAACAATTGGGTCTCAAAGATGATGCACAATTTGATGAATTTTTACACTAAATGTGTCTTGTTTGTTTATCTAAGTGGACTTTGATTGCATTGGAAAACCTTACCTTACAATGATGATTAACCTTTTAAACAAATCGTTTTCTGGTTGATGTGTTGATGTGTACTTATGATTTGATGGTTATATGAAACGAGCTGGGTAGCCCGTGCGTTGCTGCGGGGCGATGTGTTCATGTATAGAAGGCAATTGTTGATGTATAGGTTGATCATTAGTAACATAGAAAAACAATTAATGGCAAGATTTATGTCGATGCTTTATGAGTACATACTAATAAAAGTGATAGGTTTAGTATTTAGGGCTTTGCATTCTGTGTCTACAAGTTTTTATACATGATCATCAATAACATACGTACAACAAATTTCTATAAGTACATACAAATTCAACATAATGCTAAACCTGTAAGATAATTAATACAATGAATAGTTTGTCTAGGAGGCACATCCGAATACCACTAC

AaMyc-bHLH15

TGTTTTAGCTTTGTTTTCTTACAGAAGAATAACAGCTTTATTTTCTAACACACATTTGAACACCTTTTTCTTCTTTATTACTCAGCCAGCCCCTTATATTTTACTAATATAATCCTTGCTTTCTTGTTAGTATTAGCTTCAAACACCCACAAACTGTTTTCTCAGCTTTTTTTGCTTTTTCCAAAGCACTTATTTCAAGTCTTGCAGAATTTGGTCTCAAAATGGATACAGCAACTACACACAAAGATTCAGCTGTTAAGAATGTGCTCAAGAATCTGTGCACCTCTTATGGATGGTCTTATGCTGTTTTTTGGAACTTTGACCAACCAAATTCTATGTATGACTTCTTGACTATGTTCTTGTTCATGTTTTATGTTTGTGGTAATCTTGGTTCAAGATCATGTTTTGAGTGTTTTTCTGCATACCCTTTTGTTTGTTTATTAAGAAAGTGGTTGCTTTATTGGTAAATTGTAAATTTTTATGAAAGTGGGAATCAAGAGACATAATGTGGGTAAATTTAGTAAATCTTTACATTTATGCCACTTTGATTGTTCATATATTCAGTTTTAATTAGTATTTTTTATTTATTTATTAAAGTTTGTATCTTTTTTATGATATGTTTTATGTGGTGAAATTTTGTATTTAGTCTATGCTAAATGTTTTGTTTGTTTGTTTTTTTAGATTGTTGACTATGCAAGATGCTTACTTTGAGGTGGAAATCCAAGGTCTAATTAGTGATTTGCTTCAGCAAACACCTATGCTTGGAGGAGGGTATGTTCATTTTCCTTGATCTTTATATGAATCGATAAGTCGGTTTTTGATCGTAATTTTATAGAGTTAACAAACCTTTTAAGGCTGGTAATGATTAGTAATAGTTGCTTAAAATTTTGAAGGTTAATTGGACAGGCTGCTTTGACTAAGAAGCACATTTGGATGTCCTCTGAAGATAATTACATAGGACAGAGTTCCTCGGGTTCGATCTGGGATATGTTTCAGGTACTAGTTAGTTCGTGTTTTAGCATAACGATAATTTTCTAATGTTTATATGTGAATTTTGATCCAAGTTTGCTTTTCTATGTATGATAAGTCTTGTTGTGTTGAACCGTACCGAAAGTGTAGTAAACATTTGGACCCCGAAATTGATTGTTAATGTCTGCCTGCAAAAGAGAGATCATGTCACTCCACAATTGGTTCTTATTTTTCTACCTTAATTGAACATATTTTTCATTATTTTGCAGGATGATTCTAAATTTTTTCATCAATTTTCATCAGGTGTAAAGGTAACTTTTTTATTTTTATTTTTAAGATTAGCTTAACTTTTGAAATGGTACCACGTTGATGGTACCTCGGGTGCTTGTTTATGTGACAACACGAGCGTATTCTGATGTTTATGTAACTTTCATCTTGCAGACAATTGCAGCAATCCCTGTGGAACCACAAGGAGTGGTACAATTTGGGTCCATTGAGAAGGTTAGTAAGCAAAGTATAATCGAGCTCGATTATCAAAAAAAAAAAAAAAAAATGCAATTTTTATAGGCGTAAAGTGTCAACAACTCAACATACTTTTGTTTAGATCTAATGATTGTTTTTGCTGTACATACAGATTCTCGAGAAGACAGAATTTATACATCAAACAAAAAGAATGTTTGGTGAAATTGTAAATGGAAATGTACCAGCAATTTCATCTTGTACGTCAAACGGATTGTTTGCGTCCTTGATATCATCTGATGATTCCTATTTTGGAGGCGCTGCTCAATATTCAGTTAATCAAACTTCTTTAGATTTGCCACCCCAGTCTTTCGCTTTTGGTTCACAATCACAACCCATGTTTCTTGATAATATTCAGTCTACAGCACATTTACAACCGAACACTGTTCCATGGAGTACTTCTTCTAGTTTGACTTCATTCAACGAACCATTACAGTCGACAAACACTCATCAAGATTTGTTTGACTTACCAATGGATTTTGGAATCTTAGATGAAATGTTTCAAACAGGAGATTTCAACATTTCTCAGTTGTTACCTCAGTCCCCTGGTCAAAGTAATGTCACAGGATTACTTCCCATAAGTTCCAATGAAAAATTCAACTCCTTGACTATTTCTGGTGTAGATGTTGACCTTATCGGAAGTACCGGAGACTTAGGAGATATTGTAACACCAGTCGTAAAGGAAAATCAATTCGGTTTTGATTCGTACAAGTCTCAAAATATTGATAATGACTCTGCTCCGAAAAAAGGCCTGTTCTCGAATCTTGGGATCAAGGAGCTTTTTGAGGGTATTTCTGGTACTTCAAGCTCTTGTATTGAGGATCAAGTGTCGAAAAGAAGGAAAACAGGTAGTTCCTTTTCCGAAATGAGTTCCTTACAGCCTGGTGTATATAACAAAAGTTTTGGGCAGAAGAGCGAGCCCATTCTTAAATCAGAGCCATGGATGGGAAGTGCTTATAGCATGGATGGTTCAAGCACTGTTTTGCAAGCCAAGACGCAAGTCGAGCCTGCAAAGCCCACCAAGAAAAAAGCTAAACCAGGTACTCGACCCCGGCCCAAAGACCGGCAGATGATATTGGATCGTATGGCAGAGTTGAGAGAACTCATCCCCAATGGTGAAAAGGTACTTAAACAACACATAGTATTAGAAATATGTAAACTTTTCATTTAGGTATTTACTAATCGCTTCATTGATTTGATTATACAGATGAGCATTGATTGTTTGTTGGATCGGACAATCAAGCACATGCTTTTCTTGCAAAGTGTAACAAAACATGCTGACAGAATTAAACAGGCTGATGAACCAAAGGTGATCAAAGTTCTTATTCACTGCTTATTAATTTTGAGCGTATTATTATTCAAACAGTCGGTTTTAACATTACTTTAATACTATGTTTTCAGCATAATGGTGTCATTCCGAATAATTTATCCAATGATCCAAACAACAATGGTGTGACATGGGCATGTGAAGTGGGAAATCAGTCGATGATTTGTCCATTAATAGTGGAAGACCTTAGTACCCCTGGACAAATGCTCATAGAGGTAAAGATAAGAATAAAATACTTGTATTTCTGTATTTAGTTACTTTGCATATATCTTAATAACATCAAATATAATTTTGATGCAGATGATCTGTGAAGAACACGGGTTCTTTCTTGAGATAGTAGATATAATCCGTGGTTTTGGCTTGACCATATTAAAGGGAATCATGGAATCTCGTGAGGAAAAAATATGGGCGCGCTTTATAGTTGAAGCTGAGGTAAAGCATTGTGCAACACACTCACAAATTATATATAAAAAAGTGCAAGTCTTTTTAATTGGTTTGATTTCATATTACAGGCAAAAAGGCATGTAACGAGGCATGAGATATTTGCAGCACTTGTTCAACTTCTACAAACAATGGGGTCTAACGATAAACATCTTGAAAAAAAGATTATGCAAACGGGGAACTCTTTTCATAACGGTTTCCAGCATTCTGGGATACAGCTTCCTGTAAGCTTGGCGGACACAGTGTATGGCATGAACTAATTATAACCTTGTTTTTTTGAAGATTACCATGAGTATATTCACCTAGTACCGAATATATGTGATTACCATGATTGGTATTCTGGGTAGAAAAGATCCAATTTCCTCCAAACGGGTGATGATTTTTGCCAAAGTTAGAAAACGAAGCTCTGGTTTCTGATTGCAAGGAAGACGACTAATGCTTTAGGCTCTCATAGGCCGTATTTT

AaMyc-bHLH16

ATACCCATAATTATTTTTACTTATATTCCAAGATTATTATATCACATGTAAGAATTTTGTTCATTTTTCAATCATTAATTATAAGGTTATAATACGATTGAGTAAGCCTTAAATTCTTTTTGTCGATATGTAATTAATTATTTATAACCAAAGCCGGGATGGCAAAAATAAACATTACTACAATTTTCTTCAGAACTTCGCGGTCTTATTCCTTGCAAAGCATCTTGACCTTTGTGGGATCTAATATAAGTCCTAAATAAACCAGCATATTTTTTTTTTTGAAAAGCAAAATATTATATCAAAACCCAACAAAAAGAACTACACGAGACCTGTGACAAAAACACAGGAGACTAAACACCACTAGACATTCCTCACCAACATAACAAACAAAAAAGAAACCACCATATGAGCTATATGGTGTAAGAAAAATGCTAATGGTCCATCAAGACTACTAAAACTCCCCCACCATCAGCAGCCAAACAGAAAATTTGTACGCGCGGCACACATAAAAATCACACACCATCAGCATAAACCAGCATATATTATCATTAGTGAATTTATGCTATGGTTTGGGTATATATAGGGGAAGATGGAGGTGTTGCAAGAAATGATGGATAGGCTAAGATCGATTGTCGGTCCAGAGAGTTGGGATTACTGTGTGCTTTGGAAACCATGTAAAGAGCAAAGGTACATTAACCATGTTACCGAATTTCTATTAATCTGTGAGACATACATAAACGTAAGCTAAAAGCAATGAGTCTGGTAACATGTAACTGTAGGGTGATTGAATGGATAGATTGCTGTTGTTCTGGGAGCAATGCTGCAAGGCATGGGATTGGAAATGATGATCAACAACAACAGTTGGTGTTCCAATGCAAAGATGTCGCATTTCATCACCCAACTACTGATACTTGCAACCTCCTTTCACTTTTACCTTCGTCGATGCCTTTTGACTCTGGGTTAGTGGGGTTACCCTTTGATTCTTATTGAATTAACAGACACAATATTGTGATATATTACATGATACAAAATATGCATGTATCTTTACAGCAATTGCAAGGCTTTTACGTGTTTTTATTACAAAGTTAGTTCAAAAGTATAAAGAAAATTTCGCTCTAATCATACTTATTACATGTGATACAAAATCCAGTTTCTAAGATACAGAAATAGAAACGATAATATTTAAAGATTATGTAGAAATGACTGAAATCCTAATCATTTTGCTACTACTTCTAGCCTAGATGTAGAAACCAGTACTCTTACAAGTCCTACCCGTTCGTTCTAAGTGTCTAAATATATTCAAAATAAAATGCACAACCATGTTATTTATTTTGACACATCAAGCAGGGTTGCATACCTTTCCTATTCTGTAGACCACATAGCATTCTTTTGATGAATAATATGTGACATTGTATTGTGTGGTTGATGATATTTCAATCTTGTTTTTCTTAAATGTACTAATGTTAATTTGATCTGCAAGTTTTCTTTACGTATATTCAAAATCAAGAAGCTTTGATACGATATTTTATTCGTTAGTATTTATACATATTCTTCAGACTCTATGGACAAACAATGATATCTAATCAACCAAGGTGGCTAAACTTTTCTAATAGTTCCAATTCTGGGTATTCTGAGGTAAACTCTAATTACAAACATTTTTTTTATATGTAACCTACATATTCCACAACAGCATTGTTACTCAATTCTTTTTGCAGGAAAATCTTGGGACTAAAGTCCTTATTCCTGTTCCAATTGGACTCGTGGAACTCTTCGTATCTAAACAAGTAACATTTCTGTTTAAAAAACACATCACAGGAATGAAAACTATTTTTCTTGCCTTTAATTATCTGAGAAAAGGAAAAATGAATTCAATGTTCTATATTGATCTTAACTTTTTCTTTGAATGTTTGTTTCTTTGCAGATATCTGAAGATCAAAGCATCATCGATTTTGTGACCACTATGTTTAACATGTCACTGGAACAACCTATGTTGAACCCAAACAATAATATCGACTCGAGTTTCTCTGTTAACATGGATAGTCTGGACGATGGGGAGTCAAAAGATTACATAGCTCAAGTTCTCGATGATCAAAAGGATCAAAATAACCATTTCCAACCACCGATTTCTCCTGCTACCATGCTAGAAAACCTTAACCTCTCACCAAACAACATCTCTGACAACCATATGCATCCTATGAACTTCTTGCAGCAATTTAACTACGGGGAAAGCAGAAATACAAATAACATCTTCATGGAAGGCACAAGCGAGCCTATGATGAACCATGATGGCCCTTTTGACCCTAATTCTGAAGATAATGTTGGATTTGACCATGAGATTGATATGGCGTTGCAAGGGCAAATGATGAGCGAAAATATGGGCAAGGCTCATCTCATGGAGCCGTTAGAGAACACTCCAAAGAAGCAGGGAAATGACATGAACCGGTCAGATTCTGTATCTGACTGTAGTGATCAAAATGATGAGGATGATGACCCTAAATGTCGAAGGAAGAATGGCAAGGGACAGTCCAAAAACCTCATGGCGGAGCGCAAAAGACGAAAGAAGCTTAATGATCGGCTCTATACTCTGCGCTCTCTAGTACCCAAAATCACTAAGGTCTGCTAAGTGCTAACAATCTGTCTTAGTTCAGTTAGATTTTACAGTTAAAAGTTGTTCAAAATGCGCTCCTAACTTATCATTCATTTTTTTCCCTTTTCACAGTTGGATAGAGCCTCAATTCTCAAGGATGCCATCGAGTATGTAATGGAGTTGAAAAGGCAAGTGGAAGATCTCCAAAATGAGTTGGAAGAAAATTCGGATGATGAGGGCACTACAAACAACCAAAGCACCATCGTTGAACAAGAAGTTATCCACGGCAATGGAAGTAACTCAAAGCGTAGATACAACCACGGCCCTGGCCTGTTCGTGAATGGACCTCAACTGGAAGCGTATTCGGGTGTTGGTAACATTGAAGTCTCAAAACAAAACCAAGATGTCGAAGGTGCTAGCGAGAAAGGCCAACAAATGGAGGTAATACACGTGTTCCTGGCTATCTTTATCAATTTGAAAACTTTTTTGTATGCCTGATTTCTCCTTGTATGTGTAGCCGCAAGTGGAAGTGGCATCGTTAGATGGAAATGAATTCTTCGTGAAGGTATTCAGTGAGCACAAACCAGGAGGATTTGTGAGATTGATGGAGGCCTTTAATGCTCTAGGACTTGAAGTAACAAATGTCAATGTTACTAGTTTCAGATGTTTAGTTTTGAATGTCTTCAAAGTTGAGGTGGGAACAAAAAATTCATACTACTCGTCTCTTTTTTTAAAGCCATGTGAAATATTTGATTTCAGTCACATATAATGAAAGGAAAATTATCTCAAAAGAAAAGGTGAAACGAATGACCAAATTACTGACCACACTGTGTTATGTGTAACAGAGGAAGGATAGTGAAATGGTGCAGGCTGATCACGTAAGGGAGTCGCTGCTCGAGATAACACGAAACCCATATAAGGGTTGGCCGGAAAACTCCATGAAAGCACAAGAAAATGGTCATGGCATGGTGGATCACCACCACCAAAACCAACACCATCACAATCAGAGTCACCACCACAATCACAATCTCCAGAACCAACAACCAAAACCTCAGTACCACTACAACACCATTTATCAGGTCCTTAACTAAGCTAAGGTGTTCATAAAAGTTAAACAAGATTGAGGACCACCGAACTACTTCATTTATCTTCTTAGCATCTATGTTTTAGCTTTTATATAATACAAATGATCTGTCTGTAAGAACAGCCTGTTGTCTTCGTGAGTTTGTCTTAATCAATAATTTGCACTTTCTGCAGTTACTTTCATTACATTCTCTAGTTCTTTACAAACTAATACCGTACTTGATAAATATATGTTAACAGAAATGAGTATAAACAAAGTCTACCATTTTCTGTATGAATAACCTGACGGTATAATACGAAGACCCATAAATTAATAATGTCACATAGGAAACAAATTACACTTAAACTGAGTCGAGATTTCCCCTTTGCAACTACTATCATGTGCCTAGTTTCCCTAGTAGATTTTGCATAACAGTTGGATATAAAAAAATAAAAAATAAATAAATAAATAAAACACTTTTGCATAAATGTAAACAGTTTACTTCCGCTGGTCACTCATTGGTGCAAAATTTTCTTCATCACAAAAAACCATAGAATTGTATACACGATTTTCAACAATTATAGTTCCCACGTTGCTGCATAACAACCATCATTATTACGGGAGCATAAAACTGAAAATTAAACTTTCATGTGAAAAAAATTATATATACTTTATTTGCGAATTAAAACCAACACGGCTTCTTTGTTTTAATCATTCAATACATTACAATATGGTATTATACTGTTCTAAACGACTTCTTGCTCAGATATCATAACATGCTACAATGGCATATCCTATACTAAGTGAGAAATAGCTTGGAAGAAGAAAGCTGCATCTTTAAGGTGTGTTCACCTCTGTTTTAGGGCATAAAAATGATATACTCGTATTATGTAACTATGTGTTAGTTTTTATCTAGTTTCCTTAGTTCTACCATGATGTAACAATATAACTTTAGGAGTGAACAGATTAAATTTATTAAGTACAGCCGGTTTAACTACTTTTGATGGAGGCAAAAAGTTACAGCAGCTAAGAACAGAACGACAGGCATATAGACCTTGAGCCATAGAGACAAACACTATACAACATGTGAACTCTCTCACAAATGATTAAATGGGGACGAAAGGTTATCTTAGCTCTGCAGAACGAAAAGAACGTCACCATCCCTTTGTATCAGATGGGATCTCAGTATAGCCAAGACATATCTAACTGGAACAGTAAGCCTAGAAATGAAATCAATACACAGTTCTTTTCACTATCTCATCATGAGTATCCATAGCAAAAAAATAACATTACAGCTAGATATTATCAACTACCAGAATCATATCATTCACCTAATGCTGACATCCTAATAAAGTATAAACAAAGTATTACAGTTTTGAAGGACTGCGCAGTTTGGATTACAAGATACATTCAAACTAAACCTAGTATGAATTGCAAATAAAAAACTACAACCTTAACACAAACTACAAGCTGATAAAAATCAGCCATTATGATACATGTAACAATCCAATACACTCCCCGGATAAAATAAACAGCAAGTAACCTTTTTTATTACCAAGTCTATGCATTTGTACACAAAACGCTAAACCTCAAAACCAAATTCGGCGAAACACGATTAGTCCTTATTAATCAACTCAGAAAAGGTCAACATTTCATTTCATTTCATTTTCTAACTTATACGAAACAAACAGTAAAAAAACAAAACAAAATCTCAAAGAGCATTCTTAATAATCTGTCTCCTAGCCAAAAACCTAGCAATAGAAGGCGTAACCGCAATCGTAATAGGCACCCGAACAGGAAACAAAGCCTTATTACACAAAACAGCCAATGCAAGCAGTGGCGTAGCCAGGATTTCACTTAAGGGTAGGCCGAGATGTACCACAAAACAATAATACATCCAATCATCATTTGTATAAGATAGATGGTAAATAAATTAGCTGACAGTTATAATATGAATTTGCCTTTGGTTGAGCCAAAATGACATTAATTTGTCTAACTTATAATGGGCTAATACAAATACTAGTAAAATATACATTATCACAAATCTTTATGGGTGGGCCATGGCCCACCAAGGAACCCATGTAGCTACGCCCCTGCCCTAGCACCTCCCATCTTATTCCAGCCGCCACCACCACTGGTGCCACCCTCCGGCCACCAGTCAACATCGTCACCACCACCACCTTATCACTCTTTTTCTCCCACAAAACATCACCACACAACCTTGTATCACCTCTTGGTGAACCGTACGCGCATCCACGCGCCACCACCTCGTTATTTTCCATCCGGTTTTGGTTTAACAAGTCCAATGCAACTTGTTAGGCAAAAGTACTTTTGGAATCATTCAATGGTCTTCGGAATAAGGTAAGGATTTTTTTTTCGGACCATCTTGCTCAAAATGTGGAGACTTTTCTGAAAATTCTTTGGGCTTCAGTATAGTGTCTCATGACATTTTCTTCGGCCTTTAAGGAATATACATTTGTCTTGGGCCTTTTCTTAAAGTAACATCATCCATTTACTCGCTTAAGACTCTCTTTCACATTCTCAGTTGCTCATTTTATGCTTTTGTTGACTTTTTATGTCTTTATGCAAACCCTTCTCCGCCCGCATCACAAACACAAACAGATCACGATATCTTTGCGTTTACGTTAGTTTGTCTTTAAGTCTAAAATGGTGAATAAAAGGTTTGAATAAAGCACATACCAAAATCCCCAGACTAACATTCTTTCCTCACGCAATGTAATCACATCATACGCTTTAAAGCATAATTAATTTCACACTCTCTTTCCTAACTTTGAAAAGTAGCATAAACGTTCAAAGAATACAAAATATTTATTCCCTTAAACGCCCCGGTTTTCCATTTTGACTATGATACATCATATATTACACTCTTTTAGCTATGACGAATGTGCTTGACTTTCATTTATCTTTATATATCGATTAAGGTATTCAATAGATAAAGAACACTAGTTATAGAAAAGTGATCGGATTTGGGTATCATCAGTCCCTGATTTTCACAAACCCCCAAACTAACGTAAGAACACCATCATTTGTTTCATTACATAATTAGATTCTAATACGCTACTCATGAAAGTTACTCGGTACTCTTTGCTCCATTTCCTATTTTGATGTATCTCATTATTTTCATTTTTGTGAAGAGAAAATAATTTCTTCTAAACATCTCGTTTCAAGGTATTTAGTTGCCGATAAAGAAATCTCTCTTTTTTTTNNNNNNNCGCTTTTTAATCTTTTGAAGACCTTTGGAAGAAACGTCAACTCGAGTCCTCAAGATTACATTAGTGTTACGTTTAATGTTTAAGAACGTTTAGTAGTTTGTAACATAGTTTTAGTGCAATATCATGAATTGTAAAGGTTCAAGGGCTAAACGTGCAACATCGCCAAACATGTGCAAGCTCTTGACCTATAAATAGAAGCTTAAGCCTCATTGTGAAGGTTGGTTAGTTTTTAGGTTAGATTTAGGAGTAGTGTAGATTTTAATTTTGGTAATGAAGTTTTATTAAACCTTTATTTGCTTTCTCTCTACTCTCTCTCTAGATTTCTTGTTCAATCTTTACAAGTTTTGAGTCTTTGCTAATCTTTATTTACGTTTATCCTAATCTTTTATAAAAGCTTTTATGTATTTCATCAACTTTGCATGTCTTTCTTTTCGTATGGCTTAAATAAAATGGGTTAGGGCTTGATTTTGTGTCGTTGGCTTATATAAGCTTATTTAGCTTCTTTTAAACTAGTTTTTCATTAAGCTTTTATTAGATTTTATTTGTTTTGATCTTTAGTTTCACGTTAAAATCTGTTTCACAGCTTAATTTTTATGATTTTTGCGAGTATCATTTAGGGTTTAGCGAGTTTGGATGTTGATTTGCGAGTATGGAAATTTTTACAAGTTCTTAGCGAGTATGGAGCACTATTTAGCGAGTATGGGGTCCTAATTAGCGAGTATCACATGGAATTTAGCGAGTAAGAATTAGTTAGCGAGTATGGTTAGAGACATCTAGCGAGTATGGCATGAATCCCTAGCGAGTATGGTTCATTTTCATAGTCTTGCGAGTATGGGACGAATATTTAGCGAGTATGGTACTAGCAGTAGCGAGTATGAGATCTTTGTTAGCAAGTATGGTTGGTTCATTCCCTTTCAAGTTTTATTTCTTCCTTTTGCCGAGTTAGGCACGAGTTCCCCTCATCGTTTCATCGAGTGAGGTTTTATTTTTATTAAATCTAAACGTTATGTTGTCGAAATTTTCTTAAATCTTTTTAGTCACGTTTTCTTATTCAAGTCTTATGTCTTTTCGTTTTTAGTTTTAACCCTTATTTACGTTTAATGATTTCAAGTCTTTTATGTTTCTTTCTTCGTGTAGTAAACTTTTATTTTCAAAAGCATTTTAATTTTCAAGTTCATTTCATTTTCCAAGTCAACTTTTATTTTCTTTACCGCTTTATGTTATTTAATATGAGTCGCGAGTAGTTTAATTCGTGTTTGCTTAGTGACTCGGGTTTGCGAGCTAAGTGTGAGCATTAAAGGTTTGTGACTTATAATCGTTTAGATTATTTAGATACGTGATTTAATTGTTTGAAGAACAAAAGCCGGAGATCAATTAAATCACACGTCTAGAGTTACTATCTTTTCAAAAGATTAATTGACAACTAATCTAGAGAAAGGAAAATAACTCGTCATAAACCCTTGGCTAATACTTAGTGAGTTTATTCACGTTACTTTGCAAACATTTTGTCTTTAAAATTCATTTACGTTTTTAATTACTTTTCGCACTTTAATTCTCGCTTTCTTTAAGTCACTTTATTACATGTTTCTTTACTTGCTTACTTCCTTTATGTCGTTTTACTGCTTTCCTTTACTTTAAACCGCATTTTAATTGTCGTTGTAGTGTAATCAAAACCAAAAATCATCTTTTAAACCTATTCATCTCACATATCTCACGATCTTGAGTGACGAGATCAGTTCAACCCTCGTTGGGACTACGACTCGAGGTTATAAGCTTCACCGCTTAATTCTGCATCGATCAAATTTTTGGCGCCGCTGCCGGGGAGCGGTTGATAAGATTAAGTCGTTCGATCGTTTGAGTAATGTGTTTTGAAGATCCTTCTTTCGAGAAGTTAACGCTTTTGTAAATATTTTCAAAATATAAAATCACAAAAACATTTTCTCTTCAAATTTGTTTTCACTTTTGGCTTCCACTTTCCTAAAAACAAAAATCCAAAAGGATTTTATGTTTCAAAATTGGTTTTCACTTTTAACTTTTCATTTTCAAAACAATAAAAACCAAAAATAATCTCCTTATCTTCATATCATTTAATGGCGGATACGCGCGTTTTGGAGGAATTGCTGCAAAGACCTCGATATTGTCCGTGCGAACGGTGTGGTAACAACCTTCAGAATGGAAGTTGTTTGTTTTGTGGTTCAAATAATTCATCCTTTTATGATCAAAACTCGAATACATTCAACAACTTTTCAAATTTTTTTGACTACCCACCTCGGCCCTACTTATCTTCGTTCGATCAATCTTTCTGTAATGGATGTGGAAATCGTTTGGATCCGTCTTCGGCGTATTGTCAACGGTGTAATTGCATGAGGTGTGGTAGCAATCTTATAAATGTATTTTGTTCGATGTGTGCTTTTGTGTTTGGAAATTCATACTCTCAAGACTGTAATTCGAACTTTTCAAATTATTCTCCAAATGATTCTTACCTCCCTCCTCAAAACCCTTATGAGCAAGAACCGTATGTCAGTAACTTCTCATTCCAAAATACTCCGAATTTTGAGAATTATGGGCTATCTGTTGATAATATTCATTCTGAGCCGTATCTAACATTGAAAGAATCAAATGAATCACTTCTCAGTCTAATGAAGGAGTGTAAAGAGATAATTCTTCGAAGAAACAAGGAAAAGGAAAATTCTGAAGAGAGCAGAGAAGACGAGACTTTGAAAGAAGAGAACGAAATANATACTCTCAAGACTGTAATTCGAACTTTTCAAATTATTCTCCAAATGATTCTTACCTCCCTCCTCAAAACCCTTATGAGCAAGAACCGTATGTCAGTAACTTCTCATTCCAAAATACTCCGAATTTTGAGAATTATGGGCTATCTGTTGATAATATTCATTTTGAGCCATATCTAACATTGAAAGAATCAAATGAATCACTTCTCAGTCTAATGAAGGAGTGTAAAGAGATAATTCTTCGAAGAAACAAAGAAAAGGAAAATTCTGAAGAGAGCAGAGAAGACGAGACTTTGAAAGAAGAGAACGAAATAAATTCTTCAAGTGGCAATCCCACTTCATCTTGTGAACCTTCAAATGTTGAAAATTTTTCTTTGGCATCTCTCGACGACGACTTCTCTAAAATTCTAGCCGCTGTAGAGCTTGCGAGTGCTCGTGTTATTTATAATGAACCATATACTCCTGCTTCTTTTCCGGTGATGGAAATGGAACGAGAAACCGAGGTGATAAAGGACCCGGTGCTTTCCCTTGACAATGGAAGTGCTAAATACGCCCAACCTTCAGTTGTTCAAGAATCAGTTGTTCCCCCAAAACCGTCAAGAATTGATCCTAATCTTTCTCGATTTATGGTTGAGAGTTCGTCAAGTTTACGTGTCAAGCTTAAAACGGTGACTGAAAAGAACGTCTTGCCTTATCTTCACGTCCCTCAAGAACAAGAACCAGAGACTACAACGGAAGTGGTGGAAACTGCTAGCTCTCAGAGCACACCACTTGTCCCACCTCCGGCTTCTCCAAAATGTTCTTCGACACTTGTTTCCGATATTTCTGAGCCGTCAGATCCTTTGGAGCCCGAGCTCAAGGAGTCTTTACCCGATTTTGAAAAGTATACTTTTGATACGGCCGAAAATTGTGATGTACCTCCTTTTCCTCCCGTAGAGCCCGAGGACTCTCTAACAATGGGGGACGAGCCTCTTAACACTATTCCGGCAACGGAATCGGACGAATTCAATAAGTCTAGTGTTGAGAACCTTGTCCCAATCCAAAGTGAGTCCGGGGAAACTTCTAACGGTGATGGTGAAATTGTTGAAAGTGATCGTCTTGATAATCTTGTTGATACGAATGATAAGCCGTTTGATGATATTTGCTTTGAGAAGAACGATGATGAGATGCTCTCCGATTTTGATTCTGACGATTCTTTATCTTGTGGAGATATCGAATATGTTGAGGAAGTACATTCTGAGCTCGTCAGCTTAGAAGAAGAGAATGATGAAATCCAAGATGAAGTTCTTCGTGAGAAATTATCTAACGTTTATCTTCTCATTTCTAAGATCGAGGCATTGAATAAACCTCCGACTTCTTCTCCTCTCCCCGTTTTGGATAGTGATTTTCTACCCGAGCTTGAAACTTTTCGATTTGAAGAGACGAGTAGTGGCAATCCCATTACTCATGCTGATAACTCTCTTCCGGATTACGAGTGCTTTCATTTTGAGATCAACTCTTTACCTATTACCGATAATCCGTCTCGTAACCCTCTTTTAGAGGCTGTCGATCGATTTCTTGCTATGGATGACTCAATACCGCAGGGTATTGAGAATGACGAGAATGATGTGCTTATTAATGATGATACTTTGAATTCCAATTCCAACTCAAATCTTTTTGATTCTCGTCCACCCGCGAAACCACCCGATGTTGATTTTGGATCCGGTGTGAATGAAGTTATTGATGGAGGGGTGGATGAGACTTATGTTTATGCTTTCACCATTCGTGTGTTTCACCCATTCTTTACTTATCCCATCATTTCTTCACTTTCCCACTCCTCCGCGAGTGAGGATGTTGTTTTTGATCCCGGCATCGTCGCTTTTCAAGCTGTGTGTTTCATTTGCTCCCCCAAGGACAAGTAAATTCGGGGCGAGTCTAAGCTATACGACTCGTTAATAAACAAGCGCTTCGTGGGAGGCACCCCATGCTTATCCTTTTTATTTTATTATCTAATAAGTAACGTGAGGAGGGCGACCTCTTCCAATCTTGTGTTTGCTTGGTTGTTTGTGTTTGTGCAGGATTTCCCCAATTCATGATGACTCTCGTGCTCGTTGGATCGTCTCATCTCCACTCGAGTCCCATGTATTGATGCTTGCTTGGAAATCATATATCCAAAATCTTATCGACTAATGGTATCTTTGGCATACTTAATAAACGGCCTTAGATTTATTGTTAGGATTACCCTTACCTTTTTAAACATTGAGGACAATGTTTATCTTTAGTCTGGGGGGGGGGGTGAATCTTCGATTTGATTGACCTAGCACTTTCACCATTGCTGTTTACTTACGCAACCCCGTTTTTCCTCAAAAAAAAATCACAAAAATTGAAAAATTTTGAAAAATTTCAAAGAAAAAAAATGAGAAAAAAATTAAAAATTATGCAAAAATAGAATAAGTGATTTGACACCGTGTTTGACTTTCCTACTAGGTAAGAGTGAGAATAAAAATTTTGACTTGATCCGGGCGTGTTGAGCCCTAGCTTGTCTCCCTTAACCAATGTAATCTTTAAAAATGATTATCGGCTAAGCATTCGCGGATCTCATTATTTGAGATTTTAAATTTGCGGAGAACGTGAAATGATAGAGTTTCTTTAGAAGTTAAATGCTAAGCATAGAGTAGTTGGATCTCTTTAAAACTCATCGAAGGTACTCATGACTTTTTGTTCCCACAAAATTGCTTCACCCCATATCTGTGCTAAATAATTAAAGATATTAGGTTTAGCCGATGAGTAGTTCTTTATCTTCTTGAGAGAATCTTTGCTTGAAAAATTGGGTACACATCAAGGATACACCCCATTATCTCGAGTTGTATTGTATTGTATCTCGTTGTATAAGCGTAGTACGCATGTGGTTTCGGAGCTACCCTAAAGCCCGTAAAGTAAAAAATAATAATAATAATAATAATAATAATAATAAGGATGTATGTTGAAGATTTATGGATTATGAGTTTAAGTGTTAAGACTAAAGTTGGTAGCACGTGAGGTGCATGCGAATGATCCTAGATCGGGCGCATGAGGTGCACGTGAAGGTACTGTCCAACTTATGTCTATTTTGATGTATTCCAAAGTCTTTGTATGTATCCAATATCCTTGATGTGGACCACATTTCTCTTGCAAACCTTCTTAAAAGAAGATCGATAACTACTTAGCGTATGAATTTGGTATCTTTATCGGACTAAATACTATGAAACGAGATGTTTATGAAGAAATTCATTTCTCTCACAAAAATAAAATAATGAGATACAAAAATAGGAAAATGGGAGCAAGAGGTACCCGAGTAACTTTCATGAGTAGCGTTAGCATCTAATTTGTAATTAAACAATGATCCGTTTTTACGCTTAGTTTGGGGGTTTGTGAAAAATCAGGGACAATGAATTTTATCCAATCGGTCACTTTTCTATGAATCGTGTTCTTACTCTATGATCCTTAATCGTTATTGAAAGATAATGAAAGTCAAGCACATCGTCATAAGCTAAAAGTAGTGTAATATATGATGTACATAGTCAAAAATGGAAAACCGGGGCGTTTTAGGGAATAAATGATTTTGTATCTCTTTTGAACGTTTTGCTACTTTTCAAAGTTAGTAAAGAGAGGTGTGAAATTATTTATCTTTAAAGCGTGATGTGATTACATTGCGTGAGGACACGCAATACCTTAGTCTGGGGGATTTTGGTATGTGCTTATTCACATACCTTTTATTCACCATTTTCAGACCTTAAAAGACCAAAAACTAACGTTAAACGCCAAAGATATCGTGTTTTTGTGTTTGTGATCGTGCAGGAAAGGATATTGCATAAACGACATAAAAGTCAACCAAAAAGCCTAAAATGAGCACGAGATGTGAAAGAGAGTCTTAAAGCCGAGTCAATGATGATATCTACTTAAGAAAAGCCCAAGACAAATGTATTCCTTAAAGGCTCGAAGAAAAATTCATGAGACACTTAACTGAAGCCCAAAAGAGAATTTCAAGAAAAGTCCTCCACATTTGAGCAAGATGTCGCTTTTAAATCTTTTGAAGACCTTTGGAAGAAACGTCAACTCGAGTCCTCAAGATTACATTAGTGTTACGTTTAATGTTTAAGAACGTTTAGTAGTTTGTAACATAGTTTTAGTGCAATATCATGAATTGTAAAGGTTCAAGGGCTAAACGTGCAACATTGTCAAACATGTGCAAGCTCTTGACCTATAAATAGAAGCTTAAACCTCATTGTGAAGGTTGGTTAGTTTTTAGGTTAGATTTAGGAGTAGTGTAGATTTTAATTTTGGTAATGAAGTTTTATTAAACCTTTATTTGCTTTCTCTCTCTACTCTCTCTCTAGATTTCTTGTTTAATCTTTACAAGTTTTGAGTCTTTGCTAATCTTTATTTACGTTTATCCTAATCTTTTATAAAAGCTTTTATGTATTTCATCAACTTTGCATGTCTTTCTTTTCGTATGGTTCGAATGGTATAATAATGGGTTAGAGCTTCTTTTCGGACGTTAGCTTATTTACGAGCTTATTTGTCACTTAAACTAGTTTTCTACCAAGCTTTTATAAGTTTTTATAAGTTTTGATTTCATTCTCTTGAGTTAAAAAGTGTCCAGTACGTTATCCTTATTTCTAGCGAGTTTGGATTTTAGGGTTTAGAGAGTATGGGATTTTTCTAGATTTCTAGCGAGTATGAAATGTTGTCTAGCGAGTATGAAGTTCTTAATAGCGAGTATGAATGTTTTGTTTAGCGAGTTTCAGACCATTCTAGCGAGTATGGAGATTTTTATAGCGAGTATGGTTACTTCTTTGTTCTTAGCGAGTATGGTCTTTCAAACCTAGCGAGTATGAAGTTTTTTTAGCGAGTTTCATTTTAAGTTCTAGCGAGTATCACAAAGTTTCAGCGAGTATGGATTATCTTTTTGTTCTAGCGAGTATGGTTTTCATATTTAGCGAGTATGGACTTCTAGTAGCGAGTATGGTTGTGTTCCTTTTTTATTTTATGCCGAGTTAGGCATACTTTCACCTCATCGTTAATCGAGTGAGGTTTTATTTCTTTAAAACCTAAACGAAACTCTGTCGAATTTTCTTAAATCTTTTTAGTAACGTTTTCTTACTTAAGTTCTATGTCTTTTCATTTCTAGATTAAAACTTTATTAGAGTTTAATGTTTTCAAGTCTTTTATATTTCTTTCTTCGCTTAGTAAACTTTTATTTTCAAAAACATTTTACTTTCCAAGTTCATTTTATTTTCCAAGTTAACTTTTATTTCTTTATCGCTTTACTTTCTTTAAAATGAGTCGTGAGTAGTTTAATTCGTGTTTGCTTAGTGATTCGGGTTTGTGAGCTAAGTGTGGACGTTAAAAGTTTGTGATTAATAATCGTTAAGATTATTTAGACTTGTGATTTTATTATTCGAAGAACAAAAGCCGGAGAACAATTAAATCACTCGTCTAGAGTTATTACCTCTTCAAAAGATTAATTGACAACTAATCTCGAGAAAGGAAAATAATTCGTCACAAACCTTTAGCTCATATTTAGTGAACTTATTCAAGTCGCTTTGCAAACATATTGTCTTTAAATTCATTTACCGTTTTAATTACTTATCGCACTTTAATTCTTGTTTTCTTTAAGTCACTTTATTACATGCAACTTTACTTGTTTATTTACTTCGCGTCACTTTATTGCTTTCCTTTACTTTAACCGCATTTCAATTGTCGTTGTAGTATAATCAAAACCAAAAACCATCTTTTAAACCTATTCATCTCACATATCTCACGATCTTGAGTGACGAGATCAGTTCAACCCTCGTTGGGACTACGACTCGAGGTTATAAGCTTCACCGCTTAATTCTGCATCGATCAGACGAAAACGGAGAAAAACAGACAAAAACGGATAAAAACCGTCGAAAATATATAAAAATGGACGTAAAAAGATTGGGGGGTATTTTAAAGGAAAATAATTGGAAACAAAAAAAAAAGTGTTTTCTAGTTTTGTTTATTAAACTTGGAAATAGGAATTTTGTGTTTTGTAGAGAATTTTATTGGAAAACACTAATTTGAACGTGATCTCTGTTTTCCTTGTTTTCCACAAAAAAAACATTTTGTGGAAAACAAGGGTGTTTTCCCTCAACTAAACGCACCCTAACTATTTTCTCGAGAATTGCCTTCTTTGTATTTTGATTTCCACTTCATTTGGTGTGTAAATGAGTTTTCTTCACAAAATAATTCTCCGCCTTTTTATTTGAGAATTTGACAATAACTAAAAAGAGAGGGGTATAAAATGATTATCACATGCAAGTCACGTTATTGTGAAAGATAATTGAAGCCCAACCCTGGCAACTCATCTTCTCCACCCTATGATCTTTGTTTCTTTTCACATCCATCAGTAATTCTTCAACAATCGTTGCTCCACCACCAAGATATTCAGGTAATTCATATGTAAAAATCATTCTCGTCGGATCTGTAAATTTTATTTTGTTAATTAATTAGGGTGGTGGCAATCATGCCTCCTAAAAAACCCCATACCTCCCAACCCACGAATAATATCATTCCACCTCATTCATACTTCTTTTATACCTCTCAACCCTTTTTAGTGGCAACCATACTTTTTTCATACCTTCCAACCCACCAACTAATTTTATTTTTTTATTTTTTATTTTCCTTTCTTATTTCACCATACTTTTTTCACAATAAAATTTATATGACCATACTTTTTTCACAATAAAGACGCAACTTTACACATCCTTTCTTATTTCACCAAATAAATTATAAGCAGCACACTACCTACCTTATATCTCCTTCCTCCTAAAACAAAACATAGCCGACCTTATATCTCCTCGAAGCAGTCATCAAACCTTCCTTATCTCTCCATTTCATCCTACCTTATCACTCCTTCAAAAAGACCTTCCTTATATCTCCAATATCAAACAAACTAAATTCTTTTTTGTCACCCAAACCCATGGCAACAAACTAAACTCGAGCAGCAAGCCAGCAAACACACGTTTATTTTTAAAATAAGAAAAGCGTAGGTAGTGAGTAGATTGATGTTGATTTTTAAGCCATGGGGTTCAAGTAAGGAAGCTTGCCCAACGTTCCTTTTTTGCTCCCAACGGTCCATTTTTTCCAACGTTCGAATTGATAATTGCCCATCATCTCCACCCCGTACCATGCCGCTGAAGCATCAAATGCCGCCCAGCCATGCCGCCGAGCCATACTGCCCATGTGGCGGTGGTGTTCACCGCCGGTATGGCCCATACGGCTGGCCATACCGCTGCCATTGCCACCACCCTTAGGGGTCATAGGATATTCTTTTTTGAAATTGGGCCAAGTAAAATCATTACTTTTTGTGTAAAATTGGACTAATAGAATCTTTACACATACCTGAAACAAATTACACTCTTTAATTTTATTAACAGTTTATATATTTGTGTGCAAATTCTGGGCATCAGATAATGAAATTTTCATGTGGTTGATGGGTTTTTTGAAAAGTCAATGCAATGTATTTTGGGTTTGATGGTCCATTTTTTGATTATTAGAACTAACTTGTACATATTGATTTGTTCTATACTTACTGATATATGTATTTTGGGTTATCAGCAGACCAATGTTTCAATAAACTTTGCTATTGTACTTTGTATACATTATTGGTATAGATTGCGTTGCTTTTTCTATATACAAACAACCTACTTATACATTGTTATGTACCATATATATGTAGATGACGAGATCACCATTTGCCCAACAAAAAAAACGAAGGATACAAGCTGCCATGATAGCTGCCACAGGAAATTTCAACATAATAACAACTATGGTTCGTCGGTTTATGTTGGGTTGTTTGATCATCAATCATTTAATCGAGTCTAAAACGCTTCGTAGAAAATATATGGTGAGTTTGAGTAGTAGACGTGACAATATGTGGAGCATGGTGTATCAAAGTGATATTGCGAGTGTGGTTAATATTCGAATGAATATACTTGCGTTTTCGAAACTTTGTAAATTGCTTGAAACAAGAGGAGGACTGCATAGCAGCAAACACATGCTAGTTGATGAGCAGGTTGCAATGTTCCTACATACACTAGCTCATAATGAGAAAAACAAAATAATAGTAGTAAATAGATTTTAAAGATCGGGTGAAACTATAAGCCGCTATTTCAAGTTAGTTTTAGATGCCATATGCCGACTTCACAAAGAATTCTACAAGACACCTGTGCGAGTGCCCGACAATGAAACTGATGAGAGGTGGAAATGGTTTAAGGTAACTGAACTGTCTGTTTTTATTTTTATGAAAATAACATGTATGGATATCTAATAATTATTAGTTTTCATAGGGTTGTCTCGGAGCATTGGATGGTACTTATATTAAAGTCAGGGTTCCAACATGTGATCGAAAACCTTATCGGACACGAAAGGGTGAGATTTGTACCAATGTTCTTGGGGTGTGCACAAGAGACCTTATGTTTTCTTATGTACTTGCGGGATGGGAAGGGTCGGCAGCAGACAGTCGTGTGCTTCGAGATGCTATTAGTAGGCCAAATGGTCTTAAGATAACCCGTGGTATGTTACATATTTTCTTAATTTACAAAATCAACTCCTTTTATTACTAAGCTTTTGTTACTTTTTTTTAATAGAGTATGCTGCTATAAGTTTAGATGTAGATTGACACCATATGTGTTTCTTAAAGACTACTACGTAAAACATATATCCTTAGACCACACATAAAAAGCTATGGAATTTCATGTATTCTCATGCTTTTAAATATTGCCTTTTATGCCTCAATATGTCAAAGTTGTTGTTTATGAATTGTTAACATTCCTTTCACAGTAACATACATGTATCATGAGGAAACAATGTCACCTTTTCGGGTTGTGCTTTCAAGCACAGCCTAATGTTTACTTAGTCTTACATCCGCCTACCACGCCAAAGGAGTTATATAATATGAAGCACTCGTCTGTAAGGAATGTTATTGAGAGATGTTTTGGTTTGCTTAAGGCTAGATGGGCTATCTTGCGCGATAATTGTTACCATCCATTTGATGTGAAGTGCTGCTCTTGGATAGTTACTTAGTTTATCTTTACGATCATCTAGATGTAATCTTCTGTCACAACACTGTTTGTGAGTCCGTTGGCAATTTCTGCAAATCAGGACAGTGTTGGGATTGCTTTATTTTGTATGCACTTTTTTAGTTTTGTGTTGTCAAAGCTAGTAATGTATAGTTTTGTGTATCGAATATTATATATTTGATTATTAGTAATGGAATCATGTTGCTGTTGTTGTTTGTTAATGGTCAAATGGCTTTGTGGTAGTTTTTCATTTTTGTTTTTCATGTTGTGCAGCTGTGAACAGAAGGTATACACTATGATGGATTTGTGTGGCAACCTGCAAAAATGGTGATTTCAAATCTACTCCTACTGGTCTCCTACTTTGATTCAGCTTTGGTGTGCTGATGTATACAAAAAG

AaMyc-bHLH17

TGATTCATTTCATATGTATTTTTTAGGAAAAATTGAAGCCAATTAACTACAATAATTTTAGCCAAGCTATCCCTATGTTATTACCCGTTATATAATCTTAAATTTTCTAGCAAAATTGTGACCATTATTTAAGGAGAAGGACCAATCAAATCGGGTACTATCTCACATCTTAATCTTAGCCACGTGCCAATAAGTACTTGCTCCTTTTCTTTCATCCACCCACTAGGTTTTCTCACTCGTTTATTTCTATCTTTGCTTCACACTCTTTTCAATTTTCGTAACCAACAAATCAATCCTATGGATGATGATTTCCTAATTCTTTCACCTTCATCCTCTTCATCCATAATCCAACAAAACACTCACACCTCACCACCAACACCAACGCCACAATCACACCATAAGTTACAATTTCTACTACAAACACAACCTAACCATCCATGGGCCTATGCCATTCTTTGGAAAACAACTTTTAACCATGACAATGGTCGACCTTGGACTTTAACATGGGCTGATGGTTACTTTCTTCAAAACCCAAGTAAGCCCGCCCTTAAAGAAATCCAGTCCCTTCTTGGACCGGATAACTCAGACGACGCAGAGTGGTTCTACGTCGTCTCATTGACCCGGTCCTTTAGTGCTGGGGACGGTTCTGCCCCAGCAAACTCATTTGCTTCTAATAGTGTCATTTGGTTAACGGGTGCTCATAGTCTTTTGTCTTTTGATTGTGTACGAGCTAAAGAAGCTCATATTCATGGGCTAGAGACACTTGTTTATATTCCCACGACTAATGGAGTTGTGGAAATGGGGTCTTTTCATGTGATTAATCATACCGAATCTGATTTAGCTCATCGAGCTAAATCACTATTCGGTGCTGGTTCTTCCTCTTCTTCATCATCTTCGCCTCCCGCTAATAATATGGCAAAGCAACCCAATGATGACCATTTGATCTCCTTTGGAGAAATGGGTGCTAGGTTGCCACAAGAAGAAGAAAGTATGAACATTATCGATTTTGGAACAATCACGTTCGACCAACAGCCCAAGAAACTTGGAAAGATAGTCAAGAACATGAACATGAAAGGCAATGAAGTCTCGGAAACAGGATCCGAGGAGTCGGATTCTGATTGCCAGCTAGTTGTGGCAACCAGTAAAAAAGTGGGTCAAAAAAAGAAAGGACGCGATCCGCCTATAAACCATGTAGAGGCGGAGCGTCAGAGGCGTGAGAAGCTTAACCAACGCTTCTACACGCTACGTTCCGTGGTCCCAAATGTTTCAAAGATGGACAAGGCGTCCTTACTAGCGGACGCCGTTTGTTACATCAATGAATTAAAAGGTAAAGTAGAGGAACTCGAAGCTCAACTCCAAGCCACGAATAACCAACCCAAGTTAAAGAAAATAAAAATAGAAATACCGGATGTCACCATTGTACCGAAATCAAATGGGGCAAAAATATATAACAAAAAGACTCAAACCAAAATGATCGAAAATTTGGAAGTGGAGGTGAAAATGGTGGGTGAGGATGCGATGATTAGAGTACAATCTGGTAACGGGGATTGGCCCGCTGCGAAACTGATGGATGCATTGCGAGAAATGGAAGTTAAAGTGCACCACGCTAGCATGTCGTGTGTCAACGACATTATGCTTCAAGATGTAGTCGCTAGGATTACTGGTTCTACTGAAGATGAAGTTAAATCTCATCTTCTAGCTAGATTAAATCAGTAACTAGATATTAGGTTTAGAGTTTGAATTTAATCTTATGATCATGTCATGTAATTTAGCATCTCAACGATCTTTATCTCTATGTGTCGTTTAGTATCGAGTAAGATTGTTTTTCCTCAAGGCCTTAAATGTATATATCGTTTAAAAGAACTAGCATGATATAAGTAAATTACAATGTCACTAAAATGATACTTTCGGTTAGGCGACCCTAAAGGTTACAACAACACGTAAAACAACTTTTGACACACGAGTACACGAACACAAAAACTATCATGGTGAGTAAGCTTTTGTCACAAGACCATGA

AaMyc-bHLH18

TTGAGCACAAAACAGCTTTTTCCCTTTTAGAAATTTGACGCCATTTTTAAATATTATCTTAAGTACCATATAATACATAACCTTCAAACATCATAATCATCTATCATCATCTTGTAGTACTATTTCTGGCATTGTATCAACATGACATAACCGACCATTCTTTCATTCATTTCTTGGAAAATAAAATTTCAGCTTTCCTTTTCTTAATCATCTCCAAAATATTCCAACATTCTTTTTTGTTTTTAACTGCTTTTTACACAAATTTACTTTGCAAAGCACTTATTACAGGTGGATTTTTATTTTTTTGGTTTTAGCTACTTCGTTTAGTGCATCACAACATTCATTTTACATTATTATTGTTCACGAAAAGAGACAAAATTCATATTCAGTAAACTCAGTTTTTTTTTTGAACGACAACTAGATATTTTTCTTAGTGTTGAGTGATATTGTGTGTAAAAATGGGTACATTACAACAAGATTCAATTGTTAAAAATGTTATCAAGAATCTTTGTTGCTCTTATGGATGGTCTTATGGTGCATTTTGGAGCTATGATCAACCAAATTCCATGTATGATATTCCCCTTTAATTATCTATTTTATTTACATTTACTTTAATTTTATTTACTATTTCTTTCTTTTATGTTTTTGATATAATGTATTGAAGTGAGTATCAGAAAATTTAAAGATTATATCTTTTTAGTAGTGTTAGCAAGAAGTGAGATCTTCACAACAATAATTCCATAAAGAAGATATTCCAAATTTACTGCTCATAAATTCAACTTTATTAACAAAAGTTTAACACCTTTAACTTCAACTGGGCATGTTTAAGCCTCATTTGATTCAAAAGCTTCAAGAATTGCTCTTGCTATACACTTTACATACATTTTTATTTTTGATTTGACTGATTCTTTCAACCTTTAAATACTATTTGCTTCATAGATTATTGACACTGCAAGATGCTTACTTTGAGGAAAAATGCGGATCATTGATCGATAATTTGGTTCGACAAGTACCACTTGGAGGAGGGTATGAATTTTTTACCTATAACTTTGTATAAAAACTATGCATTTTTTTCATAATTATTTGATGAAAATTGTGACATTAAAACAATTAGGCTAGCTTTTCTTATATATGTTGATAATGATAGAAATCTCATTTCTTATCATTTTTAAGGATAATTGGACAAGCTGCATACAACAACAAACACACATGGATGTCCTCAGAAGATCATTACAATGAGCAGAATTTTTCGGGCTCGATATGGGATATGTTTCTGGTATAGATTACTTCTTATTTCTTGCGGGGTTACTTTCTCTATATTTATAGTAAAAGTCTTCGTCTTTATTTAAGATATCAAACATTTGACGCCAGTTATGGATGCTTATTTGTTCTAATAAAGTATCTTACATCGTGGTTTGAAAGTGATTATATGTTTTGTGTGTTTTCTACTCACAGATAATCAGTATATATATAGCCTTTGTGCACCGTATAGAATAGTTATATTTGATTATTTACGGGTCATAATAATCTGCTATAGTTAGCTAAGAAATAGTCGTAATAGCACTGATTTTGAAATTTTGTTGTAAGTAGTCATCTTACTTGTAAAGCAAATTATCATAATTTGTCAAGCACTCAAACAACAAAAGCACATCCAAACATTCCCTAACTAACACCCTGAACTTACTTTTCAGGATGATTATGAATTTCGTCGGCAGTTTTCTGGCGGTATGAAGGTACTTCTTTTCGTTTAATCCTATTTTACAGGTGTGATGGATATCTGGTCAAGTTGTACTTACTGGAGCATTCTTTTTACAACGTTAACTCATTTTCTCTCATGTTTTGCAGACAATTGCAGTCATCCCTGTGGAGCCACGAGGAGTGGTACAATTTGGGTCCATTGATAAGGTTGGTCCTATGATCATTGATCACTCATAATGTACTCTTTTAGATGTTTCAAGAAATTTTGAGTCCTTATATATGTTTGGGTCCTATGACAAAGTTGCCGTTGGTCCTATGATCATTGATCTCTCGTAGTCTAGTCTACCTAACATGTTCTTACATGCAATGTTTTGTTTTTAAATGTTTCAGATTCTTGAGACGGTGGAATTTGTTAATCAAACAAAAATTATGTTTCAAGAAATTTTAAATCTTGGAGGGTCTGAAGTTGGTTTGACTTCTTTGGATGGTCAAACATGTTATCAGAATGAAGCGTTCGCTTCCTTGATATCACCCCAAGAATCTTTCTTTACTGATTTTGGTATCCCAGATGAGTTCTTTCAAAATGGAACTTTCCCTCCACTGAATCCCATCGATTCTGATGGAAAACACGAGTCTTTGACTATTTCTGGTACTGATGTTGACCTGCTCAGAAACAGTGGTGATTTGGGACACATTTTAGCTCCGTTTATAGATGGAAGTCACTCGGGTTTTCATTCTTACAGCTCAGAATGTATGTCCATGTCAAAGTCAGCACAGCGTGTTGACTGTACTACCCGTGGTCCCAAAGAAAGATTGTTCTCAAAGCTTGGTATCGAAGAGCTTTTGGAGGGTGTTTCTGGTATTTCAAATGCTGATTCATTGTCTTGCATTGATGGTCAAATTTCAGCAAAAAGAAGAAAAACAGGAAATTCAAAGTGGGAAGAGGTTATGCCTATATCACAGCCTTGTTTACACATGGTTGATGGTTATAGTGTGAGTGATTCGAGCACAGTTATGCAAGCTAAGAAGCAAGTTGAACCATTAAAGCCCATCAAGAAAAAAGCCAAACCAGGGACTCGTCCCCGACCCAAAGATCGCCAACAAATTTTGGATCGGATGGCTGAGTTAAGGCTACTTATTCCCAATGGTGAGAAGGTTAGTATTCACACTTATTAGACATATTACTTCAACATCTAATAGCACTAATTTTTGACTTTTTGGTCAAACGAGTCATTTTTAGGGTGCTGCCTTATCTCATGATACACCACTTTTTACATATTGGTGACTTGATGCACCAGAAAAAGTGGTGCATTCGTTTGAATAACATGTGCATTAAGAAACATTTCACCACTTTGATCCTCTCCTTTTGCTTTGCAAGAAATGACTAGTGTGACTCGAAAAGTAAAGTTAAATGACAAACTTTAACAATGTTCCTGATTGATACCACAATCTTTATTGATAATCAAAGTACCATATGCATTGTTAAAAATCCAGTTTTTCACAATAGAACAAAGCATATTGAGATTAGGCACCACTTTATAAGAGATGCATATGAAAAACATTTGATAAAAGTGGTTAAGATTCACACAGATAATAATGTGGCAGATTTATTGACTAAAGCTTTTGATGGACCTAGGTTTCAACACTTAGTTGTCCACATTGGGATGGTTAACCCTTAAATGCAAAGTTTGAGGGGAGGCATAAGCCTCAGGGGAGGCATATGCCTCAGGGGAGGATTGATTGTTATTGTAAATATTGTATATTTTAGGTTGCTAACACTAACCATTTAATGTGTTTCTTAGCATAATAGTTTGTTTGTTTGGTTTGCTACTATGTGTCTGTTTTACAGAATACAGCTGTGTGTAGTTGCCGTTTTGGATTGTTTAGAACAAGGTCCTGTAAGCTAAAGTTTGTTTATCCCGATGGAGTCATTGTTTCTACTGAAGAAGGATAAAAATGATTCTCATTGCTTCTACTGAAGTAAATACAACTGATTCTCATTGTTTCTACTGAAGTGGAATACATGTGATGTTCATTATTTCTACTGAAGAGAAAGACAAGAAGTGCAATGAAAGATAAATTCAAGTTGTTCATTCATTCAAGAAACAAGTTACAGCTTACAAACAAGTTCAAAAGAAATTAAAGACCATTCCTATACATATTCAACTACACAAATCCTAAAATTCTATCATCCATATTGCAAAAGCCTGGTGGATATGCATGTCCAGTACGATAAAGAAGCTGAAGATGAAAATGGAAGGATGGATTGATGAAAACGATGATGAAGATGCTCAAGAACCTATGTGTTTGGTCTCTGGCAACATCACATCTTCAACTACTCCACCTTCCCCTTCCCTTTGTCTTCTCCTTTGCCTTCTTGAGCAGGAAGACCAATCACCACCTCTCGTGATTTTGCAAAACTCTTGCATGATGATCAACAAGCTGATTTGGCAAGAATTCATGCGATCAAAGTTAGAGAAGTTGAGCTTGCTGCTTCTATGGAAGTACTAATTAGGTTGCAGATGGATGCTAATGTAGCTGCTGAAGTATCTGCTGATGTTCCATTGGTTAATACTTCAGTGGAAACACCTGAGTCAGCAGATGTCTCTGCTACTGAACCTTCTTCTCATCATTCGATATATATTCCTGGTAGAAGGGCTAAAAGAATGGCAAGGATGAAAGTCTCGTCTTCTTCTGTACATAGGGAAGTGGATCTAGATGCTGCTGAATCTTCATTTANAAGTATCTGCTGATGTTCCATTGGTTAATACTTCAGTGGAAACACCTGAGTCAGCAGATGTCTCTGCTA

AaMyc-bHLH19

TTTGGAAGACGAGAAAACCTAAAGATGTGTGTGTGTTTTTTTTTTTGAAACGGAAATAATCAAGAGAAAGAAAAAAAATGTTTAAGTGAAATTATGAAAGATAAAAAAAAAAAGAGAATAAAAAATAATTAAAAGTACATAATAAAGTACAAAATAAAATCTTTAAGTGAGATTTGGAAAGTACAAAGAAGATCTTTCCAAAAGATTCTCTGCCACCTTTTTTATTTTTAAAATAGATAAAGTACACCGTCAGTCGAGCAGCCGTCAGTCGAGCAAGTGAGTCGAGCAATAAAATATATTAAAGTACATCCACTACCAACAGTAACAAAGATCTTGTCAGAGATCTTCCTGCACCCAAAAAAAGTAATATTAAACTGATAGTGGGACACGTGTCGCGCATGTAGGCTGGTTGAGCAGTCGAGCAGTCGAGCATGACCAAAATGGGAATGAGGTCAAATCTCGACCAAAACTTGAAATGTTGAAAAGTTTTTGGCCTAAATTGGTAATTTTCTCAAATAACAAATAAACAGTTTTCGATAACAGAATTCTCCTTAAAATTCCCAATTTTCTGTCTCATACTAGGGTCAGCTTTAGTAGGTATTGTGTTTTTAGCTTTGAGCACAAAACAGCTTTTTTCCTTTTAGAAATTTGACGCCATTTTTAAATATTATCTTAAGTACCATATAATACATAACCTTCAAACATCATAATCATCTATCATCATCTTGTAGTACTATTTCTGGCATTGTATCAACATGACATAACCGACCATTCTTTCATTCATTTCTTGGAAAATAAAATTTCAGCTTTCCTTTTCTTAATCATCTCCAAAATATTCCAACATTCTTTTTTGTTTTTAACTGCTTTTTACACAAATTTACTTTGCAAAGCACTTATTACAGGTGGATTTTTATTTTTTGGTTTTAGCTACTTCGTTTAGTGCATCACATCATTCATTTTACAAAATTATTGTTCGCGAAATGAGCGAAAATTCCTATACAGTATACTGAGTTTTTTTTTTTTTTTTTTTTGAACTAAAACTATATTTTTTTCTTGGTGTTTATTTATATTGTTTGTAAAAATGGGTACATTACAACAAGATTCAATTGTTAAAAATGTTATCAAGAATCTTTGTTGCTCTTATGGATGGTCTTATGGTGCATTTTGGAGCTATGATCAACCAAATTCCATGTATAATATTCCCCTTTAATTATCCATTTTATTTACATTTACTTAAATTTTATTTACTATTTCTTTCTTTTATGTTTTTGATATAATGTATTGAAGTGAGTATCTGAAAATTTAAAAACTATATCTTTTTAGTAGTGTTAGCAAGAAGTGAGATCTTCACAACAATGTCACTTAAAACTTTAATTCCACCAAGAAGATATTCCAAATTTACTGCTCATAAATTCAACCTTATTAACAAAAGTTTATCACCTTTAAGTCTCATTTGATTTAAAAGCTTCAAGAATTGCTCTAATTGCTATACACTTTACATACAGTTTTATTTTTGGGTTGACTGATTTTTAAACCTTGAAATACTATTTGCTTCATAGATTATTGACCCTACAAGATGCTTACTTTGAGGAAAAATGCGGATCATTGATCGATAATTTGGTTCGGCAAGTACCACTTGGAGGAGGGTATGAATTTTTACCTATAGCTTTGTATAAAAACTATGCATTTTTTCATAATTATTTGATGAAAATTGTGACATTAAAACAATTAGGCTTGCTTTTCTTATATTTGTTGATAATGATAAAAATCTCATTTGTTATCATTTTTAAGGATAATTGGACAAGCTGCTTACAACAACAAACACACATGGATGTCCTCAGAAGATCATTACAATGAGCAGAATTTTTCGGGTTCGATATGGGATATGTTTCTGGTATAGATTACTTCTTATTTCTTGCGGAATTACTTTCTCTATAAGTTTAGTAGAAGTCTTCGTATATATTTAAGATATCAAACATTTGACGCCAATTATGGATGCTTATTTGTTCTAATAAAGTATCTTACATCGTGGTTTGAAAGTGATTATATGTTTTGTGTGTTTTCTACTCACAGATAATCAGTATATATATAGCCTTTGTGCACCGTATAGAATAGTTATATTTGATTATTTACGGGTCATAATAATCTGCTATAATTAGCTAAGAAATAGTCGTAATAGCACTGATTTTGAAAATTTGTTGTAAACGGTCATCTTACTTATAAACCAAATGATCATAATTTGTCAAGCACTCAAACAACAAAAGCACATCCAAATATCCAAACATTCCCTAACTAACACCCTGTACTTACTTTTCAGGATGATTATGAATTTCGTCGGCAGTTTTCTGGCGGTATGAAGGTACTTCTTTTCGTTTAATCGTATTTTACAGGTGTGATGGATATCTGGTCAAGTTGTACTTACTGGAGCATTCTTTTTTCAACGTTAACTCATTTTCTCTCATGTTTTGCAGACAATTGCAGTAATCCCTGTGGAGCCACAAGGAGTGGTACAATTTGGGTCCATTGATAAGGTTGGTCCTATGATCATTGATCACTCATAATGTACTCTTTTAGATGTTTCAAGAAATTTTGAGTCCTTATATATGTTTGGGTCCTATGACAAGGTTGCCGTTGGTCCTATGATCATTGATCACTCGTAGTCTAGTCTACCTAACATGTTCTTACATGCAATGTTTTCTTTTTAAATGTTTCAGATTCTTGAGACGGTGGAATTTGTTAATCAAACAAAAATTATGTTTCAAGAAATTTCAAATCTTGGAGGGTCTGAAGTTGGTTTGACTTCTTTGGATGGTCAAACATGTTATCAGAATGAAGCGTTCGCTTCCTTGATATCACCCCAAGAATCTTTCTTTACTGATTTTGGTATCCCAGATGAGTTCTTTCAAAATGGAACTTTCCCTCCACTGAATCCCATCGATTCTGATGGAAAACACGAGTCTTTGACTATTTCTGGTACTGATGTTGACCTGCTCAGAAACAGTGGTGATTTGGGACACATTTTAGCTCCGTTTATAGATGGAAGTCACTCGGGTTTTCATTCTTACAGCTCAGAATGTATGTCCATGTCAAAGTCAGCACAGGGTGTTGACTGTACTACCCGTGTTCCCAAAGAAAGATTGTTCTCAAAGCTTGGTATCGAAGAGCTTTTGGAGGGTGTTTCTGGTATTTCAAATGCTGATTCATTGTCTTGCATTGATGGTCAAATTTCAGCAAAAAGAAGAAAAACAGGAAATTCTAAGTGGGAAGAGGTTATGCCTATATCACAGCCTTGTTTACACATGGTTGATGGTTATAGTGTGAGTGATTCGAGCACAGTTATGCAAGCTAAGAAGCAAGTTGAACCATTAAAGCCCATCAAGAAAAAAGCCAAACCAGGGACTCGTCCCCGACCCAAAGATCGCCAACAAATTTTGGATCGGATGGCTGAGTTAAGGCTACTTATTCCCAATGGTGAGAAGGTTAGTATTCACACTTATTAGACATATTACTTCAGCATCTAATAGCACTAATTTTTGACTTTTTGGTCAAACGAGTCGTTTTTAGGGTGCTGCCTTATCTCATGATACACCACTTTTTACATATTGGTGACTTGATGCACCAGAAAAGTGGTGCATTCGTTTGAATAACATGTGCATTAAGAAACATTTCACCACTGTGATCCTCAGTTAAATGAAAAACTTTAACAATGTTCCTGATTTTGTTATGAATCTACAGATGAGTATCGACTGTTTGTTGGATCGGACCATCAAGCACATGATTTTCATGCAAAATTTAGCGAAACAAGCTGACAAAATAAAACAGGCGGAAGAACGAAAGGTGAGCAAAGTCATGTTCATTAGTTGCGTAATTTGTTGAACACCCGAACAGGTAACATTCAAGACTCTACCTTTAACATGGTTTATATAATTGATCAGCATAACAGGATTGACTCCAATGACACGAGCACAAATGGAGTGACATGGGCTTGCGAATTGGGAAATCAGACAATGGTTTGTCCACTAATGGTTGAGGACCTTGATGAACCTGGTCAAATGCTTATAGAGGTAAAAACACAGATACACATAAGTTTGCATATGCTCATGCTAATTATTTGTGAATCTGATATGATTTAAATGTACATATATATGTTTCAGATGATTTGCGAAGAACAAGGATTCTTTCTTGAGATTGTAGACATAATTCGGCGTTTTGGGTTGATAATCTTGAAAGGGGTTATGGAAACTCGCGGTGATAAAATATGGGCACGCTTCATAGTTGAACCCGAGGTATGTCGTTCAAGATTCAAGAGATGATTAAGAAACGCCGTTGTTAAATAGATAAGAAAAGGGTGAAATCTTAGTTGCTCAATTTACTTTGCTTTTTACAGGTGAACAAACACATAACAAGACACGAGATATTTTCAGCACTTGTTAAGTTTCTACAAGAGAATGCGCACATTGTTGATGAAAAATGCACACGACAAGGGAATTCTCTACTTGGCGATTTTCAACAAGCTGGGATACAAAATATTGGGATACAAAATCTTGTTAACTTAGCAGATATGCAATATTTTGTAAACTTGTAACAAAGGTTTCAAGAATGCTTTCACCATTTATCTAATATAAAGTTTGTTATATATGTAGAATAAAATCAGTGACCTAAGGCCGTGTGTAATGGTCACCTCGGTCCCCTTATTAGGAATTATATTTTTTTTAGTGCAAAGGTTGATTATGATTAATCTTCCAATCATATGTTGACATCCTACAAATAGAAGTAGGGATTGACACATTCCAAAACTCCTATTACCAACACAACACTGATTGCCTATACTTAATGATCAAGATACGTTGTCAAGCTATACAATGACTATCCTGAATAAATCTAATCTCAATACCACTTTTCTGCATTAAACAGAGTATCATCAAAACATTGCACTAATACCTGTTATGAATCTACAGATGAGTATCGACTGTTTGTTGGATCGGACCATCAAGCACATGATTTTCATGCAAAATTTAGCGAAACAAGCTGACAAAATAAAACAGGCGGAAGAACGAAAGGTGAGCAAAGTCATGTTCATTAGTTGCGTAATTTGTTGAACACCCGAACAGGTAACATTCAAGACTCTACCTTTAACATGGTTTATATAATTGATCAGCATAACAGGATTGACTCCAATGACACGAGCACAAATGGAGTGACATGGGCTTGCGAATTGGGAAATCAGACAATGGTTTGTCCACTAATGGTTGAGGACCTTGATGAACCTGGTCAAATGCTTATAGAGGTAAAAACACAGATTTTGCATATGCTCATGCTAATTATTTGTGAATCTGATATGATTTAAATGTACATATATATGTTTCAGATGATTTGCGAAGAACAAGGATTCTTTCTTGAGATTGTAGACATAATTCGGCGTTTTGGGTTGATAATCTTGAAAGGGGTTATGGAAACTCGCGGTGATAAAATATGGGCACGCTTCATAGTTGAACCCGAGGTATGTCGTTCAAGATTCAAGAGATGATTAAGAAACGCCGTTGTTAAATAGATAAGAAAAGGGTGAAATCTTAGTTGCTCAATTTACTTTGCTTTTTACAGGTGAACAAACACATAACAAGACACGAGATATTTTCAGCACTTGTTAAGTTTCTACAAGAGAATGCGCACATTGTTGATGAAAAATGCACACGACAAGGGAATTCTCTACTTGGCGATTTTCAACAAGCTGGGATACAAAATATTGGGATACAAAATCTTGTTAACTTAGCAGATATGCAATATTTTGTAAACTTGTAACAAAGGTTTCAAGAATGCTTTCACCATTTATCTAATATAAAGTTTGTTATATATGTAGAATAAAATCAGTGACCTAAGGCCGTGTGTAATGGTCACCTTATTAGAATTATAATTTGGCTTGTTCATTAATTAACTCATGATTACAATATATAATCTTTTGAGGATTATTTTAGAACTCAAAATACAAAGTGGGCTAAGCCTATCAACAAATAACAAAAAGCCTAATGACTTGTATATAAAATAATCATCTAACACTCCCTACAGTTTGAGCGAAAGAGGATCGAATACGAAATTCTTCAAAAAGTGCAAAAGGCAACCCTTTGGTGAAAATGTCAGCATATTAGTAACATGATGACACATAAAGCACACGAACCTAACCCCGGGCAACCATATCTAGTACAAAAATGTATATCTATCTCAATTTGTTTGGTTCATTGATGCTGAAAATGATTAGTGGTCAAATAAATGGCACCAACATTGTCACAGTAGACAAGATTGGCAGATAATAATGGCGTATAAAGTGATCGATGT

AaMyc-bHLH20

TTTAAGAATTGTTTTACTTCACTTATAAGTATAGTTTTGTTATAATTGTACACACTTATATACGTATTTGATAATGGCTACAATCAGTTATAATTCACTTATAAGTGTAGCTTTGTTGTAATTGTACACTAACAAGTGTGGCTTTTTAAGAATTTGTTCCACTTCACTTATAAATGTAGTTTTATTGTAATTGTACACAATTATATACGATTTTATAATTGTACTTATGAAATAGCTTTGTTTGTTTTTTTTACTTTCTTGTAATTATTAGTGTTAGTGCGGAGAATTCAACTATTTATTGTAGTGTAATAATGTATTTATGACATAATATATTAAATACATATCTTTGATAATAATCAACATAATCCAAACCTTGAACCTAATGGACCCAAAACACAATTTGGGCAAAGTAACCTATCTAATAGCAAGTACTCCGTAGTCTTTATAAACATGCTGCATTATTGCATTAAGGTAGTATTTATAATACATCACCGGTGCAGCTTGGACGTTTGATGGAATTACTTAACTCAACAACCATGGCACAATCCTCTCATCAAACCCGTCTCAAGTTCATCCTCCAAAACCGACCCGAAAGGTGGCTTTACGCCATTTTCTGGATAGCTTCCAAAAAAACAGACGACCATCTTGTTCTAGAATGGGCTGATGGTTATTTCCCTGAAACCAACTATGTGTTTGGACTAGATGATGTTTCTGATACCCAATGGTTGATTATGTCATCCCTAGGGATGTGTTTTACGGCTGGACATGATGTTGTCGGCCAGTGTTTTGGTTCAAGATCTTGCGTGTGGTTGGCAGGTGATATAGAGTTAGGGAAATATGATAGTAAAAGATGTGAAGAAGTGAGAGTTCATGGGATTAAGTCTTTGGTTTGTATACCAACTAATAACGGTGTCGTTGAATTGGGTTGTTGTGATNTTTTTTTTTTTTTTTTTTTTTTTTTTTTTTTTTCCTTTAATGGTTTTACATGACACCCATGAAGCAAGATCAAAGTTATATATGACTAATTTTAATTAAAAAATGAAAATTCTAGAAAGAAGATTAGATATATTCTGTCTGCTATAGGTGAAATATTTAATATAATCGCTAGTAATTAATTCATGAAAAAGATTGAATGATAAGTATTTTAGTATTTTGTTCAAAAAAAGATTAGTATTTTAGTAGGCTGGGTGCAATTGGACTCCGGTATATTGTAGCTAGAAATTTTAGTTATAATATAGGTAGCTAGAACATCTTTTTTCTGTGTATAGACACTAGTTAATCATCGATCATTCGATCACATGATGGAGTAATTCGCGCGAGTTCTATGGCTACGTAGTCCCTTATTTTTCTTAGTTTTGCCTAACAAAAGCTAGATCATTTTTAGGCCTTCAAAATCAAGTAGGTGATGGTCAGGAAGAAGTGATGAGCACAAAGAAGATGAAGATGTCCTCTTCTGATTCAGATCCACTGGAAATGAATAGCTCATCATCATTAACAACCAAGAAAACATGTACACCGAAGAGGAAGGGTCGACGAGTAAAGGGCACAATCGCACAGCCAGAAGTATTGGTACCTGGGTACCATGTGGAGGCAGAGCGACAACGAAGAGAGAAGCTAAATCATCGCTTTTACGCACTTAGAAGTGTTGTTCCATACGTTTCAAAGATGGACAAGGCTTCTCTACTAGCAGATGCAGTTACTTACATCAATGAGCTCAAATCCAAAATCCAAAGGTTAGAAAATAATAAAGAGTCTGAATCTTCTCTAATAAGACCTAGAAATGGCAACCAGCTGAACATCAATCAATGTAACCATGTTCATGATCAAAGAACTACTGGTCATTCAATGTCTAATAAGGTTGAAGTCGAGGTGAAGTTACTTGAATCGGAAGCAATAATTAGGGTACAATCTGCGGAAGTTAATCATCCGGCATGTAAGTTGATGGATGCACTTAGAAGCCTTAATCTAAAGGTTAATTATGCAAGTGTCTCTTGTGTGAAGGATTTGATGTTGCAAGATGTTATTGTGAAGGTTCCTAACGGATTCACAAGTGAGGAAGACACCTTACGACTTGCGATTCTTAACAAAATGTGCTTGGATTAACAAGTAGTTATATTTCCATCTATTGTACCAGCATTGTTAACTATCAAAAAGTAATTACTGCATAAACGACGTGTACTAGTCCCGTTTATAACTATCTACACTAAGTTTGAAATCCAATATTATGCTAAGTGTGAAATCCAAAAGATTACGCACTTATGCTTTGTGGATGTGATGCGGCGGTTGCAACACCGAAATTTTATAACCCCATTGTCAGTGTACCTAACCGTTGTAGTATAGTTTAGGCAAGACCGAGAGAAGATCATTCTCTAGAGGAATAATGAGGTAGGTTTGAGACGTATGACAATTTGATCTCAAGACGCAAGGAGTTTATTTATGTTGTTTTTATATTTTATGAAAGCAAGTAAAAATCCATTATTGAGGACATTTGTTTTAAACTATATTAACCTAAATGCAAGAAAAGCAAAATAATAAATAAATAAATAAATAAATAAATTTTTAGTGTGAACTTTTAAATGGAAAAAGTGTATTTATCTAGCATGCACTACT

AaMyc-bHLH21

TCTATTTTCTTTTTCAAGTAAAAATTAGAGAATTTAGTAAGGAATATTATATGTGAGTGGTTAAAAATTAAAGTGAACCAATTTTAATTAAACTGTGTGACTTTTGTCTCCGGACAATAATTTGAAGATGGAGGGAGTATAATTTTTTTTTTGGCATAAAACATATTGACCTCTTTTATCGAGGTATTTTTGTCTCGTAAAGTACAGTACTATTTTTCAGCCAAAATCAAAATTGGTCGTATTCGGTCAATATACTATCAAACCATATATAGTACGATCACAGAGTATCAGATTCCTTTCACTATGATACGAGTAGTTTTTAAACTCATTTAAACCATACCCATTTCACAAAACTTATTAAAATTTCATTCCAAAAAAAAAAAAAAAAATAAAAAAAAAAAAAAAAAAAAAAATGAGTGCATTAACTGAGCGGTTAAGACCTCTTGTAGAAACTAAGTCATGGGATTACTGCATTGTTTGGAAATTTGGTGATGACCCTTCTAGGTACATTGAGTGGTTTGGTTGTTGTTGTAATGGTAGTAGTAATCAAGATGTTTGTGGCAATGTCAAGAAGGAAATCGAAGAAACGAAACCGCGTTCTTCTCAAGTGTGTAGAGATACTTTTGTTGAACATGGTTTGGGTACGAAAGCTTGCGAGAAACTAGCTGATATGCCTTTTTATCTGCCACTGTACTCTGGGTAATCATCTTTATGTATTTTTATCTTTGTGTGCATTGTTATTATCTGTAGTTTGGTTATTTAACTGAATCGCGTTAATGCCGGATACTAAACAGGGTCCATGGTGAAGTTGCAATGTCTGGTCAACCATCTTGGAGTCATGTGAGTATGCTTGTCAGTAAATAAAAATGTGAATGGCTAATTCTTATATAACATTGTAACAATCTCTTAATGTTTTTGTGTAAACTTTATAGGATACTATTGGGACTCAAGTTTTGTTTCCGGTTAATGGTGGGTTGCTTGAGCTCTATATCTCAAAACAAGTAAGACCCGTGTGTTTAATTCTTAGAAAAACAAGTTAAAAAGTGTTTTGAGACTAATTTAATGTGAACTGATGAGTGTTTTATGTTTTAGGTTCCGAGAGATGAAGAGATGATAGAGACTCTTACGGCACAGTTTAATGCACTTTCTAAGGATGAATGGTTTTGTGACACGAAAACTGCTGCACAACACATGGTGTATCTGAAAACTGAAGGATCGCCAAATGGTGAAGGACTATGGAGTGAAAACTCGTCACTTGTTTCAGCTGGTTCGGCGCAAGTTTCACCCACTCAGTCCATTGATAATCCGATAAACGTGTTGGGTGACATGAAGAGTAGGCAGAAGAATGGCAAAGAACAATACCAATCTAAGAATCTTGTGACGGAAAGGAAAAGAAGAAACAGAATTAAAGAAAATCTCTATATTTTACGCTCTGTAGTCCCTAAGATTTCAAAGGTTTGTATTTGTTATTGCATTTTATGAAACTTTTCATTCAGTTTTAGGCTTATAAATAATCTGAAAATCTTGAACAGATGGATAAAGCTTCCATACTTGGAGACGCAATTGAGTATATAAAGGAGTTGCAAAATAACGTTCAAGAACTTCAAGATGAGCTTAAAAGATCGGAAGAAGATGAGATTAAGTCTCATGAGGAAGAAGTTGAGGTCTGCAAACCGAAAAGAAAACGAGCATATGAACACTCGCCTACAAAAGGACATAGTCTTGTTTCAACATCACCAGACAAAAAAATTGAGGTTGATTGTTTACTTTTGGGAAAAAAAAATAAAAAAAAAAAATAAAAAAAAGCCGTTTTTGGTGAAATTTAGATGTCAAATTCTTCTAAATTTGTGCTTTGTTTTCATGAAGGTTACAGTGGAAGTGCATCAAATTGGGGCTAAAGATTTCTGGCTTAAGCTAGTTTGTGGCCAAGAGCGAGGGGTTTTTAAGAGGATTATGGAGACTCTCGATTCTTTGGAGCTTCAAGTAATTGATGTCAATGTCACTACTTGTTATGGCCATGTCTTGACAAATCTCAAAGTAGAGGTATGCCTAATTTTTTCCCCCCCTGTCATGTCTGTTGCGCCGGCTAGTAGTTAATAGCTAACTGTCATTTCAAGGAGTCAAAGAACATGCCATTTTAATAAGGGCTTATTATTAGTATTTATTTCTCTTAGCCTAAATGTTTTTTTGATAACTGATTGTTGATCTGTTTGTGAAGGCAAAAGGGAAGGAGGTTGTTGCAGCCAAGAGTTTGAAAGACTTGTTACTAAATTGTTGGATGCCTGGTATACATGACGAGAACCAAAGGCGCGGTTGATGGGGAAGTTTACTTGAATGTTAATACACATTTCTAAATGATGAAACTAGTACTGTTGGTCACGCTGTCAGGAGTCATGGAAAGCATGATTAACTTAATTAGCACATATGATTTCAAGTTATTATATTTCTAGATGCTAACATCATACGTAGCAACACATTATGTGTCTAATGGGATGAGTTGCAACATCACTTTCGATATATGAAACTATGAATTATATCTATCACTCTACTTGATAATGAGTGGGTTAAGCATGTGACCCAGCATCTATAAGCATATGTTTCGTATATGTGTAGTTTATAAGCACAACCCTTCATTTTTGTTCCCTTAAACATGATGATTCACATATTATTTTAGGTTCCACTAAGCTCAATTGCAATTAGGAAAAATACAGATACCAATTTAGATTCCAGAAAGCTCATAGTTCATAGCACTGGGTGTAGTGAGGAGCTTCCCCTCCTTATTTGGGGCCCACGGCATCACTTTAACAAACCTCCCCAATTAAACCTTTCACTAACAACTAACCCCAACTTACTCATTTGGGCCCACCACTTACAACTTTATTTTTTTTATTCAAATTTCTAAATAAACTAAAATCATTTTATTCAAATAAAAATATAAATAAAATTTTTTATAAAAAAGTTTAAAAAATAATAATAATAAAAACTTAACTAATAGATTAA

AaMyc-bHLH22

GAACTCGACCAAAACCCTAGACCGAGGACCGGACCGAATGTGTTCGGTCCGGTGCGGGTTGGTCCGGTCCGGTCCAAGAAGGCATAATGGTCGGTTTTTACGTAAGACTGGTTCAGACCGGACCGGACCCAGACCAAAATAAGGAAAATGTTAAGACCGAGACCGGACCGATGATCTTTTGGTTCGGTCGGTCCTATCGGTTTTCTCGATCTAGGTCGGTCTTGGGTGTGCCGTGTGCGATACTTAGCCCTAAATCTCGATCAAACTTTTATCTACTTTTGATAGTATAACAATTAATTTGAAATATCTTCGAAAAACGATAGTTAACAAATAAGTTGAGACCCCGTGAGTAACTATCAAATTCACGTTAACTATACAATCCCAATATCGGAGTGTACGGAAAACGTGTCATTTTCAGACCCACCAAATTATTCCTTTACTTTTCTGTTAGTCTTATACGAGTACTAATAATCACACCCCCAAACACTCACTTCATTCAAACAAATATCTCCTGCCCTTATCACCGTTTCCAAACACTCCCTCAAAACAAAAAAATGAATCAAATTATATAAACTGTTAATCTAATGGCTTTGTTAAATACATTAGACTTGCTAAGACCACTTATATCAACCAAATCATGGGACTATTGCATTGTTTGGAAATTTACTAATGACCCTTTAATGTACTATTAATATTTTTATTGTTATTAAATAGTTAATTTATGGGGTGTTTGTTTCGGCTTATGTTTTATAAGCTCAGTGGCTTATTTTGAAACTTTTCGGCTTATATAAGCCTATTGTAACATAAGAAACTTATACAAGCCAAAAAGATTTAAAATAAGCCACAAAAAGTGAGCTTATGAAACTTATATAAGCCGAAACAAACACCCCCTTAATGTTTTTTTTATGGGTTTTTAATAATGGGTTAATTTTTTTTTTAATCAGGTGTATTGAGTGGGTTGGGAGTTGTTGCAGTGGTAGTCAAGGTGTTTGTGGAAATGTTAAATGTGAAAATGAGGCTTATTTATGTAAAGATAGTTGTGTGAAGCATTTGATACGAACCGATGCTTGTGTAAAACTTGCTATGGTTCCGTCTTCTTTGCCGTTTTATCCGGGGTAATGTAGGGTTATCTGTCTTTAGGGGGTGTTTGTTTAAGCTTATTTTGGGGCTTACGAGCTTATGTGGTTTTGGCTTCTGGCTTATTTTGAAACAAGCTTATAAGTTTCAAAATAAGCACAAAAAGAAATAGGCTTATGAAACATAAGCCAAAAAATAAGCTTAAACAAACACCCCCTTAGTTTGTTAGTAGTTAAATGTAAGTATTTTTAATGATTGGATATAAGTTTTTTGACATTTGTATTTGAAACCAGGATTCATGGGGAAGTCGCAGTGTCTAAACAACCATTCTGGCAAACTACTGTAAGCTTTCTGTTTGTCTGTGTTTGGGATTGTTAACTAGATATGTTTGGAATAAGCAGTCACCAATACTGATTATTTAGAACTGCTTAATCATCATTTAAATAATGAGATAAACAGTTTGAAGGGATAGCTTATTAAAACTGTGTAATTGTATCCTTTATTTATGCGGTTTGATACAGAAAATGAATAGTTAGATAAGCGTTTGAGAGATTATTTATACAAACTTATTACAAATAACTCGTTTTGACCGATCAGCATGAAAATGCTTACTGTAAAAAACATTAAGCACATAAACAGTTTTAGTAAGTAATCCTAAACGCCCTCAGAACTCTTTGTATAGTGTATCAAAATACAATGATGAGATTACTAGCAAAACTATTTATAGCCAGAATAATTAAATTCCTTGATTTCTATGTAACAGGATTTGAGTGGAAGTCAACTTGTTGTGCCTGTTGATGGCGGTTTGATTGAGCTCTATAGATCAAAACATGTAATCCGTAACTTAGTTATTTTATCTGTAAACTTTAAAACTTCTTTTTTTGAAGTTAATTTAACATCGTTCCTTGTTTTTAGGTTCCCACTGATGAAAGGACAATAGAAGCACTTATTGCACGCCTAAGCAACATTGTTGATCCTAAGTTAGACGAAGAAGACTCGAAAACTCGTCTGAATTCATATCTTTTCGATCACATTGGTCCTAACCTACAGCTCCTTATCCCGCTTCCAGAACTCATTAGTCCGCTTCCTGAACTCGTTAGTCATCCAACCACCCCAGGGTTGACATCAGGTTCGTATCTGCTCTTTTATGTAGACTCCGTTTATGTTTTTGAACTCAGTTCATTGATGAACTAATAAACACAGTGACCTACAACTTGACAAAACGACATGATGGGGGGATTTGGGTCATGAGTCATAAGTCATTAGGAATAAGTTATTTTTTACGGGTCAAAACACGCTTTTCGTCCATCTATATGCGCTCTATAGAAGACATTAATGTATTAATCTCAGTAACAAACTAGATGGTTGAGATAATATGCCTTTCTGCCAATTTGGCTCGTTTCAAGTTTAGCGATAAAAATTATTTTATACTGATTATCATTTAAGTTCTAAAAGATCAAATGTTGCAAGCCGACCCATTTTGGGTAAACGAGTCAAATTTCCGCCTCTAATTATCTATGGATCAGGGAATGATAACGAAGTTATGGTGGCAAAGCAAAAGAAAGTGAAAAAACAATTCAAATCAAAGAATCTTGAAGCTGAAAGAAACCGAAGGAAACGAATTAAGGATAATCTCTTAATTCTACGTTCTTTAGTCCCCAAGGTTTCAAAGGTTCGGTTATTGATTTCCTATATTTGTATTATCTCTATCTGAATTCGCTGTTTATTTATCTTAAAGAGTGTGATCTTATCATCTGAGTAGATGGATACAGTTTCGATAGTTGGAGACGCAATCAAGTACATACAAGAATTGCAAGAGAATGCTAAAGAACTTGAAGACGAGCTTAAGGCACTTGAAGAACAAGATTGCATGGTTAATGGTCATGAAGTCAAGGTTAGTAAACCAAAAATAGCATATTATAGCTCTCCTCAAACGCATTCAAAATCACATATTCGAGCTTCAAATTCTGCTGATAAAGAAACTGAGTTGCAAACTGATGAACTTCCCAAAAAATAGAAACTGTAATTTGAGTAACAGGCAAAAGTTATCGTATTATTTATTTTTTAGGCGAAAATTAATTGATTTACAATTATTTACTGAAAATTGTAACACAGTCTATTAATTACAAAATTGTTTATGAAGGATGTGGAAGTGGAAGTACACAAAATCGGAGCTCGAAAGTACTTGCTTAAAGTGTTTTGCAGCCACAAACCGGATGGATTTTCAAGGTTAATAGAGGCTGTTCAGTCTCTTGGACTTCAAGTAATCAATGTCAGTATGACCACTTGTATTGGTCTTGTTTTAAACACACTTGCTGTCGAGGTGAGATAGAATCTACATGCTTCATAATATACATCTTGTTAACCTTGCAGCCTTCATATACTGATATACATTTGATTTAGCTTACACATCTTGTTAACTCCTGTACACTGATTATTGTTTTCTGAAGGCTAAAGAGGAGATCGATGCCAAGAGTTTGAAAGACTCGTTGCTTAGCAGCTGGACTTCTTCAGAACTAGATCAAAAGCTCTGATAAAAAGCACTTGATGAAGCATTTTTGAACTTGGAATGACTTAAGGATTATGGGATACATGGTTTATAACTTTATTTATAGAGTATACTAGAAACCAGAAAGCTAACAATATGATTATAATGGTATTACTATGATTATTACTGTAAGGCGGATTATAATTAGTAATGAAAACAACGCTAGGTTTATATCATGACGATTCTATCGTTGCCTAAGCATGAGATCAATACGTTAATAGTAGTCTTTGTTATGAGATTATGATTCTAGATGGCAAAGCTAGATTAAAGACAAGAATATACTCTCTCCCTTTTACAATATAAGTCCATTTTGTTACTTTTTATGTCCCAAAATTAATGTATAATTTCAATTTCAATCAATCAAAATGCTTTTAAATGAGTTAGAATGAAATTTTGTTGGTTAATATCGTGTGGGATTATTTTCAAAAAATTTTGTTGTGAAATATGAAGTGTAC

AaMyc-HLH1

AAAAAGAACCACCACTAAAGAATAAGAAGACCCACCAATAAAGAAATTTCCAAAACCAGCTAATTTTAACTTTTTGGTTCACCTTTTAGTAAGAAAAAAAAATGCTAGACTTTGCGATTTCATAGATTTCTTGACATGTCTAAAAATTGATGCGATGTTTTTGTTAGACTCGAAGTGAGTTTTGTGCATTTTATTTCTTTTAAAGACCGAGATGATTTTTTGTATTATTAGAAAAATGAAACCCACCATTGACATTTTTTTCTTATCTGAAATCTATGTTTTGTTTTATTTTAAAACTTACTCCATAAGAAAACAGGTCTCACTTTATTATAATAAGATAAAAATATCATGTCGGTCTAGAAAAATTTGGTCGCATAAACGTCACTTCTATCTAAGAAAAACCAACCCACCAACTTTTGAACAAATGTCGAAAATTTTATAGAATTGCACAATCTAACGATCGCACAAAACTAAATTTGAAAAACATTTTTTTAACTAGAAGGTGAACCCAAAAGTTCTTCCTACTAATATTGCATATCATGCTTTCACCTCATACCCCTCTTTAAATAAAGATTTGTTTATTCATATTAAGTTCAAGATAATATACAACATTATTTCACATAATTATTTTGTGGGGTGCACAAACTTTGCAAAGTTGGTGCTGAAATGGAAAACTTGAGGCAAAGATTGGCTATGGCTGTCAAAAGCATCCAATGGAGCTATGCTATCTTTTGGTCAACCTCTTCAACAGAACAAGGGTACCTCTTTACCTTAATTTTTGATAATATATGTTCATGATATAAGCTTATTAGCACATGGTACACTTTGTTGACCAAGATATTAAGTGAACATATCTCCATGAATTTATATGTATTAGCAGTAGTGTCTTCCATCATAGTTATTTTATGTGTTTTGATTTTGCTAGCTTTAGGAATGCAAGAAAAAAGTTTTTTTAGTCGGTGGTTCAAACCCCGCGGATTAGTCGGCCAAAAGTTCAAAAACCACGGTTACCCAAAACAGAAAAATATTCATTCTAGTGGATGAAGTTAGTAAAGTTAAACTAAGTTTTGAAGAGCTGTTACATATGAGCTGTGATGTTAAAGAACTTTTGAGCTTCTTTTAACAATTTTTGTCCTTGAATACCTTTATGAGTTGTATCGTATTATCCGTACAATAAAGCAAGAATTATGAATAAGGAATGCATAATCAAATGTGAATAGAATTTGATTTAGGGTATTGACATGGTGTGATGGGTACTACAATGGAGATATCAAGACAAGGAAAACGATACAAGCAGAGGGAATGAACGAGGACGATGATGATGGTCAAGTGGGATTGCAAAGGACTGAGCAATTAAAACAACTTTATGAATCACTTTCAGCAGCCGAAACACATCATTATGAACCACAAGCAAGAAGGCCGTCGGCTGCATTATCCCCTGAAGATCTCACCGACACCGAGTGGTATTTCTTGGTTTGCATGACGTTTGAGTTCGGTTACGGTCAAGGGTAAGCAAAATCTAACTTCTTCTCATTCATACAATGTTATCTTGTTTATATTATGTTAAAACATGCAAGTGAGCTAACCTTATTAGAACTCGTTGTTCTAATAGATGATAATGTTTACGAACTTGTCGATCCCAAAAATAAACAACACCTATCAATCAAAGGTCGAATATGCGTTCTTTGACTTTCGAATAGCAAAATCAAGAAAACTGAGGGTATATAGAAGTTAAAACCTGTTATACATGCAATAGAAAGAAACAAATTTGACTTGGCCATATTGTAACTTATTCAAATTTAGATTGCCAGGAAGAACACTGGCCAAGAATACAACTAGTTGGTTGAGTGATGCCCACTTAGCCGATAGCAAGGTCTTCACTCGCTCCCTTCTAGCAAAAGTAAGCGTTACTTTTTTCTTTTTCTTTTTTACATTAATCTCGCTACTTTAACTTTGCAAAATTTTGTTATATTAATCTGAAATATTCGATCTAATTATCTCTCTTGTTCCGTCGCTACTGTCTTCCTCAGAGTGCATCAATTCAGGTAACTCATTATTTATGCTCCTTTGAACTATGAACCGAGCATCATTTCCATATGTAGAAATCATGATTCATTATTTTGTCTTTTACCTGTAGACTGTTGTGTGCATTCCATATTTAGAAGGTATAGTTGAGTTTGGCATAACTGAGAGGGTATGTAACTTGTATTTAATCTCTTCTTGATTCAATCTTTCTCTATATTGCTAAGTTACTAACTTTCCGTTAAATATTATTCAAGGTTTTAGAAGAACAGAACATTATTCAGCGGATTAAATCTTTAATCTTTATAGCTCCGCCACAGAAGATTCATGAAATCCCTTTAGAAAGTTGTTCCGCTATGCTTGATCATGATCTTATCCACAACAATCTTAGCAGTATGCTTGAATATGATCAACCCCTAGTAAGAAAGCCACATAATTCCCCAAAAAATAGTGTTGGTGCATTTCAGCCACATGAACAAAGTTGGCAATTTGTAGACGATGATGGTGATGATGATGAAGATGAAGAAGAAGAAGAAGGCGAGGTTAGTTATTACCATAACAATTCCATGGGTTCGAGTGATTGTGTATCTCAAAACTTAGCTAGTCGCGCGGGTGATCTTTGGAGTGATGATGATAGTCGCTATCAGTGTGTTCTTTCCAAAATATTTAAGAACACCCAGAGATCGGCTATGGGCCCTGATTATAGAAACAGTGATTCTGAGAAATCCGCTTTCGTTAGCTGGAAAAATTATGATGGAATGGAATGGAAAGGAAGTTCTTCACAGATGTTGCTAAAGAATGTGCTTTATGAAGTCCCGAAAATGCACGAGAATCATTTAAGTCGGTACTATGACAAGAATGGAAATCTTGATCGGATGCAGGAAGTTGCGGTTGATGATGTGAACGACGTCAACCATAGGTTTTCGGTTCTAAGCTCCATCGTCCCTTCTAGAGGCAAGGTAAATTTCACACCCGTTTTGTCAGTTGAAAATGGGTGACTTGTGTTGACCCGAAACACTTTTTTCCAAGAAAATTTTAAAAAGAATAAAAAATATAGTACAATTCGGATTACTTTTGACCTATTTACGTATTAGCAATTTTAATAAATTTTGGCCATTTGATCAGTTAGAGATGTAACAGAACCTTTTGGGGTCAAAATGACTTTTGACACAAAATGATGATATTTTACTAGGTTGACAAGGTGTCTCTACTCGATGACACCATCAATTACTTAAAAACACTCGAGAGAAAGGTGGAAGTGTTACAATCCAGAAAGAAATCTCACGACGTTCGAGAAAGAACATCTGATAACTACGCCAACAAAAGAAAAGCTTCTTGTGCTCTTGAAGATATACAAGAAGAATGTTCTTCAGATTGTATTACAGTTAGCGCAATAGAAAAAGATGTTACAATCGAGATACGTTGCAAATGGCGAGAAAATATGATGGTGCAAGTATTTGATGCAATGAGCAGCCTAAACTTGGAATCCCACTCAGTTTGTTCTTCTACCGTTGACGGGATTCTCACATTAAGCATTGAAACTAAGGTATGTGACTAAAGAATCCATTAAAAGTACCATATTGCAATCATTATATAACGGTCTGTAAACTCTTACTGTGCAGTTAAAAAAATTTACGACGTCGACAGCAAAGATGATCAGGCAGGCACTTCAGAGAGTAATCGGTAGGTATTAAGTCGAGTTAGATTTAGAGGTTGATATATCATGTAGATTATAACCGCTGAGAACATCTCATTAGATGATTGTATTGTATTATGCAAACATTTATCATATGTTTAATTCAATAGTTGAATATGGAACACATAGTACAATGTCTGTACTATATGCATTTAAGATGGAGGTGTGCTACACCCACATAAAGTTTACAAAATTTCAGCCACTCGCTGACGTGGCAGAGTGCTGATTGGTTGGTTTTCTGTGGCCCACGTCAGCGAGTGGCTGAAATTTTGTAAACCTTAGGTGGATCTAGTATTTTCCTTTAAGATGATCAAGTATTGTGCTTGCCTGAAAGGTTATCTGATGTCCTTTTCATGTTAGATTTGTGAACTAAACAGTCATTTTAGATTCAATATAAATCAGTCATATTCTCTTGACATCGATCAAGTTTATTTGTTTATAACCACCTTTTGGGTTGTCTACCCCACGGGTTTCATTCAAAACTTGACCTACACATACAACAGATAAGTTTACCTCTTTTGGGTGAATGGACGGGAGAATCCGAAATGGCTCTTTCACGCAAGTTTAGACACTAACTGATTTGTCTGTACACTGTGTACAGCATGCAGGTTTGTTTGGTGAGGAAATTTAAGGGTGTCTTTATTGTACTTCCAAATCCTACAAATACATTCAAACCAAACATTTGAAAGGTCATTGTCCATGTTTTCCCAACCACTTAAAGTTTCTTGCAAGACAAATGCTACACTTAGATATTGACAACCCAAA

AaMyc-HLH2

CACAAAACTAAATTTGAAAAACATTTTTTTAACTAGAAGGTGAACCCAAAAGTTCTTCCTACTAATATTGCATATCATGCTTTCACCTCATACCCCTCTTTAAATAAAGATTTGTTTATTCATATTAAGTTCAAGATAATATACAACATTATTTCACATAATTATTTTGTGGGGTGCACAAACTTTGCAAAGTTGGTGCTGAAATGGAAAACTTGAGGCAAAGATTGGCTATGGCTGTCAAAAGCATCCAATGGAGCTATGCTATCTTTTGGTCAACCTCTTCAACAGAACAAGGGTACCTCTTTACCTTAATTTTTGATAATATATGTTCATGATATAAGCTTATTAGCACATGGTACACTTTGTTGACCAAGATATTAAGTGAACATATCTCCATGAATTTATATGTATTAGCAGTAGTGTCTTCCATCATAGTTATTTTATGTGTTTTGATTTTGCTAGCTTTAGGAATGCAAGAAAAAAGTTTTTTTAGTCGGTGGTTCAAACCCCGCGGATTAGTCGGCCAAAAGTTCAAAAACCACGGTTACCCAAAACAGAAAAATATTCATTCTAGTGGATGAAGTTAGTAAAGTTAAACTAAGTTTTGAAGAGCTGTTACATATGAGCTGTGATGTTAAAGAACTTTTGAGCTTCTTTTAACAATTTTTGTCCTTGAATACCTTTATGAGTTGTATCGTATTATCCGTACAATAAAGCAAGAATTATGAATAAGGAATGCATAATCAAATGTGAATAGAATTTGATTTAGGGTATTGACATGGTGTGATGGGTACTACAATGGAGATATCAAGACAAGGAAAACGATACAAGCAGAGGGAATGAACGAGGACGATGATGATGGTCAAGTGGGATTGCAAAGGACTGAGCAATTAAAACAACTTTATGAATCACTTTCAGCAGCCGAAACACATCATTATGAACCACAAGCAAGAAGGCCGTCGGCTGCATTATCCCCTGAAGATCTCACCGACACCGAGTGGTATTTCTTGGTTTGCATGACGTTTGAGTTCGGTTACGGTCAAGGGTAAGCAAAATCTAACTTCTTCTCATTCATACAATGTTATCTTGTTTATATTATGTTAAAACATGCAAGTGAGCTAACCTTATTAGAACTCGTTGTTCTAATAGATGATAATGTTTACGAACTTGTCGATCCCAAAAATAAACAACACCTATCAATCAAAGGTCGAATATGCGTTCTTTGACTTTCGAATAGCAAAATCAAGAAAACTGAGGGTATATAGAAGTTAAAACCTGTTATACATGCAATAGAAAGAAACAAATTTGACTTGGCCATATTGTAACTTATTCAAATTTAGATTGCCAGGAAGAACACTGGCCAAGAATACAACTAGTTGGTTGAGTGATGCCCACTTAGCCGATAGCAAGGTCTTCACTCGCTCCCTTCTAGCAAAAGTAAGCGTTACTTTTTTCTTTTTCTTTTTTACATTAATCTCGCTACTTTAACTTTGCAAAATTTTGTTATATTAATCTGAAATATTCGATCTAATTATCTCTCTTGTTCCGTCGCTACTGTCTTCCTCAGAGTGCATCAATTCAGGTAACTCATTATTTATGCTCCTTTGAACTATGAACCGAGCATCATTTCCATATGTAGAAATCATGATTCATTATTTTGTCTTTTACCTGTAGACTGTTGTGTGCATTCCATATTTAGAAGGTATAGTTGAGTTTGGCATAACTGAGAGGGTATGTAACTTGTATTTAATCTCTTCTTGATTCAATCTTTCTCTATATTGCTAAGTTACTAACTTTCCGTTAAATATTATTCAAGGTTTTAGAAGAACAGAACATTATTCAGCGGATTAAATCTTTAATCTTTATAGCTCCGCCACAGAAGATTCATGAAATCCCTTTAGAAAGTTGTTCCGCTATGCTTGATCATGATCTTATCCACAACAATCTTAGCAGTATGCTTGAATATGATCAACCCCTAGTAAGAAAGCCACATAATTCCCCAAAAAATAGTGTTGGTGCATTTCAGCCACATGAACAAAGTTGGCAATTTGTAGACGATGATGGTGATGATGATGAAGATGAAGAAGAAGAAGAAGGCGAGGTTAGTTATTACCATAACAATTCCATGGGTTCGAGTGATTGTGTATCTCAAAACTTAGCTAGTCGCGCGGGTGATCTTTGGAGTGATGATGATAGTCGCTATCAGTGTGTTCTTTCCAAAATATTTAAGAACACCCAGAGATCGGCTATGGGCCCTGATTATAGAAACAGTGATTCTGAGAAATCCGCTTTCGTTAGCTGGAAAAATTATGATGGAATGGAATGGAAAGGAAGTTCTTCACAGATGTTGCTAAAGAATGTGCTTTATGAAGTCCCGAAAATGCACGAGAATCATTTAAGTCGGTACTATGACAAGAATGGAAATCTTGATCGGATGCAGGAAGTTGCGGTTGATGATGTGAACGACGTCAACCATAGGTTTTCGGTTCTAAGCTCCATCGTCCCTTCTAGAGGCAAGGTAAATTTCACACCCGTTTTGTCAGTTGAAAATGGGTGACTTGTGTTGACCCGAAACACTTTTTTCCAAGAAAATTTTAAAAAGAATAAAAAATATAGTACAATTCGGATTACTTTTGACCTATTTACGTATTAGCAATTTTAATAAATTTTGGCCATTTGATCAGTTAGAGATGTAACAGAACCTTTTGGGGTCAAAATGACTTTTGACACAAAATGATGATATTTTACTAGGTTGACAAGGTGTCTCTACTCGATGACACCATCAATTACTTAAAAACACTCGAGAGAAAGGTGGAAGTGTTACAATCCAGAAAGAAATCTCACGACGTTCGAGAAAGAACATCTGATAACTACGCCAACAAAAGAAAAGCTTCTTGTGCTCTTGAAGATATACAAGAAGAATGTTCTTCAGATTGTATTACAGTTAGCGCAATAGAAAAAGATGTTACAATCGAGATACGTTGCAAATGGCGAGAAAATATGATGGTGCAAGTATTTGATGCAATGAGCAGCCTAAACTTGGAATCCCACTCAGTTTGTTCTTCTACCGTTGACGGGATTCTCACATTAAGCATTGAAACTAAGGTATGTGACTAAAGAATCCATTAAAAGTACCATATTGCAATCATTATATAACGGTCTGTAAACTCTTACTGTGCAGTTAAAAAAATTTACGACGTCGACAGCAAAGATGATCAGGCAGGCACTTCAGAGAGTAATCGGTAGGTATTAAGTCGAGTTAGATTTAGAGGTTGATATATCATGTAGATTATAACCGCTGAGAACATCTCATTAGATGATTGTATTGTATTATGCAAACATTTATCATATGTTTAATTCAATAGTTGAATATGGAACACATAGTACAATGTCTGTACTATATGCATTTAAGATGGAGGTGTGCTACACCCACATAAAGTTTACAAAATTTCAGCCACTCGCTGACGTGGCAGAGTGCTGATTGGTTGGTTTTCTGTGGCCCACGTCAGCGAGTGGCTGAAATTTTGTAAACCTTAGGTGGATCTAGTATTTTCCTTTAAGATGATCAAGTATTGTGCTTGCCTGAAAGGTTATCTGATGTCCTTTTCATGTTAGATTTGTGAACTAAACAGTCATTTTAGATTCAATATAAATCAGTCATATTCTCTTGACATCGATCAAGTTTATTTGTTTATAACCACCTTTTGGGTTGTCTACCCCACGGGTTTCATTCAAAACTTGACCTACACATACAACAGATAAGTTTACCTCTTTTGGGTGAATGGACGGGAGAATCCGAAATGGCTCTTTCACGCAAGTTTAGACACTAACTGATTTGTCTGTACACTGTGTACAGCATGCAGGTTTGTTTGGTGAGGAAATTTAAGGGTGTCTTTATTGTACTTCCAAATCCTACAAATACATTCAAACCAAACATTTGAAAGGTCATTGTCCATGTTTTCCCAACCACTTAAAGTTTCTTGCAAGACAAATGCTACACTTAGATATTGACAACCCAAATTTCTTGCAAAACCAAAAGAATCATTTAGGTCGGTACTACGACAAGAATGGAAATCTTGATCGGATGCGGGAAGTTGCGGTTGATGATGTGAACGACGTCAACCATAGGTTTTCGGTTCTAAGCTCCATCGTCCCTTCTAGAGGCAAGGTAAATTTCACACCCGTTTTGTGAGTAGAAAATGGGTGACTTGGGTTGACCCGAAACACTTTTTTCCAAGAAAATTTTAGAAAGAATAAAAAATATAGTACCCCTCGGATTACTTTTGACCCATTTACGTATTAGCAATTTTAATAAATTTTGGCCATTTGATCAGTTAGAGATGTAACATAACCTTTTGAGGTCAAAATGACTTTTGACACAAAATGATGATATTTTACTAGGTTGACAAGGTGTCTCTACTTGATGACACCATCAATTACTTAAAAACACTCGAGAGAAAGGTGGAAGTGTTACAATCCAGCAAGAAATCTCACGACGTTCGAGAAAGAACATCTGATAACTATGCCAACAAAAGAAAAGCTTCTTGTGCTCTTGAAGATATACAAGAAGAATGTTCTTCAGATTGTATTACAGTTAGCGCAATAGAAAAAGATGTAACAATCGAGATACGTTGCAAATGGCGAGAAAATATGATGGTGCAAGTATTTGATGCAATGAGCAGCCTAAACTTGGAATCCCACTCAGTTTGTTCTTCTACCGTTGACGGGATTCTCACATTAAGCATTGAAACTAAGGTATGTGAACTAAAGAATCCATTAAAAGTTACATATTGCAATCATTATATAACGGTCTGTAAACTCTTACTGTGCAGTTAAAAAATTTTACGACGTCGACAGCAAAGATGATCAGGCAGGCACTTCAGAGAGTAATTGGTAGGTATTAAGTCGAGTTAGATTTAGAGGTTGATATATCATGTAGATTATAACCGCTGAGAACATCTCGTTAGATGATTGTATTGTATTATGCAAACATTTATCATGTCTAATTCAATAGTTGAATATGGAACACAATATGGAACACACAGTACAATGTTGGTACTATATGCATTTAAGATGATCAAGTATTGTGCTTGCCTGAAAGGTTATCTGATGTCCTTTTCATGTTAGATTTGTGAACTAAACAGTCATTTTAGATTCAATATAAATCAGTCATAACTCATATTCTCTTGACATCGATCAAGTTTATTTGGCGTTTATAAC

AabHLH1

AAAAATAATAAAAATAAAATAAAATAAAATAAATAAAAAATAAATGAAAAAATTATCATCATAGATACAAAGTACTATTTTAACACTAGTCCTTTCATTCCACAAGAATCTATTCAAATATGTTAATGAAATTATAGCTAGAAAAGTATGAAATTTGCATAAAACATATTCTTCACCAACCAAATAAATTTGAACCACATATACACATCCCGAATGGAAAAAAAAAGTAAATGACTAAGCTAATTGAAAGAAAAAGAGTGAAAATGACCTTAATTAGACATATACTATATGCATACAGAAAAATCATTAAAACCATTTTAATAAAATCATCTTCTCTTCCACACACACACACACACTTTTCTCCATCATCTTTCTCTTAGTTATATGTGTGTATGGATCGATTTTCTTAGTCAAAACATAATTAAATTAGTGTGTACACACAATGGACCATCCATGGAAGAAGAAATCCTCAAACAAGTACCATGGATGAATTCCCATCTGTGACAAGATTATCATTTAATCCTTTTGATATCATCTTTCTTAATATATATACATGCCCATGTGTTTATGCATATTTATACACCAACCCTAATTAAAATCTCAACAGGGTTTCTGTAAGCAAAGTTTCTTGAACATGGACTCCATATTCGATCTTGAAGAAACAGAAAGATCTCGTTTGCTTCGGCAAATCATGGACTCCTTTGGTTTCACTTACATTTGCTTATGGTCTCACTTTACTCAGCCTTCCAAGTACATCTCTCTCATTCAAGTTCAATTCTTTATATATAGTATTCTTTGCCGTTCAAAAAGAAATGTTTAATCATCTTTGATACCCGTGCACAAATTGTAGTTGTTTGATCTGCATTGACGGGGTATACAAAGAAGGAAACAATCAAGCAAGTTCGTCATCTGGAAGTCTAACCATGACGTCTTTTCTTGACTACAAAAAGTTGATGTTCTTTATTGATAATTATACTGGGTAAGAACAACAAACTCATCGTTTTTAGTATAACGAGTATTACCAAAACTCAATCGTATGCGATAACAAGGGGTTTTAACTGAATTATTTTCTTATTAGAGGAGTTCCAGGGTTTGCATTCATGCACAATATTACATACATGGAGCGGAAAAAGTTGGAGCTCTTAACTTTGGCATCAAATTCTGCACAGCTACAATTTTATCAAGTAATTACACTTCTTATTAATTGTAAGTTTAGTTTCATTCATGTAAGAGTTGAAGCTAATGTACTGTATTCCTATTTTATCTTGCAGGAAGCAGGGATTAAGGTATCCAATATATGTACTTTAGCTCAAGATTCAGTCCATTAAACTCGCTTTATTTCACTTTTGTCATTTCTTTTTTAGCTAATTTTCGTTAAGGATCTTGTGAAATAGGCTAAAAAATAAATCGATCAAGTTGTAGTACTTTCTTTTTTGATTTATTGATCACACATATATACAATGATGATAATAAACTTCCATTACAGACTGCAATATTTATGGGGAGCAGCAATGGAGAGATTGAGCTTGGCATGACCAATGACTCCTCTCAAGTAAGAAATGTAATTCTCATCTTTCACATTGTGATCCCTTGTTCTTCGGTGTTTATGTTTATTTTAGATTATTTTAGTATTATATAATTCGTTTAAATGGTTTCGGGGTTTTTTTTTATTTAATTGTAGATAAACTTTGAAATCGAGTTGAAGAAGTTGTTTCCAGGTGATTTCCGGGAAGGTGTGCTTCTTCAACGACTTGAGGAAGCTCGGGCTTCATCTTCTTCTTCATCTTTAAGATCATTATCAATGGACAATAGCGTGGAGAACTCGCCATTTCTCTTCAACATGTTTCATTCCACTCCTTATATGTCCGAAATGTTTGCTCTCACAGAAGTACCACATATGGACCAACAAGCACCAAATCAAACACCACTGTCAACAACAATATTGAGACCAGAAGATCCTCTGCGGCAAGCTTTAGAACAAATTAGAACCTCTCAGTCACTACCTTCAAGAGAACACGAAGAAGCTGCAATGACAAGAGCAATTCTAGCTGCCATTACTTCTACTTCTTCTCCTACTCCTTCTTCTTCTTCTTCATTTCCATACCAGCGCCAACCCCCTCGAGTTGCTAGTGCTTTTAAGAGATATGGATCAAGTAATTTAGGACCTATTAAACAACGAGTTCCAAATCGACAAAATCTTCACAGAAGGTCACTTTCATTCTTACGAAATCTAAGTGAGGCTCGTGCTCAAAGAGATCAAATGGTCCAAACAACTAGACCTACTAGCAATCAACTTCATCACATGATAGCGGAGCGAAAGCGGCGAGAAAAACTGAATGAAAGCTTTCAAACTTTGAGATCATTGTTTCCTCCAGGGTCCAAGGTATGTCTAAGTTATATCATTGTTTTTATTTACAGGTGCTCTTCCTCTCTTATTGAACGTCAATTAAACAATCCTACATTTCATATCATACATATTAATCAATAATGAAACCCTAGTTTATAACTCAACATTATTATCCTATGTTTGATGAAAATCTAATTACATTTTATTTAAGAAAGATAATTTTCTACCACCTTTTTACTTTGATCTAAAAAAAATTACAATTTTTCTCAACCATAATCTTGATTCTTATATTTTGGTAGTTTACTAATCACAAAACTTATTAACTATCTTATTTTGATTAAATTGAGATGTCAAATCCAATAGAGCGAAATTTGTATTTTGCTTAAATTTTGAATGTCCATCTTCTTTAAAGTTTCTTTTAATTATTTTTCCATGGCTTATGATCACTTTAGAAGGACAAAGCATCAGTGTTATCAAACACAATGGAGTACATATCTTCTTTAAAGTCTCAAGTCGAGGAGCTTAACAAAAGAAATCAGATTTTGGAGGCTGATCAGCGTGCCAGGAAAGAACCACCTAATCAAGGTTCCAGTCGTTTTTCCGGAGAGGGTCCTGTAGTTGGCATCACTGACATTGGTGAATCAACTTCAGATTCACGAGTCGTTGACTTGGAAGTGAATGCAAGAGGGAATGTGATACTGGTGGATTTGGTGATGAGCGTGTTGGAGTTCATTAAACAGTCCGAGAATGTCAGTGTCATGTCTATAGATGCTGGGACTCGAATGTTGGAGACAGAAGCTATCGCAAATCGAGTAATTTTTAGATTAAGAATTCAGGTACCCATATCATTTTTCATTACAAACATGTATATCATTATTCAGTTTTAATGCATGCATTTTAATGACCTAATATGAATCGAGTTACAGATTCATGGATATACTAAAAAAAGTCATACTTTATTATTTCAACTTTATTAAACATCATCTAAGAAGCTTCTCATGATTCATGATAAACTAAAGATTAGAACAATGACATGAACATCAAGTTTCTAAGTTACACTAATAATCCAATATTTGTGGTTTCCTGGAACATGTTCATCAACTTATTTGACCTACCATAGTATAAAGCTATTTTTCAATTTCAATTTGTAGTTAAGTTTTATGGGTAGATAATGGTTTTCTCCTATAATAAGTTTATCATAGTTTTAGATAAGAGGTATTTTAAAAAAAATAAAATAAAATAAAATAACTTCAGTTTATCAATATATAAGTAGTATACAGATTTCTGAATGTTTGTTGATAAACGGTCAACAAAACAAAATGTTATTCTATAAAGGACAATAATAGAAAAGTATCCGTTAATTAAGTCTATACTTGCCTTTTATGTAGGGAAATGAATGGGACAGATCAAGTTTCGAAGAAGCAGTGAGGAGGCTTCTTGGTGACCTGGCACAATGACATGTTGTCATTTCTTTTTGACACTTGTGAATTTTGGACAACTTCAGAAAACCTAACAAGATATATTTAGGTTACGTTTGGTATGTTGGCACACAATGGGTATTGGTTGGATACAAGGTGGGGTCCAAAAGTATAAAAGTTTCATACATTCCTCTTTTGAGTACGCTGGAGTATGGAAGATATGTATATTTCAATCACATATTTGAAAGATAAACAACTTAACAAAACACTTGCATCATATCCGATGTGATTCCCTCATTCCATTCCCCCTTACCAAACGCACCCTTTCTATGTGCATTATTATCATCATTTACTTACTTGTAGAACAAGTGAATGATGCATATAAAGTACATATATAGCTGATTTACCTTTTATGAGTATTTATGTTGTTTGTGCAATATATTAGGTATCCGGCTTATTTGTGTAATTGAGTTTGTTTAGTTAGATGG

AabHLH2

GACCGCAGACTTCGTTTGACTCGACATTAATCTCTCATACATGAGCTGATCATCAAGATGTTGGAGACTCTCGTATGTTTATTCTCATGAATATTGGATTTAACTAGTATCATACGGAGTTATGATGTGCTGTGTATTAATCAATAAAGGAGTAAGTAAACTAATACGTCTACTGGGCCAGCGCGCGCGCCGCGCGCGCGAGAAGATGAAAATAAAATAAAATAAATAAATAAAAAAAATCCACGTATTTAATTTTAACCTGTTTAGCCGGTTGCCAATCATGCCACGTAAGCAAAAGTATGATAATGGAAAAAATTTATGAAGTTTGATGAGTATTCTTGTAATATTTTGGGTTGGTATCTGATAATGGCACAAGTGGGAAAGTATGTAGCTATTACATGTCATTAACCCTTAAAATATACAAAAGGCATGAATTCACAAAAAATACAATGTCGTTCATTGGTACATTGTCGTCGTCTACACATGAAGAGGTGGCACTGGAAAAAGGTTCATCTGGCGTGATCTCAGCTTCAGTAAGTATGGATCTGAAATAAGACGATTTCATCTGGCGTGATCTCAGCTTCAGTAAGTATGGATATACATTTGAACTTTGTGAAGTTTACCAAGTATGTCTTGAATAATGTGATCTTTCATGGGGAAAAAAACTAGAAGAAAATCATAAGTTTACAATAGGTGTTGAAGGGTATAAAGTACTCCGTATAAGATAGCAATATATATACAAATTGAAAAATTGAGTTGTATTAATACAAAGTTTACAATGGGGACAAAATAAAATAAAAACCCTATCAAAAATAATTAGGAGAGTTGAGTTTAATGATTGTATTGTTAATCTTTTATCCCCATAAGGTAAATTTTTTAAACGTTAAGTTTATTAGAAAAATCTAGCAAGGAGCTAGTTGAAGTAACAAGTTACACGGACTTCATACAAGAGTTTAATGGATAGATCGGATTATTTTTTAATTCCTTGCTAGAATTTAGAATCCAACTGTGGAAGAGGATTTTTATCTCTTCAGGTAAATTAGTATCTTTCCTTCGGCTACAATATATTTCCTTCGAAGATTCAAATCAAAACTAACATGGTTTTTATATCTCCAAATAATTAATCCAAGTCGTTGTTTGAATGATCACCGCGTCAAAGCAAGACTTGGACTTGTATATTAATTACGGTTAATCGAATTACTCCAAACTATGAGATTGAGGAGGTATTTTGGATAGTCAATAAGACCCACAACTCCTTAAGTTAATTATGATTACCTAATTAATCTTTTTTATATAAGTGTTATTTCAGTCTTGATAGTTAAACTTATTTTGTAATATGTTTTTCTTATGTTAGGAGTTCCTACCATTTAGTCCTTTTTTACTACAAATGAAAATTTAAAACAGTTGGAAAATCTGTTAGATGTTCATAGTGAGTGTATAACAAAATAAATATAAAAGCTACAGTATATATTGAGAATAAGGAGAAATATATATATGAATCTGCTGCCCACCCCCCTTGACTCTTCTTGGAAAATCTTGAAATGGTGACTTTTGACCAGTTACTACTACTTTTCTAATATTGGCTGGCTACCCATAACTGCTTTTTCTAAACCACCCACCCCAAAACAAAAACACAATGTCTTTATGATCACCATCACATCTTCCAAACTTATGACGTTCGTGAAAATTAATGAAGTTGTAATTTTTGCATCTAAGCTTCTGTTNAACAATATTGAATATTTGCAATGTAGCTTAATGGTCGAGTTATACGAGAAATGAATACGAATTTGTGGATAGATTGCGATATAGGCTATAGCTCAACGAAGAAATACATGTATATATACATAGTCTTCAGGTTTGGGAAAACTTGTTTCTGGGAGATGGATTATTTTTTCAGAGGTGTGAATGTCACTTCATCTTTGAGTCTTAAGACACTTTGAATATTAAGGAGTTTCCTTATGTAATTTAGTTAATTAACTAGTCTAAGAAAAGTAATGATTTAAATGGTTCAGTCTAGCTAGACAAAACTAGAACTATTAATAATTCCATCAGTTTAATTAGTTTTGATTAAAGTTTTCAAGACTTTAAGTCTTGCGTAGTTCCGTTTTACACCATTACAGATTCAAATGTTCTAGAAATTAAAGGGTATTTGAAAGTGTTCTAACACTAAAACTAAATTAACCTTTAATGCCAATAGACCAAGATAATATATATTAATACCAGAACTATTCTTACTAGATCAATGTTTAATTTATTATACTTTACCAAAAAAATTATTCACACCCCATAAGGTAAATTTTTTAAACGTTAAGTTTATTAGAAAAATCTAGCAAGGAGCTAGTTGAAGTAACAAGTTACACGGACTTCATACAAGAGTTTAATGGATAGATCGGATTATTTTTTAATTCCTTGCTAGAATTTAGAATCCAACTGTGGAAGAGGATTTTTATCTCTTCAGGTAAATTAGTATCTTTCCTTCGGCTACAATATATTTCCTTCGAAGATTCAAATCAAAACTAACATGGTTTTTATATCTCCAAATAATTAATCCAAGTCGTTGTTTGAATGATCACCGCGTCAAAGCAAGACTTGGACTTGTATATTAATTACGGTTAATCGAATTACTCCAAACTATGAGATTGAGGAGGTATTTTGGATAGTCAATAAGACCCACAACTCCTTAAGTTAATTATGATTACCTAATTAATCTTTTTTATATAAGTGTTATTTCAGTCTTGATAGTTAAACTTATTTTGTAATATGTTTTTCTTATGTTAGGAGTTCCTACCATTTAGTCCTTTTTTACTACAAATGAAAATTTAAAACAGTTGGAAAATCTGTTAGATGTTCATAGTGAGTGTATAACAAAATAAATATAAAAGCTACAGTATATATTGAGAATAAGGAGAAATATATATATGAATCTGCTGCCCACCCCCCTTGACTCTTCTTGGAAAATCTTGAAATGGAGACTTTTGACCAGTTACTACTACTTTTCTAATATTGGCTGGCTACCCATAACTGCTTTTTCTAAACCACCCACCCCAAAACAAAAACACAATGTCTTTATGATCACCATCACATCTTCCAAACTTATGACGTTCGTGAAAATTAATGAAGTTGTAATTTTTGCATCTAAGCTTCTGTTAGTAGTACTTATTATATATCGTAATGGCTATATTGATCTCTTTAATTTTTACGGTTTTCGCAACAAACCTAATGCATTATAATGTAACATTAATTTGTATTCAGCTCACTATCTTTTGTTAATGACCTTAGACTTATTCATTATTTTACTCTTAAGCGAAGTACGTATACCCATTTTGTACATGGTTTATACACAAATAGCATTGATAGTTTAGATTAAAGAAGTATAATTAGCATGTACACCCATTTTAAGTGTGTATTAAGGTTTCTTTCCAAATTGACAGAGACATATATATATTGCAATAAATATGATAAAGAGATACAAACTAAACAATGACATATAAGTCTAAATCAAATCATTTTCCCATTTCAAGCTATAAAAAAATAATAAAAATAAAATAAAATAAAATAAATAAAAAATAAATGAAAAAATTATCATCATAGATACAAAGTACTATTTTAACACTAGTCCTTTCATTCCACAAGAATCTATTCAAATATGTTCATGAAATTATAGCTAGAAAAGTATGAAATTTGCATAAAACATATTCTTCACCAACCAAATAAATTTGAACCACATATACACATCCCGAATGGAAAAAAAAGTAAATGACTAAGCTAATTGAAAGAAAAAAAGTGAAAATGACCTTAATTAGACATATACTATATACATACAGAAAAAACATTAAAACCATTTTAATAAAATCATCTTCTCTTCCACACACACACACACTTTTCTCCATCATCTTTCTCTCCCTTATATGTGTGTATGGATTTTCTTAGTCAATACGTAATTAAATTAGTGTTCACATAATGGACCATCCATGGAAGAAGAAATCCTCAAACAAGTACCATGGATGAATTCCCATCTGTGACAAGATTATCATTTAATCCTTTTGATATCATCCTTCTTAATATATATACATGCCCATGTGTTTATGCATATTTATACACCAACCCTAATTAAAATCTCAACAGGGTTTCTGTAAGCAAAGTTTCTTGAACATGGACTCCATATTCGATCTTGAAGAAACAGAAAGATCTCGTTTGCTTCGGCAAATCATGGACTCCTTTGGTTTCACTTACATTTGCTTATGGTCTCACTTTTCTCAGCCTTCCAAGTACATCTCTCTCATTCAAGTTCAATTCTTTATATATAGTATTCTTTGCCGTTCAAAAAGAAATGTTTAATCATCTTTGATACCCGTGCACAAATTGTAGTTGTTTGATCTGCATTGACGGGGTATACAAAGAAGGAAACAATCAAGCAAGTTCGTCATCTGGAAGTCTAACCATGACGTCTTTTCTTGACTACAAAAAGTTGATGTTCTTTATTGATAATTATACTGGGTAAGAACAATAAACTCATCGTTTTTAGTATAACGAGTATTACCAAAACTCAATCGTATGCGATAACAAGGGGTTTTAACTGAATTATTTTCTTATTAGAGGAGTTCCAGGATTTGCATTCATGCACAATATTACATACATGGAGCGGAAAAAGTTGGAGCTCTTAAATTTGGCATCAAATCCTGCACAGCTACAATTTTATCAAGTAATTACACTTCATATTAATTATAAGTTTAGTTTAATTCATGTAAGAGTAGAAGCTAATGTGTATTCCTATTTTATCTTGCAGGAAGCAGGGATTAAGGTATCTGAAATATGTAGTTATTTAGTTCAAGACTCAGTCCATTAAACTCGTTTTAGTTCACTTTTATCATTTCTTTTTTAGCTAATTTTCGTTAAGGATCTTGTGAAATAGGCTAAAAAATAAATCGATCAAGTTGTAGTACTTTCTTTTTTGATTTATTGATCACACATATATACAATGATTATAATAAACTTCCATTACAGACTGCAATATTTATGGGGAGCAGCAATGGAGAGATTGAGCTTGGCATGACCAATGACTCCTCTCAAGTAAGAAATGTAATTCTCATCTTTCACATTGTGATCCTTTGTTCTTCGGTTTTTATGTTTATTTTAGATTATTTTAGTATTATATAATTCGTTTAAATGGTTTCGGGGTTTTTTTTTTTATTTAATTGTAGATAAACTTTGAAATCGAGTTGAAGAAGTTGTTTCCAGGTGATTTCCGGGAAGGTGTGCTTCTTCAACGACTTGAGGAAGCTCGGGCTTCATCTTCTTCTTCATCTTTAAGATCATTATCAATGGACAATAGCGTGGAGAACTCGCCATTTCTCTTCAACATGTTTCATTCCACTCCTTATATGTCCGAAATGTTTGCTCTCACAGAACCACATATGGACCAACAAGCACCAAATCAAACACCACAGTCAACAACAATATTGAGACCAGAAGATCCTCTGCGGCAAGCTTTAGAACAAATTAGAACCTCTCAGTCACTACCTTCAAGAGAACACGAAGAAGCTGCAATGACAAGAGCAATTCTAGCTGCCATTACTTCTACTTCTTCTCCTACTCCTTCTTCTTCTTCTTCATTTCCATACCAGCGCCAACCCCCTCGAGTTGCTAGTGCTTTTAAGAGATATGGATCAAGCAATTTAGGACCTATTAAACAACGAGTTCCAAATCGACAAAATCTTCACAGAAGGTCACTTTCATTCTTACGAAATCTAAGTGAGGCTCGTGCTCAAAGAGATCAAATGGTCCAAACAACGAGACCTACTAGCAATCAACTTCATCACATGATAGCGGAGCGAAAGCGGCGAGAAAAACTGAATGAAAGCTTTCAAACTTTGAGATCATTGTTTCCTCCAGGGTCCAAGGTATGTCAAATTTATATCATTGTATTTATTTACAAGTGCTCTTCCTCTCTTATTGAACGTCAATTAAACAATCCTACATTTCATATCATACATATTAATCTATAATGAAACCCTAGTTTAGCATGAATGACCATTAAAATGTGTGATGCTAAACAAAAGCATTTGGACGTACTATATTTATAACTCAACATTATTATCCTATGTTTGATGAAAATCTAATTGCATTTTATTTAGGAAAGATAATTTTCTACCACCTTTTTACTTTGATCTTAAAAAAATTACAATTTTTCTCAACCATAATCTCGATTCTTATATTTTGAAAGTTTACTAATCATAAAACTTATTAACTATCTTATTTTGATTAAATTGAGATGTCAAATCCAATAGAGCGAAATTTGTATTTTGCTTAAATTTTGAATGTCCATCTTCTTTAAAGTTTCTTTTAATTATTTTTCCATGGCTTATGATCACTTTAGAAGGACAAAGCATCAGTGTTATCAAACACAATGGAGTACATATCTTCTTTAAAGTCTCAAGTCGAGGAGCTTAACAAAAGAAATCAGATTTTGGAGGCTGATCAGCGTGCCAGGAAAGAACCACCTAATCAAGGTTCCAGTCGTTTTTCCGGAGAGGGTCCTGTAGTTGGCATCACCGACATTGGTGAATCAACTTCAGATTCACGAGTCGTTGACTTGGAAGTGAATGCAAGAGGGAATGTGATACTGGTGGATTTGGTGATGAGCGTGTTGGAGTTCATTAAACAGTCCGAGAATGTCAGTGTCATGTCTATAGATGCTGGGACTCGAATGTTGGAGACAGAAGCTATCGCAAATCGAGTAATTTTTAGATTAAGAATTCAGGTACCCATATCATTTTTCATTACAAACATGTATATCATTATTCAGTTTTAATGCATGCATTTTAATGACCTAATATGAATCGAGTTACAGATTCATGGATATACTAAAAAAAGTCATACTTTATTATTTCAACTTTATTAAACATCATCTAAGAAGCTTCTCATGATTCATGATAAACTAAAGATTAGAACAATGACATGAACATCAAGTTTCTAAGTTACACTAATAATCCAATATTTGTGGTTTCCTGGAACATGTTCATCAACTTATTTGACCTACCATAGTATAAAGCTATTTTTCAATTTCAATTTGTAGTTAAGTTTTATGGGTAGATAATGGTTTTCTCCTATAATAAGTTTATCATAGTTTTAGATAAGAGGTATTTTAAAAAAAATAAAATAAAATAAAATAACTTCAGTTTATCAATATATAAGTAGTATACAGATTTCTGAATGTTTGTTGATAAACGGTCAACAAAACAAAATGTTATTCTATAAAGGACAATAATAGAAAAGTATCCGTTAATTAAGTCTATACTTGCCTTTTATGTAGGGAAATGAATGGGACAGATCAAGTTTCGAAGAAGCAGTGAGGAGGCTTCTTGGTGACCTGGCACAATGACATGTTGTCATTTCTTTTTAACACTTGTGAATTTTGGACAACTTCAGAAAACCTAACAAGATATATTTAGGTTACGTTTGGTATGTTGGCACACAATGGGTATTGGTTGGATACAAGGTGGGTCCAAAAGTATAAAAGTTTCATACATTCCTCTTTTGAGTACGCTGGAGTATAGAAGATATGTATATTTCAATCACATATTTGAAAGATAAACAACTTAACAAAACACTTGCATCATATCCGATGTGATTCCCTCATTCCATTCCCCCTTACCAAACGCACCTTTTCTATGTGCATTATTATCATCATTTACTTACTTGTAGAACAAGTGAATGATGCATATAAAGTACATATATAGCTGATTTACCTTTTATGAGTATTTATGTTGTTTGTGCAATATATTAGGTATCCGGCTTATTTGTGTAATTGAGTTTGTTTAGTTAGATGGAATGTAACGATGAAAGAATGGAATGAACATAGAATTGAATGAGTCATTCATTGTACATCTTGTTTGATTGTTATGTCGTGAGGGAATGAATAT

AabHLH3

ACCTGGGCTACCATAGTTAAAATCATGACACGTGTAAATAATCATTAGTATGTCATGTGATAAATTACATATATACCCTCTTATTGATTTGTTATTACATATACCCTCTCACCACCTCTTTTACTCTCGTACAATACCCAAATCACCACCTCTTTTACTTTTGTACCCAAGTTCTTTCATATTTATACTTGTTCTTTCATATTTACTCTTGTACACTATCTTCACCAAATCACCTCCTCTTTTATCACCACCTCTTTTACTCCAAATCACCTCCTCTTTTACTCTCGTACAGTACCCTCTCACCACCACCGGCCGGATTCCGGCCAAACGCCGGCACACCGCCACTCACCAAGCCTCACCGTCACCACCAATCCACTCCAAATCACCTCCTCTTTACTCTCGTACACACCACGGGCCAGCAAAGTCTCTCCTTCATCCCGATCTATATCCACTGCAGCCTACCACCATCACCACCACCATTTTATCACACATCTTTATAGATCTATACATTTATGGTAAGAAAACGCGTAAAAACGCGTGGGTTGACTCACCGCCAAGTCAAACCATTTTGTTTCTTGAATATAACAGGTTAAAAATTAATGGGATGGTGGGTTGAGTTGTTGTTGGTCTTTGGTATTTGTTTTTATCCGGCTATTAAATATGGGATGGTGGGTATATATTGTGTTTGGATGTACCTTATTTTTTGGATTTATTCGAGTTGGAGTCGCCGAAATCTGGGTTGGGGTCGTCGGAATCTGGGTTGGGTTGGTAGTGGTGTTGGACGGAGATTGTTTTCCGGAGAAGGTGAAGTAAATGCAGCTTTGCATCATAACTGCTTTTTTTAGTCTCATTTGAGGCGTTTGTTATTAAACAATTATAGTTCAAGAATATGAAGAGGACGAACTTAGCACAGCACGGACTCATAGAAATGATTTAAAAGATACTGCAGATTCTTGTTCTTCAAGGCTCGTAAAGAGCAGGATGGCTTTATCGTCATTTTCCCATAAGTCAAGTGGTAGTAGTGAAAGAAAACGTGAAAAGATGAGGAAGATGGTGAATACAATAAGAGAAATTGTGTCTAGTGGAAAGCAAATGAATTCAGTTCCTGTTATAGATGAGGTTGTTAAGTATCTCAAATCTCTAAAAATAGAACTGCAGGAAGTAGGTGTCGGAATTTGAAGAACTAAAGGATGCTGTGACCTAACTATATGAATAAGTGGATGTTCGTGAGATTGGTCGTGCGCGAATTTTTAGGACATGTTGATGCGCGTGGATTGCAACACGCACTACGTTTTACCTCTCGAGGGATCTCGAGAAGCTTGCAATCCCCACCAGGCAGTGTACCTGACCGATGTAGTATAGCTTAGTGGCAAGTACCGGGGGATTGATCGTTCTCTCACAGAGAATAGTGTTCGTTAGGTTAAAGACTACTCTAATTTAGGTCTAAGGGTGGAGGATGATTATTTTTTGTAATTTTTTATGTTTTTATGAAAGCAGTAAAATCTCGGTTTTGAAGGAGTGGGCGGAATTAGTTGCTAGTCAACTAACCCAAATATCTACTTATGCGAATTAAAATAAAGTTTTGAGAAAACAATGGTGGGGAAAGTAGTGTTTACTTAGATTTCATTACTCCCTAACTATCTTAACATCTTTTCCATGTAATTAAGTAATGTTGGTTTTGCGGGAATGAACGGCTGTATCCTACAAGGTCGAGGGAATGCGTTTCACCTAA

AabHLH4

TTGTCTTTTATAAATAATAGTATTTAGTAAAACAAACTTACTCATTTCTTGGTCTTAAGATTTTTTATTTATTCATTTTTTTTTATATATATTTTTTAAGTAGTAAAGTAAAGATAAAATATTTGTAGCAAGTAATTTAAAAAGAAGAAGATGCTCCCTCCTATTGTATAAACAGAAGAAGCAGCTATCAAATGCGCGTCACATAAAACCGTTTACTTTGATAATGACAAAAAAACACTGTTCTTCCGTCATCAAAAACATAAACATATTATTATTCCCTACCCAATTATTCATATCTTTTTATTCGTTTCAAAGCTACTACAATACTATCCAATCAAAAAATAAAAACAAAAATATTCTTTCTTCTTCTAGACACACATAATGGAAATTGATGAAAACTCAAACTGGCTTTTGGATTATGGATTGATGGATGATATTTCTACTGTTGTTGATTATGTTGCTCCACCTGCTGTTGTTGTTGGGTTTTCCTGGCCTTCTGACATACCTGCTGTCAGGTTAGTTTTGTCGAAATGTTTAACGAGTTGAGCTCGAGCTTATACGTATTACTGCGTAGTATAAAGTTTTTGGGCACATGTGATATGTCCTATTGGACTTGTTTTGTCTTTTGTGTGCAATAATATGGTGTGTACGTTTTTGACAATTTACCCTCGTAATATAAGGAAATTATTATTTTTTCACTGAAATCCAACAAAGGATGTGCAGGGTTTTTTTTTTTTTCACTTGTTTCAATTGGTTGTTCCACAGTTCTGAGATTGAAAGCTCATTTATCGACTTTGAAGGCCTTAAGGGGGCAGGATCACGGAAACGGTAAAAATCCATTCCTTTTTTTCTGTTTCTGGTATCAAATAGTCATTGATGAATCCTCATTAACACATCTGCAGTTGAATCTTTCAGGTTGAAGTCTGAATGCAGCAATGTATGTGGGTCCAAAGCTGGCCGTGAGAAACAGCGTAGAGATAGAATGAATGAGAGGCATGTACTTATTACTTGTTTTTCATGTAGTTATATCTTATCTTAAAGTTTAGAACTTTGACGTCAAAATTTAAAAAATATTTTAAAGGTTCATGGAATTGGGTTCGATTCTGGAGCATGGAAAACCACCAAAAACAGATAAGACTGCCATCCTGAGTGATGCTATTCGGATGATAATGCAACTACGAAGTGAAGCAGAGAGACTCAACGAGTCAAATGTTGATTTGCAAGAAAAAATCAAGGAATTAAAGGTGGGTCCATGATTTGGGTGTTTACGACACGGAATAGGTACATTAGATTTCAAAAGAAGGCCTCCCTCCGTCTCATGAAAAGTGCAACGTTTTCCACTTCGAAATGTGAATATGAACTTGAAAAAAATTGAATCTAGAACCTATATAGCTATCGGCATCATCACATAAGTGATGGTGTTGATTTTGGTCCCCTCACTTTTTCATTTAACTTATAGACAAACGCTTTGTCATGAATATACACTGAAAATGAGAGTAAATTTTTTATACATATGTTATATGGAACTTGTTACAACGTAGATATTTAATATTATATCTTTCTAGAGGTTTTCCATAGTTTTAGATGGTACACATTTATAGCTTGACTTTGACCTCAAAAGTCAAAGTAAGCGTCTCTTCATTCTTGTGATATTGCTATTGACTTATTATAAATATCGTGAACTCGGTTGATAGGCTGAGAAGAATGAACTGCGTGATGAAAAGCAGAGGCTAAAAGTCGAGAAAGAAAAGCTGGAACAACAAGTCAAAACCATGAACATTGGTCAACCAAGCTATCTGGCACATCCCACTGCAATGAGGGGTGCATTTGCTGCACAAGAACAAGCTGCTGGAAACAAGCTTATGCCTTTTGTCGGTTACCCCAGTGTTGCCA

AabHLH5

AAAAGTAGTAGTGAGAAATAGTAGCAGTGAATATTAGTTGTGAGTTGTGCGTTGTGAGTAGTAGCCGCAGCTCACGGCTCACTTAAAAAAGCATTAGAAAATTAGTCTTCTTAAAAATAGCCAACACTCACCGCTCTTTTGACAAACGACTAATAAAATTCAAAATGCTCATTGCTCACCTCTTATCACCAAAAAAATTCTACACCACTCCGCATCAAACATACGCTTTATCTTTACATGAAATTCAATAGGCAAAAAAGGGTAGACATAAGTAATTGTTATGTCCATCAATATAGTTTGTGCCAGTAATATGCAGTTATAAAAAATTTTAAATTTTGAATCCATTATTTTAACTTAAAAACAAAACCCTTTAAAATAATACACAAAACCCTATTATAAATACTCTTGACAAACGAAATTTGAAAAATGGGATGAGAGAAAAAGCTTGAGAAACATTCCAAACCAGACAAACATTGTTTGATTAATTTTTGCAAATTCAGAAATTAATCTTAACTTCACAATTTCCAAGGTAATTTAATATAAATGCATATATGTTTTTGTTCTCATTTATGAATATAGGGTTTACTGTGTGTATTTTCGTGTGTGTGTTTTTGTTTCAAGAAAATAATGTCACTCCAAAACGCCACAAACTCGTTCGATCCCGCTTCGTCTCACGATGATTTTCTCGATCAAATGCTTTCGGGCCTCCAATCTGGTGCTTCTTGGCCCGAAATATCAACCGGTGGTGGTGGTGGTAATGGTTGGGATGTAGATCAGTTTGATGATCAATCTAATTTTCTAGCAACAAAGCTTCGCCAACATCAGATCAGCTCTGGCCCGTCATCCGCTGCGAAATCGCTGATTCTCCAACAACAGATGATGATCTCTAGAGGCTTGGCTGGTGCTGGAGAGTTGGGGAATTTTCAAAATGATATGGTTGATGCTTCGTCTTTTAAATCTCCGGTAAATACTTTTTTCCGGAGATTTGTTAATAAACATAACAAAGTTATAGATTGTAATATGATTATTATGAAAAGTTATTGAATTATATATTATGTATAGAGAGGAGATAATTCGATTCAAACTCTTTTTAATGGGTTTGCCGGATCTCTCCAATCCCATCCATCCCAAGAGTTTCCATTTCCTCCGGTGAGTCGATTATTTTCGATTTACTTTGTGTTAAATAGTGTCTAGGTAGCTTGTGTTTTCATGATAAGTTGTATTGATGCGTTCAGTTTTAACAAGATCATGAACGCATCAAATACAAAAGCACACAAGTTAGACAAGCTTTTTTATGAAATGTTATATTAATTATGATTGATGTAGACCCAGAGCTTTGGATCTCCTGGGACCACATCAGCTTTGGTGAACCAAGGACAAACTAATGGTGGACCTCCCGCCACAACTGGTGGCGGGAGTGGACCACCTGCTCAGCCTAGGCAACGAGTCAGAGCTCGAAGAGGACAAGCAACCGATCCACATAGTATCGCGGAACGAGTAAATCAAAAACCCTAAATCACCCATTAATCTAACTACTTCTAAGTGATCTTAACATTCATTAAGTTTGATCATTTTTTTGACCGAAATTTGGCAGCTCCGGCGAGAAAGGATTGCCGAGAGGATGAAATCCCTTCAAGAACTCGTCCCAAATGCTAACAAGGTATGTAGATCACATCACGTGATACATAAGTGCTAATGATTATAAGCCAATAAAAAAGAGCGTGATTAGGCGCGTGAGATACTTGTTGTATGTGATTCCGTGGCATATACTTGAAACCGTAGATGGTAGGCAGAAAATCCGGGGTAGGTTACGGATCATAGTCGGATTTCCCGGGAAATTTTCTTAGTTACCTTTTTCACCCTGAGATTATCATATTTTTTGTCTACTTAATTATAGGAGATTGTAAAAATAGTAAACAAATTTACTAAATGATGTAAAGTGTAAAGTTATTTTAGTAACTTGATGTATCAAAGTTAAAGTGATGGTTACATTGAACAAATAATATGATTTTCGTGATCTAGAAAATATTTACCTAGTTAAAATGTAATTATAACTTACAAGGACTGTAAGATAGTTAGATCTTCTTTCTTATCAAAATAGAATATATACTAGTATAATAGATGATTGTTTAATCAAAAGTCAATGCATAAATAATTGTTTTATTATTTGACTTGAGTGACTTATTATTTGTAATTGTTGTTATTATTAAGATCTATTATTAAAATATTATAATGATGATTTAGGTATGTGTTGTGGAATTAATGACATATAACTTAGTCAAAGTAATTAACGTTTTCAACCTATTTATTCTCTCGATCTAAAAAGATATTTTTGATTATGAGTGTTATATATTGTGTAAATTAATAAAGCCACGGTTATATTTACAATTTCTGCTAACTATTTTTCCTACTTTCGCACATGTTGATGCTCTAACTTTTTAAAATATACATAAGATTTTCAGTTTTTAAACGATTATATTATATAATTAAATTTTTCTAGAAAAATCCCGTGCGTGCACGGGGATTATAACTAGTGTTATATATTAAAAAGGCTATATTCCAGTTTAAGTTATGTGTAGAAATAGAGACATTTCTTACTATGATGTACATACACTAACTCGTATTCTCGTTGTTTTCCAGGATTTCCCTCACATTCTCAAAATATTTAAGTTCCCTTACCACTAAACTACCTCAAAATTGTTTGAATTGTTATATTATCAATACAGATATGTTTTCATGTGATTGCTATTACAAGTCCAAGAAAATTTAGTAGAATGAACTCCAAGTATGCAAAACTCTTTTAAGAAGTGCGATTACGAATTTGATATCTTTTATAAGAATCATAGAATTATTAAAATGTATATATAATATTTTTTTATACACTTTGGGCGGTTAAAAGTAGTAGTGAGAAATAGTAGCAGTGAGCAGTAGTTGTGAGTTGTGAGTTGTAAGCAGTAGTCGCAGCTCACGGCACACTCAAAAAAGTATTAGAAAATTAGCATTCTTAAAAATCGCCAACACTCACCGCTTTTTTTACAAACGGCTACTAAAATTCAAAATGCTCATTGCTCACTTCTCTTCATCACAAAAAAATTCCACACCGCTCATCGTCAAACATACGCTTTCTGTTTACATGAAATTTCAATAGGCGAAAAATGGTAGACATAAGTTCTTGTGATGTCCATCAAAATAGTTTGTGCCAGTAATATGCGGTTATAACAAATTTTAAATTTTGAATCCATTATTTAAACTTAAAAACAAAACCCTTTAAAATAATACACAAAACCCTATTATAAATACTCTTGACAAATGAAATTTGAAAAATGGGATGAGAGAAAAGGCTTGAGAAACATTCCAAACCAGACAAACATTGTTTGATTAATTTTTGCAAATTCAGAAATTAATCTTAACTTCACAATTTCCAAGGTAATTTAATATAAATGCATATATGTTTTTGTTCTCATTTATGAATATAGGGTTTACTGTGTGTATTTTCGTGTGTGTGTTTTTGTTTCAAGAAAATAATGTCACTCCAAAACGCCACAAACTCGTTCGATCCCGCTTCGTCTCACGATGATTTCCTCGATCAAATGCTTTCGGGCCTCCAATCTGGTGCTTCTTGGCCCGAAATATCAACCGGTGGTGGTGGTGGTGGTGGTAATGGTTGGGATGTAGATCAGTTTGAAGATCAATCAAATTTTCTAGCAACAAAGCTTCGCCAACATCAGATCAGCTCTGGCCCGTCATCCGCTGCGAAATCGCTGATTCTCCAACAACAGATGATGATCTCTAGAGGCTTGGCTGGTGCTGGAGAGTTGGGAAATTTTCAAAATGATATGGTTGATGCTTCGTCTTTTAAATCTCCGGTAAATACTTTTTTTCCGGAGATTTGTTAATAAACATAACAAAGTTATAGATTGTAATATGATTATTATGAAAAGTTATTGAATTATATATTATGTATAGAGAGGAGACAATTCGATTCAAACTCTTTTTAATGGGTTTGCCGGATCTCTCCAATCCCATCAATCCCAAGAGTTTCCATTTCCTCCGGTGAGTCGATTATTTTTTATTTACTTTGTGCTAAATACTTTGTCTAGCTTGTGTTTTTATGATAAGTTGTATTGATGTGCTCAGTTTCAACAAGATAATGAACACATCAAATACAGACACAAGTTAGACAAGCTTTTTTATGAAATGTTATATTGATTATTATTGATGTAGACCCAGAGCTTTGGATCTCCTGGGACCGCATCGGCCTTGGTGAACCAAGGACAAACTAATGGTGGACCTCCCGCCACAACTGGTGGCGTGAGTGGACCACCTGCTCAGCCTAGACAACGAGTCAGAGCTCGAAGAGGACAAGCAACCGATCCACATAGTATCGCGGAACGAGTATATCAAAAACCCTTAATCACCCATTAATCTTACTACTTCTAAGTGATCTTAACATTCATTAAGTTTGATCATCTTTTTGACCGAAATTTGGCAGCTCCGACGAGAAAGGATTGCCGAGAGGATGAAATCCCTTCAAGAACTCGTCCCAAATGCTAACAAGGTATGTAGATCACATCACGTGATACATAAGTGCTAATGATTATAAGCCAATAAAAAAGAGCGTGATTAGGCGCGTGAGATACTTGTTGTATGTGATTCCGTGGCATATACTTGAAACCGTAGATGGTAGGCAGAAAATCCGGGGTAGGTTACGGATCATAGTCGGATTTCCCGGGAAATTTTCTTAGTTACCATTTGAACCCTGAGATTATCATATTTTTAGTCTACTTAATTATAGGAGATTGTAAAAATAGTAAACAAATTTACTAAATAATGTAAAGTGTAAAGTTATTTTAGTACCTTGATATATCAAAGTTAAAGTGATGGTTACTTTTAACAAAAATGATTTTCATGATCTAGAAAAGATTTACCAAGTTAAAATGTAATTATAACTTATAAGGGCTGTAAGATAGTTAGATCTTCTTTCTTATCAAAATAAAATATATATAATAGATGATTTTTTAATCAAAAGTCAATGCATAAATAATTGTTTTATTATTTGACTTGAGTGACTTATTATTTGTATTGTTGTTATTATTAAGATCTATTATTAAAATATTATAATGATGATTTAGGTATGTGTTGTGGAATTAATGACATATAACTTAGTCAAAGTAATTAACGTTTTCAACCTAATTATTCTCTCGATCTAAAAAGATATTTTTTATTATGAGTGGTATATATTGTGTAAATTAATAGTTAATAAGATATGTAATGTACTAAGTTGGATGCAATTAATACAAGAGTTTAAAAGGAATGAGATTGTTCATAATAAAATAGAGTAGGATAAGTAATTTGTTAATGAAAAGTCAGTGTACATGAAATTTGTTTTATTATACTTACTTTAGTGACTAAAGATTTATATTTTTGTTGTTATCATAATCTACAAATAAAGTAACTTTTTGCTAATGATCATTTTGGGATGGGATGTGGAATGACAGACAGACAAGGCTTCAATGCTAGATGAGATCATAGATTATGTCAAATTCCTCCAGCTTCAAGTCAAAGTACTATCTTTCTTAATTACTATATTTAACTTATTATTTGATATGTTAAATAATAGTTTATTCAATGTAACTTTGTAAAATGGTTGTTTGGATTAGGTCCTGAGCATGAGTAGATTAGGAGGTGCTGGTGCTGTTGCTCCTCTAGTTACTGATATCTCACCTGAGGTTAGATCTTGATCAATTTCTTAATTCACATATTGCGTTTATATTGTTTACTGAAATAAATTCAATATGTTAAAAAAGAATTTTCCAAATCTTGCAGGTTTCTATTACACCTACCCTTTCCATTGTCATACTATATTAAATTTTATTTTAATATATATCAAGATCAATATTTGCCTTTTATGCTCAAATAGAACAACAATAGACAAAAGAGCAACTATAATGTACAACTAATTGAAGTTGATAGCATGCTTTGGAGCCTCTTAATGTTTAGCTATTTAAACCATGCAACTACATTTTTTTATATACACTTTAATTTCAGACAACACCTTTCATAGACTTTAGAAAACAAGCAAACTTTTTTCTTTAATCTTAGTTGGTGTTGTTTTCTTTCCATTTTTGGTTTTTTATCATGACATATAGAGGTAGGTGCATTATAATATTCTTGCATCTAGGGTTCGATGTTAATCTTTCTATTAATAGAAACCCAAACAGCACAAATTAAGCTTAGAAACCAATCTTAATCACATCAAACCCCACACTCTTTTTCACAACAAATAAACGACACACAGAAAGAAACATCACGCGTGCTTTACTTTTCCAGATTCCACTACCGTCCCTTACCCCACCCCAGTAAATAACGATTTTACCCTTCATGCAAAATCTTGACGTAGCATGTTGTTTTATGAATATACACGAATGATGCGTCAAACGTTTTTGTCTGTAGTATCTTGATATACTATAGCTAATATATTGTTGCTTTAAAACTATGTAGGGAGCCCGTGACCGTGTGCAAGTAGCCAGTGGTGGTGTCGCAGGACGGACCACTAATGGAACAACGTCCTCTTCAAACAATGAAACAATGAGTATGGCCGAGAACCAAGTTGTGAAACTAATGGAAGAAGATATGGGATCAGCCATGCAATACTTGCAAGGAAAAGGCCTTTGTCTAATGCCGATTTCACTAGCTACTGCTATCTCCACCGCCACTTGCAACCCTTCTTCCGCTAGGAACAACCACCCACTACTTGGAGTTGCGGATGGTAATAGCCCTTCATCTCCCAATCTGTCCGCATTGACTGTGCAATCGGCGAATGGAGGATTGCAAACGGAAGGAGTTTCCATTAAAGATACTACCTCTATATCCAGTAAGCCTTAAACAAAACCGGCCGGCTTTGACTTCTGATTACGGACGTTATCATTTTCTCCGTGACGTTCGACTAAACTTGTTGGTTCTTTGCATTTTTTATACGAACTCACTTTTAAGTGCTTTTGGGGTGTCGTTTTGGATAGACTGTTTGTGCTCTTTAATATATTAATCTTAACTTAAAGCAAATAAAATATTAACCCTGTGACATGTATCATGTTTCTCAATTCGTAAAATTAACACTTTAAAAAAGAATAAATCGAATAAATCATCCATGTAATTTGGGCTAAATTGCAGATGCAATTCAAGCAACTCAAAAAGCATAGGCCGCAACTTATGTAGAGAACGTTGATGTGCAATTATCGTCCTTGACATGAGAAAAGATAAACTTATTAAAGTGAAGGCGCATTCTTCGGAAAAATGATAAATTCACACCAAAGTTTCTCACATGGATTGCGTCTGTATGTCACAGTTGCAGGAGCTGCTCATGCAATTTAGTTAAAGAAAACCTTTTTTCACACAAGCAAATGGCTATCGTTATTCATTATATGATTCTGGATTTTGTGACATAGGGGGTGTTTGGACATTAACAAATGGTTCAGGAACAGACAAATGGGGTGTTTGGGTACTGACAGAAGACGATGCAGAAGTAGAATCAAAGTCATCACATATATTTGAAGACTCAAAATGACAAGGAAAAATAGTAGGTAAAGGACTCATAAACTGTTGAATAATATCACTAGAATAGGGGAAAAAATATGTTCATGAGAAACAACATCTCTTGAAACAAATGATGAATGAGTAAGCACATAATAGAATTTATATCCTTTTTGATTGGAAGGATAACCTAAGAACACACAGGGAACACCTCTTGGATCAAATTTATTTGATGTTCTTGAAGGATTACTAACCACAGCAAAACATCCAAACATTCTCAAATGTTCATATACAAGCTTCTTTTTAGTAAGAAGTACCTTAAAAGGAGTCAAATTTCCAAGAACAGAAGAAGGAATCCTACCGATTAGATAAGTTTCAGTTGCAACACAATCACCCCAAAACCTAAGAGGCACATAGAATGAAATCTAAGAGCTCTTGTTGTATCTAAAATGTATATGTGCTTTCTTTCAACCCTGCCATTTTTGTTGTGGTCTGTCTACACAAAAGATC

AabHLH6

TAACGAGGTTGGGGTAGTACACCCCCACCTCATTTATGTCACAACTAACTACTCTGTAATTCATTAGCATTTACTTTGTCATTGTTACCCTCTTTCAGCATATACTTGTTCTCATGTCATAACTTGGTTTTGGGAAGAAACCTAAAACGAGATTATCGCGTGTTTTTCCCTTGCGTAGGTATTCGAGTGACGTTGCTTCATCTAGTCCGAACTACACGTGATTCACACGAATACGAAGTTTTACTTCATAGAACTATAACTGTGGGCCAACCTGCGCAAAGCCTACTCCATCTACTTATTAAGTCGAGATCCTTCTGTAAAGTTCTTCCAAAATTCCGATACATAAACTACTATGGTAGTATGGTGTTCATAACTTTTGTCTAACTATATGCGTGCTAAATATTGTGTAATAAATAATCAAAAGTCCAAAATGAATTTTCCAATGAAAAAAGCTACATGTTGCGATAGGATTCTGACCCACCACGACTACAATTGTTGGCAAGTAGTATTTAAGACGTTGCCCATGACTCTTTTATTCGATTCCTGCTACTTACAACCTTTTCTTTTCTCCCCACCTACTTATACACTACATACACAAAGTACTGTCTACAGTAGTAGACTAGCAGTAATGAATGTACAACAAATTTACATACAAAAGGGTCTTGCTATATTTCCTATATTCTAGTTTAAGTTAATTAAATGCGTATGTGAGGTCTGAAAAAATGGCCAACATGTTTGATAATATTTGTTCTTCTTCATATTCTCCTATGTCGCATGAGCCCAGTGACGATATTTCGGTTCTCCTACGTCAAATACTTTCTAAATCATCTTCTTCTTCTTCCTCGTCTTCGTTGCTGACAAAACAACAACAACAACAACAGCCGCATTCTGCCGATGTGGCTAATGTAGCGGTGAATGTTGGTGCTATTGATTATGATCATTCTGATGAATATGATTGTGAAAGCCAGGTACTCTTTTATACATTCCCTTATTTTGAGTTAAACTTTTGAATTTTTGACATTTGTGTAGTTTGTGACGATTTTAACCCTTGGTAATAGCGATAAGTAAAATAGTCATTATACATGAATAAAAATACAAATAAAGTCAAAATTCAAAAGTTTGAGTTTATAATAGCAAAATATAAAAAGTTAGCTACAAACACTCGAAAAAGTGACAAGTTTGATACTTTTTTCCAGATAAGTTTGATACTTTTTTCAAGATAAAAGTGCTTATATATAAGCCTAGCAAGAAACCTAACATTTACACTATATCGTCTTCAGTTACTCTACTAATGAAAAACAGGGGTGATTAAACATGTGACTGATTAAATAGTTAAATGACTAGCTAGCCAAATTATATTGGTTGACCAATTGGGACAAAATTTTCCCTTAATATGTCATCAAGTGACATATTCAATGGATCACATTATTTAGTGACTAATTCATGTGGTTTGAGTTCATCGTCGTATGATGAACTCTATAATCCATATAAATTAGTAGAGTAACAAATTTGAAAGAAAAAGTGTAACACATACCTTCACTTTTAGTTCATGTCAATACATTATGAAATAGTATACTCCTAGCTTTTTGAAGTAGTTAGATGCAGTAATATTTTTCTCAATTTTCTTAGTGAAAGGAATCAAAGGATGTTATTTTCGACATGTTTGTCCAAAAAGAAAAAAAGTTCAAAATTAAGTCTAGAATTTTTGTATTTCATATGTGATTGACAGATCTCACACGATCTACAATGAAAACAGAATGAAAGATAGTAGCAAATTTTTTTAAACAAACACACTTCAAAGTTCATAACTCACAAGTACTTTGTTTTGAATAATGAAACACATTTTTTTTTTTTTTTTTTTTGGTAGGAGGGGTTCGAAAACTTGATGGAAGAAATGGATGGAAAACCAAACCCTCCTCGTAACCCTTCAAAGCGAATGCGAGCTGCAGAGGTCCATAATATGTCCGAAAAGGTAAAAAAAAAAATGAGTCTCACAAGTACATTTTATAATTTTGTGCTCATGTTGATACTAATAATAATTGGATATGTTTTGGGTCAGAGGAGGAGGAGCAAGATAAATGAGAAAATGAAAGCCTTGCAAAAACTTATCCCTAATTCAAATAAGGTCTGTAACACAACACAAATACATTTCAATTATTACTATTTTGTTGCTCTGCAGAAAAAATTATTACACGTATGCACTGATACAAATACAAGTCTTGATTGTTTCGTTTTTTAGACGGATAAGGCTTCTATGCTGGATGAAGCCATTGAGTATCTAAAGCAACTTCAACTTCAAGTGCAGGTATATATGGTGTTCTTCTTGCAAATGCCATATTAATTATGGCTATATTAACGAAATTATGTGGCAATCAATCAGTTTCATACAACTCTATTTATAAAATTTAGCTAAAAAAAGGTGGGACTACAAATCCATATAACATATTTAAGTGTTCCAAACCTCTCTAGGTTATAAGAAAATAAGATTAGTTTAGTATTAGTTTCTCTAATCATACTTGAATTATGAATTTCTTACATTAAAGAGTTGGATTAACCCATCCCGAACCGATCTGTTTTCACATGTCTAAAAAAATACCTGTTTTAATTTGACGAATAGCTATTATTTACTGGGAACCACTAACATGCATGTTATTTAATGTTTCAGATGTTAACAATGAGGAATGGGATAAATTTGTACTCTATGTATGCACCACATGGTAGTGTTCAGCCGAACAATAGACCATCCAATGTGAACCAAGGAAACCATTCCATCAACATGGCACCGAACCAAGAAAGGCTTGTGAACCCGATGCTTGGCCAACCTATTCAATGTATGAGCCAGAACAAGCAATCAATACTTGACTTTTCGTGCACAATCAATCAAGAGCCTCCATTTGGAACTCAGCTTGGTTCGTCCTAGTTTCAAGTTCTTCAAGGGCGTTGATTGTGGGTGCAAAAACACTTTTTTAATCAGGTGTTATTCTCGTTGTTGTTGAACTTTTGGTTTTTAAGCTTTAGTTTCTTTTACAAGGTTTAATGTTATTGTCCATCAAATGTAATAGAATTGTTGGATGACTCGACAACTAGAATTGTTTAGACATGTTAGGATATCAATCAGACCCGTTGGGTCTGAATCCATTATGCGGCATAAGGATCAAGGAATGTGCAAAGCTCGAACGAAAATCAGAAATTCATAAAACCACTCCACATGAAAATTTATATGGTACCAAACCTTAATTCGTATACTTAATCAGGTTATCAGTTTTACTTACCGCAACGAATGATTCTCGGTTTGGTCCAGTTCAGGGTCGGTGTGGTTTGGCACTGGTCTGGTCCAAAATGATTTGTCTTAAATTCAACGAAACCACATCACACAATCGCTTAGACAAAAACTTATACTTACCATTGGCATAAAGTTGGAGTCTGGTTTGGTTCTAAAACTGGTATTATATGTCTCCCTCTCCATTTCAAGAGAGAAAAGTTCAACCTAAAATGTTCTTGAGTTGAAATGAAATGAAAGTAAAAGAAATAATGTCGCGTTCTTGAATTGGTAAAAAAAAAAAAAAAATGGAAAAGAAAAGAAAAAGAAAAAGAGACGCAATTACATTATGAGTAAATACTAATGGTTTTATTAATCAGTATATACTCAACTAGCTTATA

AabHLH7

AAACTTCATTTATGACATATTTATTAAGTTGACAGTGAAATGTAATATGTGAAAAACACGAAGGACTTGAAAAGAAATAAAAAACAATTAAGGCGGGAGAAAAGACACAAAGTTAGTTAAAACAACTGTTTAATCTCTTTTTAAAATTCAACAACCTTATAAAAACACACACACACACACACACACACAGAGACTCAAGTACAAAAAACACACATACCAAATCTAGACACAACAAACAACATAAAACTCAAAAACACATCTTTGCAAAAAAAGGAAATAATCCTCATTGTCATCATCTTCCATTACATTACATACATACATTATCTTGTACGTTTTCATATCAATCATTACTACTACACTTTAAAAAAAAGAAAAGAAAAGAAAAACTGTATTTGTTTGGTGAGTTCATTAATGGCGGATCTATACGGAAATGATCATCGTTCATTTTCATCATCTTCTTCACTTGAATCTGAAGATATGTCTTCTTTTCTACAAACTTTTATTAATAATAATAATGGTTCTTCAGCTAGTAACAAATATGGAGGGGGAGGACCTTTGATCCCTTCTCCTGTGCCTGAGTTTCATGATTCTGACATCCGGTTCTCCGATCTCAGCTCGTTTTATTCGCCCGAGCCGAATCAGGTCTCTTTTTTTGCATTTTTGTTGTGATTAGTAAAGAGATGGTTTAGTTTGATGTTTAGAGTTTTTTTATGTGGCAATTAATTGTTAATGTTATTTTAGTCCAATGGTACCTCAAGTTATGTGAGAATGCGCTCAACATGAGGTATCATTAGACTAAAATAATTAACTGTATTTCAAAAAACAAAGAAACAAAAAAAAGCTATAGTTTGTTAATTGTAGTTATTTATGTGGTAGTTAAGTTATATTTAAATGGGCTGGTACGCTAATTTGGTAATCATAGTGTAAAAATAAGTTTTACCACAAGGGACAAGAAAGAAAATAGCTAATTACACTATAACCAGGTGATCTTTGTGCATTTTGTTTGTTTGATATGAGATGATTTGGGGTTTCTATTTTTTGGTGTAATTGTTTTCGAGAGGGTCTAATTTGGTAATTACCCCGTATAGCTTTTCAATTAGTTTGGTGATTAAAGTGGAAATAATATGTTTTTACCAAATCGGACAAAAAAAGTTTATAGGATTAGGTGCTTGATAAGCGCAGAAAATGATTAATTACCAAAATAGTCCGTCTTCAAAACATTAGATACCGGCCCGGACATGATTAATTACATTAAAATTATACTGTAATTATTTAGAGAGGGTCATCTTGAAAAAGTATGTTGTTGTTTTGTGATATTTGCTCTTGTTGTTTTATGTGTATTATTTTGTTATTGATGGTTGATTGGGATTTAAAGGTGCAAAAAGTGTCGGATGTGCGTCAAAGTTCGAACCCTACTCGTTCCTCAAAGAGGACTAGAGCTGCTGAGATTCATAATTTGTCTGAGAAGGTCAGTGTTTTGTTTTTGAAAGATAAGGTCAAGTTGGAGACTATTTTGTTCAATGTATATTTGATGTTTATTCTGAAATATTGTATTCAGAGGAGGAGGAGTAGGATTAATGAGAAGCTGAAAGCATTGCAGACGTTGGTTCCGAACTCTAATAAGGTATAAAGGTCTAAATTTGAACATTTATATCAATTTTGTAGCAACAAATTGGTGGCTCTGTTTGAGTTTCGACAATTGAATTTCTCATTTTTTTTCTTTCTAAATAATGGTAGACTGATAAGGCTTCAATGTTGGATGAGGCAATTGAATATCTGAAGCAGCTTCAACTGAAAGTGCAGGTATATGTTTTTATTGTATTTCTATGTGTAGCACTTTAATCTTTTTAGCTTTCTGTTTTGTGATTTGTAAACAAGAAATTATTGTGTGTTTAGTTTGATGATAGTTATAGCTTTAGTATAGTAATATAGCTTATAGCAATAAACATGAGCCAAGAGAACTATATAGTTAATGTAAACATTATAAGAGATAGGAAGTGATATTGTTCCGGTTCATTGCATGTAAATCTTAATTTTTAGATACTATTAGATTGTTATATTTGTATGTGTGCTTCAGTGTTGTCACCGGAAAAAAAAGTTGCTATGAGTGCTGACGTGCTGTTGATGGATTTTGATGCAATATTTCTAGTTCTAGATTGGCTGCTGCTATAAGGCTTGGATAATTCGATGGCATGCTAGTAATTATCATATGCTACAACCACTCTGCCAAAGAATTATTATTTCCAGCAAATCGGAACGATCAATCTTGGAACACTCAACCAACTCAATTTGCCCATCAACTTGACAATAAAAATAGATCTTGTTCTCATGTGCAAGTCAGCTGATATAGTTCTATCTAGCTTTTACCTATATAACACTTGTCTGTTAATTTAACTTCTTTATTTTGATCAGTCATATTCAACATGCTTTGTTGCATTTGTCTTCTGTATTCCAAGTTCCTCTTAAAACAAGATAACACGATGGACATTGCTAAATAGACCAACACTCATCTGTCAAATTTATCAAACTCAGCAGTATAGAATAACCACAACTGCAATTTACTTCATCGCTTTTTTGTGGTTGCTCTTTCAGACACTTGCTATGAGAAATGGGTTTGGATTACAACCTGTATTCTCACAAGAACAAGAAATGCAAAGGGGAATATGCTACGATGAGGGATACAAATTTGTAAGTTCATCTGATGGGGCTGCTGGTACATCTTCTCAAACCCAAGGATTCTCAATGCGAAGGGAATTTGGTATGCCAAACCACCAAATCGGCATGATGATGAAACCAAATCCTGTTTTTGGTTCAGAACTTGCAAATGAGAATCAATATGGATTTACCAATCATTCCACGTCAGTGAAGGTAATATGGATGTTTAATTATTTATTTGGTATATTTGGCTTGATCCTAGAAACATCAGTTAACACAAGGTATTCCTCTGTATCAGGACACAAGCAGATCATTCTCAGGAAAAGGAGTCTCGTCATAGCCCCTTGAATGTTAGATTGAAGGAGTATATATGAGAAGACAGTTGGGCATGATTACTAGTATTGGCATTATTATAGTTGTTAAGATAAATCTGACCTTTGTTAGTATAATAATTAAGATTAAGATGATGGCATGTGTAACTTTAACGCATTTAAGTTCCTAACATAAGCATAGAGAAGAACTGTTTGGAGTTAATTGATTTGACATCCCTGGATTTATTCTTGATGCACTTTTTAGCAAACAATATGAAGTGTATGTGATCCCTTGTGGATTTATTCTTGATTATTTAGCCCAAACAAGACCAGGCTAAACAGCCCGAGCCTGATAGGAACAGTTTATGGCTTTACGAATTATTAATCTGATGTGATCTACGAGTAGTTTTCTGGTTGAAGCCTGGTAAAGATCAGGACCTGTTTAGGAACAAAGAAAGAACTATTCCAGACAGGGATGGGCTTTGTTTGGATTGAGATAAAGCTTGCGTGGTTTAAGTTAATTTTTTTCATGC

AabHLH8

GTTGCAACCAATGTTATTATAGATGTACTTATTAAAATTTGCCATTATAAGCAAGTGTTGAATCTTGATTTTTTAAGCTTTACATTTTAAGAATATCATAAATGCTTTATAATGTAGTATAGATACTCCAACAATAGTTTTTGAAGGTGATTGGCTATATAAGTACTCCCCTTGAAGAAAAAAACAGAGTGAGCAGAGAAAAGCAAGTGAAAAAACCACAAAAACAGGAAACCACTCTCAAAATCTGTGATTAATCAATCAAGACTCCATTTACAACCTTCTTAATTTTCTTCACATTTTCCCAGGTACATAAACGTATAAATCTACACATGTTTCAAATCACTACTTTCTTATAATATCATCTTACACTTATATTTAAAACTATCTCTGTGTTTTTTATAGAAAAAAAAAATGCAGGAATCACAAATGTCTCTTCAAGATCTCCAAAACAACGAAACCAGCAACAACAACAACACGTTTGATCCAACTGCCGCTCACGATGATTTCCTCGATCAAATGCTTTCTGGTCTCCAAACAAACGTATCTTGGCCAGACATTTCCAACGGTGGTGGTCAAAACAAACCGTCACTTCCATGGGACGTTGACCATTTTGACGATCAGTCTGCTTTCTTATCATCTAAGCTCCGTCAGCACCAGATCACCGGTGGTGCTCGTACTCCTGTCTCACCGGTATGTTCTTTTTGTTTGATGGGATTTTTTTTAATTATAACAGTGCATGTCGAAACCAGTACTAAAAGCTATCGAGGCCATTTTTCGTAAACTAAACAAGACATGTAACATAAATATTTTTGACATGGTATGTTTAAAATTTCTTACGTTATGTGGTTCGAAAATCATCCTAGGTTCGTCTGTGACGATATGTTTACTTTTTTTTGTAGATTGGAGAGAATAACAATAATAATGCTATTCAGAATTTGTTTCATGGGTTTACTGGATCTCTAGCAACGAATCAAGGCCAACAATTCCGTAATCTGCCGGTTAGTTTTTATTTGTTTATTTAATTTAATGCCATTTGTAAAAAAATTAAATTTTTTTTTGACTTTGATATTTAAAAAATGTATACTTTGCAGGCCCAAAATTTTGGATCTCCGGCAGCGGCAGTTGCTGCAGCGGTTATGAACCAAGTGCAGGCTGGAAGTGTGACCGCAGCTGGCGGCGGTGGTGGTGGTGCGCCATCGCAGCCACGACAAAGAGTTCGAGCCAGACGCGGACAAGCAACTGACCCACACAGCATTGCTGAAAGAGTAAGGATATAATTTTTGTTTTTAGCATAATCCTCGTATCATGTTATGTTGGAACATCCTATTATGTGAAAATCACGTTTTGTCAAAACAATAACAAAAAAGCCCAACTAGCAATTTTACGTGTAAGATTAGTAAATTTATTACTCCATCCGTCTTAAATTATATGTTTATCTTTATATATTTTAAAGTAAGTGTTTACTTGTATATTTCAAGCACTCAAAATGATTTTCAAACGTAAAAATAACACTTTATTGATGCGCAAGACTTCATTTTTTTTTAATTACGTGCAATTTGAAATTTTGGAGTGATTGAAGTATAAAATACAAATACAGTAAATAGTGTGGCAAGCCCTTAACTTTATTTATGTCAAAATTAGTTGTAGCTGTTAGCATTGTATCATGTTTAAAAAATCTTATTAATGTTGCTATTTCTTATAATATTTGTCAGTTACGACGTGAGAGGATTGCCGAGAGGATGAAAGCGCTACAAGAACTGGTCCCAAATGCTAACAAAGTAAGCGTTACATTAATTAAAAATGTTTAGTTTAAATCTTTTAATTAATTAATTGTTTATGGCGTTAGTTACGGTGGAAAAAGCAGATTATACGGGGAGAAGTTGTTAATAATGATAGTGTAGTTATTAACCTATTATGTTGGGAGGAAGTTATTTTAGTATTATCCTTAATCACTTTTTAATGGTAATTTAAGATCAAATAATCTTAGTTAATATTATTAAATGGTTTTATACATACTTTAAGATATCTTTAATCAATTATTAATTGTTGATTTAATTCCAAATAATTGTTCTTAAATGTTATTATATGGTTTCACACATACTCAAGATTTGCACATTCATTTTTTAGTATTAAAATCTTGTTAAGTATTACTAAAATATAACATGTTACAGGTCTGGACTTATGGCTAGATGGTTCTTTTTATATTTAGGTGGTGAATGACAAATTTCCATAACTTCCTGTTTAGTGATTAGAAGTGTACTTTTAGTCCTTTAGTATCTTGATCTATGAAGATATATTAGTTTTAATGTTAAAATAAAAGTGAATCTTGATTGAAAGAATATATAATAAATCTTTGATGTTTTGTTAATGGGGGTGTGGAATGACAGACAGACAAGGCCTCAATGCTTGATGAGATCATAGACTATGTCAAATTCCTCCAGCTCCAAGTCAAGGTACCTTTTCTCTAAACCATTTACATTAATACATAGCTATGGACATAAATAAAACCATTATGTTTGATAAATTTTGATCATAATTACTTTGAAACAAATTGTGATATATATTCTATGGCCACAGGTTTTGAGCATGAGCAGATTGGGAGGTGCCGGTGCTGCTGCAGGCGCGGCTAATCCCATGGCTGCTGAGGTACAAACATGTTCTCTAGCTATTTATCATGAGAAAAGTTGTTATTATCACACCTTATATCTTAGGGGGTGTTTGATAATGCTTATTTCTCAATGTCTTATTATTTTGAAAGTAACCATGCATCTAAGCTATAACAACAAATACTGAATTACCAATAGTATTGTTTCAACTGATCATGTACTGATTTGAACATTTGGTGGGCATGTCAATGTCATATAAGAAATACCTTTGTCAACGTGACATCGACTCATATCATATTGACATGGTCGGCCAACTAGTCAAACCACGATTAAAAAAAGATACCTATAACATCAGTGTTCAATTATGTTACCATCAATATCACTATTATTGACCTAATGTAAGACACTTTATTCATTTCTGAAAGCATTGCATTTTTAGTAAATATGTTTAGTATATGAGTACTTAAAAAAGAAAGATATCATATAAAATAACCTTAAAAAGACCCATCTTTATAAAAACATCACTCCTACTTTCTGTATAACAGAGAAGGACACAAAAAAAAAGTAGCACGCGAACATTACTTTCCTAGATTCCATCTTTTCTTATATTTACATTTTCTAACCTTGTACTTTTTGTTTTGTTTCCAGGGTGGTGGCGATTGTGGACAAAGAGGTGGACCGGGAACAGGACGGAGTAGCAACGGAACAACGTCATCATCTAACAATGAGACAATGACAGTAACGGAGAACCAAGTGGTGAAACTAATGGAAGAAGATATGGGTTCCGCAATGCAATATTTGCAAGGGAAAGGTCTTTGTCTTATGCCTATTTCACTTGCTACGGCTATTTCCACCGCTACTTGCCACCCTTCTGGCACCCGAACTAATCATTTGGCAGGTGGAGAGGGTGGACCAACGTCACCGAACATGTCTGTGTTGACTGTTCAGTCGGCGAATGGGGTGAAAGATTCACCTTAGTTGTGTTGATCCAACTTTTTGCTTTTGTGAAGAAAAAAAGATTGAGAAATTTGTTAACTTTTGCGAAAAGCGAGTGTTTTTGTCAAGTCTAACAAATATGAAGGAATGTTTTGAAATTAGTTGTAAGCATTGTACAAATGCTTTATCTGGTATGCCTTTTGATCTTTTATTGAATCTTTATGTTCGAGTTGTATAATGTAATCTTTTTCTTTTGACAAAACATGTAAGTTATAATGACTAAGGATAACTAGACGAATATCCTTGTAAAGCGGCGATGTATAACGACAAATAATTATGTTTGTGAACTTGTGGTTTTGGTATTTCTTTTGATGCAACATTTACGAGTTCAATTTATAGTAATTGAGAGAAAATAAACGAAAAATGATAAAAATTTGAGGATGAAACAATATTTAGAATATTAATTTAGTGGTAGATATCATTGTCATTTTTAATCATGGGTAATATAAAGTTTTCGCATTACTCAAATTTAAAAACT

AabHLH9

TAATGCAAGTTAGTAAGCGAGAACTTTTTAAAGTTGACACTAATCGGAGAAGTGAAAATCAATAACGTATGGCAATAGCCAATGGGAATGACTACTAGCATCAAGTACTTGTGGTTTGATGCAAGTGATAAATACCTAAACAAGTAACCTAACTTTCTACTACAAATATTTTTATTTTATAAAAATCATAACATAACATTTTTTATATATTATATTCCACTTTACAGTATCATCAACATTCACACACCCACTCCCATCAAACATCAATCATATATATCTCTTTGAACACGCCCACAGATTTTTCTCTGTTCTCCAAAATCTTCACTCCAAAACACAAAAAACACTGTTTGCATTTGTGTGTGTTTTTGTCAATAAAACATGTCATACAACATTTAATTTTGCATGTTTGAGCTTCATATGTTTTCTTTTTTATATAGAAAATAGCAAACATGTACAACACACAAGCAACTTCTTCTTTACCAGAAACAACAGACGACATTTCGTTGTTCCTACGTCAAATCCTTCTTAAATCATCATCCTTAAGTTCAGCAACAGCAAGCACAACAACAAAACCAACAATGTTGTGTTCTTCTGTTGTTGGTGCTAAGCAAATGCAATGTGAAATGCCACGTGTACAACCTGTTTATTCGTCTGGTTTAGTATCGGTTCCGGAGTGGGGTTGTTCTGATCTGGTTGGTGGTTATGCTCCGGTGACATATGTGTCTTCTTCTTCTGTGGGGACCTTGGATAATGAAGTTGATGAATTTGATTGTGAAAGTGAGGTATACTAGTAACATTTTTTTTTTATTGTTAAGTTTTATGAAATTTGTTTGTAAAGTTATTTAATGAGCCTTTTTGGTGAAATGGTAACGTTTATTCGAGATCTGTTCGAAAATAAAAAAACAAAAACCGAGAGGTAATGTATACACATACAAAAAATGACGTAATTATCACCTCTTAAAAAATATATGCATTATAATCATTGTGAAAGTGAGGTATACTAGTAACATTTTTTTTATTTTATTATTATTTTTTTTTTTAAATTTTTTATATTTTTGTATTGTTAAGTTTTATGAAATTTGTTGGTAAAGTTATTTCATGAGCCTTTTGGTGCAATGGTAACGTTTATTTGAGATCTGTTTGAAAAAAAACAAAAACAAAAACCGAGAGGTAATGTATAGACACAACTACATAGCAGACTAGGTGTACACATACAAAAAATGTCGTAATTATCACCTCTTAAAACATAATTATACTTGTACTTGGTAGCCAAAATCTTTCAAATATATACATATATGCTACATGTGGCTCGAAAATGCATTTTTCTTAAACCACTACACAAGTATATGTTGCATACATGTGAAGAAAAATGGGATGTTAAGTTAATGCACTATGTTTAAGTATATTTTGCATTAATGTGAACAAAAGTACCCGAAATAATATTTGACATAAACAAGTCAAGTTGGAGCTAGTATGCGGTTGAAGTTGAAAATTTGATAGTACTAGCATAATGTGCTTAAAAATATGTTCTAGGGAGTTTGAGATTCAAATGACATTAGTAAAACGAGGATTGATTATGCCAACAGCTTATCTGGATTTTATGGGTTAATTACTTGAAAATGTATCATAATTGCCACTTTTGTTATTGTGTATACTAAACTTTTTTTTTTTTTTTTTTTTTTTTTTTNTTTTCCTCATTGTGTGTACTAAACTTTCAAATTTCGATCATCGTGTGTATTTTATGACCATTTTACGCTTGCTTATTTCGAAATTTGAAAGTTTAGAACACACAATGAAAAAAAAAGTTCAGTACACACAATAACAAAAGGTGACAAGTATCATACATTTTCAAGTAATTAACCCGACTTTTTACCTATATACTTACAAGTTTAGCACTTTAGCTTAAGGATGAAGCTTAAAATCTTGATTTTGGATTGATGTGATTAGGAGGGTGGTTTTGAAAATTTGTTGGAAGATATGGCGACAAAATCAAACCAAACTCCTCGAAATCCTTCGAAACGAACTAGAGCTGCAGAGGTTCATAATATGTCAGAAAAGGTTTGCCTTGATCCATCTTCATTTTCTGCTTGCATTTGTGTTTGCGTTCGTAAATTTGATATTTATGGGTTGTGTTTCTTACAGAGAAGGAGAAGCCGCATAAATGAGAAAATGAAAGCGTTGCAGAAACTGATTCCTAATTCAAATAAGGTTAGCGATTTGCATATTCCTATTCGACAACTGATATGCCAAACATCACAGGAGAATAATTACACAGAATGATCGTCGGTGTCGTATAAATGGCCCTAGAAGTAACTTCATTTGTGTAAATTTCTTGTTATTGATTGGGGTTTTTGATTTGTGGTTTTGAATGTGACTAATAGACGGACAAGGCTTCGATGTTGGATGAAGCCATTGAATATCTGAAGCAGCTTCAGCTCCGAGTACAGGTACGCAATCAAAGTTGTCCGAAGTCTTTTTCTTGAAGTATTTAATCTCCTAACTAGTTTTATCTGATCGTTGTTCTTTGTAAACATTACTAACGTATTAACTATCTATTGAGTTATTTTAAAATGGACAAACGTGCAGCTGGTTAACCTGATCGACCCTTTTGGGTTCAACCTCAACCCGCCCATGTTGCCACATCTAATCTTAATATATCGTGTTAGTTATAAAGAATTTTTACACAGATTATGATTAAGGTTACCGTAAATTAACAATTTCATTAATATTCAGATGTTGACAATGAGGAATGGAATAAATTTATATTCCATGTCAGTACCACCTGGACTTCTACAGCCTCAACAGCTCCCATATTCACGAACCGGCTTCAATGAAGGAAATGATCCCCCAAACATGACCCGACTGAACCAGGAACCACACATGAATCCCATGCTCAACCTCCCAATTCAATCCACCAATCGAAGCCAACCATCAATCCCTAATCTTTCACATATAATGAATCAAGGGCCGTCATTGTCTTTCAGACCTCCACTTGGACCCTTTAAGGTGAGGTCTTAGTCTTACTTCATGTTTGATAAAAAGAAGCACAAAATCTGTACATATTCATGTTTCTGTGATTTGTAGGAATGCTTGATAAATAGCAGTGTATCTGATGGTACTCAACTGATGAATTCGGAGCAGAAATTGGTCCTTCCCGATAATTTAAACGGGTAAGCTTTTATTGGTATACATATAAAATGTCCGTAAGTCAGTAACATCCGCGGACCATGTTTTAAACCCTGTGTATTTGGCTTCTTGTTAGGGGTTTGCACCAGAAATGAATCTTAGGACGACGATGCAAAGAGCTAAAGTATGCCATTTCTCGCAATGGATTTGAAATTTCTTTAGCAGTTGGCGCTTTGACTTAGTATTGCTTCCATTGCATATGAAAGACTTGTGCATATACTTTGTTTTGTTATAATTTAAACCTTGAATGATGCTTAAGCTTTCATTATGGTTGACTTTTGGAAACTGTAATCTGACTGATAAAAGACCTTAGCTATATGGATCAAGGGTTATCTTTGGTAGCATGACCTTCTATTTATTGATTTATGGTTACCATATCTTGGTACAAAACTATAAATCCAATGAAGTAACAGAACTGATGCTCCCTATACTAAAATGCCTAGTAGGATTCAAGACCCAGCCTTGTGAAATGTATGTTTACTCGATATATTTAAGCGATTATGTACCCGAAGGCTGGCCAACTGTTAAATTTCT

AabHLH10

GATCTGGACTGACAATCCTATCTTTCATCACACACACACACACACACCCCTTTTTACACCCTCAAAAACACACACACATTATATATCCGAGGTTGATGTCGTTTGGTTTTATAAATCTATCTTCAAAGGTTTTCTAACTACTGAATTACGCGAAATTGTGCAAATTCAGATCGGTTTAAGGTATGTATATTGTTATATAACTGAATTTTGGTCTATGTGTTTGTATTGGTTCATTAACAAAAACAACCAGTTTAGAAAGAATTTTGTTTTTTATATTTTGTAATAGTTGAGTAGTAATGAATTGAGCATTCTATAAACTGAAAAAAAAAAACATTGACATCAGAAATAGTTGAATTATGAATTAAAAGTTGCTTACTTTGTTTTTTTTACAATAAGTTATCTAATTTTTAACTAACAAAAACAAGCCGTTAAAGAAGCAACGGGATTTTCTAGCGGCAAACAATTATACCTGTATTATTCTAATGTTTTGTTATGTATACAGATAGAGAGATTCTGATCAAGAATTACGCAGTGAATGCGCCTCAGAAAATTCGAGGCGCAATCAAATTCACGTCATCTACATGGACGTTGCTAGATCTGATTCTTAGTGACATTTCGGTTCCAATACGAGAATTTTGAAGATTTAGTTAGCCTAATTTTTCGCGGATCGAATTGTAGTACAATCTAGATGGAGCATCCTCAACAACGTTTGTTAGGTACTCAAGGTACACGCAATGTTTTTACCACCATCTTAATTAGTAGTAGTATGTCACACGTGCCCATAGTGTGTGTGTACGGATACACACAGTGAAGGTGTTTAACTAACTGTGTGTTGAAATACTGTAACAGGTAGAAAACCAACGCATGATTTTCTTTCGCTTTATTCACCTGCTCAGCAAGATCCATCTGCTGCTACTACTACTCCTTCAGGTAAATAATTATTCCATTTCTATTTTGTAACTATTAAATTTTACCGTTACATATGTATATACTTTGGTTATATATTTTCGGATACAATAGTTTTTTCGTAATTTAAAAAATACGGATAAAACGCTATTAGAGTCGTCGTGGATGAAGTTACGAAAACCCTAATCTAGTTTCGAAAAGTTTGACAATTCTCGGTTTGAAATTGATTTTTGTATTATGTTACATAGCGCGAAATCTTGCTAGTCATAATTTATGTTAATAGAAGAATTAGTATCATAAAATTGTTTGGAATTGGAAAAAGTATGTTTTTGTACATCTAAAAACATCCTAAAACAGATTTAAATTTTGATGGGACATGTTATAATATGTTTATTTATAGATAAAATAAAATTTCAAGACTTAAATTATGCTAAAAATAAAAGTTTATAATTTGATGGGACATTTCAAAATTGAGCTTGTTAAAAGATGGAGTGAACAAAACAATCAAAAGCCTTATCAGTTCTACAGTGTTTTCTGGATAAGCGTAGGTACATAGTTTATCATTCACGTACAAATTATTCTTTCATCATTTTTTTTTTTTTTTTTTTTGAACAGACTTTCATCATTTACTCACTACAAATAAATAATGTAAAAACTGTTAGTAGTAATCAAAAGAAGTAAGTAAAAAGTAAAAAATAGAAAAAGTGAGAAAAAGACTGAAAAGACAAAAGAAGAAAATGAAAGGACGACAAAAACGATAGTAGAAATGTCTTTGTTGGGATCGGTGTTAAAATTATCAATCCCATTTTATTCCACTACTGGATTCATGTTGCTCATCACGTGCTTTCATCCAGACACCTAGCCTATAAATATTTTATTCTTTAATATTTCATTTTTCAAAAAAATATTTATTCATTAAAAATTATTTTGAAAAGTTATTCTCTTACTTATAGTCTATATCGTTCTCTTGTTTATGCTACAACACAATTTCAATGAAAAAGAATATAAATTTTTCATATGAGGTACAACTTTTTATAATCATATTTAGGTACATTGCTAGGGACGGAATAGGACCAAATATTACCGCGTGCACAAATTTTTTTGAACCGTAATATTAGAAACAAATTTTGGTGGACCGGGTGCATTTAAGAAAAATCAATGTTTTTCAAAAATTGACACTACATCGAAAAAATGCATCGAGTTCCCGGGAACCCATGGTTCCGTCTTGTTACATGTCATGTTATAGCATACAGGCCCGTAAAATGTATTCATTTAATTTTGAGGAGTTAATCATTTTGCTGAGAATTACTTCAGACTGGAACCCATCTAAGCCTTGCATGTTGAGCATATATGCTAATTAAGGATCCTAGCAAAGTTAACTAAATTATGTTAAAAGAAATTGCGTATGGTCAAATCTAAAAACGCCTTATACATGATTGTGATTTTCTTATCACGTATAATGGTTTTCATTTTATGCTTTATGTGTTGAACATCTAATAATGTACTTGACTCTTGGCTGCCAATAGGTAGCTACCTCGAAACTCATGACTTCTTGCAACCATTGGAGCGGGTCGGGAAAAATGTTGCTAAAGAAGAAAATAAAGTTGAAGAATCATCCATCGGAAAGCCCCTACCACCAACTCCCCCAACTACAGTGGAGCATATTCTTCCAGGTGGGATCGGGACATATAGTATTAGTCACATTTCTGTAATCAATCAAAGCCAAAGGATGCCAAAGCCTGAAGGGGTCGGGGTCGTGATCACGGGTGCACAATCTAGTGGTAGTGATAAAAACGATGAAAATTCAAATTGTAGTTCGTACACCGGAAGTGGCTTTTCACTATGGGAAGAATCTGCAGTCAAGAAGGGAAAGACAGGGAAGGAGAATATTGCTGGAAATCGGCATGTGATTAGAGGTATGTTCATTGTAAGTTGTCTTTTGCTTTTGTTTTAAATAAATAAATTAGATGGTTAATAAACGATTTAACTTTCATATAACAGAAGGGGGCATGAAGATGGGAGGAATGCCATGGATGACATCTATGGAGCGGCCATCACAGTCGTCATCTGCCCATAACCATCCAACCGCAACCATCAGTTCTCTATCGTCTTCTCGGTCTGTTGGTTTTCTTTCCACTCTTGATTCTTGAAACTTCAGTTTTAGAAATGGCAATTCTGACCTTTCTAGTTCATGTGGCTTGATTTGGGTTGTGTTTTTATCACTATTGGGTTCAATAGGTAATAAAGAAGTTAGTTTAAAACTGATTGGGTCAAAAGTAAAAAAACGTGTACTTTTCATGCATAAATCCTCCTAAGATCTCTGGAATAAAAAAAGTGGGCAGAAGGAACATGACCGAGGATATATAAAATTTAGATGTTTTGATTTGAACCCATTTTGACCTATTACATGATCCGCATGCTCCGACCCATTTGTCAACTGTAATTTATGTATATCGTCAATTATGCCTATCTAACTTTTCTTTTTGGTGATTTTTCAGTCCATCATCAGCCCAGAAGAATCCGAGTTTTGTTGATATGTTAAAGTCTGCCAAGAGTGTGCAAGAAGACGAAGATGAAGAAGTAGAAGAGTTTGTTATCAAGAAAGAACCATCCACACACTACAAAGGTATCTCTCTTTTTCTCGCTATCTTCATTTTCACATAGTCTGAATCATATTCTCAGAATGTACATATTGTTCTGTAAATGATAGGTGTTTTGTCTGTTAAAGTTGACACAGCACATCATGATCAAAAGCCCAACACCCCGCGATCTAAGCATTCTGCAACAGAGCAGCGTAGAAGAAGCAAAATCAATGACAGGTACCTTTATATTAAAGTCGCAATATAGTCAACCTGTAATGAATTTTCCATACTGTTTCTTTTTCTTTTCATGGGCCAATGGAAATTTCTTTTGTATAAGCTATTTGAATCGATGAAAGTTGTGACAGTTGAGTTAAACTATGGTCATTGTCAAGACTAATGTATGATCTTTTCGTACATGTGCAGATTTTCAATGCTAAGAGAACTCATCCCTCACGGTGATCAAAAGAGAGATAAGGCCTCGTTTTTACTAGAGGTATATCGAGAGGGACATCTTCACTTTATTCTATGGTTCACAAAAAAAGGCCAAAACTTACATGTAGCTAAGCATAAAGTTTTGGTTTTTCAGGTTATTGAATACATTCAGTTTCTACAAGAGAAGGTACACAAGTTCGAGGACTCATGCCGGGGATGGCCTAATGAACAACCTGCAATGACCCCATGGGTAAATTCAAACTTTCAAGTATTTTTATGTTTCAAACACTGACTCTGAAAGAGTGAACTTTCTTATTAACTGTTTATGTGAATTAAAATTTTCAAATACAAATATGCATTAAAACATGGTATAGAAGATATGTCTGATTTTTTAATAATCGGATAACATATGTTGGAGTGTCTAGAAAATTCTGGTTATTTATAAGTTTAATAGGTAAAAATGTAAAATAAGCAAAAAGGACACTGTTAGAGAATAGGAGAGGTTTTATATAATATGACAGCTAAAATTTCTAAACATATGGTAAAAGTAAGGGGTAAATAAATGACATAAGCTTATCCTTTACTAAGCTTTTTTGTTGCGGCTGATATTTGTATATTCATACATCAAAAACTGTGTATTGTAGCTTAATGAGACAACCAGTTCAAAAACGCAAATCCAAACAGAAAAAGAGTTAACTGATAGATGTAACTTGGATGATTTCTTAGTCGTTTTAACTATACTTATCTCTCTTCATTTCAACAGAACAACAACCAAAGACCAACAGAAGGGTTTATTGATCAACCACGAGTCCAAAACGGTGTGTCTGGCTCAGCATTGCCATATGCTTCAAAACTGAACGAGAACAAGCACAGTGTTGCCTTAAACCTGCCCAAAAAAGATCACCAAAACATATTAGATTCTGATTTAAGTTCCCTCGAAACCATGAAGGACATTGGTCAACATCCTCGTTTGACCAATTTGGCATCTCCTTTCCCATCACCCTTGCAACCAAACGTGTATTCACCTGGTTGTGGAAGCACGAGTGTCGCTGCACCGCCTCCTTCAACTCTGCCATCTGATACAACAAACACACCATCCCAATTATGGCAAAGCAGATCATGCACAACTGACTGCACCGTTGCTGGTGATAAATCAAAAGACCAGGACCTGACGATTGAAAGTGGCACAATTAGTATATCAACCATCTACTCTCAAGGGTAAGACTTTATTAATTTGTTTTTGACAACTAATCTTATATGAATAGAAACTTGATCAATGCATTGTTTTTTGGTTTATTTGACAACTTGGTTTGCAAGCAGGTTGTTGAGTACGCTAACACAAGCACTACAGAGCTCGGGCGTGGACTTATCAGAAGCCAATATCTCAGTACAAATCGACCTTGGGAAAAGAGCAAATACTAGTAGACGCGATTCCTCAACACCCATTTTTAAGGTACTCACGTTACTTTATAAATCAATAGTTAAATAGCACTTGAGGTGCAAGAGGCCATGTCGGCTTAGGTAACGGGTCAGACGAGTCTGGTTGGTATTGGTGTAAACACTTTTTGGCATTTTTTTAATTCTTTGTGCATAAATTATGTGTTGATTCTAGTTTTTAATAAAACTATTATTTCAATGATGAGTTAAGAGGTTTTAAGCAATGCATAGACTTTTGATGACTTTCAGGCCGTTTGACATTCTGACCCATTTGACTTGTCCTCCTTAAAGCAAAGTTATTTCAGTTTGACCCATTTGAGATAGAACACGATTCACATCGGCCTATTGAGTAAATGGGTCGAATAGCCACCTATTTTATCAACATATTCTAAATCTTTTTGTGCTTGTTGCAAACAGAAAAACGAAGCTCCGGTCATAGATGAAAACCTGACACGTTCGCGACTTGCTAGCACAAGGGAGGAGAAGAACGACAATGATTTGAAGAGGTTTAAAACGAGCAGAAATTAAAGCTAATGGTTACTAGGACATACTCAAGTAATATTGGTATGATCACTACTCATTTTTCCACCAAGGGATCCTCTTTAAGTTTATTGAGGATCATTCTTGTTCGAGTACTCATATTCTAAGCTTTCTCCTACAATTCCTCAGCGGAGACTATGCAACGTGAGGTTGACTGTTGTAGGTGTATGAGTTCTTGCCAAGTGTTCATTAAGGGGACTCGTAGAAAGAAGCTTAGAAGGATATGACATTACTCTTGTTTTTATTTTATTTTTCTTTTGATACGATGTACATCTAGGGAGGGGGTTGCACAGAATTCTGACAAAATTGTATTATTGTTCATTCTTATCTCAATAGGTAGGCTTCCATTGTTATAATTCGAGAAGCCAAATTTATACGATTGAAGAAAATCTAGTCTAGTTATTTATTGGAACAGTCTACTGTTATTATGCTTTTTGCAGAACACAATGCTTCATGTACGTGTTTTTAACGTTTTTATATTGATCATTTTATGTAAGGCACCATTGACTGTCGAGTAATGTTAATGTTTTATCTATATTTCATATGGGCATACCTAAAAGGATAAAGCTATATAAAGCTTACAAGTCTTTGGTTTTCAGCAAATGCATTTGACAGTCATGACTCGCACCAACATGATCTTATTTGTAGCAGATTCAACGTAACTTTGATAGTATTTGGTGCTTTCAATATTAGTTCACAGCTCATATCCATCTTTAAGAAACTTCTGGTACTGGTTAGTGTCTATAGCTGATTTCAATTCAACATTAGAAAACATAATAGTAACATTCCTAGTGCTGACAATAATCTAATTTTGACACAGTATAAAACTTCTGGTGTTGTCAACATCATCATATCTATAAAGCTTCAATGAACCGAAGAAATACGAATTGCTATAAAGTAAGAACATTGAAACGAAAATGAAG

AabHLH11

CATAATTTCATTGAGCTATATTGAAATTTTCAGTTTATAAGCTATTTTGAGTGTTGATATATGATGAATCATATTGTTCCCGAATTCGAAACCGAGGAAGATTGCTTGATTCCGGATATTCATAAGAAATCAACAATGTAAAGTGCTTTCAAAGTTAGGTGATTATGTTTTTATTTGTGATCCAACAATATTTTTTTCATCTTGTAGGGGAGGAGATGAAGATATCATGGAGTTACTATGGCATAATGGCCAAGTTGTTATGCAAAGCCAAAACCAAAGATCTAGTGGGAATAAGAAGCCAGAAACAAGGCTTATGGTCCGGTCTATAGAACAACAACAACAACAACAGACAGGACCATCAGACTTGTTCATGCAAGAGGATGAGATGTCCTCCTGGCTTCATTACCCGGTAGAATATCCTGCTAACGAAAATTCATTAGAAGGATATTTGTACAATAACGATCTTTTGTTTCCAATACCACCACCAAATCCGGTTACTACAACGCCTATGGCGCCTACTACTTTACCGCCACCTCCTCCTCCTTCGGTTGTGATCTCTTCTCCTCGTCCACCAGTTGCGCCTATACGGCGCAACCATGTGGACATACAACCTAGACAACAACCGAAGTACCCTAACTTTTTGCACTTTTCTAGGCCTAATAAAGTCACAACTCTAGAGTCAGGTGCTTCAGCGCCAGTGACAGAAGCACCTGAATCTAGAGCTTCGCGTGTGTCAGAAAAACCACCGCCTACTTCTGTAGGCGGTGAGAGCGTTAGTGGTGTAGGTTTAGTAGGTACGTCATCTATGGGTAGGGAAGTTGAGACATGTGATACAAGCATGATGTCATCGCCCGATGGGTCGGGAGCAAGTGGAAGTATCGAGCCATCGACTCAAATGCCACCTCCTTTGACGAATGACCGTAAGCGCAAGGGTCGCGACACCGAAGACACCGAATGTCATAGTGAGGTACGCAAATTTTGTCTTAAATCTAGCTATGCGTGCTTGTGAACATGCATATAATTAAGTTTAGTCTTATTATCGCATATAGGTTAATTAGCAATTAATTAAGTACTTAAGCACACATGAGTTTGCCACTTTTAGCAGCCATTATATTCTTACGTGCATAGAGGGCAGAGCCGTATTCGAGGGTGTGCGAGGTGTGCTACCGCACAGGGCCTCAAAATTTTAGGGGCCTCAAAATATATATCCCTTTATATATTTTAGAGCGTCAAAGTATACTTCCAACTTCAAAATGTTAACGATATATGAAATTATGCTCTAAATATCATCTTAAAGTGGTCTAGAGTAACGAAGAACTCATTAAAACTTAAAGCTGGAAGGTTACAATAAATTTTAATCATAATGTTTAGGCCTCATTAAATTTTTTGCACAGGGCCTCCGCTAACTCAGCTACGGCACTGATAGAGGGATGTACCATTCATTGATTTTCGAATTTTACTGTCGTGACTCGATGTTTTAAATTTGTAACAGTGAATATGTGTTGGAAAAAGAAGAAAATCGAGACAAATAAGGGAAATATTGAGTTAGATTTAGTTTGTCCATAAATATCGCGTATTTAAAGCTTCTTGTATGTGTCGTTTAGTAGTAGTGCTTGAATGAGGGCCTCTTACACTAACTCCTAGACAAAATCTTGTTTATAAAAGCATCTTCTTGCGCCAAAGAGTATGCATGTATATATACCTGTAGTTTTACTATTCTATAAATACCAATTCTTAGTACCGGCAAACCCATGCACTATATATCTATGAAGAAACTGTATTTAACGAATTTCTTCAGTTAAGTACTATAAAAATATTGTGATAATAAAGAAGCTGAAGGTCAAACATCTTCACGGTTCTATATATAGCAAATTTTGAAGATATGCTACTTTTATTAAAGAGAATATCTACATCACTTTTTATGTGATGTTGTTTGATACTATAAGATATAAAATATGTGTTGCCAAATGACCCAAAAAATTCTTGAAAGGGATGATTCTATGGTTTGTTTTTGAAGCTTGAATTTGACGTTTGGAATGAATGTTCATTACAATATAGATTAGTGAGTTATGTATTCGATGGATATGAGTATAACTTGGGCGGGTCGAGTAATGCCAAGCACAAGCATGTCACTAGGGACGGTACCAGGGTGGGTTTTAGGTGACACTTGTGGATTTTACGATGTAGTGCTTATTTCTGTATTAAAAGAAAATAATAAATAACCATGATTTTCTTTCTAAATGCACATGATGCTGAAAATTTTTGTTCCTGACATTGTTTGTATATGTTTTCTTAAATGTATCTTGTCAAATTTTATCCTAATCGTGTACTTGCATGTCACCCTACTGACTATGCTCATAGGTTAGTCACCTTACCGGCTTTGCCCCTAGTATAAGGTCACAGAAACTTCTACAACATACATGTCAAACCAAACCTATCACACTTTCACGTTCTCTATGGCCAAATTTCCAAGTTCCAATGCATACGATATATATTTCTTTGTGAGAGTTGCCACATCACACTTGTTGTGAAGGTCATATATACTCATATATATAGGCCAGCTTGGCACGTGTATTTGCATACGTACGGACAAACAAGTTAACTTGATATCTTTTTGTGTACACTGAATAACAAGTCTTAAAACAAACAAACAAAAACCTGTTATAATAAAGTAGTGGTCATGAGTTAGGACACAGGAAAAGAGTTGAGCTACTCGTGGTTTCAGAAAATGTCTAGTGGCCCAAGTGGTGTGGGTATTTTATGTCTTTTTCCGACGTACTTTCTATTTTCTAACTAGGAACACATACGATGATGGTCAAGAATATATGGTCCCTTCGGTTGGGCAACGTACATCTTAGTGACTCGCCTTTTTTGGCTAACGAGAACAGGAAAGACCTCATTCGTTTATATATCTATTTTATGACTAGGGTGTAAAAATAGTCGAGATGAGTACAAGCCTAATTAGTTTGGCTAAACTTGAGCTAGACTCGAGAAATTGAAGAAGTAAGATAGAAGTTTAAGTCGGTTTGTACTTGTACATATTTTGACCAATATCTTGGAGCGATTTAAGTTGTAGAAGAGAGGTGTTTACTAAATTAAGTGATTAGATATAAAAATCTTAATGAGCCTCTTATAGAGAAAAGTTTATTTTAATGAGTCTTTCAGTTAAGAGACATTAAGTGTTTTGGGCCCACAATTCACTCCACTAAGAGAAAAGTAAACATCCCTTAATCTTTTCGTACTAATGCAAGTCTTTTTTAGATAGTGTTCTAATGCTTCATGCATACAAATTTTGACAATAGATATTAATGTCAAGGGGGGTCATGATACTCGCTTGCCACTTTTTCTTCATCATTTTCAATTTTGTGTTCCCCCCTTTGGGGTTTCAAATCAAATTGCTTTTGTGAGTGAGTTTACTATGTAAAATCAATGTATATGAGCTATGTTAAGACATTATTATTTGCCACAAGAAAACATGAAGATAAAGGCGTGATGGATTGAGGGACAAACATTTTACTTGTAACTTAAGGCATCATCATCAATCGCGGAACCAAAAAAGCATGGATAATGCTAGCAGAACTTATGGAGCTTTTAGTGGCCAAAAAGGATGATGGAATCATTATCATTTTGTTGTTATGATGGGATTGTACTAATTTAATCGGAAAAGATCATTAGAGGATCTACTTTAAATATAATGATTAGTTAGTTACTAAAGTGTTTCTTAAGGTTTAAAGAGTCGTTATAGGTTGATTACGTGGATAACCGAATAGGAGGGGGGAAAAGCATTATTAAGTTTTAACGCAATTGTTTTTGTAAGATTAGCGAAAAATTGGTGTTTTTCAAAATTTTAGTGACCTTCTTAAACATTATATGTTTGTCTTTGTTGCTTAATATATAAGATGATGGATAGTTATTACGGAGTAGTTCTGAATTCTAGATTTTTATATAATATGTGAAGGACGTCGAGTGTGAGTATCCTGAAGCCAAGAAACAATCTCATGGATCAACTTCTACAAAGAGGTCGCGTGCTGCAGAAGTCCACAATCTCTCGGAGAGGGTTTGTATTTTACTTCATTCAAAGAATTCAATTGCGGTAATACTCTTGTGGGTGGTCACTAAAATGTTGCAATTCTAAACTTTTTTAGAGACGTCGAGATAGGATTAATGAAAAGATGAAGGCCTTGCAAGAACTAATACCTCGCTGCAACAAGGTACTTAGTATAAATATTCTTTTTAAGTTGCAAATGAAATTATGAGTTTGTAGTTAGTAACTCTTATACGTCTTCTCAATTCTTTTAATTTATTTATTTGCAGTCCGACAAAGCTTCAATGTTGGATGAGGCAATTGAGTACTTGAAGTCGCTTCAAATGCAAGTTCAGGTATATAAACCTTAAATCTCTGAATGTTTCTTTACACCTACTAACTGTGACGCCTCGAGCAATAAACGAACCCAATAATCTTTGAATCAACTTAGTGAGCGAAACTCATATCAGAGTATATGGTAGTTGTGAATTCAAGTTGCACAATCAGTTTCTTAATGTTTGACATACATATAGAGTATTATTTTCATTATTGGATTATAAAGTAGGGGTCAAATAATCGGTTTTTGATTGTGATTGTATGATTATTAAAAGCGTCGAATAATCATTTTCGACTAAATGAGTTTCATACAAATGACAAGACTGTATATCTTAAGAGTAAAAAGTTACTTATTTGCCTATTATTTGACATAACTCGATATTTGGATGACTTTTTTTTAATCTCATTTCACGAACCACTTCTAATCTTTTCTATAGAAAACGCCATTTTATCTATAAATTCATAAATATATATAGGGCTAATCGTTTTGATGTCAGACGGTTTGATACATTAGTACTATTCAAATCAACTCTATACCTGAGTTTTTTAGAAATGACATGTATGATCGATTTTTGCAGATGATGTCCATGGGATGCGGCATGGTTCCTATGATGTTCCCAGGTGTCCAACAATATGTTTCACCAATGGCAATGGGGATGGGAATGGGTATGGGTATGGGCATGGACATGGGAATGAATCGACCCATGGTTCCTTATCCCGCCATTCTTCCAGGCCCATCAATGCCCAATCCAGCTGCATCTGCTGCAGCAGCCGCTGCTCAGCTTGGTCAACGGTTTCCTGTTCCAGGATTCAACATGTCACCCGTTGCCGTTGCGGGTCAAGCGGCTAATATGTCAGCTCCAATGATGAGTTCATTTCCGCTACAAAATCAAAATCAACCACGAGTTCCCAATTTTGCAGATCCATATCAACAATACCTTGGCCTCCATCAGACACAAGTACCGCTTCCACAGGCATGTTTATCTCTTACTAAACTCTAAAGATCCCCAAAAACATACACAATTAGATGCATATCGTTACTATCCATTGGATCTGCCTTTACTATCACGTATGGTTTTCTTGGTCTACCAACAGAATTTTGAAATTCCCTGGTTAGTATATATCAAAAACGCTGCATTCAGGTTATATACAATCTCTAAAGAATCAGAACTAATTCATTAGTTAATTAGTATAAAGTTTCTAAAAAGAAGACATGAAAGTTTTGGGAAGTCAACCCATCAAGAGTCACTTTCACTTGCAAAATAGTTGCTGTATTACTTTATACCATCAAAGCTCAATAGTTGTGGTTTCATTGCAGAACCAAGGAGGCATTCCCCCAACTGCAACTAAACCGGGCAGTAGCAAAGATGCTACCAACCCTGATCATCACCAAAATGGTAAGCCGAGGTCCCTGTATTTCAGTGCCTATTTATAAGCATGTCGACTATCGAGGATACCCATATAAGTAATAATTATCTTTGTGCATTTTCAGGTTGATTAGCAGGAGATTGTTCACGAAACAGGCTTATTAGCCGACCAAATTTTGTAGCGTAAAGGGATTTGAGGATTTAAAGGTTGTCAAGTCAACTACCCGCTGGCTTCATGTGTAACGTGTAATTGTATAAAGATAATATAGCGTTTTGTAGTTTTATTAGTTCTTCCTATAACGTTTACCGTTACAAGATATGTATCTCGCTAATTCCAAACAATCTTATAGTAGAATTCATGACTTATACGCTACAAATTTTTGGGCAGTGTATAGACTAGTAGAAAACAATCATTCATGATGGAATTAGGTTTGGTTTTACTTCCTATTCCTTTGATTTTTTGTCCCTTTTAGCATTTTACTGTACTATAGTTGATGGGTAGAAAATTTTGGG

AabHLH12

CCACAGCCTGTAAGAGGGCTAACGTTCAAATTTCAGCTTTGTTTCAAAGCGTGAAGTCTAAGAAAAATTAGCATGAGAAAGTGAGAAACATATGTTGGTTTAACATGTCTAATCAGTTGAGGAGTTATGAATTAGTTTATGATAACGTATCAATATTAAAGAAGCAAGTCCTGAAACACAAAAATGATTGAAAAACAACAAATTCCAAGAGAAAATGGTTACAGACTTATCGCCTGGCTAGACTCTTCTTCCTCCTCCATGAACGTGCTAAGTTTGAAAAGTATCGGCATAACTCATCAACACATTAAGCAAAGAAGTAAAAGCAAGCAACAAATAGTGAAATTCAAAAGAACTCAAGAAGGAGACAAAGAAAATATACGGAAACCTTTTACGTCTAGTATGTATTGAAGGTTTAGGATGATCGATCGTTTGATTTCTCGCATTCGGTAAGGACCCCAAAGTGTGACCGGAGTCACGAGTTAGGGTAACGCTCATGAGTCGACTCAGTTGGTCTGTGTCACTTACCCGAAAGGATAATCGACTTTGATGTAGAGCTGCCAGGGTAGGCCTGGCGTGTATTGATTGGGATAATCGACTTGGACAATCTCTCATTTGTTTATTTACGTGTAACGTGAACTGAGTATGAGGATACGAAAGACATGATTGACATGGTTAGTCGCCGACAAATATGTTTCAAACATATAATTATAATGTGATAATGGCATGTGAGTCTGTTTTAATGGCGTATGGGCCTGATTGCATTGGAGCCTGGTGGCGTTTGAGCCTGATGGCGTATGAGCCTGATGTCGTTTAAGCTTGCTGGCGTTTGAGCCTTGTTTTGTGTGATTTCTCTTTATCAAAGTGTTGTTTTGTTGTCAGGTTTATCATGTATGATTTGTACTTCAAAAGTTTAGAAGTGATTGCAATGAAAGTATTTTGTGTTTTGTCTTAGTAAATAGCCTCTTAGGTTGCGTTGTTGTTTTGAACGTGCAAGGAATAATATAAGAGTAACACGGACACTTTACATTAAGTAGCATTCACTTGAATGTTTTTACATTATATCCATGTTACAATCGTATTGAAGTTATTGAAAGAATAGAAATAGCAAATTTTGTAGCGTATACCTAAAGATAGCGTATCCTTGGTTTGGTCGCGTGCCAAGACAATGACCCCCTTGTCACGTTTGTACGTACGTTATAATCTTTCTCATTTTCCTAATCGTGCTTCGGTCTTTTCAATGATCATTCCATTTTTCCCTTTGCTTTTTCCTTTGCTGACTTTGAAGTCTTGGTCTGATTGATGACGTAATAGCCTGATGAAATAGCCTTTGAGCTTGACTGGATGTTCTTGACCGAGGGGTTGTGTTGTTTATGTGAGGGTTTTTTCTAGGGGAATGTTTGGTTATTTCTTTGTTCATCAAGTAATGAGCTTTAGCCTTTGATTGCTCTGTGTCATTCCGTGTCTTAATGAAGCCTCTTTGCGACACTAAATGTTTTGCTTAGGTTTTGTTTTTTTGTGTATAGGCTTGTTTGTCATGCCTAGGTGACTTTTGTCGTTTTTCTTGTGTCTTCAGGGAATGTCCAGGTTGAGGTGTATGTGTTAGTTGTTGTGATTTGTTTTGTTGTATGTGAGATTTTCGGGTTTATGTCATGGGATCAGGTTCCAAGGGCTAGTTTTGTGAGGCGTTTCAATGTGATCTCAGGCTCCGTATATATTTTTATGGGGTTTTTTTATAAGTTTTGTGTTTTCTGGGGGACAGAAAAGTTTTTTCAAAGAGTAGGAGGATGAGAGAGGTGTTGTATTTTTTTTTGAATTTTTTAGTGTGAGGAGGATTTTTTCATGTTTTTTAAACGAAAAACAGGTTTCGGCTTGTTGCTTGGAAACAAGTGTTTCATGGTGAAACCTTCGTTTTCTGGGTAAGGATGACTTTTCTGGCATCAGGAAGCAGAAACTGTCATTGTTTCGCTTGTTTTCCGGCGATGAATATATCGAAAACAAAGTTCTCAATTGTCGGAAAATCAAAAACGGTGTAAGTTTTAGTTTTTCCGGTGGAAACACTTTTTTTAATTAATAGAAAAAAGGAGGAAATTTTTGCTATCACTATAGTATAGCACGGACGGCGCCATTTTTACGTATGGTACGTATTGAGTTTAGGTTGGTCGATCGTTTGGTTTCTCGCATTCGGTAGGGACCCCAAAGTGTGACCAGAGTAACGAGTTGGGGCAACGTTCACAAGTCGACTCAGTTGGTTTGTGTCAGTTACCCAAGAGGACCATCAACTTGGATGTGGAACTGCCTGGGTAGGTCTGATGTGTATTGCTTGGGATTAGGCTAGACCATTTCGTGGAAATTCATATATTTATAGGATATCCTTTAGGGTTGTGAGAATCCGTAGAGGCAATCTGATGGTCCATGTGGTGGCCATCAAAGGAAGAAGTGAGGAGAGCACGTGGTGCTCGTTCTAACGGTCCTAGCGAGTTTTCCAAGCATACCATTCGCCTAGACACGTTTGTTAGACGAGCTTTGTTCTAGTGCTCCTTTTTGTTAGACTCTTACCTTGTTAGGCTCATGTTTGGGTTGAGTCTAAAATGTATGGAACTTAGCCAAGTGGGATGAAGGTCCTATACGGTATGAAGTCCCTCAATCCATTAGTGTATTTGACTGATTTCAGGGAAATGCGGTGATGGACTTATGCTAAAGAGACGAGGTCCCCTGGGATTCTCTTGATGTGTCTTTCTGGATCCAAGGAGGCTGACTTTTTTTTTTGCGCTGCCTGGATATTTGAGACATCAGGGTCTTTCGATGTGATGTTCGGAGAATACTTTCATTTCCAAGACTGCTGCATATTGAATGCATGTAAAGTTCTTAATGTTGATATCTGTCATGAGTTATTGTGAAGATTGAGTAGTTATCGGACTTTTCTTATCCCAGGCTTGAGCTGCTTCAAATTTTAGCGTGGCCTGGTAATTTGTTTTAGCTGTAAATAGTCTCTCTCCCAGTCATTTCGGAGTCTTCCTGACTGGGGATTGTGTTATTTTCCTCGATTTGTGTTCCAGGTTGTTCGATTAGCCGTTTCTAATGTCTTAAAGTCTGACATCTTGAAGTACGTTACTTTTTTTAACATAACTATGAGAACCAAAATGTGCTAAGGAGATATTTGTATGAATATATATTGTCGTCTACATAAACAGCAGACAGTTCAAAGACATCAAGTTCTACTAGCAGCTAGGAGGCTAAGTATATTTCATGAAGGAAGGTTCAAAAGGTAACACCTACCTATCATATGCAAATATCAACTGCAAAACTTTATTACAACATCGAGTCGTCGAAATTTGTTTCCCTTTATCAATTTCATTCGTCTGGGGGATTGTTGGAAAAACATGGTCAATTATTGGGTTTGAATGACAATTGATGTTCATCAAATATACTAATGGGGTTTTAGAGTAAGATTAATGTATGGTCATATGTAACGACCAATTTAATTCTATGTTCATGAGCCATTAGTAGGATTGATTGTGATAATTATTTGACCGTTTTATTTCATTTCTTTTCCCTATAAATGAGAGCTTTTCTCATATGTCTTTTACACCAAGAAATACACTCACTTCTCTCCTTTTGTTTAACAATTATCTTGATTCGTTGTGTATTGGTTCGTAATAGAGGGAATGCACATATTGGTTCACTTAGATAATCTGTTTGTGCTAACGGGGTTATCCGTTGTTGTTGTATCATGGAGATAGACGTTCATGAGCTTTCAAGCACCATGAAAAGAACGAATCTATCTTAAGGAAACTGTGAGCATCACATGGCTCAACATTTTCCTTGTTTGTGTTCTTCGGGTCTAAATAATCAAACGGTTTACGATACTATTTTCTACACTTTACATTTATCTCATTATTATCATGTTTGTCTCTTGTAGCAATACACACGTTTTATAACAAAAATTGGATTGCGCGACCGCACCTAATTTAAGTCACGAAATATAAGTTTAATTTCTTAAATGTCATAAAGTTAAAGTTGGTCCTCGTTTTAATGTTCTATAACACAATTCTCGAGTTGTCCATTTTGGTACATAGAATAGAAATCGACGGTTTTGACCACCTTTTGTTCAACGTAGTGTCAAAGGATGATAAAAGTTAAAGTTGTACACGAGTACAATGGTCACATCTTACGTAATCTTCAAAGTGTAACTTCTTCATAAACATTTGACAATTATAGCTTTTTTTGGAAGTTATTATTTATAAATTATAAAGTGTCATGTAAANTTTTTTTTTTTTTTTTTTTTTTTTTTTTTTTTTTTTATTGTTTTATTTTCTCTTTTCTTTCGTAAATATTTTTTAAGTTTATTGTATGCATCTTGGTAAGTATTAATTATAAAAGCATTGATTCAATACGTTATGTGTAATTAAACATGAGAAAGATGTTAGATGTTAGAACTTTAAGCTTTCATGTAATGAGCTTAGCAATTTATCTTACATCAACACTAAAACAGCGAGCACTTGTAAGTCATTCACTTTTAGTTTATGAATTTCATAGTAACTTTCGTCGTGATTTTCTATCAATATAAAACGCTATTGATTTGTTAAGCTACTTTTGAGTTAGAAATGTCTCACTGATAGCACTAACAATAATAAGTGATCAAATGCTATTTGTATTTAGATTACTCACCACCAAAAACGACTTTTAAGTATAAGCTTAAGATAACGGAAATAAACATTTACACTGTGTTCTCAAGTTGAGTAAAAGGGAGGAAAAAAAGTAAAAGTAATTTTAGTTGTGTTCTCCAATTTTTCTTAAAAAGTAAAGTGAGTGAAAGTAAATGAAAGTGGTTATACTTTAGTTATAAAACTTTCACTCCACTGCTTACTCTTTTTTACTTTACTTTCTCTTTCTTTCAATAGATCTTTTAATTAGTCAAATTACTTAAGGGTGTGTTTGGTATAATACTAGGAATTATAATCAATGGGTTTAGATATAAGGTGGACCCTTTAGGTATGAAAGTTTCATACCTTTACCCCTTTGAGTGGTTTGTATAGTAGTATAGGATATATTTATCTATCATTGCCATATTTAATTGCAAGATTTATCAAACAAACACCATTATTTAATGAATCTCATTCCATGATTCCCATAAACATGTACCAAACACACCCTTAATTTATTGTAATTGACATGAGTTATGAGATATAAAATCTTTGAGCCCCATATGACCTTGCATCTCCTCTCAGTACTCGTTGCACCAGTTATACGCACGTTATAGGGCTAATTTAGTTTCTATAACACGGGTAAACTGCACCGCGTATCATGTTATAGGTAAGTTACAGGCACAGAAGTTAATGTGCATAATGCACGGTTTTTGTGTGTGGTGCTTTGATTTTTCTAATTTTTTTTAATTTAAACTTTTATTTTATTTTATTTTATTTTATTCCTATAAAAATGTTATTAGGAAAGGTGTTTTTGAGGTCTTGTGTTAGAAAAATGTTGTAAAAATTGATGTTGCGTGCCTAATAACTTGAAGTGCACCTAGCACTTTTAATTTATATCACATACACAACCCACTTTCATATATTTTTGACACATCTTTTACCACTTACCTATTATATAAATCGGTAAAATGTGTACAGTGATACTCTATCATTTAAAAACACGAATGGAAGAAAGTATAAAGCCCAATACATAATGCAAATCAAGCTTAAAAGCTATTTTTTATCTGTTTCATTTATCTAGAAAGTACCAAGAACTATTTTTTTTTGTTCAAAAAAAAATAGTTCTTGGTACTTTCTAGATATATACTTTGTATAAGACAAGAGTAAACTATAGTTGTAAAATGCTTTGTCGTATACGATATTGTGGTAGTATAAGTTTGAGATTAGTGTAGTGCACATGTAGGTTTATTTTAGTTGTGAAATGTTAGAATGTTCCCCACGCCTTCGAGTAACTTTTAAAAGGCGAATGCATATCAAGTGGATTGATTTTCCCCATACAAAAATGATTCAAGAAAGTAATAATTTAGTACTACAAAATCTAATATATCACTTAAAAGTGATTTATTCTCTGTTACTATCAATAACACATATAAAGCGTGTTTGTGTGTTTGCAATTTTGTATAGTACGTGCCGAAAAGATATCATATACGCCCGTTTAGAAACTGGATACATACTACATAGTTTACAACATCCTCGTCATGTTTGATTACTAGACAATATTAGTATTTAGTAATAAGACAATGCCGATATATAGACTGCTTAACCTAAATCTATAATTTATTATTATTATGCTATTCAAGTTCATATTTCTTGTCGTGTCGAGTTATTGTTTCTTCTTTTTGCCAATATAAGAAAGTGAAATCGGCCCTCTAAGAGTTCACGCCGCTCGATCACACGATCAACATTCAAAAAGGTAGATTGTGCGACATCACGATAATGGACGTGGAGATTTATTTGAATATGTAACATCCACGTTAAAAATAGCTTGACCCCGTTGCTAGGACTTTTTGACCGGTATAAGTCATTAAAAGTCTGGTTTATGTCATCTTCGAGATATTTATGATGTTATCCGTAAGATAATAATAACGTAACACTCTTTTTTGCCTCACTTAAGCCATCCAAAAGTTAGTTCATGTCCCTTTTGTAGTCATTAAAAGTTTGCTTCAGATATCTTTTATATCATAATCAACATTATCGACCAATCAACATATTATCATGTCTAATAACCCTAAGTATAACGTATAGAATCATTTCATCGCGACACCGTAAAACACAAAAATATATAAAAAGAAAAAATCTATATACATCACACATTTTGCTAAATCTATTATTTATTCAGATTTTATCTCTTTTATACTCCGCCTTTATTACATAAAGCACATTGACACCAACATGTTTTATACTACTTCATCCGTCGTAAAACTAATGTCCATCTTTTATTTTTGTTGTCTTGTAATAAGTATTAATTTTGTGATTCAATCAAGCAAAAAATATTTTAATCGATAGTGATGATATTTGATTGATATATAATGCAAGTTAGTAAGTGAGAACTTTTAAAGTTGACGTAATTGGAGAAGTGAAAAACAATAACGGCAATAGCCAATGGGAATGACTACTAGGCTACTAGCATCAACTACTTGTTTGATGCAAGTGATAAATACCTAAACAAGTAACCTAGAGGTAAACTCTCACTCCATGAGGAAATGTGACATATTCGATAATATTCGTCTTTTTTCCTTTTCTTGGTTAAAATCTAGATGTAAACTTTGTAGTAGTTGGAATGATTGGCTACAATCTCCTTTGTAATTTTCTAGCGCCTTGCTAGTTGTTTTAATAAATATTTATTTTATAAAAATCATAACATAACATTTTTATACTATATTCCACTTTACAGTATCATCAACATTCACACACCCACTCCCATCAAACATCAATCATATATATCTCTTTGAACACGCCCACAGATTTTTCTCTGTTCTCCAAAATCTTCACTCCAAAACACAAAAAACACTGTTTGCATTTGTGTGTGTTTTTGTCAATAAAACATGTCATACAACATTTAATTGCATGTTTGAGCTTCATATGTTTTTTTTCATATATAGAAAATAGCAAACATGTACAACACACAAGCAACTTCTTCTTTACCAGAAACAACAGACGACATTTCGTTGTTCCTACGTCAAATCCTTCTTAAATCATCATCCTTAACTTCAGCAACATTTTTTTTTTTTTTTTTTTTTTTTTTTTTTTTTTTTTTTTTATATATTTTTGTATTGTTAAGTTTTATGAAATTTGTTGGTAAAGTTATTTAATGAGCCTTTTGGTGCAATGGTAACGTTTATTTGAGATCTGTTTGAAAATAAAAAACAAAAACCGAGAGGTAATGTATAGACACAACTACATAGCAGACTAGGTGTACACATACAAAAAATGACTTAATTATCACCTCTTAAAAAATATGCATTATAATTATACTAATTGTACTTAGTAGCCAAAATCTTTCAAATATATACATATACAGTATGCTAAATGTGGTTCGAAAATGCATTTTTTGAACTACTACACAAAAATATGTTGCATACATGTAAAGAAAAATGAGATGTTAAGTTAATGCACTATGTTTAAGTATATTTTGCATTAATGTGAACAGAAGTACTCGTAATAATATTTGACTTAAACAAGTCAAGTTGGAGGGAGCTAGTATATATAGTATGCGGTTGAAGTTGAAAATTTGGGACTAGCATAATGTGCTTAAAAATATGTTCTAGGGAGTTTCAGATTAAAATGACATTAGTAAAACGAGGATTGAATATGCAAACAGCTTATCCGGGCTTTTTATAGTCAGATAACTGGTCTTCAATTAGTTTGTGACGCTTATTCTATAACTATATATATAAGCTAGTGATATTCTTGGTAGATTTAAAAGTACTGTATGTACAAGTTTAGCTTGAGGATGATGCTTAAAATCTTGATTTTGGATTGATGGTGATTAGGAGGGTGGTTTTGAAAATTTGTTGGAAGATATGGCGACAAAATCAAACCAAACTCCTCGAAATCCTTCGAAACGAACTAGAGCTGCAGAGGTTCATAATATGTCAGAAAAGGTTTGCCCCCATCTTCATTTTCTGCTTGCATTTGTGTTTGTATTTGTGATTTTGATATTTACGGGTTGTGTTTCTTACAGAGAAGGAGAAGCCGCATAAATGAGAAAATGAAAGCGTTGCAGAAACTGATTCCTAATTCAAATAAGGTTAGTGATTTGCATATTCTTATTCAACAATTGAAATGCCTGAGAATAATTACACAGAATGATCATTTGTGTCGTATAAATGGCCCTAGAAGTAACCTCATTTGTGTAACTTTCTTGTTATGGATTGGGGTTTTGATTTGTGGTTTTGAATGTGACTAATAGACGGACAAGGCTTCGATGTTGGATGAAGCCATTGAATATCTGAAGCAGCTTCAGCTCCGAGTACAGGTACGCAATCAAAGTTGTCCGAAGTCTTTTTCTTGAAGTTTTTTTTTTTTTTTTTTTTTTTTGGAAAGGCAACAATATATATAAAACCTAGGAAGGAAAATACAATACGAGGCATACCCAAAAGGAACAAATACATACAAAAACTTGAACTAATGACATAGACACATAGGACCTTGACTAACCCACGAGCCAAATGAAGATGCACCTCTAAAATAAAATTGGTCATGGCCCACTTGAAATATTTAGGGTGCGTTTGGTATGCTGGCATGAATGACAATGGGTATGAGTTTGATAGTAGTGGTGGGTCCCTCACCCATGAAAGATCCATACATATGTCTCTTGATGTATATGGAGTATAGATTATATTTATCATTCATTACCATAGTGGTAGGCCCTCTCATCTTAAACAAACACCTTTAATTACTTTCATAGTCATAGTCTCCTAACTAGTTTTATCTGATCATTATTCTTTGTAAACATTACTAACGTATTAATTATCTATTAAGCTATTTTAAAATGGACAAATGAGCAGCTGGTTTCCCCGATCGACCCTTTTGGGTTCAACCTCAACCCACCCATGTTGCCACATCTAATCTTAATATATTGTGTTATATTATAAAGAAGTTTTATGCAGATTATGATTAAAGTTACCGGAACTTAACAATTTCATTAATATTCAGATGTTAACAATGAGGAATGGAATAAATTTATATTCCATGTCGGTACCACCTGGACTTCTACAGCCTCAACAGCTCCCATATTCACGACCCGGCTTCAATGAAGGAAATGAACTCCCAAACATGACCCGACTGAACCAAGAACCACACATGAATCCCATGCTCAACCTCCCAATTCAATCCACCAATCGAAGCCAACCATCAATCCCTAATCTTTCACATATAATGAATCAAGGGCCGTCATTGTCGTCTTTCAGACCTCCGCTTGGACCCTTTAAGGTGAGTTCTTAGTCTTATTTCATGTTTGATAAAAATAAAAGAACAAAATCTGTACATATTCATGTTTCTGTGAATTTGTAGGAATGCTTGATAAATAGCAGTGGATCTGATGGTACTCAACTGATGAATTCGGAGCAGAAATTGGTCCTTCCCGATAATCTAAACGGGTAAGCTTCTATTGGTATACATATAAAATGTCTATTTCAGTAACATCCGCGGACCATGTTTTAAACCCTCTGTATTTGGCTTCTTGTTAGGGGTTTGCACCAGAAATGAATCTTAGGACGACAATGCAAAGAGCTAAAGTATGCCATTTCCCGCAATGGATTTGAAATTTCTTTAGCAGTTGGCGCGTTGACTTAGTATTGGTTACATTGGCATATGAAAGACTTGTGCATATACTTTGTTTTGTTATAATTTAAACCTTGAATGATGCTTGAGCTTTCATTATGGTTGACTTTTGGTAACTGTAATCTGACTGCTAAAAGACCTTAGCTATATGGATCAAGGGTTGTCTTTGGTAGCATGTCCTTTG

AabHLH13

CAGTCAATTCCATAATTTACCTCATAATTCAGGTCATATCGATGAATTTCAGATTATAGGTATGCATCTGTTGACCTTTTCAGCTTTTTTATTATCACGATCTGTTTTTTTTTCGTTAGTTGAATAATTTATACCTAAATTGTGTAGAATTCTGTTATTGTTTGAAGTTAAACGTTCATGAGCTTAAAAGATAAAAGAAGTGAAAGAAATTTGAATGTGTTTTTAGCATAATTCTGTTATTTCAGAATTTTGATATTTCCTTAGTAACTGAATTTTGATATGAAAGTGATCGCATCAACAGAATTCTGTTAAAAGCTTGGATAATAGAACCAGAAAAAATTGAATGTATATTAATTTTGTTATCCCGAATTATGATTATTAAATTTTGATTATAAAATTTTTGTAGGCACGATCTACTTGAGGTCCTACTGGATCTGGACAGCTAATTTGAATCTACAAACTTCCACTTTAGTTTTCCCGCGATCTGAGACATAAAGAATTAGTTGTCAAATTTTAAATAAAAAGTTACGGAATCCGAATTTCGGTTTTCGGCGCGGAGATGGAGCTTTCGCAACCCCGTCCCCGCGGAGAGCCAGGTAAAATCAGTTAGTTTTTGTTTTAGTGTGGTGGCGCGTGGGCGCGTGTGTTAGTTTTGATGTGACGGTTGAATTTGCAGGTGCGAAACCCACGCATGACTTTCTTTCGCTTTATTCACCTGCTCACCAAGATCCGTCCCCTACCATTCCAGGTACCTTTTTAACCTTCTTTCTATTTTTATTGTTTAAATAACCACCCCATAATTCACACTTGGTAAATTTAAATTTTAGATAATTTATAGTATGAGTTTCTAGTGCTGTAATAATTTAAGCATCAATCAGACGTGAAAATTAATTACTTTAGATAAATTTGTTTTCTGGTTTAATTAACTAAAATATTTCTTTCTAAAATAATACGATAACATGTATTTTGTAATTTTTTTTTAAACTTATATTGAAGTCAACTAGTTGCCCTACTACTTAGACGTCGTAAGTTTCTCACAAAAGATCTTGGGTTCAAATCTACACATGTAAATATTTTGAGGATAACATAGAATGGAAACGACTCAAAAACAGTAACATGACACACAGTTACACTACATAAATCTGAGTCAAATAGTCGCCCTTAAATAAACTTAACGGAGAAAACAACACTTGGCTACTTTTCATAATAATGCAATCTGCATAGTAACATAAGGCGGGTGACAGTGGGACGAAAAAAATCGGTTGCCCAGCTTGGTCGACCGATATTCGAAATCGGTTGCCCCACTGTGGGCTTTGGTCATGAAAATCGGTCGTCGGAGGAATTTGCAATATGGGTCATGTAATCGACCGATTTTCAAACAAGAAAAACATTACCGTTTATTAAAAAAAAAAAAAAAAAAAAAAGAATATGGGTTACGACTGTGAAGAGAGAGGTGAATATGAGCTGAGGTGGCAGCCGGAAAGATGATGCAATATATGTCACGACTGTCACCAGCCTAACATGGCTACGGATAGTGATAATGTAAAGTGAATTACTCCTCTCTATTCACTCCTCTCTCATCATTACCTTCTGGTTAAGTTTGATATAAACGAAACAATTTTATCCTCGCAGGAAAACACTTTTTTGTATCTATCTTTCTTATTGTAGGAGCATAAATTTGTCAGAAACAAAATAACACTTGCACGAGTTGAAGTAAATGTTTGGATAACGCTGATATGACAACTCGATAAAAGTTGTTGTACTTATAGATATAAAAATAAGTACAATAATTGTGATACGTTCATTCTAATGTTATATTAGTAACTTTGAGTTATGTGATAATTAAAATGATGCATTCTTTTAACCCAAACAAGTGCACATGATATATGAGTGGTTGTCCCATGTCCGTCATGTCATATACGAAATTTCAATGGATTTAACATAATACATTAGTGGATTATCACAAGAAAACTTAGGAGCTTCCAACATGCAGTCTAGTTGGTTGCGCTGAGGCATATTAAAGTAACACAGGGCCTGCGAGTTGCCGCAGTTCAAGCTTCTGAAAAAAATTGTAGGATAAATGAACACTTGTGTGAAGGGTGTGAAACAGAGTTCAGTTTATACCAATGTGATTAGTATGTATTTTGTGTGAACTGATGTGCATCAGTCTTATTACTCCATGATGTTAGTTCGTATGTTTAATTTGAGGATGAAATTATTATAATAATTTGGTCACATGTATGACATATGATTATCCATCTTAGAAAAATGACCCAGGAATTATTAGGTAGTATCCGTGAACCCCATACTGGGTCCGGTTCACCAAATTGGTAGGCCCATACTATTTGGATTTTAGCCTTACTGACACAACCCCCACCCATCAAGGGGAAAGAGGGTGCAGGCTAAAAATGTTTGATGTTGTTGCTATTTGGTCTTGTTGTTAAAAGTTTTTAAAATTCTATAACTTCTCTGATTCATGATACATGGGTCCATTGTCCGTGCAACCTCCAAATGTTTATGTTATTGAATAGTTTTGTGGTTTTAATTTATTTCTATTCTATATAGACTTGCTATGTTTCTCTATATGCATGTTGCTAAAATTTAGAAACATGTTACGTAACGAGGTTATTGAGCCGCACGATGCTATGGTTATCCCATTTTTCATTCCGGGATAGTTATAACAAATCTAATATGTAAAGTATAAATAAATAAATAAATAAATAAATAAAATAAAAAATAAAAAATAATAAATACTCGTAAATTATAAGCTTTTACGTATAGTATTTAGAGTAAAGATGTAAAAGGTGTTACGTGTTAAAGCGATGTTTTAAACAATCCTTTTCAATAAAAAGAACAAGTATAGACACCAAATATTAAGCTAGATAGTTTTATATATGCGTCAAGACAACATATTCACATGATTCTAATGAAATTCCATATAATACTCGCTATAAAACCATGCAACTAATCTAAGTATTGTAATATTTATCGAACTACCCAACCTATTATATGCTCTTTTAATGTTATAAAACCATGCAACTAATCTGAGTATTGTAACATTTGGCTCATTTTGATTCTGATTTAAAGATCTTTTAAATAATAAACTTTTAAGGAAATAAAATTATTATTTTATTTAATATTTAAATTAAAATGACACTTATAATTTAATCATTTAATTAAACGACACGTTTATGTGTGGAAATTCTTTTGTAAAATTTGAGTGAGACTAGCATTTCTCTTAGTAAATAGATGAGGGTTTAAAAGCAAAACATTTTAAAGAAAACTACATAACTCACCTTAATATAAGGGGTTGTTTATAGAGATAGAGATATATAGAGAGAGAGAGAGAGAGAGAGAGAGAGAGAGAAAACTGTTTTTTTATTTCTAATGAATAGTACTCGGTTATTTTGTCACAGTTAGGCCTGATATATTTTTTCATGATTGTATCATATATAAATTTAAACACAAATATAAATTTGTTGATAAAGTTACTTTTTTTTTTAAACAATGGTTTCCGACTTTTTTGGCCGTCTAATCCACGGGGCTTAAACCCACCGTCACACAGACTCAAATCGGGTAAATCCCTCCCTTTTCAAGATGTTGGCGTGGCCACTTCAGGAAAAGTTACTTAATTTTTTATAGGTTTACCTTGATCTTGTGCACCCTTTTTTTGGTGCAGACCGACCCTTCCCTAAGGACCTAGCTCAGACAATAAGTGCCTATTCAAGTGACCTTTAGCCGAGAGTTGGGATTGAACTTATTACCTTTTTCTCTAAAGCCTTCATTGATCAGTCAACTAGACATTTGCGATGCACGAGTGATACCACTTGAGCAATACCCCAATGGTATTAAGGCTACTTGATTAAAATGTATTCTATGAGCAATACCCCAATGGTAAAAATAAAGTGAAAGTATAGAACTACTAATTATATAAATATGTGTTGTTTATAGCATTTTAACATGATCTTCTTATGTCCTTTATTACTTGAATACAGGATGTCTGAGTCCCTAGTTGACAAATCCAAAATAAGATTTTGTGACTTGGTAGCATACAAGGCTTAGGTTATAAATACCCCTAAATGGATAACAACACCCTTGCTAATTTTATGATGTCGACTGTTGCTATAATGACTTGCAGTCATTCAGAACTATGTAACTTCCACCTGTTTTTCGGGCATTTCTGTGATATTAAGCTTCTACCTTTTTAGTCATTTTTTTCAATGGCCATTGTCAAATTGTGTTTAGCACCTTCTAAGTTGAGTCATCATAAGAATAAAACCTCATTATATGTATCTTTTTTCAGGTAGTTACCTTAAAACGCACAACTTCTTGCAACCACTAGAACAAGTTGGAAAGACTGTTTGCGAAGAAGTGGAATACATTAAAAAGTCTTTCCCACCATCTCCACCAACTGTTGGGGAACACATTCTACCCGGTGGGATGGGAACTTACAGCATTAGTCACATTCCGCATATTAATCAAACCCAAAAGGTATCAAAGCCTGAAGGGATTGTGATATCGGCTGCACAGTCAAGTAGTAGCAATAACAATGATGAAAATTCAAACTGCAGTTCTTACACAGGGAGTGGTTTTACGTTGTGGGAAGAATCTAATGTAAACAAGGGAAAGACGAGGAAGGAGAATAATATTGCCGCAAATAGGCATACAATGAGAGGTAACAACTTGGTATGCAATATTAAATCTGATAGTAGCAAGAACAAATAGTTTATCTTGTTATATTTTTCAATTTCATATATAGAGAGGCAGAAATAGTTGTGGAGACTGAAGTTATGTTTTAAAATTGCTTTATAGATGGGGGTATGAAGTTTGGAGTTCCATGGATGACATCGATTGAGCAGCCATCAAAGTCATCATCCATAAATAATCATCCGAGCACAACGTTTAGTTCCCTCTCATCCTCTCAGTCAGTCATTGTCTCTTGGATTCCAGAAATTTTCTACCAACTGTTCATATTGTTCATTAGTGCCAAATCTTAAACAACATTGCTTTGTAAAATTGCAGTCGATCATCAGCCCCTAAGAGTCAGTACTTCGTCGATATGAAGTCTGGTAGGAATTTCCAAGAAGTCGAAGACATTAATGAAGGGTTTGTTATTAAGAAAGAACCATCCTACCACCATAAAGGTCCCTCTCTAAATTAACCTCCCCCTCTTTTTATTTTCTTTATTCAAAGATGATGTTCAAAACTGAGTTTGTTCAAATTCACCTCTAATGTGATAGGTGGTACGTCGACTAAAGCTGACACTTCAAATTCTGATAAGAAGCCTAACAGCCCACGCTCTAAGCACTCTGCTACAGAACAGCGTAGAAGAAGCAAAATTAATGACAGGCATGTTTCCTAATAGTAAATTTCTGTTTATGTGGTATATGAGCACTTTACTTACGGTGCTATTTACCTCTTAAATCTAGCTATTAATTTTCAGTGGTTTGATGATTTTGTGTAGCATATAAATCACAATGCTTCATCATTGCATAGACATTGTTTTGAGAAACTAATAGATCTCATTCATATTATAAACCAGGGAAGATTAGAATTGGTTCCTTGTAATTCTAAACATCAGAGAATTAAAGATGTTATTTTGACCCATTCACTTATATTAGTGGGTCAATACGGGTGGGGTTTTATCTCAAATGGGTCAAATAAAGAAATTAGCTAAGAGCCTAAGAAGGTATCAAATGGGTTGAAAGTCAGCCAAAGTTTGTTTTCGGTGCATACAAGTATACAACCTCTTGAGTTTTATTCAAAGATTAGATTATTGTTCTAAGAATATCGGTCTTGGGATCATAAGTAACACCTTTAGTATTTATTAACAAAATGACAAAAGAGTGTTTATGGGTCAACCAATCATGTTTTCATCTACACTAGTGATTGACCCATTTTAACATGTAGTCTAACCCTTCCATATTGCTGCCTCTGCTAAACAATATTTACGCTTATTCACAATTTCACAAGTGACTCTCATGTATTGATATTACTATTCAATTTGCGGTTCATTTTTATTTACTCTTGTTCAAAGTGGCCAAGGTTTTGATTTTTGAGTTTCAGTACTAACCTCATTCCTACTGGTTGTTACCAAGATTGATTAATAATCTTTATAAACTCACGCAGATTTTCAATGTTGAGAGGGATCATTCCACATGGAGATCAAAAAAGAGATAAGGCGTCGTTTTTGCTAGAGGTATACTATGAGACAAAATACTCTTTTATGTGATTATGTTTTTATTTAGAAATCAGCATTTCCAAGCTTTTTATATTAGTTATTCTCGACTCTGATTGAACATTCATCAAGTATTTCTAGGTTATTGAATACATTCAGTTTCTACAAGAGAAGGTGCACAAGTATGAGGACTCATCTCAAGGATGGACCAATGAACCACCAAAAACAATTCCAATCAACGACTTCATTCATCAACCCCAAATCCCAACAAATCAAAACCTATTAGATTCTAACATGATCTCTCATGATGCTACTAACGAAACCAGTCAACACCCACAATCAACAAATAAACCATCTGCTTCTGCTGCAGCCTCTTATGAACAGATTTCCACTCCTGATACAGCAACCACAGCCTCACAGGCTCAAGAACTGATAATTGAAGGTGGCACAATTAGCATTTCAACCATCTACTCTCAAGGGTGAGAATCTTTACATTGTCCTTGTTCTTGATCAAATATGGCAGTTTGAACCCATTTGCTTGAAAAGGGGTTACTCTGGGCCGTCCTTTACCTCAGACGGGTCAGATCAAAACGATTAGCTAATAGAGAATGGGTCTAACAGTTTAAGTGACCCAAAGTTTACTTTTGAGCATCAATCTCTTAAATCATTTCATTCAACGATTCAGTCATTTTCTTAAAATAATTATTAATCGTACTTTTTGTTAATCATAAGTGTTTACTGGTCAACCGAGCTCGTTTTGACCCATTCTAAAATATGACCTGTTTCAATTACAAATCTAGTGATCATATTTCTCTAATATGGACTGACAACTTTTTCAAAACAGATTAGCTAGTGCTCTAACGCAAGCACTAAATAGCTCGGGTGTGGATTTGTCACATGCTAATATCTCGGTACAAATTGATCTTGGAAAGCGATCAAATGCAAATACAAATACACTTGAATCCTCACCACACGATCTCAAGGTACATCCCATAACAACTTCTAGTCATTTAAAATTTCTCTAATGCAGCTTTATAAAACCTTTTGCTCGTTGTTACAATAGGAGAATGAGACTCGTTCCAATGATCAGTCAATAGCACATTCGAGACTTGTAAGCACATGGGAAGACGAGAATGACCAAGGTTTCAAAAGGTTGAAGACAAGCAGAAATTGAAAGTTCATATAACAAATTTCATTCATTTGATTCAGTTCATCATTTAATGACCCTCTTTTAACAAATTGAGGATCATTCATGTGTTGTTTTTGCGTTCAACATATACTGTAGATGTGGTTTCTTTCTAGTAAATTTTGATCTCATATGTTTGGCGGGGTGGGGGTGTAGAGAACTGTAATATTCATTGTAATTTCTGCAACATAATTTTGAGGAGCTTACATTTTGTCACAGGCGAAACTTTTTTTTCCTATGAATATGCTCAACAAGTTGTAGCCGTGTAATAGGGACGGAATCAGAAACAAATATGATCGGGTGCACAAATTTTTTGAACCATAATATAATAGAGTTAAAAAGTAATACTAGAAACACATTTCGATGAGCCGTGTGCATTTAAGAAAAATTGATGTTTTTTTTCAAAAATTAACACTAGATCGAAAAAATCCATGAGTGTCCCGGGACCGCGAGAACCCCCTGATTCCGTCCCAGCATGTAATGACCTACTGAAGTTTACAATGTGGGAATGAAAATCCTGAATAATACCTATCAGATAGAGATAAATCACATTGTAACATACAGTTAGTTGATATGCATGTAAAATACCATCTGATAGAGATAAATCAAGAATTTTGGACTTTTGGTGGTTTTGATAACGACGATAGTAAGTAAATGGGTTAAGTTAGAAACTGAA

AabHLH14

TTATCCACCTCACTCATTCCTTTGCATTCCCTTTCCCTTTTCTTTTCCCCACTTTAAACCAAGCGGGCCCTTAACTATCTCACAACATTTATACCACATATTTATCTCACATGTCATAAGCAGGAGTTGAACTCAGAACTACTTGGTGGTCCAACACAAGTAATAATTGAGGATTTGATTCCATGCACAAATATCTTTCATTATTCGACATGAATGGTAATTTGTTCATCAGTGAAACATGTGACTTAGTTCAGACATTTTAGATGGCAAAGTACTCTTGCTAAAATAAGAACATGTCATGTATGTTCAAGAAAAATATAATACTAAAAATCACAAAGAATGTAGTAATATTTTAGTAATGAAGTCTAAGATGCAATGGTGGTGTTCCATGTCTCTGCTTTTCTACAAAGTTTGTTCTCATTTCTCCTTTATTCACACGGCCATCTTAAGACTCAAAACATTCTCTTCAACAGTTAAAACATACTGTATAAAGTAAGATAAGAAAACAACTTTGGTTAGGATAAGGTATAATAAATGTTCGAGGGTCATTGAAAGCAATTTATGTCTAAGTAGTGGCCCAAGGTTATCCCTAGCTAGTCCATATATATACATGCAAACTACACCACAAAAGGCAACTTCAATTCCTCTAATTAATATTCCATCTTCTTGGTTTATATGCACCATACACATTGTATAGGACCTATCAAATAAGTATACAAACTTTTAATGCAGAAGTGTTTATCAACAAACTTTCCTTTTTACACATATATAATAAACTTCTTAAATCTCCATATCCATATACTTATTGTGAACTGTTTTCCTGAAGATACTTTCTAAGAAATGAATCAATGTGTACCGAGTTGGGATTTGGAAGACTTCAATTCTAATCTCGACATTTTCAAGTAATATCTCTTTACCTTACACCATTTAAATTATCGCGTGTGCTCAAGTATTCATATATGTTTACATGAATTGCTTATGTGATATTCCATGTATGGATTGTACTTGTGTCGATCGTTTTTTTAGAATTTGAAAAATGTTTTACTCGAACTAGTTAAAAATTCCGTACAAAATATGCTAAATGAAGATTAACTACGCTATGTTTTAGGCACTTCATAAAGTATCTAAATGGCATTTTACCTAAATATTTAGAATACTAATAGATCTAAGATATATATAGTTATTCACTTAATTCAAGAATATTTGTAAGAGTAGTATCTTCTTTTATGCGTTAAAAATAGATTAGGATAGATTTCTAACAAGAGTGTAAAATGGTCAACTAGTGTAAGAGAAAATTTTATTAAAGTCTAACACAAAGAATTTCATGCAAACTTAACGTCGTATTTTTATTTTGTTTTATAAAAAGCGATAGTATAATTTTTATAATTTATTTTACAGATTAGGTTATGAAGTAGCAGAATTGACATGGGAAAATGGCCAAGTCGCCATGCACGAATTAGGTCACCGGCGCGTGCCATCTAAATCCCAACCAGAAACCTCGTGGGACAAGCCACGCGCCGGTGAAACCCTTGAAGCTATTGTAACCCAAGCCACCTACCAACCCTACTGCAAGACCCATGTGGTAGTTAACGACAACGAGCTGGTCCCATCTGCCACAATGGCTTCAGACGCATTGGTACCAAGTGCTCGTGATGCTGGTTGTTCAACACATGTCGGTTCATGCAGTAATGACCCATCAGCGTTTTTAAACGAGAGGGTGGCTTGTGGTGGTGATGATGGTGGTTGTGCGACGGTTAGATGTCATGATATGACCATGAGTGGATGTGGGACTTATGAGACGTTTGATACTTATGATGGCGATACGGGTGGTCAACGGTTGATTGAGACTTCAATGGGGTCGCCAGAAAATACTAGCTCCGGTGGAGATTGTTTAAAGTCAAGGTCTCCTGATGACTCAGCTTGTCATTGTAGAACTAAGGTAATATATGCAAACAGTGTATAATGTTTTTTGAGTAAAGGGTTTCCGGTAAATGTATTACATCAAAAAAGCGGTTAATTACATACGATGTTGGTGAAATCTCACATCTCAGCGCGTTACATAAACGAAAACAAATGCACCATTTGTGATTCAAACTAAAACTGACACATGTCCTATTATAGAAGTGTATGTGAAAAAATTTCAAATATAGAGGGTTAACATATATTTTAAGTACCAAAAATACTTACACGCACGTATGTATGTTACTACGTACTTACGGGACTTGAACTCAAAACTTATGATTTGTGAAATGATGCCAGTCGTTTGTACTCAAAGAAGGACTTGAACTCAAAACTTATGATTCGTGAAATGATACCAGTCGTTTGTACTCAAAGAAAGTAAGACGGCCCTTGTATTACACAGATTATTAAGTAATATCCAAGAACATGATCTATAAAATATTTCTCTCCTGGTTTGATTTGAGGTACAATGTTTAATAAACCGATTATATTATTTTTGAAGCAGGAGGTGAACGTAGTTACGGAAAAGAAAAGGGGGAAATCGGAATCTTCGACATCAACTAAGAGAAGAAGGACAGCTGCAAATCATAATCAATCTGAACGGGTACAAATTTTTCTTGTGTTTAATGTGAAAATTGTTTTAATTTACTTAATTATTTTAGCTTGAAATGCTTAATTGTTTTGATGTGTAAAACTTATAAAAGCAGGTTTATTATAAATGTAATTTTGCGTTACGAATTTGTCAGAAACGAAGAGACAAGATTAATCAAAAAATGAAGACTCTACAGAAGATGGTTCCGAACTCAAACAAGGTAGAGTTAATTAAGTAATTAACGCAGTTGTGTAACGTGAAAAACACTTGATTTTACAAATATAAAACACTACAAATTATTTGGTAGTGGACAATGATATATGTAGAACCACCCTTGATATCATGGAACTTTTATATGTGCATGGGTTTCTAAATATTTAAGTACAGTTATTGTACTAGTAGATAGCTAGTAATGAATTAAATCAAGTCTTGCTAGAGAATTCTAAATTGATATATAAATGTGTTTTAAAACGTAAACTTTGACACAGAAAAACAGAGGAAATATGTATCTTGAGATTATAGGTTACTAACTTGACATACACTTTCTGTACGTATGAGCATCAGTTACGTATATATCTTTGTATTTGCATAGTAGGCTAATGTGAATTTGATATTTCAGACAGATAAAGCGTCTATGTTGGATGAAGTAATAGAGTACCTAAAACAAATGCAAGCACAAGTTCATATGGTGAATAGAATGAACATGCCACCAATGATGATGCCATTAGTCATGCAACAACAACAACAACAGCAACAACAAATGCAAATGTCTATGATGAACTCCATGGGCCTCGGCATGGGGATGGGAATGGGAATGGGAATGGGGTTCGGAGGTATGGATATGAATACCATGGCCCTGTCTCACCTTCCAACCGGTTTTCACCCAACCACTTTCATGCATATGCCTTGGAACAACCACATCACTGATCGGGTTGTCAATTACGGCCCAATGGCTGGTGATCCCATGTCTGCATTTCGTCTGAGCAGGTCTCAGGTAATACCATATTTGCATTGCATCTTCTTGTCAATCCCTAAATTTGACTGTTTAATATAACCATGATAAATTATGATAGTTAACGTGTACATTCTTGTCGCAGCCAATGAACATGGATGCTTATAGTAGAATGGCAGCCTTGTACCAATATATGCAAAATCAATCGAGTGGGTCACATCCAAAGAACTAACACAATTTTAGTGATATGGAAAAAAAAAACATATATATACTTTATGGAAGGTTTTGATGTTTCTCACCCGTGTGATTCATGCTAGTTGCTTTGGTCATTTCAAGATACGATCAAGAACTGTTGGTAGTTAGCGTTGCTACAACATATCTATTACTATAATTTGAGGTAATAGCGTAAGCTCGTTACTATATATGGGACTATTAGTTTCTGCTCTTCTTTTTGTTCTACAAGTCATCGATACACTGGTTTAGTTTTAGGTTTGTGAGTTTGAGTCAGTTGTACCTCGATGATGGGACTTCACCTAATTTGGATTTGTTTGATGAATTTGAATATGTTAGTGTTGAGTGATTAATCAAGTTAATTGGATATTGTGTGGGCTGAGGTTTTGTTTTTAGCCTTTTTGATCACATTTTTTAATCTTAGACTCTTAGTGGACTATTTTAGGTCTCTGTTTGGGTTCATTTGGGCCTATGGAGCCTTCATAGTTTGATTAGTTTCTTATTTTTCTA

AabHLH15

AAATTGAAAATACTACTATTATCACTTTCCTATAAAGTCAATGGTTCCAATGATAAAATGAACAAATAACAAAAATAGAGAGAGAAAAATAATAAGAGACACAAACAACAGCACACACCTTATCTTCATCTTCCTAATTCATGTTGAGCTTCATGTCCTTTTTGTACCTCATAATTCTCTAAAACTTTTTAACACAATCATATTTGTCAAGTTGTTGTTCACTCTCTTTTATTAGTTAGATTTTGAGAGTGATTGTGTTCTAGAAAATTGAGAATTTGTATTGGTTTTTCGCGGTTTTGAGCTAATAGAGGATGAATCATTGTGTTCCGGATTTCGAAAACGAAGAAGACTACTTGCTTCCAGCTTCCTTAAACTCGAAAAGGCACAAGAAGTCAACAATGTAATTTTAGTTATATCATGTTAATTACTTTGTTTTTAATAGTGAATTATTGTTACTTAGATATGTTTTTATTTTTATTCAAAACAACTTTTTTGCTAGCAGTTTTGTATATTAGTACAAAACTGCAAGCAGTTTTGAGAGCGTTTTCAAAACTGCTTACTGCTTGATTAATATAATACCAATATATAAATTTGAATTTTGTAGGGGAGATGAAGATATAATGGAATTACTATGGCAAAACGGACAAGTTGTAATGCAAAGCCAAAACCAAAGATCCAACGGTAACAACAACAACAATAATAAGAAGATGGAAACACAACCACCATTAACAACTAACACCACCGTAAACCACCATAACAACAACAACCGATCCACCGTGCTAGACGAGGATACGGCGCCGTGTAATCTCTATATGCACGAAGATGAAATGGTGTCATGGCTTCATTACCCTAGCGATGACAATAATAATCTAGACTTATATTTACATAACAATGATATTTTATATCCAGTTCTCCCTGCAGCGAATGTCGCGCATTCGTTGCAGGGAGTTTCGGCTATTTCTATTCCTCCGCCGCGCGCGTCATCAGTCGTGACGCGCGTGGGAGAGGAACAGAGTCAGAATAAGTTTGGAAACTTTTCGCATTTTTCTAAACCTAGTTTTGTAAAGAATAATCCCACAGCTGGATCAGCTCCGACAAGTTCTAACAAGATGTCGGAGGTGACTACAGTTGTGGAATCTAATAATCAGCCAATGGAAATGCAAAAGTTACGAGGAGGGATGATGACGTCATCTTCGGGGAGGGAAGTTGAGACCTATGATGTCAGCGCGTCGACCTCGTCGCCAGGTACAGGTGGCTCTGGTGCGAGTGCGAACGCTGAACCGGCGGCGGGAAAGAAATCGCCGCCGGTGGCTGAGGATAGGAAAAGGAAGGGGAGAGATGTTGCCGAGGAGACTGAATGTTACAGCGAGGTAAATTTTAAATATTACTCCCTCTGATCCAAATTATAAGTCTATTTTTAATTTGCACAAAGTTTAATAAATTCCCACTAAAGTGTGTTTTGATTGGTTGAAAAAGAAAATGCACAAGTATTTTGAGACAACCAAAAAGTGAAAAATGGACTTATAATTTGAGACGGATGAAGTACTGTTTTTTTTTTTGGTTATTTTTACTGTTTGTTTGCTGTTATTGAGTTGTACGTTTGAGTCAGTAATAATTTAGGATGCGTTTGGTTCGCAAGTGGGATTGGAATTGGATTTGGATTTGGGAAAAGTTTGAAAGATTTGGATTTTAAGTGTAGGTTTGAATTAAATGAGGAGAGAGAAAAATAAAATAAAAAATATATATATGAGTGGTCTAAAATTTTAATTTATGTATTTATTTATATATAAATTAAGTTAAAAAATGAGTGGTTGAATAAAATGAGTGGTTGAAAATTTTTAAGAAATAAAAAGTGAATTATGATTGGTTACTATCAATTGAAAGGAGCCATTGAAGGTTGGTATGAAATCTATCATTTTTGATAGTGTGTCAAAAAACAAAAGTGCTACCAAACATGTTATTTGATGTGTCTTTTAATCCATACCCACCAAAAACCTAATACCAAACGGGCCCTTAGTACTGTAGTTTGCCTCTAACGGAATAACAGTATCTGATTTATTTACAACTGTGCACTGACGGTTGTACTGTGGCTGAAAAAATTACAGTGCCATTTTTAAGCTATGGTTAAACTTTTTTTTTTTATCGAAATAAGGTATAGTGAGAAAACATCTCCGTATAGGTTAAAATAAGATCTTGTCACGTACTGTCTTTTTCAGTAAATGTTAACGGACAAAATAGGTATTATTTCAAATTTGCAAAAGACATTTTTTTTTAAGATAATTTATAAAAGTTACTCCCTCCGTCCCATAATAAGTGTCCAGTTTGACTTTTTAGAGTCAAACTGGTTCAACTTTAACCTTAAATATTATTATTTTTGTTATATAATACTTGATGAAAATTATACCATTAAAAAATACACTTAAAACTTAATCCATTCACATAGTTTGCATCAATTATTATATAACACACGCAAAAATAGTTAAGGTTAAAGTTGAACCAGTTTGACTCTGAAAAGTAAAACTGGACACTTATTTTGGGACGGAGGTAGTATATTTTTTTTTATTAAATTATTAAAAACCGTATGTTCTATATCATTGTTAATTTTTGTCCAATATGCTTCTACTATTAGTTAATGATGACAACGTGTAATTTGTATTGGGTTAATTAGTATTTATATTTGTATTTAGTTAAGAAGTTAGTTTTAGTGAAGCATACATTTTTAGTTTCGAAATTGGTTCATTTATTTTTGTTTAACTAAGATGTTAGCCCGTATTGGCCCCGAAGATGCGTTGATTATTTATTTCTATTATAAGCTAAAACTAATTAACGGGAGGTACAAAAATCGTAAATCATTACCTTTGTACTTTATTATTCTAGTTCTAAAACTAATTAATAGGAGGTACAAAAATCTTAAATTATTTCCTTTGTACTTCATTCTTCTAGTTTTTCATGTAAAGGTTAGGTTGCAAATTGTAACTTTAAGCGTGGCATGTAGAATGCCCGAAGAGTTATATTATCTCGTGTATACTTGATAAATAGTCTACGTAACGCTAATTCTTGTTTAAGCGCGTAAACTGAACCGGGACTGGCCCCAAAAGGTTCAGGAAATATTTTTATGGTGTATTGCTAACTTTTGCGAAAAAAGCTTAATATTTGTGTGTGCCTCATGAAAAGCAAAAGTTATTCTTCTACTCTTGTTTAGTTTCACTAATACAATATTTACTTTTTCTAATACACGTGGTCGGTTTCACTAATACATTATTTATATTGTGTTTTGCTGTGTTTAGAAAATGCAATGATTTTTTCTAAACACAACAGATATAAGAAATATGTAAAACACTAAAACTTTTTTTTTCTTTTCTTAAAAAGCAGTTTTAAAAATGCATGGCATAACTAAGTGCGTAGCAAGAAATCATATTTTAAGCAGTAGTTTAAATCAAATTTAGTATCGAATTAAGTAGGTTTAGGTTCAGTTGCACCCACTAAGTTCTACGTGTCTCCGCATTTAGACATAATCGCATGTCTAGGCTTTATTTACATCACTCTCGATATATGCATGTTGATCCACTTGAGTTATGTTAAAAAAACAATTTAAGTCCAGCCAATAGATTGTGTTGTTCTTGTAGTTTCTACTATACTCATTGTGTCGTTTATATAACGAACCCATATCGTGCAAGTTTCAATGCAACCTTTGGCTTTGCAAAAACACGTCGAACGCTGTACGTCTTCACACGTGGTTTTTAGTATTGACACGTATGTATGAAAAGTGGGGTCACACTGACTACAAAACACCTTCATACATTTTTGGAGATTGTACTAAACGACAACCTTTTTAATATGTAATAAACATTAAATTTTGTACTACATGTTAGGAGAACGTGTTAGTTATTGGATATAGGGAGATTTCATATTTTAGTGTTATAGATAACATCACACTTATATATCTCAAACTGTATTGTCATGTTACATATACTAGTAGTATATAGTAGTTTCTTTAAAAAGAAAGTTATAACTTATATCTAAAGGCCTTTGAGTTAAAATTGTAACCGAGACATTTGTGAAATAAATGGTACGTGTGGACAAAAGAAAGTTATACCAAGTATATATGATTTATATGATATAGTGAACTTTTCTATAATCTTAGTGGAGAAATTTAGTAAAGATTTAGTGTTATGTGTACAAGGATTACTAATTTAGCTTTGAACATACTTGACAAAAATCCTTCGTCGTACATCGATCGAGTAATAATATGGTTATAGGAAAGGATTAACGCGTGTCTGAAATTCGCGCAAGTAATTAACATATTTGAAACGACCTCTAAAATGTGTAATTCTTGGGTACTAAGCAGTTTGTACAGGATGCGCATTTGTTATCAAGGCCTGTGTAGTATGTACAAGTGCTGCGCGCATGAATTTATTTCGATCTAGCTAGTCTCAAAGAGTGCCATGTGCATGCTTTTATTATTTCGACTCGATCATTGTTTTCAGCACTATCATGTTCAGTTTTGTTTGCTCCAATTTGGGATCTTGAATGTCAAGTCACCTGCTTGAAATTCAGGACTCGGTTGTACAAGCGCTTCATACATTTAAGTTCTTAATAATTGAGTGTCAGGAGTATATCATGTGCAAGTTTTATATTGTTTATATTTGTTTATTTTGAGACTTTGTGTTTTACTTAAATTTAGGATGTCGAGTTTGACTATCCTGAAGCAAAGAAGCAATCACGTGGATCAACGTCTACCAAGAGGTCTCGTGCTGCAGAAGTCCATAATCTCTCAGAAAGGGTTTGCATGCTTACTGAGTTTCATTTCAGACTGGGGTACGGTAATGCCGTGATAATTATGTGATGAAGTTTCATATATGTGCAGAGACGTCGAGATAGAATAAATGAAAAGATGAAGGCCTTACAAGAACTGATACCTCGTTGCAACAAGGTTTGATTATACATGTTCTTTAAGTTTGCGTATGAATTACTAGTAGTTTCCCCGCTAAAACTTGTTTATCATGTTTGAATGCTAGTCTGACAAAGCTTCAATGCTTGATGAGGCAATCGAGTATTTGAAATCACTCCAAATGCAAGTGCAGGTATTTATCTTTCTATTTCGTTGAATAAGTACTACTTTTTTTTCGCTTCTGTAATCAAATGGCATACATGTAGTGTCACTTATATTTTGAAAATCCTTGATTGATGAACTTGAAAAATCAATAAATAGAAAAAGTGTGACATGCATAACCATTTATCAATTATGCAGATGATGTCCATGGGATACGGCATGGTTCCTATGATGCTCCCAGGTGTCCAACGGTACATGCCACCAATGGCTGCTATGGGTATGGGAATGGGAATGGGAATGGAACATGTAGGAATGAACCGACCCATGGTTCCATATCCAGGAGTTCTTCCAGGTCCACCCATGCCAAATCCAGCAGCAGCGGCTGCAGCAGCCGCTCATCTCAGTCAACGATTTCCAGTTCCACGGTTTCCAATGCCACAGATTCCCGCCATGGGACCGGCAAGAAGTCAAGACCCGATGATGAACTCGCTTCCACCACAAAACGCAAACCAGCCAAGGGTTCAATTTGCAGATCCATATCAGCAATACATTAGTCTTCCCCAAACACAACTGCCACAACCGCAGGTATGTTTAATAGTACTAGACTACTTTACAGCTAGTTTCAAATAAATCTCACAGATACTAGTGCTAGATTGGAAAATGGGTAGGTCTGATAATTGGGTTAAAAAATGGATTTGGGCCAAAGCGGGTCATAATAGTGTAGGTCCTATCTCAAGAGGATTAGTTTGGTGTTTTTATACCACTAAATAAAATACTCGCCATAACCGTTCTTTTGTGCAGAATCAAGCAGGAACGCGAACGCCTCCAGTTACAAGTATGCCAAGCAGTAGCAAAGATGTTAGGGATCCAGGGCATCAGCCAACAGGTATGCTTAGCCCTATTGGATAGTATCCATAATGCATTAATGCATATGTTGAATGAAGATGACAGTTGTGTCCAATCTTTGTGCCTTTGCAGGCTGATGTTTGGGGGGTTTCAGGCTCTTAGCCGATCAAATTTTGTAGTTCAAAGAGCGAGATTATCTAAACCGGGTGGATCACACCATATAACGTAGTTGATGTGTAATTATACCATGATAGATGCAAGGCCAATTGCTTTAAGCACGCACTGTGTACAGGATCAGAGATATGTTGAGATGTTACTTATTTACCTATTTAGTTTCCCCCCTTTTGACATTCTTGTTCATAATTGCTTAATAATTTCAGATGTCTTTTAGATATAATTACAAAATTATACTTAATATTTGGAAATGAAACGATGTACGAGGTCCGAATCATGTAACATGTTTCTTTAACCTAGTTTTATCGTTTACCCACGGGTTTGATTCGTAAGAGTTAAAAATATTTGTGGTCATTTGTAAGTCAAAATCGATCTATGTAATGATGTTTCTTACACTTGCAATAGACTCTTTTCAGTAGAGATGTGATTGTGATTTGTACGCCATCAAAATTATTTGATACGCCAAAGCACGTATAAAACACATTGTGTTTACAACGTTGGCCTTACGCAAACCATCTTTCCTTTCT

AabHLH16

TAAACATAGTGGCATATTACACATTAAAAGCACATCGCCTAAAATATAAGAACTGATATACTTATACAATGTACATTATTGAAAATGATTCGTTACTGGTCTGGTGGTATGTGAGTGGTATAAACAAAAAGTTATGGATTTAAGTCTTGTTACGTTTGAGACATTTGTAACATATTACCGTTAAAAAAATAATTCGCTAGTATGATCCTAAAAATAAGTCTAATAATAAAACTAAGAGATGGGTTAAATCAATCTGCTTCTAATCTTATATCATTTTCATGTAAAATTCCATTAAAGTTTATCATGATAAAAAGATTCTTCTTTTGCAATCATCTAAACTATTACACAACAGTACACAATTTTCTGTTTTTCCAACACACACATTTATGTCTGTCTATATGACATATGACTTTGACATATTCAAAAACTAAAAATTGAAAATACTACTATTATCACTTTCCTATAAAGTCAATGGTTCCAATGATAAAATGAACAAATAACAAAAAATAGAGAGAGAAAAATAATAAGAGACACAAACAACAGCACACACCTTATCTTCATCTTCCTAATTCATGTTGAGCTTCATGTCCTTTTTGTACCTCATAATTCTCTCAAACTTTTTAACACAATCATATATATAGTTGGTGAGATCTTTGTCAAGTTGTTGTTCACTCTCTTTTGGTAATAGTTAGATTTTGAGAGTGATTTTGTTTTAGAAAGTTGAGAATTTGTATTGGTTTTTCGCGGTTTTGAGCTAATAGAGGATGAATCATTGTGTTCCGGATTTCGAAAACGAAGAAGACTACTTGCTTCCAGCTTCCTTAAACTCGAAAAGGCACAAGAAGTCAACAATGTAATTTTAGTTATACCATGTTAATTACTTTGTTTGTAATATATTGTGAATTATTGTTACTTAGATATGTTTTTATTTTTATTCAAAACAACTTTTTTGCTAGCAGTTTTGTATATCAGTACAAAACTGCAAGCAGTTTTGAGAGTGTTAGAAAAACGCTCTCAAAACTGCTTACTTAGCTTCTTGATTAATATAATACTAACAATATATAAATTTGAATTTTGTAGGGGAGATGAAGACATAATGGAATTATTATGGCAAAACGGACAAGTTGTAATGCAAAGCCAAAACCAAAGATCCAACGGTAACAACAACAACAATAAGAAGATAGAAACACAACCACCATTGTCAACAACCACCGCCGTAAACCACCATAACAACAACAACCGATCCACCGTACTAGACGACGAAACGACGCCGTGTAACCTCTATATGCACGAAGATGAAATGGTGTCATGGCTTCATTACCCTAGCGATGACAATAATAATCTAGACTTATATTTACATAACAATGATATTTTGTATCCAGTTCTCCCAGCAGCGAATGTTGCACCACATTCGCTGCAGGGAGTTTCGGCTATTTCTGTTCCTCCGCCGCGCGCGTCATCAGTCGTGACGCGCGTGGGAGAGGAACAGAATCAGAATAAGTTCGGAAACTTTTTGCATTTTTCTAAACCTAGTTTTGTAAAGAATAATCCCACAACTGGATCAGCTCCGACAAGCTCTAACAAGATGTCGGAGGTGACTACAGTTGTGGAATCTAATAATCAGCCAATGGAAAATATGAAAAAGTTGCGAGGAGGCATGATGACGTCATCTTCGGGGAGGGAAGTTGAGACGTATGATGTCAGCGCGTCGACCTCGTCGCCAGGTACAGGTGGTTCTGGCGCGAGTGCGAGCGCTGAACCGGTGGCGGGGAAGAAATCCCCGCCGGTTGCTGAAGATAAGAAAAGGAAGGGGAGGGATGTTGCCGAGGAGACTGAATGTTACAGCGAGGTAAATTTTAAATATTACTGTTTTTGGTTGGTAAATTTTAAATATTACTGTTTTTGGTTATTATTACTGTTTGTGTGCTGTTATTGAGTTGTACGTTTGAGTCAGTAATTATTTAGTACTGTAGTTTGCCTATAACGGAATTATAACAGTATCTGATTTGTTGTACAACTGTGCACTGACGGTTGTACTGTGGCTGAGAAAATTACAGTGCCATTTTTAAGTTATGGTTAAACTTTTTTTTATCTAAATAAGGCATAGTGAGAGGAGTAATGATAAAGAGACCTAAAACATAAGCCTAAATGTGTGCTTAATTAAATCATCATTTGACACATCATATTGACATTTTCTCACTCCCAATTTTCACTATTGATTTTGACACATGTAATGATCTTGTAGTTAGCCATGTTTTTAGGCATACATTTTAGGCATGAGTATCATTTTCATAGTGAGAAAACATCTCCGTATAGGTTAAAATAAGATCTTGTCACGTACTGTCTTTTTCAGTAAATGTTAACGGACAAAATAGGTATTATTTCAAATTTGCAAAAGACATTTTTTTTAAAGATAATTTATAAAAGTTATATATATATATATATATATATATATATATATATATATATATATATATATATATATATATAAAAATTATTAAAAACCGTATGTTCTATATCATTGTTAATTTTTGTCGAATATGCTTCTACTATTAGTTAATGATGACAACGTGTAATTTGTATTTGGTTAAGAAGTTAGTACTAGTGAAGTATGCATTTTTAGTTTCAAAAGTGGTTCATTTTTCTTTTATATTTAACTAAGATGTTAGCCCGTATTGGCCCCGAAGATGCGTTGATTATTTGTTTCTATTATTAGCTAAAACTAATTAACAGGAGGTACAAAAATCGTAAATCATTACCTTTGTACTTTATTCTTCTAGTTCTAAAACTAATTAATAGAGGTACAAGAATCTTGAATTATTACCTTTGTACTTCATTCTTCTAGTTTTCCATGTATAGGTTAGGTTACAATTGTATAACTTTAAGCGTGACACATAAAATGCCCAAATAGTTATATTATCTCGTGTAAATGTGTAAACTGAACCGGGATTGGCCTGAGAGGTCTCGGGAAAGATGTTTATGGTGTAGTGTTAATTTTTGAAAAAAAATTTCTAGTCTTGTTTAGTTTCACTAATACAATTTTTACTTTTTTCTAATACACGTGGTCGGTTTCACTAATACGTTATTTTTATGGTGTTTTGCCGTGTTTAGAAAATGCAATGATTTTTTCTAAACACAGCGGATACACAAAATATGTAAAACACAAAAACCTTTTTTCTTTTCTTAAAATGCATGGCATAACTAAGTGCGTAGCAAGAAATCATATTAAGTATAGTAGTTCAAATCAAATTTAGTATCGAAATAACAAGGTTTAGGTTCGGTTGCACCCACTAAGTTCTACGTGTCTCCGCATTTACAAGTTGCACCCACTAACGTCTTTGTTGAAATCACATGTCTAGGCTTTATTTACACACCTCTCGATATTTGAATGTTGATCTACTTCGACTTATGTGAAAAAAAACAATTTGAGTCCAGCCAATAGATTGTGTTGTTCTTGTAGTTTCTCTACTATACTCATTGTGTCGTTTATATAACGAACCCATACATATCGTGCAAGTTTCAATGCACCTTTGGCTTTGCAAAAACACGTCGAACGCTGTCATCACACGTGGTTTTTAGTATTGACACGTATGTATGAAAAGTGGGGTCACACTGACTACAAAGCACCTTCATACATTTTTGGAGATTGTACTAAACGACAACCTTTTTAATATGTTCTAAACATTAAATTTTGTACATGTTCGGAGAACGTGTTGGTTATTGGATTTCATATTTTAATGTTATAGATAACATCACTTATATATCTCAAATTGTATGTCATGCTACATATATTTTGCCCATATCAACAATCTTTCATTTACATTAAAAAAAAAGGTTATAAACTATATCTAAGTTAAAGGCCTTTGGATTAAAATTGTATAAGAGAAGTTGTGAAATAAATGATATGCGTGCACAAAAGAAAGTTATACCGAGTATACGATTTATATGATATAGTGAACTTTTCTATAATCTTAGTGGAAATTCAGCAAGATTTAGTGTTATTAATAACGTTTTGAACTCACTGATATTGTGTACAAAGATTACTAATTTAGGTCTGAACATACTTGACAAAAATCCGTCGTCCCTGAATACATCGATCGAGTAATATGGTTATAGGAAAAAATTAATAACGTGTCTGAAACGACCTCTAAAATGTGTAATTCTTGGGTACTAGACAGTTTGTACAGTATGCACATTTGTTATCACGGCCTGTGCTGCATGCATTTGGTTCGATCTAGAGTCTCAAAGAGTGCCATGTGCATGCTTTTATTACTTCGACTCGATCATTGTTTTCAGCACTATCATGTTCAGTTTTGTTTGCTCCAATTTGGGATCTTGAATGTCAAGTCACCTGCTTGAAATTCAGGACTCGGTTGTACAAGCGCTTCATACATTTAAGTTCTTAATAATTGAGTGTCAGGAGTATATCATGTGCAAGTTTTATATTGTTTATATTTGTTTATTTTGAGACTTTGTGTTTTACTTAAATTTAGGATGTCGAGTTTGACTATCCTGAAGCAAAGAAGCAATCACGTGGTTCGACGTCTACCAAGAGGTCTCGTGCTGCAGAAGTCCATAATCTCTCAGAAAGGGTTTGTATGCTTACTGAGTTTCATTTCAGACTGGGGTACGGTAATGTTGTGATAATTATGTGATAAAGTTTCATATATGTGCAGAGACGTCGAGATAGAATAAATGAAAAGATGAAGGCCTTACAAGAACTGATACCTCGTTGCAACAAGGTTTGATCATACATGTTATTTAAGTTTGCGTATGTATTACTAGTAGTTTCCCGACTAAAACTTGTTTAATATGTTTGAATGCTAGTCCGACAAAGCTTCAATGCTTGATGAAGCAATCGAGTATTTGAAATCACTCCAAATGCAAGTGCAGGTATTTATCATTCTATTTTGAAAAAAAATCCTTGATTGATGAACTTGAAAAATCAATAAATTGAAAACGTGTGACATGCATAACCATTTATCAATTATGCAGATGATGTCCATGGGATATGGCATGGTTCCTATGATGCTCCCAGGTGTCCAACGGTACATGCCACCAATGGCTGCTATGGGTATGGGAATGGGAATGGGAATGGAACATGTAGGAATGAACCGACCCATGGTTCCATATCCAGGAGTTCTTCCAGGTCCACCCATGCCAAATCCAGCAGCAGCGGCTGCAGCAGCCGCTCATCTCAGTCAACGTTTTCCAGTTCCACGGTTTCCAATGCCACAGATTCCAACCATGGGACCGACAAGAAGTCAAGATACGATGATGAACTCGCTTCCACCACAAAACGCAAACCAGCCAAGGGTTCCATTTGCAGATCCATATCAGCAATACATTGGCCTTCCCCAAACACAAATGCCACAACCACAGGTATGTTTAATACTAGACTACTTTACAGCTAGTTTCAAATAAATTTCATAGATTACTACTTACTAGTGCTAGATTGGAAGATGGATAGGTCTGATAGAATGGGTAAAAATGGATTTGGGCCAAAGCGGGGCATAATAGTGTAGGTCCTATCTCGAGAGGATTAGTTAATTTGGTGTTTTTATACCACTAAATAAAAAACTCACCATAACCGTTCTTTTGTGCAGAATCAAGCAGGAATGCGAACGCCTCCCGTTACAAGTACGCCAAGCACTAGCAAAGATGTTAGGGATCCAGGGCATCAGCCAACAGGTATGCTTAGCCCTATTGGATAGTATCCATAGTGCGTTAATTCATATGTAGAATGAAGATGACAGTCGTGGCCAATCTTTGTGCCTTTGCAGGCTGATGTTCGGGGGATTTCAATTCAGGCTCTTAGCCGATAAAATTTTGTAGTTCAAAGAGCGAGGTTATCTAAACCGGGTGGATCACACCATATAACGTAGTTGATGTGTAATTATACCATGGTAGATGCAAGGCCAATTGCTTTAAGCACGGCAATGTGTATAGGATCAAAGAAAAATGTTGAGATGTTACTTGTCTACCTATTTAGTTTTCCCCCCTTGACATTCTTGTTTATAATTGCTTAACTATTTCAGCTGACTTTTGAGATATAATTAC

AabHLH17

CCATCCCCGCCTCCATACCCGCTTTTGAGTAATGGGGAATCCTCATCCCCATCCCCATACCCGGTCAACTCGGGGATTCCCCGGTCAAATCGGGGTCGGGTATCGGATTCTCCGTCGGATTCGGATTTTTTTGCCATCTCTACGCGTGTAATATGCCAACTCTCATGTCCGGGAATGCGAGATATGCAATACACAACTTTGCTTAGTTGGTCATTTTCTAGGTCCGTCACTAATATAATGTGATAGGACAATGGTAGTCACTATGTTTGTGACATCATTTTTGTCACTAAAAGTAGGAGATCGGTTTTTCTTGTAGCATAATTGCATGCCCATCATTATGTGGGTTTAATAAGATCACATAAAATAGGTTGTTCTAGTATACTTCGAACATATCTGTCTGGTACGTGTCAACGTAGATCATAATGTTAAACTACTCGCAAACACAATGTGATGGCATATCAAAATTTTTTATTTGCAAGTAATTTGAACTAGCTCGGCTCATGAAAGATTCGAGCTCTAATAGCTTATTTTTCATAACTTGGTAAAACCGTAAAAGCATTCACACCCAACATTTGAATAATACAAGAGCTAAGAGTATAATGATAACGATGTATATATATCGATCTAATCTTTAATAACCGCTTAATGCTCAAATTTACATTCATTTTGGCATTCTACCCTCTTGTTTACTGTCAATTATCACAAAAAGAAAAAGTATGAAAAAGAAATGCCGGGGACATATGTAGCAAAACCAGGTCCTAGTCCGGCCTAGTTGATTTTTCCTATCAGTCGACAATCGACACACATGAGGGTTACAATCCCACTCCTAATCGACTTGCTAGCCAAGTTGACTTGACTTTAACTTCAAGAAATGTATACTCTAAGTTTTTCTAGTTACAATGTTTTACAGTTAACTAAGTTTTAACTAAACGTACCCTAACATTTTAGAAACTATGTATTATCACTAATGATGTAGATGTCGTAAAATTTTCAACAAAACCTATACTCCCAAGTCCCAACGTAAATTATTGTTTGCAGACATAAAAAACATACACTTAAAGAAAAGAGCACTACTAGAAATCGGTCGAATGTGCACGCTTATTTGTCCACACTTTAAAAAGAGTGGAGTTTTAATTTGTTTTTTACACGTTAATAAGTGTGCATAAAAATATCACAAGTATATGTAAACTAGTCGACGTTTAAAACGGTGTAGAAGACTTGCACTATACAAAGCGTGGATATGAAATACCGTGACTCACACAAAGAAGCGTCGACAAATCTTTTATAAAAGATAGTAAGAAAAAACAAAATTGACCGTAAAAAGCGTGTAGTTTTTAAACAGTATACACACATAAAGCGTGGACAAAAACTATTTTAAAAATTATAAAAAACAAAAGACTACGTGTCATTCCTCTCTATCACTTTCAGGAATAAATTTTTCCCCAAATCACAATCCTTTCTCATCATCTTTGTTTATAAAAAATTTCACTTTCAATAGCTTTTATAAATCCTTTCCCTAAACTCATGTAACAACCATCAAAGCAATGAAGCAGCATATTAATAGTTTTTCCCCAAATCACTTTCAAAATTTAGGGTTTCGTTTTTCCCTAAATCACTTTCAAATTTAGGGTTTCGTTTCCAAATCGATAGTTAAATCACTTTCATATTGATAGTTTAATCGGTCATTGGTCCAGTACGTTGCAGCTTCAAATTGATAGTTTAGTCGGTCATTGGTCCAGTTGCAGCTTCATATTGATCGTTTCCAAAGAAGTTTAATCGGTCATTGGTCCAGTTGCAGCTTCATATTGATAGTTTAATCGGTCATCAAAGGTATAAATTTTGATTGAATTAAGCTTGCGTTATTTTAAGCGTTTTATTGTCGAAATATGCTTGAGTTATTGTCGAAATATGTTAAGTTGTTTAGGTGTAAGATGACGTTAGAAAATTGGTGTGTGGTGTAGTTGAATCTGTTAACGATCATATTTGAAGTATTTTCGATTGTTTGTGTTAATTTGTTGTAATTGCTCGTTAAGTAACGAAGTTAATGCTAGTTACTGTAAAAACATTATCTGTCAACTTGTTTTATACACATTGTTCATCATTCACATAATAATTGATGTCAATTTGGAACCACTGTATTGCACTCATTTAGCATTTGATGGAGACAATATTTTGTTACTTGATGGTCGGTAAACATTAATTTCATCTCTTGCAAAGCTATAATCATTAGTTACTGTAAAAACATTATCCGTCAACTTAATTGAAAAATTGCATGTGAACATAGGAGCCCTTGTTGTTTGCTTTTTCATTATGGTTAACAACATTAGGCCTTAATAAATCTAGTATATAACGTATATTCTATACCCGTTATTTGTCTGTACTCATTCATATTTAGAATTTAATAAAACTAATAAGATTTAAATTCTAATGATTTTAGGCATTATATCGGAAACTTGGTAAGTATAAGTAATGGATAAAAGTTGATTAATGCTCCGATCAAAAGCAAAACTTTTAGAGATGGAGCTAATAATTTTATAAGTTTTGCTCGTGTTAGAAGTATAAGAGGATCCATTAAATGTCCATGTAATAGATGTTGTCTCGGTGAATGGGTAGATTTGGATAAAGCTCACGGCCACATATTGCGTTATGGGTTTTTACCCGGGTATACAGAATGGACTGTACATGGAGAGCATACTATATCTTTAGCACCATCTCAATCTAGTTATGTTAATGTTGAGGAAACTTCTTTAGGTCAAGAGGACATAATAGGTTTGGTTCGTGATGCCTTAGGTATTAATTCTTTGCCTTCTGATAACACACAATTAGGAGATACAACGATGGAAGGAGATACGGGAGAATCCACCAAAGCTGATGATCATGGTGATGAGGGTGTTTCATATAAGAAGTTGTTAGAAGAATGTGACAAAGAATTGTACTCTGGTTGCAAGTACTCAAATTTGTCCTTCACTTTACATTTGTATCACATTAAATGTGTTGGTGGAATATCTAACAAGACTTTTAGCATGATACTAGAACTTTTGAGGGATACATTTCCACATCTTACAGCATTACCATCATCNGGAGATACAACGATGGAAGGAGATACGGGAGAATCCACCAAAGCTGATGATCATGGTGATGAGGGTGTTTCATATAAGAAGTTGTTAGAAGAATGTGACAAAGAATTGTACTCTGGTTGCAAGTACTCAAATTTGTCCTTCACTTTACATTTGTATCACATTAAATGTGTTGGTGGAATATCTAACAAGACTTTTAGCATGATACTAGAACTTTTGAGGGATACATTTCCACATCTTACAGCATTACCATCATCTGCCAATGAAGCCAAAAAGTTTACTAAAGATTTAGGTCTAGGTTACGAAAAAATCCATGCGTGCCCTAATGATTGTATGCTTTATTGGGGTGATAGGGTGGGTCAACAATCATGTCATATTTGTAAAGCTTCGCGATATAAAAGCAATGAAGTGATTGGAAGTTCAATAAATTGATTGCAATTGGAGTCTAGATATTGAAACGTGTAGAAGCAAACTAAGACATCAACTGGTTTGGGATTGCTTATTTATCTTGATTATGCTTCTACTTATAAGTTGCTTTGAAATTGTGTTTGGATTTGCTTATTAGTTATTAACTTACCAGACCTGTTCAGAACCCCCTACTATAAATTACCCATTTGGCCATTACATAGCCGGACCACCCATTGTTTTACCTCTATACTGATAGGCAACTTGTAACCTGAAACTTGTTTGGAGCGTTAAGGAACATACAAGTATTAAGTAGCTCTTCAAAGAAACATCAAATTGTCTAACCACTTAATGCATTATCAAATTGTAACAGGTGAGCTCCTTAAGTCCTTTTAAGTCTTAGGTAGTCGACTAATTATAACCATCCACCATGGATTTAGTTTCTTATAAAGTTAGTGGACATGTTTGCTTCAGTTAGGCTTAAATTGTTGCATAAAATCCTTTGAACAAGAATAATACTTCATGTGTACATTGTAAAATATTAGTAGTAAAAATATAAACAAATTCATTTTTACCTAAATGTGTTACAATAAAAACAAATTAACTACTGGTGGTAAAACTTTCGCACGAAGAAGGCACGAATTTGTGAGTTTTATTGCTGAAAACATTTAATCTCAAAACTATTATAACTCATATTATATTGCAAACTTTGATGTTACGTATCTAATCTACAGAAAGTGACAAATAAAAGATCACCGAATGATTTAGAGTCATTTGATATCTGCTATAAAACGACTGATGACACATATATTCAAGAAGCAACAGCAGAAATGATGGTATACACATACTGGAATATTCTTTTTTTTTTCAACTTTTATATGGCATGCTTTGATTTTTGAACTTAAAAGTGTATGCGCTGTTTGTTTTAGGTTATAGCAAATCAAGAAATATCAAGGAAGAAATTAGAGCTAGTAGGTCCCGAAGGTAATATTGAACCTGCCTTAGAGGCAGAAATTGCTAGGGAGGTTCTTAACAAACTGTTTGGAAATGAAGAACCTCGATGTTTTGGGGCCGGTGTGACAAAATCCCAAATAACTAAGTTTTGTTGTGATCTAAGAATGATGAGGGGTGAAGTACTAGCAAATGAAAACCGTTTTCTGTTGGAGAAGGTAGACAATCAAAGTAAGGAAATAGCGACTCAAAAAAAGCAATTAGAGACTCAAAAGAACAAAGTTGAGTCTTATAGTAAGCAAGTAAACACTCTAGTTTCGCAACTGAATAATATGGGACAACAGCTGAATGAAGTTTATGGAATGTTGAAAGTTTTTCAAACCGCGTTTCCAGATCTATATAATACTGCTTCTACTTCTGCTGCTGCAAGTACATGTGACAAACAAGTGAGTGGTTTATTGTTTATTGCACTATTTTGTTTCCAGTCCCTTGTTTGAAGATAATATGTTTTTTTCAGCCCTCTTCTAGCGCATCACCCATCATGGATCATTACTCACCTGTCATGGATCACTACCCGGGTATATATATTTTTTTTACTTTAATTAGTTTATATTTGATTGATGGTATTTGATACTTTCTGAGTTTATATTTTACAATATAGAACTGATCTTCATTAAGATTAAGGCTATTAAAAATTGGTTGACCTCTTGGTTAGTCTTTATGCCAATATGTAGCTGTAGAAATGAACGGTTATATCTGTTACGAAGAAAAACAGTAAGGTAGTGAATAGTTTTGGGATTGATAGTATAGATTGAGCACATAGTGTGAATCTGTGAAGTAAAGTGGATTATTTTGATGTCATGATAGTTGGCTTTTTATTTTGGGTTTTATGATTATTATAATGTTAGTTGATAGACTTGGTGTAATAAAATGTGTTTGACTATAGGAAATTGTGTGATAAATATGAACATAAAATGGGTGAATGTGTGCATTTTTTAAATAGGTTGACGAATAGTTATTTGATTTGGTTATGTTACTGAAGTTTCCTGGATAATGTAGTGCGGTTTTTGGTTTGCTTGTGTCTTTTGACGCTGACTTATTATGAGTACTTTAATGGCAAATGAAATGTATGGAGTTCTGTTATTCAGCTAACTTGTTGAGAAAGAAAGTTATACAGTGAGTGTTTTCATATACCATAAACAACGATGAAAGGTAAAGCAGACCAGAAAGTAATTAGAACTAGTAACCAATATTACTAACAAAGTAATTAGAATGTATGTGACTGTCGATGACTTGTATTTTGTTTGGAACTTTGAGCAGGGGTGTGGTTGTGATAGGTTTACCGCTGAGTATGAGGATGACTTAGATGTACATGCAGACAATACTGTCAATACACCTTTGTTTATCGCTTTCATTTGATCATAGAATTGGTAGTCTGAATGATCTCAAATCATGGCTTTTGATTTTGTTCATGATACTATTGATTCTGTCATTTAAACTTAAAGTTACTCAAAGACCATATTTAACTTCATTGAGATACTTCTACTTTTAGACATTATACCCTTTTTCTGGTTGAACCATGCATGCCAAGAATACAAAAGCATTTATCAAGATTTGATATGGGTTACTGATTTTTAAGCTGTTGTGTGTTATTTAGGATCAGGTTGAAGACAAAGGAGCAGGTTGACGACAAAAGATAGAGGAGCTGCAGGTTGAAGACAAGTTAGAAGAAAATTTTAATGGATAATAGAAGATTAACTGTGAAAAGATTGATACTTTGTTAACGTACTTAAAAGTCTTTAATATCAGTTTGTGAATCATTAGTTACTGATTTATAAGCTATTTGTGAATCATTAGTTGCATATTTAAGCATATATCTTAAAAATTAGTATGTTATCTTTTATATTGTAAAATTAGTATTAGTTGCAGATTCAAAATCAGGGTCAGATTTGTAAAATTGTCAAAAGAAATGGTTGACACAAAAAAACGTGTACAAACTTATGTTACCGGTTACCATTTTGTCACCCATAAAAGCGTGGACTTTAGATGCCTTTTAACAAGTATTGGTGTTGACATGTACATTGTTTAAACACGCATAAAAGCGTGTACTTCTTGGACATTTCTCCACCCTTTTTAGTGTAGAGTGTTTGAATCTTTGTTAACACTGATAAGCGTGCACATTTTTGTGACGCCAACATACTCCACGCAAAGGAAACCGTGGACTTAAAACTTGTACACACTTTTAACCGTAGGGATATATGAATATAACTGTGTAAAATCCCCTGCTTTGATATAGTGGAGAGAGACCATTGCGTGTATTCTTCGACCAATAAGATTTCATCTCTAGTTTTCTTCAAAGGCTATTGATTGGTTTTGGAAAAAAAAAAATAAAATAAAATAATAATAATAATAATAATAATAATAATAATAATAATAATAATAATAATAATAATAATAATAATAATAATAATAATAATAATAATAATAATAATAATAATAATAATAATAATAATAAAATAAATAGGACAATCATTTTAGAATATCTGATAATGGAAAGAAAACATTTTTAGGTTGAAAAAAGTAATACAAGATACCACAAGTGTTGTTGTGTCAAAATTTGTTTTAAATTTCTTCAACCGAAGATATATAATGCTATCCATCACCTTAAATATTTAAATACTTTGGTAGTAATTGCTTTACAATTGGAATATCATTTTACGAAGAAGAAACTCATTACACATAATGCATTAATGTATAACCTTTTGTACGTAACAAGAGAAGGAGATACCATGTATTGCTGATGCCGTAAAATGTTACTTCCTCAGTCTTAGAAAAGCGTTTGTTTTGTTAGTGACACACAATTTTACAGAAGTATAAAGATTTCATTGTTAACCAAACAAATGTCATTACAAGAAAAACTTATGTTAACCAACTAGACAAATGTCACCACAAAATAAACTACTTGTGTTTGTGTACGCTAAAGGGCCATCAAATGACCAAACTTCTGATAACAGCCTTTAGCCATGACTGGTTAGAGAGTGACATGGTTTTGCTAAAGCTTTTAGCGACAACTTTAATATTTTTAGTGACGCGTCGACAGTCGTTTCTAGTAGTGGATGTCTATATTTGTTTATTGGTATTTGCTATAACCGCCATATAAGAATAGAAAATAAGTTATAACCCGTTTAAAGCCACTTGTGTACGTAGTCGTATACATTTCATACTTTTCATACTCAAAAAAGGTCGATACAAAAATAATATAAATAATAAGAAAAGGAATTAAAGACACACCTTATCATCTTGGTTCGACTTGAGCTACATACTGACATTCAAATCAAAACTCATACCCAAAACTCTCTCTAAATCATTTTCATCTATAAATAAATTAACTCACAATTCATGCTCAAATTAATAGAGGTTTGCTCCACTCCAAGAACAACATCAACATATTTGCATCATTTCATTGAGCTATATTGAAAATTTCAATTTATAAGCTATTTTGAGTGGTGATATATGATGAATCATATTGTTCCCGAATTCGAAACCGAGGAAGATTGCTTGATTCCGGATATTCATAAGAAATCAACAATGTAAAGTGCTTTCAAAGTTAGGTTTTTATGTTTTTATTAGTGATCCAACAATATTTTTTTCATCTTGTAGGGGAGGAGATGAAGATATCATGGAGTTACTATGGCATAATGGTCAAGTTGTTATGCAAAGTCAAAACCAAAGATCTAGCGGGAGCAAGAAGCTAGAAACAAAGCCAGCGGTCCGATCAGCTGAGCAAACTGCTCATCAAACCGGACCTTCAGACTTGTTCATGCAAGAGGACGAAATTTCCTCTTGGCTTCATTACCCTATTGAATATCCTGCTAACGAAAATTCGTTAGAAGGATATATATACAATAATGATCTTTTGTTTCCAACACCACCACCAAATCCAGTTACTACAGCGCCTATCACGCCTACAACTTTACTGCCGCCTCCTCCTCCTTCGGTTGTAGTCCCTTCTCCTCGTCCCCCAGTTGCGCCTATATGGCGCAACCGGGTGGACATACAACCACAATCTAGACAACAACCGAAGTATCCTAATTTTTTGCATTTTTCTAGGCCTAATAAAGCCAGAACTCTAGAGTCAGGTCCTTCTGCACCAGTAACAGAAGCACCTGAATCTAGAGCTTCGCGTGTGTCAGAAAAACCACCGCCTATTTCTGCAGGCGGTGAGAGCGTTAGTGGTGTAGGTTTAGTAGGTACGTCATCTATGGGTAGGGAAGTAGAGACATGTGATACAAGCATGATGTCATCGCCCGATGGGTCAGGAGCAAGTGGAAGTATCGAGCCATCGACTCAAATGCCACCGCCTTCGACTAATGACCGTAAGCGCAAGGGTCGCGACACCGAAGACACCGAATGTCATAGTGAGGTACGCAAATTTTCTTAAATCTAGCTATATGTGCTTATGAACATGCATATAAGTTTAGTCTTATTATCGCTTATAGGTTAATTAGCAATTAAGTATACTTAAGCACACATGACTTTGCCACTTAGCAGCACTTCTTACGTGCATAGAGGGCTGTACCATTGATTTTCGCATTTTATTGATGTTTTAAATTTTATAACAGTGAATACGTGTTGGAAAAAGAAGAAAATAGAGATAAATAAGTGAAATATCGAGTTAGATTTAGTTTGTCCATAAATATCGCGTATTTAAAGCTTCTTGTATGTGTCGTTTAGTAGTAGTGCTTAAATAAGGGCCTCTTACACTCACTCCTAGACAAAATTATAAAAGCATCTTCTTGTGCCAAAGAGTATGCATGTACACGTATACTTGTATTTTACTATTCTATAAATACCGATTCATAGTATACTTGTATTTCCTACAAAAAATATTGTGATAATAAAGAAGCTAAAGGGTCAAACATCTTCCCGGCCGGTTCTATATATAGCAAATTATGAAGATATGCTACTTTTATTAAAGAGAATTTATATCTACATCACTTTTTATGTGATGTGTGATGCTATAAGATATAAAATATGTGTTGCCAATTGCCAAATGACCCAAAAAATTCTTGAAAGGGATGATTCTATGGTTTTTTTTTTTTTTTTTTTTGAAGCTTGAATTTGACGTTAGAATGAATGTTCATTACAATATAGATTAAAAGATCATAGTAGTGAGTTAACTATTCGATGGATATGAGTATAACTTGGCCGGGTCGAGTAATGCCAAGCACAAGCATGTCACTTAGGGACGGTACCAGGGTGGGTTTTAGGTGACATTTGTGGATTTTACGATGTAGTGCTTATTTCTATATTAAAAGAAAATAATAAGTAACCATGATTTTCTTTCTAAATGCAAATGATGCTGAAAATTCTTGTTCCTGACATTGTATATTTTTTCTTAAATGTATCTTCGTCAAATTTTATCCTGATCTTGTACTTGCATGTCACCCTACTGAGTATGCGCTCATACGGAGATCAGTCACCATACCGGCTTTGCCCCTAGACCCTAGTATAAGGTCACAGAAACTTCTACAACATACATGTTAAAACAAAACTCACACTTTCACGTTCTCTGTGGCCAAATTTCCAAGTTCCAATGCATACGATATTTCTTTGCGAGGAGTTGCCACATCACACTTGTTGTGAAGGTCATATATACTCATATATATAGGCCAGCTTGGCACGTGTATTTGCATACGTACGGACAAACAAGTTAACTTGATATCTTTTTATGTACACTGAATAGCAAGTCTTAAAACAAACAAACAAAAATCTGTTAGGACACAGGAAAAAGGTTGAGCTACTCGTGGTTTCAGAAAATGTCTAGTGGCCCAAGTGGTGTGGGTATTTTATGTCTTTTTCCGACGTACTTTCTATTTTCTAACTAGGAACACATACGAAGATGGTCAAGAATATATGGTCCCTTCGGTTGGGCAACGTCATCTTAGTGACTCGTCTTTTTTGGGTAATGAGAACAAGAAAAACCTCATTCGTTTATATTTTTATTTTATGACTAGGGTGTAAAATAGTCGAGATGAGTACAAGCCTAGTTAGTTTGGCTAAACTTGAGCTAGACTCGATAAATTGAAGAAAGTAAGATAGAAGTTTAAGTCTGTTTGCACTTGTACATATTTGACCAATATCTTGGAGCGATTTAAGTTGTAGAGTGGTTACTTTACTAAGGGTCTTTACTAAATTAAGTGATTAGATATAAAACTTTTAAGGAGCCTCTTATATGGGAAAAGTGTATTTTAATGAGTTTTTCAATTAAGAGACATTAAGGGTTTTGAGCCCACAATTCTCTCCGCTAACAGAAAAGTAAACATCCCTTAATCTTTTCGTACTAATGCAAGTCTTTTTTAGATAGTATTCTAATGCTTCATGCATACAAATTTTGACAATAGATATTAATGTCAAGGGGGGGTCATGGTACTCTCTTGGCACTTTTTCTTCATCATTTTCAATTTTGTGTTCCCCCCTTTGGGGTTTCAAATCAAATTGCTTTTGTGAGTGAGTTTACTATGTAAAATCAATGTATATGAGCTATGTTAAGACATTATTATTTGCCACAAGAAAACATGAAGATAAAGGCATGGTGGATTGAGGGACAAACATTTACTTGTAACTTAAGGCATCATCATCAATCGCGGAACCAAAAAAGCATGGATAATGCTAGCAAAACTTATGGAGCTTTTAGTGGCCAAAAGGATGATGGAATCATTATCGCTTTGTTGTTATGATGGGATCATACTAATTTAAATGGAAAAGGACATTAGAGGATCTACTTTAAATATAATGATTAGTTAGTTTGTTTTTTTTTTTTTTGTTTTTTTTTTTTTTTTTGAAAAAATAATGATTAGTTAGTTACTAAAGTGTTTCTTAAAGTTTAAAGAGTCATTATCGGTTGATTACGTGGATAACTGAATAGGAGGGGGGAAAATCATTATTAAGTTTTAACGCAATTTTTTTTGTACGATTAGCGAAAAATTGGTGTTTTTAAAATTTTGTGACCTTAAACATTATATGTTTGTCTTTGTTGCTTAATATATAAGATGATGGATAGTTATTACGGAGTAGTTCTGAATTCTAGATTTTCATATTTAATGTGAAGGACGTCGAGTGTGAGTATCCTGATGCCAAGAAACAATCTCATGGATCAACTTCTACGAAGAGGTCACGTGCTGCAGAAGTCCATAATCTCTCAGAGAGGGTTTGTATTTTACTTCATTTAAAGAATTCAATTGTGTTAATACTCTTGTGGGTGGTCACTAATATGTTGAAATTTTTAAACTTGTTTAGAGACGTCGAGATAGGATTAATGAAAAGATGAAGGCCTTGCAAGAACTAATACCTCGCTGCAACAAGGTACTTAGTATGAACATTCTTTTTAAGTTGCAAATGAAATTATGAGTTTGTGGTTAGTAACTCTTATACGTTCTTCTCAATTCATTTAATATATTTATTTGTAGTCCGACAAAGCTTCAATGTTGGATGAGGCCATTGAGTACTTGAAGTCGCTTCAAATGCAAGTTCAGGTAAATAAACCTTAAATCTCTGAATGTTTCTTTATACCTTCAAATGCAAGTTACCACTTCTAATCTTTTCTATACAAAACACGATTTTATCTATAAATTCATAAATATATATAGGGCTAGTCGTTTTGATGTGAGACGGTTTGATACATTAGTGCTATTCAAATCAATTCTATACTGGGGTTTATTAGAAATGACACATATGATCAATTTTTGCAGATGATGTCCATGGGATGTGGCATGGTTCCTATGATGTTCCCACAACAATATGTTTC

AabHLH18

ATCGTCAATGTGGTTACCGGAGCTGGTAAGCAACATATAAGATTGCATATTCTAGATTTATATGGAAAGAAAGAATATAGCCCCGCTTAGATTAATTCATTATATTGGTTTGAATATTGCAGGAAATGGAAGATGGTGGGTTCATGACTCAATATGATAACATGTGCAAGCCATATGATATGGTTGATAAACTAAGTGTTGATTCTATTTCCTCAGAAAACATTCTGGAAAAAGAATCTTCCATCGATAGATTTTTTCAGACTCCAAGTAGGTTTGAAGAACCAACCGAAATAAATTTGCTTAGTTATCAAAAAGCCAGTAACATTAATCGAAGGTCTAGTACCCCAAACACTATTGCTGCTACTACTCATTCTTCCTTCAACACTTTCACCATATCCTTTAGAGATACAAAGGCTAAAGAAGAGATCCATCCATCTGATGATTCACTCGGTTATGAATCTGCTGGTACTGGAAAGGCTCCAATCATTGCCAGGACTCCACTTCAGGCTCAAGATCATGTGTTGGCTGAGAGGAAAAGAAGAGAAAAGTTAAATCGGCAATTCATTTCTATGTCTGCCCTCCTTCCAAACCTTAAAAAGGTACGATACCAATTTATTTTGCTTAAGACCTCCATTTTGACATATATAAAGAATTTGAGATCCATGCATGTGACACTGTGTTATGATTATTTCTTGTGCTGCTTTAGATGGACAAGGCATCTGTGTTGGAAGATGCAACAAACTACATAAGAGAACTTCAAGATCGCGTGAAGGAACTCGAGGCATTATCAGACCTTATGAGAAAAGATACTAAAGATATTCTAGTTGCTTTAAAGAGATATAGGCTTAGTAGGGATGAGGAAGACGATTCATCTCTGAATGAAACAAACTCTGGAGATCATAGTGCGGGTGTCCCTTCTGAATCATCTGCTGAAATTGAAGTGCGGATATCAGGAGGCAGCGTGCTAGTAAGAATCTATTCTCATAAAACCTATTCATTGGCTGTGAAAGTGCTCAGCCAGATGCAGAGTCTTGGGATTAACATCATCAGTAGCAGCACGATGCCTTTTGCTAATACTATCACTGTTATTACCATTGTTGCACAGGTACATCTTCAAACCCTTATGAATCTGTAGAAAAAAAATAGAGATCTCAAGTTTTTTGTTTCTTGGATAACTAAGAATTCATAATTTTATACAGCTGGATGTGATAAATTTGAACACATACAATTTAGACGTCCTGAATTAATACTTGAATCGATCTATAAATNTCCACTTCAGGCTCAAGATCATGTGTTGGCTGAGAGGAAAAGAAGAGAAAAGTTAAATCGGCAATTCATTTCTATGTCTGCCCTCCTTCCAAACCTTAAAAAGGTACGATACCAATTTATTTTGCTTAAGACCTCCATTTTGACATATATAAAGAATTTGAGATCCATGCATGTGACACTGTGTTATGATTATTTCTTGTGCTGCTTTAGATGGACAAGGCATCTGTGTTGGAAGATGCAACAAACTACATAAGAGAACTTCAAGATCGCGTGAAGGAACTCGAGGCATTATCAGACCTTATGAGAAAAGATACTAAAGATATTCTAGTTGCTTTAAAGAGATATAGGCTTAGTAGGGATGAGGAAGACGATTCATCTCTGAATGAAACAAACTCTGGAGATCATAGTGCGGGTGTCCCTTCTGAATCATCTGCTGAAATTGAAGTGCGGATATCAGGAGGCAGCGTGCTAGTAAGAATCTATTCTCATAAAACCTATTCATTGGCTGTGAAAGTGCTCAGCCAGATGCAGAGTCTTGGGATTAACATCATCAGTAGCAGCACGATGCCTTTTGCTAATACTATCACTGTTATTACCATTGTTGCACAGGTACATCTTCAAACCCTTATGAATCTGTAGAAAAAAAATAGAGATCTCAAGTTTTTTGTTTCTTGGATAACTAAGAATTCATAATTTTATACAGCTGGATGTGATAAATTTGAACACATACAATTTAGACGTCCTGAATTAATACTTGAATCGATCTATAAATGTATGCTTCACTTTTTATTCATGTATCAGATTGAGGAAGATTTCGTTATGACAGCAGCAGATCTTGTGAGTAAGCTTCAACTAGCTTAGCTTCCTGAACTACGATGACATGCAACTCAAAGTTCAAGATTTCCACTTCCAGATTGTTTTGCGGGTCCAATTTAGTCATTGTTATTATACATGATCATTTCGCTTATCTTTGATCCAGATAGCTACTTATTTTTTAAAGTGACTGATATTGTTAGACTATTGCAGTATATTCCTCAATTATGTGTTCTTCGTAACACACCCTTTGTTAAATAAAAAGGAGTTCATTCTCTATTGAGGTCCTACGGATCTTGGATCAATCACGGATGAATATGTATGAGTATGCACAATCTGATGTGCAGTGACTCGTTTATAACAACAGAGAGGGTGGGTTTGTTTGACAAAACACACGTTATTC

AabHLH19

GTTTACGTGTACTGCAAAACGCGTGATGAAATCCTTGTTAATTCACATTATAGAGCTCTAAAAGTTAATATAGTTTAAGAACATGTCCTATTTTTAGACTCAACAAACCTGTTTGAAATAATTATCGAATGAGGACTATACATGTAACCGCAATCATTATGAAAGTATTATCAAGTATAATCAGAAACAAACTCAAAACACCAATTGTAATACTCCGTAGCATTTGTATTCAGAGGACAAGATCTTGCTCATGATGAGTTAAAAAGCTAGATATAGTATATCGTAGGATCACATGACATATGGACATTGTATTATAAGTATAAGTATAATAATTATTTAATAGTAGTAACTTTATCATATCGCTATCCTACGTTAAAATATCCGAATATATTCGTAAATATGGAACTGTACGTATGTTCAACTTTTAGTTTTGGATGATTGTTTTCGAATATTTTGTGCAATAATAATAAATAATTGGTGGTCCTTATGTTTCTCTTTCTGACATTGTTCTTATCATAACTTCTAACCCAATAATAAATGCAGTTAAGTTGTGAAGCAATTTGCTCTCAACTGTGACCCCAAGTAGTCCTAGTCCATATATGTAGCACCTACAGCCAAAAGGCAGCTGTTACCATTTCTTATTCCATATGATATGTACCACATATTCTGAACCTAATACATTTAGTTTTGTCCAACTCTCTTTGCATTGTTGGTTTGAACTACACAAATTTTGAAGTATGAGTCAGTGTGTACCGAGCTGGGATGTTGATCATGAGAATAACTCTCGCAATAATTCGAACCTTGCTCGAAACAAAGTCTCCTTACGTGCTCCTTCCGGTTCCATTTCTTCAACTCTTGATGTTCCAACGTACGTAGTTAATTCTACTTTTTTCCTGTGGGTGATATCAATTCTAATGTTGAGAACTCCTTTTGTGTTACGGTGATTCGAAACATATATTTCTTGTGAAATGAGAGATTTAAATTTCGTAACTAAACTTTCATTTACATGCATGCTATGATTTAATTAATCCGAATATAGTTAGACTTTTTAGCATTTAATATTAAAAGACTAGTTAATTATATACTCCTGCAGTATAGTTTAGACATTTTAGTCTTTTACTTTTTGTTAGAGTTGACTAATAATACCATATCATCTGAATGATCGTTTTGTTATAATGTAATTAAGCAGTTTTGAACCAAAGAATCTCAGTTTAAAAGTATTTTTCGTAACTTTCAATACGACCAAACACTAAAAACGAAAGATTTGGTTTCGGTCATAGACATAATTCTCTTTAGCTCTATATAATGAATTTTGGTACGTAGTTTTCCTTATCACTGTTTTCTAACTCTTGTTCTTGCAGACTAGATTATGAAGTAGCGGAATTAACATGGAAAAACGGACAACTAGCCATGCACGGTTTAGGACCACCGCGTGTGGTTAACAAACCCCATGCGAACACCGCTAATCTCACCAAATACACTTGGGACAAGCCACACGCGGCCGAGACTCTGGAGGCCATTGTCAACCAAGCCACCCTCCAACCAAAACAAAAATCTCATATCAATATTTACTCTGACGACCTTGTCCCATGGCTAGACCACCATCACAATTCTGGTGTCACAGCTGGTACTGTTAGTGCGTCCGGCACAGTGACAATGGACGCGCTGGTCCCTAGCTCTAATACACAACCCCACGCGCTATCTGGTACAAATGGTGCCCCAACAAATTGTTCTACACGCGTGGGGTCATGTAATGGTGACCAGTCTTGGTATGGGGACCACATGACGGCCCAAGGTGGCGCAGCCACACATGAGTGGAGCAGTTGTAGGGACCACAGTGGGAGTGGCAGTGCTACTTTTGGAATGGAAAGTAGCCGACAGCTGACTGTTGAAACATGTGAGAGGGAATTGGGTCCGAAAGGATTCACCTCAACTTCCACGGGCTCTCCAGAAAATACTGTTTCCGGTAAACAACAGTCCACTAAATCGACATCTCCTGATGAACATGATTCTGTTTGTCATAGTAGACCTCAGGTATACCATTAAGTTTTTCATTTTCGTATAGAAATCTCTATAAAAAAATATTTGTAGCAAAAAGTTCATACTCAAGTCTTATTATAAGAGATTTGTAGACTCTAATTCGGTGACTAAATATTTGCATGCTATAACAATATGTGCATATCGTTTGCTTTTGTTTGATTAGTTATCTGATATAATTGGTTATTTAGAAGAATGAAGTGGATGAAAAGAAGAAAGGTAAAGGAAAATCATCGATCTCGACTAAGAGAAGTAGAGCGGCTGCAGTCCACAATCAATCTGAGCGGGTACATTTTTTATATGAATTTTGAGGGTCAATTTGCTCTAATAGGAATTGTTTTATCAATTGAATGATGCTGTCTAACCAAACATAGTGTTGATGAATCAAATATGTAGAAACGAAGAGACAAGATCAATCAGAAGATGAAAACGTTACAAAAACTCGTTCCAAATGCTAATAAGGTATAAAACTAGACTTGATGGATTTCTTGATTATACCATACTAGTTGGACATGATAGTATTTTGCTACTATTTTTATGTTCAAATATGCATTTTTATATATGTTTGGGTGAATTATATCTACTTTCAGACAGACAAAGCCTCGATGCTTGATGAAGTGATCGAATACCTCAAACAATTACAAGGACAAATTAATATGATTAATAGAATGAACATGTCTCCTATGATGATGCCATTAGCTATGCAACAACAACAATTTCAAATGGCCATGATGAACCCCATGGGTTTGGGAATGGGTATGGGGATGGGAATGGGGATGCCAGGAGTCATGGACTTGAATTCCATCAGCGCCAATCGCCCCAACATCCATGGGATGCCGCCAGTCTTCCACCCCTCCAACTTTATGCAGCCGACGATGGCTTCATGGGATATGAACACCACAAGCGATCAAGTTCCGAACCATAATGATCAAATGGCTGCATTTCTTGCATGCCAATCACAGGTTAATTATTAGTCAAACAAATTTTTTATATGAATAGTTTCTTGTGTTGCAAGCTTGCATGCAAATTGTGTGTTAATGAATTGAATAATATTGTCTATGCTTTCAGCCCATGACGATGGAAGGTTATAGTCGAATGGCTGCAATGTTTCAGCAGATGCAAAATCAACCTACCTACCCTGGTCTCAAGAATTGAAGGCAGTAATTTTTCCATTTTTATTTGGTTACAGTTGGCCTATATATTGGGTGATTTCCCTTATTTGGAATATAAGTTCATTTTTTAGGTTAATGAAACGTACGTACGTGTGTATAAATGTAGATTTTGAATATATATTCTAGAGGTAATAACTCATTTTACTCCAACGTATACATACGTCTGAATATATTTTGATTTTGTTTACTCTATTAAGATTCAATAAATTTAACTTAATTAATTACAGAGAGTATGTGACAGTGATCAATCTTTGTTTAAACTGTCTTTACATGAGAGCCACAAAGCAAATTAAAGCCGTGAGTTTTCATGCAAAGTGGACAATATTGTGGTCCGAATAGATGGGATGTTATATACAACGTAGTTATTAACTTCTTGCATACTCAGTTAATTTTAAAACATATAAAGTTTGGTGAGATCTTATTTGCGGAACCAGCTAGAAATTGTAAAGTTTTCCCGGTAAGGTGTGAATTAATCCTTTTTTTATATTTACAACAAAAAAGTTAAAAGAAAAATCCAAATGAAATGTTGATCAATATATCGGTTCTTATTGTGTGACTGATTGTTGTCACGAGTTATGTTTTCTAATTTTTGTAAATGAATACTACTTTTGAGTTATGTTAGGTAAAGAAAGGGTAATTGTTTGTAAATGTATATTATTTGCTATCTTTTCAAATTTTTTTTTTGGTAAATGATTTTCCCGGCAAATGTATTACTTCAAAAAACGTTTAAGTATAAAGAATATGGGTGAAGTCTCATATCTTTAAACAAACCATTACTCACTAGATTATATTTGA

AabHLH20

ATATCATCCCACTTGTTTCTTTTATTTAAAAAAAAAAAAAAAAAAATACCATCAAATCACAAATGTGTTGCTAAAATCAACTCATCTCCAAAAGGGTCTCTTTTAAACCATCTTTTTTATACTCAGGTTGCTTTCTTTTAATTCTTTATTTGGTTATTTGATGACCCTTTTGTTAGTTAATTAGTTAAATGGCTTAAAGATTTGATTTTGTTTTACTTATGTTTGACTGATGATGATTAGAGTATATTTGTTTTATACTTTTAAGTTAGTTAATACTAATAGTTTAAGGTTAATTAGATTGTGTTTTTTCAGCTTTAGCTTTTTAGATTAATAAAGAATTAGTTTGACTGATTAGTAGTAGTATTTTTTTTATCACATTTATTGTCAGATTAAGACACTAGTAGCAGTAGTATATTAATTGGAAAAAAAACATATTAAATTGTCAATACAGACATGTCTTGATTTAAATAAAATACAGTAAATTATTTATGTCAGTAAATAAAGAAGCAAGTTTCATTATCTCATGTCATGGTTTTTGCAGGTGTTCCATCAAGTGGTTGGAGCTTTATAGAACAAATACTATTTAGTTTTATGAGTTTTTGAGAACATTGTTTGTAGTAATAATTTTGTTTCAAAAATGCCACTTTCAGAGCTGTATAAGAACGAATCGTCTCAGCAGAAATTGACCGATATATCGTATATGTAAGTTTCTTTTGGTTGCTTTTTTACGAAAAATAATATGAAATTTTGGTTAAAGTTGTTGAATTGATGTTTGTATGTTGTCATAATTGGATTGCAGACCGAATGACGAGTTTGTGGAGTTGATATGGGAAAAGGGTCAGGTTATGATGCAGGGTCAGTCTAGTAAAGCTAGAAAGACGCCGGTTTCTAGTAACTTTCAGTTTCATGCGCCAAAAGTTCAAGGAAAAGATGGTATGTTGAATGTACCTATGGCGGAAATAGGATTGGATCAAGATGATGATATGGTGCCATGGTTGAATTATCCGCTTGATGATTATTGTGCTGATTTGTTACCCGAGATATCTGGTGTGACGGTTCATGAGCCGACTATGCATAATGGTATGAGTGTTATTGATAAGAGAGGTAATAAGGATACTAGTGTGTTTAATGGTTTAGACCAAGCGAACACATCTAAGGGTTTAAAAGTTAGCAACTTGTTTTCTTGGCCAGATCCGATGGTCAGATCAGGAATTACGGATATAGGTAGTAGTAATTGTAGAAGTAAAGTCGATAATGTGGTTCATAGAGATCCAATACAGATTCAAGGTTCAGCAGGCAGGGTTGAGAAAATTGCGCAAAAACAAGATTCATCATCTACCTTGTTGAACTTTTCTCATTTTTCAAGACCTGCTGCTATGGCTAAAACTAATCTTCAGAACACTGCAGTTAATGTTGTGAATCATGGATCACAGAAAGAAATTGGGTTCGTTAGTCAACCGAATTTGGATTCAGTTGGAGTAGGTTCGAACCCTTTTGTGAGTAAGCCACTTAATGAGCCACATTCTGTTGAAAAGTCATCAAATGTGGTTCTTGATGTGAATGGAGCGAAAGGAGTTCAAGAAACTGTAAAGAGCAATGAGCCAGTTGTTGCTACTTCTTCTGTTTGCTCAGGGAATAGTGCTGAGAGAGCTTCTAATGACTTTTCCAAGAATTCTAAGAGGAAATCTCGTGATACGGAGGAGTTTGAGTGCCAAAGCCAAGTATGTTTATAATCATTTGTTTCTTATATGTCGTGTTATTTTTTAATGTACTGAGTGCAAGTAATGTTTTGATTAGGATGTTGATGAAGAGTCGCTGGGTACTAAAACAGCCAGCGCGTCTCGTGGAGGTACAGGTTCTAAAAGAAGCCGGGCTGCTGAAGTTCATAATTTGTCTGAAAGGGTGAGCAAATTCATCTATTTATGATAAAGATTATTTGCAATTTGGCTCTTTTGGATGGTATCAGACACGTTGAACCTAGAGGTGGAAATATGGGTGGGTTGGCCAGGTTCGTAACAGATCAATAGGGGTTCGGGTTAAAATGGGTCATTTCTAGCACGGTCCAAGATGGGTTGATCGGAAAGACTTGCAGACCTCGTTTAGCATTTGGTTAATTTGTTTTATAATTGACACGTTAAGTATAATTGTCATAAAGATACTATAATAATAGTAATTTAGGAGGTTGTATTAATGTTAAATAAAGGACTTTGGATAACTTCAATCTGTTTTACCCATTTGACCCATTTCACATCAAGAAAAACAACCCACGTCTTCATAAGTAAATGGGATGAAGTTACCACCTCTACTCGAGACCAACAATTACAAGATTCATGAAGCCTGTTGGAGTGGTTGTTTGTATTAATTTAACTCAAAGATGCATACTAATTGTTGGTTTATTAATGTTGAAACAGAGGCGAAGGGATAGGATAAATGAAAAGATGCGTGCACTACAAGAACTCATTCCAAATTGCAACAAGGTACACAATGGAATGATGTAATCTCCGTTTACGAATCCCTTTCAATTTGAAGATTTGTGGAATCTTTTGTACATATCTGTTTATATTTCCAAACTGACATCTTTGTTTTTTTAATGACAAGGTGGATAAAGCATCAATGCTTGACGAGGCAATCGAGTATTTGAAGACACTTCAACTTCAAGTTCAGGTAATATTTCCTAACTAAAATACTAATCTTTATGCTTTCGTCATTCCTTTTCAATTATCATGGCGTTACATGTACTAATTTCTTTTTTGTTGCAGATTATGTCAATGGGAAGTGGATTATGTATGTCCCCAATGATGTTCCCAGGTGGAATGCATCCTCATTTCTCCCCCATGGGAATTGGCATGGGAATGGGCTATGGAATCGGAATGGGAATGGAGATGAATCAGCATGGACACCCAAATATGTTTCAATTTCCTCCAAATGCACAAGGGTCACGGCACCCTCTCCCTTCCCCAACTGTTTATGGGCATCCTAGCCAAGGAATGCCAATGTTATTTCCACAACCGCCTATGCCCAGGGTTCCTATGCATCCTGCTGCTCGACCGGTAGATGTAGCTCCAAGCTCAAAGGACCCGATGCAAACTAAGAACTCTCATGCAATGACCCAATTATCTAATCAGGTATACCTATTTCTTTCGACTAAATTATGTGTTTGGACTTGTGTGTTGGACAATTAGGGTTTTGAAGCCGTAATTTTTAGATGATATAGGGGTCAGATATTTGAATAGTTATCTAAGGGTATAATATAATTACCTATATACTCCTTATTAGATATATTCATTACTTGAACAATCATAATTCTATAGAAACTCAGATGCTTAAAACGTTTACAGAAGCATATCAGAACACTAACGTCTATGGCGTCTGATTTAAACTACAGTGTCACATTAAAATTTCGAAGCTCAAATACACACACCCCCTAAAATATATTTTTGTTTAATACGGAAGAGTCAGTTTCGCTAGAAATATAATGAATTCAATCTTGTGTTTCCACGTTCCAGTATTTTTTTTTGAAAGGCAACTTTTATAAAAAAACAACACAAAGAACAAATACAAACAAGGCATCCCCTTAATTAATCAAGCCAATTAAGATAAGAGGGACCCAAAACCAAACCAACAAAAACTAACAATACCCAACAAAATCCTAACACAACTATGCTGTCACAAACTCACAATCATGAAACAACCAAACAGCCCACATGGAAAAAGAAATACATGTAGAACATGTTAACAGTGGGCATATAAAATCCACATGCAAAGTGCAAAGGCACCATATTGAGTTAACAGTGGACCTGTCCCAAACAGCCATAAAGCAGTTCCAGTATGCATAGAAGTTTAGATTCTTTCTTATAATAGAACACACTCCATGTATGCTTTGCTAGTGACAAACTATCATTCATTAAATTGATCAAAATTTGTATCTGATGCCAGTCCCAAGATGCACACAAGACGCTTAACCAGTCTACTTTGGTTGACAAAAAAGATCAAGGTTTAGAAGCCGGATGTAGTACAGCTGGTAAGCTAGTTTCTTTTCTTTTTGTGCTATAAACTTTAATTTATTTTTGAAAAGTGAAAATTTATTTAGGATTGTTTTGATGTAGGTATTGATTGATCTTTCTTACAAAAGGAACAGTGACAGTGCATAAAGAATAGGAAAGTGAATGTCGCGTTGACTTTTTTGACGAGTTCCTGCAATGACTTATTTTAATCGATTTTTATTTACGTTCTGCGCGGAGATTGGAGAGTCGGGATGAGAAGTATCTTTTTAGCTCGTTCTCCGTTTGTGAATCATCCCATGATAGTGATTATTTGGTGTCAGTACAGTTGAATGTTGCTTATAAACTATTACATGTTAACAAACTTCAGAATTTATGAAACAACAGTTATAATTTTCATGTTTGGCTTGCGGTGTATCTGCAGTGCTGATTGCAGTTATGCGATATATGTTCTTTGTTGCTGAATTTATGTTTTGATCCATAACAAAAAGTAGTCAAATAGCTAGAGGCACCTGGAAAACTTTGAAAGTAAAAAATATGAATTCGGATAAGGCTAATTACATGATTATGATGAAGTGTAAAGGTTTGTCAAAGGTCTAAAGGGGTAGCTGCATGTTTTTGAAAAGTATACTTATATGAGAAAGTAGTGTGAAAACGCAACTATATAATAAATTTTAGGACACAAATGACTGAAATAAAGCTTTTAATCAT

AabHLH21

TTGTGTTGTAGAATTCTCATTTGTTTGATTTTATTTATTTTTTATTTATTTATTTATTTATTTTATCAAAAGAAGATTCCTGAAAAAATGTCAACATCTCAATCTATGATGTCGTAACTTAAGGATTTTTTAATACTTTTTGTTGATATTAAATATTCTCTTATATTTTAAAATTGATTATAAATACACTAATGCACACAATTTTCTTATCATCATCAATTTGTTATTGGACATTCTGACAAGAATCCTTGTTAAACAAACAAAGAAATAAAGAATCCAAAAAGATGCTGGTATATCCCTTCCTTTGTTTACATTGACATATGGATGCGTGTGGAGGACATGATTACCCAAAATCATCACAGGATTGCAGGGACAAAATTTCATACCTAATAATCAAAAACCATGCAACATTTTGAACATGAAAACTCCACTTCTTCTGGCATAGCCGCAAATGGTAGTACTGGAGATGATACTGTAGTGGCAAAAAAGATCAATCATAATGCAGGTGAAAGAGATCGCCGAAAGCGCGTTAATGACTTATATTCGTATCTTCGTTCACTGCTACCCATATCAAGTTATCACAAGGTGCATATGATCTTATGTCCCTCATGATTTAGGTTTGGAGTTATTTTATAATTAAGCAGACTAAAGGGTATTTGTTTAAGCTTTTATAATGGGTTTACGAGCTTATTAGCTTTAGCTTATTTAGTTGAAATAAGCTCATATATTGTGTTTGAATAAGCTTAAAAACTTGAGCGTATGTGCCTATATATATATAAGAGAGTTTTCATAACGCCAACTTTTCTGGCTTTTTAGAGAGGCTTTAGGCTTATTCAAGTCAGGTACGTCGTGGCTTTAGGCTTATTTGATAGGGGTTGTAGCAAAAGACAAATTTACTCTTATTTGGTTCATTTAAATACATTAACATGCCTAGTATCATCACGCCATATGTTAGCCCATGTTAGCTCATCTATGTGATGTATAACAAAAAAACAACAACACGGATGTTAGTAAAAATATAAAAGTAAACGAGTTTAATTTCTAGTATACATGCAACGAGTGAAAGTTGACCGCGCGTTGTGGCGGAACAAAAATGATCAACAATAAGTATTTCGTTTCTTGAATTTTCATTGATCTATATATAATTACTTGTTGTCAACTTTATCCGACATATTTTTTTGTTAAACCTTGAAGTTGTGGACGTTCTTTGTAAAGTGAGGTAACTAACATGGACTATTCATGTAATATTTTGTAACACAAACAATCCCTAAATTTTACTCGGGACCATGTCTAAAATCAGTAGGTAACTAAGGGATTATTTGTGTAAATTTTTATTACATAAATAGTCACATATTTAACATAAGGATTGTTGTTGCAATGCATTTGACAACATGGATAAATTTCAAATTATATTAACCGAATAGGACTGTTGTTGCCATCGCATTTAACGAAGGGAATAAATTTTGAAATTTATGATGGACTGTTTTTGAAACGCAAGTTAACCAGAGAGATTATTTTTACTACTTTTGAAAATATACTGAAGATGAACATTAATTTCCCTCACAAAACATTAAACAATATTTGTTTCACAAATATTATTTAGGGAAAATTCCACTTTTGTGTCAAAAATCGAATTATGAATCATAATTGAGTCAATTTTCATACAACTTCCAATTTTGGTCATGTGCTACCTGTGCTACTGTGCTACCCACTTAAAATTGTGACACGTGTCCAGTTTGATTTGATATTTTGTCTTTTATTAAATATATTAAAAGACTAAAATACCCTCTTTTAAATGTGTGACACGTGTCCATTAAATGTGCGACTGTGCTACCATTAAATGTGCTACTGTGCTACCATGTTGCCGGCAATATTTCCTGCTAACTTTTTTTTTCCAGTTTCCGGCTAACCTCTTTTTTCCAGTTTCCGGCTAACTTTTTTTTTTAAATCAAACTGGACACGTGTCACAATTTTAAGTGGGTAGCACAGTAGCACAGGTAGCACATGACCAAAATTGGAAGTTGTATGAAAATTGACTCAATTGTGATTCATAATTCGATTTTTGGCACAAAAGTGGAATTTTCCCTATTATTTATATAATTCAATTTTATATTTTTGATGTTTTACATATAATAATAATAACAATAATAATAATAATGCATTTTTTAATAAAATGTGTTAACAATAATATAGAGAAGGATGGGAAGGTGCGCAAATATGAAGTTTAATAGTTTTAAAGGTTAATTTGTTATATTACTAAATTTGAAGCATTATTTTTGAAAGTCTATATAAAAATTACCAAACTTTCAATATTACCAAAATTTAAAGGGCTATACAGTATATAAGATTGAGCGTATATTACTGTAGATGAACAATTTAACTTTAATAAATGTTACATATTCAACAAGGTGTTTGTTTTCTTTTAAAAAGTTTAACAACTTCTTTAATAGACCAAAGCTCTTAGTTAGTTGGAGCTTTTAGGTGTTAAGTGTGTTTGTTTTTTATTTTTTGTGCAAAGTCAGTCAGAAATCAGAGGTAAAAACATCTAAACGGACCAGACATGGAAGGAGCATCTTAATGCCTCTAGTTGAACTAATAAATATTCAAACATTTTCTTTAAAAACACAAACGGTATTATTTGATTCAGAGTCAGACAGCTTCAGATTCGGATTCATATGTGCGAATCAGATTCAGATTCAGATTCAGATTCAGAGCCTCTTAATAAACAAACAACACCTTAAGTTCATTATAACACTCGAGTTTACCAATTTACGGAATTTAAGACACTTATAGGCTTATTATTTATAATGGATAATTACCTTTAAATGTATCAACTTTGTTATTTTTACATAATATGTGTAGTCATCTTTCTATTGTGATTTTATATAACCATATTTTAAAATGTTGTTTTCTTGAATATTTTATGATTATTTTACATCTTCCCTATTAATAGAAGGGTTGATAAATACAAAACTCAAAAAAGTGATAATTTCTAATATAACGTTTATCTGGGCAATTTCCATATCTTCTTTTCTAAAATAAGAGAATTTCATATACACCCAAATCATGTTTTAAAAGTATTATATTTAATGCATCCAATGCTTATAGTATGTGTATATATGAAATTAATGATGAAAAAAAAGAATGAAATATGAAATTTTTCCATCCAATAATTAGCACATACATCTACATTTTTTTTTAAACTTTTCTTGAATTCAAAAATAACCCTTTAGCTTTACTACTAATACAAAAATGACCACTAAACTAATTATCTCTTTACAATGTCTGAAAAAAGCCCTTAAAAGTTTAAAATAAAAAATATTCTCTAAAAGCTCATTAAACTTTTCATTTTAACCTCGCGATATACAAAAACATCCCTTAAGCTAACTAATAATACAATACTAGCAGGGTACCCACGCGATGCGGTGGCAGGTACCCTGTGACTACTTTTATTAAATAATTAATTTACAAATAACAAAATATTGATTTTTTCCTTCAGCACAATCACCCTTCGTCAATTTTGGAGCCGATCTACCCAGTTCGACCCTCATGCTGCAATGGCGGCGACCGAACCGGTCGACCGACCATGGCGGGCAGCCTAATGGCTTTGTTTTTAAGTGTTTAGTTGAATAGCTTGATTAGATTGTACATTGTGGTTAAATGGTTATATATTTTAGTGAATGGGTGAATTAAATAGTATATTAATTGTATGTGTTGAATAGTGTTTTTTATTTGTATTTGTGCTTCAAATAATTTGTACACTGTGTTTTATTAATTATAGCTTTTATCAAAATGGTGTTTTAAATATGATATTAATTACAAATATTGACCGGTCAATATGTGTTGGTGTTTATGTATAAATTAATTGTATATATAGCTTAAGTAGTTGTATTTGCTTAAAATCTATACGAGGAAGTCTTAAAAAATTGGAAGAGAGTGGGTGTTGAATGTTTTATAAGTAGTGTAGATAACATCTAAACTCATTCGATTTATACTTAACACCACTTTAATTTTAGTAATATAACAAATTAGGGGATATGTTTGGCAAAAGTAACTGTTGGTGAAATTGAAAATGAAGATTGAGATATAGANTTATTGTTTGTTTAACAACATATTATCGTTATAACGGAATTTCGTAGCTAAAAATGAATGTTTTTGCACCCGCCACAACTAGCGGCCAACAATATCTCGTTAACTACATTCATAGGCAATTATTTCCATTTATAAACTACAAATCATGATAAATAAGTCTAAACCCAAAATATGCTTCAGCAGTTCAGCTGTTCGGCATGTGGCTCGCATTTAAGTTACATTTTGTCCTATGTTTCCAGGTAGTTTGCGCTTTTGCGAAACTAAACTTATACGTTTATATATTTCCCATCTGTCACAGAAAAAAGTAAGCATTCCCGAAACTGTATCGAGTGCAGTGAAATATATACCCGAACTACAAAAGGAAGTGGAGAGACTAAAGCATAAAAAGGAAAAAGTTCAGTGGTCTTCATCACCGACCATTAATGCCAAGCAAGAGCATCTTGCCATCAAGAAGAAAAGTTGCAAAACAAAAACAAATTCATTTTTAGTTTCTTCAGTGAATGTTTTGGGTGATAAAGAGGTTGTTATCCAGTTGATTTCCTCAACTGATCATACGAGCACGAACAAAGAGATAGGCTTCTTGTCGAAGGTTCTGGAAAACTTAGAACACGATGAAGATGGATTTGTATTGCTGAATGCGACGACCATGAAATGTTCCGGAGAAGGGATGGTTTTAAACACTCTTCATCTCCAGGTATATTTATCAATTTTTGTGCCTATAAGTTACAAACAATTTCGTTTTAACTTGCACTCCGTATTAATATAGTCCTTTAAGAATATCTTTCTCATTCTAGACAATTCAAGAGTGTGATGAAAATGGCCCCCCAAACGATCCAATTAACATGAATGGAGCAATACATACTTCTTTTTCAGCACCAAGATCACAAAGTAATAAAACCTGAATTTCTTTCGTTATTCCTTGAGTCGGATAAAAGAGAAACCCAAACATGAAATAACCTTAATCCATGCGTAAAGCTTTTACCCAAGAACAATCGGCCAACAAGAGTTTTGAAAACAATCGTCCAACTGGTTAGTTTTGTTTTATACAAAGATTATATAATCATGGGCGTTGCTGCATGGGTACATACCTATTGTTATCACTTACATTATTTTATTTGGAAATTTAGTGCAATTGCGGTACAAGACATGCATATAGGTTTAGAGGTACAAAGTGAACACCTTTTAAGTCACTAAACCTCCACTCGAATACTCGAATAATGGTGCAATAGAAATCAGGTAGCTAATTAGTTTAGTGAGTTATACATTTTAATGACTAGACTATGAACGAACTCATTAGGGAGTAGTCTCATCAGAGTTCAACCTTTTAGTGGAAAGCTCATCAGCAATGTCGTAGCTGAGCGAACGAAGGCCATGTGCAGAACCTTATACAATAACTAAATATGATAGTCAAAAATATACTATACCAACTATTGAAACCAAAAAACAAGTTATAACAAATATTTACAATATAAAATTATACAACGTTAACAATTTCGTTTGAAGTGAATTTTGAAGCCCCAAACATATAAAGAGGTGTACCATTTGAGGGCCCAAAAAATTTGAGGCCCTGTGCGGTAGCACACCTTGCACCCTCGTATATGGCTATGCATTCATCAATAAGAGAACTCATCAATGAGTATACTCAGGGAGCAAGCTCATCAACCAGCAATCACACTGGAACTGATTTCCTAGTAACTTGTAACAGTCTTAATGATCAAAATAAGCAATTGTCTTTTGGGATAAGGATATTCAAGGGTTGCTTGAACTGCAACTTAATGATCAAAATCAATTGGTCTTGATTGCTATGATGGAGGAACATTATGAGGAGATTGATTGAGTGATTAATTCTCATAATTCTGTTGAATGGGTATTAATATCTAAAAATTTATGAGTACTTCTACAATCTATCAAAATGTGTAACTTATGCTTTTCCACATGAATGTGTTAGTACCATTAACAGCAATTGTCTTTTGGGATAAGGATGTTCAAACTAGACTCTCGCTGTTAGGAGTGTGTTAATACCATTAACAACAATTGTCTTTTAGGAATGTGTTAGACACTCGCTATTGGAATGATTTAATGTGGAAGCATAAGTGTTGGAAGTTAATCTAGGTGTAGGCAGAATATGTGTTCTTAACCTCTTTTTAGCCGCATTTATCTTGGCTTTCTACGATTTTGACAAATGGTACACCTCAACTAGTGTTTTGGGATGAAAAATTCTCACAGCTAATTCCACTTAAGTTTGTAAACCAGCAATATAGAAGCTAATTTGCTGATCTTCAGGCAAGTCAACTTCATTAGTCAACTTATCATAAGCATTATGATAATCATCTTTAGTTCCTATATGCCTTACCTTCTTGATTTCAACCTAATGATCATCATAAGCACTACCAGATATCTGTAAAATAGCTTCCCTAGAAATTTTTCAAGCTACATTTTATCCAATTATTATAGTAAACTGCCTATGCCACATACTGGCAATGTCAACAAGGTGAACATAAGAAAGTTGTGCCTTATTTGCATCATGTTCATAGTCAAAAATAAAGAACTCTTCAAACAACATTCATACTCTTACATCTTCACCCCCAAATTTTGGAAATTTAATCTTGGTCATTTTTATGAACTTTGGTGGTTAAACTCATCTACTTACGCCTTCCCCATGTGGTCAAACTGCTTCGAAACTCCTCTTCCCCATAATACTCATTGAATCATTCATAACTGACCTTAATTCCAAATTCATTCCGTCCATTGTTGTTCTTACCATCTCAGCTAACTACTCATTAAGATATTGTTTTGTATTATCATCCATGGTTACAGTAGATCTTGTATTAGCCGACATTTGTGACTAATTATGTAGGTTTGGTGGTCACCCTCATGGCGTACAAAACATGCACAACTAAAAATAAACACATAAACGCCATGCAGACCAAACCCTAATACCAAGATCTCCTAATTAGGGATTTAACCTAAACCTAAACACGTATTAAGGGTGTTTACAGAACTACTTATTTGTCGTGAACGGAATTTCCTTCTTCTTCTTGATGCTATCTTGATCGACCTATATAAGGGTTTATGGAGATCAATCGCCACTTGGATGATGCCACAAGTACTGCACATGTAGAACTAGGGCTTCGAACCTGTGCAAGTTAGGCGAGTACGAAGCGGGTAAATCTACTAGAAGCCGATTGGGTTGACTTAGGGTTTAAGAAACCCAAAGTTCGACCAAACTCGGAAGAGAGGAGGAGAGGGAATTGGTTTTAAGGTTATGGAGGAGTGTATGAATTGTTTCTAGGTGCCTTTGTATTTGTATACGAAGGAGAGTTCTAGAACAATTAAAAACTCCGTAGACTTGTTTACCAAGTAAACAAGTTTGACAAAAACACAAATACCCCCTTAGAGGGTATTTGGCTGACCTTCCCTAAGGGTTAGGGATTGGTTTTGTCTTTTGTCATTAATTACCCTTTAATATTTATCCCTGATAAATATTATACTTTTTGTAATTATTGTTTAATTCCTTAAATGAAAATATCACTATAGTTTCATCACCAATAAAAAAACTATCATATTTTCATTTCCTCTTTATTTCCATTTCATTTCGTATCTCATTTCTTATTTCTGCTTACTTATTCTGCTTAGTGTGCACTGGTGTAACCCTTCGACATTTCTGTGAAGGCAAGGGTGAAAGTCGTTGTGTGTTTACACATAATCCTTTGAGACTACTATGTGCAAACACAGGAACATAGGAACCCTTCCACTGTACCAAGAGGTCACGAGCAAAGTTGTCAAAATCGCACCTAAGGCGCGCCAAGGCGCACAAGGCTCTAGCCTTTAGGCTTTTTTCAGATCGAGGCTCACGACGTGCATGAAGCCCAAAAAAAGCGCGCGCCTTGGGGGCTTTTTGCGCCATTGCACCTTGGGGCTTTTTGCGCCTAGGAGATTTTTTTTTTTTTTTTTAATATTAAAATGAGATGCATTGACCTTTAAGTCATAGGGTGAGTATTTGTATGATTTGTAAATTTGAATTGATTTTATGTTTATTTATTGATTGAAATTTTCTTAAGAAATTACTTTGTGTCATTAAGTTTTTTCTTTACGATATATTATCATGAAAATGTGGCATTGCGCCTTGAATATTAATATGTTGCTTGCTTGATATTTTGGGTTAAATTGTGAGCTTTGTTATATGTAGGCTACAAGTGTAGGACACCAAGCATATTTAGTCTCATATGGATTTTAATCTCTATATAGATCATCGAGCCAAAGAGTTAATCCATTTTAGAATTGATAATTGATACATTTTAATCTTTAAAATGGCAATATGTGTCATGTATTAAGTATTTTAATGTTATTTTGCATTATTTAAGCTACATAAAGGGTCACATATGTTATAAAAAACTTAATGTATTTAGTGCGCCTCGCATTCATGAATCCTGCGCCTTGGAATGCGCCTTGACTCGAGGCTCTTTGCCCCCCTTTGCGCCTTAGTGCGCCTTGAGCCTTTCAAAACATTGGTCACGAGCAAGACATTGCAGTACCTTACTCATAGAAACTGATTCAATAACCAAGTAGTGATTTTCCTGATTTAAGCTATGTCAAGCTAAGTCTTGACTCAAGCTCCACCAGGCTGAGTCCGAGTATATGATTGTATCTCTCCGCACTTTATAGACAATTTTAAGTACCGGGCATGGATATATTAACCTCGTAATAACTTCGTCCTTTCTTGTTCTCATGAATGATTTGCCTCCCATAATACCCCAGACACATTATCGTTCGGCTGAACGTTCCACAATCATCTCACACAAGGGGCCCAGGAAAATATCATTCTCTCCGGTCTATTTCAACTAGGAGATGCGAATTGCCATCTCAGCCAATAAGCCATATTTCCTTACCTCCCACTTAACTTGACACTAATACACAGCGTTACGTTTTTACACAATAACTGAAAACTAGATCTAGGCCCAGTGAAAAACAAAGTCAAATGACTTAAATCCTCAGGTCATTGGATAGTCCGTCACGATCAGAGAACACCATTAACTTTGTCCATGTGGTCGCTTCTCGGTCACAATCACGTCTAACATTTGCCCCGCAAATGCATGTGAAATTCAACATTTCGCTGAATGTCTCTTATTTCACAATAGCTACTTATATTATCTCAGTCTCCCACCATGATAACCAGCTAAGGAACCTTTTAGAATGAAATGTATCTCATTCATGGATTCAAAATATTGATGCGTGGTTTTGTAACCTTACAACAGGTTTTACCTCTCGAGGGATCTCAAGAGGCTTACAACCCCACCAGGCAGTGTACCTGACCGATGTAGTATAGGATAATGGTAAGTACGGGGGATTGATCGTTCTCTCACAGGGAATAGTGTTCGTTAGGATAGAAAAGCTAATCTAATAGGTCTAAAGGTGGAAGAAAGATTCTTTTTGTATTTTTATATTTTTTAGAAAGCAAGTAAAATCTCGGTTTTTAGAGGAATGGCGGAATTAGTTGCTAGTCAACTAACCCAAGTTTACTACTTATGCAAATTAAAATAAATAAATATCTAAACTATTGTGGTTGTGAAAACAATATGGAGAAAATTGTGTTTACTTAGATTTCATTACTCCCTAACTATCTTTTACCGGTTAATTGATATGTGTGTTACTTGTATGGATGAAGGGTGTATTCGTAATAACTCATGGAGTGGCATACATATCTCTCCAATTCTATGAGCTCTTATCTCGCTCTATGCCATGCACGGCACAACCATAGGGGTTGTAAACAGTGGCTCAAGCGTTAAATACTACCTTAATAGCCTAGTATGCCTTGGCTACATGTTTCCCAAGACGTTCAAGCACGTTACCATAAAGGAGGTTGGCCAGTTTTCTTGAGCCTAACATTGATGGAACAATCATTCCAACAAACAACAACACACACCAATTAACCAATAATTAATAGTTAGGTTTCATTAGATCTAAGCAAACACTAGAGAATTTAGCTACTCATGTTAATTGAAAGACAAGCAAGATAGATAGACAACCAACACATAGACATATTGATTGAATTAAAGTGAAGACTTATCTTTAATTAAGCATGAAGGTTCCAAGGGTTGGAAACCCTAAAAAGTACTGAAAATTCGTCTTTAGGGTTTGCCCTAAAAGTTCTCCACAAAGTTCCACACTAATGAATGAAAATTAGAAAACTATATATAGATTTTACACTCTGACGGCGTCAGGTTATAAAAGCTGACGTCGTCAGCTCGCCGGCATTAGGTTTCTCGGATGGCCTAGATCCACTTCTGACGCCGTCACCTTATAAAACCTTCTGGGCCGTCACTCTTTAAAATATAACCTGACGGCCTACTTTTCCTAACACCTGACGGCGTCAGGTGTTAAATCCCAAATTTCCTCTGGCCCGTCATTTGATAATTGGATTCCACTCTTTTGTCAAGGCTTTCTGACGGCGTCACCTTGTGACTTCTGACGCCGTCAGGTTGTAAATCTTCATCTTTTGAGACTTTGTCGTTTTCTTCACTTTTATGCTCCAAAAACGTCCGAATATGCATGTTTTCCAGAATTCCTCTCAAAAGTACCTAAAATGACAAAAGGAACGATTTAGGGCAATATTTGATCACATTTATACGAATTAGGGCTTAAATGACCCCCTAAAAGCGTAACACTTTTCGTCTCATCAAATATGCTAAGTAGAACCTTAGTATTGTCTTGTCGATTTGCATGAGTCTTTGACATTACTTTGATCCTCGACGATAGATTTAGCTTGTAATAGAAAGTATTGTGAGCAAGTCACTACTATTCAAATCTTTCTATCATTCCTAGAAGGACAACTGAGCCTTACTTTCTCGATCCTTTAGTATTATCTTACTGCAAATGAACCTAAGAGAGTTTTGGTACAAGAGATTTAGTCTTACTGGATGATAGACTTAGCTAAATCGCGTCAAACTCAGCTTAGCGTTCAACAATACTTCAGGCAGAATGGAATTCTCTAATAAATAAGAACCTTGACTCCTTGTTACTGATTTTCCTAATTCCCTAAGAACTTCAATACAAGCCCTTTGAAACCGCGATTTAGTTGCGTGTTTCCTGATTTGAAATCTCAGAGTTCAAATCTTTTAAGTCACATGGATTCTTTATTTGTTCAGAACAACTTAAGATCAACACATCATATTGTACCAAACTATCTTGCTCCATTGAGCAATGTATGTTTCGTTTAATGATCTTGACATCATCCTACAAAGCTTGGAATACTAGGATTTGAAATTCTTGTCATGTTTTGAACTCTTTAGTTAAAAGATCTAGAATTAACTTCCTGCGCTCTCGCTTCCATTGCTCAATGCTGAGCTGAACTCCTCTAATTAGGTTCAGTTAAGGACATTCGGCTTGAGCACATTAATAATTAATTCGATCAGTGTTTAACAAGTGCTAACCCGGTTAGTTGTTCCTCCCGCAAAGCATCTTTCAGCTTTACCTGAATATTCCAAAGCAGAGCGAGAATTTAGTCATGATGTACAAAGTCATGCCCTTTCGAGTTTGACTTACATATCTAAAATGTTGCCTGGCAGCATATGAGCTTTACTCAATCTCTAAATTTCTAGAAATACTATCACTCAAGCATTTCTCCTTTTACAATAAATCTTCCTTATCAAATATTTGTGACATTTGATTATATCGCACGTGGGTTTATGCCTTGATTCCATCAAGACTCGAGATAGGCAATACCTTCAAGTATTAAGAATTTTATGTTCTTAGATTCTAGTAGAAATGTTTTTCATAGTCATTCAATGACTATCTTCTTTGATTCATCTATCCCAGATAGATATCATAACCAAAAGATTCTTATTACTTTAAACCTCCATCCCAGATGGATTGCATCAAAGTCCATGATTATCTTTGAAATTCTATCCCAGATATATTGCATCAAAGTCTATCATATCGAGTCTTTTTTTGGTAAAAGGTTTACCCCCTGGTAAAATATTATTACTCCAAAAATCAATTTAAGTACAACCGATATTTCCCACACCTCAGCCCAAAAAGGTAAACCAAAAGCGCCTACAGTGCTTAAGACCTATAACTAGCATAAGAAATGACATGCCATACTTATGCAAAGTAACGAGAACAACCTATTTAAGTTGACCATGGAGACTAATTTCGTATGCATTAGCAATCTCCAATTGTGCATCAGTCAGACCACAATCATCAAACTCATCATCATCATAAGGGTTTTGGTTATAGGTGTCCTCATTCCACTGTTCCAATAAGCTCTTTATGCCCACTCCAATACCACTTTTAGAACCATCAAATGCCATAAAACTTGTGGTTTCATTGTAGTCCTCAAGAACTTCATATCATCATCACTATCACTATCTGAAATAACCAAATTATCAACCTTAGAAAATGGATTACCAACATTTTTTTTCATCTTTAACCATGTCAGGTGTTCTCTTCAAAGAAGCACTAGCATTGGACGAACTGGGGATGTTTTCATTATTTTTCACGTGTCCTCCATCAACATTAGTAGCAGCATTACCACTAGATGCTACATTAACAGCAGCACCTTCATCTAAATCATATTGAGCTTTGAAGCTCACGTAAAACATTTTCTTTTACTTCACTAATTCTTTAAAAGAACCCATAAAGTATGATCTTCCAAAACTAGACCAGGTAGATTGTTCATCACTCGCAAACACTTTTGGGAATACCTTAAGTTTTATCAAAAACTTGCAAAACTATAGATTTTAAAACTGTGCTAGATTCAAAATTTAAAAAATGATAAGAAAAGATGAAAATCTTTTAAAGTTTTAATATCCTTTATTTATGATAACTCCCAAAGCAGTCATCTAAAGGATAAGCTCCGAAGAGCAAATACAGTTTTATGTTAGAAGTTTAAAAGTTTTATAAAATATATCCCAAAAGGTAGTTAACTTAATGCATATCCCATACCTCATTCTTTATGCAAAGTTGCAAATTGTTAACTGATGTGCGCCCTCGGGACGACATCGTTCTGCAGAGCTGCACTTCTCGACTAGGTTGTTTCAACAACTTTAGTCAAGTGAATCCCTTTTCACTTTAAGTAACTCTTATGCACCGTCGCATAATCGATTAACTTTAATGATTCTTAAATAGACCATGTGTTAATAGAAGTGATTAATAGACAAAATCTATAATCATAGATTTCTCACAAATAGACATACATTGTCTATACGATTTCTCAGTACCTCAAGATACCTGGATTTTCATCAAGCATCTTCTGAGAAAAATACTTGCATTCACAGTAGCACATCATCGCATAATCTCCTCCTTGTTCGAAACGCCGATGAACGAGATTATTATCCAATAGTTATGGATAGCCACGTTACATAAGATGATTAAATCCCCTTCATCCCAACATGATGAATATTATGAGATAGCTCGCTTCCATCTAGCAAACATGTTAAAGTTATTTTGTGATCTCATACCTTATGGTATTCCTTGCACGAAATACATGTTGCTTCTCCTTGCAAGTCTAATGATCTAATCATGTGACCATGACATATTCTCCAAGACTGCACATACCAACTGCCACTGGTATGATGGCGGGTGATAGATTATCTCAGGTTTTATCCAACACATACTTTTCTTGAAGAGTTACCAATCAAGAAAAATAATATTACCAAGCATAAAACTCAAGAACAAAATATGTTGTTATATCATGCATCTACAGTTTTTAATTAAATACAATTAGTGAAGTCAATCATTTATCCTAATTACTAAACCCTATCATTTCGCCCATTACACTAGGATCCAGAAAGACCATTACAAGGCACCATGGTATGGCGTAAACTACCTTGTAACAATTTTTAGGTAGGTACTTTACCGCCACCAATTGCCTCCGATAGATAATTCCTAGGGTTGAGACTCTCACACTAGTTGGGCATACTTAGTCTCCCATGTAGCCTTAGGTCCGCTAAGGCAGAATGCCAATAGACTCTACCATAAAAGCCAGCGGCCTTGCAATTCATTGGCTTCTACTATGGCATATGACCAATGAATCCAATAACCACCTCCTCCATGTCAGCCATGTACCCACAAACACAACGATATGGCATTACCTGCTTACAGGGTCTGAGCTAAGAGAGTGGAACAGTTTATGGATGGTGTACTAGTATAAGATTACACATAGGGTAAGACCTTAAACTCATCAGTTTATCAGTCTTTTTTTTATATCCGGTATTAAAACGATTTTCTTCAAAACTCCTTTTACGTATGAATTATGCATGAATTCATACCATGATTTGTTAATTCATAAAAACAAATTATTTTATAATAAAAGTGTTTAAACTCGGGTTACCCTTCCAAAGATATTTTTTTAAATAGGTTCAGCTAAACTCATGACATTACCCTTTTATTACGTGAACTCAGACTCGGTGCTTAAGTACACCCTTTTCCCATGACTTTAGCAGAGACGTCCAGTTTTATTAAGTCTCTAAGCAATACCGGTTGACATATTATTTAACATATAACATGCCCATCAGACTTAAATAAAACACTGTTGACAGAGCCGAGCCATTATCTAATGTTGTCCCGAGATCCACTTCGAGAATCAACTTGGGTCTTTTCCTAACGGCTAAAACATGATAGTGACACAATGTATGAAGGCTTCTTCAGACTCCGTGTCTTCTCAGAATCATCCATGATACTACTTGCTGCTAGTGGATCTGCATTCCTCCATGCTCCCACTGAATCTCCTCTCTCTTCGTACTCCATAGGTAGTTCTACCTCATGTGGGCTTCCTTGTGTCATGTCTGTTTTGTTTTTCTATTTATTTAAAAGCTACTCTGCGTTTTAAATTAAAATACCCTCCCTTAAACCCAAAGCATAAAATATAAATGTAAACTACTACTACATTACAACCCCACAGTTTATATTTTGGTCTATGGCACATGGTTGCATCATGCATGATTTTGAATCTAAGCAGGAAAGTACTATTTAATAAAGTATAAATGAAATACTTAAGTTATGATTCGCTAGGACCTACTACACATGCACATGCACCGCATGTCTAGCCCATCCTAATAGTACATGTGAATTATGAGATTCACCTATATTGTTGCATACTAATGCAACCTGTCATTCCCTTATTGCTTCATATTTTTGCTTTAGGAAATTTTACTTTTAAAATTTTAAACCCTGATTAAGGTTTCCAAAAACTTAAAAAATTTTAAAAAGTTTGTGACTTGTAACAAGAAACCCTAAGTCACTATATTTAATTTTGGAAAACTTTTATTATGGACTTTTTAATAAAACTTTTATTTTCTAAATCTCTGGTTATGACTTAAAACCTTAGTTTCCATATTCAGGAATCTCATATTCCAAATTAGGTAAAAGTGAATCTTTTATTGCTTTATGAAAACCACCTGCTAGGTCATCAATATTCGTGCCGGTAGATTTTCATGATCCCATCTAGCTAGGATCCAAAAAATTGACCCATATAGGGTTTAGGTAAACCCTAGTCAGTTTTTCTTGTTCCCTTAGCTATCTTGTTTACCATATGATTTCAAATATAGTTTTAGTAAAGTTTACACCTTTCAAAAAATACTTAAAACTCCCAAATCATACAAGGAAAAATGAAAGCTTGCAAAACAAGAATTAGGGTTTTGTTTTACAGATTAGGGTTTTGCGATTAACCTAAATTAGGGTTTCCTTTATGTTTTAATCACGGATACCCTTGTATCCTCGTAAAGCTGACAACTTTAGGTTTTAAAAAACCATAAAGATTTGACCTTTATGTGAACTAAAACTTCAAACCCTAATTTGATTACAAGAACCCAGTTTTTGTAATGACCATGACAACTTTAATTAGGGTTTCTAGCGTTTGATCCTGGATACCCTTGTATCCTCACAGAATCGACTCCCCAAGGGTTTAAAACCTAACTGATCGATACTGTGTGATCAAACCATATACACCCTAGTTTGATCACAACAGTGATCAAACCCTAGTTGTCATCAAATCGCATAAACCCTAAAACCTTAATTTGCATAAATCCCTCCAAGCAACCTTATAACCAAAATCAAGATTTGATTTTTATGACAAAAGAATCAACTACTAATAACCCTATCTAGGTTATATATGATTAGGATGGTTCTTATACCACTGTAGGGTTTTGTTAGTCACTCGCATGGCGTATAAAACACTTACAGCAAAAAATAAACACATAAATGCCATGCATACCAAACCCTAATACCAAGATCTCCTAATCAGGGATATAACTTAAATCTAAACATATATAAAGGGTGTTTACAGACTTACTTACTTGTTCCTACAAGATGTAAGCTTCTGATTTGAACTACAGTATGTTGCGTGCACGGAATCGCCTTCTTCTTCTTGATGTTATCTTGATTGACCCATATAAGGGTTTATGGAGATCAATTGTCACTTGGATAGCGTCAGGTATCCACCAGTACTGCATAGGTAGAACTAGGGCTTCGAACCTATATATGCAAGTTGCGCGAGTACGAAGCGGGTAAATCGACTAGAAGTCGATTGAATCGACTTGGGGTTTAAGAAACCCTAAGTACAACAAAACTAGGAAAAGAAGGGGTGAGGGAATCAATTTTATGGTTATGAAGGAGTCTATGAATTGTTTCTAGGTGCCTTAGTATTTATAGGATACCAACGAGAGTTCTAGAACAATTAGGAAACTCCCTAAACTTTTTTACAATGTAAACAAGTTTGACAAAAACACAAATACCCCTTTGGAGGGTATTTGGCCGACCTTCCCTAAGGGTTAGGGATTGGTTTGTCTTTTGTCATTAATTAACCCTTAATATTTATCCCGAATAAATATTATACTTTGGGTAATTACTGTTTAGTCCATTAAATGAAAATACCACTATAGTTTCATTTTCAACAGAAAAAACTATCATATTTTCATTTCCTTTTTATTTTTATTTCATTTCGTATATGTTTTCTTATTCTCGCTTGCTTGTTCTGTTTAGTGTACACCGGTGTGACCCTTTGGCATTTTTGTGAAAGCAAGTGTGAAAGCCGGTGTGTTATTTGCACATAATCCTTTCAAATTAGAGTTCACAACTCAATCTTAAAATTGAAAAAGAACTTAATATGAACTTTAATTTCAATTAGGGCTAATATCATAATAAAGCATCATACTTGTCAAAAAAAGCTGTTTTGAGTAACGAACTTTTTTTTTCCTCGTTGATGTACTGAACTTGCATTTTATTGCCAAAAATACGTACTTTTGTACTTTTTCTCACCTTTTTATGACATTTTTGCCCCCGACTTTTTAATAACTTGAGGGGCAAAAACGTCCAAAGTACGTAGTTTTGGCAATAAAATGCAAGTTCAGTATACCAACGAGGAAAAAAAAAGTTCGCTACTCAAAACAGCTTTTTTTGACAAGTATGATGCTTTATTATGGTATTAGGCCTTTCAATTACAGCACAAATTACGGTTGACTACCACTTAACAATCAAAGGATTTAAACACAAATGTGTTGTACGTGTAATCAAACCATAAATGTCTTGTAATTCTACCAAGAAGCAATCTAGAATCATAATAAGTCATCATAACCGTTAGCGACTTCGCCAAAGAAAACACCATAATGTACAACTTCGTCGAAATTTTACCGGAGAAGATAATGTAATATGATCAAAAAAATTACTGAAATGCAAGATTGATACCAAATGATATACTTCTATAGAAAGTATTTAGCATATGCATCTTTACAAATTTTTCATACGATCGTACTTACTATGTTAATCAAGCATCCATATTTCATATACGTCTATTGGTGGCTGAACTCTATGGACCATGCATAGTTCTTTCAATATCATTATAGTTTATTTGATAAAAATAATTCCAATTCTTTGTTATGTGTGGCAGGTACAAGGGGATCACAAGATAGGCGGTGAAAAGTTGAAGGAACAAATGTGCTCTTTCTACCAAAATATATATGGAACTTTACTCTAATGAGGAGATAAATATAATGACCTTGTAATAAATAAGAAAAATGATTCTACAATGTGCTTAGAGAATCAACATGAGGACAATCATTCTTATATTTCATCATTTTATGTAATGGAATATAATGAGGATGCAAACAACGCGTGTTTGGCCTAGTGTTATGCTGTAAATGTGTGTGTTATTCAATCATATGGTTTTGGGACAAATGTGTGGTTTAAATATATTTTTATGGGTGTATGAGTTTGCCTTTCAAAATAATAGGATGAAGATGTATTTATTAAAGCAGTCCTCACGACCACTGTTTAGTACAATGCTGCGTTTGGTGCATGGGGTCATGCTTGTTTTTCTTTTTTCTAAATTATGTGATGTTAAATTTGTGAACCTTTTGTAAATTTTTTGTTACATTAGTGGTATAATTTTAACTAATCTAGTTTTTAGGTGACGAACTGGAATATGCTACAAGTATTTCAATATAATATAAATTATAACATATGTGTTAGACCCGTTTCCCGGGTGTTGGGACCCCACCCTAGATGAAATGGATAAAGGCCTTATGGGATTGGCTAGTCAATAAATGTAAGGTTTTAGTCAACCCGATAGTCAATTTATAGTCAAAATATGACTAATGTAGTTATATAAATATAATGCATAACAAAAGGGGCCTAGGTTGCCAAGTGTTATATATGTTATAAAG

AabHLH22

TTTGAATTTGATATTAGGGTGTTTATTTTCACTTAATGAGTGAGCGCAATCCTTTAATTCATTAATTGGGAGGTCCACTAAAACCCATTTCCCTCAATTAAGTGGCTAATATGGTAACATGTCAAGGCTACTTAATTTAGTAAACAAGCCCTTAATCTTGCGAAAATATCATAGCGTCTGATTATTGGTCAACATGTATTCTTACGTTAGAAGTGTTGTGTTCACTATTTTCTCATGATGGCTTATATATCAGAGTACCTAAAGGGTCAATGTAATGTAAGCCAGTGAATATATATTCATTACCAACCTGAAAATAAGCTAAGTAAAATTAACCATGAATTCCATGAAGACCGATATGAAAACATCAAAACAAACGTACTAGACTGAAATACATAAGACGTCAACTTAACACAATAACGTACTAAAGACACGACTAACATTTAATGAAACAAAATGAAAAGTCGTCACGCTAATGAGATAAATTCCCAACACCCGTACCCCACGACCTAATCCCTATCTCTTCCTTTTCTCACTCTATGTCCATCACCATTTACTAATTCTATACCATGATTAAAGAATACCATATATAGGATTATTGTTTTATTCTTTTTCAATAAAGGATCTCCCCCCTTTGCCAACAAAAACCATATAAATAGACCACATGTGACTTGGAGGAAATGTAAACTTTATAATAATAGCATAACAAAAACACCCTTTTTCTCCCAAAATCTCTTAATCACCATTGCAAATTTCTGCAACAAGATTCCATTCCATGGAAGACATTGGTGATGAATACAAGCACTACTGGGAAACCAATATGTTTCTCCAAAACGAAGAATTCGATAGGTAATACTTACTAACACATCACTTATCAACTATACACACAATCCAGTATAATCGGGTTTGTGCCAACTAACCTAAACTCTGTCTGTCTGGTCAGAGTTTTCTTTAACATACTTGATATAGTAATTGTTATGCAATTTTTGACATGCAGTTGGGGAGGATTAGAAGAAACATTTTCAGGATACTATGACTCGAGCTCGCCCGATGGAGCGCAGTCGTCGGCAGCCTCAAAAAACATTATGTCCGAACGGAATAGGAGAAAGAAGCTCAATGATAGGCTGTTTGCACTTAGAGCCGTGGTCCCCAACATTAGTAAGGTGTGGACCTCCATACTCGTTTAATCTTAAAATAAGTGGAGTGTCGCATCACTTCCTTTATTCATCTTTATGCATGGTGCAATATGGCACATTTTATGCCACCCAATTTTCATTTACTATAGTGAAAATTTATATTTAGTTTGGCAAGTTAGGTCTGATCATTTAAGATATTTATTTATCATTTTAGATGGATAAAGCGTCTATAATTAAAGACGCAATTGATTATATACAACATTTGCATGATCAAGAGAGGGTTATTCAAGCGGAAATAATGGAACTCGAGTCGCGAAAATTGGAATCCGGGGTGCTTGAATATGATCAAGAGATGGCGTTTATGTCGACGGAAAATTCTAAGAAGAAAAAGATTGAACAATCTTTTGATTCTAGTGAGTCGAGGGCATATCCTGTCGAAATACTTGAGGTAAGTGTTTATTTCGACTTCAAACAATATGTTCTCGTATGTTACATACTTATATTTATATTATGGCATCGTCATATTTATATGGTTTGATAGTTAAAAGTGTCGTATGTTGGTGAAAAGACGGTTTTGGTGAGCTTGAAGTGTAGGAAAAGCAGAGACACAATGGTTAAGATTTGTGAGGTTTTCGAAACATTGAAACTCAATGTTGTAACGGCTAACGTTACTACTTTTTCTGATACCCTTTTCAAGACACTCTTCATTCAGGTGACTTCTTTTAACTTTACTTAACAAAGTAACTTACTTTGCTTTATGATAATTTTGATTGATATTAGTTTGGATAAGATGACAAGCTTCTTCTCTATTATATTATTGATTGTGTTTGGTGTGATTAAATAATGAGATTTCATATTTATTTATAATAGTTTATTACTAGTATTTGGAGAATAAGCTAACATAATCCTAAACCTATTCGACCATAAGTTTTTAAGTTTGCGCCTTAACCTATCATCTAACATTCCCCTGCAATTTGAACGGGAGGTTTCCGAACATACAAATCGGATAAAATGTTGAAATCGCTTTCTTCTTCTCTTCTTTTATTTCTCTCCTCTTCTTTGTTATTCATTTGCACGGAGATGGACTTGTATTTGGCAACATAATTGTAAACTGCTTCACGGTTTCATTCATTTTTTTATGACGTTTGCTGTTTCTTTTACGTCATGGCTTGCTTTGCATTTAGAAGGGAGCGACAATTTAGTGTCAACTTTGTTGTCGACGACAATGATCTTCAACAAGGAAGCAATTTGATGAGAAAGGAAAAAAAATATAGGGAAAAATATCTAAAAATACATTCAACTTACCACTTATTACTATTGTATGCTATGAACTTTCTATTTTCCTATTGTATGCACAAACTTTCTTAATTACCCTATTATAACAATATTACGGGTTTTTTATGACGTGACAATACTTAATCACACTTATTACGGTGTAATTAATAGAATAAAAACCCGTAATATTGTTCCAATAGGGTGATTAAGAAAGTTTGTGCATACAATAGGAAAAAAAAAAGTTCATAGCATACAATAGTAATAAGTAGCAAGTTGAATGTATTTTTAGACATTTTTCCCAAAAATATATGGTTGATTGAAGGCAAATCAACATATATTGTGTGTTTAGACGGCTAGCGTTTAGGGTTTATGCATTGAATACGAAAAACAAAAACTAAAAAGAAGAAGCAATAAGGTGAAAATAACTCGGATTCATCCGGATCACCGGCGAGTAGCCGATGATCGGATGATAGCAAATTTTGTAAGTGAATTTTACCTAAAGCTCAGATATCATGCACGTAAAGAGAGATATTATTGATTGGGTTTTGTGTGATTACATTATTTTATAATAGAATATGACTTAAAAAATAAACTAAGTAACTCTAAACCTAAAAGACTTAAACCTTAAGTCTTTAGGTTTGATCCTTAACCTACCATTTAACAGCTTCATGATGTAAATGTATCCATTTATGGAATTGTCAATGATTTCGTGGGTGGTTCTAGAGGATTATGGTAGTGGATGAGTATTAAATTAGAAAGATATAATCTCTTACCAGTAATAATTAAAAATATCTCATTTGTGTTAAATATAATGATAAGTGTGTGCTTAGCTTTGTTTAATATGTATAGGTAGTGGTAGGGTATCCAAATACAATTATTTTACAAAAGGGAGAAGACGTAAAGTAGAATATTAATACAAATGACTCAACATAATTAAATACGTATGAACTAAAATCATGGGAATTTTAATTTCTGGTTTTATAATTCATGTACCACCATATAGATACGAGTGCTAACATTAAGAAGAATCATGTAAAATAATGTTGTAGAAAATTTATTCATACATGTGGTTGATAAATGGCACGTTTATGATATCACCAATAATTGAACTCTGGAATTATCAAGATTTTGTTAACAGTGTAAACTTGGACACTAAAGATTATGTTGCTCACAAAAAGGTGATGTCGATATATCTAAGGGTGAGCATCGGTCCGAGACCGGACCGGACCGGACCGGACCGACAAGACCGAAGACCGAATTAAGGTAAAAACCCGGACCGAGACCGAGACCGAATGTGGTCGGTCCGGTCCGGTCCGGTTCGGTCCGGTCCGGTCTTCGGTCGGTCCGGTCCAAATAGGCAAGATTTGGCATGTTTTTTCGGTTTTTTGGTCGGTCCGGACCGGACCGACCGGACCGAAGACCGAATTAGCCAAAAATCCTAGACCGAGGACCGGACCGAATGTGGTCGGTCCGGTCCGGTTCGGTCCGGTCCGGTCGGTTTTGTCGGTCGGTCCGGTCCATCCGGTCCGTTTGCTCACCCTTAGATATATCTATTTGTCTTACTAGTTATAATACCCGTGCACGCACGGGGTTTTTCTAGAAAAATTTAATTATATAGTATTATCGTTTAAAAACAGAAAATCTTATGTATATTTTAAAAAGTTAGAGTATAAACATGTGCGAAACTAAGAAAAATAGTTAGCAAAAATTGTAAATATAACTGTGGCATCGACCTTGTATTTGATTCTTGAGCGTGTCTCCTTATAATGTTATTGGGCCAAAGGTTCTTTGATCACATGGGTTTACACATAAATCAAATTTATTTTAATTTTTTTGTTTATTATATGGATATATAAGAATTTACCAGTAAAAATTATGATATAAGTACAACTAATAATTATGCATGAGTTTTTCATTAAGTATTAATATCAATTAATATTTTGTAAAATTTTTATTGAGTAAAAGATTTAACTTTGTTTAGCTGACATAGATGTTAATGTTGAGGTTGATTTTGTTGTTGAACTTTAACTATTAATATCATGTTGTATAAAATGTGCGATTTTAGTAGCACATAATGAGCGTTGATGGAGTTAAAATACACTCCATAAAGTTTTTTAAAAATATTTTTCCGGATATCCATATATAAACTTTAAATAATAATTACATGAGTCGTTATAAGTACAATCGCGTATATTTATTTTCATATCACAGTAACTCGTACTTGACATTTGATATTTATATTACCGTTAATAATAATTTAACTGTATTTAATATTCAAACATGTGTACGTATAGATTAAACTTTTAAATAGGATAACATGAGTTCTTATTTATATTTAACGTACATAGTAAAAAGATATTTTAATAGATGAAAGTTTAAAACTAATATCTATTTTAAAAGTATTTAACGTATATTAAAACTAATTCGTACATTTATAAAATTTTGTAAGTTTAATAGATGGAAGTCTCAAACCAATACTAATACTCATGTAATTGTAAACAAAGTTCATATTATAAAGTGTCGGGGATATAAAGTGTAAGTGTTATTTGTTTATTATCTAAAGTTATAAAAAAAACATGGAGATTTATAATTTTACACTTAACTCCATATAATATGTTTAAAATTCAACCACTAATGTTTTAATAATTACCTCCTCTTTTTTTAAGTTTTTATTTTCATAAATTTGACACCTATATTTTTATGTGTTTTTTTCCTTAATCAAATTCTCCACTAAAATTATTAAAGTTTGACCAAAATCAATTCAATTTTGACTTTTTATTGGTCAATCATATTGCTCGATTTCGCCTATGTGTCAAAATCGAGCATTCCTATTGGTCCAATTTTTTTTTCCACTAAGATTGTTTTGTCAAAAAAACTCCGAAATAATGTAATTCTTTTATTATATAAAAGATACAAATTACCAAGAGACATCAATCTTATTATGAAATATGTTGTTTCTTGTTTCAATTAAATTATTCATGAGTCTTTATACTTGTTTTAAGTTTCATTCAAAAGAAACTCTTTTTTTTTTCTTTTTTTTTTTTTATGTTTTCTTATAGTAAAGAAACATACTGCATAAAATCATGATGGTGCTAAAGCCATCATTGTCTATAAAAGTATAAAACAAATACAACACGTTTATTTGAAGTTGTCAAAAAATTAAATATGAACGCAAACTTTCAACTATTTATTGTCGTTTTACTCGTGGGCATCTTTGAATGTTTCACGAGTATTTTTAAGACACAGCTTTCACACTATAAACGAGTTGTTTGGGTGTAGGCGTAGCTCGTCATGTTTCGAAGAAATAAAAAGTACTAGTAACTTTTATTGATAATGCACAACTGAAAGCAACTTTTATTAAAATTGTTTTCTTTATTTTGTTATCTTAGTATATGAATTTATATGAAATATGTATATTCACTATCCCTAGAGTCCTAGACACTTAAAATTTGTATTATAGTTTTTTCAACTAAGGCACCACAGTTAAATGTATTGATGTGTATGTGTTACTGCAGGCTGATGAGGAAGAAATTGATCTCCTGAAGATACAAATCCATACTGCCATATCAGCTCTAAATGACCCTCCGAGTCCAATGAGCACCTAGAGGCTAGGCATCCATTTCCGTCTTCTAATTTTAGCTTGAAAACGTTTCTGGAGGTATGCGGGTCATACATGGACATGTTTTTCATAGAAATATCCGATGACCCGTATACTTTATGGGAAACCGGAAGAAGAAGATGATGCAAACGAATAAACGAGTGTATCCATCCCCACTCCCTATCTTTTTAGTGTTCGCCTTGTAATCTAGCTTTTTGCCCTTAGTGACCTAGGAGAGTTTTTCTTCTTGATTTTTTTCTGGTTTGTTTTTCCTCGAGAGGATACTTTGTCCAGAGTATATCATTAAGATGGTCTCCTATAAGTTTCAGCTATAATATTTGCATATCACAGTATTAACTATTAAGTGTTCAATGGAAAGTTTTTATTCTGTTTTTTAAAGGACTAACGTGCACACAAGTTCTTTGACTCGTTTATTTTACCATCTAAAAGCTAACATCAACCCCAGTTGAGAACTCAATTTAATGAACTCAAAAACCTGAAATCATAAATGCCAGTAATAATTTATCAGTGGCT

AabHLH23

TGCGGTGTCGTGGCGCGGTCATGACGCCGACCACCTCAGCCTTAGCGCAGCCCTCCGTGATGTTGTGATGATACTTTGAGCTCTAATGGCGTCGCAATGAAGTTTTATAATTTGTGATTTCTATAGGTATGTATAGAGTATTTTAGTATGAATAGTAGCTTTGTAATTTTAGCATAATTTTTTAAGCATCTAAAAGAAGAAAGTGGTTTTAAGTACGTTTAGGGTTTCTAGATGTTAGAACTTATATTTTTAAAACGAAAAGCTTGAATCATCTAGAGGGAGATGTAAAGCGAAAAAGCATTGCTTCCGATATCCCCTTTTTAATCATCCCTTTTGCCCTCACTAGCGTCCAAATGCTATTCAATCTCACGACACTTTGTACACACATGCATCACATTGTCATTTTATCGAACCATATAATATGTGTCTATCAACGATTATTCATCTACTAATACAACCTTCATGTTCCCACAATCATCATCATCACAAATCCCTCTAATCATGCATCCTCATCATCATAATCACCAATCTTCATGGACAACTCTATCTCAACCCTTCCAACAATACACCTTCAATCATGTTCTTCCTCAATCATCACTACCTAATGGTGGTTTGTTTAAAAGGGTTGTTGATGGTTTGCAGTTTGCCTATGAGGGTAGTAGTAGTAGTACTACATCTCCATCGGCTGACCATTATCTAGGGTTTCAACCTGGATCCAATACCCTTGGGATCCAAACTGATACGACTAAGATGACAACTCAAGAGATGGCGGATGCTAAAGCCATAGCTGCCTCGAAAAGCCACAGTGAAGCAGAGCGAAGACGCCGAGAACGAATCAATAATCATCTTGCGAAGCTTCGTAGCTTACTTCCCAACACCACCAAAGTAAGCCTTACTTTGTTCTTGTTTTTTCTTTAGCTAAAAAGTTTAATAATAATAATGGACAGCCTCTTGCTTTTACCAATTAAATAAGTATATAAAGCTGAAATCCTGCAATCATATATATCCAACATTTTAACTTTTAAGTATGTACAAACCATCATTCCATATACAAAATCTAATGGTTAGATCGATTATAAGTTTGAATGTATACTGATTTTAGATGACCCCGTTTCTTTTGAACGGCTTATAGTATATTATAAAAACACTAGCAAGATGGTAGGGAAACAATTACAACATGTTTTTACAAGAATCGAACGGATCAGGGATCCAATCCATCACAACGGTTTTACTCTCCTGTTCCTACGTGAAATCCAAGAACAAGAAATATAATCTCCTAGTGTATCTTTCCAAACAAATGATCTTATAGTGGTTGATATAATTTTAATATAAGCGCTCAAAAACCTTTAACACCGAATACATGAGTAACTTCATAATTAGAAATATAGGCCTAAGTATCAAATTGTAGAATACACAAGGTTTTTACATCATAATTTTTTCTTTAGCTAAATACATGAGTTTCCTATAACTATATTTAACGCATTTTAAATTTTATTTCACACTCATACGATGTGTTCATATGATGTGTATGATTTGCATGAAGAAAATTTCTTGGTGTGTTGTTCAGGGATATAATAAGCAAGACTTTCAAATTAACACGATTGTGTTTAAATTATATACTGTTTGATACATGTCTATCAAGTTATCTAATTAAGGATTCTAATTAAGGATTCACATACTGATTTTATTGTTGTTGCAACTATTTAGACAGACAAGGCTTCATTACTTGCTGAAGTGATACAACACGTGAAGGAGCTCAAGCATCAAACTTCCATTATAGCGGCACAAATTCCAGTCCCTAGTGAGATAGATGAGCTGGTCATTGATAACACATCGGATGAAGAAGGAAGAATTGTAATCAAAGCATCGTTATGTTGTGATGACCGGTCAGATCTCTTGCCTGACCTAATCAAGACTTTGAAATTGCTTCAATTGCGTACCCTAAAAGCCGAGATCACAACAATTGGAAGCCGTGTGAAGAACGTTTTATTTTTAACAGGAGAAGATCATATGAATAGCAACGAGGATGAAAAGGTTGTAAATCACTGGGTATACTCTATTACAGAGGCGATTAAGGCAGTAGTTGAGAAAACAAATGACGGAAATGATTGTTCTTTTGCAAGTATTAAGAGGCAAAGAACAAATAACATTGATATATTTAATCATCGTAGGCGTCTTTAGTCAAATGCAAAAAATGACTAACCTTTGGGCTTATGGTGAACGTTAATTTGAGACCATTAAAAAACTTGAGATCATGTCAATCACTCGTAGTCAAAATTGTGAGATTTAGATGTCCCTTTTTTCTTATCTAAATCCCGGATTGATTGATTATCTAGTACTCAAGTTTTTTGATGGTCTGAAAAGAACTCACCCCTTTCGCTTACACGCCTTCATAAATATATGTAGTATTACTTGAATTATTGGGTTTTAAGTCCTTGTTTCCTCCCCTTTTGTAATATTTCCTTCTTTATTTGTGTAGTCTAAATTTATCGAACGTCCCCGAAGGGACGGGGTACTCTATATTTATGTGAAAGCTTGAATTGTTTTTTTAACGGCTGTGATTTCGTTAAAGTTAAAAATCTAGCAAGTTGGTAGAGGGCAAAGAGAACATATTGCAAGACAATAATTAAACAAGCCATAGTTGTTGATTTAGCTAGCTTTCTATTTCTATGTCGAATACACAAGTTTGATAGGATTTTGATTTTGATTTCTTCACCAAGAATGTCTTTCTAGGCAGTT

AabHLH24

TTAATATAATTCTCTCCTATAAGTGTTAGCACTTGAATTTGATATGGATTTGATCTTATTTCTAGATTAATAAGGTCAAAAATGTAGTCCAAAATATACTGGAGTATTGTTTTAAACTAACATTGCTTAGAATATCAGTCTCCACAGCGTATGATTGGTCCTTTACGCTTAATTGCTTTGACCATACATTTAAGAACATAGTAATAAAAATTTTATGTAAAAAGAGGCACGTTTTTGGAAGCAAGTGAGAAGAAACACCGTTCATATATAAAGACCGGCCAGGAATTTCTATTTACACGTGCCTAAAATGAATAAACAATGTTAAATTTGTCTTCAGGTTACAATATATAACACCAAGGAATCTGTGATTTTAATCACCAATAAGAATTCCAGAAGAAATATTCTTATCTTTTCAAAGGTATATCACCTTCTCTCGTCAGTCTCACTAAAACTCTCGATTTAAGATTAAATTAAAGTTGTTTTTGATAGATATAATGAGGTCTGGTTGTATACTTGCAGGTTACTTAACTTTGGGTTGAACATATTAATATCCCGAGTTTCTTTTTACTTTTTTTCTAACGGCAAAAGAAGATTTTAGTGAGCTTATTAATCTAGCAAGAAGTGATGAATATGTCACCGGCATGGCTATCAGAAATGGTAAGTAACAAAATAGTAAATTGCTAAATAATTGTAAACTTACCAGGAAAGAAGTAAATATTCTTAATTAAGTTATTTGTTTGTTGATCGTGTTATGGATCTATATTGTAGGAAATAGAAGATCCAGGGTTCATGAATTATGATCAGATGAGCAGACTTTGTGATACAAGCCTTGATTCATTCTCCCCAGAAAGCTACGCCGAAAACATGACGTTTATCGATCAATCTTTCCAGGCTCCACAGCTCGAGATCGAAATTCCTAACTATCAAGAAAAGAGTAGTAGCATTGATAAATGTTCTCCAAGTCCAGATACCCTTGTTGCTACTACTCTTCCCTCATCCAATACTTTTACTATATCTTTTGGAGATCTAAAACCTAAAAGCGAGATCCTCCAATTTTCTGATCCGCTTCCTGGTGCCATAAAGGTTCCAACCATCCTCAGGAATCCACTTCAGGCACAAGATCATGTTTTGGCAGAGAGGAAGAGAAGAGAAAAGTTGAACCGACACTTCATTTCTTTGTCTGCCATCATTCCTAACCTAACGAAGGTACTAATCTACCTAAAAACCTTTTAATTTATTCGACATATTGTCTGAGTTTTCAATCATGTTGTATTTATCCTATAACTCATTAATTATATATTGGGAGAATGTGAATGAACTGACGGCTATCACCTTTCCTCTGCGTAGATGGATAAGGCATCTGTGTTGGAAGATGCAACTAAATATATAAAAGAACTTCAAGATCGGGTCAAGGAGCTCGAGGGATCTCCAAGTACTAAGAGAAAACATGTACAAGAGTCTGTTATTTATGTAAAGAGATCTAGGCGTAGTGCTAGTGATGATCAATATTATTCGTCGGATGACACAAACTCTGAAGAGAGCACTGCACCTTACAAGACATCTCCTGAAATCGAAGTGCGGATGTCAGGAAGCAGTGTTCTAGTGTCTATTAAGTGCCATGATAACATTTCTTCATTGACAAAAGCACTAGATCATATGCAGAAACTTGGGTTGTCCATCATCAGTTGCAGTTCCATGCCCTTTGCTAAGACCACCCTTCTTATCGGTATAACTGCTCAGGTATATAAATTCGTTCTAAAATCTTGATCATGACACCTTCTGAGGGAAATTATAACGTAACTTCTAAGTTAATGCTTCACATCAAACTATATTTAACCGGGAAGTATATATAATTTGGTTTTGCTTAGAAATATGATCTTTCTTATCTCATATAAAAGGAAACCACATACAAGTTTTTTATCTTGTATAAATAATGATTTAGTGATAATTGTAACAACCTCTAAATGTCGGTTCTTCTTTCTTTTGTTTGTGCAGATTGAGGATGACTTCTGTATGACAACAACAGAACTTGTAAAAAACCTTCAACTAGCTTTATGATGACATCAAATAACAAGTTTAAGGAAACCATGTAATATATCAAGGACTCGAATATCGTTCATTAATTAGTAGGTATAATTTGGGCGTCAATTTTAAACTTCAGAAGGACGTGAATTGAGAACATTCTCATTCCCATATTGGGTTTTTTTTCAAGTACTCCTGAAGAATCATATCTACTCGTAGCATGATTGATGGAAGGATTCATGAGCTCAATCCCGCAATGGGTTTTAATAGCTTTTTGCGTGGTGTTTACCACGTTGTTTAATATGTTTTACCCATGTGATATGCTGATGAAACTACCATTTTTTCTCCTTGAATTAGCTGTATTTGATACTTTATGTTTCTTTTTCGAGGACAATGAAGATTTGCAAAAGATTTCGTACAGTTTTCCTTTAACTTTACTTAACAAAGTCCAGATAAGCAGCTTTAAATTTAAATAGCTCATTAATTATTGTTGATATATACGGAGTATGTATCAAATGGCTTCGTAATTGCTCTAATGAAAGGTTAATATAATTCGATATATTACGTAGGGGCTCTAAAAAATTAACACCCACAATATAACATAAAATTATGTCGGGT

AabHLH25

GACAATCAAATGCATTTTAGCCTTTTATATAGTTTTTGTTGAGATAGTACTATACATGAACCCGATTAGTGACTATCCTACACAACACCCTTTCAAAATAGTTCAATGAATGTCACTTACATCTACTATACATTTGTACACATACATATTTGTCTTTAAGTTATGAATAAAAGTTGAAAAATTGAAAATATAAAGAAAAAAAGTTTATGTCGTAAGAAGAAAGGAAAGGTACCAAGCGGTCACTCCCGGCCCTCATGAGAGGTGAGAGAAAAACAAAAAGGAAACCCTAAAAAGATCACCACAAACTTATAGCACAAACAAGAAAGTGTGTTTGCTTTACTTGATTATTCCAAGTATCAAAAGGGTCATGCCATGCCATCCCATCCCACTAACAATCACACAAAACCTGACATTTCCAAGAGAGAAAGTGATCATTAAAAAATACAAGGTTTTTATACAAAGAAAAGTGATGTGATTAATGCCTAGCTTTCACTATTATCAGAAGGTTTATTTAACACTTTTATGACAATATTCTTTTTTCATACTCGTATTTATATCCCACCAAAGTTGTTCTTCTAAGTATTTATTTATGTGTTCTAAGAGAGAAGAACAAGAACTAGTAGAAGAACAACAACTACAACAAGAAGGTGATCATCAAGAACATCCATCACTTATTATTCCACAAACTCAACATCAAAATATGGGTATTGTTGATTATTCATACACATCATCATCAGAAGTCTCACCTATCTTGCACCCACAACAACAACCATGGATCATACCTCAAGTATTTAACCATCACAATACCGCTTGCACTGATCTTCCATTTACTGACCACGGAAACTATATATTCCCTCCACCACCTCCTCCATCATCACTAACTTCGTCCTATGGTGGTTTGGTTAATAGGAGAGTCCCTAGTGGCTTGCAGTTTGCATATGATGGTAGTACATCCTCATCAGACCATCATTTGAGGCTCATATCCGAGACACTCGGCCAAATGGTTCAGCCAGGGTCTATGCCGTTTGGGCTACAAGCAGAAATGGGCAAAATGACTGCTCAAGAGATCATGGATGCTAAAGCTTTAGCTGCTTCAAAGAGCCATAGCGAAGCAGAAAGACGACGTAGAGAGCGAATCAACAATCATCTAGCTAAACTCCGTAGCATACTTCCTAGCACCACCAAAGTAAGTCTTCTTAGTATAAACACAAAAATAAAAATCTTAAGTGATGTTCCACCTATCACTATACATAATGTTAGTTTGCATGAAACTAAAGGTTCAGATCTTTATTTACATTGAACTTGATTAGTTTGTTATAACATAAAATGACACTTAACAAGAAATAATGATGAACTTTAGAATTATATTTCTTGTGTAATCAATTTAGTTTGATGGTATTTTGATCCGTAGATCTACAGTTAATGTTCAGCTATCATAAGACACTTGATCATTTTTGGAAAGAAAAACACTATAAATGTACTTGCGATAATCTCATACCGGATTTTTGCAGTACAACTATTTTCTGAAGTATATATACCCGTATAATTTATAAGAAATTTACGATAATATTCCTAAGAAATCCCAGGAAAACAAGATTATTGAAGGTTCCTATGACACTAAGTTGTGTTCATAGTTCAAACCATGTTTGACGAATCAAAAACCCCACCAGAAGTACACTAAATAATTGTAACCTTTTGGTAGAGCTAATTTTTAGATCTTCAAGTAAGTTTATCTTTATGTTTTCCTTAACTATTACTCTTATGGTGTGCATCAAATGATTATGCATTTGTTTGAGTTGGTAGATATATGTGAGGAATTTAAGGCATTATATAACTTTGATCAGTTAACGATTCAAAGGATCATGCTTGACTATTCATGTCCAATTATTGGAATTATCTTCCATCACATCATGCATACATCATACAAACATAAAAAGTTCAATCTTTGATCAGTATGGTTTTTCTTATGTTTCAGACAAAAACAAAGATGTAGTCCAAAACATACTCGCTTTATGATCCAGATTTAATATTAGAAAATGTATATAGACCATTATAATATATAATGTTTGTGCACTATCCTTATTTGGTTTAATCAAATTGTCTGATTCTTATTTTTATAATTTGACATAGACGGACAAAGCTTCGTTGCTAGCTGAAGTAATACAACACTTGAAAGAGCTCAAGCGTCAAACTTCAATAATAGCTGAACAAAGTCCGGTCCCTACAGAGACTGACGAGTTGATCATTGACAACACGTCGGATGAAGATGGAAATTTAGTCATCCGAGCATCGTTGTGTTGTGAGGACCGCTCAGATCTTTTACCAGACCTCATCAAAACCCTAAAAGCACTTCACCTAAGAACCCTAAAAGCCGAGATCACAACACTTGGAGGACGTGTGAAGAATGTATTGTTCATTACAGGAGATCAAGATCCGAACAATAACGATAGTAATACAAATTACTCGATAAACATGATTCAAGAAGCATTTAAAGCAGTAATGGAGAAAACAAATGGGGATCATGAATCAACTTCTGGGAGTTCTAAGAGACAAAGAACAAACAGCATTCATGTTGTTGATCATCGTCGGACATGATCATATATATGTTGTCTTTATAAACATATGAAGCTTTATGGCTTACACCTTTAAGAATTTCGGTTGTTTTTGTCTTCCTTTGAGATCTTGGGTTTAAGTCCTTCAATTCCTTTGTACTTTGTGTGTGTTTTCTTTTATGTTTTGGGGGAGCACTTTTGTTTGATTAATGTGTATGCTACTATGCTTTGATGTGTTGTTAAACTTGTATCATTCCAGAAAAGATAGAAAGTACTATCTGGATTATGTCCTTATCAGTTTTCTTATCTCATGAAATAGAGGGGGAGAGAGAAACTTGTATGAAAACCAAACATGGGAATGTCCAGTTTGATCCAATTTTATAACGGTTTTGCTACTAGTTACTCCTAAATCCTAATAACATCTGCCGCACTAGTACTCCTAAGTCCTAATAACATCTGCCGCGCGCACTAGCAAACTATAACAGATCGAGAGTTAAATATTGTTGCAATATATTAGAAACGCACTAGCATGAAGCTAGAGCAAAGAAATTACAATAAAATATTACATGCTTTGATAGGGTTGTGAAGTTGCAACAAATATATGTAACGAGGATTTTAACCTGCACAT

AabHLH26

TAATTCATCATAATTCATATAACTCGATGCCATTAGGGTAGGTGATTGAATTAGCAATGTACAAAGAAAAGTACTTGACACAAAAGAAAGTGACAAATCAACATGCATGCACATATAGTATAAGCCTAAATATGGTTATTTAAAAGAAAAGAAGAAAAACAGACATAAAAATATTCGCTTATCCCACATGACAATTCAGTGCATTACATTATTTGGTATGTTCTATCACAACTCTAATTTGGAAATCCAAATTTGGACCCACAATCCCCTCAACTCCTCATCAAATCGACACCAAAAGACATACATCTTAAATCCCAACCATAGATAGGCTAGTTTATTTTTTATTCACCTTTTAAAAAAATTGAGGGTGTAGTAGCATTATTTAAAGACCCCAAAATAAGTAGGGAATACCAAATTTGGAATTTGACAACACCAAAAGGAAGCAAAGAACAAGAAGAAGAAAGATAAAGATCCTCTCACATGCATCAACAGCAGCCTACCCCTCCTCTTGTCCCCATGTACATATAATCATATAGTCGTTATAGAATCTTCTTCTTCTTCTTCTTTTTATTTACGGTATTTTTTTTTGGTAAAAACCTTTTTTTCTAGACATGCATCAGTGACATGTGCCTTGTGCTTTAAGAATTTTACATTGCTTATCTGGAAATAGGACTAGTATTTTTTTTTCCATATTGTATAAATAGCATGTCTTGCAAGTGACTGTGTGTATTTACACGGCCTTCTATCTGGCTTCACTAGCACAGAAATAAATGGCTTTTTCAAGGTTACCAATAATATACTACTAACAAAAAAAATCTTACAAATTAAAGAAATAATCTTTTTTCATTTTTAGCATATAGGTATATTAGTGATTTACTTACTCTGTATGTATATGAATTTTCAGGCTTGCTGACTATGGAGTAGCAGAAATAACATGGGAAAATGGCCAACCAGCCATGCATGGGCTAGGAAGAGCAAATGAGACACTAGAATCAATTGTTCATCAAGCTACAACATGTTACAACCAAACTCAATATCCAGAAATCGACTTACAACAAAGTCAAAGTCTTCCAAGAGCTCGCAACTTAAGCTCGAATGTTGCATCATCGAGTCGCCCGACTTACCTAAGGAAACGGCCTAGAGAATCTGTCATTATCCATGATCAATGTGTAGGAAATTTGGGTAACGCAAGTTTGCAAGAAGATAATGTTAGCAATAGTGGGACGGTTAATTCTAAAGATAATGATACTACAATGATGACATGGCCTTCATTCGACTCGCCTAATCAAAGCATGAAGAGCCAAAAAACAGATGATGATTCTGCCTGCCAATATGGATCGGTATAAATATTCAAATTCTTAAGAAAGTTTGAGTTTTTAATAAAACTTGAATTCATCAAAGTGAAAAAAATAAAATAAAAAATTAGGATGTCTTTATGCACAAATATTTTTAGTTGAATTTTCTGTTTCTTGATACTGAACAGGAAAATCAAGAAGAAGAATGTAGGACTGAGGGTGAAACAATTCGATCTCAATCAAGTCGACGAAGCAGAGCAGCTGCTATTCATAACCAGTCCGAACGGGTAATTTACTAATTTGTTTGAAAATTTTATTATTTAACAAAATATAGAAAAATTAAAGACTTCATAATGAATAAATAAATCTTACATGATATATTATTACTTTAGCGACGAAGAGAAAGGATCAACCAGAAAATGAAAGCTCTACAGAAGCTTGTGCCTAATGCTAATAAGGTTAGAATCTATGTGTTCTCTTGTACTTTTGACTTCAAAAGTCAATCCTACCAAACTACATCTCCAAAGTTATTATTGTATTAATTTCTTAAAATAACAGAATTATTTTTCCATATATAGAATTATCAAAATCTTAGTTTTAGGCCAGTCATGTCAATTTTTATAGTTAAAGTTAATCCAGTTTGACCTGAAAAGTCAACATGTACTTTGTACCTCCTTTTTAACTCATTTCAGTTTAGTGGAAAGAGTATCTAAATTTAGAAATTGTTTATACAGACGGATAAAGCATCGCTGTTGGATGAAGTGATTGATTACTTAAAAAAGCTACAATCACAAGTACAATTGATGAAGAACATGCCGTTTACACCACAACAAATGATGATGTCAATGCCCCTACAATTGCAGCAACAACAGCATCAACATCAGCAGCAGCAGCAGCTTCAAATGTCGATGCTAGCGCGAATGGGAATGGGATTCGGCCTTCAGATGGGAATGCCTGGAGTTATACCTCAGCCAGTTCATAATCCATTCATGGTCCCACAAACCATGCTTAGCCCAGCCCATGTAGGCACCACGTCACAAACTATTCATAGTCGCCCATCTACCAACACCCCAGTTCCTTTCAACGATCCACACAGCACGTTTCTAGCACAAGTATGTCAAGAACCGAAAATGAGCAGTTCAAGTTTAACTTTTAGTAGTTCTTAACTTCTAGACTGTTATTTTAATCCTTGTTTTTGAATGCAGCAAATGAACATGGATATGTACAACAACATGGCAGCTTTCTATCGGCAACAGGTCAACCAGGGAAAATCGATGAGCGTTGACTCATCTCAACTAGACCATGTTCGGGGAGAGTGATGATAAACAAAAAGCATCTTGTAGTTATTACAGAGTATTGAAGAGTCTATAATAGTAATTTACAACTAAGTATGTCTTAATATGTTGATCATGTAAGTGTTTTTCGTGTATTTCTTCCTAATATCAGTTTTGTTGTTTTGTTATTATTAATCAAAGCACAAATGAACAAAAGACATGTTAATAATATTGTTCCCACTTACTAAGTTAGTAACATGTCTAAGATATAGTTTTACCATGTTAACAGTTTTACAGTTTATATGAATGATCAATTAAAACACACCAATGACTAAGTTGGGTAATTATTTCAAAAAATACATTAGTCATTTGGGTTGAATCTTAGGAAGGAAAAGAGAGGTTTGTTGTGTTTTGGTCTTTGCCACGAAATGATGATTAGCGACAGTTTTCTGTCACAAAACGTCGCTAATGAACCATCACTGACGTAAAGACACACCTATCGAAATGGTGGCGGTTATATCTGTTAAAAATGCATCATTGAGCGAACCATC

AabHLH27

ATTTTCTACCTAAAAACTAAATTCTTTTTCTTTCGCTACAACGCGCAGGTTGTATCATCTATAATAACAATCACTGATTTGGTATACTATTTACCTTTATAAAGTATTTTTCTAACTTGTGTCCTAAGGGCACAAGTTAAGCACACCAAAAAGTATAGAAAAATACTCAGAAACTCACACAAAAATATAAAAAAGTATATGTTTGTGTAGATTCTTAAGTCCTTTTTCATGGGTCTTATAGTATTTTTAGTGTGTTGAACTTGTGCTAATAGGGCACAAGATAGTGAGCATAAATAGTGATTTTCACGTAACTGAAATTATAATTTTCATTCTTTATCTAATCTTGTTGAGAGTGAGATAGATCGTGGGTCCCAATAGAAACACCAAGTAACAAACTTCCCTTCCTTGGTTGCCCTTTTCTACACTTCCATTCCCTACTCTTAACCTACTTCTCCCCCTTCAAATTCCTTCTCCCATTTCTCATTCTCTAACAATTTTTCTCAACTCATTTTCCAAAAACACAAGAACACAAAATGCTAGTTGATCCTGAAATGTATGAAGGAAGTGCTTGCTATGATCCAACTCATCTCGAATCGCTTATCGACCATCAAGATGACAACAATATGTCCCAAGCACATCTCCATAACTACCATCAAGCTCAAAATTTTAACTCACTTGATCAACAACAAGATTATCATATGATCAACATGGAAATGGAACACCAAAACCAAATCATGCAAGACCACCTTAATTGGTCCACTACTCATTCCCATGAACAAATACATATAGAAAACAATAATACTAGTAGCATACCAAACATGCCTTTAATCACACCTAATCCAACCCCACCTGATCTTTTGAACTTGTTTCAATTACCAAGATGCTCGAACTCATCTATATCGTTTTCTAACCCTACCCATATGGACCAAACTTCGTATGATCCTTTGTTGCCTTTAAATTTACCTCCACAACCGCCTTTTTTTCGCGAGTTGCTTCATTCGCTCCCTAACGGGTATAATTTAACGGGTGGTGGGTCGATTTTCGGAGAAATGGACATGGAAAGAGATCACCAATTGTATCATCATGAAGGTAATGGAATTTTGAAGTTTAGTGGTGCTGATATTAGTGGGATTGTTGGAAAAGGTAGAGATGTTAAGGATACTAAACATTTTGCTACTGAAAAACATAGGAGGCAACAATTGAATGATAAATTTGATGCTTTGAAGAATTTGGTTCCTAATCCTACCAAGGTATAATAGTTTTGTCTATCATAGATATTCTAGTATATGCAGAAAAAAGAAAAACATGTTTGGAAAATTATAAAAGTACCCAAAATGGCAAAAATCAGAATGTTACTTTTTGTTTTTATTTATAGTTTGTGATCTATGTGTCTTTTTAGTTATACCCTAAATGGCTTTATTATGATTCTCTTAGTTATCATGACACATGAAACAATAATGTCATACTACTTTGATGATTTTTTTTTTTTTTTTTTTTTTAAAAAAATTGCGTCCACTACTTCTTCCCTATGAACAAATAATATAGAAAACAATAATACTAGTAGCATACCAAACATGCCTTTAATCACACCTAATCCAACCCCACCTGATCTTTTGAACTTGTTTCAATTACCAAGATGCTCGAACTCATCTATATCGTTTTCTAACCCTACCCATATGGACCAAACTTCGTATGATCCTTTGTTGCCTTTAAATTTACCTCCACAACCACCTTTTTTTCGCGAGTTGCTTCATTCGCTCCCTAACGGGTATAATTTAACGGGTGGTGGGTCAATTTTCGGAGAAATGGACATGGAAAGAGATCACCAATTGTATCATCATGAAGGTGATGGAATTTTGAAGTTTAGTGGTGCAGATATTAGTGGGATTGTTGGAAAAGGTAGAGATGTTAAGGATACTAAACATTTTGCTACTGAAAAACATAGGAGGCAACAATTGAATGATAAATTTGATGCTTTGAAGAATTTGGTTCCTAATCCTACCAAGGTATAATAATTTTGTCTGTCATAGATTTCTAGTATATGCAGAAAAAAGAAAAACATGTTTGGAAAATTATAAAAGTACCCAAAATGGCAAAAATCAGAATGTTACTTTTTGTTTTTATTTATAGTTTGTGATCTATGTGTCTTTTTAGTTATACCCTAAATGGCTTTATTATGATTCTCTTAGTTATCATGACACATGAAACAATAATGTCATACTACTTTGATGATTTTTTTTTTTTTTTTTTTTTAAAGAAACATGTAAAACTAAAAGTCACTTGTTTTCTTGTTAATTGTCTAATTGAAAATTTGATTTGATCATGAACCACTAAAGGCGGATCGAGCATCAGTGGTAGGAGATGCAATTGACTACATAAATGAGCTCAAAAGAACCGTCGAGGAACTCACAATTCTTGTGGAGAGGAAAAGATGCAGCAGAGGAAGGATGAAAAAGCACAAAACCGAAGATGACTCGACACTAGATGTTGAAAGCATCAACACTAGGCCGAATGGTGGTGGTGATCAAGACCAACAAGGTTATAATGGTAATTCCACTTCTACGTTGAGGAGTTCATGGCTTCAACGAAAATCAAAGAACACTGAGGTCGATGTTCGTATCATTGATGATGAAGTTACCATCAAATTTGTCCAACAAAAGAGAATCAATTGTTTGTTGTTTGTTTCCAAAGTTCTTGATGAACTTCAATTGGATCTTAACCATGTTGCTGGTGGACTAATTGGTGACTTCTATAGTTACCTTTTCAACACCAAGGTACAAGTACTCACTATCTTATTTCTTGTAATTGTTCGTCTTGGTTGTTCTTGCTGGTTTTCTGATATAATCGCTGTTTAAAAAATAAATAAATACTTATTTATTTATTTTTTTTTTAAAATTGCATGCAGATTTGTGAAGGGTCATCAGTTTATGCAAGTGCTATAGCAAATAAGCTAATTGAGGTTGTCGACAGGCACTATGCAAGTATCCCAACACCTAGTGGCTACTAGGTAGGAAAATCTACAAGGTACAACTGAATATGCAGAACGTACAACATCTTATGCATAGTGTACAACCAACTTATAACTGCCACATGCATGTTCTTGCTCTGATTTTGTGAATGGTACACCATAGTATGTAATATTTGATCCAGTATCTCAGTAACTACTGGCTAATACGGTAAAAAAAATACACAAGGTACAATTGACTATGCATAAGGTACAACCCTCTATGCGTTATGTACGTTTTCTTTGGATGTACACTATTAGGTACAAGCCTTACAACATTTATAAATCTTAAAGGTATTGTTGTTTTTTATATAAAAGAATCTGATCTTAGCATATGTTTTCTTAATCTTACCTAACTCAAAGGATAAATAACGAGTAGAATATAATAGGGTTATATTTTTTTGAGCAACATATTTTTGGCAAATGTATAGTAGGCTAATATGGGCTGAAAAAAAATATGTTGCAATCCTAGTCAAAAGTTCTTTATAGATCCAACTTTTACCATAGAACCCATCA

AabHLH28

CTTTGAGTAATATATTTTACCAGGGAAACACTTTACACCCAAAAGATAATACTGTTCGCTTTGCAAAAAATATATGAGGATGTTGTTCACAATACCATTGAAAAAATGTGTGTATTAAAAGAACTTTCAAGTAATCTATGTGAAACACCGCTCAAAAGAAAACCCTATGTGAAACACAAATAATGTAACAAGTAGCCTATGTAAAACTCGGAAAAACACCATTTTTCATACTATCACGCACTTCAATAAACACCACTGTTTCATCAATTTCTCCATGATTACTCATTGAATTTCAAATCTGATAACACCATTGAATTCCTCTTATCATTCCGCACATAATGGTGAGATCGAATTTGATTTTTAGTTATTTTTGAAGAAACCCCTAAATTCAATAATTTGGAGGTATAATTCAGTTTCTTATTTTTGAATTAGTTTGCCTTGTATTTCATTCTACTACACTGTGTTTAGCTTTTAGAGTACTCAATTTACTATTAGTTGTTATGACGAATAAAAAAAGCCAAAATCAAAGAACATCAAAGCGGAACCCTAGGGCTCCGATTTGGTTAGATGATCATGTTGTTAGTAGTTTGGGGCAGAAGAGAAATGAGAAGGAGGTTGATGTTGGTAAAAAGGTGAATGTGAATAGTACAGGGAAAACTAATGTGGCTGAGAAAGTATGTGAATCAGAGAAGGAGGTTAGTGTAAGCATGGAAGAACAGGCTAGGAAGAAGAAGAATGATAGTAATAAGATGCTGAATACATATGTTTTAGAGAAGGATAACAGTCTTAATGTGGGGGGTAAGGATAGAATGGAGGGTCATGTTGTGAGTAATGATGTTAATAAAACGGATAGTGTGAATGGTACTAATGGTAAGGTGGATAATGATCTAGCTACTGGTTCAGATGTGAATAAGAGTAAGGTTGCTGTTGTTGACAAGAGCAAGGAGCAAGGTAATATGGAGAAGTGTGAATCAGTTGATGAACATGATGAGATTAAGAAAGATGATAGGAAGACTTATGCTAGTGCTACTTATGATACTAAACTAGATATGTCTAGGAAGTTGTTTGAAGTTCCTACTGAGGTGGATGAGAATGGATATGAGTATGTAGTGTTTGATGAAATAATGATTGATGAAGGAAGCAAAAAATGGGATTTGACACTATGTGGTTATTTTGTGGGGCATAATATGACAATCAATGAGTTAAGGTATTAAAACAATACATAATTAGTTCTTTAGCAAGTTCCCCAAAACTCACATAAACAACAAAAGTACAACTCGATCTAAATACGATTGTGATCAAACACTTTGACAACAGCAACAATACGCAATTCCGCAAAGGCGGGTACGGGTTGATCAAAACATTAGACCTATAACCTTTTTTCAGAAGATGAAGATTGTGTAAAGTTGTGTAAAATAACCTTTGAGCCTACCTAATTAGAACCAAACCTTCACTTACAACTTGAACTTAGGAATGCACTCTTTTAGTCCATGCACTTACAACTCTATTCTATTCAATCACTGCATTTACTAGAGTTTCAAGTTAAAAATTAGAATGTAAATATAGATTTATAACACAAGTCGCAAAATATAATGAAGAATTGGTATAGAAAAAATTGCGGTTTTCATTAATCCGTTTGGTAAAAATCCATGCATTACCTAATTTAAACTTCTCATCAAAACTAGATGTCAATATGAGTTCTAAATAAGTAATTTGAAAAAAACATAAAGACTTTCATATAGACGTCAAACTGAAGAGTTGAAGATATGTGCAGGTTGGGAGTTGGTAGAGTGGGTTATGCTAGGGTGATGGTTGAAGTATCAGCAAAGAAATGTTTGCCTGATATTATAGAAATGATTTACAGGAATAAGAATGGAGGTGAGATATGTAGGAAAACAGTGAATGTGGTATATGATTGGACTCCTCCTAGATGCTCACATTGTTGTGTATTTGGTCATAGTGATAAGATGTGCAAGGTTTGTGAGAGTAATGAGGAGCCAAAAGATGCTAATAATACAGTAGAAGTGTCTGCTGAAAAAGAGAACAAGTCAGAGGAAGGAAAAGAGAATGTTGAGAAGAAGAATGATGGTTTTGAAGAGGTTAGATATAAAAAGAACAATGGTGGTAATAAAGGAAAAGGGCATAACAATAATAATGGTCAGAGGAAAAATGATGGTAAGCAGAATGTGCAACAAAATCAGGGTGTATATCAGAAGAAGGTGAATAAGGAGCAAGGTGAATCTAGTAATAGTAATAAAACAACAAAGTCTCCTGTGAAATCTCCAGTGAAAGCACCACTTAATAATAACAATGTCCCGGGTACACCTAATAGTAGGAAGGCTTGGAAAGTTCAGGGTGAGATTCTGGAAGAATTAAAGAGGTCTGCTAATAAGTTTGCTGGATTGGAAGTACCTGATGATACTGTGGTTGATGGGAATGGGCAGAGTAATGGTAAAAGAATGAATGGTAATGGTATTGAGGAAAATGATGTCTATCCTGATAAGAATGGTATTGCACAGGGAATGGAAGAGGAAATTATTGAAGGAATTGATAAAGGGGTGTTTGGGAAACAATGAGTGTTCTATGGATCTGAAAATTGGATGTTGGAACATCAGGGGTTTAAGTACCACTGATAAACAAAATGAGGTTAGAAAGTATATTGATGATGAAAGGTTGCATATGTGTGGTATTATAGAAACTCAGTTAAAGACAAAAAAGTTACAAAAAATTGGAGATTCTGTCTTCAAAAATTGGTCTTGGGTTAATAATATGAGGATGTGTGATAAGGGATGCAGAATCATGTTAGGATGGAATAGTGATATTGTCAATGTAAATGTCATTCATTACAGCAAGCAGTCTATTTTGTGCAAGGTTGAAATTGTAACTGGGAATATGGCTCTGTTTTGTACAATCATTTATGCTGCTAATAGTGGTAATGAGAGGAAGGATCTATGGTATGAGTTGGGTCAGTATAAAAGGGTGGTGGGCAATGATCCATGGGTTCTTATGGGTGACATGAATGTCACTATGGATCCAAGTGAACATTCTGCTGGTGGTTCAAATATGAGTAGAGATATGATTGAATTCAAGGAATGCGTTAACCACATTGAGGTAGAGGATATCACTTGTTCTGGGTTGCACTATACTTGGACTAAGAATTTGTTTAAAACTAAAGCTGGTGATAAGACTGGTATTCTCAAAAAGCTTGACAGAGTTATGGGGAGTGAAGAATTCATAAGTAAGTTCACTAATGCTCATGCTATTTTTCTACCTTATATAATTTCTGACCACTGCCCTGTAGTATTAGTTATACCTAATATTGTTCAACAAAAGAAGAAGGCTTTCAGATTTGCTAATTTTGTGACTGATAAAGAGGAATTTTGTGAAGTGGTTAATAAGTATTGGGGATCTACTAGTGCTGGATGTTATATGTTTAGGGTTGTAAAGAATCTTAGACTACTGAAGAAACATATGAAAAAGTTGGCTTGGCAAAATGGGGATGTATTTGAGAATGTGAAAAGTATCAGAGAAGAATTGAAGAATGTACAGGGCAATATTGATAATGATCCTGATAATAAAAAGCTTAGAGATGATGAAAGCAGGATTCTAAGTCAATATAGTATTGCTTTAAAAGAAGAGGAAAAACTGTTGTATCAAAAAGCAAAAGTGAAATGGCTAAGTTTAGGTGATAGAAATAATGCTTTTTTCCACAAGGTGTTGAAAGGTAGGAATCATAGGAATAGAATCAATGCTATTCATGATGAGCAGGGCAATAGATATGAAGGAGTTCAAGTTGCTCAGCAATTCATTAAACATTTTGAGAACTTCTTGGGTAATAGTGTTCCTGTGGCTGATTTTCATGGTGCTGATGTTCAATTCATAACTAGGCTGAGTAGTGATGAAGCTGGTTTTATGGTTAGAGATATATCTGACAATGAGATCAAGCAAGCTATGTTTCAGATTGATAGTAATAAAGCTCCTGGTCCTGATGGGTACTCCTCTCAATTTTTCAAGAAGGCTTGGCATATTGTTGGTGGGGATGTGTGCAAGGCTGTAAAAGAATTCTTTGATAATGGGAAGTTCTTATCTGAGATTAATTCTACCCTAATAGCACTTGTTCCTAAGATACAAACTCCTTGCAAAGTGTCTGATTACAGGCCTATTGCATGCTGCAATGTTATATATAAATGTGTAAGTAAGATCATCACTGATAGAATGAAAAAGAGTCTTGGGAAACTTGTAAGCAGTAATCAAAGTGCATTCATTCCTAATAGGCAGATCCAGGACAACATTTTGGTATCTCAAGAGTTGTTAAAAGGGTATGGAAGGAAGGAAGGGCCTAGTAGAGTTGCTTTGAAGATTGATCTACAAAAAGCTTATGACACAGTAAATTGGAAATTCTTAGAAGAGATTTTGCATGGGTTTGGATTTCATAATAAGATGGTGCAATGGATCATGAAATGTGTGACTTCTACATCTTTCTCTTTAAGTGTTAATGGGGAGAGTTGTGGTTATTTCAAAGGGGGGAGAGGACTCAGGCAGGGTGACCCCATGTCTCCTTATCTCTTCACACTTGTCATGGAAATTTTGAATCTCATTATGATTAAAAAGGCTGAAACTGCTAGTAAATTTCAATATCATTTTGGGTGTAAGAAACTGAAGTTGACCCATGTCTGTTTTGCTGATGATTTGATCATGTTTTGCCATGGAGATAAAGATTCAGTGAATGTTATTAAGGAGGCTATTGAAGAATTTGGGTCCATTTCAGGGTTGAAACCTAATTACAACAAAAGTACTATCATTTTTGGCAGTGTTAGTGAGAATACTAAGAATGATATTCTTGGAATTGTGCCTTTTAAAGTTGAGAGTTTGCCAGTGAGATATCTTGGTGTTCCATTGATTACTAAGAGGATTGGTGTTAAAGAATGCAAATGTTTGATTGACAAAGTGAGAAACAGAGTTCTAAATTGGAAGAATAAGTGTTTGTCATATGCTGGAAGGCTTCAGTTAGTGGCCTCTATATTGGAATCTATCCATGTTTATTGGGCTAATGTTTTCTTACTTCCTCAAACTGTGATTAAAGATATCAATAGATTGTTGAAAGGTTTCTTATGGAATCAAGGTGAGAGAGCTGATGGCAAAGCTAAAGTAGCATGGAGTACTTTGTGTAAACCTAAGTCTAAGGGAGGTCTGGGGTTGAAAAATTTGAAAGTTTGGAATAAAGCATTGATCACTAAACACCTTTGGTATATAGCAGATGACAAAGACTCTCTTTGGGTGAAATGGATCAACACTTATAAACTAAAAGGTAGAAATATTTGGGAAGTTAATGAAGATGCTAATGATAGTTGGGGCTGGAGAAATTTACTAAGACTGAGGCATGATGTTAGAGTTCATATGGTGAAGAGAATTGGTGATGGGGCTGACACTTCTGTTTGGTTTGATGATTGGACTACATTTGGGGCTATTCATAGATACATCACATACAGAGACTTGTATAAAGAAAGGTACAAGGCTGATATGAGTGTTAAAGAATTTGTTGAAGTTAGTAATGGAAAATGGCCAGCTGCATGGGTTACAAAATTACCAGTTAATGATGAGAACCATAATATTTTCCTCAACAGTGAGCAACATGATAAGCTAATGTGGAGGAGTAATGATGGTTTATTGGGGAAATTTTCTGTCAAGCAAGCCTATAAAGATTTATCTAGTACTGAAAATGAGGTCCCATGGTGCAAATTGGTGTGGTATTCACAAAATATACCAAAATTTGCATTTATACTATGGTTAGCCATTCAAGGAAGGTTAACAACTCAAGATCAGATAAGGAAGTGGGGATCCTATGATTTAATGATTTGTCCATTATGCTATGAGGAAACTGATTCACATGAACATCTATTTTTCAAATGCAAGTTTTCAAATGGGCTGTGGAGTAAGGTCTTGGAGAAAATTCAGGGGCAACAATGGGTGAATTTAGAATGGCAGAAGTTAATAGAGCAATTAGCCAATGTGTATAATGGTAATACTATCAACAGTGTGGTTAGAAGATTGTGCCTAGCAACTAGTGTGTATATGATCTGGCAAGAGAGGAATTTTAGAATCTTCAGAGATGAAAGTAGAAATGTGGAGGTGATTTTTCAGATTGTTTGTGAAAATGTTAAAAATAGGTTAAGTGGGCTCAATGTTAAAAGTTCTGTTGCTGTCAAAAAAGTGGAAGAGATTTGGAATGTGAAGTTTGCTGGTTCTGTTTGATAGTATGAATGCACAAGGGTCTGTTATGGATGCTGACTTTTGCAGTCCTGAAAGTATTTTGTCATCCAGTGGAATATGTTTTGGATGTGCAGAGGTATTTGGCTTATGTATCTCTGAAAAAATAAGGTTTAACCTTTATTCATCAGTTCATTTGCTAATTGGATTGATTGAATCAAGTTCACTTAAGATGATGGCTCAGTACCTTGGGATTCAGATTGAACATAGTTGTTTCTGAGTCAAATAGTCTCTTTATAGGCCTTTGTTAGGTAATAAAGGGGTATTTGGGTCAAAAATAACTCATGGGATATTCTGATAACCATAATGTGAGTTTCCATCTGTGAAGGGTAAAGTGAACAGGTGTGGTGGTGTATGTTTCTAGGAATTGTGAGGTGTGAAAGATGAACCTACATGGTCCTAGATATGCACTGCTAAGGAGGAAAGGGTAGGTACTCACTGCAAGGTTGTGTTCCCATTTATGTGAAGCCATTGTTGTAGATGGAAATGTAGCATAGATGGTATAGCACTTGCAGTGGAGAGGTTATGGGTTCCTAGTGAAGCCACTAATCTAGTCCATCATGGTTGTTGTGGTGGCAGGTTGAATGATCATGATGGGTAGATAAGGTGGAAATGTAGCTAGATGGTTTATGGTGCACTTAAAGGTGATATTGGTTTCTGCAGTATGTGTTGAATGCTGCAGATTGTATGAATTCATGAAGGTTATATGTGACAAAAATGGGATGTTGTACTGCTTCTTGTGTTTCTGTTTTACTAGTATGTGAAGATATTGTATCTGGTACGATTGTTGTATTATAAGGTCTATAGGCATGTCTATAGTCCTTCTATGATGTATGAATTGTTGGTCATTAATAAAATATGTTCACCTTGTGCACCAAAAAAAAAACTATTTACTCTTCCATAATGTTTTTTGATTCTAATTTTGATTGATATCTCAAATCACAATCATCACCAAACACATCCTCGTGTTTCAGCAAACCTATAACTACCAAGGTGCAATAAAATCAAGCACCAATCAAACCAGCTATATTACGTTGTACCCTGCAGACTAAAAGTATCTTTCTAAAGGGATTGATCCTACAAACTGAATACTATACTAATCTATCTTTTCATTAGCTGATCTATACTCTGCACNAAAAAAAAAAAAAAAAAAAAAAAAAAACTCGGAAAAACAACTATGAATGAACAAAAACTCAATATATAGTTCACGTCCACTTAACGTGACATTTAACTTCACAAGTACTAGAATATATATATACTTCGATCATAGATAAGCTATGTGCTACGATCACACCACCATTACGAATTATACAAAACCATGTCTCGATGTGGCTGTTTTAACCACCATCTACACTATTTGACATTTTTTATTGGAATAGCATTGATCCAAAACATCCAAAGAGAGACACTTTATATATGGGATGATATACAAATCAGTCTGGTCTCACTCTTGATTATCATAGCAGTACGTTATTCCGACCGCATTGGTTTGATTGGGATTTTAATCCAATCAAAGCTTGTAATGTAACCGTAATTTTTTTTTTTTTTTTTTTTTTTGGCTCTAATCTAGCTTCTTGCTAGAATTCATAGAAGAGGAGAAAGAAAAAAAATGTCTTTAAAAAAAAAGTCATGCATGTTGATGCATGCAAGTATATGATGGTTGCTGGTTCTACTGCATATATGATTTATCATATTATATTCATAAAAGGATGCGTTAATATAGTTGAACAATGTCTTCAAATGCACACTCAAGTAATATTTCCAAGTCTCATATCTTGAATAACCGATAACTATAACTATTCTATACTCGCACTTGCTACTTTTATATTAAACGTACGTACTTCAATGTGAGATTCTTTCTTCCAATATATGAAGTATAAAAAGCATATACTATAGACCGCAATAGCTTTTATAATTATCTTAGTATGGAAAATGTGTTTGACCGATCGAGGCATCAACAACATATGCCACAGACCAAAGAAAAGCTACATGCTCATAGCTTCCCTTTCCATCATTATTCTCTCTCTCTCTCACACACACATAGTGAGTGAGTGAATGAATATGTGCGCTTTAATCTTCGTTATTGTCTTTGGTCGGTCGACAAACTGTGTATTTTGTGTTCACTGTGTTCTACTCGTGTTTCACCCCAAACCATCCTTCTCTCTCTTCTCATGCCGGCACCACATTTCACACAGTCTAATAATGCTCTTTCAACCAATCATAAAGCAACATATACATACCAATAAATAGATATTTACTATATGCATCATCATCATATTCATATATATCTATCTATATGTAGGTAGGAAGTTTACAAATAAATAATATAGTTAAATTGTGTAAATTTTCAAAACTAAAAAGCACAAGTGTGATAGCTACCTAGTTGGATAGTGTGAGAGAAAAGTGGTAGAAATTAATGCCGTAAGACGAAAGGAAAGGTAATGTCTCCTCTCTCTCTCTCTCTCTCTCTCTCTCTCTCTCTCTCTCTCTCTCTCTCTCTCTCTCTCTCTCTCTCTCTAGAGATATGTAGTGTGAGAGATAGAGGATTGTGAGGGACCAATATGAGAAAAAACCAAAAAAGATAAATAATTTTTTAACACAAGGAACAAAAGCTTAAGCCTTTTTAACTTTATTGTTTACTAGAATAAAAAGGGTCACCACCCTCCCACCTCTTCCTCTTCCCACATCCATCTCATATTCCAAAACCTACCATTTCTAGAGAGAGAAACTCATCAAGAACAAGTATTTATTGGAAGAGGATTTCAATATAAGGTGAGTATTCAAGAAACCATCAAAAATCTATTATTTTCTTCTTATTTTAAGATTTTTTGGTTTTAACTTCTTGTGAGAATTAGGGTTTTCTTAGGACACTTCTGTTCTCAAATCATTAATCATTAGGATATGAAGATTCTAACCATGTCTATACATGGAGTGGTGAGTTAAACTATTATGTGTGCTAAAAGAGAAGAGGAGGTAGTAATAGAAGAAGGTGATCATCATCATCAACAACAACAAAATATTCAAAGGTTTCAAGAACAACATCAATTAGTGCTTCAAGAAATCCAACAACAACAAAACATGGGTAGTATTAATGATTATTCTTATAGTAACACTAGGTTAATGTTTCCATCATCATCATCCTCAGAAAATATTCCAATCATGCATCAACATCATCAGCAAGCATCATCATGGAGTACCTTACCTCAAGTGTTCCACCAAAACAACCTCAACCCACTTCTTAACTACCCGAACCGAGACAACGATCAGTTTCTCATCCCACAACCACCACCTCCTCCACCTTTATCATGCTATGGTGGCTTGTTTAACCGAAGGTTACCTTTGCAATTTGCATATGAAGGTAATCCCTCAGCTGATCATCACTTAAGACTTTTGTCAGAGACACTTGGGCATGTGGTTCAACCTGGATCCGGACCGTTTGGGCTCCAAGCTGAAATGAGTAAGATGACTGCTCAAGAGATCATGGATGCTAAAGCTTTAGCAGCTTCGAAAAGCCATAGTGAAGCTGAAAGACGTCGTAGGGAACGAATCAACAATCATCTAGCCAAACTTCGAAGCTTACTCCCTAGCACAACCAAAGTAAGTTTAATCTATTTTTCTTTCTTTATCTCAAAACGTTTTTCTGTATCATACTATTGGACCCACAAAATCAGAAGCAAGCTAGAATATTTCACGTGGTCGGTACATTTATACACAAGTTTTTTTTTTTTTTTTTTTTTTTTTTTTTTTTTGAAAGGCAACATTAATTATACACAAGTTTTTAATGATGTTAACCATCTTAATTTACATAAACTATATATATAATATAGGTATTAGTTTTTTAAAATACAAGATATTACAATCTCTCACAAATGCACAACATTTGTTTATGATTATAATTGAACTCACAACCTTTTAGGTGAGATCATTGGTCAGTCTATGTATTAGTATTAACTATTAAGTAAACGTGAGACAAAAGATAGATAAAGGAAGATTTAAGTGTGTTTAAAGAAGCACTTGAATTGTACGAAAGATTTATGCTTTTTTGGTGCAAATCCTTAAAGATCTCTTGAAAATCTAGTGAGAAAAAATGTGACCAATTTGATAAGATAAAGAGGAACCAATGAACAGCCAGTAGCTATAAAAGGTTGCTATGAAAATTTTGTTGCCGGGGTTTTTGAAGTCCAAGAAATTATGAAAAGAAACCACACTTTTCTACTAATAACAACACCAGACAAACTAAGCATTCTTGTGTCAGTCATTACACAAACACACACTTTGTATGTGTGTTTATACCCACGCCCACGCTTTAAAGTTTAAACCCCATCATTACTAATACACTCATGATAACTTGCTAGCTACTAGTGTTTTGAAATCCTCAACTATCTTCTTCTTTTTAAGCATAAGAACCCACCATCTTTTGTTTTCTTTTTTCACATAATGATGATCCTTCCACATTTATTCACACTCAAATGAATTGAGCATTGATATATCAAACAATGCGATGCATGTTATGAGTTATATACATGATCATGTATTTGAATGCTAACAATTTAGTTAATTAAAAGAAATTATGTAATGATGCATCATGAACTAAAGAACTGGCCTCAAGATGATCATTTCATATTTTTCTTTTGAAGTTGAGTTGTTTTTCCTTGTGTCTTCCACACCACCTATCAAATAACGAGTACGCCGATTGATTTGTCCACTTAGACCTTCGAAGAGGGGTCATTTGATTCAAAAATTGTAGTTACAATATAAAATGCATTAATGTGATAATTTTCGTATACAAGATACATTATTGTGTCAACAATGGTTAAAATGGATTAACTTTTTGATCTGATTTGAAGTTTCAAGTGCTATTCGATTTCATTATACTACTTTCTTATCACTATCTAGTAGCTCTCATGATAACCTACATGGTTTTTATTTTTTCAAGGCTCATTTAGGTATGAAGGTGTTCTACTGTTCATCCGTTGAGTAAACAGTGTATAACATAACCTGTGAGTCTGGAGTATGATAACTGAATAAAGCAAAGTAAATTGTACATGTATTATTAGCTAACAGAAAGAGAACAAAATCAATGTCTAGTTATCTTCGAACTAAATGAGTAAACATTTACATTTTATATGAGGATACATTGCATTTCGGTTTTCAGTAGCTCTAGGGTTTTTTTGTTAGCTAGTTAGTTTGTCACAAATGTTATTTTAACTTTAGTATAAGTAATTTATTTGGTTGTATCTAACTAACGATTTGCTTATACTTCTAGACTGACAAGGCTTCATTGCTAGCCGAAGTAATACAACATGTTAAAGAGTTAAAGCGTCAAACTTCGATAATAGCTGAACAATGTCCAGTCCCCACAGAGACTGACGAGTTAACTGTGGACAACGCGTCTGACAAAGATGGTAAACTTCTGATCAAAGCTTCATTATGTTGTGAGGACCGTTCAGACCTCTTGCCGGACCTCATCAAGACATTAAAGGCACTTCGACTAAGAACCCTAAAAGCTGAGATCACAACCCTTGGTGGACGTGTAAAGAATGTTTTGTTTATCACGGCAGACGAAGATCATTTAAATGGAAATGATGATCAGCAAATGGTAAATTACTCGATAAACACAATCCAAGAAGCACTTAAACAAGTGATGGAGAAAACAAATGGTGATGATTCAGGGAGTGTAAAGAGACAAAGAACAAACAACATTAACATTTTGGAGCATCATAGGTCTCTTTAACCATATTCAAGTGATTCAAAGTCATTAGCTAGCTAATCTTAGGGCTTTTTTTCCTCAAGATCTTCGGCTCAAGTCTTGGTTTGTTTGTTTGGTGTGGGGTGTTTTTCTTCTTTCTATCATTTTCCTTATTATTTTTCTAGTCTTTTGACATGGGCATGCTTAAATTGTTGTTGAAATTGTGTGTAAGAAGAACAAAAAACTAGTACTACATGGGTGGGTTCTTTATGTATCTCCTGTCATGAAAAAAGAGATCTTTTTCGTGAGGAGAGAAACCTAACATACAAGTGTCGTTTTTTTGAAGGAAACACTATGTTTGTTCCTTGTTATGTAGTGGAACATGTATTTATGACTATTATCTTTAGCTATTCTCTTTTTCTCTCTCTAAACATATGCTAAGTTAGTGGCGCGTAACTATAATGATGAGAAGGTGACGATAATAATTACATCACCATTACATACATAAGTTCTACTGTTAGGCTAAATGCCAAAAATGCATAACGAAAGTATACAACCAATGATTTAGGTGATTCAGTCAATTTGTTGTTGTGGACCTACTACATCCACTTCCAAAAGGAGTTTGTTAATGTTAATCGATCACC

AabHLH29

TTAGTCTAATGTGGCATCAATAATGGGCAATTACTAAACACAATAACTCTTGCTAGAACGTTAATAACTCGTTATCTTTTCTTCACAATCTAGCTCGAGAGATTTAAACAGATATTGACTTTAAGACCGTTCAACAGGGACGTCCAGAAGTGACATTCACCTTTGTTTCATTAGCTTTAATGATAAAATAATAATTCAATATTGTAGCCACCATACAAAACAAAATTTTTTATTAAAAAAGAACCTGTTTAAATATCAGTAATTTACGTGATTCTCGCTTCAAACCCCAAAGCCACTTTTCATTTCTTTTCAATTCTATTAACACGTACTGCTAAAAATAACAATCTTTCAAAAGATGAAACCCATTAATAATCTTAATCATAAACCAAAACCCATTCCTTAATCACTTTATTTCCACTATAAATCACTTCCAAAAAAAAAAAAATTAAAAAAAAAAACTATAATGGATTTCCCTCCAAATTGTTTCAAAGGATTCACTTCATCAGAAGAACATGTGCTAAAGGAAATGATGATGATGAGTCGTGTTAGAACGACTTCGTCATCATCTTCCTTAGTATTAGACAATGAAAAAGGTGAAATAGTGAGAGCTTTAGTGACACCGGGCAATATTCGCCATACTTTTCATGATGATCATGATCATGCAAATCTTCAAAAGGGTGCGAAAGGTGAGAAAGCTTTAATGGCGTTGAGAAATCACAGCGAGGCGGAAAGAAGAAGGAGGGAGAGGATTAATGGACATCTTTCTATGCTTCGTAGTCTCGTCCCGGGCACTACTAAGGTAAATTACTTGTTTCTGGCATGATTTAGTTCATGCTGTTTTAATTTTTTTTTGGGTTTCATAGTGTTTGAATATTTTATATGGTTATTAATTATTTAGGATAAGTTGGCTCATATAGTTTGATCATTTAGGGGAATAGACGAATAGTCATTGGCTACATTTTGACTTTGGTGGGATCAAACTTTTTGCTTTAATGGGTGTTTGGGATTGGCTATATGGGAAATATTAGTACTTATTTGTGTATTTTACATCAAAATGATCAGTTCAAAATAAGTAGGCTGATATTAACTTATTTTTAAGCCGATAAGCACTTTAAAATAAGCAATCGCACACCATTTTAATTTATTATTTATTTTGGAGAAAAAAAAAAAAAAAAAAAAAAAAACTCGCTTTTAAAAGAAATTGAAAGGAGAGGTATGATGACACAAGGTTTACGGCTTCGATTATAGGATACAATAAATTTTGCTAACTTACCCAAAATTACTGAACTGCTTCGAGTAACTTACAGGACCATGCCTATAGGCTTCTGTTAAAACCAAATTACCTATTCAACCGTTAATGTAGAGATATGAGCTACCAAAAAAACTAGATAATTATAAATCATCACTTCAAATTCTTAAGAAATTTTCAGTTACATAGGGATTAGTATCAATATGGAATCTTCCAATTTTTTTGATCCAAAGAGCCAGATTAGTTCCCATTTTCCAAGTTTTGGCAGTGTTATATGAACTATTCATAACTTGGAACCTAATGAGATTGATAGATAATTATATCTATCACTTGAAGTCTGACAAAAATTTCAGTTAACCATGTGATTACAATCTTTGGCTAGTTTAGCGCGATACTTATAATACGCAATTAATAGAGCTACAAACTATAACTAATTATGCTAAACGTATATAATTAATCTGATAAACAATTAAACTTGCTTATGATGTATTTTCGTAAAAATGCATCCCTATCGGAAACATGCTTATGATGTTCGGGAGTATTATAGCTTTCTCGCTGTTTGTCCTTTTTCTGTAATATGATTGAATATATATATCAAAAGAGCGATGAGCCTCGTTTTAGGGGTGCTTTAATGTATTTTTATTATAATTAAGATTTTTATACGCGGATTGATTCATCAAGATAATCATCGTTCAAGAAGTTTAGCGACAACGTTAGAAACTTCACCATTCATCCTTCAATTCCATAAATAGTTTTACTCCACATTCTATTAGTTTGACCTTAAGTAACCTGAAAACCGTATGATATTAAACTTGAGCAGAAGATGTTAAAAGTTGTAGTTTATTGGTTTGCATGTAACTGTAGCTGAACTTTATTTATGAATAAACAGATGGACAAAGCCTCATTACTTGCTGAAGTTATCTCCCATTTGAAGCATATGAGGATGACCACCACTGAAGCCACTAAAGGTGTTCTTATACCAATGGATATAGATGAAGTAAAAGTTGAACAACAAGATGAAAACAGTTTGGATGGATCCTCATATTCCATAAGAGCATCTCTATGTTGTGAGTACAAACATGAGGTTTTATTAGATTTAAAAGAAGCTCTTGATGGCCTCCAATTAAAAACAATTCGTGCAGAAATAGCGACCTTAGGAAGCAGGATGATGAACCTTTTTGTAATTACGGGTAGCAAAGATGAAGTAAATATCAAAGATATCGTGAGTTCTATACGCCAAGCACTCAAATCTGTACTTGATAAATTCTACGCTTCACAAGAGTTCTCAGAAAGTAATTCACTTTCAAACAAACGACGGAGGGTGTCTTTTTTTACACCCTCAAGCTCGTCTTCGTTAGGAGACTTCTGGTGAATGAGGTGATATCTTTATAACCTAAATAGATTGTGTATTAGTATATGCTTGCGCATATGAAGTCATTTTACGCATTCCATTAATTAGTGATGCATAATCTGTATGTTAAATAACTTATTGCAAGTTTAGTTGTAGATAATGAATCTCCCATTGTTCATTATTCTTATCTTCATGATCAATGCTTATATATATATATTCTATTTCCAAGGTTCTGAGCTCCTTCATCTTTATTATGATAGCTTGTTTTTTACTACTTTTTAAAACATCTCCAATGCATCCAGGTATTGCAAAATATGTTTTCATAAGAAAACTAATTTTCGAAAAATTACTACAATGAAAAAAAAAAAAGGAAAAAAAGGGTTTTTAACAGAATTTTATTAGTACATATATTATTAACATATTTATATGTGTGAGATGAGTTTGATAGAGTTTTTCCTTCAATATTTGAGCAT

AabHLH30

GAATTTTACATTGATAAATACACATGTTTTTGTTGCCTGAAACTCAAATAAATATTAGCTTGTTATCATGTCGATCATCAAATATTAAACTGAAGAATCATTTTGCACGTAAACAAGATTTATTTGACTTGTTACTATCAAATAGGATTTGGATTATAAAAATCGACTAATGATAATTCAGTTTATTTAAAGTAGTATTAAAAACTCAAATAATTGCATATAAAACCTAACCATGATTCACTGTAGATGTAGTGAGGGTGTGAAATGATGTTTCCATGGGTTGGATATTGGTCCACTGCTCAGTGTGAATACAGATAGATGGGTTTCCATATGTTCAAACTATAATATTATAATCAAATAATCAAATAATTAAACCCAATAAATAATACACTCAAATTGAAAGAAATATATAGCTGGTGTGGTAGCCATATTATTTTATTATTAATAAAAAAGATGCTTTTTAAATAATTGGAAAGAGTACAAGAAATCTTGATAAACTGGTCATCGTCCCTACTGCCGAAGATCTCACAACCAAATGTATCTCTAGAAATACTATTTAACATGTACTTTATATTCATATTTCTAAAATACAGTGGTTGATCAAATCTTTTTGGGGCTTAATTAAAGATTAGCATCATATCTTAATGATATTATGTTCTCTTTCCAGCAAAGTGATGAGTTGGTATTTCATCAGATTCCTTCCTTAATTTGCTTCCAACAACCGGCAAGAAATCAACAAGATCTTATAGTGGAGGATAAGGATCATGTTACGATGGAGGGGAATACAAATAAGTCGGCCAGCAAGACCCGAAAAAAGCAAGAAGATCAGTCGCCTTCCTCGTCTAAACCTAATTCGGCAACTCCGGAAGATAGTACTAAAGATGAGCATACACAAAGAAAATTGGTACATAGAGAGATCGAAAGACAGCGTAGGCAAGATATGGCTAAGCTCTATGCTTCACTTAGGGGTCTACTTCCACTCGAATTCGTCAAGGTAAGCTACTACAACTTCAAGCTCTTGGTAATTACTAATTAGTTGTGTCTATGAAATAAGAGTTCTAGCTTGAGAATGAAAATGAATTGGAATTACAATCTATTGTTCAAAAACCTAATTTATAAGTTATTGGATTTCTTTTTTTTAGGGAAAGCGGTCTACATCGGATCATATGCATCAGGCAGTGAACTATATCAAACACATGCAAGAAAACATCAAAGAAATGACTGTCAAGCGAGACCAGCTCAAGAAATTCGTGGAGATGAGTGTTAGTGGGCTTGGGACAAATTCTAATGAAAAAAACTTAACGAACCTGCTTCCTAACACAGTTTCACTCCACTCTTCTAATGGGGGAATTCAGATTTCCATCAATAGTTGTCCTTTCGAAGAGGGGTTCCCTCTTTCAAGAATACTAAAAGCGATTTCCAAAGAAGGTTTTAAGGTTATAACCTGCACATGTACTAAAGTAAATGATCGGTTGATTCATTCTATCCAAGCCGAGGTAAATTATGATCCCTTATTACATAGATACATAGTGCTAATTGTTCAGATTTTTCAGCATATACCTAGATATTGTTAGAAAAACAATCAATCTAGCACTCATTTGTCAGAATATTTTGCAGGCAAATGATCCCGTTTTGACTGATCTCTCCATGTTGCAACAAAGGTTAACTGTGGCAGCGAATAACTACTGATTTTCAAGAAAAAATGTTGAACTAATTATGGTAAAATTGCTTGCATGAGTTCATTATGGAGATGTTTGCCACCATCCATTTGTTTGTTCATACACAAATCTTTATGGTGAACTTCCATAAAGCTTGTTTTGATGAACATACATGTAGGATTTGTGGTTTGTTTTGTTTTTTGTATCCTTTTCATAATATATATCTGTGTAAGTGTTGCCATTTTTTTTATCAGTTGGTACAATGTTGTTATATCAATAGTATGATCTATTAGCATTACAAGCGTGTTTTCCTATGTGAAGTTCATTACTCAATATCTTTCTTAACTTGACATGTTTCTTGTTTGTAACTGATNTTATTATTAATCATCACTTTATTAAAAACTCATAGGATTTACAAGAAATTGGTGGGAAGCCGAGAAACCAACTGGGCCCCCACACCTCTAGCAATAGCAAAACCGATCCTATTAAAAATATGAGCCGCAGCACGTGCCCCAATGTCTTGAGTCATAGAGAATCGTCGAATACGCTTCAGAAGGGTTACTGCATCTTTCTCAAGTTCTCCAAAAGAAGAAAAAGAGAAAGGGAGGAAACCATACCCGATATCAACACATTTGGCACCGTACTTGACACGCTTACGCTGTGCGGCATCAATAACCGCCCGCCCTGGCAAAAAATCATTATTATTATTATTATTATTATTATTATTATTATTATTATTATTATTATTATTATTATTATTATTAAATAAAATTTGCTATTATGAATAATTACAAAAAAAAAA

AabHLH31

TTTTGACTTTTAATAATTCTATTATTGTTGGAGTAGACTTTTTATTTCTTTTTTATTTATTCAATAATGACCTAGGCTGCCTAAGTGGCAATTATTAGGGTGTCGACGAAATGTTTAGTCATTATATATAGAACAAGCTAGGAAAGGAGTTGTGTCCTTCACATCCATGTATATTTCTTAAACAAAATCTTGTCCCATTTGTCACATACACACACCCCCAAGAATCTTGAGTCCACTTTTTTCTATATTCATTATCGATTTAAGCAAGTGAATCTTTATTCAACTCATCGAAATGCTCGAATTCAAGACTATTAAGAAGAAAATGTGGATGTTCTCCCTATAAAAACTTTTGTTTATAACAAAACCCTCTTGTCTTTTACCCTCTCATGGACAAAAATATGATCACAAACTCATCATCTTCTATAGTTTTCGTTAAGCTCTTCAGATTCCCTCTTCGTATCGTTTCTTTTTGGTTTGCAAACACCATTCGTCCTCCTGGTGTCGACCCTTAATTAGTTTCGCGTACTTTTACTTTTATTGCACTCAATATTACACACAAACTTATAAAAAAATATATATATACGTATAACTTTAAACGGTATTTTAAAGAATGGAATTATATGGTTGGACTAATCCAGGGTTCTCAAACTTGGGACCAAATGGATTTGATGGATCTTTGATGAACTCAAGTGCGTTTAACGAGGACTATGGAAATGTTGATGGTTTGTTCATGAAGTCTAGTGAATCTTTGGTTTTGGACAACGAAAAAAGTGAGCTTGTTAAGGGTCAAAGTAAAGTGATTGGAAAGAAAATAGGATCAATATCTGATGAGAAAGCTGTGGCTGCTTTGAAGAGTCATAGCGAGGCTGAGAGAAGGAGAAGAGAAAGGATTAATGCTCATCTTGATACTCTTCGAGGCCTTGTACCATGCAACAATAAGGTATTCTTCTATAGTACTCTTTAGTTGATTACTTGATTGTATAACTTGATCATGAATGTGAACGTTTAATAATCTACAAAATCACACTAACTTTCATTCCATTTCTTTTTCTTTTCCTCTTTAAGTGCTACTCGATAACACGACTTCTTATGTTTCCTTTTCATTTTCTTTCTTTTTCTTCATAATAATATTGATCAAAACAATGGTTAGAAAAGATATTGTTTGGATGTACGTTTGTAGTTTGAATTTGTGATATCGAGAATTTGAGATAATCGGCGAAGTTTGGTGAACTCTAATTAGCTTAGGATTATACACGTAAATATACCTTGGATGTTAAGATAACCAGAATAATTTCTGTTGCAACTAATCCTAAGTTGACTTCCAGAAACTGATCATTTGGCCCGTGTTAAACACAATCAATTTAACGATACTCAAACACTAGAAACCGAACCATTGGAGCTTTAGTTCAATGGGTGGAGCTCTCGTGCACGAGTCAATAGACCCGGGTTCGAGTCACACCCTTGTCCTAAAGTTCTCAATGGTACCATCCCCCACCGCAAATGCTCAGATTGACTGGTCAACGAGGGGCTTATTGGAGAAAATGACCAGAGTTTAAAGCCCACCTCCTGATAAGTACAGTCACTGGGACAAGTGCTCATTGCCTGGGGCCGGGTCCTTGGGGGAGGGTCCGGTTGGCATCCTTAGGATGCATGAGACCAGGGTTATCCCACCGTTCTTCACCTTTTGGAAAAATACTAGAAACCGATACGTGAAAACCGAATAATTCTTACTGAGACATGTTTTAATCTTTGCGGTCAAATCCTTTATTTTAATATCTATGAATGTGGTTTCAAAATGTTATTTCTAGTTTTGGAAGAGGATATACTGCTACTACACAAATAGATAAAACTCTTATTCTAAGTTGGGTAATGATATTTGATTTGGTTACAGCATGCTACACCTTGACATCTCTAATAGATGTTCAGTTTAATATGGTAGTTTAATGAAACACATATTATGATATGCTATATAGTTTTATTGGAAAGTTTGGTTTACATAGAACTATAGAAGGTATAGTCACCAAAAAGTTAAAGTCGAGCCTTCTTTATATAATCATGATCGATGACTCTCACACTTTAACAGCCAGGGGGGATCAATTGTTGACTTCAATTAGTGAATATTCCTAAGTCATCTAATATAGCATCACAGCTAAAAATCAAGAGTTTGCTTGCTTCCTTTATATATTTTTGGACTTTTGGTGTGTATTTGATAAATGGAACTTGTAAGTTGTATATCACTTTCAAAGTTTCAATGTTCTTGTCATCGTAATTACTTGCTCCATTGCTTACAATTGTGTCGTTTTCAAACCACATCAAACTTTTGATGGCGGCCGAAGTTATCTTTTGTTTAGTGTATGTCAATTTGAGATTGATGTTGATCTCATTTTTATTAAATGTTTGCAAGACTAGCAAAATTTCTATGTGTTACCTATTACTATCTTTGTCATATTGAACCTTCTTTTCTACTAAGATTTCAGCCAACAACTTTGATATAAGTGTCGATCTACCTCCGTCAAGTTGCTAAATTTGATTTTCTTTTCCTAAATACTTATCAAATCAAGTTTTGCAATTTTCTGAAGGATATATAAACTAATGCTATGGAAAATCACGTTCAATAACCACCAAAAGTTGATATGATATTTAAATGCTGGAAATTGATGTTGATCTCATTTTTTAAATGTTGGCAAGAGTCTCGGAAGTTCTTTTTTTTTTATTATCAGAAGGCAACAAATGTTGTTTGTAAACGACACTTATTGGCAGGGTGGCATTTCATAGGTTATAATATTTAAATGTCAACGACATATAATCTTGTATAGTTGTAATTGTACAATTATTTCAATAAATTATAAGGTCAATTTTTCCCTAATTAGTTAGTTAACAACTTTGGTTTGTTATGTATATTTAAAAGAGGTTCAGTAAGTCAAAGAAGCAACTTTTATCACTTTTGGAAATTAAATTTTCAATAGCTCACATTTTTTATATTACATCTGTTTGTATACTTGTAGAGTTGTAGCAAGAGAAAACATACAGCTTACTTGTTTTGTAAAAAACTCTATGAACCTCTATATCTATGCCATTATTAAATAACTCCATTTAATCCCAAGTAAATGAGCTAACACTTTGACTTTTTGAGGTCAAAACTATCACTTTTGGAACTGAAAAAGTCATGGTACACTCAATTTTAAAAGGTTGAAAATTAGCGCACTCAATACGAAATCAAAAGTGTATATTACATTTAAGATTGAAGTTAAACTAAGATTTTGGGTTAATTATAACAGATGGACAAAGCCACATTACTCGCCGAAGTTATCCGCCAAATAAAACAACTGAAAGTAAATGCAACACAAGCTAGCACAGGGTTACTCATGCCAGAAGACGTCGATGAAGTAAAAATTGAAAAGCTTGACCAACTTTCAGTCAATGGAAGTATCATTTTTCACGCGTCTTTCGGTTGCAAGCACCGTCCCGAGCTCCTAACAGATGTAAGAAAAGCCCTAGTTGACCTCAAAGTCCACATGGAAAGGGCACAACTGTCATATCTAGGAGACCATGTTAAAATAATTTTTGATTTCATGGCGAATGAAGATCTAGTTACTTCTGTTCGTGAAGCTCTTACAGCTGTTATAGAAAAGGGGTCAATCTCACCCGAATACTCCCTACGAACACTGCCTAACAAGAGGAGGAGGTATTGTCTATAACATGTCAAAGTCATCATGATAAACGAGGCTCAGGTTGCCTTTGTTTCACATGTTTTTTCATTGTGAAATATCAAATATGAAGTTTATGTATAAAGTTTAATATGTTTGTGATGGTAAAACTAGATAAGTAGATATATTTGTTATTGAAGTGTTAAAGTACGGGGTCTTTCTTTCTTGAGTGGAACTGTGGAAGTGTTTGTTTGTAACTGAAGCTTTTTGATCCTACAATGGTGATATGATCTTCTTAGAGCATCCACACAATGGTGAAGATTTTAAAATGGTTTATAACATGTCAAATCATCAATTTATAAAACTTTTGATACTATAAACCTATTACCACCATGAATAAACTTTTTACATTTATATCAAATGTTTAATCATAACACACGTACATTCAATTATGAAGAGGTCTAGGATTGTGGAGCTCTTCATGCCTCATAAATATAAATCACAATCTCACTATTGTTGAAACAAAAGATAAACACTTTCATACTTTTTAGACCTTTTCATAAACCTCTCATAGTTGATGCCCTTATATGTATCTTGTACTTGTAAACATATGGAAAGGTGAATATTAAGAAAGCTTATACTTAGTTAATTTGGTGTGTTCACAAATCGAGTAATATTTATTAAATTTGATAAAACTCCATATCTCAATTCTCATTAA

AabHLH32

ATGTAGCTTACAAAAATAATCTCAGTTTTAATCTTCTACTCCATCTCCATCTCCATCTCCATCTCCAGCTACAGCTCCTGTTACTTTCGCTAAACATACCTTAAAAAAACGTTTGAGCATAAAAGTTGGTTAAGTAAGTAACAAAAAGATGATGGACTTATATGAAAAAGAAGTTAGTAAAAGGAGAAAAAATAAAAAACAAAAAGATAGTAGTATGTAAAAGGATACGTGTAAGTAAACAAGCGCCACCCAGTCACACAAACACAAACACATACAAATCCACTTTCCTCCTCCATTTTCCAAAAATTCCACCCAATAATTATTTTATATAAAAAAAAATCTATTCAAAACTGTTCACGTGTTTTCAACGCGATTGAAATATTTTTATTTCAATCGATGGAAGAAAATAATATAAATAATAATAATAATTTCTTTGATTTCAGTTTGATCGACCACTTTCCGGTTCCTGGCGGTGAACTTCCTTCTCTTGAACCGGGTTTTCACTGGTCTGCTAACTCCTTTCCCGGTTCAACCAGCGTTGTTAGGTCAGTTTTTAATGTTTGATGTTTTTTTTTTTTAATGAATCTGTATTATGTTTTTTATTTTTAATGAATCTGTATTATATTCAAATATACTTTAGTAATTTATCAGTTTATTTAATGTGTATTGAATGCATTTTTTTTTATTAAATAAGAAAGTTTGTGTTTATTAATTACGCGCTAACTTGTGCCCAGTGGGTACTTGTCAGTAAAACCCATTTTTTCTTATAGGGATTTGCTAATGGGTGCCCACTAGGCATTGTTTTAAGTATAAACATATGGAAAGTTTGAGTGTATTTTAAAAATCATATTTAATTATAGCAAATTTTACATTTATTTAATGTTCATTAAATGCATTTTTGATTGAAAAAAGGAAATTTGAGTGTATTAATTACATATTAATTGCATGTATTTATAGTTTAAAAGATGCCCACTAGGCACTCGTTAGCAAATTTTACATATGGAAATTTTACTCTTAAAGACTTAAGAAGTTTAAACAATGTAACATAAATAGCATATGCATCTCTACGTGGGTGTTCGGCGTTGGTTATTTTATTTGCTTTTTTGTTTAGAGAATAAGCAAATTCAAATATTAGTTTAGAAGTACTTATTTTTAAGCAGAAAAAAAAAAAAAAAAAAAAAAAAGCAATCGCAAACACCCTCTAAGTTACATGATAGTATACTACGTCTTAGGACTAAGGGGGTGGGTGTGGATTAGCTTATTTAGCAGGTTTGAGGGCTTTTTTTATTGAACTTGGAGAAGCTCTTAAGCCGAAAAAATTGAGCTTATAAGCTTTTCCTAATTCATATAAGCCCACAAACGGGCTATATCAGCTAATCCAAACACATCTTAAGTAAATCACATTGAACATGAAGGTCCAACTACCTCTAAGAAACAACATTTTTGCATTAACCGACTAGTGTTTTTTTTATTTTTATTTTTTTTGGGGAAAAAAAAAGAGAGAAAGGTTTACGGCCAAATTTATTAGATTATTGAATTCATGATATTAACTTTGAAAAAGTGGGTCTTAAGTGGATATAATGGGTGGGTAGAATGGTAGATAGAAAGCTGTCCTGATAAAATATCGATAAACGGCATTTATAATTTTGTTAAACTTAATGCCAAAATGGTTGATTAGAATACCCCACACTTGTTTATTGTGTACCATATGATTTTTTACAACATTTATATATCATGATTAATTTTTTTATTGTCGAAATAACATGCATTTTGTTTTATTTTTTCAGTAAAGGATTTTCTGATTCTTATGGACAATCGGATGCTACCAAGGAGGACGAGTCACGAAAGAGGTTTAATTTCCTTTTTGTTTCCAAAGGCTTCTTTTTTGGACACGTTGTTATATAAAAGGCGACTTTTAATGTAGGGCTCATCCTGGATCGTGCTCTAGTACAAAAGCATGTCGAGAGAAGAAAAGGAGGGACAAACTAAACGAGAGGCACGCTTTTAGCTTTTATGTCTTTTTTGTGCTTTTCCACGTGCTTGTGAATATTTACTTTCTGATATCTGGAAAAGGTAATGTGCTTAACTGTGACTGTTGTTTGAACAGGTTTCAAGAATTGAATGAAATACTGGACCCTGGAAGGTCAGCTAAGACGGATAAGACGGTTATTTTGGCGGATGCTATTCGAATGATCACACACTTGAGAAATGAAGCCACAAATCTTAAGGACTCATCTCAAGATTTGCTCGTTAAAATCAATGAGCTGAAGGTAAATTATCATCTGAGCACTTTTTGTTTGCTTCCGTTTATTTTTCCAATAAAAGATGTGGTTTGACTTTTGAGGCTGAGTAGAGAGAATTTTCAACCCAATATATGCTATCCATTCTTAGAAATTTCTGCATATTGACAACGGAGTCTGTCTTCTTACCGAGTGTGACTTGTGGATGTATGTTGGTCATAGATGGGAATATACACAATCCATCATAAACTTGAGTCTGATTGGTAGTATTTTTGACCTAAAGAGTCAAAATGTACTTTTTTGTGGGATCTAATCTGGGCTTTATACTAAATATCTGATACGAGCCCTAAACTATAATATTATTTTATATTTATATTATAGTTTAGTGGTCGTAATAATTATCAATATCTAGACCTATTCAAGACCATCGGTCGTAGCTTAACATTTTTAATTTTGGGCTGGTCATTCTGACTGGCCTTGGGCGTTCTAGATAATAATGTAATTCTTGCAAATATATTTGACACCTTAATTACTTATTAAATATTTAATTTTTTGTGTGTGTACATTAGGGGTTTTTTTAGCTCAAGAACTTTTGAATCCTACAAATTACTCATTTTGACTAACCTGTTTTACTTTACCCAAAAAGGATCTTTTTGTAAGGAATAGTCTCTATATATGTTTTTTCATAAACTTTCCATAATTGGCAGGTTGAGAAGAACGAGCTGAGAGACGAGAAGCAAAAGTTGAAGACAGACAAAGAGAGACTAGAACAACAGCTGAAATCCACCTTTTGTGGTCCTCCAACGGCATTCTATCCTCCTGCACATCCCGTAATGCCGGTCCCATGTCCAGGCCCCACCCCCGTTGGTGGAAACAAGTTCATGCCATACATGGGATTCCAAGGGGTTCCCATGTGGCAATTTGCTCCCCCAGCTGCGGTTGATACTTCCAAAGATCATGTTCATCGATCACCACTTGCATAGAAGAAATTATGCCTTTGATGGGAAAGTAATTATGTGAAAATAACACCCTAAAGTCTAAATTTATGAGCTATTTATGATTATGGTCATTATCAGTTTAGTGTAATTTGACTTGATGATTAAAGTTTTTAAGGATCCAGATTTTGACCTTGCTGGAATTTTACACAATGGGTTTGACTTTACTCTTGCAGTCGACACAACACAGTCAAGAGTGTAGTATCATTATCTAGAAAGTTATGTTTGCGTTGAGTAATTATTTTTGCGTGTTCTTATGCTGGATCAAACCAAATCAAACCATGGCGTTTGACTTTATTTATGTTATACTTTGTACAAATCCAGGTTACCTCGTCGTAATTAATATGTCATCATAAAATGGCTTTGACATTTTCTCCCAGTAAACTGGTTCTTTTGGCTTATAAAAAACTTGCACTTCATTAGCAAGTCAGACTGACCATGTCCAAAACTCGGGTTGAATTTGATAATTGAGTTTAACACGTATGGATTACACGTTATTTGATTTAATCAACCCCAAAACCAAGTTTAAATAAGACCCAATAGTA

AabHLH33

ACATTTCACCCATATGACTTCTACAAAAAACACGTACTAGATGAGTAGCGGTTGAGTTGTTTGTTTGTTGGATCTGATCACACATCGAAACTAAGTGTGAACTGCCCAACCATACACCACTGTATGAACTCATTAGCAAGCAGGAATATAAGAGTTGATTGTTCCTTGAAGAATTAGTAGTATATGTATATCTAATTTGATCCAAAAAAAATATGTATATCTAATAAATAACTGTTATTATAAAATTGATGAACTTCTTATGAATAAAAAACAGTTTAATTCCAAGATGATGCAAAAATGTCACTTTTATAATACAAGAGGACAGGCCTAGTTACCATTGTTTAAATACACCAACTCACATCCTCATACTTCATCCAAATTCATATTAGCAGCCATTCCCTTTACCTGAAACGAAAACAAGAAAGGATGCTAGCATTATCTCCTACATTGTTTTCCACAAATTATGAATGGCCCTTGGGGGATAACCTTGACAGAAATCACCAACAAGATTGCAATAATATTTCTATGGACGTTGAAGCAAATTCATATGATTCAGTTCTTGATTTTCCAACATATGATCAAATCCGGCAGGATTTTACACCCGAGAGTTCAAATTCATTTGGAGGAGCTATAAATGGAAATACTGGTAATCCCATGAAGGTGTCCAAAAAGCTTAATCATAATGCTAGTGAAAGAGATCGGCGTAAGAGGGTTAACGATTTATATGCATATCTTCGTTCATTGCTGCCCATATCAGCTGATCAAAAGGTAATTGAATTTAGATTTTCAGTAGTCAAATATTTTTTCAATGGATCTTAGCAAACAACTATATGTAGAGAGAAAATAAATGAATATAAAACTTAAAAGGGCACAACTATATGTAGAGAGAAAATAAATGAATATAAAACTTAAAAGGGCTGATTATCTAAAAATGCATCAAACTTGACCCTTTTTACCGTTGTATGTAGCCATGTTATTTTTTTCCTATTTTGTGACTAAACTTTCGAATTCCGTATATTGTGTGTATTTTGTGACTATTCAGCCCTGTAAAATGACCATTTTACCCTTCAAAATTTAGAAAATGTACGGGTAAAATGGTCACAAAATACACACAATGTACCAAATTCAAAAGTTTAGTCACACAATAGAAAAAAAATAACATTGCTACATACAACGGTAAAAAGTGCCAAGTTTGATACATTTTTAAGTAATTAGCCCAAAGTACAAATGTATTTGATTGACACTAGAGTTGTAAATTTCCTAATTTCTTGTTACTCATAAAACTCATTTTTGACAGAAAAAAGTAAGTATTCCAGGGACAGTATCACGGGCACTCGAATACATACCAGAACCACAAAAGGAAGTAGAGACATTAATACGTAAAAAGGAAAAACTTTCATCGTACTCAACATCATCCGCTAGTACAAGCCAAAAGAATATCGGCATCGAGGGACAAAGTTCTAAAGATGTTACAGCTAATACAAATTCATCGGTAGTTTCATCTGTGAGCATTTTAGGTGAGAAAGAAGTGGTGATACAGCTGATTTTCTCTATGGATCGTATGAACAAGAATAAGGAGATTGGCTCCTTGTCTAGGGTTTTGGCATACCTAGAGTCGGAAGAAAATGGATTTGTTTTGCTAAATTCAACAACCTTCAAATGTTCGGGAGAACATATGTTATTAAACACTCTGCATCTTCAGGTACATATACTGTCTAAAGCGCATATAATTTATACCTGTAAATTAGTTTTACTTGATTGTACATTAGGACATCATTTTCACAACCAAGTTTTTTCTACATTATACTCATAGGAAAACTCTCAACAGAGTAAATACAAATATTCTACAGACTCAAGATTTCATTTTTATATACAAATCAACAATGTCAATGATAGATCTATAAAAAGAGTCCGTTTACCACCTCAATTACCGAAGTCAATTACTATTAAATATGTCTATGAGCATTTATATTTTATTGGACTTGCTAACAGAATCAAGCTTATGTGATCATGTACCAAATGTTTGTCACAATGGACATTGGAACAGTTGACAATATTAAAATATAACTAAATATTTGGAAATAATTGCAATACCGCAATGTCTTACAATACCGCATTTTGTTCAACAGGTGCAAGGAGATAAGAAAGTGGACGTTGAAATGTTGAAGGAGAAACTCTGCCATTTCAGCCAATAAACAGATGGACTTTTACGCTAATGTCATGGCTGTAAATATAATGTCTAGTAATCAAACTTCAGCTTAGAATATACTTAGAAGTCGATAAATGGATCATTGTGGCTTTAAAATATGTCCATTTTGATTGATAGTGTATCAAGGGAAAATGATGGCAGAAAATTACCTACCTTAATCAACAACCCATATTTAATCTACTAATGACTTCAATCAAATTAAGACCCCTTAACTTGATTATGATTGTTCATTATGTTAAAAAGTTCTCATATACGCTACACCAAATTAGCACGAAAGACACACAAAGCTCGTCAACATCATTTCTAATAACATTCAGTAATTTCTTTGAACTTGATCAAAATAAAACTGCTCACATTATATCCTAGGGGTGAGCAGAAAAACCGAAAAATCCGATAACTGAAATCGAACCGAACCGAAAATAACCGAACCAAACTAATAACCGAACCGAATTTGCCAAAACCGAACCAAACCGAACTTTTAATACGGTTCTCGGTTTGGTTATGGTTTTGATAATCAAAACCGAAAACCAAACCGAAAACCGAATTCATATAAACTTATATATTTATATATATTTATATATATAAAAAGATCAATACAATGTTATATATATATATATATATATATATATATATATATATATATATATATATATATATATATATATATATATATATATATATATATATATATATATATATATTTATTTATTTATATTT

AabHLH34

TTGTGGTCTAGTGGTAAAGGCTTAAGTAGTTAAGCAAGCACAAGTGAGAGTAGTACCGGTTTGGGTGACCGACCGGGAAGTCCTCACTTGGGAACCACAAGCTTTAATGATATCAGGCGAACCGGGAATAGGAGTGGGGTTATCCCCTAAGGAGATGCTCGCGGTTGGTATCTCTGTCCGAGGATGCGATACTAACTCGAGCCAACAACTAACATCTAACAACTAACCCGCCGTTATAAAAAAAATAAAAAATAAAAAAAAAAATAAAAAAAAAAAAAAAAAAAAAAAAAAAAAAATCGTGACATCTTACTGGTTTTTACTATTTTACTTTATATAACTTTAAAAAAATATAAAAAAAGATTCCTTTGTAAAATTTCGAAGAAATTTTTGTCCTAATACGACAACACACTAATTAAACCCCCAAAATTTACTTATAAATAGTCCACTTGTCTCATGACAAATAAACATAGTACTCTTTTGTTCCTTAAACTTCACCAAACATAAACCATTTCCCACTCTTCACTTTTCAAATGGAAAACATCACTGATGTCAACTGGGAATCCATCCTTCAACAAGAAGATTATAGGTATTATTATCATTTTTGTTTCACTCATCTTAACCATATATGTTTTCGATCACTTATATATTTGAATAACTTTTGAATAACTTGATAGTCCAAATTAATCATATGTGACCAAAAAAAAGTTTACATATTTTCAACTATGGTTTTGAACTTTATGATATATATGGCTTAATTTGAGGTGATATTGACTGTTATATATAGTTGGGGTGGATTAGAGGAGACTTTTTCGGGCTACTATGATTCGAGTTCTCCAGACGGAGGACAGTCATCGTCGCCAGCATCGAAGAACATAGTTTCTGAGAGGAATAGAAGAAAGAAGCTGAATGATAGGTTGTTTGCACTTCGGGCAGTTGTTCCTAATATTAGCAAGGTTTGACTCTCCACATTTGATTATGTCTTCACTTTATTAATCATTATCCTAAAGAGCTGTTAAATATTATATGCCAAAACATATAATGTATATCTGGTTTTAGTAGTTTAGAAGATGTTATTTGCGTCACAAAGTTACAACTTTTTGTGCTTTAAATAGTTATCAAAGGACGATGCAAATTTATATATTGCTTTACAAAGACATGTGTTTAATTTAAATAATATGTTTATCTGCAATAATCAGGATGCATAATTGATAGTATCACCTTTGCTTTCTTATTATTGTTTTACTTAATTTGTTGAATCAAAAGATACTTAAGGAGAAATGATTATTTGGCCCAACTAATAAGCTTAAAAATCAGTGTATAGCAACTTACAATATGATGATGACAAATGACAAAATTCAGAGAACATATTAAGTGAGACTGAGAAACTACTCAATTTGTGATGGAAATTTCAAATGTTAGGTGTGATTTTGTTATAGTGAATTACTAACTAACGAGACATCGGGAACTTCATAGAATCATATTCTGCATAGTTTGTATAAGAAATTCTTGGACTAGAATTATAGTTCAGTAAAATCATAACTTGCGCCTTTTCTTTTCTTGAAGATGGATAAGGCGTCAATAATTAAAGACGCGATCGAGTATATCCAATTATTGCACGATCAAGAGAGAACAATCCAAGCAGAACTAATGGAACTCGAGTCTCAAAAGCTAGAGCCCGAGAATGTCGATTTCAATCAAGAGACAAAGTCGTCTATGTCAATGGAAAAATCAAAGAAGATAAGAGTTGAGCAAGCTTTTGATTCGAGTGGATCAAGCTCGTATCCCATTGAAGTTCAAGATGTAAGTCAATGCTTATTCAACATTTTAAGATGCAATGGCGAATCATATTTGATATTGATCTTAGCTGAATAATAGACTTTTAGCATCAGTTAAAAACATTTATCTTAAGATGTTAATATATTTCTTTAATGATTAAAATGAAAGTATGTTAATTTTGACAGTTACATGTGTCATACATGGGAGAGAAGACAGTGTTGGTGAACCTAACATGTAACAAAAGAAGAGATACAATTGTGAAGCTTTGTGAGGTTTTTGAATCTTTGAAGCTCAAGATTGTCACAGCCAACATCTATGCTTTCTCAGAAAGGCTTTCAAACACTCTCTTTATTCAGGTAAGCCTCATCTTTTTTTAGCTATGTTTAAATTACATCTCCACCGGTGTCTATCATTTTTCTTCTGTTATGTTTTCACCGTGACAACTAATTGTATTCTTTTCACCAAATAATATCATTCATACACATACTCTTTTAATAAAATAAAACTTGCTCGCGATTAGAACCACGGACTCCTCGACCAAATATGTTAACGAATGACAACCAAATAACACACCTATTGCTCCATAAGATAGCCTTGCAATCATGTTAATTTGCCATAAGAAGCATTTTATTAAATGGTGCAAATATATATAAAGCCTAACGACAAATATAGTCATATTGATAAACTGTACATTAACCTAAGCTTAAAGCTATGTTTATAGAAACCTTAGAGAAAAATCTTGTTCCAAATTCATGGGAGAATATAAGCTGTTTTAAGAAATAGTTAATTTGACATTGGATGTGATTTCTCAATACGTTAACAAAGCCATGTCTATTGGCTGTTAGTTTAATATATTATCAAAACTCAAAAGTATAAGTTATCCATAATTCAATTTTAAAAAGTTATTGGGTCATAAGTAATTAAGATTTGTTGATACGATTAGTATAAGTTTGAAGATTTGTTGGACAGAGAAAATGATAACAAACTCATTTTGTATTGCTAAAGATACCTTAATCAAAGCACAATTGATAACTTAATCAATGTGGTTGTATTAATATGCTACAACTAAATCACTTTATATCAATAAGCATTTTAGAAAACATAAGATACACTGTATCCTTTACTTCTTTCTTCAAGAAACATAGTGTGAGGAACGTGTTTTATTTTATATAAAGTTATTTGAATAAAATAAAGGTTAATGAATATAGAAAGGTGCTTTATTATATTATACTATCAAGTTGAATGTAATTATTTGAAATAATCATTCTAATGTTGTAGGGTTACCTAATTTGATTTTTGGAAAGTGTTGGGCATATAATCTGTTTAGTAGGTACTTAAGATCTTAAGATAATGTGTGTCTTATTTAATTTGTCTCAATACTAATTTGAATTTAATTATGAAAAGAAATATACGCTTAAGTGACATTTAGTGATATTTTCTTGTTCTTTTCTCTAAAGTGGTTTACTCTAATATTGTATTTTTGTTGCTTTTAGAAAAACTTATTCTACACTTGAAAAATTAGAATTGTTTGTTTAGCAATAAAATAAAATAAAACAAAACAATACGACCTACTATACTACTTGTCTTTATAGAAGTATACTCCCCTTCTTGAAAATTTTCAACACTGCTTATCATAAAATTTTAATCAGTTAAGCTATATGCTTTTTATTTTTACTACTGCAAAAACAACAATCATTTATTTGTTACTCAGTAAATGTTTCTTCCGCGCTTCGGGCAGGGATGAAGTTGTGATTAACTCATGTTTGAGTTTCATTGTTACTACGTACTTTTGATTATGAAAAAGAAGGTTACGGGCTAATGCAGAATTTATCAACAGCTATTTTTTTATTTAAAGATGAAATAATGATGAATACATATTACTTTCTTAGAAAAGGTTTATTATTTCAAAATGCACTATGTATCTAAGACGTTCATAAGAAATTAATTAATCTATCAAATTATTCATAAAAAATTGACGCATTTATTTTTTAATTGATACTACCTCCGTCCCAAAATATAAGTACAATTTGACTTTTTTAATCGAACTTTGACCTTAAATATTTTTGTTCGTATTATATACCACTTGATGAAAATTATTCCAAATGAAAATACATTTAAAACACTATCCGTTTATATATTTTGTATCAAATATTGTATAATACAAACAAAAATATTTAATGTCAAAGTTTAATTTAAAAAGTCAAACTGAACTTATATTTTGGGACGGAGGGAGTATACCGGTAGACAATTTCATTATCATAAGTTTTCATCATCTTTTTCATATAATATATGTTCTCACCATAACCTACATGTTTTCATATAATATGTTCTCACCATGACCTACATGTTTATAATATTAGTAACATCTTTGTCTATAATCCAAATATGACAAGGGTGTCTTGACTTTCTTTAGTATATAAAATCAAGGATTTTAACTTTTTGATTGTATCGGAATTTTATCATTTCTTTCGCATGATATTGACATCTTTAGTCACATCAGTCCTTATTAGCTTAACGAAATTATTGCTCGTGTATGTGATTTAACAACTTGATTCGTGTAACAGGCTGACGAGGAAGATACAGACGTGTTGAAGATACAATTAGAAACAGCCATATCGACCTTAAATGACCCTCATAGTCCAATGAGTATGTGATCAAATTTACTATTTTTACATTTCTGTAATTAGCTAGAGATTGTAACGTGGGCTAGGATCATAAGTCCCTTTCGTTTGCAACGACCTTAACCACACAAACCTGAGAGTTCGGTTTTGGCGTTCATCGTATTAGCGTGGCATTTCATTGTTTGCCCACTGGTTTATGTTTAGTTTCAGTTTCTGGCCTAGTCCAATTTATTTCCTAGTATAATTCGAATGTATGCATGTGTATATACATTACGTTGTGCTTTAATAAGATTTTACGAACTAACATATTGTATCACAGCCTAAAGATCCTCTCTCATAAAAACCCTAAAAATCCTATTGACCCTGCCATTAGTGGATTGTAATTTATTTAGTTTTTCTCTTTTTATTTTTTGCTTACTTTGCACTAATCTTGTCATAGCATCATGACGGGAAAAACACCACTACCAAATCCGCTTAAACAACCCATTGATAAGGCTTACTAAAATACTAGTATGAAGGCGTGTATCCTCACACCCCTTGATATGGATAAGCTAAACTATAACTCGTGGAGTGCGTTATTTAAAGGATTTTGAAAAACCTACGATGTACATCATCACCTAGCCACTCCATCCACCTACACGAGTACCACCTCTATTGATCCTCTCCATGATTCTAGTGACTCATTTGTCGTCATGTGAATGTATTTCACTATATCTCCCAAACTTATTTTTATAATCATTGATGATAGTGCAACTGCTCTTAGTGTTACAACCGAACCCGACGAGGTATTATATTATATTATATAAGTTTTATATTTTAAATTGCCATTGTTATAAGTAATATTATAATATGGTGCTAGAGTGTATTTTATAAGAAAAAAAATGTTAAGTTATAAGTTATAGTGTAAGAATGTAAAAATGTAGAAAGTGTCGTGGCAAATATAAGAAGAATGGGACTTGGGGGACCAAAATTATAATAGGCTAAACTTAACTAGATAAACATGTTTATCCAGGAGATTTCCAGAAAGTTATAGTGTGTGTGTTTGTTGATTTCGGTTCCGGGAAAAGAAAAAGAGAGGGAGGAAAAGCTACTTTGGTTCTTATGTATTTTTATTAAGATTGAAGTGTAGAATCAAGCTCTAATCTATTAAATCATCACAAGTAAGTGAATTTGTAATTTGAAATTAGTGTTCTTGAGGTGAGAATTAGGTAAAATGAGTAAAGGTTATTAATTTCTTATCCTACTATTGAATTAGATACTAATTAGGGTTTAACACCCTTATAGGAATCAAGAAGGGAGCAAAAGGAAGGCTTGTAATAATTTCAAGTAGGAATTATAGTTGGTGTAATTGAGGTAACTACATGGTTATGAACTTGTTGTAGTTAAATTTATGAATTTTATGACATTTTGCCATGTTATAGAAATATGAAGTATGTTATAGATTGCTTGGATCCATAGCTTATGTCTAATGTTATTAGCATATTTTTATTCTCTGATTAAGGCATGTATATATGTATGTTATAGTTTTACTCCTATATGCAATTGATTTATTATTCTTACTATGTACTTATTAAGTGTTTTGCTTACGAGTTGTAGTTTATGATGTTCTTGATAGGTACCGCAGAATTTAGCAAGGGTAAGGGTAAGGCGTAGTACATAAATTTAGTAGAAGTGAAGCTCTTGGAGTTGAATTTAGAAGACTATCGTCTTTTAGACCAAGCAAGTCGAAGTTGGAAGCCCTTCGAGTCTTCTTTCATATTTTGGGTATATTTTGTTAACATAGGTCATTTCGGTGTCTATCGGTCTATGTTCATGTTTATGATTAAGATTTGATAGTGTATTAGTTATAGTTTATGTTTAAAGGGGTGTTTGGCAATGTGCCGTTTTACCCATTTTGACCCGTTGTCTTGGGTTAAACCTAAACGAAACATGTGTATGTTTTAAAATGGTTATAATAGAAGTGTTTTCATGTTGAGTTCTCAAATGTTTGTGTGTTTGTGTATTGCCGATTCTGGTTTTTCTGCAGTTTTCTACGTTCAGCCAATACGGCGTATTCAGATTTTTGGATATCGTGTATTGGGCTAAGCAGCCAATACGGCGTATCCAAATTTCTGGATATGGTGTATTGGGCTCAGTAGTCAATATGGTGTATTCAGATTTCTGGAT

AabHLH35

CAAGCCTCACTCAAACAAGGTAAATTTTATGCTTTCGCAAAACCTTGCCTTAAAAAAATTGACTAGTTAATATAAATTTCTTCCTATAAGTTTTAGCACTTGAATTTTGGGATTATGGCATTTGATCTTACTTTTCTAGCATTCGAATAAGGTCAAAAAAATGCTAGCCTCCAAAACTATACTGGCAGTATGCTTCTTAAACTAACAGTTTTCCTTTAGCAATATCAGTCTCCACAGCGGCTATGATTGGTCCTTTACGCCTTAATTGCTTGACCATACATTAAGAACATAGTAATAAAATTTTATGCTAAAAAGAGCACGTTTTTGGAAGCAAGGCTGAGAAGAAACACCGTTCATTATATAATTTGACCGGCAGGAATTTTCTTATATTACACGTGCCTAAAATGAATAAACAATTGTTTAAATTTGCTTCAGCGTTTACCAAGGTATATAGACCCAAGTGTTAATCTTTATTTTAATCAGCCATAATAATTCCAGGTGGGTGGCCAGGTCCGCCCGCCCCGGGGGCGGCGGCCCTGCCGGCCCGCCGGGGGCGCGGGCCGGCCGCGCGGCCGCGGGCGCCGATGCGCGGCGGGCGGCGGCGCCCCCGCCGCCGCGGTGACCCGCCGCCGCGCCCGCCGCCCCTACTTATTATCTTTTCGAGATTCTAAACCTAAAAGCCGAGATCCCTCCAGTTTCCTGGATCTGGCTTGCTTGGCTGCCATAAAGGGTTCAACCCATGCTCAGGAATCCACTTCAGGCACAAGGATCATGTTTTTGGGCAGGAGAGGAAGGAGAAGAGAAAAAGTTGAGCCATTTCTTGGCTCTGCCATCATTCCTAACCTAACGAAGGTACTAATCTACCTAAAAACCTTTTTTTTTATTCGACATAACCGAGGTGTCTGAGTTTCAATATGTTGTCATTTATCCTATAACTCATTAATTTTATTGGGAGCAAGGTAAATGAACTGACTGCTATCACCTTTCCTTTTGCCGTAGGATGCGTTAAGGCACTCTGTGTTGGAAGATGCATCTAATATATAAAAGAACTTCAAGATCGGGTCAAGCGAGCTCGAGGAATCTCCAAGTATTAAGAGAAACCATGCTACACGAATCTGCTTTAATTGTAAAGAGATCTAGGCTTTAGTGCCTAGTGCATGATGAATATTTATTCGTCGGAATGACAACAAACTCTGAAGAGGGAGCCACTGGCACCTTACATGACATCTCCTGAAATCGGAGTGCCGGATTCAGGGGAAGGCAGTGTTCTAGTGTCAATTAAGTGCCAGAATAACCATTTCTTCTTTCACAAAAGCACTGACCATATGGGAGAAACTTTGGGTTGTCCATCATCAGTTGCAGTTCCATGGCCCTTTGCTAGACCAACCTTCTTATCGGCAAATGCTCAGGGTATATAAATTCGATTTTAAATCATTGATTCAATAACATAATTTGAGGCGAAATTAGAACGTAACTTCTAAGTAATGCTTCACATCAAACTATAGGAATGCCCAACATATGATTGTGATGAAAGTGGGTTTAGACTTTAGAGCTAATCATATTATGAATATGAGATACTTGCATCTTGCATTCAAACATAATTAACCGGAATATATACAATCTTGGTTTTGCTTAGATTAATGATCTTCTATATCTTCATCAAAGGAAAACTATCATACAAGCTTTCTATCTTGTGTAAACTAGCTAGGCAGGCGGGCGGGTACCCTGAAATAAAATTTTCGGTGCAATCATGTATTTTTTAGTTATTATATTCTTTTTGACAATAAATTTACGGATATCAAAAATTTCGTTGTGCTCTGTCCGTTCGGCGCCTCGAGGGCCGTTGGCTCGGTGTCCGTTCAGGCTCGAGGCCCGGTTGGCCCGTGGGCCCGTTCGGCTCGACCCATTTTGGCCCGTTGGGGGCCCGTTCGGGCTTGAATATTTTATAATTGTGCTTATATTGTTATTAGTGACATTTAACTGCAGGAATTGTGTTTAAGTTGGTTGCTACAATATAGCTTTGATAATTTGTATTTCTTATGGTAAGGGCTGTTTTAATAAGTGCATTAATTGTATTATTTACCAGTTAATTAATTATATTTAATGTAGATAATGATTTGTTTATTGTATTTAATGTTAGTATTAATTGTATGTTAAAATTAAATATGAGGGGAATTTATATAAGTAGTATTGGTTGTTGTACTTATAGCTTTAGAAGTTGTATTGCTTAATTTTAAGTATTTCTATTAATAGTAGGCCTTCCTCCATTATAAGATAGTTAGATAATGATTTAGTGATAATTGTAACAACCTCTAAATGTCGGTTCTTCTTTCTTTTGTTTGTGCAGATTGAGGATGACTTCTGTATGACAACAACAGAACTTGTAAAAAGCCTTCAACTAGCTTTATGATGACATAAAATAACAAGTTTAAGAAAAATATGCAATAATTAAGTACTCGAAGATCGTTAATTAATTAGTAGGTATTTGGGCGTCAATTTTAAACTTCAGAAGGACGTGAATTGAGAACATTCTCATTCCCATATTGGGTTTTTTTTCAAGTACTCCTGAAGAATCATATCTACTCGTAGCATGATTGATGGAAGGATTCATGAGCTCAATCCCGCGATGGGTTTTAATAGCTTTTTGCGTGGTGTTTACCACGTTGTTTAATATGTTTTACCCATGTGATATGCTGATGAAACTACCATTTTTTCTCCTTGAATTAGCTGTATTTGATACTTTATGTTTCTTTTTCGAGGACAATGAAGATTTGCAAAAGATTTCGTACAGTTTTCCTTTAACTTTACTTAACAAAGTCCAGATAAGCAGCTTTACATTTAAATATATAGCTCATTAATTATTGTTGATATATACGAAGTATGTATCAAATGGCTTCGTAATTGCTCTAATGAAAGGCTAATATAATTCGATATAATACGTAGGGGCTCTAAAAAATTAACACCCATAACATAACATAAAATTATGTCTGGTATAAAAAAGATGTCTAGCAAAAACATCAAATTTGGTCAAACAAATATAAGAAACAAAACAAAATAAAA

AabHLH36

GCTTTACATACTTGTACATTATGTTTAAATGATTGTAATATTGGGTGTAGGGGTGTTTTTAATGCTATTAATGTATAATTCTAAGGATTTTGGTCTGAATTTCGATTCATGCTTGTTTTTGTTGTACACAATGCTTTGCTTGTTGTAATTATGAAAATTCCTTACTTTAAAGTGTTGTAAATCTTGCATCCTCTTTTGTACATCTTAATTTTTTGGAGAGAGAGTCATTCTTCTATTATAAGTAGTATAGATAATGACCTAACTTCAATAAATAATGAGCATATTGTTATCATAACATAGCTTTGCATAAGCAAGTGCACATTTCACTATTTTAACATATATGGAAGAAAACACACTCCCAGCTTGAGTGTAGAGTGTGGATACTAAACATGAATAAATGTAAAAAATGGTTAGCTTGCATTAAGCTTGAGCATGGCACACGCTCTGCCTCAATCCATCCTCCTATTTTATAGTATTCATTTTAGTTTTATTTATTGTCTGACCTCATTAGCCAGTAGGAATACACGGTAGATTATCCGTTAATGTATTATTATAAACTTGAGGAACTACTTAGAATAAAAAACAATTTTTATCTTTGATTGTAACACAAAACATTTATATTCCAAGATGATGTAAATGCACTTTTCTAATGCAAGAGGATCACCTAGTTACCAATGTATAAATAGACCAACACATCGTCCTCATAATTTTCATTTAACCTTTCAACAACTATTCCCTCTAATAAATAAAAAACAAAAAATGCTAGCAGTACCCAGTACATTATTTTCCACATCTTATGGATGGCCCTTGGAGGATAACATCGCCCCAAATCACCAACAAGATTGCAATGATGTTTCTATCGACATTGAAGCAAACTCGTATATCTCCCTTCTTGATTTTCCATTATATGATCAAAGCCAGCATGATTGTGCACCCGAGAGTTGTTCTTCTGGAGGAGCTATAAATGGAAATATTGGTGATCCTAAGAAGGTGTCCAAAAAGCTTAATCATAACGCAAGTGAAAGAGATCGGCGTAAGAGGGTTAATGATTTATATTCATATCTTCGTTCATTGCTGCCCATATCAGCCGATCAAAAGGTACTTACAGCTATTCCGAAAAAAAAATAACCATGATAAGAGATTTATAAATTTTATAAATACTTGTTACTCATAAAACTCATTTTTGGGACAGAAAAAAGTAAGTATTCCAGGGACGGTATCGCGTGCACTAAAATACATACCTGAACTACGAAAGGAAGTAGAGACATTAATGCGTAAAAAGGAAAATCTTTCATCGTACTCAACATCCACTATTGCAAGACAAAAGAATCTCGACATTGGGAGACAAAGTACTAAAGATGCTATAATTAATACAAATTCATCAGTAGTTTCTTCTGTGAGCGTTTTAAGTGAAAAAGAAGTGGTGATACAACTGAGTTACTCTACTGATCATATGAGCAATAATAAGGAGATTGGCTCCTTGTCTAGGGTTTTAGAATACTTAGAGTCCGAAGAAAACGGATTTGTTTTGCTAAATTTAACGACCTTTAAATGCCCGGGTGAAGAGACGTTACTGAACACTCTACATCTTCAGGTACATATATAACATCTCTTCTTTACTATTTATAAAATGTTTATGCTTAAGAAATTTAAAATGGCTTAAAAGTAAATAATATATTTTGCCTAAATTAACCCTTAATACCCCTTTGTCTATTTGAAATAGCCGAGCTTTTTGTGTTTATAAATGTCGGATGTTGAGTAACAAAATAAATGAAATGTCTTCTATAAAAAAAATGTATAGTATAATAAATTTCTTTAATGAGCATTTTTGATTTCATGATTATTTATTACTTACCAAACTTTTAATATCATTATATTCATACATATTGTTAATATTCGAATTAAAACCTTTTTTAGATTTTTTACTATTGATTGATGAAAGTATATCTACGTCCAAACTCGAATCATCTCATTTCATCAATCTATACTATAAGTTTACTCAACAAAATATATTTCTAACAACACCTATATTTGAACTCGTAAGTTTTGTATTAAGAATCAAACCAAACTCACGATTACGCTAGTATAATTATTTGTCGCTTTACGTGGGTATTCATCTAGTATATCTACACATGTATATAATCCTTGTCAATTAGTTCTACTTGCTTGTAAATTAGAAGATTTTCATAAACAAGTTCTTTCTAAATATACTTGCATACAGCTAGAGACCTGGTTGCTCATTAAAACATATATGTTGAACAGGTCAAAGGTGATTATAAAATTGAGGCTGAAAAGATGAAGGAGAATCTATGCTCTCTCTACCAACAATCATATGCCTAATTTTCATCTGTTGTTGAGCAATCTAGACAGTAATCTGAAGCCTGCTATTTCTAACATAATTTGTGGTCATGAAGATAACAATAACTAGGAATGTAAAACTTGTGATTTTGTGGATAATAGTATCAATCCAATCATGAAAATTGACATACTGTATATAAGAAACTTTCAGAATTGGCTTTGTAACTTGCAATGTTAGTGAATCCCATTGGTGACGACAAAAATGGTATTAGAGCTAGGTTTGTAGAACTCGAAAAAATCTTTTTTACAATTCAAATGCGGACATAATAATGGCTACCTTGCAGACCCACCCCTGGAACAAGGGATGGAGGTGTAAGGGGATAGCTTGGCTACAGTTTTTGTAGCCAATTGGCTACAATCATTCATAAAAAATACACTAGCATTGAATCAGGGACACACAAAAAAAATCATGACCCACAAAATGAATCGGGGACCTACATTTA

AabHLH37

CAAAAAAAAAAAAATGTGTTTAATTTGTTCATTTTCTTTGTTTTGTGTAGCTTTTTTTTTCTTGTTAAAACACAAACTTTAATTTCAAATTTCGACTTTTGTGTGTATTTTATAATCACAATTTTACCTTTACGTTTTATAATCACCTTATTAACATCGAATGGTAAAATGATCATTGTGCATGAACCAAATACACACAAATGTCGAAATTTAAAAGATTTGTGTTTTAGTAGACAAAAAATGCTATACAGCACACAAAAATGGATAAATTAGATGCATTTTTTTAATTATCCCTGCTTTTACTTTTGAACTGATTATAAGTAAATTAATACACAAAGTTTTCTTTATCATCTGCATTTTCTAATCAGACCTCGTAATCATTGCTAAAATAACAAAGAAGCAAGCTTGGAATCAAAAAAAGATGCTGGTAATATCCCACCCCTTGCTTACATCAACCTAGATGGTGTTTGAAGGACCTTAATATATACTCGAAATCATCAAATGTTTGCAGCAACATAGATTCATATTTCTTTCTTAATATTCATATATATAATTAAATCATGCAAGATTTTGCAACTGAAAACTCTATGTCTTCTGGCGTAGATGCAAATGGTGGTACCGGAGACGACACAAAGGTAGCAAAAAAGCTCAATCATAATGCAGGTGAAAGAGATCGTCGCAAGCGGGCTAATGATTTGTATTCATATCTCCGTTCACTGCTACCCATATCAAGTGATCAAAAAGTACATATTATCTTACGTCTTATGAATTTAAATTGGGAATGATCTAATAAAGTAGACTAAGCTTATGTTATATAATTTTGAATTAGGAGTTATCTTATTATAAATGAACTAAACTTATGTCTTATGATTTAAATTAGGAACTATCTTACAAATAGACTAAACATAGTTTCAACATGTGACTGGCATGCAAGTTACATTATGTCCTATACTTGCAAGTGGTTTAAGTAATTTATTGGAACTAAACTCATACTTAGTACTTATATAATTTCCTTTGCAACAGAAAAAAGTAAGCATTCCTGGGATAGTATCGCATGCACTGAAATATATACCTGAGCTACAAAAGGAAGTGGTGACATTAAAGCGTAAAAAAGAGAAGGCTCAGTCATCTTCATCACAAACCATGAATAGCAGCTGGCAAGAGCATCGTGCCATTAAGAAGGAAAATTGTAATGGTACTACAACAAATAGAGATTCTTGGTTAGTTTCTTCGGTGAATGTTTTAAGTGACAGAGAGGTTGTCATCCAGCTGATTTCCTCGACTGATCATATGGGCACAAATAAGAAGAATGGTTTTTTGTCTAAGGTTCTGGACAACTTAGAGCGTGACGAAAATGAATTTGTTTTGCTAAATGCGACAACAATGAAATGTTCTGGAGAAGGGATGGTACTAAACACTCTTCATCTTCAGGTATATTTATCAATTTCTGTCTCACAAATTACAAACAATTTAGTCATGCATGTTCGACTAAGTCAATCCAAAATATGCCCGGGGCGGACACGAGGGCGTGAATCACATGAATTTTTGCCGCATAGTGTAATTTTGGTCAAAAATCCCAATTTCTCTGAAACAAACTTGTCCTCCCAAAATTTTATGTTACATGCTTTATTTAGTTACTCAACTCCACTGTATTATCGTAAAAAGATAGTCTTGGTCCATCACAGAAAATATGCATATGTCACATGTCTTTCACTAGTAATATTATACATCAATACATACAACTTGCCATTGATTTAGAAAATAATACTAGTATAGGTCAAACATTATATATAAATTATCTTGACGCTATTACGTGCTATCGATTATAAAAATACCAATAAATGCATTCATTTTTATATATATATTATTGAGTTCAGCAACAGCAAAACAAGTGCTTTATTTGAGCTTAAACTAATAAGTAGATCAAACATAATTTCTCTTAATTGAGCATTAAGTAGTTTTTATTTTTGGGAAATTAGTATTTTAAGAAGATTAATATACAATGACAATGGTTGAAACATCAGGAATACGTTTTCTTTTTCTAAAACAACCAATATATCATATAAAAATACCCAAGGATATTTAAAGCCCATTCAGTTTGCAATCTAAGAATATTCTATTAGGAGTAGTTTTACTTTAAATCCAACAAAAGATTGCCTTTGAACTTTGTGATCATAATAAGTTTTACTTGACAATTATTCGTTGTGTGGGACAGGTGCAAGGGGATCACAAGATAGCGAGCGAAAAGTTGAAGGAACATATGTACTCTTTCTATCAAAAAGTATATGAGACTTTACTCTAAAAAGCTGTTTGCTACTATACGAGGGAGATCATTGGGAATGATATTGTAGTAGAGATAGATACGTAGTAATAAACTTGAAAAAATAAGCACATATTGATTTACAGTTTAGAAATGATTCTTGTGTTTCATTGTGTTATGTAATGGAATGAATGCAATGAAGATGTAAACAATAGGCGTTTCGCCTAACGGTATTTATGTGAACGTGAGTTTTTCTAATCAGATGGTTTTCACCTCAAGTCTCAAATAAAACAGATCATGATATGTGGTTTTTTTTTTTTTTTTTTTT

AabHLH38

AAGTGATGTAATACTAAGATAGATGTATGATGATGTCGTGTTACCATTACACCGATTATAACATCCTTCCTCAAGGAATGGAAAACTCACACGGAACATGCATGATTATAATGGCAAAATGAAGAATCAAATTCCACGAAGAATTGCCTTTTGTGTCACGTGTATCAGCTGATAAGTAATTATTATACAACTATCCATCAATAACAACATAAACTTCCAAGTAATTATTCTCTGTGGTCATATTGCCAAAGCAGAAATATTCCACGAAAAAGCTGATTACTAAATAGTACTGTAGTCTTTCTTTTTCATAAAAGGTTGTTTTTATATAGTCTCTCTTTATCACAGCTCTTATTGAAGGTTCACTTTCATAATCATTACATCAAATCATGGCAGAATAACTCATTATATACCCTAATCTGGGTGTCTCTTTGCTTTCATTCCAAACTTGAGTAATTAAAAAGCAGTCTCTTGAATGAAACATATTTTTCACTTTTTCTGAAAGAGTTTATGCCTTCAAAAGCTTCTGTAAACAAGAGTGTCAATTTTGACATTGCAAACTTGTGAAAAATTCTGTAAGAAGTACTATATCTTTAAAGTTGATCTTAAGTAAAGTGCGCATCTGGCAAACATTCTATCTATATTATCAGTCTAACTAAAATTAACATTCTCATTATAAGGCCATGGATATGCCGTCAGCTTGGCTACCAGAACTGGTAAGTATAATATAGTAATCGCTCGTAAGTTTCGCACTGATAGTATATGTACTCTTGCTTTATTTATGTGCTAATATTTTTGTTTGTTGAATTCATATTCTAGGAAATGCAGGAACAAGGGTTCATAAATCAGTATCAGATGAACAAACCTTATCATCCTCTAATGGATGACTTCAGTGTTGATTCGTTTTCCTCAGAAAGCTACACAGAAAACCCATCTTTTATCGATCAATCTTTTCAAACTCGTAAGGGAGTTGAAGAACAAGCTGATATTAAGCAGCCGTCTAGCTACAAAAAGGCCAATAGTATCAACAATAAATTTACTCCAATCAATCAAATACCGAAACCAAAGCGTGTTTCTGATCCTCCTAACACTTTCACTATATCTTTTGGAGATATAAAACCAAAAGATGAAATACTTTCTTTTAGTGATTCATATGACCTCACAGGAGATGGTGCCAACAAGGTCCCTGCGATGATTAGGAATCCAATTCAGGTTCAAGATCACGTGCTAGCCGAGAGGAAGAGAAGAGAAAAGTTGGCTCAGCGGTTTATTTCTTTGTCTTCTCTCCTTCCAGACCTGAAGAAGGTATTTCCTACCCTCGAAATTAATGAAGTGGTCTTTGGGGATGGTTATTAAATATGATTATATATTTATTGCTGTTTCAAAGAAGCAGTTTGGATAATCAACTCCAAACCCTATATTAAGCAAAAGTTATTTCACTCACGAATAGATAAATAATCAAATTAATAAGCAATCCCATACACCTTCTGAATTTGTTCTTATAATCACATACGTACAACAAAATATTAGATTGAACATACTAATGATCTAGCTAACTATCTGGTGATAGATGGATAAGGCAACTGTATTAGAAGATGCAGCTAATTACATTCAAGAACTTCAAGGTCGAGTGAAGGAACTCGAGGAATTATCAGGCTTGAAGCGAAAGAACATGCAAGAATCAGTTATATCTGCAAAGAGATCAAGGCTTAGTTGTAGTGACGATGATGGTTCTTCCTCTAACACAGCAAACTTGGAAGAGAGTAGCAGTCCAATAAATCCAGAAATTGAAGTTAAGATGTCTGGATCCTGTATGCTAATCGAAATCTATAGCCACAAAAACTGCACATCACTAATGAAAGTGTTGAGTGAGATGCAGAGGCTTGGTTTATCTGTTACCAGTAGCAGCACCATGCCGTTTGCTGATACCACTCTTCTTATCACCATTGTTGCTCAGGTATAATGTTGAACCCTACTTTCAGCATAGAAATCAAATCATTCTCGCATTATTGGATTACGCATAGCTATATGATTTATCAGCCTACTTGGTCTGCTTGCTCACTCTTATTGTACATTGTTCTTTTGTATTTTCTCAAGCTTGTTGCTTGAGTCTTAAAAAAATTTCACCCTTCAAAAAATAAAACAATTTTCGACCTAAATTAAAATTAATTCCTAATTTTGTTTCTTTATAAAATGTAAGAAATATAAATGAAATAGTCTTATTCAACGTAAACGGAGTAAATAAACAGTGTTCTCAATATATATATTTTTTTTTTTGAATTTGTGCAGAAGAGTGATGATTTCATTATGTCATCAACAGATCTTGTGAAGAACCTAAAACTAGTTATTTGAACTTCTGCCAGACGAAGAACTTGTGCCAAACTAAAGGATTATACGATCTTTGATAACTCATTGAACAACGCCGTCGTAGCATATCCGTCGTAGCATATGAATGTGGACAGGCTTCAGCCACTTAGAAAAGAAAAAGGTTAAGATAAATTTCGTCCCCTTGATTCAGTATTTTCAAGTACTCCAGACAAAGCACGAGGGTCCCAAGGAGAGTAGATTAAATCAAAGTTCGAATTCATAATATATCTTCTGTATTTGCACAGTATATAACTTGAGTTCCTTCTATATTACTGCATTAGCCTCCTCTAATCTTAATACAATTTCAGATTCTGGCAAGGGTTTTCACTATTGCATCTTTTTCTGTGGTATTATGAAAATCAAGTTTATTAATTAAGTCTTGAGATGTATCATGAAATTAAAGCACCCTTTGAAATTTTTGTTATTATTATGCTAGAAAGCATGTGTGTTTGTTTTTTTTAAAAAAGAGTCTTACTAGCCCTCTTACTCTTACCAGTAAGAGATGGGCAGACGTTTTGCTTGTTTGTTTTTTCTGTTAACTCAGACCCAGTTAGACATATAACATCTTACTCAGTAAGAGGTGGAGGACTTGTCTTACTGCCTCTTACTCGATAAGAGATTGAAGACTCGTGTTAATATATATAATATATTTGATGGTTTATTAACTATGTTATATTTATGATAAAAAGTCTTGTTTCATATTTACATAAGTTACTTAAATAAACTGCAGAAACGAGCACTTAAAATCACTTGAATAATTATGCAAAAACAAACAACATTATTTCGTTCAGATTTAGACTTACCAGCCATATCTTA

AabHLH39

ATGACATAATCAAGGACTTTTTTTAATTGTTAGCTTCTTTTTTAATATATATTGAGAATATTTTAACGTAAATTAATCTATCGAATGGCTAATCAGCTGTTGTAATTTAGTTTACAAGATTCCTAAATTCAAATACTATATCCCTAACTTATTGGGAAATGACACTTGTAAACATTAAAAAAAAGCTCTTAAATGATTGGATGTATTGCTTTTAGTATTTAGCACATGTTAACACAACAACCAATATTTATAACATGTGATTGGTCCTGATGTTATCTGATCAAGATTATCCTGAATAAATTAAACAACATAAATCAGGTTGCTTTATAGACACCAAAGAAACTATTCATTTGAAAATTTTCAGAAATATCTCTATCAATAAATCTTAATTTTTGTGTTACATTGAAATAAGTCAAAAATATAGTGATGGATATGTCACAGGCATGGTTGGCAGAAATGGTAATGAATGCTTACATGCATATTGTGGACTTTATATATATATATATGTTCAATCTTTGTTTGTTTAATTTTCTGGTTATTTCAGGAAATGGAAGATCCAGGGTTCATGAGTTATGATCAAATGAGCATACTTAGCGATGTTGTTAATAACTTCAGCGTTGACTCATTCTACTCAGAAATATATGCAGAAACGACTACGTGTGTTGATAAAACTTTTCAGACTCAGCAGCCCGAAATAAGACAAGAGACGAGTAGTAGTATTAGCAAAAGCTCTGCACCTCTGGATCCACTTGTAACCAATAGTCTTCCCTCATCCAAAACATTTACTATATCATTTGGAGATCTAGAACCGAAAGAGGAGACCCTCCAGTTTGATGACTCCCTCGGGTATGAAGATGCTCGTACCACAAAGGTGTCGATCACTCTCAGGAACCCGATACAGGCTCAGGATCATGTTTTTGCTGAGAGGAGGAGAAGAGAAAAGTTGAATCAAAACTTCATATCTTTGTCCACCGTCCTTCCTAGCCTTAAAAAGGTAAACTATTATACTCGCCAAAAACAGATTGTTTTGTACATGATGAAGAAAATTTTGATTAACTAAATCTAATATTATATCTATTAGATGGATAAGGCGTCTATGTTAGAAGATGCTTTCAATTACATAAAAGAACTTCAAGGTCGTGTGAAGGAACTCGAAGGAACATTAAAACCGGACAACAAAAGAGAGAATGTTGATCAAGAATCTGATATTTCCTTGAAGAGATATAAGCTTAGTAATCCGTGCGATAAAACAAGATCGGAAAAGAGTACTTCCCCTTGCAACACATCTCCTGAGATCAAAGTGTCGATCTCAGGAAGCAGTGTGACAGTTACAATTCAGTGCCAGAATAACTCTTCTTCCTTTGTTAAAGCACTAACTCAGATGCAGAAGCTTGGACTATCTATCATCTACAGCAGTGCCATGCCTTTTGTTACGACCATTCTTCTTATCACCATTGTTGCTCAGGTACATGTATTATACACCCGAAACTTAAGTTTTACTAAAATGTGAAGTGCGTAATTAGCTTTAACACTTTGGTTCCTTTTTCATTATTTGTGCAGATTGTGGATGACTTCTCAATGACACCAACAGAACTTGCAAAGAGCCTTCAACTAGCTATATGAATTGAACGTAGTTAGGTAATTTGTGGTTCCTGTTTCTGTGTACTAGTTAAAGATCGTTAATTCTTCCATTTTCATGCCATTTTATAGTACAAACAAGTTAACTTGGAAACTTGAGAGAACTCTTATTCCAACTCGGGATTTTTCGGTGTACCCCTGAAGAAGAAAAGCACCCTTTAGCTTGATTCGTACAATTTTTTTTGAAATTATATCTTGCTACTCTTGGTGAAAATATAAAAGATGGAGTTTTTGATGAGTTTTTCTTAATAGGTTATTGTACGGTATTTTTGTGTGGTTAACCACATTCATTAATTTACCCATGTTATACATGTATGATCCATCATCTTTTAAATTTAATGAAGATTTGCACAAGCTTTCTTATGGTGATCCTTTTGAATTTAGTTGTTAAAGTCCAGATAAATTTATTATCTGTATCAACTGGGTATATGAATTCAACTGAAAGTGTGTTGTAGTTTTGTTAACTATAATAAGGGCTAATATGTTCAATGAACAGTGATTTGTGAGAGCCCCCTTAATAGATCCCAGTTTTTATGAAAAAGAGCTGTAAAACTTAGTAATTCTGCGTCAGTTCTGGAAGTATTGAGTATTGAGCCATGTTTTTAATGATGAACG

AabHLH40

ATATCTTAACTATATCTTATTACGAGTAAAAAATTATCATTAACACCAAATAAAACCTCATCAAGCTGACTCAAAGTTCTACGAATGTGTTTTTTTAGAATAATTTTAAAACAAACAAATTATACATAGTGATTAACAAAAAAAATCTTGTACACAATTTTTATTTATTTTACATTGAAGAAAAATGTACACGAGACACGCAATTATAGGTACACAAGACACATACTCCCTAGTGTAACCCAACAATTTGTATTTATTTTGCAATAAAAATAAAAATAAAAATAAAATAAAAAAAATCAATTGTTCTCTTGATCCTATAAAAAATAAAATCCTATAATTTGATCCTTTTACGCACTTCAAATTATTCTCTAATCTTAATCAACATCACATCCAGTACTATAATACTCCATTGTTTACAGGTCAATCTAGTCTAAGTAAAAGCTAAAGGAAGCAATGAAAAATGAAAAAAGTAAACTGAAATGAATAGTAAGCATAGGGTTACCCTATAACACTACTCTTCTTTCCCTCCCTCCCTCAGATAATATAAATAGATATATGTGAACAAACACCAAAGCCTAAAGTTTCCACACATTAGATAACAAAAACAAAGAGTATGGCTTTAGAAACACTTTCTTCCAATGAACTCCTCAACTTCATAATCTATGACACCATTTCTGCATCCCCTTTCACCTTCAATGACTCATCTTCTCAAAACACCAACAACTCAAATACCTTCTTCTACAATCTCCATAATCAAAACCCTAACCCTAACCCTAATCCTCTATCTCAAGAACTTGAAGGTGCTGACATGGAAATTATGAGTTCTAATTCTTCTTTAGCTACAACCACAGAAAAGATGTCTCTGGCTGTTCAAGCTTATTGTGGAGGCAAAAACCCTGAAAAGAATTATTGTGATAACAACAACAACAATAAAAGTATTCATAATAATTTAGGGGTTCAAAAGAAGAAAAGAAGAAGGAGGCCTAGGGTTTGTAAGAACAAAGAAGAAGCTGAATCTCAAAGAATGACTCACATTGCTGTCGAAAGAAATCGTAGGAAACAGATGAATGAACATCTTGCCGTCTTACGGTCCCTCATGCCGGAATCTTACGTCCAAAGGGTAATTTTTATATTGTTTTTTTCATTACTTTATTATAATGTAGATCCGAAAATTTTATTTAATAGTATAGGTTCTTTATTTTATTTCTTCCAAGATAAGATGTCCAAATAAACAAGTAATTAATTAAATGAAAAAACATATTTAACATGTATTTGGAATTGAATTGAATTACTTTAAATTTCACTCTTTCATTATCTTCAAAAAGAAATCTTGAAAAGTTCTTTTTCAAATTACCTTTTTGGTGTTATGCAAATTTCAAAATGTTGTTACCTAGAAAATGCAATGCCATCCATAGATGTTCCTTTATTTTTATGAAATTTTACATTTTTTGTTAATAATATTGGCCAAACATTAGCTTACTATATAGCTTGTGATTGAGTTAGATTATGTATATGAACTAAATCTCAAACCGACAATTACTATAGGACTTGGTTAATTAGTATCATTAGATAAGCCAAACGTACGAATTTAGACATCTCTCTCTCATGGTACAAAATCTTAAAACACTGTGATTAACAGTGCTTGATCTCAATAAAAGCATTATCTTCTTTTAAGAGATCCCTTTTCGTGTCAATTTGTCGGATTCAATGAACAACAATAACATGCCAAAGTTTTGCTGTTTCATATGGTTGACTTAATAATTATTTAGACGTACATTAGTACTAAGAACAAAAATTTTCTTACTAAATGACGGTTATGCCCCTTGTGTTCATTCAATCATCAGGGTGACCAAGCTTCCATAGTTGGTGGTGCTATAGAATTTGTCAAGGAGCTTGAACATCTTTTGCAATCTCTTGAAGCTAAAAAATTTGTCATGACTCAACAACCACAAGAAGATGACGATAACGGAGGCCATGATAGTAATTTCACCAAATTATCATCAGCTGCCCCTCCATTTTCACAGTTTTTTTCATACCCGCAATACACTTGCTCTCAAATTCCCAACAAGTACACTTCTAAAAGCAAGGCAGCTATTGCGGACATTGAAGTTACTTTGATCGAAACGCATGCTAATCTTCGGATTTTATCGCAAAAACGGCTTGCTCAGCTGTCTAAAATGGTGGCTTGTTTCCAAACACTTTATTTATCAGTGCTACACCTCAATGTCACCACAATGGAACCTTTGGTTCTGTACTCCATAAGTGTTAAGGTACGTAAACGAAATCTTATATTGTACTGTAATATACAACAACCCACATTTTAACATCTCATAAATGTATCCATTGTTTTTAATATTTGCATAATTAGACACCCCAAAAAAAAAAAAAAAAGAGAGAGAGGGAGTACTGTTCATGTATGTATCGCTCATGCAAAAGTGTGTAGATGAAAATGACTTTGCATTGAATGACTCGACGATGCAAAAACCAGCTTTTGAGATACGACTAGTACTTTCCTTATTTATCATTAGACACAAATTCCTTTTTCTTGGAAAATAAAACTTTCATCATAGTCAAGGCAAAAGAAATACTCAAAAAGGGTCCATCAGTTTTACTTTATCAACTTTTTGCAAAATTCAAAGTTCAAAACTGTGAATTGAAGTGTTTGGGAAGTTACTTTAAATTTGGAATAACCAAGTCCAAACCAACTACTAAAATCAAGTTTTTTAATTTAAAACTTACAAATTTTGGATAAATAAGCACAAATTAAAAAGTCTTTAATATACTTAAACTTTTTGTATCAAAATGGATTAATTTTTGTAACATTTTAAATAAAAACTTGAAAACTCTATAAAAAGTTTGTCTAGGGTATTTTTGGGAATTCCACCAGGTATTACCCAATTTGTTAATGTTTTGGAAATGATGGCTAGAAAAGGATGTTTGTTTCCATAAACCGATGTTGTTTCCTATTTTTGTTACTTTGTCTTAAAGTTAAGGAAATATTCAACATAATATATTAAAGTTCATTGAAAAATGATGAAAATATGACTATATGTTAATGTAAAAAAAGATATGTAAACATGCAGGTGGAAGAAGGATGCAGACTCAATTCAGCAGACGAGATAGCAGGTGCAGTCCACCAGATGCTCAGAATTATTGAGGAGGAAGCTACCCTATGTGTTGTTGATTCTAGATAGAAAAAGAGATGTTTGTGCTTTTACCATTTTACCTTTTAGGCGTTGTAAAATCACCATAATGCCACCCATTCTCTCTCTAGCTTTGTTCTTGGTGCACAGTAGTAGGATTTGCTATTACTTTGAATTTCAACTAGTTCAATTAGATGTTTTGGTAAATAATGATTTGACTTATTGAAGTTATTTATTGGTGAAGAGTGATTGCATTTTTTTTTCTCTAAAGAATGTTGACATTTGTAAATTAGATATTTAGTTTTGAACCAAATAAGTAAAATGATCATTAGACCATTTCAAATGGTGTAGACTATATAAGTTTAAATCATTTACACATCAGAATTTTATAAACTTGTTTACTTGACTATATAAACCTAGCAATGGTCTCATTCACTCGTCTTTTAAGTATATTTAAGTCTTAAAATGTAAATCAGACCCCCATTTAATTTCTTCTCTTTCTCTAGTACATACGTTTAAACCTTGCTCATAAACATTGTCATGCAATGCTAAGTATACACTATAAAGTCCACGTACGATGACAGCTCAACATAAAACTCTTGTGTAAATCTCTATTGAAAATGCTCTTAGTCGCTCATCTCAAGTTAAGTGAATTTTCAGTTTGTAAACAATCAAAGCGTTTTAGAAAGTTTTAAAT

AabHLH41

TTTTTTTTTTTTTTTTTTTTTTTTTTTTTTTTTTTTTTTTTTTTTTGGTCGTAGGGGAGGGATTGACCTTTCTAATATTTAATGATCTTTGATACCCATGCACAAATTGTAGTTGTTTGATCTTCCTTGACGGAGTATGCAAAGAAGTAAACAATCAAGCAAGCACATCATCTGGGAGACTTTTCGTGAGGTCATTTCTTGACTACAAAAAGTCGATGTTCTTGATTGATAATTATAGTGGGTAAGAACAATAAAAACACAATCTTTTTTCTTATAATGAGTATTACTAAAACTCAATTGTATGCGAGGAGTTTTAACTGAATAATGTTTTGTTATTAGAGGAGTTCCAGGGTTTGCATTCACGCACAATATTACATACATGGAGCGGAATGAGTTGGAGCTCTTAAATTTGGCATCAAATTCAGCACAGCTAGAATTTTATAAAGTAATCACACTTCAGATATATATTAATTATAAGCTTAGTTTAATTCATGTAAGAGTTGAAGCTAATGTGTATCCCTATTTTATCTTACAGGAAGCAGGGATTAAGGTATCCGATATATGTAGTCATTTAGCTCACGATTCAGTCCATTAAGCTCGCTTTCATTCACTTTTATCATTTCTTTTTTAGCTAAATTTCGTTAAGGATCTTGCGAAATAGCTAGGCTAAAAAATAAATTGATCAAGTCGTAGTACTTCGTATTTGATTGATTGATCACACATACAATGATGATGATAAACTTCCATTACAGACTGCAATATTTATGGGGAGCAGCAATGGAGAGATTGAGCTTGGCATAACCTATGACTCCTCTCAAGTAAGAAATGTAATTCTAATCTTTCACATTGTGATCCTTAATTTGTTCTTCGGTGTTTATGTTTATTATAGATTATATCAGTATTACATAATTCGTTTAAATGGGTTCGGTTTTTTTTTTNCGAGTTGAAGAAGTTGTTTCCTGGTGATTTCCCGCAAGGAGTGCTTCCTCAACGACTTGAGCAAGCTCGGGCTTCATCATTTTTAAGATCATTATCAATGGAAAATAGCGTGGAGAACTCGCCATTTCTCTTCAACATGCTTCATTCCACTCCTTATATGTCCGAAATGTTTGCTCTAACAGAACCATTAATGGACCAACAAGCACCAAATCAAACACCACAGTCAACAACAATATTGAGATCAGAAGATCCTCTGCAGCAAGCTTTAGAACAAATTAGAATCTCTCGGTCATTACCTTCAAGAGAACACGAAGAAGCTACAATGACAAGAGCAATTCTTGCAGCCATTACTTCTACTTCTTCTCCTCCTCCTTCTTTATTTCCATACCAGCGCCAACCCCCTCAAGTTGCTAGTGCTTTTAAGAGATACGGATCAAGTTTAGGACCTAATAAACGAGTTCAAAATCGACAAAATCTTCACCGAAGGTCACTTTCATTCTTCCGAAATCTAAGTGAGGCTCGTGCTCAAAGAGATCAAATGGTCCAAACAACTAGACCTACTAGCAATCAATTTCTTCATGTGATAGCGGAGCGAAAGCGAAGAGGAAAACTCAATGAAAGCTTTCAAACTTTGAGATCACTGCTTCCTCCAGGGTTCAAGGTATATCTATTTATTTATTTATTTTTTTTTTCTGTGATGCTCATCTTTAAAATTTGAACGTCAAGTAAACAATCCGCCTAGCCTACATTTCATATCATACACTAGTAGTAATAAAAATGAAACCCGAATTTACTAATTATAACACTATTTTTCATTTTTATGAGAAAGTGAAATCTTCATTATATTCTGCTTAAATTTAGGTTTAGTTTTGTGCCTTAACTTACGTGTTAGTTTTTCATTTCTTGTAACAAATACACTCACTTCTTCAAAAATAATTTTAAGTTTAGGTTTCTTAATCATCTCTCTCTCTCTCTTTTTTTTTTTTTTTTTTTTTTTTTTTTTTTTTTTTTTTAATGGCTTATGATCACTTTAGAAGGATAAGGCATCAGTGTTACTAAACACAATGAAGTACATATCTTCTTTAAAGTCTCAAGTCAAGGAGCTTAACAAAAGAAATCAGATTTTGGAGGCTGATCGGCGTGCCGGGAAAGAACCTCCTAATCAAGGTTCCAGTCGTTTTTCTGGAGAGGGGCCTGTAGTTGGCATCACCGACATTGGTGAATCAACTTCAGAATCACGAGTGGTTGATTTGGATGTGAATGCAAGAGGGAATGTGGTACTGGTGGATTTGGTGATGAGCGTGTTGGAGTTCATTAAACAGACCGAGAATATAAGTGTCATGTCTATAGATGCTGGGATTCGAATGTTGGATACAGAACCTATCGCAAATCGAGTTATTTTGAGATTAAGAATTCAGGTATATATACATTCATGTCTATCATTATTCTGTTTTGATGCATTTTAATGACCTAATTTGGATCTTACAGATTCATGCATATATTAAAAAAATAAATAAATAAAAAATAAATAAAAGTCATATTCTATTATTTCAACTTTATTAAACATCATCTAAGAAGCTTCTCATGATTCACGATAAACTAAAGATTAGAACAATGACATGAAAATCAAGTTTCTAGGTTATACTAATAATCCAATATTTGTGGTTTCCGGGAACATGTTCCTCAACTTATTTGACCTACCATAGCATAAAGCTATTTTTCAATTTCAGCTTAAAGAATAAGTTTAATGGGTAGATAACTAGATATGGTTTTCTCCTATAATAAGTTTATCATATTTTTAGATAAGAGGTGTTTAAAAAAATAAATAACTTGAGTTTATCAATATATAAGTAGTATATAGATTTCTAAATGTTTGTTGATAAATGGTCAACAAAACAAAATGTTATTCTATAAAGGACAATAAAAGAAGTATCTATTAATTAAGTCTCTACTTGCCTTTTATGTAGGGAAATGAATGGGACAAATCAAGCTTCGAAGAAGCAGTGAGGAGGCTTCTTGATGACCTGACACAATGACATGTTGTCATATTTTTTTTTTTTTTTTTTTTTTTTTTTTTTTTACACTTGTGATCATAGTTATTATTTTGAACAACTTTAGAAAATCTATCAAGAATATTTACTTCTATATGCATATTAGCATTATTTACTTACTTGTAGAACAAGTGAAATATGCATATAGAGTATATATGGCTGATTTACCTTTTTTGCTGTTTGCAATATACGAGGTAACGGACTAATTCGTGTAATTTAGGCTTTGTTTGGTTAAATGAATGTAACGAGGAAGGAATGAATTGAACAATGTACTGAATAAGTCATTACTATTCTATTGTCTTGTTTAGTTGTTATGTCGATGTAATGAAATGAATATTTTCACAGTTTGATGTGTTGTAATGAACTGGAAATGAATGAAATAAATGGAATATGAATATCATTCCAGGAATGTAATGAAATAAAAACAAGGGTAGCCATAGAATGAAAATGATGGTTACAGTCGTAAAGAAGTGAATAAAGAAATAAGCGATTTTATTTCAAAACTTAAAAACAAATACCAAATTTTAATTCCATTACAATGACTTATTCCTTGCGACATAGATTACTACGTACACATTACGCAGCCATTATCCG

AabHLH42

CACTACTATTATTTATTCCGACAACTCATAGAAGGTTAAACATTGTAGCCAAAAGATAAATTTTTTGATCTTTTAACTACTTGTGGATCAACATTTACCAAGGTCAATGCCATTTGTTAGTTTTTGATGCGAATGTCACAGTCATTTGTATCCCAGACTGTCAGTAAAGTAACTACAAAATACATTCTTTTGATTGTTCTGGAGGCTAGCTTGTTAGATGCCTATGTAGTTCTAGCTGGCTCATATTCTGAGAAAGTATTTCATATAGAAAGCAAAGTAGCTAACTGAAGAGTTATTATGTCAATAGACTCTGAATCGCTGATTACAGAAGTTCCGTTGTTGTAGGTGACTAACTTTTGATAAATCAAGCAAACCTACATAAAATTGTTATGGATATGCCATCCGGCTTATGGTTGCCTGAGCTGGTAAGAATCGTAAGATCTTCATTACAAATCAAAATGACTGTTTAGTTTTTGAACTAAGATTGATGTAAGCCTAACTAATATAGGAACTGGAAGATCCTCGTTACTTTATGAATCAGCATCAGCATACATGCACGTATAGTGAGCTAGTAGATTCATTTTCTTCGCAAGGATTCAAAGGTTACATAGAATCTAATCGTGCAACCACGGTTAGCGAAAATTTGGACAAACAAGAAAGTTACAAGGCAAGTAGAACTAACCCTTTCACTCCTATTGGCGGATCCACAGCTAATACATTCACTATATCCTTTGGAGACCACAACGAGATAAACAAAAATTCATTGCATGGAGGATTTCAGCTGAAATACAATGATGCAATAAAACCTAAAGTGGAGATGAGTCTCAATGAACTTCTTGACTCCATAGAGATTCCTAAAAGAGTACCCAGCACCAGAAGAAATCATAGACAAGCTCAAGAGCATGTCTTGGCTGAGAGGAAGAGAAGGGAGAAGCTGACTCGACGCTTCATTTCCTTGTCAACTCTCCTTCCTGAAATAAAAAAGGTACCGATTAAAAGATCATCACACTTTCTTCTTCAACTTATCATTTTTTTTATGTAAAAAAAGGGCAATGCAATTCAAGCTATAATATTTACATTCTCACACAACCACAACAATGGCATTTTCGCCCCGCTGGGACTAGAATCCACAATCTCTAGGTTAAGGCTCACGGATACCGCTAAGCCAGAAGCCTTTTGGTTCAAAATATCTTGTTTTATGCATATATACCTTAATGTATATTATCCTAACCATACCATTAGAAATTAACACCTATGTTTAATACATCTCATATTAATGCTGAGTTTAGGTAAACACATACTCATATTAAACTTGTAGAATGCATTATCATTCGGTTTGATATGATTTGAATCCCGTATATGTCTATTATATCTCAAAACTGTACCATGGGAAACGACCTTGTGCCACGGCCCACGGGACTGAAAAAATAAACTTTGACCCAGTACGTGAATGTGTACGAAATAATTTACAACTTTACTACTTCAGAACAAACTTTGATGTATTGAATAGGAGCAGTAAAGTTTTCATGCATGTCATGCGAAAAGCGGATCTACAGTTTGATTGATTGCTTTCCCCGTTTGATACTCCCAAATATGTCACACATCTATTTGTTGTGCTCTAGAGCGTGGTTGGTTTTTTCATGTAACTAGTTATAGGTATCGTTTTAAGTTCTAAAATACGTGTATGCCCAATATTATGAGTCTCAACGTACTTATATTCAATAACTAGTTCTTTTTGTTTTACCTTTAAAACCAATCATCTAAAACTGATAATCTAAAATGATCCCTTATCGTAGATGGACAAGGCTACGGTGCTGGAAGATGCAAGTAAGTATATTAAACACCTTCAAAACCGAGTGAAGGAACTTGAACAAACATCAGTTGGTGAAAAACACATCATCCTGGAATCAATAACTTCAACGAGGAGCAATTTTCGCAGTAGTCATGAAGACAATGCATCTTCTTATGATAAGATTAACTCATTACCTTTTAGCACCGATAATGATCCTGGGATTAAAGTAAGAATTTCAGGTAGCCACACACTTGTAAGAATTTACTGTCAACGAAATTCTTCGCTTGCATTAAAAGCCCTCATCGAAATGGAGAGACTTCAATTTATCATCATGTGTAACAACGTTCTTCCCATCTCTGGAAATGCTGCTCTAATAACCATAATTGCTCAGGTATGTTTTTATGGAACACATAGCTTATATAATAAACAAACTTCCTGTCGATGTGTTATATACACTACCAAAAATGTTTTAGTGACTGAAATAGCGACCAATAGTACCGGGTCAAAAATCCTAAAATTTAGTGCACTGTAGCGTGGGTACTATATATTTTTTTTAAATTAATTTGCCCACATATATTTATAATTAGTCGGTAAAACGTATGAATTTACTGTTTTTAGTCATTTTTATAGAGATTATCTTCTCCAAAAGTAAAAACGCTTTGTGAAACTCTAAAATGCCGGTGTGTCACCATAAGGTGCTGCATCAAACACTTGGTACAACCCTCAATTTTATCAGAACTAAACAGCTTGAGAAATTATTTTTGTGACTTGATGGACATTTTTGTTGTGTCACTAAGATATGCTTTATTTTCAGTAATATTTCACTAAAAGATCCTTTTTCATTATTTTGTGTAGATGAGTGAAGAGTTCGAAATGACAGCGACGGATCTTGTAAACTGCTTGAAATCGTCTCTTTAAACTTTCTACGACATGTACCTTACCTTTAAATGAATGAAAAGTTGGTCATGACCGCTATGGATCTTGTAAATTTTCAAAAAGTGAGTCTGTTTGTTTGGCATCCCCTGTTTCTGCCTAGCGATTTCTGGATGTGATTTAGGTGTATTTACTTGGACTAAGAATGTTGTTTAGATTGATCAGTTTGGCTTTAGCCTTTTCATTTTCGGTGTAGCTTATTCCTTTGTAACCTACTAGCTTCTTGCTAGCTTCTAATAAATCACAGTAGTTTTTCAAGATTGTACCTGAGATGTAGATTTGATGGTATGTTAAAACGTACAACTAGCTATTTTTCTCTTTATTTTGTGAATATAACATGGTTTCATTTTTAGGTACGAGATCTAAATTCTCCAACATTGGGATAGAGACCTTTACATACAACAATTTCCAAAAGCACATGGCAATATCCTTGTATCCTCATATGATAGGGGTTTAATGAACTGTATGTTAACATAATCCCGATTATGTATAATATAATCCTAGAAGAGAGAACTTAATTAGTGTGATAAGAAGTGAGGTCTGTCAGTATTTTGTCAAATATGAGTGTGCGAGAATCGATGGAAATAGAAGCAAGTGGTGACAAAAAACAAGGTTCAAGAATTGTGCATATCTGATAATTGTGCATTCTCATGAAGATTTATAAGATTTTATCCACAACAAGATTTCTAACACGTGCTAAAACCAACTTCTATTAAGCCCAATCTTTTGATTCATTGCCCATTTTCATGTTAAGAGAGGAAGGTGTGGTGAGGAGGTAAAACTCCTCATTCGGGCCCCACGGGGTGCCACATCATCTTAACAAACCTC

AabHLH43

ATATACTCTTTCATATCGTCCTAGTTCTAATTTAAGGTTTACTTTTATGATTATCACATCAAAACACGAGCTTTATCAATCTTTATTATATACCTAATTCTGGTATTTCTATGATTTGATTTCAAACTTCAATAACCTAGAACTAGTACTTAAGAATGAAACATTTTTTTTTTATATCTGAACAGTTTTCATTGTCGGAAAGATTTTGTAACAAACAAGATAGTGTCAAATTTGATAAAGTGCAGCATCTAGCCATCTTCTGTGAACTAAAATTTTATCTATAATTGCAGCCTGAATTGAAATTAACATTCACATATCAATACCATGGAACCATCGTCATGGCTACCGGAACTAGTAAGTCTCATTTAATTGTAACATGTAAGTTTGGTATTAATTTCTTATGTACTCCTGGTTTAATTTTTTTTGTTTGTTTGCTGAATCCGTATTCTAGGAAATGCAGGATCAAGGGTTCATAAATCAGTATCAGATGAACAAAGCTTATCATCCTCTAATGGACGACTTCAGTGTCGATTCATTTTCATCAGAAAGCTACACAGAAAACCCATCTTTTATTGATCAATCTTTTCAAACTCGTAAGGGAGTTGAAGAACAAGCTGATATTAAGCAGCCGACTAGTTACAAAAGGGCCAATAGTATTAACAAGAAATTTATTCCTATCGATCAAAAACCGAAACCAAAGCTTGTTTCTGATCCTCCCAACGCTTTCACTATATCTTTTGGAGATATAAAACCTAAAGACGAAATACTTTCTTTTGGTGATTCATATGGCTTCACAGGAGCTGGTAGCAAAAAGGTACCCGCAATGATTAGGAATCCGATTCAAGTTCAAGATCACGTGCTGGCCGAGAGGAAGAGAAGAGAAAAGTTGGCTCAGCGGTTTATTTCTTTGTCTTCCCTCCTTCCTGACCTGAAGAAGGTATTTCATAGCATGTACCCTAGAAACTGTTAAATTATCTGTTTATTAGTTTCGAAGGAGTTTTTGGGATAAGCATCTCCAGACACCTTTATTCAAAATCAAAAAGCTTATTTCACTCACAAACAGATAAATATAGTTTGGATAACAATGTCAGACAAGTTCAAAACCGCGTACATGTAGGAGATAATTAAACTATTTTAACAAGCTAGCAATCCCAAATATATGCAAAAAATATCGAATGCAATATAACTATTAATGATCGAGCTAACTCTGTCATGATAGATGGATAAGGCAACTGTATTAGAAGATGCAGCTAATTACATTCAAGAACTTCAAGGTCGAGTGAAGGAACTGGAGGGATTATCAGGCTTGAAGCGAAACAACATGCAAGAATCAGTTATATCTGCTAAGAGATCAAGGCTTAGTTGCAGTGATAATGATGGTTCTTCTAGTAATGAAACCAACTTTGAAGAGAGTAGTAGTCCGTCCACTCCCGAAATTGAAATTAGGACATCGGGATGCAGTCTACTAATCGAAATATATAGCCGAAAAAACTGCATATCACTAGTGAAAGTGCTAAGCGAGATGCAGACGCTTGGTTTATCTGTTATCAGTAGCAGCACCATGCCGTTTGCTGATACCTCTCTTCTTATCACCGTTGTTGCTCAGGTATATTCATAGACCCATAAATTAATAATCGCTTAAAATGGAAAGTCATTCCTAATTTGTTCCTTGAAAAAATGTACGAGTAACAAATATAAAGGCCATTTTATTTTGCAAATTCATTAGATCTATTCCGGTAAAAGTTGGATACATACACAAACATCGTTGATCTTAATCATGCTTTCTTGATTTGCGCAGAAGAGTGATGATTTTATTATGTCGTCAACAGATCTTGTGAAGCACCTACAACTAGCTATTTGAACTTTTCTCCGACGAGAACTTGTGCCAGACTACGTACACTTACAGGCTGATTGATACAATCTTTGATTTCAGTAGTGGTGTTTAACAGTTTGGGGTTTCAGGTTTTGACTCGAAAAACCCAAATCATATAATTTCAAAATGTTTTTTCATTTAATTCTATATATTTTTAGCACGATGTATTAACTTATAAAGCTTAAACAATGTCTACTACTCAAATATATAATGAAGAAATACATAAAAAAAAAAAAAAAGATTTATAGACTTATAAATATTCTATTTATAGTAGGAAAAAATCTTAATGGTTTCTTCTTATGTATACAAATATAAAAAAAATACATATATGCATATGAAATCAGTTTACTGTTCGGTTTATGTTTGGCCAAACCCAAGAATCGGATACTTAATTTGGTTCATTTTGGTTTTGAACCGAAAACCAGTTTATAAATT

AabHLH44

GGTAATTCCGTTACAAATTTTGGCTGAAAGTAGTATCGTCAGATTATGTTAAGTCATTTGGGATGAGGATCATAACTTCTTTTGATGTAATACGGAAGCTAAAATCACAACAAGAAGAACTACACTTTCTTAATAACCCGAAAAAAAAAAGAGCCATTTTATTTCCTTCTCTACGGAAGGTTTCATGTTCGATCAAAAGGGGATCCAATAAGCTGTATGTTTTTTTGAAGAAAGTGAATGTGTGTTTCATATACAGTACTTTACATCCATTTAAGAGTTGGTAAAAATGGGTATTGGGACTTTTAAAAGAGGTATTTACAATGAAAATAATGTCTTGCATTATACTTCATTTTTTTTTCCCCAAAAAGTGTATAAAAAGTACATTTCTATAAACCATCCAAATATTTCTAGGACACTTATTCCCCAAGCAAACAATTCATCTCATTTCATATCAAAAACTTTATAAATACAACAGATTGTCACAAATTTTCACCTTCAATTCACTTCCACATTCCTACTCCCAACCTACCTTCTTTCTCTCTCAAATTCCTTCATTATTCATTTCATCTTCATAACCAAAACATTTTAACAAAAAAACACAAAATGCTTGATAATTCAGACATGTTTGAACCCAACACATACAACCTTGATCCAAACACATTCTCCCATCTCATTGACCATAACCATAACCTTAATAACCAAGAACAACAACTCAACTGGACCAACAACCTTAATAATCTCGACGTCCAAACGAACCAACCCGACTTGCTAAACCTTTTTCAGCTGCCAAAATGTTCATCTTCTATATGTTTTTCCAACCCAAGTCACATGGACCAACAAGTAATGTACCATGATCATCATAGTACATTACTAGGAATGCATAATAGTAATAGTACTAATGTAGGTGTTACACAAAATGTTCCGTATATTCGTGAGCTTTTGCATAACGGGTTTAGCCTAAATGGATGTAACTCGTCGATTTTTGGCGAAATGGATATGGAACATAATGGTGGTTGTGATGGTATTTTGGAATTTGGTAAAAATGGTAATGGTAATAAAGATAATGTGAAGCATTTTGCAACTGAAAAACATAGGAGGCAACAATTGAATGGAAAGTTTGATGCTTTGAAGAGTTTGGTGCCTAATCCTAGTAAGGTATGTATTATGACTTTTTTTTTTTTTTTTTTTTTTTTTTTTTTTATTATATATATATAATGAGCTTTTTAATGTTTATTGGGTACGCGTGAAATGCCATCCGAGACATCTTTGGTGATCGACCTGGAAAGTAAACCCCGGACTCCACCACACGCGAGACCCTTTTTATATTTTTTCTGGGTGCAAGTGAAATAGCCATCTTTGGTAACCTGACATGTAAACTTAGATTCAACATATTAAACACACAATACCCTTTTAACGTTTTGATTATATTTGATTGTTTTGATGTTTGGTTATTTGTGGGTATGGTTGATTGTGAATACGACTAAAACGTGTTAGAATTATGTTTGGGTTAATTGGTTTAAAACTTAAACTAATGTGTTTTATGTGTCTTAAGTAGCAACATATTTTCAGATGTTATTAATCATGTGCCAATTATAAGCTAAGTTTAATGTTACCATTATTGGTAACAATTGACAGATTGTTGTTGTAGTGACTGGTAAAACATGTATGTGGTATGTTCAACTAAATATTACAACAATATTGGTAAAAAAAAAATGGAAGTATTTTATTGTTATGTTTGGTAGAACATGTGTTATGGTATGCTGCTATTTTTTGCTGTTATGAATTTGATAACATGTGAAACTTGTTTTTTGCGGTCTAACTTGTGTGTTTGTGTGATTATGAACAAAGGGTATGATAATTTTGTTTTACTACGAGTATATTGATGGCTTTGATAAACTGAACATTTTGATTCATTTAAGGCTTGGTTTTTTATAACATCAGATCATTTGATTCATTATAAACTTGTTTTCTTGCTGCAATAAAGCCTGATCGCGCATCAGTGGTTGGCGACGCGATTCAATACATACATGAGCTCAAAGGGACTGTGGAAGAACTCAAGCATTTGGTGGACAGGAAAAGATGCAACAGAGGCAGGATGAAAAAACACAAAACTGAGGACGACTCAACTCTAGATGTCGAAAGTATTTACACAATGAGCAATGGCGTAGTTGGTGATACTACTCATGATCAGCAGGCCTATAACGGCAACTCATCTTCATCAATGAGGAGTTCAATGGTACAAAGGAAATCAAAGCACACTGAAATCGATGTCCGTATCATTGACGATGAAGTTACGATCAAACTAGTGCAACAAAAAAAGATCAACTGTTTGTTACTTGTTTCAAAAGTCCTTGATGAGCTTCAGTTGGACTTTCATCATGTTGCTGGTGGACTTATTGGTGATTTCTATAGCTACTTGTTCAATACCAAGGTACTAACTTTTTAGACCATTTTGTGCATTAAGGACTTGTACAGTACAACCCATTGGTGTAACTTCTTGTGGGTGTTAACCGGGTAGCCTAGTGTCAAAAGACTTGTGACTATTATCATAAAATATGATAGGTCTCAAGTTCAATTCGTGTGAGGTGCAATACCGTGAATTGGCCACGCCAACCTCTTGAAAAGGGTTTGGGTCTGTGTAGCGATGGGTTCAAACCCGTGAATTAGTCCGGGCCAATATTTGAAAAAAAAAAATTGTTAAGTTATGGAAAGTTTTGATGATATTGAATGACATATGCAGATATGTGAAGGGTCCTCTGTTTATGCAACTGCTATAGCCAATAAGCTAATTGAGGTGGTGGACAAACAATATGCAGCAATGCCAGTCACTTCTAGCTACTAGGTAAGAAACTTATTAGGTACAAATTCCTATGCGTATGTTACACGTTCTTCTGCATAATGTACAGCTCCTTTCTGCATAAAGTACAGACCATCTAATAGAAAAAGACACTTTTTTTGCTGGTGTTTCCACATTTGGGCATGCATATCTGCCTTTTTATTTCTGTGAGCTTTATTGTTTCATATGTTTTGACATGTTGAAAATCAGAACATGTTTGTATATGAACAATTTGTTTACTGTTTAGACATAAAATAAAGACTCCATTTATAACAAGAAAATGTTTTACAAAATGCATTTTTTTCATTGTATTGAAGTTTTAGTTCAAGTTGAAGTCTGTCTACTGACAACCTTCACAATATCATGTTAGTACTAAATTCTAGTTAAGCAAGTGTTACATTCTGTTTTATGCATTAAAAAATTATGACTTAAATGTTTTTGTTAATTTAAATTTTCAGGCTATGAGTACTTATATGGGCTTAGATACTAACAAGTCTTGAAGCTATTGGGAAGACAGGAAGAGACTAATTACTGAGTTAACTATAAATAAATGCAGGGATATTTTGAGTATTTAGCAATTATATATGAATGTGACTACTCAGAATGGCATATAATTTATGA

AabHLH45

ATTGATCCTATAAAAACCAAGGGTAAGGCTCTTCTCATGTTCATTTTGAACATGAGAGGTAATGTTTTGTTTATTTTGATTGTACATTTGAACTAAAATAAAGCAAAAGTGTTGCAATAATTAAAATGCACAATCAGAACAAAAACAACATTACCTCTCATGTTTTAAATGAACATGAGAGGATCGTTATCCAAAAACACAATCAAATCCTATAATTTGATCCTTTTACGCACTTCAAATTATTCTCTAATCTTAATCAACATCACATCCAGTACTATAATACTCCATTGTTTACAGGTCAATCTAGTCTAAGTAAAAAGCTAAAGGAAGCAATGAAAAATGAAAAAAGTAAACTGAAATGAATAGTAAGCATAGGGTTACCCTATAACACTACTCTTCTTTCCCTCCCTCCCTCAGATAATATAAATAGATATATGTGAACAAACACCAAAGCCTAAAGTTTCCACACATTAGATAACAAAAACAAAGAGTATGGCTTTAGAAACACTTTCTTCCAATGAACTCCTCAACTTCATAATCTATGACACCATTTCTGCAACCCCTTTCACCTTCAATGACTCATCTTCTCAAAACACCAACAATTCAAATACCTTCTTCTACAATCTCCATAATCAAAACCCTAACCCTAACCCTAACCCTAATCCACTATCTCAAGAACTTGAAGGTGCTGACATGGAAATTATGAGTTCTAATTCTTCTTTAGCTACAACCACAGAAAAGATGTCTATGGCTGTTCAAGCTTATTGTGGAGGCAAAAACCCTGAAAAGAATTATTGTGATAACAACAACAACAATAAAAGTATTCATAATAATTTAGGGGTTCAAAAGAAGAAAAGAAGAAGGAGGCCTAGGGTTTGTAAGAACAAAGAAGAAGCTGAATCTCAAAGAATGACTCACATTGCTGTCGAAAGAAATCGTAGGAAACAGATGAATGAACATCTTGCCGTCTTACGATCCCTCATGCCGGAATCTTACGTCCAACGGGTAATTTTTATATTATTTTTTTCATTACTTTATTATAATGTAGATCCGAAAATTTTATTTAATAGTATAGGTTCTTTATTTTATTTTTTCCAAGATAAGATGTCCAAATAAACAAGTAATTAATTAAATGAAAAAACATATTTAACATGTATTTGGAATTGAATTTAATTACTTTAAATTTCACTCTTTCATTATCTTCAAAAAGAAATCTTGAAAAGTTCTTTTTCAAATTACCTTTTTGGTGTTATGCAAATTTCAAAATGTTGTTACCTAGAAAATGCAATGCCATCCATAGATGTTCCTTTATTTTTATGAAATTTTACATTTTTTGTTAATAATATTGGCCAAACATTAGCTTACTATATAGCTTGTGATTGAGTTTTAGATTATGTATATGAACTAAATCTCAAACCGACAATTACTATAGGACTTGGTTAATTAGTATCATTAGATAAGCCAAACGTACGAATTTAGACATCTCTCTCTCATGGTACAAAATCTTAAAACACTGTGATTAACAGTGCTTGATCTCAATAAAAGCATTATCTTCTTTTAAGAGATCCCTTTTCTTGTCAATTTGTCGGATTCAATGAACAACAATAACATGCCAAAGTTTTGCTGTTTCATATGGTTGACTTAATAATTATTTAGACGTACATTAGTACTAAGAACAAAAATTTTCTTACTAAATGACGGTTATGCCCCTTGTGTTCATTCAATCATCAGGGTGACCAAGCTTCCATTGTTGGTGGTGCTATAGAATTTGTCAAGGAGCTTGAACATCTTTTGCAATCTCTTGAAGCTAAAAAATTTGTCATGACTCAACAACCACAAGAAGATGACGATAACGGAGGCCATGATAGTAATTTCACCAAATTATCATCAGCTGCCCCTCCATTTTCACAGTTTTTTTCATACCCGCAATACACTTGCTCTCAAATTCCCAACAAGTACACTTCTAAAAGCAAGGCAGCTATTGCGGACATTGAAGTTACTTTGATCGAAACGCATGCTAATCTTCGNAAAAAAAAAAAAAAAAAAAAAAAAAAAAAAAAAGAGAGAGAGGGAGTACTGTTCATGTATGTATCGCTCATGCAAAAGTGTGTAGATGAAAATGACTTTGCATTGAATGACTCGACGATGCAAAAACCAGCTTTTGAGATACGACTAGTACTTTCCTTATTTATCATTAGACACAAATTCCTTTTTCTTGGAAAATAAAACTTTCATCATAGTCAAGGCAAAAGAAATACTCAAAAAGGGTCCATCAGTTTTACTTTATCAACTTTTTGCAAAATTCAAAGTTCAAAACTGTGAATTCAAGTGTTTGGGAAGTTACTTTAAATTTGGAATAACCAAGTCCAAACCAACTACTAAAATCAAGTTTTTTAATTTAAAACTTACAAATTTTGGATAAATAAGCACAAATTAAAAAGTCTTAAAAATACTTAAACTTTTTGTATCAAAATGGATTAATTTTTGTAACATTTTAAATAAAAACTTGAAAACTCTATAAAAAGTTTGTCTAGGGTATTTTTGGGAATTCCACCAGGTATTACCCAATTTGTTAATGTTTTGGAAATGATGGCTAGAAAAGGATGTTTGTTTCCATAAACCGATGTTGTTTCCTATTTTTGTTACTTTGTCTTAAAGTTAAGGAAATATTCAACATAATATATTAAAGTTCATTGAAAAATGATGAAAATATGACCATATGTTGATGTAAAAAAAGATATGTAAACATGCAGGTGGAAGAAGGATGCAGACTCAATTCAGCAGACGAGATAGCAGGTGCAGTCCACCAGATGCTCAGAATTATTGAGGAGGAAGCTACCCTATGTGTTGTTGATTCTAGATAGAAAAAGAGATGTTTGTGCTTTTACCATTTTACCTTTTAGGCGTTGTAAAATCACCATAATGCCACCCATTCTCTCTCTAGCTTTGTTCTTGGTGCACAGTAGTAGGATTTGCTGTTACTTTGAATTTCAACTAGTTCAATTAGATGTTTTGGTAAATAATGATTTGACTTATTGAAGTTATTTATTGGTGAAGAGTGATTGCATTTTTTTTTTCTAAGAATGTTGACATTTGTAAATTAGATATTTAGTTTTGAACCAAATAAGTAAAATGATTATTAGACCATTTCAAATGGTGTAGACTATATCAAGTTTATATCATTATATTTATACATCAGAATTGTATAAACTTGTTTACTTGACTATATAAACCTAACAATGGTCTGATTCACTTGTCTTTTAAGTATATTTTAGTCTTAAAATGTAAATCAGACCCGCATTAATTTCTCTTTCTCTAGTACATACGTTTAAACCTTGCGCATAAACATTGTCATGCAATGCTAAGTATATACTATAAAGTCCACGTACGATGACAGCTCAACATAAAACTCTTGTGTAAATCTCTATTGAAAATGCTCTTAGTCACTCGTCTCAAGTTAAGTGAATTTTCAGTTTGTAAACAATTAAAGCGTTTTAGAATGTTTTAAATAATTACATTTAAGTAAACATTTTGTTTATA

AabHLH46

AAAACACAAAAGAAAGGCGATATCTCCCGACTAGTATCCGGAGAAAGCACATTAACCGGACGACCTCTTACGATCCCAGGAACAAACGTTACGAGTTATCAGACAAATCAAATCGGTTCAAGTGCATCAAAATATTCGTGATTATTTGGTGGAACACTGTTCTCGTGAAGTTTAATTTAAGGGTTTTTTAGATTTATGATGTCTTTTATTTTTAATATACTGTTCTTAATTATGAATAAATGTTTAATATAGTTTTAATTAAATTTGTTATAAAAATAGGTTAAAGGTGAAGAGAGCGACGATCATTAGTGGTGTAAACTGAGGTAAGCAGTGATGTGTTTAATGATGATATGATGCCGAGTTGAAATGTGAGCGAAGGTGACGTCTAGTATTAGGAAAACGCATAAAGTATACTATATTTTTCTTTTAATGTTTTTATTTTTGTTTCCCATTGTGTACAAGTAATAACAGCAAGGATAAACACGAGTGACTTAACTTAACAAAAAGGGAAACCAAACTTATCTGAAACCAACAGAAGAAAAACCAAACCAAACCAAACCAAACAGAAGAAAAATGCAACCGACAACCGGTGGTGGTGGTGGTATTGGTCTCTCACGCTTCCGGTCAGCACCAGCAACATGGCTAGAAGCACTCCTGGAATCTGAAGAAGAAGATGTCATCATTGATCCACCCAAACCACCTTTAACACCACCACCTCATACCCATCCTTTTCATCAACAACATAGTACTGGGCCCACTTCTAGACCTCCTGCTACTTACGTGGATCCCAGTTCTACTATGTTGTTACCACCTACTAGTAGTGGTGGTATTAGTGTTGCTAACAGGCAGAATAGTTTGCCTGCTGAGTTCTTTTCTCAGATCAATGCTCCTGGAGATGGTACTTTTGTTTCGGGGTACTCGAATTCTGGTTATGATGATTATGTTTCGCCTTCTGGGTTAGATGGTCAACGAGCTAAGTTTACTACACAGCTGGTTAGTCATTTTTTTCAAAACTGTACTAGGGCGGTGGGTAAGGAAAGGGTCGAAAATAGTCACGTGATACCTCTGTTAGGCCGCGTACATCTGAGTCAAAACTCGCCCTTATGTTTATATTATATGTACATTATATTTTTCGTTTTTGTTTGTTATGGTAATTAAGTACTATAAGTCATGAAAATGGACTAGTTTGGTATTTTTTGGGGTAGCTTCAAGTACTAGATTTAAGTTTAGAAAAAACTTACTATATTTAGTAATTAACAAAACGGCTTGAAAGAAAGTACTAAGCAACTTGCTTTATTAAAATACAAGAGTCAATTCATATAATAAGCTAGTTTTATGTGACAAAAAAATTAATTTCCAAAAAAAAAAACATTTATATAGCATATTCAGAAAATTTGTAAGGCTTATATATATTTAGCTAGTTACGAGCTGATAATAGATAGAACTTTGATTTAATTTGAATATTATTCAGAGTGGAGACCAGAGTGCATTGTTGGATGCTGAGATGGACAAGCTGCTAGGGGAGTCAGTGCCGTGTCGAGTTCGAGCAAAGCGCGGATGTGCTACTCATCCTCGGAGTATTGCCGAGAGGGTAATTTTCTTGTTTTAGCTGTTATTGTTGTGGTTGTGTTAATAGTGACTGGGGAGTAATTAACTTTTGGTGAAATTATTTATGTGTTATAGGTTAGGAGGACTAGAATTAGTGACAGAATAAGGAAGCTACAAGATCTGGTTCCAAATATGGATAAGGTAAGTAAATCCCTTTTAGTCCTTGCTTTCCATTATTAGTAACTGCTTTAAATTACAATGGATACACTGTACTGCTGCTGCCTTTATAGTTTTATTTGAGTGCTTATCATATGCCAGTAGTAAAGGTACCTAAATGAGTAAATTCAATGATTATGGGGACCATTATGACTTTTAGACGTCAAACTAAGGCCTACACGTTGACCACGCCAATCATTTTTATCACCATTCATCGAGGTAATGTGACATTATTGCCGTGCTGCCGAGGTTTGAAAGTTGAAACTTTTGGTTTACGATAACTTGGTATATAGTTCATGAACCACTTTTAATCATGGATTAACATGTTTTGTTTTAGAGGTAGGTGTTTGAGGATTGAGATTGTCAAAGATCTTACGAAATCTCTTTGTAGTCATTTATTCATGTATTTTACGAGGTTTAGTTAAACTACGTGAGTAGTTGGGTTAGTTGTATTACGTGTAGTCATTTCAATTCGGGGTTTACCAAGTCCAATGGTAACAATGGAACTGTAAACTGTCAAGTTGTGAGCTTGAGTCACCACCTCTATCGGTTTCTTTTCTGTTAAAAGATGTGCTGTTACAGTTGAACTATTTTAGCCACAGCGATACAAACATGCGGCCATTACGATGATTGTTATTACTGAAACTGAAGACATGAAAAGCCGCATATAATTTTAGAAGTGCAGGAACTTATCTAGTTCCCACATTGGATCCTAATGTATATCAACAGGACCAGCACGAATCTTGAGTAGTATGTTTAAACCTATTGTGGTAGGTAGAAATGATTGATTATATAGCCTAGGCCTAGGCACTAAGGTTTTTCGATCTTTATGGGCTTTTTGACTACTCTTTTATCTCTCCTTTGTTTCGGTTCATATTTAGTGGAACAGATGTGGTGGGCATCCAAATATATTGTTTTAATGCTACTTACGAGTGGGTTAGTAACTATTAGCCACTGCACGGCTAATCTTATTAATCGACTCTTGTAATATTGAGCAAGTGGAGGGTTAGAAAAAGTTTTTAATTGTCAGTGCTTTTTGGTGTTTGGTTGCCTCCCATTTTTCGGTTAAGTATATAGTCCATATCAATTAAGGTCTTGCTTTGGCTTCAAATTGTATATATCTAAATATGATACATCTACATGACCACTTTCTAGAAAATGGGCCTTTTTTTGTTCATATAATGCGCAATGCTGTCATGATTCAGCAAACCAACACAGCGGACATGCTAGAAGAAGCTGTTGAGTACGTCAAGTTTCTGCAGAGACAGATTCAGGTACATATTGTCACTTCCATGCTTTTATTTGCTTAAAGCCGTCATGAAGATGAAACTCTCACTTGAACACCAGTTTCATGTAGAGGTGGCAAAATGGGCAGGCTGACCCATTTGGGTAACGGGTTGAAATCGGTCATTTTTGTCGGCTTAAAATGGTCTGGTCAGTTTGGTTTGATCTGTCCGTTCCTAAATACTTACTTTTTGATATCAAAGATACTCAGACTATTTTAGCGACAATCTAATGTTATTAGCTTTTTAGTTTAAAGATCCAAGAGCTTGAATGCACCATCTCAAAATGTGTTGGAAAAAACAGATTTTAAATAGCTTTTTCAAAGTTTACCCATTTACCCTGTTAGAGATATAATATGCCAACCCAAGTTGACTCATTTATACGTTAATGGTTTAAAATTTCCACTTTATTCCGTATATAAATTGAATGGGGTACTTCATTAGAGTAGTTGACTATGCAATCAAAGTGCATTCTGATGGGATTCAATAACACCCACACTCTTCCAAATGCAGGAACTTAGGGAGCATCGAGACAAATGCACATGCTTGGTTAAAGCTTGATCACAGGAAAGAAGTAGTCATGAATTCCCTAGACAGACAACAAATCGATGGTCTTCATTTTGTCAAGACTCTTTCGAACTATGGTTCTTTCAAATTGTCAAGACCTCTTTCGAGATCACATTGTATTTTGTTTCAAGATCTGGGAAATACCCATCAGGAGTGAGTTAATTAGTACTTATGTAGTGACGTTTGTCACTCTCGCTTGTATCATGTGTACCGTATCTGGTCATGTTACATGTAGGATTCCGTTAGTATGCGTTAGTAGTAAACTTTTATAGATAGAATGACCGGTATGTATACCTGTGTCACTAGTTGCCTACTCCCATAAACGATCTAGGGAGCTTGTATGTCATGATGCTGACTATAGTGGTTCGGATGTGTAACACGGGTATAGGAGTGTACAATGTTACCTTTCAACACTCCAGATGAATAATTGTACGTGATATGTAGTAGTATTGGTTCAATGCTATATGGCACGAAATAAATAGTGTGGTTGGATGATCTAAGATCCTTTTTCAAACCTGCTCCCATTTCGAGTCAGGGACAAATTGGGTTTGAATTGGTTGGGTCAACTTGGTTTGATCAGTGAGTT

AabHLH47

ATCACACTACTAGATGTGATTCACTTACATATAAAAGACGTACAATCCAAGTAAAAATGTGAAATAGTTGTACGTCAAACTTTACAATATATGAACAATGTGAAAAATGTGTTTGATTAAGACCCTACTAGATGTTGTAAATTCAAATCCATCAAATAAAATTTCTATACAAATAACTCGCTCATATATATCTTATGAGCTTTGAATAAAAAACTTGCCTATGACTAGCTTGTTGGTTATGAGCTACAGAATGAAAAAAGAAAATCATATAAGATACGGAATGAAATAATATCATTGAAAAAAGTAATAAAAGTGACAAAAATCTCTCTTGGATCCATGGACTATTGTTTTCCCCAATCATGCATCACTTTCTCTTTGGCATGAAATAATCATTTAAATTGTCTTTGCGGCTGGTGACTAATTATTCATTACCTTTTCTAGAAATGATACCATTTAATAATTATCAAAGTGGTGAGGTGACTTGTGGATAACCATGGTTTTTCATTGGAGAACAAACCCTCACCATTTACCTTCAAATATATCATCAACACCATTCACTACTCATAGACCTTGTTTCATTCAAAATGGACAATGTTGTTTGGTCTACTTCTTGGACACAAGATGGTGGTGTTTTTGATCAATTCAACAACTGGGATCGTGCTAATTTACTAGTTCCAAGTGCTCATCAACTAAGTGATTCTAATTATGGTGGTGATTGTGGTGGTGGTAACAATGTTGGTGAATGTGAAGATCATAAAGGGGCTGGATCAAGTGCTTCCAAGAACCATAGTGAAGCCGAAAAGCGGCGTAGAGATAGGATTAATGCACATCTTGCTACTCTAAGGAGACTCGTCTCCAACTCGGATAAAGTTAATATTCTATCTCTTTTTTCTTTTCTTGACAGTTTAGCTATGCCGAAATTTCGGTTTAAACGTGTTTTAACGGCGAAACTATAGTTTTTGACACAAATTTTATTTTTGTGACCATGAAAAGACCCCACTATAAATGTTCTCATTTATGTTGCCATTGAATATTTGTGTATGCAGATGGACAAGGCAACATTATTAGGGAAGGTCGTTGAGCGTGTAAAAGATTTAAAGTTGGAAGCAGTGGAATTAGGCAAAGTGTTTACAAGTACCGACAGATTTTGATGAAGTGATCATTGATCATGATTCCAAAACTCTAGGCCCTTGTAACAATATTCTCATCAAGGCTTCTGTTTGTTGCGAAGATCGAGTAGAACTATTTTCGGAGATCAAGGACGCCCTCAATAGCCTCGGATTAACCATGGTTCAAGCCGATATGACTTGTTTGGGTGGCAGGATTATTTGCAACTTCATCCTTTGTTTGACGAATAATACAAGTGAGAATGAAGTTACATCAATCAAACACTCTCTCAAGATATTACTAAATAGAATTGTTTCGACGTCTTCTTGGACAATGTCATCAAACTATCGCATCAAAAGCAAGCGGCAAAGGTTCTTTTGTTCTTCTAGTTATGACACCAATGGCTGTGAATAGAGATTTCACTTCTAGCTGCATTATAAACGTTGCAAAATATAATTTGTTGTTCAGAATTGCAACGGGTAATCACAAATAACGTACTCTATTTGCTTTCAAATTTTTAGGTGTTGATTTTAAGCTAGTGTTATAAGTTTATTATTTTATGTCTTCTTTGTTTCTCCAATTGTTTTGCTATGGGTGAGAGTTTTTTTTTGTGACGGGTGACTATTGTAACGTGAGTACGTGACAGTCAAAATGTATGCAAAATCATTTGTATATATACTTAATTATTTGATGTTATACCTTAGATCAAATAATGGTCAAACTTAACTATATATAGCACTACTTTGTGATCTAACAGAAAACGACATAGAATTTGTGATCTAACTATATACAGCACTGCAGTAATGTTGAATTTAATGTTGACTATAAAGTATAAACACTTTAATTTCCATGAAATATGAAAAAAACTGAAAAAGCACATTGATACTAGCTGCTGTATATGCGTAAAAACTATTTCTTTAAACATAAACGTGTGTCAAAAACTCTGTCAATTTCATCTTTTATAAAGCTATTCAAACGTGTGGAAACAGAATA

AabHLH48

AAGATACGGTTCAGATAGCTACTCGTAGAAGTTTCTTGAATAATCGCTATTTGTCAAACTCAAAGAAGAACGATAGATCTATCGTTTTAACGACAAAGTCGTGATCTTTGACATTTTGTGTCTCCTCTCTTTTCACCACTTTTTACTCAACTTATATTAACTTAAAAGACTGTTTAGGACTCTCTACATGATTAAAACTTTCATAATACAAGGTTAAACACAGATCAGTATCTTTTAGGATTTATCATATTCTGGGTCTTTTGGTTAGAATTACTATATGCGATGTGAAAGGTAACTTTAGCTGTAGCTAAAATACACTTGATACCTATCTCATGTCCGAAAGTATTTCTTGTAATTTTGTTTTTCGAAGCTTGTTTTAGTCGGTCAAAGACACACACACAACACGCCCGGACACCGCTAGACTAGAAGCATTTTATTTTCCAAAACTCGTACTTACATTTGAAAAGTACTTTGAAAGACTTGTTCCTATCAAATCAAAAGTTCAAAAATAAAGGTTTTTCATTTAAACTAAAAACCTTGTCAATGAAAACTTAGCAAATAGCAAGTTAGCAACCCATTGCCCCTTCCCTTCTGCAACTTCTACCTTCCTTTTCTCTTACACACACAAACATAACCATTTTCTCCTTTTTCTCACTCTGCAATATAGCCACCAACATGAACAACAATCATGAATATGAAACCAACAATATGAACTCTTTTAACTCGCTACATGAACTCGAAGAAAATAGCAACAATTGGTACTTTAATCACCATGAAAACAATCTCTCTTTTGCGCCGAGTTTACCTTCAACTTACCAAAATTTCGATACAAAAACTAGATTTGATTCACAACCCCACATGGATGTTCCAAATATAATCTCAAACCATCATTTTCCAACGAACCATTTGGAAAACGCGTTTGGTGCATCAGGTTTTCAGGGTCTTGGACTCATGGGTAGTTCAAGTAATACAAAAAGTGGCGATGGACCAAATACTTTGGTTATGGATGATGTTGATGACATCAGTTTTGATGAGTCCGTTTTGAATTATGAGTCAGATGACGTCGCTAGAAAGATGGATTCTGGTGTAGGAAACATAGGAGGTTATAGGAAAGGTAAAAGAAAAGGGTTTCCTGCAAAGAATTTGATGGCAGAAAGGCGTCGTAGAAAGAAGCTGAATGATAGGCTTTTCTTGCTTAGGTCTATTGTGCCAAATATCAGCAAGGTTAGCGTGTGTTTAGTTATATCTAATCCGGCATATAAAAAAATATGTTTAAACAACGACGATGATCTTAATTTATTTTTTTGAATGCATATAATTTTAGTTTCTCTACGAAGCAATTTAGGGACTCATTTATAATTCCGTTCAGCAAAAGTATTGCTGACTACTTTGGTATAAACAAAACAAATTAAATAGTAAAATGGTCTAGCCATAAATTACATGTAAGAGTTTCTATTTTTTTCCCCTTCTTTTGCATACTTCTGGTGGTGAATATCTTATAAGTTATTATTACCGGTTATATGAGGTTTAGTACTTTCCTTGTGATTGAATGGTACAATGTTTTTGTTTCAATTATGTTTGAATGTTTGCTTTTTAATGAAGTCACATGGATTCTGAAGTCAATCAATCCTATATGGTACAAACCGTAGGTTTTTGGACCACCGCGAGGATATTGATTTTAATAATCGTCATCCAACTAATCCAAAATTATCGAAATTTCTATATTCCACTCTCATCTATATTCATTGTTATTATGACTAATATTCGGCAGTGATCTTGCGTCTCATGCATTTACAACAATAAATTTGAATTCTGTTTAAATTTTGTGATTATGTTAATGTTAACAGATGGATAGGGCTTCGATTCTTGGAGATGCCATTGAGTATGTCAAAAAGCTTTTGCAGAATGTAAATGACCTTAACCTTGAACTGGAATCAACCCCTTCAACCTCTTTGATGCCCCCTGTTGTCGCCCCTACTACCACCACTGGATTATATCAGCTAACCCCGACTGCCACCATTGTCCCTTCATGTATCAAGGAAGAGGTTTTTCAAACTGGACAGCCTATCAAGGTAACTTTATTGTGCTTGCTTATCTTCATAAGCGAGTTCGTGATTTTTGGGTCCAAAATTATTCATAAATCATCCACACGCTCATATTAACAATCCTTAGTTGTTTCATGTTTCTATGCTTTGAAATAGGATCTTGCTAGTAATTATCACCTTCACTAGTAACTGGGAGTAGCATCAAATAGCTTACGATAGAAGCAAATGCTCTACAGCGTAAACTAATACTGTTCTCTGAAAATCGGCAATTCGGCATTAACCAGTTTTTGGATATAAAACATAGTGTTTTAGTGTTGGACACTAGAAAACTTATATAATCTTATATACACTAAATTTCTAGGCCAAATTATCAACAATCTTTGCTGCTTATAAGTGACTTGCATATAATTTCTAGGCCAAAATAGAACGGGGCTAAAACACTCCCTTTACTGGCATTCTGTTTAAGACGGTTACGGATATTACAAAACAAACATATACCATGTATGCTTAATTATATTTTCATGTATTTAGTAACTCGGCAATCAAATACTAAACGGAAATTAAAATTACAATTTGTAACACCACAGACTTAATTATATTTTCATGTATTTAGTAACTCGGCAATTAAAAACGGAAATTAAAATTACAATTTGTAACACTACACACTTAAGGCCGAAAGTAAAACATTTTAGTTCATTACATAGTATGCATGCTAATCTGATATCAGCAGTTTTAAGTTTATGATCAAAAACAGCATAAAATGTCTGTGGAATGATTTATGCTTTTTTGTTGTATGTTCAATGTAGATTGAGGTGAGGCAAAGAGAAGGAGGGGTGTTGAATATACACATGTTTTGTAGTCAGAAACAAGGCCTTTTGTATTCTGTCATGAGGACTCTTGAAGATCTCGGTTTAGACATCCAACAAGCCGTCATCAGCAGTTTTAATGGTTTTGCACTCGATGTTTTCCGAGCTAAGGTATATAAATTTAACTTGAAATCCTCCATCTAGAGTATATATAAAATATATGAACATTGATCTTATAACATACCGATAAGAGAAGATTTAAAGCATTAATTAATTTTGGTAAGTATTCGGTTTTTTGCAGCATTACCTTGAAGGCCAAGATATTAACAGTGATCACATCAAGGCGGTGCTGCGTGAAACAGCTGAATATCATGGGGTCATGTAGATGTGGTTCACTGCCATCAGTGGAAGCAAGTACTTTAGGAGTTTAGAGAGTTCTAGTAGTAGTCGTAGCGTCGTTGAAATTAATGTTTAAAAACCTAAGCCAACTTTGTCGCTTGAATCGGGGGCCTGATTTGGAAAATCTAATTTTGAAAACATGCATGTTTCAATTTGGCAACAATTGTTTGACAGTTTACGCTAGTGAAACCTCTGGTGGTTCAATGATTTAGACCTAGACGGTAAAGTTTATATAAGTGTGTAATTTGTAGTATATAGATAGGGTGTCAATGGAAACCGTCTTCTGTGACATGTTCGGATTCTGGATCAAGAGCAAACCCACTAGAGACGAACTCTAAACACATTTGTTTAGCTTAGCCCACAGTGACAGGTTATAATTAGAGCGCGGTTATAGCAATATAACTTAGTCCTAAGTGACGTTTAACAATTTTGATCTCGTCGATCCATTTTCTTTTTTGCCAAAACCTAATCGATGACCTCCTTCCAGTTGATCCGTGAAAGAATATAAGTTGATCGCTAAGAGCGGTAACTTCACCCAAAATAAATTGCTTTTAGTTTAATTAAGAGTTGTTGAAACAAATACACCATAGAAAGCTAATTGCTATTTTGGGCGGTTTGCCATTTATAGAAATAAAATAATTGAGTATAGTAGTAATTACATACACTAATGATCATGTTTTAAACAGTATAATGACCAGCTAAACTTGTGAATTATGTGCCAATTCTCTGTAAAAAACTGCATTCATAACTTTTAATTTGCATCTCGAGTA

AabHLH49

AAACGAGCTCGAGCCTAGCATGATTAACGAGCTTTCGAGCTTTATTTCATTCAACATGTTATATAACTTTGTCAAGCCCGAGCCAAGTTCGAGCTTTTGAAAATTTTCACGAACCGAGCTCGAGCTCAAAATATAAAGCTCGAAACGATCTCTAGCCCAAGCCTGTTTCCACCCCTACTTGCCAGTAATTTAACTTTGTCACTAGAAACGAGTACGGAGTTATATAGCTACCGAATTTAAACGTTTATATAAAAAGATCCGAATCCGATAGACCAAGTTTGATACACTAATGGCCCACTAAAAAACAATCAAATGGTTAAGACAATAAAAGCACCACACGATAATGTCTATAGCCTAGTCACCATCGATGTTCAGAATTCTAATAAATATTTCCTGGTTTTGGCATTATATTAAAGACAACAAGTTCATCTTTATACTCTCACAAAACTGTTTGTTATTTTTTGGCTTCACTCCCTAAGACCTATATTAACAGTTTTGTTTTGTTTGTTTCTAATCAAAATGATTAGTGAGTGTACTTTTTTACCTGATTTAATCCATACATATGTCGATCAGTTTGGGGAAACCAACTTCTTGTCACCTGAGACAGTGTCTGCTGACGATGTATTTAGGGGTTTCGTGGCTGATTTGGAGGGTGGTTCTGACAAATTTGCGGTTATGACATCCGTTGGGCGCTTACACGAGGCTGTGGAGGGGCGTAAGAGGAAGAAAATGAAGATGACTGCGGTGGAGCAGGATGGCGTTGGTTGTGGTAATTCAGATGGGCAGCAAAAGGTGTCACATATCGCGGTAGAGAGGAATAGGAGGAAGCAGATGAATGAGCATTTGAGTGTCCTTCGTTCGCTGATGCCTTGCTTTTATGTCAAGAGGGTAATTCATCATGGCAAAACGGTTTTAGGGTTGTTTGCTTTGTTTATGTACTTAGCACGTTTTGAATTATTTAGTACTACATTTATTTCTCAACTATAAAATTGAAGCTAAAAGAACAAATCACATTTACCAGTCTTCCTTACTTGATTATATGATATTATATTGTTGTTTCTAAACAAATATGTGATTATATTGTATAATACTTATAGGGTGATCAAGCATCAATAATTGGAGGTGTAGTTGATTACATCACTGAATTGCAACAAGTTCTACAATCCCTAGAGGCAAAGAAGCAAAGAAAATCTTACAATGAACTAGTCATGAGTCCGAGGCTACTTGCGAGCCCCAGAACCTTACCTATCAGCCCACAAAAATCGCAATTTAGCCCTAGACCAATTAGCTTGCCCATTAGTCCGAGAACCCCGCAACCGTTGAGTCCCTACAGACCCATGCCACACCTTCCATTCATATCACCATCGCTTGATTATCCTTCACCTTCTAATTCTTTTACAACTTCAGAGAGTGGTAATGAGCTCATGGCAAATTCAAGGTCAACGGGTGCTGAAGTTGAAGTGAAATTTGTGGGTGGTAATCTTCTTTTAAAGACTTGCTCGAATAGGATACCAGGCCAAGCAACAAAAGTAGTAGCAATTCTTGAAAATCTATCTCTTGAAGTTATTCAAGCTAACATTAGTGTCGTTGATGATACCATGTTTAACTCATTTACCATCAAGGTACGCCAAATATTTTATTTCTCAGCTTATATATTTTAGTTTCATTTTCATTATTTGCATCTGAATTTATTTGTGCATGCATGCTTGCATGTATTTTTTTGAGTGATATCACACTTTAAGATACGAAGTACTTTTTTATATAATATATATATTTTTTAGTTGGATGCACACATTTTGAAGAATTGCTGTGTTTTTTCACTCCCTCCGTCCCATATTAAATGTTCACATTTGACTTTTGAAGTCTTTTTTCTTCAATTTTGACCTTAAATATTTTTGTTTGTATTATACAATACTTGATGAAAATTATATCTAACGAAAATACATTTAAAACTCAATTCATTAATATATTTTGCATCAAGTATTATATAACACACATAAAAATATTTAGAGTCAAAGTTTATAAAAAAAGACTGAAAAATCAAAGCGGACATTTAATATGGGACGAATGTCGTACTATTTTTTAATACAAGTACACTAATTTTCCCTCATTTGTAAACCATTATTTAGTGTTTGTAGCTTTGGTTTTGGTTCCAAATTTGTTCACTTTAAGTTTCGTTCATATTACTAATTTGTTGGGCTTTGTTCTTTTTGCAGGTTGGAGTTGCATGCAAACTAAGTGCTGATGAACTTGCTCAACACATTCAAGAGACATTCTGTTAACTTTTAACATGACTAGCTAGTTTTCAATCTCGATCCTTTATCACCTTTGTGTGATGGTAATCTCCTTTTAAATAAACCCGATACACATTATGTAATGTACTAATGTATAGTCAGTGTACATTGGGCATGATTTCAGGAAACTTAATAAGCCTAGCTAGAAAATGAAAAATGGGCAATTGTACTAGAGATTGTACGTGCTTGACAACAATTCTTAACCCCTCCATCCCCCAATTAATAAAATTTTAGAGTTTGTATGTTGATTACTTTTTTTTTTTTTTTTTTTTTTTTTTTTTTTTTTTTTTTTTTTTTTTTTTTTTTTTTTTTTTTTGAAACATAGACATTCTATAAGACAAAACTAAGGAACATACAAATCACTCCTCGCAAGCGAGGTAATTTCATGAGGAAAAGAACTATCCCAATTAAATCCAGAAGCAGAACAAAATGCATGAGAAGCAACCCAGTGAGCTACTTTATTTTGTTCACGCTTAACCCAAGAAAATGTAATATGCATATTCTTTGCCTATAAGCGAATGTTATAGTAAAAATAGGTACCGGGAAGACAACTTTGTCTACTGATCACAACCGATACTTGATTGGAGATGATGAGCATTGTTGGAGTGACAAGGGTGTGTCAAATATTGAAGGTGGTTGTTATGCAAAGTGTATTGATCTCTCAAGGGAGAAAGAACCTGATATCTGGAATGCCATTAAATTCGGGACCGGTATGATCTCTATGAAACTTTCAATACTTTAACTCCTCGGATGTTTTTAAAAGTCTTAACTTGCAATTCATTTACTTTCAGTGTTGGAAAATGTTGTCTTTGACGAGCATACTAGGGAGGTGGATTATCTAGACAAATCTGTCACAGAGAATACTCGTGCAGCATACCCCATTGAGTACATCCCCAATGCCAAGATTCCGTGTGTGGGTCCTCACCCGAAGAATGTTATTCTGTTGGCGTGTGACGCTTTTGGTGTGCTTCCACCAGTGAGCAAGCTAAACCTTGCTCAAACGATGTATCACTTCATCAGTGGCTATACAGCTTTGGTACACACTCATATAAATTAATATTCAATTTTTCACCTGGTTTGAAATATTAAATAAACTGGCAATGAGTTTATATACCTAATTGTGTAGGTGGCTGGAACTGAGGAGGGTGTAAAGGAGCCACGTGCAACGTTTTCAGCCTGTTTTGGTGCAGCGTTTATCATGCTACATCCCACAAAATATGCAGCAATGCTTGCTACCAAAATGGAGAAACATGGTGCTACTGGATGGCTTGTTAACACTGGTTGGTCAGGTGGCAGGTATAAAACTAAAAAACGTACTAATTGTTTTTATGGGTCATCTTTTTATCTATAGGTATTTTGAGTCCCTAATTTTTTCTTTTCTATTTTCTGTAGTTATGGTTCTGGGAGTCGTATGAAGTTGGCATACACACGAAAAATCATAGACGCCATTCATTCTGGACAGCTTTTGAATGCTAACTACAAGAAAACCGAAGTGTTTGGGCTTGAGATCCCAACTGAGGTCGAGGGTGTGCCTTCTGAGATTCTTGATCCAGTGAACACCGTAAGTTGGCTCATCATTCCTCTATTTGTATTCCTTAAAACTTTGGATATATATCTTGTATGAATATTGACTTGGGGTTAAACCGAATAATGCAGTGGTCAGACAAGAATGCGTATAAGGAGACATTGCTGAAGTTGGGTGGGCTGTTCAAGAGCAACTTTGAAGTGTTTTTGGACCACAAGATTGGGAAGGATGGTAAGCTGACTGAGGAAATTCTTGCTGCTGGTCCCAACTTTTAATCTTTGGGTCGATCCGTATTGAAGAAAGAAGTGAATGTTGAATTGGTGATAGAAATCAAGGCTTATTATCAGCTTAACATACAACACATATATTATTAGTTGTGTGTTTATATTTAGAGTAGTACGTTATTTGACATTTTCAACTGTTCTTTTTAATTTATCTTTATATGAAATAAAAGAAGCCATTCACTTCAGCTTGTTATGGCTGTTCTTGATGTCTATTTTTTTTTTTTTTTTTTTGAACATAGACATTTTATAAGACAAAACTAAGGAACATACAAATCACTCCTCGCAAGCGAGGTAATTTCATGAGGAAAAGAACTATCCCAATTAAATCCAGAAGCAGAACAAAACGCATGAGAAGCAACCCAGTGAGCTACTTTATTTTGTTCACGCTTAACCCAAGAAAATGTAATATGCATATTCTTTGCCCATAAGCGAATGTTATCAATAAGAGCTGCAAATCTCCATGGTGGGTATGCTTCTGAAGATGAAAAGGAGATAGCCATTTGAGAATCCGATTCCTCAATAGCATTTAGCCATCCATGACTAGATACAAGTGAGCAAGCAGAATGCACCGCAATGACCTCGGCATGAAGGGGAGAAACGGCACAACTTAAATCACCGTGAACATAACGAAGAGAGC

AabHLH50

TGTAGAGGAAGACAATTGACATTTGACTTACAATAAGGAATTAAAATAACGTAGTAGTAAGTATGGGTTCAAGTAAACATAGTACGGTATTAAGTATGCAAACATTTGTATTTTTAACCAATTCATGACTGTAGTTTTGTTCTTGAGGACCAACATAATTAGTTTTAAATTCTAACTCATCTATATGCTTGTATTGTAAATGTACTATAACTCTAAGCAAGGGTTAACTGATATAAAATGATCCAAAACTTAAAACTCCAAAAAATCTTTTTTCCACGCACTAAAAACTTACAATTGAATTAAACCATTCGGTCCAAATAACCATATCTTGGCCTTAATGCAACCACTTATGACTTATACTCCAACAAGATTCGTATTCATAAAGTTTATTAAACCTCAGCCCCATGATAAATCAAACACCATCTTAATGGAGCTAGGACTATAATTTCATGTTTCATCAAAGAAGCAAAGAGTTGAGACAACATAATTTCCACCTGTCACGGGTGTACCGGGCCCCGTATACTGCCTATGTAGCCCTCTAGCCCGACATATGACGTGTACATATTGACCATAACTAAAACTAAAAGGACTCACACTCACACTAACACTTAACACCTACAAATATCAATCGATACATTGTGTTCTCATCACTCACAACTCAAACGTGAAACACATATTTTGAACTACTTCAAACGTTTCTTAATTCTTCTTGTGTGAAAATAATATGATGCAATATTTACCAAGTCATGGCTATGAGTTTGCTAATTGTGCAAGTATTATAAGAAATTTAGTATATGATCATGGAGCGAAAGATTTGTCAAATGACACGGAGGCAAATGTAGCGGCAGAGGCAGAGGCGAAAGCGGCAGCTGCTTGTAATCGACATAGCGAAGCTGAAAGGAGACGTCGAAAGCGAATAAACGGCCACCTTGCCACCCTCCGGAGCATACTCCCCACTACCGTTAAGGTGCGTACTATATATACACTTCTACACTTGAACATATTCATTTAGTACATATATGCTCCCTTCGTCTCAAATTTAATGTGCACTTTCAGTTCTAAATTAAAAGAGTGAAAGCAATACATTTTAATAAGGTTTAATTTGAGAAGAAGATTATTATTATTATTATTTTACTTAGTAATTAGGTTTCAAATATGAAATTTTCCTAGTATAAGAATTAAGCTCAATGCATTCAACTTGTATATGGGAAACGTAACTTTTGTCTTGTCATGATTTTGGTTGCTTTATGGATCATTTACTAAGCAATATTGTTTTTGTTGCAATTCATTAAGCAATATTCGATATTCTCTAATGCATATTTGATTAAAAGTTAATTGGTTTGATCTCAAATGTCGTGTTGATTGTAATAAGCTATTCTTTTCTAACAGACAGACAAAGCATCATTGTTGGCGGAAGTAGTGAGACAGGTGAAGGAGTTAAAGAAAATGGCCGCGGAAATAGAATCCGCGGCTATTGATCAAGGTGATGACATTATACGAAACAACGAGTACTATATGATTCCAACTGAAAAGGACGAGCTCGAGTTGACATATGTTGGAGAAGATTCTAGTACCAAAAAGATTATGATAAAGGCACGATTGTGCTGCGATGATAGGGCCGAACTGATAGCTGAACTAACGAGGGCGTTGAGTTTAGTACATGCAAAATTGGTGCGGGCGGAGATAGGGACATTGGGCGGGAGAATCAAATGTTTGTTGTGGGTTCAAGTCTCAACGGTAACTATAGACCAAGGCATACATGAGCTACAACGATCTTTAAAGGTGGTTATGGATCGCGCAACGTTATTGGATATGCCACGAAACAAGAGACCACGTAGTTTCTCGGACTCTGTCGAATTTTAGTGCTAAACTTAATTTTTGACCGGCATAAAAGATTATGTCGCACGGGTACAAATGTTATAGTAGTTGTGCTTTTTTAAGGGTTTTGTTTGTGTATGTTAGAGATTGTTGAAAATAATTAGAGTTTTATGGTTGAGTTTCGAACATATGCTTGTCAAATGTAACTGAAGTATTTTTCATTTAAAATAATTTTTTACTTCATATAGAGCAATAAATCGATAATACAAGTCATAGGATTCACACTTTGCCGGTTTGCCTTCTGGAAAGAAAACAATTTTTCTTTTTACTACTTTTTGAATAAATGAGATATTATACACCTTTACACTACCAAATAAAAGGTTTGTCGTTAATATACCACTTCTATGTTAGTTTAATTGCTCAAATTTTTTATAAGTAACTTGATTATCTTTGGTCCACTAC

AabHLH51

GTGGGTATATATGGTCTATTAATGGATCACCATCAAAGTGATCTTACATCTCAAATATCGCTAGTTATACATTTTGTAAGTTATTTTCCGGAAAAAAAGAATATATATTATTATTATTATACATTTTGTAAGTAGTCACAGAAGAGATTGAAAATTAATCACTAAAATATCTCGTTTAGAGCGCGATTTGAGTCAAGGGCTAGTATTTTCAAGTCGAAATAGTTACAAAAATATATTGGAGCGCGATCTAAGTTAAAGACCCGTCTGTCAAATAACATGAAGATGAAAAACTTTTTCAAAATAGATAGTCCATGGATGATTCAGGTAGTTTACTTATATTCGATATATTTGAAATCAAATATATTTACAGACCACAAAAAACTAACAAAGTAGGTACCATACCAGGGTTTCTACTTATATCCAAACCAAAAAATACAATAAATGAAGGTTGCCAAAAATAGCAGCAGTAATTTATACAAAAATAAATCTTAGCTAAAAAAAGAAAAAGTCTCATCTTTCTATATTTGCTTTGGTCAGCTCCATTTCCACCGTTGAGATTCATTCATCTGTCTCTTTATTTTATCATATTGATTCCTCTCATTTGATTGGCTGAAAATGCAACTCAAATCCATTCAACATTTCAACTCATCTTCATAAACCAACAAAAGCTAGCTAGCAGCTTCTTTTATATTTATCAGAAACAATATTTCTATATATATTTTTCTAGCTATGTATGGTGTTAACAATTCTTCAGATGCAATATCTAGAGACATGAACTCAATCTTGTACTCATCAACTTTCAAACATCCAGCAGACACTGAGTTTGCTAAGATTAAACAGCTGATATCCTTAGACAACAACAATAACTCCTATGAAAACCCTAGTACTCATCCACAACACCAAGAAAACAGCGAAAATCCACCGTTAGTAAGTTATCGGTCTACTCCTAGCTCTTTTTTCTCGAATCTATTAAACGAGAATGAAAATGATGCCTTCCAAGATCATGAACCTGAGGAAATATATTTTATGGACCAGCAACAACATAAGAAGAAGTCTGATCAATCTGAAGCATATAATAATATGAAACGTGAGAAACAAGAAATGGGTAGTAAGAATATTGAAGTTTTGGGTTATGGTTATTCGAAACAAAGTGATTTGGATTGTGGATCGTCTTTTAGAAGTGATTTGGTTAGGCAAAGCAGCTCTCCTGCTGGATTTTTGTCCTCGTTGACTGGTGAAAATGGTAAATAATATATATATCAACTTGTTTCTTCATTTGTTGTTTTTTTTTTTCCTTTCATTATTTACTTCAACTTGTTGTTTGATTTGTTATGTTTGTAGTTCATATTGATGGTTGACTTTTTTGCTTATGGTTTTTGGATATTAGAAATAGTTTCATAGATCTTAAGTTGTGCTGGTAAAGGATTTGTTACATCATATATGACTATATGTATTGGATAGTTGTAAAAACTTGTAGGCTACATCTATACGTATGTGTTTAGGTGTAAAAGTCATGTTTACCGGCACCTAGGTGCCACCGCTAGCCGAAGTACAGTTTGGTGTATAGATTCCTACATGTTTGACCTAGTGGCATAAGGTAATCCGCAAGTGAGGTCTTGGGTTCAAGCTTGGGCATCACCTTCTTTGATGTGTGAGTATGATGTTGGATTGTTGGCCATGCCTTTGGCTTCTAGCTCCTCTGCCTGGCTTAACCACCATTTTATTCCGTCACCATGTGAAGTAGTGTGTTGTCGGCTATTCGAGAAGATTCAAGGGTGAAAAACGCCAATCCATTAATGTTTTTTTCCATCTTTTTATAGTTTATAACCTACACCTAGTTTTATTTTGTTGTAGTTTAAGATTTGTTTTGATTTAGGAGTTGTAGTTTGAGATTCCTTGTTAGTGTTTATTATCTATGGAGCGATGGTTCAAATTAATCCTCGTCTACGGTTAATTCCTGAATTTTTCTATAAATACGTGTATTATCTTTCAGTTTTTATTATGTTCTCAGTAGGTCAGTAGTTATTGACAAATGACAAGAGTGACAAGAAGTTCTGTTTGGGCACCCTCAAAAGTGTTACCGAAATTCTGTGTTGCTTGTGTCGTTTTCTTCCATTCACAATGATAGTCGTCACTCATGTTGGCCAAAAGATTAACGTTAAGTTTGTTGAGAATTTTCTAACAATTTTGTAGACCAAAATGTTGTGAGTGAATGAGTGATCATGTTCCAATGATTTTTTTTCTATGATTAAATGATTAAGACTGTTGTTTTGAATATTGAAACTCAGCTTTTGCTAAAGATCTGAGAAACGGAAGTTCATCGAGTTCATTTAATAGCCACATCAGTTTCTCGTTAGGGTCATCTTCTTCTTCGTCGAGATTTTTGCCCCAAATAGCTGAAAACGAGAATGAGTTGAATGATTCCACATTCCATAGCTTGAAAAGGAGCAGAGATGGTAGCTTAAAGATGTCACAGGTACATCTTAATTTAAGAATCGGCTTGATTTATTTATTATGGATGGACTTGAAACCCACAACCTATGGTTGGTGGGGCACCATTGGTACCGCTAGACCAAAAGCCTATTGGTATGCACTACTTCTCTAATCATCTCATTATGCTTTCTCGTTAATGTGAACTTGTTTGTAGAATGGAGAGACTGTAAACCACACTCCTAATTTGGTCCACCATGTGAGCTTGCCAAAAACGTCTTCTGAAATGGCTGCTGTTGATAATTTCTTGCATTTCCAACAAGATTCATCGGTTCCTTGGAAAACACGTGCAAAAAGAGGATTTGCTACACATCCACGAAGTATTGCAGAGAGGGTACGTACAAACATCTTTATACTCTTGATTCAGTATGATCAATGTCACCTAACATTCAGGAGATATTAACCAGTCTTTCATTTGGACCATGATTTCAGGTCAGAAGAACTCGAATCAGCGAAAGAATCAAAAGGTTGCAGGAGCTTTTCCCTGATATGGACAAGGTAAAATTTTCAAGCCTAAAACGTAAATCTTGTATTGAATTACTAAAAACAAGAAATCAAATGATCCATTTCATCTACGTATGCTCAAAAAACTGTAAACACTTATCAGAGATGCGTTTCAAATAAATTGTGGACAATAATTTCACTCTTTATCTTTAATTGTACTCATCAAAACTAGAATACTGGTATAATGATATGAGTATCTTAATGAAAGTTGATAGTTGAATAATGGATCAGTATAAATTCGCACATTCATACATGTTTTATAACGGTTAGCACACACATTGACACATTTGATAAATGCAAAAGTCTCCCAAAAAAAGATTGGCGTGAGCCATAACAAGGATTGTATTGCCCAACGGAATCAAACTCATGCCCTCTCATTTTAGGAGAAGATATATAGTTTGCTCAGTGATACAAAATAAGTTGACCCATGTTGGAATAAATCTTAATGTTGGAACTAATAATATAATCAGTAGTTAGTTTAAAGTCTAATTGTATTTCAGTACGTTTTATGTTTCTGTAATTTGTAAAGCCTGTAAACTTAGGTAGAATATGGGCTGGGTCAATGTGGGCCAATATGGGTCATTAGTTTGTTAGGGTTGACAGTTATTAGTTAGGGTATCGTTTAACCTTTTGTGAATCGGTATAACTGCCACAAAGGCACCGATTCATCATGTATAAAAGGTCTAGAGTCTGTAAACAATGTAACTTACTTCAATCATCAAATACAATATACGTTGTTAACATTTGTTGTTTACTTTTCTGTCAAGATTACCTTACTGTCCCAAACGCCCTTTACAATTTCATTCTAAAACAAAACCACACCATGTCGAATTACAGAAACATGTTGGAGAGGAATTGGCTTAGTTATTATCCTGTGCAACAACCCACTGATTGCCGATGAGCCTCCCGCCCCTTAAGTTCATTTGATCCATATTTAGTAGGGTGTTATTTACTGATAACCTATTATTTGCAACAACAAAAAAATATTATTAATGGAACCTAATGCTTTCGGGCTACTTTTAACATCTCCAACCAAAATCACGGTATCTATAATTTGTCATCATTACAACTGACAGATAGACAAACTTAACTTCAAAGGTAGATAAAGTGGTAGCTTTATAGAAGATGAGACTTGAAACTGACATTTTCTTATTACTATTGTGCAGCAAACTAATACTGCAGATATGTTAGATATGGCTGTTGAATACATCAAAGACCTTCAAAATGAACTTCAGGTATACCACTACAATCGCCTATTTACATACACAGTATGGCATGACTTCACACAACGTTAATGATATAAAACTGTAACCGACTGTGTTACAGTTCTAATTCAGTTAACAAACCTTCTTTTCACCACAAACCATTAAATATATGTAAAAAAAAATTGATTCAAAGTAATCAGTGCAACATCTGTAAAATGCTGACATGGAAAATGGCTGGTTGAGTTATAGAGTTGACTTTTATTGTTTAACGCTGAGAAGCCTGTTACTAGCAGTCAAAACTGAAAATTAAACTAAAATTAGTTACCTTTTTACACAACGCCCCCAGTATGCAACATGTATGGTTTCAACTTTTTCCTTGTTCTAATGAATTTTCATGTTCTGGTTCATAGACTTTGAATGATGCTCGAGCAAGGTGCAAGTGCTCAAAAGAGCAATTACAGTCCGGTTCGACCATGTAACTTGCTTTTTGTGTTCAAATCGCAAGTGAACATTACAAAAGCAGACAAATCAACAGGTATCTGCCGCAAATCATCATATACTTAAGGTCGAATGCTAGTTATCATCTAAAATGTAAATGATTTAGTTTTTTTGTTAATTAGACGAGACATTCTGCTTTGAATTTGGGGATTAGATCCAATGTTTTGTTTGATCCAAAATGGATCAAGAACTTTGTGAAGAAGTTGATGTGATCTGAATTCTGGTCTTAATATTGTTTTTCTTTTTCTCCCCCCATTTTCTATAAATCCCATGTCCATTTATGCAAATTCATGCGTGTTTGTACTATGCAAAATCGCAAGAGATAACAACACTTTGCAATTGTTACCGTGTATAGTTGTGGGTGCTAATGCCTGGTATATGCTGATATGGCACCATAAACTTGCTATTACATTCAAAAGATGAAAAAAACATCTTATGGTGCCATGTGAGCTTTTTGCAGGCATCTGATTTGTGCGAGAAAAAGAGTATGTTCTTATTTTTGTATGGATGACACCAAAGAGACAAGTCATCTGTCATATATTATGACACTTGAAAACTTCATCGTATTCTATTAGATAACATTAACCAGCGAAAAGTACAATTGCAGGAACATAAAAAAACTACGATCGAATGACAAAACATATAAAACCTAAAATCAATGCAAGACCGTAACAATATAAAATT

AabHLH52

ATCGACATAAGATGAATATAGTTCATTATTCTGTTTCCATATCAACTTTATGTTATATATAGCTAAGTTAGTGCATGTATCTAGTATATGTGATTTTATTCAATTTCATCACATCAAATTAATCTCGTGAAAATGGAATCCTTTGAGTACTCAATTGGCCATTCCAATGATAGCAACTTTAACATGCTCCAACCCGATTTTATACCAGAAACTGACTTCGAAAACTTACTTTCCGCCATTCAATGTGAGACCAAGGATCCTAATGACATTTTTTGTGTAGACTCAGCATGCAATCACTTTACCAATGCATGTACGGCCTTCCAGTTGTCGCCGCAGTTGTATGACCAAAATGAAGCCATTGATGGTGTTGGTATTGATATTGATATTGATGTTGATGTTGATGTTGATATTGATAGTGATAATGGTAACGGTACTTGTAATGGTAATGATGCTGGCATCGGTTATGATGCGATGGATCCACATTTGTATGATCAAAATGAAGCCATTGATGATATTGGTATTGATTTTGATATTAGTAGTGGCAGTGGCAGCGGTATTTGTAATGCTAATGATATTGGCAATGGCATCGGTTTTTATGATGAGATGGATCCGAATTTGTATGACTACAATGAAGCCATTGATGGTATTGGTATTGATATTGATAATGGCACTGATAGTGGCATTGGTTGTTATGAAGACATGGACCCAAATTTGTATTTGATTTGGGACGAGGACGAGGATGATGTGAAGGAATCTGGAGATGGTGATTCCTCTGAGACGGTGACTACCCGTAATCCAAATACACAAAGGAGAGGTGGTGGTGTGAAAGGTGATCGTACAAAGTCTTTGATTTCCGAGAGGAAGAGGAGAATTGGGATGAAGGAGAAGCTATACACACTGCGCACAATGGTGCCCAACATCACTAAGGTTAATCTCTTTTTGTTACATATACTTGTGTGATTTTTCTGAAACGTTGTTTGCAGTCAATATATAACTGTTTGAGGTTAATCTTTTAAGTAATGTATATAACTACTTTTTCCGTGTCATTTTAATGTCAACCTTAAAAATTTTAGTTTGTTAGAAAAAAAAAATTGTCCACTTGCTATAAATAAAAATTATAATCACCACGTTACCTAGTTTACTTCTAAATATTTCGATATATAATAAATTTGCATTTAATTGATTCATTAAAGGTATAGAGGTAAAATGACATTTCATTAAGTCGAATTTGCATTTATTATACGTATCATAAAATGCACAATATTAGTTTGTGAACACTCTTTTAGAGACGGAAGGATTATTTAAAGCATCCTTATTTCTATATTTAGCTGCAAAGTTTCATAACTAATTTAATGTGATCCATGTTGAAAGAGAGTTATGTTGTAAATTAAATCGACTTTGTTTGTCTCATATCTATACGACCTATAATACATTTTAACACTTCACTCTTAATTAATTTTAAGATTTCTATAAATATTTTTTAAGCATTACAAGTAAGAGATCAATGCTTATAATTATTTAAGGCACTAACATATCAATTCAACACATACAATTAAGGGTCCATTTGGTTTATAAGTGAGTTTGGATTAGGATTTGGATTTGGTATATGATTTGGATTTGGGGTATGTTTAGGAGAGAGAAATTATATTTATTTAAATATAATTTATAATAATTTTATATAAAAATATCATTTGAATAGTACTATGTTAGAGAGAACAAGATTTGAAAAAAGAAAAAAAAATGACAGGACAATATAATTATGGTGGAGTGAGAAGAAAGATACGGAGTACCAATTTTACATGGAATGAGTTGTATGAGTTTTCTGACAAAAATTTACTATCAATCACCCCTAACCAAACGGGCCCTAACACTTTTCTTAAATCACCCCCTCACTCAAAAGTACAACCATTTTACCACATAGTACAATCTAAATCCAAGCCCTTAAAAAAATTTAACAATATATTTGGTTATGTCCAAACTTTTAAAATAAGAAAGAAAGAAAAAAAAAAGTTGAAAGTGACCTTAGATAAAATATTATCAATGGCCTCAGAATTTCAACGATTTATCTTTACTGTAAAAAGATTCTCGCTAACTCTAAACATATTGTCACCGTAAAATAAAATAATAAAAAATTATTATTCGTCAAAAATGTCAATTACATGGTAACCGCCGCCGCAACGCGCGGGTACCATGCTAGCAGATGTAATTTAGTAAGACAATGATATATAAAGATTGTATTGTTTTTGTTTTTGTGGATATCATGTATATTTTTTGAGAATTTCAAACGTTTGATTTATAACTTTCTTCGATTGTAGATGGATAAAGCTTCGATAGTTGGGGATGCAACACGATACATTCAAGATCTTCAAACACAAGCAAGGAATTTAAGGTCAGAGATTGCAAAAATTGAAGTGAATAGTAACCATAAAAAGGCTTCACAAAATTCGAAGATGGCCAATGTATCAAACTCCCTCCCAATATTTAAGAAGATATCAAAGGTTAGACAAAAAAAATAAATTGTCGTTTAAACATCTACGAAATCACATGCATCCGGTTTTTACTTGTTTCTTTTTTGGAGGATTTAGTTAGAAATGTTTCATGTGGAAGAAAAAGGATACTATGTGAAAGTAGTTTGCAACAAAGGCCGAGGTGTTGCGGTGGCCCTTCTTAAAGCACTTGAGTCGATCACAAGCTTTCAGGTTCAGAGCTCGAATTTGGCAACCCTTGGTGACACTTTTGAGTTAACATTCACGTTAAAAGTAAGTTCAACTTTGTAACTCCATATAATAGTCTTTTCAGTTAACTTTGATGAGTAATTATCCGTGTGTGAATATATAATATACAGGTTGCTGCACGCGAATTCGATAAAAAACCACCAAATTTGAAGCTACGGCTTTCTGGGGCTTTCCTTAAACAGGGTTTCAAATTTAAGTAAATCCTATCATCTTAAATCATTGACGAAATGTTGAAATTGTTCATATGCATTTAAGTAAGATCAACTGTAAACACTAACACGCAAGGATGTGCATCACCGATTATTTTGTTTTCATGCATGTTTCATTAACGGCATGTAATTACCTTTGTTAACTAGATAGTGATGGTTTCATGTGTAATAAAGAAAACTTCCTTTGTCTTATGAAGATTAAAATTTCCAACTTAACGAAAAACCTTCTTCTAAACGTCAAAAATGCATACACCGGCTTATAATTTGTGTTTCTAACAGGTAGCAGAACCTTAAACAATATGTTGAGTTGCAATCGTGTAAAAAGTTTGAGAAAAATCCACTTTCTACAAAGTTTAGAAAGGGTCTCATATTCTTAAACAATACGTTTTTTTTTCTCATCTTGCGCTTTCTTCTCCCATTCTTAGCTCGCCCTTTCTTTCTACTTTTCTAGCAACACTTTTTTTTTCCTTCAATTAGATACTGTTCACAACATTTTTTTCCCCAAATTGAGTTTGAAATCATTTTAAATCACAAAACTAGGTTACGATTTTCATATACTTGCATCATAAATATGTTTAATCGCTACAAATTACAACAACTTTCATGAATTCGAAGTTCATACAAAATTTAGGTTTTGATGACTTTGAGTCAAAAATTTAGGCTTGTTATTTAATTTATTGTGTCCTTGAAATATGAAAGTGTGATGTTTTTAAAAA

AabHLH53

TGTCTATATCGTATTCTTCCAAAACTGTACATATTAATCCTCTCGACATGTTTTTTACAAAAATCAAAAAGCGATTTTAACATTTTGCACGTATTCAGAATATATATGCCTGCATACAGTGATGAGTCATGGATGCGACCAACCTGACAAGGTCTATAGACCCTAGTCTATCGTTTTCAGTGCAGACATACCTGTGGGTCAAACAACCTAGGCGCCATCCCACTAGATCTTTTTTTAATACTTTACAACATCTCCAAGATAATCAATACCTCATCATTTACCATATAATTGCACAATGCCACAATATATTATTTATTACATCAACATAATACTCAAACTCAAGTTCCCCATTCTATGCATTAGATTCTTGCTAAATTCATTTTGCTAGCTAGATGCAAATGCAGCAACCAAATAGTCAACCACAAGGTCTTCTTCAAGACCTTTTGACTCCAAACACACACACTTTGACCACATTTCCTAATGAACTACAATGTGATGATTCATTTTTAGCTTCTTCATCATCTTCATTTTTGGAGCTTATATCATCCTCTTTAGATCCCACTTTCACAAATCTTGAAAGCTTCACGCCGGTTAATGTTTTGGATAATGCTTGGTCATATGAAAAAGAAGTATTTGACTATGATTATGGATGTGAATATTATGACAAAAAACCTAGATCATTGGTTTTGGTTCAAGAAGATTATTCAACAATATTGGAACAAGTAGATGCTAAGGTTCATGAGGAACATCAAGTGGTTGTGTCGGATAATCAAATTCCTAATGTTTTCAATATTGGAGAAAAAAATAGTAAATCTAAGAATGTTCAAGGGCAACCATCTAAGAACCTAATGGCAGAAAGAAGGCGTAGAAAGCGGTTAAATGATCGTCTTTCCATGCTTAGATCAATTGTTCCTAGAATAAGCAAGGTGTGTTTCCTTTTTTAACTAACATAATTTATCATATACAACTTCTATTATTTTATACTAGTTTTCTTGAGGCATATTATAGATGGACAGAACATCTATACTTGGTGATACAATAGAGTATATGAAAGATCTAATGGAAAAGATCCGTAGCTTGAAAGAACAAGATACAGGATTGGATTCGAGTGGTCTGAATGAGTTAAAAGTGAATCAAACACAAGAAAGAAACTCCCCAAAGGTGATTTTTATTCTAACTATTTGAATTTATATAAACACACACACGCGCGATTTGTTCACAAGTAACACATACTAATTTCTTGAAAAATCTTTTGTAGTTTGAAGTAGAGAGGAGAAACATTGACACTCGTATACAGATTTGTTGTGCACCGAAGCCAGGATTGTTATTTTCTACAGTGAACACTATTGAGTCCTTAGGCCTAGACATTCGACAATGTGTTATTAGTTCTTTTGGTGATTTTACCCTTCACGCTTCGTGTTATGAGGTACATGATCTTATTTATTTTACACGTGTGTGTACTTCTATCAAGCGTTTTGTGATAATATAAACTTCGATTTTTAGGCACCGGAAAATCAAGTGCTTGCAAGTTCTGAGGAAATTAAGCAAATATTATTCAAAAATGCTGGTTATGGAGGTAGAGGTCTCTAAAGAAGTATTTTCGATAATTATAAGTAGTTAGAGGATGTTAATTGGCTTGGATATCTTTTGATCATTTTGTTGTTATTGATGTATTATCAAGGAATCATGTATTACATCTTTTTAGTGATCTACTTATGAACGTACACGAACTGCTCTAAAGTCTTGATTATCAACTTTCTTGATTAAAAAAGACAATGTTCCACTTTTAAATGAATTAAAGGAAGGTACAAAAGATGCAAAATTCAACAAAAGAATTAAATATGTAATTTACAAGACATGAGAATGTTTTGTGGCCCCTAGCCATGTAGAAAAAGAATGTTTTCAGTGTTATGGATCATT

AabHLH54

TATTTCCTTTCTTTAATTCATATGATACGGATACCTTCGATAGTAAACTTCAAGGTAAAATAGGTCAAAAAATATCTGAATATTTCTCACCATAAATTCATACTTTTATGATTTCACTTTGTATATATTATATATTCTATTATGACTAGATCATGAATCTCCTAATAACTTGTTGCAAAATTATTTTATTGCTCAGTTTTTTACTCCTACTAAAACTCATGCTTTTCAAAATTAACAAACTCACTGCTTAAACAGTGAACATAACTAAGTACTACCAGTTTTGTAGAATTTTCACTTCTTTACTGAAACTCTATTTATCCGGCCGAGAACCTTACTGAATAATTTAGTGTGGAGTTCTCATGCGTGTGTAGGTTTGTAGGATCTTACATTTGCAGGGGAGGTATAAGTGTTTGGATAGTCCTGGATAATTTTTTGAGCAAGTATTTTCACAGGGAAAGTGAAGTAACTAATCAAGAGTTATATATCAGTCAAAACCCAAATTCTGACAAAAATGTTTTGACGTTGTAGCCGACCAATCATTGATTATCTTGTGCATTGATCAATCAAGCAAATCTATACACACTTGTAATGGATATCCCATTTGGCGCTTGGTTGCCTGACCTGGTAAGAATCAGTGACCTTAATTTCCAAACCATAATGATTTTCATTATATTATTTTGTACGTTAATTAACTTTGACCTAACTAAGATAGGAAATGGAAGATCCTTGTTACTTTATGAATCAACATCAACATACATATCCGTATAGTGAGTTAGTAGATTCATTTTCTTCCCAAGGCTTCAAAGGTTGCATGAATTTGGTCACAAGAAGTGAAGCTATTCACGCAGCAACCATTAGCAAAGAATTAGACAGTCAAGAAATTTATAAGGCAACTAGAAATAACTCGTTCACTCCTATTGGCGGATCTTCATCCAACACATTCACTATATCCTTTGGAGACCACAGTTCATCATCAGAGATTAACCAAACTACATTACCTGGAGGATTCAAGTTAAAATACGATGATGTAATGCAACCTAAATTAGAGATGAGTCTCAATGAACTTCTTGGATCCATAGAGCTCCCTAAAAGAGCTTCCACCACCAGAAGAAACCATAGACAAGCCCAGGAGCATGTCTTGGCTGAGAGGAAAAGAAGGGAGAAGTTAACTCGACGTTTCATTTCCTTGTCTGCTCTCCTTCCTGAAATAAAAAAGGTACTTATAAATATATCATCGAACTTTTTGGCTCGAAATGATCATATTTTATGTTTATCTGCGTGTAGGGAAGGACCTACGATTTTCGGAAGTGTATTGCACAATTTTTCTTTATATCATAAGTACGAAACTTGCATCATAAAGAATTCTTTTAGCTAAACTAAAGTTGCATCATGAAGAAAGAAAGTAGCACAAAAATAATAGCGGAAGTTTAACGTACAAAAATAAATAGACTTTTGGAAAAATTATACACTTAGCGGCCTAAAAGTGGGCGTGGCCTATATTTAAAACATCCTAGTTGTAGAATTGATTCCATAACTATCAAATCAGAAACTAATACCTGTTTTTAATACAATTCATATCAATATTGAATTTATGTAAACACATATTAAGCTTGTATGATATAGTTTGCCCAATGTTATACATCTATTTGCCGTGTTCTATGCATGGCTAATTTTCTCGTATTAGTAAATATACTTTGATCCCATTGCCCATTTTTTTGGATTTAGGCTATATAGCAATAAATTCGTTTTGATGCCAAACCCCCTCATAAACACATTTTATGTGACGAGATCAAGACTTGTTGTAACATTCCTTTGCATCATTTTATCGTATTAGTTCTTGCTTAAGGAAGGATCGTCTCAAACTAATATCTGAAATGATCTGTTATCATAGATGGACAAGGCTACAGTGCTGGAAGATGCAAGTAAGTACATCAAATACCTACAAAACCGAGTGAAGGAACTCGAAGAAACATCAGTTAGCGGAAAAAATGTCATCCAGGAATCAACAACTTCAATGAGGAGCAAGTTTTATGGTGGTCATGAAGACAATGCATCTTCTTTTGATGATACTAATTCTTTACCTATTAGCACCGCTAATGATCTTGGGATTAAAGTAAAAATATCAGGAAGCCACACACTTGTAAGAATTTACTGTCAACGAAATTCTTCTCTTGCATTACGAGCCATCACTGAAATGGAGAGCCTCCATTTCAGCATCATGTGTAACAACGTTCTTCCCATCTCTGGAAATACTGCTCTAATAACCATAATTGCTCAGGTATATATATGTTAGTACGGGAAAATCAACAGACTGCATTTAAATTCGAAAAATAGTTTATGATGAACAAACTTCCAACAAATTGTTGAGACTACGCATATTGTTTTAGTAACTGACATAACCACATATTAATTATGTAATTTAAGTCGCACGATCCAACGTTTTTACCCACATTTTATCCAATATCTTTTCTCAAAAGGTCCAAACGTATTCTGAAACTTTTTTAGTGTCACTATAGGCCATTGCAACAAACACTCTTGCTAGACTCAGTTTATACGAATAGGTGAAACGGCCAGATTGCTTTTATAATTATTTTTATGACTTGGACTTTTTTAGTGTCACTATAAATGACACTTTTCTTTTAGTGATATTTCTAATGACATTGTTCATTTTTCCTTGCTATGTGTAGATGAATGAAGAGATCGAAATGACAGCCATGAATCTTGTAAATTGTTTACAATCATCTCTTTCAAACTTTCTATGACGTGCATAGTACCTGATCTGTAGATCGATGAGTGAAAATTTTGTCATGACGGCCATGGATCTTATAAATTTTTGGAAAACGAGTCTGTTTGCCTGGCATTTTCTGTTTCTGCCCAGAGAATTCTTGAAGGAATTCGGATGCGTTCATTCATTTGACGAAAGTCGTGTCCCAGAGTGTTGTTTAGATTGATCCGTATGGTTCTTGTTCATTACCGGGATAGCTTTTTCCTGTGTAACTTTTCTTTCTAGCTACTTCTAATAAATTGTTGCTTTTTTACTAGATTGTACGTGATATGTATAGTAGTTCTAATGGTGTAGTCTTACAACTAGCTCTTTAAAGTTATATTTGTATCGACATCGATTTGTTATTTGCATGATAGTGTCCGAACCAATAAAATATTATTATTTTAATAATTTAAATTGAAAAATACCTTTAGTGACGACTTATGTCATCGCTAAAAGTCAGTGAACCCCACGTTGGTCAAGCATTAAAGTTGAATTCATGTGGGCCCGCATGTCATCACTAAAGGCATAGACCTTTCACCGATAAAAGTTTTAGCGATGTGGTTAGGCGACGACGTTAGCACGACATGTCGTCGCCAAAGTGTTTAGCGACGATGGAAGTATACTTTTTGCAACGAATCCTTCTTCGCTAAAAGTTGAAATTCTTGTAGTGCTAGTGATAAGTCCCGTGCACACACATAGTTTACACATGAATTAAATTTATTTTAACCTTTTAGTTTAATAAACGAACATATAAGAATTTGACACTGTTTAAT

AabHLH55

TTTTTTTTTTTGGATAAAAAAGTAAATGATCAAGATTATTCATTAAATCTATGACAAATATAAGATCCATATTGAACTTTTACCATTAATAAAGATGAGTGACTCTTTTCTTTATAACTCATCTTTTTAACCTTTTCACATTTTATATAACAAAATCACATACGCTCTCCGTCTCAAAATTATTGTCAGGAGACAAGAATCACACAGTTTAATAAAAGTGGAACCACTATAATTCTCAACCACTCAAATAGAATATTCCCATACTAAGATCTTTCTTACTTAAAATGGAAAATGGATAATTATTTTGAGACATCCCAAAAAGAAAATATGGACACTAAATTCGAGACGGAGGGAGTATCTCTTATACTACTAAACTGTGCCTCACTCACGTTTTAAAACATAACATTCACAACTGTAAAGTATATTATGTATAGAAACAAAAAAAATTATGATGATTATTCAATGTATTTTGTGCTCTACGTAACTTAAAAAGAAAAAATAAATATAATTCACAATATCTTTTTGAACACACCTTTATTTTCTATATATTCTACAAATGCTTGAAGGGGCAGGCCAAGAAGGATATTTTTGGGATTCCTATTTGTGGACTATGTCGAACAAATCTGGTTCTGGTTCTGGTTCTGGCTCTCGCTATGGTGAAGGTCGTGATGAAGAAAAAAGTTTAGGCAATAAGAAATTAGTTGAAATAGACGTTGGTAATAGTCGTAGCTCAATGAAGCAGTCTGAGATGGGAGGTATAAAAGGTTCGGGTAGTGGTGAGCAAGAAGGGGTAGCTATAACTGTCACTAGTGGTAGCAAGAGGTCTAGTGATGCTTTTGAAGACGACGTCAAGATTAATCGTCCTCTTAGTGATCATGATTTGCATATATTGACAGAGAGGGAGCGAAGAAAGAAAATGAGGAATATGTTTCATCAGCTTCATGCCTTAGTACCTCATCTCCCTCATAAGGTACCAATTCTATGTCATATATATCAACTATTTCATCATTTAATTTACTATATGTAGAAATGAAATGAAGCTTCGAAAAAATTATGTTTTATTAGCTTATCACAAATAAATTTTTGTAGATCGATCATATATATGTAGTTGCACATCTACGGATATACAATAAACTTGAGTACTTTTTGTTGTTGTTGTTGTTGTTGTTGTTGTTGTTGTTGCTGTTGCTGTTGCTGTTGCTGTTGCTGCTAAAAAAGTTTCAAAGATTCAAACGTCTTGAAAAAGAAAGATAACTACAAACTTACTCGGTTCTTTTGAAATTAATGGGTTCTTTGGTTTAATGTGATATGCTGATTTGGCATCCCAATGATTTATGATACATATGTGACACATATAAGATTTCTATTTTAAGAGTTTTTAGCCTGTACATGTTGATGTTATTCGAATAAAATTTCAAAAATTGAAGTCATGTCGTATGTGTACATTAGTTCCCCGGAAAGGATTTGATGAAAAATATGTTGCATTTAATCAAATCAGTTTTATTCTCCGATGAGATTTAGATTTTCTAAATAAGAACTAGTTGTAATTGATTATCATTTTATAATTAAATAAAAATCATTTTAGTTTTTCAAATTAATGCAAAAACCATAAATTTATTTCTTCACATAAACATCCAAATAATTGCAATGTCTTTTGTTAGATGATCTAACATATTAAATTTATCACAATACTTTAGTTTGTAATAAAGTTTTTTTCTGAATCATTTTGTATCTGTCTATGTTCTAAAAAGGTTATTTTTGTCCTTATCTGGTAGTATTTGCTTTCTATGCTATCATTAATACGTTCAAGTATATTATTTTTAGTAATGTTTCAATGTGAAAAGAAACATCCTCATAGTGGAATAACATTAGGTATTAATGAATGTATCATTGTAGAAGCCTATTGTAATAATGTCGGCTTGTTTTGAGCATTTGTAACTTGTTTTTAGCTTCTTATTAGCATCAATGAAAGTTGTCGCTTATAAAAATTTATAGTAGGTAAAAGTTTCTGTTGATACAAGATCAGTATTTTCTTTCATTCTTTTTTTAAACCACTATATATTTTTTTGACTTCTTACACTAAAAAACAATACTCCAGTTACTAGTCTGGATCATTGATAATAGATCCAATCATGGAGTAATTCAAGAACTATATATGATAAAGCATCTTTTAAGCAACTTCTTATTAACTTGATTGGTATATGTCACTCTATTTAAAATAACTATTTATTCCAGACAGATAAGTCTACCATAGTTGATGAGGCTATAAGCTACATTCAAACCTTAGAAGAAACCCTCCAAAAGATTGAAACAAAGAAGCTAGAGAAGCTTTATGGTACTCAGTCTGCAGCCAACTCGACCACTGTTTCTCCAATTCAATCACCAAAACCAGCTTTGGACACTAGGGAATCCTTTTTAGCCGATCAAGGATCCTCAACAGTCTCGCCTTCTAGTTCAAGCACATTTTCCTTTCCAATATCTTCACCAACGGTTTTCCAAACATGGGCTTCTCCAAATGTCACTTTAAATGTGTGCGGCATGGATGCATTTTTTAACATTTGCTCCTTCCCGAAGCGTGGGCTATTTACTGCAATTTGCTTTGTGTTGGAGAAAAACAAGGTGGAGATGGTTTCTTCTGAAATTTATTCAGATCAATGTAAATGCTCATTCTTTATTCATGCCCATGTAAGTATGTCTTTTAATGGTATCGTCTTGCATTTTTTTTCTGTTGAATGTCCCATGTCATCTTTTTATACTTATCACTAGTAAATATATTTTTTAAATTCTATCACTGCAATATTAATTAGGTGAACGCTCGTGATCAAACTGTGAAGGATTTGTCTTTTGAAGAGATATACAAGCAGGCTGCAATGGAGATAATGCGTTGTGTTAGCACAAAATCGCCATAGATATCTGAAAGGTTTTGCCATCATCAGTTATGTTAGCAGGGCACATGAGATGGACTTTGTTCCAAGGCATGATTTTCTTTGTGGAAGTTGTTTGCATTTCGTTATTGTTTTTGTAAGCTTGAACCTTTAATGGCTTCTTGGAAGTAGACATCTCTATATTTCTAGACTTTAAGTTAATATGTTTCAGTTACAATTGAAAACTCATTTTATTTTCTCATTAATTCTTCAAAATGTCTTATATTGTGGAAGTTGTTGATTGCCAACAACCTTGAGGTTTATATTTTTAAACTTGTCTCATACTCTTTATTACTCCCATGTCCAATAGTAAATGTCCAATTACAAAATACACATACATTTAAAAGGTTTAACTAACTAAACTTCCACCAATTAGATATCATCTCTACCCGTTGAAGCTCATTTTGTTTGGTTAAAATATAAAGTTGACACTTATTTTGAGATCATAAATTAACAATATAGAGAGTTAATATGGATGAATGAAGTATATAATCAAAGCGGTTAAAAAATGGGTTGTTATATGTACACCCCTAAAAGTGAGGGTCAAAGTACAAAAGATTCTTTTAATCCCACGGTTTAACAGTATAACAGAGTTTTTATCAGTTTCTGCTCCGTATTTTTTGTAAGTTTAATACTTTTGACTTGTTAAACTTTATATTTTTTGTTCTGTACTTTCAGGTTTATAGTTTTTTTTAATATAAACAAAATTATAAAAATAATAAAAAATAAAAAAGGGGAAAATCCAGAGGGGGTACAACTTGTAAAAATAGGAGGGTCCTTATAACCTAACCAAAAAAATACATGAGACAATGTAATAACAGGAATAATAGTACTATCTTACAACAATCTTAATAAT

AabHLH56

CATTTGATAATCTGACATATATAAAGTTACCAACATAAACAAACATTTAAGTAGAAAACGAATCCTGAAACCTGTAAAAAAAAATGTACTACGTCTCTAGTGAGTTAAGAGAGACCCATTTCCCAACACAATTCACATGCGAGTGTGTGCATCTGACTTATCTCACCTGGCACTTGGCACAAAAATTTCTTTCTAAAAAAGCTGACCAAATAAACCCATCTTTGATTTATCATCATCTTCATTTCAACGGTGGCTATCTACCTCTTTTAATTTACATCTCCCTATTTTTAGCTTACCACTTTCTATTTTTACACAAACCCTTAAACTGTACTCCACTATGATATCTCCATTCCACAAATCAAAGTCAATATCAGTTTGTTAGGGTTCAGCTCAGATTTCTATTTTTAGAAACCCTTTCATGGATTCAGATCTTCATCATCAACTCAACCATCTTCCCCAACAACAAACCATGAACACTCCAGGGTTAACAAGATACAGATCTGCTCCTAGTTCATACTTTTCAAACCTGATCAACAGTGGAATATATGATGACACTGATCAGTTTTTCAATACTAGGGTTTCCTCAAGTAGTACTACTGATCAGATATTGTCAGGTTATGAGTCTAGGTTTATGATGGATTCTGTTAAGCAAGAACAAGAAGTTATATATTCAACACCACAACCAAGTACAATGAATTATCAACAACAACAACAACAGCAGCAACAGAATCAGTTTCAGTCACAAATGGATCAGCAGACTAATAATAGTAATACCTCAGGGATGGATAGTTCGAATAATATAAGGAGTAATAATTTGCTAAGGCAAAGTAGCTCACCTGCCGGGTTTTTCGATCAGCTCGATATGGATAATGGTAGCTTTCTATATCTTTTAGCATATTTTGTTCAGCTTATTTTTCATAAACTTGTTTTTTTGGCTTTTTTTTAGTTTATAGTCTTAGATTCATAGTAAGCTTAGGGGGTGATTGTTTCGGCTTATATAGCCGACTTATGGCTTATTTGGTTTTGGGCTTATGTTTCTTGAGCTCATTTTTTGTGGGTTAATTTGAAACTTTTCAAATTAGCAATAAAAAGCTAGTGAGCTAACGAAACATAAGCCCAAAACCAAATAAGCCATAAGCCGGCTATATAAGCCGAAACAGACACCCCCTTAGTAGTACTGAATGGCTTATAGCCTAGCTGGTACCCATGAGATGTTATGTATGGTATTTGTAAATATTTTGAGATTGTTAGTCTGCCTCGGTAGTAAGCGAAAAAAACCCGTAAAAATGGGCTTATCTCAAATTAAAGCAGAACAAGATAAGGTAAAACTAACACCATTAATGATTTTGGTAAACAGGTAGGGAGCTGCTTAAATTTTATAGTATGAAAAATGGGCTTATGAAAAATAAGTGTAACCAGTGTATGATCTGTAGATTTATTTCTAATTTAGGCAAGAAGGACAGTTAATTTGCTAGATTTCATGTTCTTTTGGAATTGTACTATTTGGGGTAATTCTTGTGTTTGGTATTAATTGTACTTTTAAAAGAGTTGATATCTGTGTATGGTGTACAAGTACAAATGCTTAAAAATGATTATCATGATGCTGATGCTTATATGTGTCTTTTTGGATAATGGGATGATGGTGAAGACGGTTTAATTAACGTTTTCTTTCTTGATTTGGAATGTTTATTGCTGCAAGTTGAGCGTTGTTTATAATTAAATTTGACTATGTATGCTGTTTGTCTGTAATTAAACCCAACTTGTCACTTGTTTGTTCTTAATTAACAATTTGCGTTACTTTGTTGAGGTTGAAACTTGGAACTAATTGTTTGTAGGTTTTTCTGCGATGAGATCGTTGGATGATTTCAAAGTTGGTGGTAGGGGTATTGGAAACTCAATGCTCTCCTCGACAAAGAGAATGAAAAATGAAATGGGATTTACGTCTAGTTCACTAACGTCTTCTGGTATATTGCCGCGTATCCCTGAGAATGAGAGCAAAGTTATGGATATAAAAAGTGGTCGTGATGATGGAGGCTTTGGTTCACAATCATGGGATGATTCGGATATCTTATCTGATAGTTTCTTGAAAGACTTTGGAGAAATTGATCAAAGTAAGATATCGAGTTTAAAACCATCTGAAAATCAGGTATGCTATTTGACACTGATCTGATCTTCCTAGTGTTGAGATTAATGATTGTTTTAACCAAAATTAATATGACCATTACTGGTTGTGCTCGGCAGAATGATGTTGGGAGAATACGTGCTCCCAATACGCTTGTGCATCACATGAGCTTGCCATCAAGTACAGCCGAGTTGGATAAACTGCTGCAATTTCAAGATTCTGTGCCTTTAAGAAGCAGAGCAAAAAGGGGTTGTGCAACTCACCCAAGAAGCATCGCTGAGAGGGTAAAAAACCTTGCAAAACTACTAATTTTTACCACTCGTTAACTTTTGTGTCAGAAATTGATCAAGATACTTCCTTTCGGATGCTTTCAGGTAAGACGGACCAGAATAAGTGAAAGAATGAGGAAGCTACAAGAGCTTGTCCCAAATATGGACAAGGTAAGATTTATATTTTCCTAACTTACAACTCCAAAATCTTAAATCTCATATACATTGAATTAAGGCCATCATATTCGACACTTGCAGCAAACAAACACAGCAGACATGTTGGATTTAGCCGTTGACTATATTAAAGAACTTCAGAAAGAAGCCGAGGTATGTGATGAAAAAAGAATTTCAAGTCCAACGCTATAGATTTCCCTTGAAGATAACTAATTATGTGTTATTGTTGGAACATTTCAGGCTCTTTCAGACCATCACGCAAAATGTACATGTCCACATAAAGAAAAGCTGTAACACGAGTTTAAGCATAGGTATGTATCACTTATGTACATGAAGGAAGTGACGGTTGAATTATTTTCGGGTGATTAGAAATTTTCAAAATAAGACTATGCACCTGTATTGGTGTATATAAATGGAACAATAAACTTCCATTGTTGTTGTAACTATAGCTTACTGTATAGTGTTACTCGACTACATTAAGCTTGTCCATGGTCTCAAATTCCCCTGCTCTACTTTTCGGCTAAATTTAGGTTCATTCTTGTACCTTGTATGATATTTATTGTTGTAGTTGTATCGTGTTATCTTAATCATTCGAGGCGTTAAATCATTCAAATTTGCTGATAGATATACTGTTTTTTGTTTTTTGTTTTTTTTCGTTTTTAATGTGTTTTGTGAAAATGAAAAGTGTGTCTTTTTGGAATATTGATGCATGTTGGAAGTATGAGTGTAAAGGTACGGACTATTTTCTTAGTTCATCAAAACAGATTTGGTGAATATATGGACCACTTCTCAACTATTATAAAATTAAGCATGGAAAAGAAAGAAAGCTACAACATAAATAATTTATGAATCTAGTATTTTCGATCCTCTAGCTTTATCTTATATTCTTATATGAGTTGGTGCTCTTTCAGCCAC

AabHLH57

TATGATAGTGAATTAGTGATATCATAACTTAGCTTTGCAAAAACAAGTGCAAATTTCATTAGTTTAACATGTCTATATTCTAGAAGAAAACATACTCCAGGGTGTGTGTAAAGTGTGCCATACCGCATGTATCCTAAACTAGAATAGTAGTTACTTGCGCAAGACACGCTTTTGGGATTGGCTTCAAATTTTGTGAGATTATTCTTTTATTTAGGTTAATAGTATTGTCTGAACTCATTAGCTAGCAAGAATCTTTGAATTAGTCGTTGATGAAGTACTAGCTATTAATAATTCTAATGAAGTTATCTATCCTAAAGTTGAGGAACTAGTTTTGGATAAATAAAAAGTTCTAAAACCATTTTTAGATGTTTAATATACCAAGATGATGCAAATAAATGTCACTTGTATAATGCAAGAGGATACCCCTGTCACCATTGCAATATTGTATAAATACACCAACACAGCATCCTCACTCATATGTTCATTAAACGTTTAACAAGTATTCCCTCTCTTTTTTTCTTTTTTTTTTAAATAAAGAATAAAAATAAAACCAGGAAGAATGCAAGCATTATCTCCTACATTGTTTTCCACAACATATGGATGGCCTTCGGAGAATAACATCACCCAAAATCACCAACAAGATTTCAATGATATTTATATGGACGTTGAAGCAAATTCGTATAGTCCCCTTCTTGATATTCTATCATGTGATCAAAGCCAGCAAAATTGTGCACCCAATAGTTGTTCTTCTAGAGGAGCTATAGATGGAAATACTGCGGATCCCATGAAGGTGTCCAAAAAGCTTAATCATAACGCAAGTGAAAGAGATCGGCGTAAGAGGGTTAATGATTTATATGCATATCTTCGTTCGTTGCTGCCCATATCAGCTGATCAAAAGGTACTTGAATTTAGGTAGTTACATTTAGTTTTTCAGTAAGTCAATAAACAGAACAAAGAAAACCTCTCAAGTACTATATGAAGAGTTGATTGACATTGAATTGGTTTTTTCCTATTTCTCGTTCCTTATAAACTCAATTTTGTGACAGAAAAAAGTAAGCATTCCTCGGACAGTATCACGTGCACTGATATACATACCAGAACTACGAAAGGAAGTGGAAACATTAATACGTAAAAAGGAAACTCTATCATCGCACTCATTATCAACCACGAGTACAAGCCCAAAGAATCTCGGCATCAAGAGACAATGTGATAAAGATGTTAACATTAATATGAACTCATCGGTGGTTTCTTCTGTGAGCATTTTAGGTGAGAAAGAAGTGGTGATACAGTTGATTTCCTCTACAGATCAAATGAGCAAGAATAAGGATATTGGCTATTTGTCTAGGGTTTTGGCATACTTAGAGGCCGAAGAAAATGGATTTGTTTTGCTAAATTCAACGACTTTCAAATGTTCGGGAGATGAAATGTTATTAAACACTATACATCTTCAGGTACATGTCAATACATCGAAAATACATATAAAATCCATAATGCATGTCAATTAGTTTTACTTGATTATACTTTAGGATTTCATATTCACAACAAATTTATTTCTACATTATCCTCACATAGAGACACCAAATTTCTCATTAAGAAATATAAGTGTAGTATGCTAAAACATTACATACAAAGTTTCCATGACAATATTACTAAGTATCGCAAAAATAGATAAGGTTACGAAAAAATTAACTATTTTTTTTCTTCATTTTTATGTTACGAAACAATTATGGGAACACTCCTAAATCTTGCACTAAAAAGAGGGGGGGAAAATCTTATTCCCCAAGATTCTAAGAGTCGAATTTTAGTATTCTTCTTTCAGGAAAAACAGAGTGAGGCAATGAGCAAATATTAATATTCACTAAAATAACTAAATATTGTTCGGATATAATTGCAATGCCATATTGACCTAAAATGTCACATTTTGTTCAACAGGTGCAAGGTGATAATTATAAAGTAGAAGCTGAAATATTGAAGGAGAAGCTCTGCCGTTTGAGCCAACAAACAAATGAGCTTTCACCCTAATATATGGACATGGACGTCATTAGTTGTATATATAAGTAGATCATCGTGGCTTTAAGGCACGTCCATTTTTTGATAGCAAGAAAATTTCGTACCATTGTGTTGTGCTTCGTATCCCTACAAGAGTTTAGAACTTTATCATCCGTGCATGCAAACCAAATATCTGCAAAGTCCTTTGTACTTCATAGTAGATTCATGCTTAAAATGCCTTTGCATAATATATTCTCAGTGCTTAGAGTTTAGACAATGGCATGATATGAATCTATTTATCACAATATGTTTGTTTCGTCACTAGTAGAATCCCGACTCTATTCATCTACTCAGCTGTCCTCCCAACATGAATGCCAATTGAAGCAAAAAGTTGTAGATTGACGTGTTATCAAATTCACCTATGTGGTCGCATTGGAACGATTTAATTTTTCATTAAAATTGGGTTTTAACACATGCATGAAAATGAATAAACTTCGTAAAAGCATCAACCCTTGGCATAACTTGCCAGAGCTCGAGCTCGGCTCGTTTATATATATAAAGCTTGAGCTCGGGCTCGAGCTCGTTTCGAGC

AabHLH58

ATGTCTAATGGTGCCAGTGGTGGAGCTAGCTTGAGTTCTCATGAGAATTCAAGTCAAATTCGAGTAAAAGAATCGCGGTTTCTTGATCATATGAAACATGGACAACCTAGTTTTACAACTCAGCAACATGTGATGTATCAAAACCATGCTCAGTCTAAGCAAGGTGTTCCTAGCTCTAGCTCGGGTTTTTCGCCAATGAGTAACAATCTTATTCGTCAAAGTAGTTCTCCAGCTGGATTTTTTGAATATGTTAATATAGATGATGGTTACTCTATGATGAGAGCGATCGATAAGTATCGACATGCTAATGGGAGTGTTCAGGATTCACAGATGGCTAAAAGCAGGATGGTGTTTTCATCAGGTTCTCATTCGCTGTCTCGTATACCTGAAAATGAAGGGAAAGGGATAGCGAATAGTAATGGTGGTTATGTTGATAGCTTTGGTGGTGGTTCTTGGGATGCATCAGTTATGTTAAATGACGGGTTCATGAAAGAATTTGGAGAGAGTGATGGAATTAGCTCATTTGATAATCAGAATGACGAAGGGCAGATTCGGGTTTCTAATGGTTTAAGTCATCAGTTGAGTTTGCCAACGAGTTCGACTGAACTGTCTGAAATGGAAAAACTACTGCAGCTTCAAGATAACGTGCCTTTAAGAAGCAGGGCTAAAAGGGGTTGTGCTACTCATCCAAGAAGCATTGCTGAAAGGGTAAGACGAACACGAATAAGTGAAAGAATGAGGAAGCTACAAGATCTTGTCCCAAACATGGATAAGCAAGCAAACACAGCAGACATGTTGGATTTGGCAGTAGACTATATTAAAGAGCTTCAGAAACTATCTGAGAAACTTTCAGACCATCATGCAAAGTGTATGTGTCCACATAAGCGGAAACTATAA

ATTATATAAAGAATTATCCGAGATATAATAATAGTCACAGAAAGTGGCAATATGTTGAATAGTGGACAACAATATGAAAGCAACATTTCTTTCTTCCAATTGGCTGCCTGCAAAGCGCACTCACAAACACACGCCTACAAAACAGGAAAATAAAGTGTACAATCAAAAAATGGCTGCCTTCTTCAATCAACCCCTGCCTCTCATTCCACTTTCAACTACTTATTATCCACCCTATTTAATTCATTCCCCATTCATCTTCTCATTTTGCATCATTTTATTTTGTTTCATCATTTTTTTAGTACACTTTTCGCAAAACGCGACAAAATAAATATTCATACTTCTTTTGTTTCAAAATAAATGTTCACTTTGTTATCAGGTTTGTCACGGGTCACCTATAACATAATTTACATTAGTTAACGGTTAAAATTTTTACTTATTAAAACTGTGTGTCACTATCAAAGTGAATAACGTGAAGTTAATATACTTCTTTAATAAAAAATTTAAAAGCTCCTTGTCATATAGTACCTTATTACAAACCAAGATTTCTTAATAATCACTTCTTTTTTAAAAACATTTTCAAGTCTAATGGATTCAGATCTTCATCATATTCATAATCCTCAACAACAACAACAAAACATGAACTCAGGTTTAACAAGATACAGATCAGCACCAAGTTCATATTTCTCAAACTTGATCAACAATGTTAGCAACATCTTTGAAGTTGAAGAAAATGGGCAGTTTTTTAACACCAAGCTTTTGAGCTCCGAAACCGGTAGAATTCAATCCAGATTTATGTCTAATGGTGCCAGTGGTGGAGCTAGCTTGAGTTCTCATGAGAATTCAAGTCAAATTCGAGTAAAAGAATCGCGGTTTCTTGATCATATGAAACATGGACAACCTAGTTTTACAACTCAGCAACATGTGATGTATCAAAACCATGCTCAGTCTAAGCAAGGTGTTCCTAGCTCTAGCTCGGGTTTTTCGCCAATGAGTAACAATCTTATTCGTCAAAGTAGTTCTCCAGCTGGATTTTTTGAATATGTTAATATAGATGATGGTACTTTTTAAAATTTTAATATGTGGTATGATTAATGATGTTGTTTTTTAGATTTTGTGTTGATGTTTATGGTATTTGGTTTCTTAAGGTTACTCTATGATGAGAGCGATCGATAAGTATCGACATGCTAATGGGAGTGTTCAGGATTCACAGATGGCTAAAAGCAGGATGGTGTTTTCATCAGGTTCTCATTCGCTGTCTCGTATACCTGAAAATGAAGGGAAAGGGATAGCGAATAGTAATGGTGGTTATGTTGATAGCTTTGGTGGTGGTTCTTGGGATGCATCAGTTATGTTAAATGACGGGTTCATGAAAGAATTTGGAGAGAGTGATGGAATTAGCTCATTTGATAATCAGGTAAAATGCGTGATGATGGTTGATTAGTTAGTAGCAATTGATTTGAGTAATGGCTTAATGTTAGCTATATTGATGAACAGAATGACGAAGGGCAGATTCGGGTTTCTAATGGTTTAAGTCATCAGTTGAGTTTGCCAACGAGTTCGACTGAACTGTCTGAAATGGAAAAACTACTGCAGCTTCAAGATAACGTGCCTTTAAGAAGCAGGGCTAAAAGGGGTTGTGCTACTCATCCAAGAAGCATTGCTGAAAGGGTAAATATTTTTAACCACCAAATGACCCGTTTTGCAGTTTTATGCTTATACCAGTTTTTTGAAGCTTCTGCAGACGAGGATTATAAAAATACTATCTATTTTACGTATTTAGGTAAGACGAACACGAATAAGTGAAAGAATGAGGAAGCTACAAGATCTTGTCCCAAACATGGATAAGGTAAGATCATTGTTTATTCAGTTCTCGCATTTTAATGTTTACTCAGAATGTCGTAAAAACAGTAAGTGTGTATATTTGATATATGCAGCAAGCAAACACAGCAGACATGTTGGATTTGGCAGTAGACTATATTAAAGAGCTTCAGAAACTATCTGAGGTATGCCTTTCTGAAAAATAGGTTATCAAACTATTACGGTCCTGTATTATATTGCTTAATTAGTCTTACTGTTGTAACATTTCAGAAACTTTCAGACCATCATGCAAAGTGTATGTGTCCACATAAGCGGAAACTATAACACAAGTTTAAGCTATATAGGTATGTCTCGCTTTTATACATGACATATAAGTGGAAGTAGTCGAGACTTGTTTTAGTAAAGAGTTAGTTCAGAATGCAGAATATGCAAACTTCATTTGTTGTTTAACATTGAGCCAATTGTATAGTATTCTAAAGTAAATAAGATCGAAGTGCAAGCTCATTGTTACACCACCATGTATTTCCTTGGCATAGTGTCAGATATGTAATCTTTATTCGTTCTGTTCTGCTTGTATCAATGTAAATATTGTTCCTAGTAAGAGATGAAAAACTTATGTTTAGTGGATGGTGTTCATAGTTTAAACTTTTGGTGCCAATTGGTTAAGCATTGTTCTTGGATCTTGAAAGCACAAGATAACGATGCTGATAATGAGGTTTTAACAAGAGATAATGGAAGCATTAATTAGTGCGAAAAGTCTCCACTTATCCTCGATTTAGAGTTTCGTTTTTTTGTCAATATGTGGCTTAATTTCTACTTTTAGTAAGAAATCCGTTGGTATGGTCCATTATCGTATTATAGGAAAGGTAAGTTCCCTGTCCT

AabHLH59

GCATATCTTTGGTTCGTCAGTTAACAACTTTTCACATAACTATCTAGCTATGAAGCTAGCTGAGTTCAAAGTGTGTTCGTTTAACGACAGTGTGTTTTCTGGTACAATCTGTCACATATTTAGCAATTTAGATGACAATTATGATCAGAGTACTCTACACTTAACTGTAATCATTGTATGTATGAGCCATATCAAATTATCAATATTTTTACAAAAAAGATTATGTTAATGCTTGAAAGTTATAAACGCGGCCGCTTAAAATGACCGTGATGTTTTGTGGCAGGTATATCAAGCTTCAAAAACATGTTTTAGCTAGATGTTTAAAATATATTTGATTAATTATAAATTACCCTATTAAAATATATGTTTCATCAATATTTTAGAAAGGCCTCTTTCAAGATTTTTGATAGAGTGCACTGATGTCACTATCCAGAAAGATATGTGTATTAATACCTCATGATTCTGCTATTTATCTTTTCTTGATCTTGACAACCTCCATTAGCCAACACCTACATAGTAACATCAAAAACAATTTCCCATAAACTATCTGTTATTTTGCTTCAGCAACCAAACCTAGTTTTCATATTTCTTGTCTTTTAAGTTGAACAAAACATTCAAAATGAGTGGTGACTGCACTTCTTTAACTGATCTCTTTGATACTTATGTTGAGCAGTTTTCGGATACCAGCTTCCTATCACCCACTGCAGCATCCTCTGACGACATCTTCAGTATACTTGAAGTTCTTGAGGGTGTTTCTGATGATTTCAAATCCATGGCGCCTATGAAGAGTGGGCCATTTCATGTGAAAGAAATTAAGCATCCACTAGTCTCTCAGAAGTCTACTTCTTCATGTAGTGCTTTACAGGAACTTGTAGAGGAGAATATTGAGACACCTAACTCGTCAAAGAGGAAGCGACAGAAGCTTTCGTTGGTTGAAGAAGGCGGGGTTAACTCAGATGGACAGACAAAAGTGTCACATATTGCAGTGGAGAGGAACAGGAGGAAGCAGATGAATGAGCACTTAATAGTTCTTCGTTCACTAATGCCTTGCTTTTATGTCAAAAGGGTATGTACAGTTAGTGACCAAGCTAGAAAATTTGCTTATTTAAGTGTTTATCTGCTAAAAACTTAAGCATATTAACAGTTTTAAGTGTTTGTGTAAAACTGCTTATTTATAAAACAAGTGGATACCATACTGAGCATTTAAAATAAGCAATCTCTAACATTCTAATAAATTTATTGTTTTATACTAAAACTTTTACACTTACAGGGTGACCAAGCATCAATAATAGGAGGAGTAGTTGATTACATAACTGAATTGCAACAAGTTCTTCAATCACTAGAGGCAAAGAAGCAAAGAAAAGTTTACAGTGATGTTTTGAGCCCAAGACCAGCACTCAGCCCTCGTAAACCGCCTATTAGCCCTAGGCCACTCTTGCCGATTAGCCCTAGAACTCCACAGCCACTAAGTCCATATGGAGCTAGATCTCCAGCTGCATATAACTCCTCTTATATGTCATCATCACAATCATCAATGTCTAATAACATGATACCACATTCATTTGACCCTTCGCCATCTAGTTCTTCATCAACTTCAGACATCATTAATGAGCTAGTTGCAAATTCAAAGTCATCCATTGCTGATGTGGAAGTTAAGTTTTCATCGGGCTCTAATCTTATTTTGAAAACAACCTCACCACGGTTGCCAGGCCAAGCAACGAAAATAGTTTCGGTTCTTGAAGAGCTCTCTCTTGAAATCCTTAAAGCTGACATCAACACTGTCAATGAAACTATGGTGAATTCCTTCACCATTAAGGTCAGTCATGGACTTCCTTATCAAAATTTGCATGCATGCATGCTTACACATATATATCTCTTTAGTGTTTGATTCTTCTCACTAAAATAGCAACTTTACTTTTACTTATTTGGGAATGTCCAATGTTGATTACTACACTTCTTCTTTGGGGTACTCTACTCATTGTCCGTGTTGGTAATCAAAGTGATTATTGCAACTTATGTCTCAACCGAGACCTGAAATCTCAACTGCTTGGTTGATATGTCCCTATATACATGTATGCCAATCGTGAAAGTTAAACTTTATTCTAATAAGGTAATCAAGTGCTTAATGTGGATGTAATTTTAAACTGATTACCATGATTTTAGGTTGTTATAAGTAGTTTTCATTGGTTGGATAAACTGAGCATGGTTATGATTTTTATATAGCTAATTACTTGTTTACGGAGTAAAATATTTTGAAGTTACAACAATCACTTTCAAGATGCGAATTGGTACACCACCTAGAGATAGGATATTTAATTCTATTTTTAGTTATCTCGATAATCAACATTAGATATTAAGATCATAAACATTACGAAACATATTAACATATATAACGATATCAATATTAGGTTAAATGCACATCCAAATCTATCTTTATGGTGATCCGTTTTTGTCAAACACTCGGAATTAAACTGGATACTTGGATGTGATATTTTAACTACTCCGTATTAGTTTATTTATATGCACATCCAAACAAACCCTAACTTGAAAGTTTTAATGGCACTTTATCCTATTAATATCACATTCGAAAATCTCCTAAAGATGAAATCAACCTTTACAAAAACAGGCATATTTGCTAAAGAATCTAATAGTTAATTAGCCGTTAAGTAATTAGTGCTGCATGAGGAAGTTGAAAGTTGGAACCCAAAGTTACTATTATATATGCTGACAACCTGAGTTCTATCCTTTAATTGTCAGTTCTATATACGTACTAACAATTATTATTTCTTGTGTTTGATTGATTTGTGGTAGATTGGAATTGAATGCCAGCTAAGTGCGGATGACCTGGTTCAACATGTCCAACAAACATTCTGCTAGCTAATTAACTATCGTAGTCCGTATTCAGTCTTGATCATCTGCCTTGCCTCTTATTTAATTTATGCACTTGTGCTGATAGTATTGTTGTTTTAAGATCATTCTCGATACATTAAGCATTATGCTTATTGCATTGGGCATGATGTTAAAGGAAAGTACAAACCGGTAATTTTGAAGTTAGAGAAGAACAAGGCAGTAATATAAAGAAAGAATAAGAGAGGTTTAATTAACCACTGATCATGTGTTTTTATGAGTAATTAACATATGTAATCTTGGTTTAGATAAGTTGTTAATCGTATGTCGTTGATTAGTTTAACTCTTGTATCAGACTATCAATCATAATATGTACTAAGGTTATTATATTCGTTTAATATCGTCTGCAATCCAGATTTTCATGTTATGTGTCTCGTTTATATGCTATGAACGGCGATCAATTAAGATGCAACTGTATAAACTGTTAGTCATGAAAGGGCAAATGACCTTTTTGAGATGTGTAGCTTTTGAATGAATGAAATGTTTGTGCTTCTTGAATTATTTTGGCTTCAATTGTTGGCTATAAAAGAAAGGCTTCATTGCCTCATTCATCATCCCATCTCATTCCTTACTAAACCCATATAAAATTCTTCCCATGTAGTTGTGAGTTGATTAGAGAGTTTGTGAAATATCTCTTAATTACAAGAGATATAAATGTGTTTATCCTTGTAATAAAGAGAAGTGTAATTCTTA

AabHLH60

CAGAAGATTTGTCGAAACTATCACTCATGAAGTGAATAATAATTGATGCCAAAAAACACCCGAGTTAAAATATTCCGTCAATTCACACCTTTCGTGGATAATATGTTACATAACATATATACCTATTAAAAATTCAATTATTACTAGGATGTATAAAACAAAAATCTTGAGACACCTGGCTAGCTAGTTGCCTTAAATCTGAACATATATCTATTCGGTCTTATACACGTTAGTCAAAAAGATTAAAAACAACCAATGATGTGCTGATTACCATTATTTCTAGTTCAAATTGTTAATATTTCTTTAATTTATTCAAACTCGGCTACCTTAGTTCACATCACCGACTTGTGTTAAGATGGAAAACAACACTTTGTGGACCCTATTACTTACATCAATCTAGACTTATAAAACCATGCTAATTCTGTAGAACATAAACATTTTACAGAAACCATAATCATTAACCATCCGCTTATATTTTTTCTAACAAAAAGGTATAGTACAAGTTTTTTGTCATTTCTTGAATTTTCATGCAAGTTTTCTTGATATGGCTATATTAGATTTGTGTTGATATATAGTTTGCTTTTCGCGTGCAGGGTGATCGTTTGTTTTCGATTTGTTGTTGTTTCATTATCGTCGCAAAGAGTGGATTTGGTCGAAATTATATCAAGGGTCCTCATTAAAACCTTTTTTCATCACCAAATTTTTTTTATGGAGATTACACAATTCAGAGGGTTTTCGGAATTGTCAGGAATTGAGGACCCTTGTTTTAATTCTCAGTGGCCATTTAACTCATTTGATGATCAACTAATGAACGTGGATGCATGTAACTCATTTGCTTATGCACCCATGTTTGATCATATTCATTATAAGCCTATTATGGAAGCATCACCAAGACCATCTAAGCAACTCAAAACCAGCAGCTGGAATTCGAGCATTAGCTCTGACCAGTCCATGATGAATCAGACTATACATTATGGTAGTGATCATTCGCATGTCGCAAATCAAGCTACCCTTATGAGTCCTAAAGAAGAAGCAACTCTATCTTCCAGCAGTGTATTCCAATCATCATCCCATGTTCAGTTTGGCAACCAAAACCACCATGCGAAAAAGATAAATTCTTCAAAATTCACTACACCTCAAGACCATATCATGGCTGAAAGAAAGAGAAGAGAGAAACTGAGCCAAAAGTTCATAGCCCTCTCTGCTTTGATCCCTGGCCTTAAAAAGGTACCCCTTTTAGTTAAAGATTTTATAATAAATTCTACTATCATGCATGGATATGTTCTTACTTATGTTACTATATATTTATAGATGGATAAAGCTTCTGTTCTTGGAGATGCAATCAAGTACTTGAAAAACCTTCAAGAAAAAGTGAAGACACTTGAGGAACAAACCAAAAAAAGATCAAATGTAGAATCAGTGGTTTTTGTTAAAAGATATGAGGTGTTAGGTGATGGTGGTGAAATCTCTTCATCCGGCGAAAACTCTCAGGAGCAACTGCCTGAAATCGAGGCACGATTCTCAGGCAAGGATGTTTTGATCCGAGTTCATTGTGAGAAAAAAGCCGGTATAGTAGAAGAAATACTAGCTGAAATTGAGAAACTTCATCTATCTGTTATCAATTCCACTGCCTTGACTTTTGCCAATTATGCACTCGATATAACCCTAGTAGCCACGGTAAACAGCAATTTCATATTTTTTGTTATTATTGCTCATATATTGTTTTTACACACTTATTGATACACATATCATATTTTATTCAGATGGATCAAGAATTCACCATGACAATGAAGGATCTTGTCAAAAATCTACGTATCGGTCTTAAACGATTAATGTGAACATTCTGGACTAGTTTCATATCCTTGTTTTTTTTCCCCCCCAAATAGCTGTAATCTGGTAACAAAGGCTTGATGTTCTCCACACCATTAAAACTATCCACATGCCTGTAAAAATTTGTTTGCTATTAGGTTAATTTTGCAGGAGGGTTTTTTACCATATTTTTTACATGGTTAACTTGTTGACCATGTTAACTACCCTCCTGCTGTAAAGAAGTAACATTTAATGGCTATAGTGAGGCATGTTTTTCTATAACTTTTTCCCTTTTCACATTGACTTAAAAGGGGTTTTTAGTGTGGATGAACATATAGACCTTTGTAATCAGTCAAGTGTTTGGTGATTATTGTTTTAGAAGCCTTTTTTTGGGGCCTCTTTTTCCTTATCTTCTGTAATTTGTTTGTGAAAGCTTTGATTAATGGTTTAAGTTTTTTTCCTTATATGTATGTAAAAGCTTGATTAATGATAATGAATAATGTTTTAAGTATTTCCACGGAATACTAGTTCGGTTTTTAATTCTACTTAGCGTAAACTACAAAAATCGTTTGTCCAGTTTTTTTATTTTTTGGTACCAGCCATCTAGGTTAGAAACTATTTGCATAAATTGTTTCTAAGAAGTCAAAAGTCTATCTAGCATGCTTTTTTACAAACTTTTTGTATGAATTATCCGTGAGGATTTTCTTTTCATTGTTACGGCTTCTTATGGATGATTCATGTTAAAAGTTTCTAATCGAGATGATTTGTGTGAGATTCGACATAACCACATGAACAAATTTGCAATACACTCTAGTATTGATCATGTGAAGA

AabHLH61

CTTGTCACTTAGTCCGTTTAATCATGATATTACAGGATTTACCTTTTTTTTTTGGGTATGTAAATATATTACTCCAAAAACGTTTAAGTACAAAGGATATGGGTGAATCTCATATCTCAGCCCATTACATATGCGGAAAAACTTACATAACAAGATACTTCATATTCTTTATATAACAAAATAAACTATTACTCAAAAGATAACCTCTTGGCAGTGAGATGACCCAATTTTTAATTAATAATATATATAAATAGAAAGTCAAAGGCATCCTTAGCAGACCAACTAGGGCACGTTACATAGAGATAAACCTTGTCTCAACCTTGTTCATTTTGCACACTCCACACCATATATAGCTCTGCAAAACCTCCCTTGTTTGTCCATATCTTCATGGCCTAACCCCACTCTCCATCAATTAGTTATTGATTACTGAATTCTGGACCCATTAGGGTTTGTCTTTTACGTTTCATTTTTGTTGGTCAAAGTCTCTTCTAGCTTGTTTTGTATTTATATATACCCAATGGACAAAGATGATAACAACTACTCGGTATGCACATATTAACCGTAAATATTTATCTCTTGTTTTCATCACTAGGTATGTTGTGTTACAATGAATTTGAACTCTTTCAGGGTGGCTTTCTTGGAGTCGATTTTAGTGGTGAGCATGTGCAAGACCATGAACATCAGAATCAGCAACAGTTCATGGTGAAAAACACGTACGAAAATGGGAATCAAATGGTGGATTACTTGCTTAATAATCCCATACAAAATCTACCGTCTAGTGGTTTTTGTAGCTCGAATTCTTTCGATAAGTTGAGCTTTGCAGATGTGATGCAGTTTGCGGATCTTGGACCAAAGTTGGCTTTGAATCAAAACAAAACAAGTCATGATCATGAAGAAGAACAAGAAAACGGGATAGATCCCATTTACTTCTTAAAGTTCCCAGTGTTGAATGAGAGGTCACAAGAAGATCATGAGTCTTTATTGGCTCCTCTTGGAGACGAAGAAAACGAGCATAGATTAGTGATTGAAGGAGGTGAAAGAGATGAAGAAGCTAGGGTTTCCGAAGGTAATTCTGTTCGACTTCAGTTTATGGGTGAAGATGTTCATAAAACCCTAGGTACAGAAGGCAAGAATAAGAGAAAAAGACCCAGAACTATAAAGACTAGTGAAGAAGTTGAGAGCCAAAGAATGACTCATATTGCGGTCGAAAGAAACCGTAGAAAGCAAATGAATGAACATTTACGCGTTCTTAGGTCTCTCATGCCAGGCTCCTATGTTCAAAGGGTATGCTTATTACAGAAAATAACTCATGTTTGAACTCATAATCTTTAGCTGGCTTATGGGACACACTTAGTACCACAAAAACCTTCGGATCCCTGCACGCTCATTTTTTTTATAAAATAAATATATATGCAAAGCAAATTAAAACACCTAATTGATGATATGATTTCATGAACTATAGGGTGATCAAGCATCTATTATCGGTGGGGCAATTGAGTTCGTGAGGGAGTTAGAGCAACTCTTGCAATGTCTTGAGTCACAAAAACGACGAAGACTCTATGGAGACACTCCAAGGGTGGCCGGTGATTCATCGTCCCTTCCAGTTGTGCAGCAAGGGCCTCCAGCAGTTTTTTACCCTCCATCTGATGAGCAGATGAAGCTAGTGGAATACGACGGCGGACTGAAGGAGGAAATGGCAGAGAGTAAGTCCTGCTTGGCTGATGTCGAAGTGAGATTATTAGGGTTTGATGCCATGATCAAGATTTTATGTAGAAGAAGACCTGGTCAGCTTATTAAAATTATTGCCGCACTTGAAGATTTACAGTTTACTATCCTTCATACCAACATTACCACCATTGAACAAACTGTGCTCTATTCATTCAATGTTAAGGTATATATATCATCATTTCGTCATATTCAAGTTCATTCATTTTCGTTTTTCAGTCTTGTAACGTATTTGAATCGTTCTATTTGTAGGTTTCTAGTGAAGCGAGGTTCTCAGCAGAAGACATAGCAAATTCTGTACAACAAATTATTGGTTTTGTGCATGCAAATACTAGTTCTTGATCGACAACAAAGAAACAATATACTTCATCAGAACATACCAACGGGTGTAACTTCTTGAACGAGCAAAATAGAGACACCCGTTGGTTGTAATATCTTTGCCCGACTTATTGAGCTATTCTAATAGCTAAATCTGCTGGGAGCGACATTTTCATAAGGCATCATGTTCTTATTATTCAAATTTTTAACTTGTGCACATTAATTATTTAGTCTATTTTTATGTACGAGTTGTTAGTATTACATGTTAAAGGTAGCATAGAGTATATATACTACTGTAAGGATTTATTCCACATATTTAGTCCATCGTCTTGGATTATATTGAAATATGAATTATATATCATAGTTAACACGATCTCCTTTTTATTCATTTGTCCATCAAGATTATAAAGCTTGATAATTCAGCTAATATGTATATATAATAACTGGTCAAATCGAGTTATAGCTTTTAGTAATTAATCGGTGTCGAATGACTAGAGCAAACGAGTATTTCATAACATTATTACTCGTTTTGACATATTATTCATTCCAGATTTCCATCTATTTACCACCTTCGATTGTAAGAGTTTTATCTGATAT

AabHLH62

CACTACGTGACTTAAAATCGGTTATAGCCCGTGCCACACCCGGCAGACATGTAGAGTCGTCCCTGTTTAACATTATACACCGTTGCTATACAGATACTCGTCTAATATATACTCCCTCCGTCCTAAAATTATTGTCTCCTATTGACTTTTTCGGTCTTTACTTTTTAACTTTGACTTTAAGTATTTTTGTTTATATTACATAATACTTGATATAAAATATATGAACGGATTGAGTTTTAAATATATTTTCATTTGGTATAATTTTTATCAAAAATTTTGCAGCCAAAGTTGAGAAAGAAAGACCGAAAAAATCAATAGGGGACAATAAATTTAGGACGGAGGTAGTATACCGTCTAAATGCAAAATCACGCATGTACCACTAACTCTTTTTTCATTATGTTGGGACCAACTTCAGATAAGATAAAATTCTACTAAAGAGAGAGAATATGATGTCGATAGAGAAGTCACTATCCTTAATCAGATGCCTATTAATATTTCCTGATTTTGGCATTTCTATTACCTCGAACTTCACAAGATTCAAAAAAAAAAAAAAAAAAAAAAAAAGAATCATATTTCATGGTTTAGCTAGCCTCTATCTGACTATCTTAATAAATTGCACACAACTATTTGTTAAGTTGTTGTGCTTCCTTTTTATTTTATTTTTATTTTTTCCTAAAACAAACAATCAGAATGGTGAATGAGTCTACTTCTTTAACTGATCTATTTGACCCTAATGGATCATTAGCATATCCTGACGACATCTTTAATATTCTTGAGGCGTTAGAGGGTGTTTCTAATGAATTTACGGTGTCTACACCTTTAGCTGATTCCTCGAGTGGTGTACAAGAACTCGTGGAGGCGGAACATGAGGCGTTTTCACCAAGAAACAAGAGACAGAAGGTATCGGGGGATGAAGGTTGCGAAAACTTTGATGGACTGGTAAAGATGAATCATGTAACGGTGGAGAGGAACCGAAGGAAGCAGATGAATGAACACTTAACCGTTCTTCGGTCACTCATGCCTTGTTTTTACGTCAAAAGGGTATAAATTTATTCTAATTATCCAAGTATTGTAGAGTTAAATAATTTATTTGAGAGTAACTAATTAGTTTTTGGTCATACTACTTGTTAGATCATAACAATTTCCCAACGACTCCAACCAATTGTTATATGTTAATTGTGTTTTAAACAAAAAAAGAAGAATCAAATTACTAAGGCGCGTTAAGTATCGCTTTCAAAACACAATATGAAACACCGGCACTAGATATGATATACCATGAATTTGATCGATTAACTTGTGTGTTTATGCAAACTGTAGGGTGATCAAGCATCAATAATTGGAGGTGTGATCGAGTACATCACCGAACTGCAACAAGTGCTACAATCTCTAGAGGCAAAGAAGCAAAGAAAAGTTTATAGTGATCATGTGTTGAGCCCAAGGCTTATTTCAAGCCCTAGAACAACCCTACCTCTCAGCCCTAGAAAACCGCCCTTGAGCCCGAGGCCAATTAGCTTGCCAATAAGCCCCAGGACACCACAGTCAGCTAGTCCATACCGGCATAGGTTACCATCTGCTACTTCTTACCTAAATACCAGACCAGCTTCACCTTGCAACTCTTCATCAAATTCTGACACCATTAACGAGCTTGTTGCAAATTCGAAGTCATCCATTGCGGATGTTGAGGTGAAGTTTTCAGGAGCAAATCTTCTTTTAAGGACTTTATCACCACGGTTACCAGGCCAGGTGACAAAGATAATATCAGTTCTTGAGGACTTATCTCTTGAAATCCTCCAAGCTAAAATCAACATTGTTAATGAAACTCTGGTTAACTCATTTACCATCAAGGTGACCTATCTTTAAAGCTTATTTTTATTTTATTTTTTTGTGAAATACCATTATTATTTTCATGTATTGCATGCATGCTTACATTACCATAAATATTTCACTTGTTCTTTCTTTTTCAAATATATATTCTTCTTTCATAAAAGATTTTTAGGTAAGCCTAAACAAACCGCCTTTTGTTGTTCTTTGTTATGTAATAAACAAATTATCCTAAGTTTTAAGTGAGTTATGTCCTCAAGATTAAGTAATCTAAGTTTACATTACAACTTTTTACTTTTTTTTTGCCAAAACTGAAAGTTGAACGCATATATCACAAAATTAATAAACTTTCGACCAATTTTTCATATATTTTGTTCTTAATAGTACTACACTATACTACTTAACTACTATAGGTTTGTATTGAAATTTTCATAAAAACTATTACAAATTCGGATAAAAGAAAAGGCAAATACCATAATTTTCAAAGTTAATGATTCAAATTAAAATTTCAAAAAGACAATTGGAGAGAGAAATGAAATTATTTTGGTTGTGTTGAGTAGAAAATCATGACAACATCATTCTCTTCATGTAATTTTGGTGGACCACATAATTAACTTAACTAAAATAGTAGGACAAAAATGTAAAAGCCATAAACCTTAGGATGAAAACTATATATTACCCATATTCATCATCTTCAACCGTGTATCATGCCTGTTACAGTGTTACTCTGTTTTTTTCGGATTTTCCTCATTAAATCATTTGAAGTTGATCTTTGTTTTGCTTCTTTAAAATCAAATATCTTAAGTTCATCTTTTTTTTTTTTGCAAACCCAGAATAAAATCAGCGCTGCATTTTTAACATCGGAGGCCCCAAAATTTTGGAGGCCCTGTGCGGTTGCACACTCTGCACACCCTAAAATCCGGCCCTGGTCCTCCCTCACTCGCCACCCCATTCCAACAACACTCCTCACGTGCGAGGAAGAGAAAAAAGATAAGATAGAGGTGAGCCGGTCATTTCTCGGTTCTTGTGCATTTTTTTTGTATTTTATTGTTTATTTTATTTATGATGGCGTGTGTGGTATTTAATAGGGTATATTTGTATAAAGTGGATATTGTAATGAAAGATGAGCAGTGGTATGACTACTACGGATGAAAGGTGAGAGGAAGTGTGAGGAAGTAAGAGGAGAGAAAAATATTGAGGTGACATTGATGTAAAAGATGAGGAAGTGTGACCCCTCGCTCTAGTCTAAAGCTTTCTACCATTATCCATGTTGATTAGGAAATAAAAATTTGTTTCATAATTTGTCTATTATATAATGTAGGGTACCCTTTTTATTAAAACATAAAAGCTTAAAAGATTAGTAAAGTTCAATTACTAATATATTATTGATTAAAAAATGGTAACAACATTTATCGTTAAACTAATAGTTTAAATATTTTTTAATTCTTAAACTTTTATTATTTTCCGGTTTTAGCCAATAGACTTTTTTTCTCTCAATGACTACATATTTGTACCCTTATGTGTTAAATCAGGGTCAAACGAGAACAAGAAGTTAACAAGTATAACTATTTAACAATTTAGGGATAGACCGAAGATTTTAAAGAGGGTATTGGACAAAAGAATCGTTAGGCTAACTAAAGTTACATTGTAAGAAATAAAAGTAGCAAATAAAAATTTGCGTTCGTAAATACCAAAAAATGACAGTTTTTTTAAATTTTTTGCACTACGTGACTTAAAATCGGTTATAGCCCGTGCCACACCCGGCAGACATGTAGAGTCGTCCCTGTTTAACATTATACACCGTTGCTATACAGATACTCGTCTAATATATACTCCCTCCGTCCTAAAATTATTGTCTCCTATTGACTTTTTCGGTCTTTACTTTTTAACTTTGACTTTAAGTATTTTTGTTTATATTACATAATACTTGATATAAAATATATGAACGGATTGAGTTTTAAATATATTTTCATTTGGTATAATTTTTATCAAAAATTTTGCAGCCAAAGTTGAGAAAGAAAGACCGAAAAAATCAATAGGGGACAATAAATTTAGGACGGAGGTAGTATACCGTCTAAATGCAAAATCACGCATGTACCACTAACTCTTTTTTCATTATGTTGGGACCAACTTCAGATAAGATAAAATTCTACTAAAGAGAGAGAATATGATGTCGATAGAGAAGTCACTATCCTTAATCAGATGCCTATTAATATTTCCTGATTTTGGCATTTCTATTACCTCGAACTTCACAAGATTCAAAAAAAAAAAAAAAAAAAAAAAAAAAAAAAAAAAAAAAAAAAAAAAAAAAAAAAAAAAAAAAAAATATTACAAATTCGGATAAAAGAAAAGGCAAATACCATAATTTTCAAAGTTAATGATTCAAATTAAAATTTCAAAAAGACAATTGGAGAGAGAAATGAAATTATTTTGGTTGTGTTGAGTAGAAAATCATGACAACATCATTCTCTTCATGTAATTTTGGTGGACCACATAATTAACTTTTATATGTGTGGACAAATGGTTGTAAAACATTATGGGACTTCTGCTAGATTAGTTTCTTTGGTTCACGCATTTTGTTTTTTATAGCTCTCACACATAATACAAAAATGACATAGGAAAAAAGGTACAGAGGGTAATAAAGGTTGTTCAGTATGGTAGTTTTGGGAGATGAAATTAAAGTGGTAACAACAAGGTTGCAACGTACACATAATAATTCAAGATATCAAGAAATTTAATGGCCAAATTAAACTATATGTATCATGATGTAAAATAAATTAGTTCTGATAGTACATTGTTTCATTTATAACAAATTTGACCTCTAACATATTACTAGATGTATACATTTCTGTGTAACATATGAGATCATGTTTACTTTATAATTTTGTAAAACAAAACCATCTGTATGTTTAAATTTTAACTGCAATTTTCATTATTCAATATGTTTTATGCATGATTTGGGATATACGAAACGATAACATAATTAATTTGATCGCTGCTGATTAAATAGTTTAATTAAGTATATATAAATCACTTGATCATGCTTTCACTCAAATACGTTGAATAGTTATGATTGCATCATCATTTGTTGGAAACATCATAAAAAGAATGTTAGCATTAGGTTGGTTTAGATGTCAAAAGGTCCCATCTTTGTTTGTGACTGAACAATCAATATCATGGCCATTAAGTATGTACTGCATGTGCCTTTCCCAAAAAAATAATAATAATAATAAATAAATAAATTGTACTGCATGTATGAGTCTTAAAACCAAACCTAGCTACTGCTTCCACCTGAAAACTTAGTCTTATTGGACTATATTTTTTGACTGTTATAGCAAAAATAAATGTATTGGTTTTGAAGGTATAGCAATCCAATGAGATTTTTCCCTATTGAGGTTAAGGCGAGTCTTTGACTAAGACGTACATGGCTTAACTAGGGTTACCCTGTGACCATTTTCGACTCTTTAGTGGCCACCCCCAAGATGTAAACCACTGGCTTTGAACGTACATATAACAAAAATAATTATACCTATTTGAATGTTTTCTATACTATTTATTAAGTAAATATTTGTTAGTAGAAAGAAATTAAATTGCTCTAGAAATTCATATTTATGGTGGCTGGGTTACACGTAATTTTGTTGAGTTGCGGCTTTTTGTTATGGCAGATTGGAATCGAATGCAAGCTTAGTGCTGAGGACCTTGCACAACACATTCAGCTCACATTTTGCTAGGATGAACCTAGCTATTTCAGTTCGATATCTAGTCTCCTTTTGTGTGACGGATAATTTTGTTCTTTGAGATCATATATACCGGTACATTATTATATGTAATGTATTGAGCATGATGTTAGCTGGAGCAGAATATGATCATCAAAAGAAGCAGAAGCAGGTAAAGCTAAGAGGATAGAACTCTAGCAACATCTATCTCGGTCTATTTGTAGTTTTATTAGATTTAGTGACATTTTATATCAAGTATTTAATTGATTAGTGTAACCGTCAAGTTGAGTAGAACTTGACATGTACCTGTTTGATTATTTTGTTTAACAAATGTATTGACTATTCTCAACTTTATAAACTTTTACACATTCTGTTCTTTTCTTTTAATTGTATAATCAGGGATAGCAGAAAATAGCTTGAACCTGACGGAGGAACCTTTAAACTCGAACCTTTTGGGGTGGGGCATACTTATCGGTTTACCGGAACAGGTTTACGGTGAAACCTGAAACTTGAACGGGTTTCAATCATGCTTGAACCCGCCCTGCCTTGATCTAAACATGTATACATATAGGTTTATGTTAGTATATACGTAATAAAAGCATGATTCGTATTTTGTTAGTCTTTTAT

AabHLH63
[truncated: 668,524 more chars]
