# Supplementary material for: Genome-wide identification and characterisation of bHLH transcription factors in Artemisia annua
Source: BMC Plant Biol. 2023 Feb 1;23:63. doi: 10.1186/s12870-023-04063-8 (PMC9890702; doi:10.1186/s12870-023-04063-8)
Supplement: Supplementary file 4 — Additional file 4: Supplemental Material 4. 226 TFs protein sequences. [file 12870_2023_4063_MOESM4_ESM.docx]

**Supplemental Material 4 226 TFs protein sequences**

(Green background represents bHLH or HLH domain, yellow background represents Myc domain).

>AabHLH1

MDSIFDLEETERSRLLRQIMDSFGFTYICLWSHFTQPSNCLICIDGVYKEGNNQASSSSGSLTMTSFLDYKKLMFFIDNYTGGVPGFAFMHNITYMERKKLELLTLASNSAQLQFYQEAGIKTAIFMGSSNGEIELGMTNDSSQINFEIELKKLFPGDFREGVLLQRLEEARASSSSSSLRSLSMDNSVENSPFLFNMFHSTPYMSEMFALTERQPPRVASAFKRYGSSNLGPIKQRVPNRQNLHRRSLSFLRNLSEARAQRDQMVQTTRPTSNQLHHMIAERKRREKLNESFQTLRSLFPPGSKKDKASVLSNTMEYISSLKSQVEELNKRNQILEADQRARKEPPNQGSSRFSGEGPVVGITDIGESTSDSRVVDLEVNARGNVILVDLVMSVLEFIKQSENVSVMSIDAGTRMLETEAIANRVIFRLRIQGNEWDRSSFEEAVRRLLGDLAQ

>AabHLH2

MSFIGTLSSSTHEEVALEKGSSGVISASNLESNCGRGFLSLQNLESNCGRGFLSLQGFCKQSFLNMDSIFDLEETERSRLLRQIMDSFGFTYICLWSHFSQPSNCLICIDGVYKEGNNQASSSSGSLTMTSFLDYKKLMFFIDNYTGGVPGFAFMHNITYMERKKLELLNLASNPAQLQFYQEAGIKTAIFMGSSNGEIELGMTNDSSQINFEIELKKLFPGDFREGVLLQRLEEARASSSSSSLRSLSMDNSVENSPFLFNMFHSTPYMSEMFALTEPHMDQQAPNQTPQSTTILRPEDPLRQALEQIRTSQYGSSNLGPIKQRVPNRQNLHRRSLSFLRNLSEARAQRDQMVQTTRPTSNQLHHMIAERKRREKLNESFQTLRSLFPPGSKKDKASVLSNTMEYISSLKSQVEELNKRNQILEADQRARKEPPNQGSSRFSGEGPVVGITDIGESTSDSRVVDLEVNARGNVILVDLVMSVLEFIKQSENVSVMSIDAGTRMLETEAIANRVIFRLRIQGNEWDRSSFEEAVRRLLGDLAQ

>AabHLH3

MGWWVELLLVFGICFYPAIKYGMVGIYCVWMYLIFWIYSSWSRRNLGWGRRNLGWVGSGVGRRLFSGEVQEYEEDELSTARTHRNDLKDTADSCSSRLVKSRMALSSFSHKSSGSSERKREKMRKMVNTIREIVSSGKQMNSVPVIDEVVKYLKSLKIELQEVGVGI

>AabHLH4

MEIDENSNWLLDYGLMDDISTVVDYVAPPAVVVGFSWPSDIPAVSSEIESSFIDFEGLKGAGSRKRLKSECSNVCGSKAGREKQRRDRMNERFMELGSILEHGKPPKTDKTAILSDAIRMIMQLRSEAERLNESNVDLQEKIKELKAEKNELRDEKQRLKVEKEKLEQQVKTMNIGQPSYLAHPTAMRGAFAAQEQAAGNKLMPFVGYPSVAMWQFMPSSVVDTSLDHVLRPPVALKSECSNVCGSKAGREKQRRDRMNERFMELGSILEHGKPPKTDKTAILSDAIRMIMQLRSEAERLNESNVDLQEKIKELKAEKNELRDEKQRLKVEKEKLEQQVKTMNIGQPSYLAHPTAMRGAFAAQEQAAGNKLMPFVGYPSVAMWQFMPSSVVDTSLDHVLRPPVA

>AabHLH5

MSLQNATNSFDPASSHDDFLDQMLSGLQSGASWPEISTGGGGGNGWDVDQFDDQSNFLATKLRQHQISSGPSSAAKSLILQQQMMISRGLAGAGELGNFQNDMVDASSFKSPRGDNSIQTLFNGFAGSLQSHPSQEFPFPPTQSFGSPGTTSALVNQGQTNGGPPATTGGGSGPPAQPRQRVRARRGQATDPHSIAERLRRERIAERMKSLQELVPNANKICFHVIAITSPRKFSRMNSKVYSTKLRQHQISSGPSSAAKSLILQQQMMISRGLAGAGELGNFQNDMVDASSFKSPRGDNSIQTLFNGFAGSLQSHQSQEFPFPPTQSFGSPGTASALVNQGQTNGGPPATTGGVSGPPAQPRQRVRARRGQATDPHSIAERLRRERIAERMKSLQELVPNANKTDKASMLDEIIDYVKFLQLQVKVLSMSRLGGAGAVAPLVTDISPEGARDRVQVASGGVAGRTTNGTTSSSNNETMSMAENQVVKLMEEDMGSAMQYLQGKGLCLMPISLATAISTATCNPSSARNNHPLLGVADGNSPSSPNLSALTVQSANGGLQTEGVSIKDTTSISRGVWTLTNGSGTDKWGVWVLTEDDAEVESKSSHIFEDSK

>AabHLH6

MANMFDNICSSSYSPMSHEPSDDISVLLRQILSKSSSSSSSSSLLTKQQQQQQPHSADVANVAVNVGAIDYDHSDEYDCESQEGFENLMEEMDGKPNPPRNPSKRMRAAEVHNMSEKRRRSKINEKMKALQKLIPNSNKTDKASMLDEAIEYLKQLQLQVQMLTMRNGINLYSMYAPHGSVQPNNRPSNVNQGNHSINMAPNQERLVNPMLGQPIQCMSQNKQSILDFSCTINQEPPFGTQLGSS

>AabHLH7

MADLYGNDHRSFSSSSSLESEDMSSFLQTFINNNNGSSASNKYGGGGPLIPSPVPEFHDSDIRFSDLSSFYSPEPNQVQKVSDVRQSSNPTRSSKRTRAAEIHNLSEKRRRSRINEKLKALQTLVPNSNKTDKASMLDEAIEYLKQLQLKVQTLAMRNGFGLQPVFSQEQEMQRGICYDEGYKFVSSSDGAAGTSSQTQGFSMRREFGMPNHQIGMMMKPNPVFGSELANENQYGFTNHSTSVKDTSRSFSGKGVSS

>AabHLH8

MLSGLQTNVSWPDISNGGGQNKPSLPWDVDHFDDQSAFLSSKLRQHQITGGARTPVSPCMSKPVLKAIEAIFRKLNKTCNINIFDMIGENNNNNAIQNLFHGFTGSLATNQGQQFRNLPAQNFGSPAAAVAAAVMNQVQAGSVTAAGGGGGGAPSQPRQRVRARRGQATDPHSIAERLRRERIAERMKALQELVPNANKESQMSLQDLQNNETSNNNNTFDPTAAHDDFLDQMLSGLQTNVSWPDISNGGGQNKPSLPWDVDHFDDQSAFLSSKLRQHQITGGARTPVSPCMSKPVLKAIEAIFRKLNKTCNINIFDMIGENNNNNAIQNLFHGFTGSLATNQGQQFRNLPAQNFGSPAAAVAAAVMNQVQAGSVTAAGGGGGGAPSQPRQRVRARRGQATDPHSIAERLRRERIAERMKALQELVPNANKTDKASMLDEIIDYVKFLQLQVKVLSMSRLGGAGAAAGAANPMAAEGGGDCGQRGGPGTGRSSNGTTSSSNNETMTVTENQVVKLMEEDMGSAMQYLQGKGLCLMPISLATAISTATCHPSGTRTNHLAGGEGGPTSPNMSVLTVQSANGVKDSP

>AabHLH9

MYNTQATSSLPETTDDISLFLRQILLKSSSLSSATASTTTKPTMLCSSVVGAKQMQCEMPRVQPVYSSGLVSVPEWGCSDLVGGYAPVTYVSSSSVGTLDNEVDEFDCESEEGGFENLLEDMATKSNQTPRNPSKRTRAAEVHNMSEKRRRSRINEKMKALQKLIPNSNKTDKASMLDEAIEYLKQLQLRVQMLTMRNGINLYSMSVPPGLLQPQQLPYSRTGFNEGNDPPNMTRLNQEPHMNPMLNLPIQSTNRSQPSIPNLSHIMNQGPSLSFRPPLGPFKECLINSSVSDGTQLMNSEQKLVLPDNLNGGLHQK

>AabHLH10

MEHPQQRLLGTQGRKPTHDFLSLYSPAQQDPSAATTTPSGSYLETHDFLQPLERVGKNVAKEENKVEESSIGKPLPPTPPTTVEHILPGGIGTYSISHISVINQSQRMPKPEGVGVVITGAQSSGSDKNDENSNCSSYTGSGFSLWEESAVKKGKTGKENIAGNRHVIREGGMKMGGMPWMTSMERPSQSSSAHNHPTATISSLSSSRPSSAQKNPSFVDMLKSAKSVQEDEDEEVEEFVIKKEPSTHYKGVLSVKVDTAHHDQKPNTPRSKHSATEQRRRSKINDRFSMLRELIPHGDQKRDKASFLLEVIEYIQFLQEKVHKFEDSCRGWPNEQPAMTPWNNNQRPTEGFIDQPRVQNGVSGSALPYASKLNENKHSVALNLPKKDHQNILDSDLSSLETMKDIGQHPRLTNLASPFPSPLQPNVYSPGCGSTSVAAPPPSTLPSDTTNTPSQLWQSRSCTTDCTVAGDKSKDQDLTIESGTISISTIYSQGLLSTLTQALQSSGVDLSEANISVQIDLGKRANTSRRDSSTPIFKKNEAPVIDENLTRSRLASTREEKNDNDLKRFKTSRN

>AabHLH11

MSSWLHYPVEYPANENSLEGYLYNNDLLFPIPPPNPVTTTPMAPTTLPPPPPPSVVISSPRPPVAPIRRNHVDIQPRQQPKYPNFLHFSRPNKVTTLESGASAPVTEAPESRASRVSEKPPPTSVGGESVSGVGLVGTSSMGREVETCDTSMMSSPDGSGASGSIEPSTQMPPPLTNDRKRKGRDTEDTECHSEDVECEYPEAKKQSHGSTSTKRSRAAEVHNLSERRRRDRINEKMKALQELIPRCNKSDKASMLDEAIEYLKSLQMQVQMMSMGCGMVPMMFPGVQQYVSPMAMGMGMGMGMGMDMGMNRPMVPYPAILPGPSMPNPAASAAAAAAQLGQRFPVPGFNMSPVAVAGQAANMSAPMMSSFPLQNQNQPRVPNFADPYQQYLGLHQTQVPLPQNQGGIPPTATKPGSSKDATNPDHHQNG

>AabHLH12

MACESVLMAYGPDCIGAWWRLSLMAFIMYDLYFKSLEVIAMKPDEIAFELDWMFLTEGLCCLCEGNVQVEVYVLVVVICFVVCEIFGFMSWDQVPRASFVRRFNVISGSCEEDFFMFFKRKTGFGLLLGNKCFMVKPSFSGKQKLSLFRLFSGDEYIENKVLNCRKIKNGEGGFENLLEDMATKSNQTPRNPSKRTRAAEVHNMSEKRRRSRINEKMKALQKLIPNSNKTDKASMLDEAIEYLKQLQLRVQMLTMRNGINLYSMSVPPGLLQPQQLPYSRPGFNEGNELPNMTRLNQEPHMNPMLNLPIQSTNRSQPSIPNLSHIMNQGPSLSSFRPPLGPFKECLINSSGSDGTQLMNSEQKLVLPDNLNGGLHQK

>AabHLH13

MELSQPRPRGEPGAKPTHDFLSLYSPAHQDPSPTIPGSYLKTHNFLQPLEQVGKTVCEEVEYIKKSFPPSPPTVGEHILPGGMGTYSISHIPHINQTQKVSKPEGIVISAAQSSSSNNNDENSNCSSYTGSGFTLWEESNVNKGKTRKENNIAANRHTMRDGGMKFGVPWMTSIEQPSKSSSINNHPSTTFSSLSSSHRSSAPKSQYFVDMKSGRNFQEVEDINEGFVIKKEPSYHHKGGTSTKADTSNSDKKPNSPRSKHSATEQRRRSKINDRFSMLRGIIPHGDQKRDKASFLLEVIEYIQFLQEKVHKYEDSSQGWTNEPPKTIPINDFIHQPQIPTNQNLLDSNMISHDATNETSQHPQSTNKPSASAAASYEQISTPDTATTASQAQELIIEGGTISISTIYSQGLASALTQALNSSGVDLSHANISVQIDLGKRSNANTNTLESSPHDLKENETRSNDQSIAHSRLVSTWEDENDQGFKRLKTSRN

>AabHLH14

MNQCVPSWDLEDFNSNLDIFKLGYEVAELTWENGQVAMHELGHRRVPSKSQPETSWDKPRAGETLEAIVTQATYQPYCKTHVVVNDNELVPSATMASDALVPSARDAGCSTHVGSCSNDPSAFLNERVACGGDDGGCATVRCHDMTMSGCGTYETFDTYDGDTGGQRLIETSMGSPENTSSGGDCLKSRSPDDSACHCRTKEVNVVTEKKRGKSESSTSTKRRRTAANHNQSERKRRDKINQKMKTLQKMVPNSNKTDKASMLDEVIEYLKQMQAQVHMVNRMNMPPMMMPLVMQQQQQQQQQMQMSMMNSMGLGMGMGMGMGMGFGGMDMNTMALSHLPTGFHPTTFMHMPWNNHITDRVVNYGPMAGDPMSAFRLSRSQPMNMDAYSRMAALYQYMQNQSSGSHPKN

>AabHLH15

MAERLRGDEDIMELLWQNGQVVMQSQNQRSNDEDTAPCNLYMHEDEMVSWLHYPSDDNNNLDLYLHNNDILYPVLPAANVAHSLQGVSAISIPPPRASSVVTRVGEEQSQNKFGNFSHFSKPSFVKNNPTAGSAPTSSNKMSEVTTVVESNNQPMEMQKLRGGMMTSSSGREVETYDVSASTSSPGTGGSGASANAEPAAGKKSPPVAEDRKRKGRDVAEETECYSEDVEFDYPEAKKQSRGSTSTKRSRAAEVHNLSERRRRDRINEKMKALQELIPRCNKSDKASMLDEAIEYLKSLQMQVQMMSMGYGMVPMMLPGVQRQRFPVPRFPMPQIPAMGPARSQDPMMNSLPPQNANQPRVQFADPYQQYISLPQTQLPQPQNQAGTRTPPVTSMPSSSKDVRDPGHQPTG

>AabHLH16

MNHCVPDFENEEDYLLPASLNSKRHKKSTMGDEDIMELLWQNGQVVMQSQNQRSNGNNNNNKKIETQPPLSTTTAVNHHNNNNRSTVLDDETTPCNLYMHEDEMVSWLHYPSDDNNNLDLYLHNNDILYPVLPAANVAPHSLQGVSAISVPPPRASSVVTRVGEEQNQNKFGNFLHFSKPSFVKNNPTTGSAPTSSNKMSEVTTVVESNNQPMENMKKLRGGMMTSSSGREVETYDVSASTSSPGTGGSGASASAEPVAGKKSPPVAEDKKRKGRDDVEFDYPEAKKQSRGSTSTKRSRAAEVHNLSERRRRDRINEKMKALQELIPRCNKSDKASMLDEAIEYLKSLQMQVQMMSMGYGMVPMMLPGVQRYMPPMAAMGMGMGMGMEHVGMNRPMVPYPGVLPGPPMPNPAAAAAAAAHLSQRFPVPRFPMPQIPTMGPTRSQDTMMNSLPPQNANQPRVPFADPYQQYIGLPQTQMPQPQNQAGMRTPPVTSTPSTSKDVRDPGHQPTG

>AabHLH17

MPGTYVAKPDVVKFSTKPILPSPNALYRKLDLDKAHGHILRYGFLPGYTEWTVHGEHTISLAPSQSSYVNVEETSLGQEDIIGLVRDALGINSLPSDNTQLGDTTMEGDTGESTKADDHGDEGVSYKKLLEECDKELSPNDLESFDICYKTTDDTYIQEATAEMMVIANQEISRKKLELVGPEGNIEPALEAEIAREVLNKLFGNEEPRCFGAGVTKSQITKFCCDLRMMRGEVLANENRFLLEKVDNQSKEIATQKKQLETQKNKVESYSKQVNTLVSQLNNMGQQLNEVYGMLKVFQTAFPDLYNTASTSAAASTCDKQPSSSASPIMDHYSPVMDHYPELIFIKIKAIKNWLTSWLVFMPICSCRNERLYLLRRKTGHQMTKLLITAFSHDWGGDEDIMELLWHNGQVVMQSQNQRSSGSKKLETKPAVRSAEQTAHQTGPSDLFMQEDEISSWLHYPIEYPANENSLEGYIYNNDLLFPTPPPNPVTTAPITPTTLLPPPPPSVVVPSPRPPVAPIWRNRVDIQPQSRQQPKYPNFLHFSRPNKARTLESGPSAPVTEAPESRASRVSEKPPPISAGGESVSGVGLVGTSSMGREVETCDTSMMSSPDGSGASGSIEPSTQMPPPSTNDRKRKGRDTEDTECHSEDVECEYPDAKKQSHGSTSTKRSRAAEVHNLSERRRRDRINEKMKALQELIPRCNKSDKASMLDEAIEYLKSLQMQVQVNKP

>AabHLH18

MEDGGFMTQYDNMCKPYDMVDKLSVDSISSENILEKESSIDRFFQTPSRFEEPTEINLLSYQKASNINRRSSTPNTIAATTHSSFNTFTISFRDTKAKEEIHPSDDSLGYESAGTGKAPIIARTPLQAQDHVLAERKRREKLNRQFISMSALLPNLKKMDKASVLEDATNYIRELQDRVKELEALSDLMRKDTKDILVALKRYRLSRDEEDDSSLNETNSGDHSAGVPSESSAEIEVRISGGSVLVRIYSHKTYSLAVKVLSQMQSLGINIISSSTMPFANTITVITIVAQMDKASVLEDATNYIRELQDRVKELEALSDLMRKDTKDILVALKRYRLSRDEEDDSSLNETNSGDHSAGVPSESSAEIEVRISGGSVLVRIYSHKTYSLAVKVLSQMQSLGINIISSSTMPFANTITVITIVAQIEEDFVMTAADLVSKLQLA

>AabHLH19

MSQCVPSWDVDHENNSRNNSNLARNKVSLRAPSGSISSTLDVPTLDYEVAELTWKNGQLAMHGLGPPRVVNKPHANTANLTKYTWDKPHAAETLEAIVNQATLQPKQKSHINIYSDDLVPWLDHHHNSGVTAGTVSASGTVTMDALVPSSNTQPHALSGTNGAPTNCSTRVGSCNGDQSWYGDHMTAQGGAATHEWSSCRDHSGSGSATFGMESSRQLTVETCERELGPKGFTSTSTGSPENTVSGKQQSTKSTSPDEHDSVCHSRPQKNEVDEKKKGKGKSSISTKRSRAAAVHNQSERKRRDKINQKMKTLQKLVPNANKTDKASMLDEVIEYLKQLQGQINMINRMNMSPMMMPLAMQQQQFQMAMMNPMGLGMGMGMGMGMPGVMDLNSISANRPNIHGMPPVFHPSNFMQPTMASWDMNTTSDQVPNHNDQMAAFLACQSQPMTMEGYSRMAAMFQQMQNQPTYPGLKN

>AabHLH20

MPLSELYKNESSQQKLTDISYIPNDEFVELIWEKGQVMMQGQSSKARKTPVSSNFQFHAPKVQGKDGMLNVPMAEIGLDQDDDMVPWLNYPLDDYCADLLPEISGVTVHEPTMHNGMSVIDKRGNKDTSVFNGLDQANTSKGLKVSNLFSWPDPMVRSGITDIGSSNCRSKVDNVVHRDPIQIQGSAGRVEKIAQKQDSSSTLLNFSHFSRPAAMAKTNLQNTAVNVVNHGSQKEIGFVSQPNLDSVGVGSNPFVSKPLNEPHSVEKSSNVVLDVNGAKGVQETVKSNEPVVATSSVCSGNSAERASNDFSKNSKRKSRDTEEFECQSQDVDEESLGTKTASASRGGTGSKRSRAAEVHNLSERRRRDRINEKMRALQELIPNCNKVDKASMLDEAIEYLKTLQLQVQIMSMGSGLCMSPMMFPGGMHPHFSPMGIGMGMGYGIGMGMEMNQHGHPNMFQFPPNAQGSRHPLPSPTVYGHPSQGMPMLFPQPPMPRVPMHPAARPVDVAPSSKDPMQTKNSHAMTQLSNQSQDAHKTLNQSTLVDKKDQGLEAGCSTAGID

>AabHLH21

MQHFEHENSTSSGIAANGSTGDDTVVAKKINHNAGERDRRKRVNDLYSYLRSLLPISSYHKKKVSIPETVSSAVKYIPELQKEVERLKHKKEKVQWSSSPTINAKQEHLAIKKKSCKTKTNSFLVSSVNVLGDKEVVIQLISSTDHTSTNKEIGFLSKVLENLEHDEDGFVLLNATTMKCSGEGMVLNTLHLQVQGDHKIGGEKLKEQMCSFYQNIYGTLL

>AabHLH22

MEDIGDEYKHYWETNMFLQNEEFDSWGGLEETFSGYYDSSSPDGAQSSAASKNIMSERNRRKKLNDRLFALRAVVPNISKMDKASIIKDAIDYIQHLHDQERVIQAEIMELESRKLESGVLEYDQEMAFMSTENSKKKKIEQSFDSSESRAYPVEILELKVSYVGEKTVLVSLKCRKSRDTMVKICEVFETLKLNVVTANVTTFSDTLFKTLFIQADEEEIDLLKIQIHTAISALNDPPSPMST

>AabHLH23

MHPHHHNHQSSWTTLSQPFQQYTFNHVLPQSSLPNGGLFKRVVDGLQFAYEGSSSSTTSPSADHYLGFQPGSNTLGIQTDTTKMTTQEMADAKAIAASKSHSEAERRRRERINNHLAKLRSLLPNTTKTDKASLLAEVIQHVKELKHQTSIIAAQIPVPSEIDELVIDNTSDEEGRIVIKASLCCDDRSDLLPDLIKTLKLLQLRTLKAEITTIGSRVKNVLFLTGEDHMNSNEDEKVVNHWVYSITEAIKAVVEKTNDGNDCSFASIKRQRTNNIDIFNHRRRL

>AabHLH24

MSPAWLSEMEIEDPGFMNYDQMSRLCDTSLDSFSPESYAENMTFIDQSFQAPQLEIEIPNYQEKSSSIDKCSPSPDTLVATTLPSSNTFTISFGDLKPKSEILQFSDPLPGAIKVPTILRNPLQAQDHVLAERKRREKLNRHFISLSAIIPNLTKMDKASVLEDATKYIKELQDRVKELEGSPSTKRKHVQESVIYVKRSRRSASDDQYYSSDDTNSEESTAPYKTSPEIEVRMSGSSVLVSIKCHDNISSLTKALDHMQKLGLSIISCSSMPFAKTTLLIGITAQIEDDFCMTTTELVKNLQLAL

>AabHLH25

MCSKREEQELVEEQQLQQEGDHQEHPSLIIPQTQHQNMGIVDYSYTSSSEVSPILHPQQQPWIIPQVFNHHNTACTDLPFTDHGNYIFPPPPPPSSLTSSYGGLVNRRVPSGLQFAYDGSTSSSDHHLRLISETLGQMVQPGSMPFGLQAEMGKMTAQEIMDAKALAASKSHSEAERRRRERINNHLAKLRSILPSTTKTDKASLLAEVIQHLKELKRQTSIIAEQSPVPTETDELIIDNTSDEDGNLVIRASLCCEDRSDLLPDLIKTLKALHLRTLKAEITTLGGRVKNVLFITGDQDPNNNDSNTNYSINMIQEAFKAVMEKTNGDHESTSGSSKRQRTNSIHVVDHRRT

>AabHLH26

MHQQQPTPPLVPMLADYGVAEITWENGQPAMHGLGRANETLESIVHQATTCYNQTQYPEIDLQQSQSLPRARNLSSNVASSSRPTYLRKRPRESVIIHDQCVGNLGNASLQEDNVSNSGTVNSKDNDTTMMTWPSFDSPNQSMKSQKTDDDSACQYGSENQEEECRTEGETIRSQSSRRSRAAAIHNQSERRRRERINQKMKALQKLVPNANKTDKASLLDEVIDYLKKLQSQVQLMKNMPFTPQQMMMSMPLQLQQQQHQHQQQQQLQMSMLARMGMGFGLQMGMPGVIPQPVHNPFMVPQTMLSPAHVGTTSQTIHSRPSTNTPVPFNDPHSTFLAQQMNMDMYNNMAAFYRQQVNQGKSMSVDSSQLDHVRGE

>AabHLH27

MLVDPEMYEGSACYDPTHLESLIDHQDDNNMSQAHLHNYHQAQNFNSLDQQQDYHMINMEMEHQNQIMQDHLNWSTTHSHEQIHIENNNTSSIPNMPLITPNPTPPDLLNLFQLPRCSNSSISFSNPTHMDQTSYDPLLPLNLPPQPPFFRELLHSLPNGYNLTGGGSIFGEMDMERDHQLYHHEGNGILKFSGADISGIVGKGRDVKDTKHFATEKHRRQQLNDKFDALKNLVPNPTKADRASVVGDAIDYINELKRTVEELTILVERKRCSRGRMKKHKTEDDSTLDVESINTRPNGGGDQDQQGYNGNSTSTLRSSWLQRKSKNTEVDVRIIDDEVTIKFVQQKRINCLLFVSKVLDELQLDLNHVAGGLIGDFYSYLFNTKICEGSSVYASAIANKLIEVVDRHYASIPTPSGY

>AabHLH28

MEEQARKKKNDSNKMLNTYVLEKDNSLNVGGKDRMEGHVVSNDVNKTDSVNGTNGKVDNDLATGSDVNKSKVAVVDKSKEQGNMEKCESVDEHDEIKKDDRKTYASATYDTKLDMSRKLFEVPTEVDENGYELGVGRVGYARVMVEVSAKKCLPDIIEMIYRNKNGGEICRKTVNVVYDWTPPRCSHCCVFGHSDKMCKVCESNEEPKDANNTVEVSAEKENKSEEGKENVEKKNDGFEEVRYKKNNGGNKGKGHNNNNGQRKNDGKQNVQQNQGVYQKKVNKEQGESSNSNKTTKSPVKSPVKAPLNNNNVPGTPNSRKAWKVQGEILEELKRSANKFAGLEVPDDTGCLGNNECSMDLKIGCWNIRGLSTTDKQNEVRKYIDDERLHMCGIIETQLKTKKLQKIGDSVFKNWSWVNNMRMCDKGCRIMLGWNSDIVNVNVIHYSKQSILCKVEIVTGNMALFCTIIYAANSGNERKDLCLLVLFDSMNAQGSVMDADFCSPESILSSSGICFGCAEFLIPQPPPPPPLSCYGGLFNRRLPLQFAYEGNPSADHHLRLLSETLGHVVQPGSGPFGLQAEMSKMTAQEIMDAKALAASKSHSEAERRRRERINNHLAKLRSLLPSTTKTDKASLLAEVIQHVKELKRQTSIIAEQCPVPTETDELTVDNASDKDGKLLIKASLCCEDRSDLLPDLIKTLKALRLRTLKAEITTLGGRVKNVLFITADEDHLNGNDDQQMVNYSINTIQEALKQVMEKTNGDDSGSVKRQRTNNINILEHHRSL

>AabHLH29

MDFPPNCFKGFTSSEEHVLKEMMMMSRVRTTSSSSSLVLDNEKGEIVRALVTPGNIRHTFHDDHDHANLQKGAKGEKALMALRNHSEAERRRRERINGHLSMLRSLVPGTTKMDKASLLAEVISHLKHMRMTTTEATKGVLIPMDIDEVKVEQQDENSLDGSSYSIRASLCCEYKHEVLLDLKEALDGLQLKTIRAEIATLGSRMMNLFVITGSKDEVNIKDIVSSIRQALKSVLDKFYASQEFSESNSLSNKRRRVSFFTPSSSSSLGDFW

>AabHLH30

MGIKDEQSDELVFHQIPSLICFQQPARNQQDLIVEDKDHVTMEGNTNKSASKTRKKQEDQSPSSSKPNSATPEDSTKDEHTQRKLVHREIERQRRQDMAKLYASLRGLLPLEFVKGKRSTSDHMHQAVNYIKHMQENIKEMTVKRDQLKKFVEMSVSGLGTNSNEKNLTNLLPNTVSLHSSNGGIQISINSCPFEEGFPLSRILKAISKEGFKVITCTCTKVNDRLIHSIQAEANDPVLTDLSMLQQRLTVAANNY

>AabHLH31

MELYGWTNPGFSNLGPNGFDGSLMNSSAFNEDYGNVDGLFMKSSESLVLDNEKSELVKGQSKVIGKKIGSISDEKAVAALKSHSEAERRRRERINAHLDTLRGLVPCNNKMDKATLLAEVIRQIKQLKVNATQASTGLLMPEDVDEVKIEKLDQLSVNGSIIFHASFGCKHRPELLTDVRKALVDLKVHMERAQLSYLGDHVKIIFDFMANEDLVTSVREALTAVIEKGSISPEYSLRTLPNKRRRYCL

>AabHLH32

MVKSLIDHFPVPGGELPSLEPGFHWSANSFPGSTSVVSKGFSDSYGQSDATKEDESRKRAHPGSCSSTKACREKKRRDKLNERFQELNEILDPGRSAKTDKTVILADAIRMITHLRNEATNLKDSSQDLLVKINELKVEKNELRDEKQKLKTDKERLEQQLKSTFCGPPTAFYPPAHPVMPVPCPGPTPVGGNKFMPYMGFQGVPMWQFAPPAAVDTSKDHVHRSPLA

>AabHLH33

MLALSPTLFSTNYEWPLGDNLDRNHQQDCNNISMDVEANSYDSVLDFPTYDQIRQDFTPESSNSFGGAINGNTGNPMKVSKKLNHNASERDRRKRVNDLYAYLRSLLPISADQKKKVSIPGTVSRALEYIPEPQKEVETLIRKKEKLSSYSTSSASTSQKNIGIEGQSSKDVTANTNSSVVSSVSILGEKEVVIQLIFSMDRMNKNKEIGSLSRVLAYLESEENGFVLLNSTTFKCSGEHMLLNTLHLQVQGDKKVDVEMLKEKLCHFSQ

>AabHLH34

MENITDVNWESILQQEDYSWGGLEETFSGYYDSSSPDGGQSSSPASKNIVSERNRRKKLNDRLFALRAVVPNISKMDKASIIKDAIEYIQLLHDQERTIQAELMELESQKLEPENFDFNQETKSSMSMEKSKKIRVEQAFDSSGSSSYPIEVQDLHVSYMGEKTVLVNLTCNKRRDTIVKLCEVFESLKLKIVTANIYAFSERLSNTLFIQADEEDTDVLKIQLETAISTLNDPHSPMRIKKGAKGRLVIISSRNYSWCN

>AabHLH35

MLRNPLQAQDHVLAERKRREKLNRHFISLSAIIPNLTKMDKASVLEDASKYIKELQDRVKELEESPSIKRNHVHESVISVKRSRLSASDDEYYSSNDTNSEESTAPYMTSPEIEVRMSGSSVLVSIKCQNNISSFTKALDHMEKLGLSIISCSSMPFAKTNLLIGITAQVGGQVRPPRGRRPCRPAGGAGRPRGRGRRCAAGGGAPAAAVTRRRARRPYLLSFRDSKPKSRDPSSFLDLACLAAIKGSTHAQESTSGTRIMFLGRRGRRREKVEPFLGSAIIPNLTKIEDDFCMTTTELVKSLQLAL

>AabHLH36

MHFSNARGSPSYQCINRPTHRPHNFHLTFQQLFPLINKKQKMLAVPSTLFSTSYGWPLEDNIAPNHQQDCNDVSIDIEANSYISLLDFPLYDQSQHDCAPESCSSGGAINGNIGDPKKVSKKLNHNASERDRRKRVNDLYSYLRSLLPISADQKKKVSIPGTVSRALKYIPELRKEVETLMRKKENLSSYSTSTIARQKNLDIGRQSTKDAIINTNSSVVSSVSVLSEKEVVIQLSYSTDHMSNNKEIGSLSRVLEYLESEENGFVLLNLTTFKCPGEETLLNTLHLQVKGDYKIEAEKMKENLCSLYQQSYA

>AabHLH37

MQDFATENSMSSGVDANGGTGDDTKVAKKLNHNAGERDRRKRANDLYSYLRSLLPISSDQKKKVSIPGIVSHALKYIPELQKEVVTLKRKKEKAQSSSSQTMNSSWQEHRAIKKENCNGTTTNRDSWLVSSVNVLSDREVVIQLISSTDHMGTNKKNGFLSKVLDNLERDENEFVLLNATTMKCSGEGMVLNTLHLQVQGDHKIASEKLKEHMYSFYQKVYETLL

>AabHLH38

MDMPSAWLPELEMQEQGFINQYQMNKPYHPLMDDFSVDSFSSESYTENPSFIDQSFQTRKGVEEQADIKQPSSYKKANSINNKFTPINQIPKPKRVSDPPNTFTISFGDIKPKDEILSFSDSYDLTGDGANKVPAMIRNPIQVQDHVLAERKRREKLAQRFISLSSLLPDLKKMDKATVLEDAANYIQELQGRVKELEELSGLKRKNMQESVISAKRSRLSCSDDDGSSSNTANLEESSSPINPEIEVKMSGSCMLIEIYSHKNCTSLMKVLSEMQRLGLSVTSSSTMPFADTTLLITIVAQKSDDFIMSSTDLVKNLKLVI

>AabHLH39

MDMSQAWLAEMEMEDPGFMSYDQMSILSDVVNNFSVDSFYSEIYAETTTCVDKTFQTQQPEIRQETSSSISKSSAPLDPLVTNSLPSSKTFTISFGDLEPKEETLQFDDSLGYEDARTTKVSITLRNPIQAQDHVFAERRRREKLNQNFISLSTVLPSLKKMDKASMLEDAFNYIKELQGRVKELEGTLKPDNKRENVDQESDISLKRYKLSNPCDKTRSEKSTSPCNTSPEIKVSISGSSVTVTIQCQNNSSSFVKALTQMQKLGLSIIYSSAMPFVTTILLITIVAQIVDDFSMTPTELAKSLQLAI

>AabHLH40

MALETLSSNELLNFIIYDTISASPFTFNDSSSQNTNNSNTFFYNLHNQNPNPNPNPLSQELEGADMEIMSSNSSLATTTEKMSLAVQAYCGGKNPEKNYCDNNNNNKSIHNNLGVQKKKRRRRPRVCKNKEEAESQRMTHIAVERNRRKQMNEHLAVLRSLMPESYVQRGDQASIVGGAIEFVKELEHLLQSLEAKKFVMTQQPQEDDDNGGHDSNFTKLSSAAPPFSQFFSYPQYTCSQIPNKYTSKSKAAIADIEVTLIETHANLRILSQKRLAQLSKMVACFQTLYLSVLHLNVTTMEPLVLYSISVKVEEGCRLNSADEIAGAVHQMLRIIEEEATLCVVDSR

>AabHLH41

MERNELELLNLASNSAQLEFYKEAGIKTAIFMGSSNGEIELGITYDSSQKLFPGDFPQGVLPQRLEQARASSFLRSLSMENSVENSPFLFNMLHSTPYMSEMFALTEPLMDQQAPNQTPQSTTILRSEDPLQQALEQIRISRYGSSLGPNKRVQNRQNLHRRSLSFFRNLSEARAQRDQMVQTTRPTSNQFLHVIAERKRRGKLNESFQTLRSLLPPGFKKDKASVLLNTMKYISSLKSQVKELNKRNQILEADRRAGKEPPNQGSSRFSGEGPVVGITDIGESTSESRVVDLDVNARGNVVLVDLVMSVLEFIKQTENISVMSIDAGIRMLDTEPIANRVILRLRIQGNEWDKSSFEEAVRRLLDDLTQ

>AabHLH42

MDMPSGLWLPELELEDPRYFMNQHQHTCTYSELVDSFSSQGFKGYIESNRATTVSENLDKQESYKASRTNPFTPIGGSTANTFTISFGDHNEINKNSLHGGFQLKYNDAIKPKVEMSLNELLDSIEIPKRVPSTRRNHRQAQEHVLAERKRREKLTRRFISLSTLLPEIKKMDKATVLEDASKYIKHLQNRVKELEQTSVGEKHIILESITSTRSNFRSSHEDNASSYDKINSLPFSTDNDPGIKVRISGSHTLVRIYCQRNSSLALKALIEMERLQFIIMCNNVLPISGNAALITIIAQMSEEFEMTATDLVNCLKSSL

>AabHLH43

MEPSSWLPELEMQDQGFINQYQMNKAYHPLMDDFSVDSFSSESYTENPSFIDQSFQTRKGVEEQADIKQPTSYKRANSINKKFIPIDQKPKPKLVSDPPNAFTISFGDIKPKDEILSFGDSYGFTGAGSKKVPAMIRNPIQVQDHVLAERKRREKLAQRFISLSSLLPDLKKMDKATVLEDAANYIQELQGRVKELEGLSGLKRNNMQESVISAKRSRLSCSDNDGSSSNETNFEESSSPSTPEIEIRTSGCSLLIEIYSRKNCISLVKVLSEMQTLGLSVISSSTMPFADTSLLITVVAQVYS

>AabHLH44

MLDNSDMFEPNTYNLDPNTFSHLIDHNHNLNNQEQQLNWTNNLNNLDVQTNQPDLLNLFQLPKCSSSICFSNPSHMDQQVMYHDHHSTLLGMHNSNSTNVGVTQNVPYIRELLHNGFSLNGCNSSIFGEMDMEHNGGCDGILEFGKNGNGNKDNVKHFATEKHRRQQLNGKFDALKSLVPNPSKPDRASVVGDAIQYIHELKGTVEELKHLVDRKRCNRGRMKKHKTEDDSTLDVESIYTMSNGVVGDTTHDQQAYNGNSSSSMRSSMVQRKSKHTEIDVRIIDDEVTIKLVQQKKINCLLLVSKVLDELQLDFHHVAGGLIGDFYSYLFNTKICEGSSVYATAIANKLIEVVDKQYAAMPVTSSY

>AabHLH45

MSMAVQAYCGGKNPEKNYCDNNNNNKSIHNNLGVQKKKRRRRPRVCKNKEEAESQRMTHIAVERNRRKQMNEHLAVLRSLMPESYVQRGDQASIVGGAIEFVKELEHLLQSLEAKKFVMTQQPQEDDDNGGHDSNFTKLSSAAPPFSQFFSYPQYTCSQIPNKYTSKSKAAIADIEVEEGCRLNSADEIAGAVHQMLRIIEEEATLCVVDSR

>AabHLH46

MQPTTGGGGGIGLSRFRSAPATWLEALLESEEEDVIIDPPKPPLTPPPHTHPFHQQHSTGPTSRPPATYVDPSSTMLLPPTSSGGISVANRQNSLPAEFFSQINAPGDGTFVSGYSNSGYDDYVSPSGLDGQRAKFTTQLSGDQSALLDAEMDKLLGESVPCRVRAKRGCATHPRSIAERVRRTRISDRIRKLQDLVPNMDKQTNTADMLEEAVEYVKFLQRQIQELREHRDKCTCLVKA

>AabHLH47

MDNVVWSTSWTQDGGVFDQFNNWDRANLLVPNHKGAGSSASKNHSEAEKRRRDRINAHLATLRRLVSNSDKMDKATLLGKVVERVKDLKLEAVELGKVFTNRVELFSEIKDALNSLGLTMVQADMTCLGGRIICNFILCLTNNTSENEVTSIKHSLKILLNRIVSTSSWTMSSNYRIKSKRQRFFCSSSYDTNGCE

>AabHLH48

MNNNHEYETNNMNSFNSLHELEENSNNWYFNHHENNLSFAPSLPSTYQNFDTKTRFDSQPHMDVPNIISNHHFPTNHLENAFGASGFQGLGLMGSSSNTKSGDGPNTLVMDDVDDISFDESVLNYESDDVARKMDSGVGNIGGYRKGKRKGFPAKNLMAERRRRKKLNDRLFLLRSIVPNISKMDRASILGDAIEYVKKLLQNVNDLNLELESTPSTSLMPPVVAPTTTTGLYQLTPTATIVPSCIKEEVFQTGQPIKIEVRQREGGVLNIHMFCSQKQGLLYSVMRTLEDLGLDIQQAVISSFNGFALDVFRAKYSVFCSITLKAKILTVITSRRCCVKQLNIMGSCRCGSLPSVEASTLGV

>AabHLH49

MISECTFLPDLIHTYVDQFGETNFLSPETVSADDVFRGFVADLEGGSDKFAVMTSVGRLHEAVEGRKRKKMKMTAVEQDGVGCGNSDGQQKVSHIAVERNRRKQMNEHLSVLRSLMPCFYVKRGDQASIIGGVVDYITELQQVLQSLEAKKQRKSYNELVMSPRLLASPRTLPISPQKSQFSPRPISLPISPRTPQPLSPYRPMPHLPFISPSLDYPSPSNSFTTSESGNELMANSRSTGAEVEVKFVGGNLLLKTCSNRIPGQATKVVAILENLSLEVIQANISVVDDTMFNSFTIKVGVACKLSADELAQHIQETFCTGKTTLSTDHNRYLIGDDEHCWSDKGVSNIEGGCYAKCIDLSREKEPDIWNAIKFGTVLENVVFDEHTREVDYLDKSVTENTRAAYPIEYIPNAKIPCVGPHPKNVILLACDAFGVLPPVSKLNLAQTMYHFISGYTALVAGTEEGVKEPRATFSACFGAAFIMLHPTKYAAMLATKMEKHGATGWLVNTGWSGGSYGSGSRMKLAYTRKIIDAIHSGQLLNANYKKTEVFGLEIPTEVEGVPSEILDPVNTWSDKNAYKETLLKLGGLFKSNFEVFLDHKIGKDGKLTEEILAAGPNF

>AabHLH50

MMQYLPSHGYEFANCASIIRNLVYDHGAKDLSNDTEANVAAEAEAKAAAACNRHSEAERRRRKRINGHLATLRSILPTTVKTDKASLLAEVVRQVKELKKMAAEIESAAIDQGDDIIRNNEYYMIPTEKDELELTYVGEDSSTKKIMIKARLCCDDRAELIAELTRALSLVHAKLVRAEIGTLGGRIKCLLWVQVSTVTIDQGIHELQRSLKVVMDRATLLDMPRNKRPRSFSDSVEF

>AabHLH51

MYGVNNSSDAISRDMNSILYSSTFKHPADTEFAKIKQLISLDNNNNSYENPSTHPQHQENSENPPLVSYRSTPSSFFSNLLNENENDAFQDHEPEEIYFMDQQQHKKKSDQSEAYNNMKREKQEMGSKNIEVLGYGYSKQSDLDCGSSFRSDLVRQSSSPAGFLSSLTGENAFAKDLRNGSSSSSFNSHISFSLGSSSSSSRFLPQIAENENELNDSTFHSLKRSRDGSLKMSQNGETVNHTPNLVHHVSLPKTSSEMAAVDNFLHFQQDSSVPWKTRAKRGFATHPRSIAERVRRTRISERIKRLQELFPDMDKQTNTADMLDMAVEYIKDLQNELQTLNDARARCKCSKEQLQSGSTM

>AabHLH52

MDPNLYLIWDEDEDDVKESGDGDSSETVTTRNPNTQRRGGGVKGDRTKSLISERKRRIGMKEKLYTLRTMVPNITKMDKASIVGDATRYIQDLQTQARNLRSEIAKIEVNSNHKKASQNSKMANVSNSLPIFKKISKLEMFHVEEKGYYVKVVCNKGRGVAVALLKALESITSFQVQSSNLATLGDTFELTFTLKVAAREFDKKPPNLKLRLSGAFLKQGFKFK

>AabHLH53

MLEDYSTILEQVDAKVHEEHQVVVSDNQIPNVFNIGEKNSKSKNVQGQPSKNLMAERRRRKRLNDRLSMLRSIVPRISKMDRTSILGDTIEYMKDLMEKIRSLKEQDTGLDSSGLNELKVNQTQERNSPKFEVERRNIDTRIQICCAPKPGLLFSTVNTIESLGLDIRQCVISSFGDFTLHASCYEAPENQVLASSEEIKQILFKNAGYGGRGL

>AabHLH54

MDIPFGAWLPDLEMEDPCYFMNQHQHTYPYSELVDSFSSQGFKGCMNLVTRSEAIHAATISKELDSQEIYKATRNNSFTPIGGSSSNTFTISFGDHSSSSEINQTTLPGGFKLKYDDVMQPKLEMSLNELLGSIELPKRASTTRRNHRQAQEHVLAERKRREKLTRRFISLSALLPEIKKMDKATVLEDASKYIKYLQNRVKELEETSVSGKNVIQESTTSMRSKFYGGHEDNASSFDDTNSLPISTANDLGIKVKISGSHTLVRIYCQRNSSLALRAITEMESLHFSIMCNNVLPISGNTALITIIAQMNEEIEMTAMNLVNCLQSSLSNFL

>AabHLH55

MKQSEMGGIKGSGSGEQEGVAITVTSGSKRSSDAFEDDVKINRPLSDHDLHILTERERRKKMRNMFHQLHALVPHLPHKTDKSTIVDEAISYIQTLEETLQKIETKKLEKLYGTQSAANSTTVSPIQSPKPALDTRESFLADQGSSTVSPSSSSTFSFPISSPTVFQTWASPNVTLNVCGMDAFFNICSFPKRGLFTAICFVLEKNKVEMVSSEIYSDQCKCSFFIHAHVNARDQTVKDLSFEEIYKQAAMEIMRCVSTKSP

>AabHLH56

MDSDLHHQLNHLPQQQTMNTPGLTRYRSAPSSYFSNLINSGIYDDTDQFFNTRVSSSSTTDQILSGYESRFMMDSVKQEQEVIYSTPQPSTMNYQQQQQQQQQNQFQSQMDQQTNNSNTSGMDSSNNIRSNNLLRQSSSPAGFFDQLDMDNGFSAMRSLDDFKVGGRGIGNSMLSSTKRMKNEMGFTSSSLTSSGILPRIPENESKVMDIKSGRDDGGFGSQSWDDSDILSDSFLKDFGEIDQSKISSLKPSENQNDVGRIRAPNTLVHHMSLPSSTAELDKLLQFQDSVPLRSRAKRGCATHPRSIAERVRRTRISERMRKLQELVPNMDKQTNTADMLDLAVDYIKELQKEAEALSDHHAKCTCPHKEKL

>AabHLH57

MQALSPTLFSTTYGWPSENNITQNHQQDFNDIYMDVEANSYSPLLDILSCDQSQQNCAPNSCSSRGAIDGNTADPMKVSKKLNHNASERDRRKRVNDLYAYLRSLLPISADQKKKVSIPRTVSRALIYIPELRKEVETLIRKKETLSSHSLSTTSTSPKNLGIKRQCDKDVNINMNSSVVSSVSILGEKEVVIQLISSTDQMSKNKDIGYLSRVLAYLEAEENGFVLLNSTTFKCSGDEMLLNTIHLQVQGDNYKVEAEILKEKLCRLSQQTNELSP

>AabHLH58

MSNGASGGASLSSHENSSQIRVKESRFLDHMKHGQPSFTTQQHVMYQNHAQSKQGVPSSSSGFSPMSNNLIRQSSSPAGFFEYVNIDDGYSMMRAIDKYRHANGSVQDSQMAKSRMVFSSGSHSLSRIPENEGKGIANSNGGYVDSFGGGSWDASVMLNDGFMKEFGESDGISSFDNQNDEGQIRVSNGLSHQLSLPTSSTELSEMEKLLQLQDNVPLRSRAKRGCATHPRSIAERVRRTRISERMRKLQDLVPNMDKQANTADMLDLAVDYIKELQKLSEKLSDHHAKCMCPHKRKL

>AabHLH59

MSGDCTSLTDLFDTYVEQFSDTSFLSPTAASSDDIFSILEVLEGVSDDFKSMAPMKSGPFHVKEIKHPLVSQKSTSSCSALQELVEENIETPNSSKRKRQKLSLVEEGGVNSDGQTKVSHIAVERNRRKQMNEHLIVLRSLMPCFYVKRGDQASIIGGVVDYITELQQVLQSLEAKKQRKVYSDVLSPRPALSPRKPPISPRPLLPISPRTPQPLSPYGARSPAAYNSSYMSSSQSSMSNNMIPHSFDPSPSSSSSTSDIINELVANSKSSIADVEVKFSSGSNLILKTTSPRLPGQATKIVSVLEELSLEILKADINTVNETMVNSFTIKIGIECQLSADDLVQHVQQTFC

>AabHLH60

MEITQFRGFSELSGIEDPCFNSQWPFNSFDDQLMNVDACNSFAYAPMFDHIHYKPIMEASPRPSKQLKTSSWNSSISSDQSMMNQTIHYGSDHSHVANQATLMSPKEEATLSSSSVFQSSSHVQFGNQNHHAKKINSSKFTTPQDHIMAERKRREKLSQKFIALSALIPGLKKMDKASVLGDAIKYLKNLQEKVKTLEEQTKKRSNVESVVFVKRYEVLGDGGEISSSGENSQEQLPEIEARFSGKDVLIRVHCEKKAGIVEEILAEIEKLHLSVINSTALTFANYALDITLVATMDQEFTMTMKDLVKNLRIGLKRLM

>AabHLH61

MLCYNEFELFQGGFLGVDFSGEHVQDHEHQNQQQFMVKNTYENGNQMVDYLLNNPIQNLPSSGFCSSNSFDKLSFADVMQFADLGPKLALNQNKTSHDHEEEQENGIDPIYFLKFPVLNERSQEDHESLLAPLGDEENEHRLVIEGGERDEEARVSEGNSVRLQFMGEDVHKTLGTEGKNKRKRPRTIKTSEEVESQRMTHIAVERNRRKQMNEHLRVLRSLMPGSYVQRGDQASIIGGAIEFVRELEQLLQCLESQKRRRLYGDTPRVAGDSSSLPVVQQGPPAVFYPPSDEQMKLVEYDGGLKEEMAESKSCLADVEVRLLGFDAMIKILCRRRPGQLIKIIAALEDLQFTILHTNITTIEQTVLYSFNVKVSSEARFSAEDIANSVQQIIGFVHANTSS

>AabHLH62

MVNESTSLTDLFDPNGSLAYPDDIFNILEALEGVSNEFTVSTPLADSSSGVQELVEAEHEAFSPRNKRQKVSGDEGCENFDGLVKMNHVTVERNRRKQMNEHLTVLRSLMPCFYVKRGDQASIIGGVIEYITELQQVLQSLEAKKQRKVYSDHVLSPRLISSPRTTLPLSPRKPPLSPRPISLPISPRTPQSASPYRHRLPSATSYLNTRPASPCNSSSNSDTINELVANSKSSIADVEVKFSGANLLLRTLSPRLPGQVTKIISVLEDLSLEILQAKINIVNETLVNSFTIKIGIECKLSAEDLAQHIQLTFC

>AabHLH63

MLPENDTAWMDNHQDETSSWTPTTTPNHHQPPCTNQNTYTSLTTLKNMLETEWFQNNNNNNLNLTSTDTNPMFLPLDSSSSCSPSQSHSHSHFTQFPFIPTKYSNNHSTNVNLTSFDLPFEFGCDPSFISNNPPTNFLNFPTQQMATSSEYTETSYHDDLTGTGTGFTGFEPVNPVFPVRSKVLRPLEVSPPVGAQPTLFQKRAALRQSGEKLDGLEIVKKRKRGSGSGSGPHEDEIDEMGNGDGSGFNYDSDEVELTGEVNGNGNGGNSGSVMVGDSKGKRKGLPAKNLMAERRRRKKLNDRLYMLRSVVPKISKMDRASILGDAIDYLKELLQRINDLHNELEATPQGSLMQASSSIHPLTPTPPTLPQHVKEELCPSTLPSPKNHPSKVEVHAREGRGVNIHMVCGRRPGLLLSTLRALENLGLDIQQAVISCFNGFALDVFRAQQCREGQEMLPEQIKAVLLETAGYHGAI

>AabHLH64

MRGKTVVLCKMLGQNSSSSCSPSQSHSHSHFTQFPFIPTKYNNNHSTNVNLTSFDLPFEFGCDPSFISNNPPTNFLNFPTQQMATSSEYTETSYHDDLTGTGTGFTGFEPVNPVFPQMATSSEYTETSYHDDLTGTGTGFTGFEPVNPVFPVRSKVLRPLEVSPPVGAQPTLFQKRAALRQSGEKLDGLEIVKKRKRVELTSEVNGNGNGGNSGSVMVGDSKGKRKGLPAKNLMAERRRRKKLNDRLYMLRSVVPKISKMDRASILGDAIDYLKELLQRINDLHNELEATPQGSLMQASSSIHPLTPTPPTLPQHVKEELCPSTLPSPKNHPSKVEVHAREGRGVNIHMVCGRRPGLLLSTLRALENLGLDIQQAVISCFNGFALDVFRAQQCREGQEMLPEQIKAVLLETAGYHGAI

>AabHLH65

MLSRVNSVSWMNDDENNQTNNNNNNNNKSLLDDVQLESNNNNTNISTQNNWFSTSSSCSPNSSFQNHPFYIPPKPNNTISSLFNPNHNPLDETFNMLPNFCDLGSQTQTHMGINNLTPSLPFPDTLLAQVPGTGFGSPGYPFSDNGSTAGLMLNRSKMLKPLEGFTPTGSPPTLFQKRALKRNSGELSGAGEFSGTGVLGKRKSGSWDEVSLDGSGLNYDSDEFTAVATGGNGGGGGSAGGGGGGKGKKKGLPAKNLMAERRRRKKLNDRLYMLRSVVPKISKMDRASILGDAIEYLKELLQKINDLNQELEATPASSSQTPTAAATIAAGPPGFYPLTPTPTSLPTRIKEEVCATIMPSPTGQQPARVEVRQSEGRAVNIHMFCSRRPGLLLSTMRALDNLGLDIQQAVISCFNGFALDIFRAEQCKEGQDVHPDQVKAVLLESAGYHGVT

>AabHLH66

MHQPNSQQGLLEDLMAPTSEAWSTFSNLQELNTFDTQLFPTQLDDDLLMLPPSSSSYSNSSFLELLSSSIQPSFQSPYTDESIYSSTPCFDSFLPVNGSVSSSYDQQMFPFMAEGEDNDGDEDHKDVVVSHDQLPTVFNMGANGEKKSKSKKVEGQPSKNLMAERRRRKRLNDRLSMLRSIVPKISKMDRTSILGDTIDYMKELLEKIQDLKEQEAESDVDQLKLEGSFREFNMNESQVIRNPPKFDVERGNINTRIQVSCSTKPGLLLSTIDTLEALGLDIQQCVISSFNDFSLQASCSEAQDHRSMISCEEMKQILFRNAGYGGRCL

>AabHLH67

MEFHHGFLEEIYNSNFPFGLTKDIDDHFDQNTLFSTPSNFLETSPLSITSQNSCSNFEDFTLPCNFDPQTLNSSSSYGQHFMNPFIDYTPDIKLESSLLNDQDYYNVFSMLDDVQNCHFVHDMSNVPNPEMPGLQGVTVESELQRPGSFNIGKNGKNSKVEGQPSKNLMAERRRRKRLNDRLSMLRSIVPKISKMDRTSILGDTIDYMKELIEKIKHMQEEMAISSNDQLNSLNAKPKETYIRNSPKFDVERRNTDTQVQVSCTGKPELLISTMTTLETFGLEIHQCVISCFNDFAMKASCSEEMEHGLIIDSDDIKQALFRNAGYGGKCL

>AabHLH68

MESYGNVFNEEWMNLSSMFSCDQNSDNFMGHELFSNEYEHGLNPSILWQSSNEYNSSNSSVVDDQINLAYPSDHDLKSNFHNYFSQESSNSTDSPPNPSHDIFQFSAPYNTLPDNVSNQSNDVSVMEGDYTNFFLLAQVFSDEAMEDILCLKQDEVAERGKKENSAGQHVPNIGDVGRETFLKRKYEMLELPILLKEGNTGRMDENPKKKSRVSRDNKSTKKSPPKKNQKMTTTINRNEDDHGSKENDQININGRGDVQSSSSCSSEDDSNISQKLQEETNNSSAKTRASRGAATDPQSIYARKRRERINERLKTLQTLVPNGTKVDISTMLEEAVHYVKFLQLQIKLLSSDDKWMYAPIAYNGMDMGLYQNISQNAPFQNRSHSSFHIRLLGEKVAIHHTWKWFGHYNLDVFLSNGLGKGI

>AabHLH69

MDLSSMWLPELEMEDGGLTTQYDNMCKPYDMVDKLSADSISSENILEKESSIDRFFQTPSRFEEATEINLLSYQKASNINRSSTPNTLAATPHSSFNAFTISFGDMKSKEEIPPSDDSLGYESAGTGKAPIVARTPLQAQDHVLAERKRREKLNRQFISMSALLPNLKKMDKASVLEDATNHIRELQDRVKELEALAGLKRKDTKDILVALKRYRLSRDEEDDSSSNETKSGDHSAGVPSESSAEIEVRISGGSVLVRIYSHKTYSLTVKVLSQMQRLGINIISSSTMPFANTITVITIVAQIEEDFVMTAADLVSKLQLA

>AabHLH70

MDSSSTFRSMNSNNNLIRQSSSPAGFLSALNSENGFASMRDVDKSIPSSRLNNHISFSSDKSSSSMFLPQIPENRNNMNDSTFRSLKRTRDDDSLTLDKQSGASGQYTPSLIHYMSLPKTSSEMAVAEKLLRFDQDSTPCKTRAKRGCATHPRSIAERMRRTRISDRMKKLQELFPSIDKQTSTSDMLDMAVQHIKDLQKDLQNLKNARSRCTCSCKES

>AabHLH71

MQDQSFMNHEYQMMKPYHLVPGFSTIDSFCSGSNTNNPSFVDHEFQIPKSINETSFIKRQPSYKMTNNIVKKLTSDEHLKPKPMPLSSNTNTFTISFGDLKPKDEVITLCDSFGNTPGMKKGTVIRNRIQLQDHMLAERKRREKLSRGFISLSSLLPDLKKMDKATVLEDAANYIQVLECRVKELEGLPHLKTSDMEMDMSAKRPKHSFSDNNCSLYDETTVAANHIPHNPEIKVRISGSSVLVRILCQKDYVCLVNAISEVEKLGLSVSSSSVLPFANVALLINIVAKHLVCVCATFMLQR

>AabHLH72

MDMSQGWLEELEMEDPGYMSCDEMNILNYDIDNFSLDSFYSDIYTEKTKNINQISQTRQSEVNNPNHQEKSSSTINKISTTPEPLIPTNLPSSKTFTISFGDLKPKDEPFQFHDLPGYEAAHTTKVSVALRNPIQAQDHVLAERKRREKLNKNFITLSNVLPKLTKMDKASMLEDASNYIKELEGRVKELEGTLENNKRENVDQESVISVKRYRPSSSNDDLRDEPATSGESTAPCKTTPEIKACRLGNTLTVSIQCQINHSSFVKALTQMQKLGLSIISSSSMPFAKTHLLITIVAQAVRSDNNVAAVASTTLGETDYGFIEVKNRKAKGKKVWVQMLQRKPFCGIDVGKVAQETIPLLPALQTPALLVNQQTQASSAVEDARIPKEELEKRLDNFEHVVFRKQPCVRAAYTRWISRDDVTTNHIIEVPGGMIAGHEDRFHVSISTDDIMSLWKLDGLNRNLCLQPGSAVKDYLTRTLSVSDYDFMLAPYAQEGHWVLFVICPKQRTGYILDSKNRKQTKTERTYWLTSHLQDAVGSYTWTMAKETSFTCNQQPRDSDSGYYIMRWMYDLVNTDELHFPSKDRRTT

>AabHLH73

MEKKDYERHCDGDNLGHMENKECKDEDVQVGKGKSKADKKNWTAIEEVALAKAWIHISTCKKVGNEQGRDKMWQRILEHFATTITDTKRTHHSLNTKWKNMNHAMGVFNGLYIQQANEQNMNHVIIYPFYLFCVLPENHQFPTMVSPETTNWIYYPTSVEQDGSLADSDGHKETGSKKRGRAEPCSGTSSKACREKLRRDKLNDKFVELASILEPGKPPKIDKAAILVDAVRKLAQLRNEAQKLKNSNTEIQENIKEMKNEKTELRDEKQRLKAKKEDLEQKVKSMNTQPNFMIPPPGIQAAYPAAIAAAQGQTMGNKFVPVVSYPGMAMWQFMPPAAVDTSQDHVLRPPVA

>AabHLH74

MDPNLNLMWNQDDDDVKVSRDDDSSETETTRNPDTQRSGGGVKVDRTRTLISERKRRSGMKEKLYALRALVPYITKMDKASIVGDAARYIQDLQTQARNLRSEIATIEATKNQKLSSRNMNKKNVPNSLPVLKKISKMDMFNVEEKGYYVRLVCNKGRGVAVALHKALESITSFQIQSSNLATVGDNFVLTFTLIVTACEFDINLQNMKLWLSGAFLGQGFEFNTFPSP

>AabHLH75

MSEILQIFDHSPPSHYQMHHHGHFQHGLENVGSQLNGVWESESPLSSIFNFGCSEVAENCFMGFSVDGLSKVTTEVEQTQSSRKRKLELDEVEVDSKKQSVECDSELVEPPKAKRNTRNGNSKPKVDPKTDYIHVRARRGQATDSHSIAERARREKIKRKMQFLQDLVPGCSKITNKAAILDEIITYVQRLQMEVEVLTMELAASTTSVDLEMNNSLPQEAMNTF

>AabHLH76

MNFEMNNQNNNGLLRFRSAPSSVLQSYVNDIEKNNKDLNDGLNYNFISNTFQDLQEEVDLKPNLVNEFHYPRQSTTQGSMDSNGYSMVQQPKMSSSLLRQNSSPAGLFTHINQPNGYGGMSGYKLVNAGNNGEVNPSSSRLKRQMSSSSGVKMLPRIAEVEPDTMDPGVLNESSDYPFGSWEQESSQFTDSFTGLKRELDLQNGNTLGNQPPMLAHHLSLPKTSGEMAAIEKLLHCQDSVPCKIRAKRGCATHPRSIAERVRRTRISERMRKLQELVPHMDKQTNTSDMLDFAVDYIKDLQEQYKALKDCRANCRCSAMSGLT

>AabHLH77

MVLEINNTFLRHKQHKCSTLKTLLDDHLIGRSSKMFIIGRSVIGMCEFPNNTVETSFPPWFNHKIREKSVAKDPSCSPELLSLACGPSSSASTYPACIVNGVKFMVHERDILHTTQGSGVSTPGLDGDMYYGQLEEILELTYMGNRKVVLFRCKWFDTRNPNNPTTRSRRSYSERGIRHILTDKDGFRNQQYILATQAAQVFYLEDPARRPPHWKVVEDVHHRKIWHRDVPSFNLPGYCAGNMVTGATSNRAIKRALRGNNNKPLPIGFDHDDQGTFSPIGTYAPQLASLIGEQVRPLPLDCKWEEIPDVYKAHIFPALRTYFEIEPWLNDNRQVRYEGQVYRVGDRVCEGLKLQMQLYYRKNKNRIKKKYFDIHSTPAVAREHPPPPNKWVNRTRQEWELLVDWWSDPARMERSAKNAENRARSKTLAMKSLYDQVKAGTAPSMTEREILHKVVPSDSRQNISGVGRRMTGSTSKSTHQPFEQDYITRQHMTEVLRREQQRADLAEQRARVAEQEARLARETANATNARMGTFESFLGQFFTFYNNQGNPFPVPFPPPNSVPGPFSFPGPNPVPGPNPVPGPIPFPGPPTFQAPFPDPNNAFNNCYRPSFPDSTTCPASGQYTPSLIHHMSLPKTSSEMAVAEKLLRFDQDSTPCKTRAKRGCATHPRSIAERMRRTRISDRMKKLQELFPSIDKQTSTSDMLDMAVQHIKDLQKDLQNLKNARSRCTCSCKES

>AabHLH78

MDFFLQCRNPNFHSSSVVATNNKSSITFHGKQGENKRNRSKDKINAKVKLSTDPQSVAARERRHKISEKFKILRSLIPGADTRNMDTVSMLEEAIQYVKFLKSQIWLHQTMINFDDYDKANTNSHHQDLLSLYQPYVENNHLSSLPQIEHEILQQLGFPHGSCFKVEGVSEMESFYHDHHHVIYP

>AabHLH79

MESVSTLLGEWNSFGVNVSEEAFFMSQLLENFPFSNDSETNLPFEVPSTFWLQHELTMGVDEVHETSVYLSHNTISKPHCLSQDHNSSDDSSLLFSNSSGTGYPLIDPMREGSDNLVPKKSKYDKSVASSRKRSASMSDVHENKEKIKCRKIQKLVSEIYEVETEGEVVVGQTMKVYASDDDSNWSHESSISPRPEAALITNSNGKTKASRGSATDPQSVYARKRRERINERLRILQKLVPNGTKVNIGTMLEEAVQYVKFLQLQIKLLSSDDMWMYAPIAYNGMDIGLDFIVNPCLAQKRGKI

>AabHLH80

MVSPENTNWIYEYGLIEDVSAFNCWPPTSFNNNNGSSSNPPPSAELDGSIVDSDGHNDSRSKKRGRAESCSGTSNKACREKMRRDKLNEKFVELASILEPGRPPKIDKAAILVDAVRTVTQLRKEAQKLKDSSSELQEKIKELKAEKNELRDEKQRLKMEKEKLEQQVNTMNVQPTFMAPPPAIPGAYAAAQGQALGNKLVPVISYPGMAMWQFMPPAAVDTSQDHVLHPPVA

>AabHLH81

MAISDFKGFSELIAMEDPPSFNFQDLVNSYDDHLDPMGIAVNSFDANVGVFNYYKPVMEPSSRPTKQIKTSSWNSSCVTNEHSLMNVNMSQQANLVTPKEETTVSSKINGFQFGPCMKSGFDNGYRGGVVDGAKNSTKVSHAQDHILAERKRREKLSQRFIALSALVPGLKKMDKASVLGDAIKHMKTLQEKVKTLEDQIKKRPNTESVVFVKRYEVLADNGESSSSNDGPINEQLPEIEARFFGNDVLIRIHCEKKKGVLEKILAEIEKLHLSVLNSTCMTFANYALDITVLAQLDKEFAMTMKDLVKNLRSAIKQFV

>AabHLH82

MQKGDDDQNEHIPTSQGISEHHAASSQFHEAPYTSVFYTNDVTNFADIVKVLSQVELPSPSPVLATCCTSTREDNLITFHEGQSSGTKKDDETKVNAEQRETELKDKHDNLQEPHETDFFASRTQNKERRRRSQIHEKIRVLKELVPNCNKRDQASILDDTIAHIKSLQMQLQMMQCMGAGAMSQGPYFTMRPMHGMGYMSDGHYGSYFTSSYPIFPPFASGFGPSVPTMENFVGSPRLLQIPYQEFLSQTIEPACSGTLSSDTIIPTAGDQVGSGISSHYTYHMPVTNQVYEVQDD

>AabHLH83

MNSFEYSIGHFNNRNFNMFQPDFIPETDFENLLSTIRCETTDPIEKFCPDYNCHHFTNACTGMQLLPQLYGQNENQEDVKVSGDDDSYETVTTDNPDTQRRSGGGVKGDRTRTLISERKRRSGMKEKLYALRALVPYITKMDKASIVGDAARYIQDLQTQARNLRSEIAKLKHQKTNKCRGVAVALHKALESITSFQVQSSNLATVGDEFVLIFTLNDLEKILCDSPWNVMGFYLSMAPWDPNKSFAEIDFSQGSFWVQAHNLPLGKLTKSFATDLAKRIGILLDIDCESYQKDLNFDQQADGQSIQGKQTPSSLIRLPTMPENSTPTTHSNNPKTIVSTTTQTIPTQPQIIITNLVKTHIPTNLFEPRSLNRKPNTEQPVYFITSPTESPVWWLRKISHKTNVNMLSWNCQGVGRPLTVSHLRELCQTHRAEVVFLMETKNKERRMESIRRSLHFSGYYYIHPAGLSGIYLIGDLSDKQGSSTNISHQIEEFQDFISASMLFDIPFKGLSNMWDNNRKDGASIRERIDCALANDDLVEAFPHHMLTHHPLIAEIKSIQSLPPTSENCARQNFLKLKLEEIWLKEEMFWHRRSRNEWIDDPKALNQLILNHFKAVYSSSRARDFSDVLKPIDVVVYESMNLSLEAPVSDSEIHKAVMQLGALKAPRKDGFPGLFFQRYWHIVSNSVIKVVRQVFENGVMPSSLNKTVIVLVPKVPSPEKVGFKPFIHKIISPQQSTFIPSRLIQDSKVKASISQSQLPISLQIPQKQIMNPKARYLCMPSIHGRNKSELFSFILERVLNKMQGFLNKLLSYVKRFFWGGYAHGSHIHWVSRDHISKPKDEGGLGFRDLKAFNLALLAKQGYSMVHLFHNSQLLILWFFIAPLKKVHVHLFHNSQLLILWFFIAPLKKVHLNCDGAFKLNQGVVGIATRNCEGSLLLCLGERWHASSVIATEVITLRSECSLAMMKRWHNVIIESDSQLAISLASSESDPPWSLDAIVGDIKD

>AabHLH84

MESMSTLLGEWNSFSGVNVSEEAYFMSQLLENFPRSDESENISPFEAPPSTFWPSHELTMSLDEVEETSVYLSDNTNSNPHCLSQDHNSSDGSSLIIPTSSGAGYPPIDCLREGSDNMVPKHTKNNKLFASSRKRSASMSDVHENKEMIKFYASDDDSNWSRESSISPREKEVAIPNSNGKTKASRGSATDPQSVYARKRRERINERLRILQKLVPNGTKVDISTMLEEAVQYVKFLQLQIKLLSSDDMWMYAPIAYNGMDIGLDITIPSPR

>AabHLH85

MDCHVRNLLTRAIFVKVFVDGKQDDAKVVKVVGVVDEQNNDERNVLEGKGDLGVEKWIGAFSSQFVRMEDSSFSFHWPVNSIDDQLSSIVAVGENMQRVNAGVPLFEYHEPVMEPSPRASKQIKTHSMNQNLIHGSDFDIVNRGILVNPKEEAFASSSSYNSIPSPSSGNQNYYGFTEGFDGDGSGAKVVPTSNRSRISPYQDHILAERKRREILSQRFIALSALLPNLRKLDKASVLGDAIEYMKTLQEKVRTLEEQTPETNRKSVSFEMVGDDVEKSSSDEKLSRLSNQLPEIEARFSGKDVLIRVYCVKKAGIVEKTLAEIEKLHVSVINSTAIIFANSALHITVIAQMDKDLAMTMKDVVKNLRFSLTQFL

>AabHLH86

MALETIIYPKGEIGNLGYDYFMQEEHAFDEFLECANSNITHYQESSNNVHAYWGDYYSSPENCIGAHDMKVELSPTVVVATSTATTTVGARKKRRRTKSGKNKEELENQRMTHITVERNRRKQMNEYLAVIRGLMPSSYAQRGDQASIVGGAINFVKELEQQLQTLEARKRSNNNNVFSPSPQPFSDFFSFPQYSIRPDANDGSSTSTTKSRLPAMAEIEVTMVESHANIKILSKKRQRQLLKMVAGLQCLWITILHLNVTTIEQMVLYTLSVKLEDGCQLSTVDEIADAVNCLLFTIEEESLSFSNIYIIPMGWRVNSSSAPLACLTQDIKAYPDEPQSILEFSSICPSRGELVVTRLTITRTGFFHRFFSTETRLCEISHGIRISCTPNC

>AabHLH87

MESFGSFFDEEWENLSKMFSCDQYSDHGLYSSEQDHGLNFEIPSFVSTLITEANNANSSFIDHNDFHYTSENVNSYHHYSQETSNNANECVAYDGSASLSYPSSNTIPLPTNGVYEHEPMNLYNENNNISSLQAPVFSDDSTEPMVPNADQRTHMIVKRKIEMPESPLEDKVNEDKPDEKPKKRARVTKDNKNKKKAQPKKKQKVIDSASNENVIDGEDTNNNKGGNAPIASSSSCSSEDDLNGGGDNVNWKTRAGRGAATDPQSLYARKRRERINERLKVLQNLVPNGTKVDISTMLEEAVTYVKFLQLQIKLLSSDEMWMYAPIAYNGMDMGLYQRLSLNMS

>AabHLH88

MDDNKFHLVPFDNNNNNNTIIQSRPGPEPKDRKLQKADREKQRRDRLNEQFTELGKTLDPERPKFDKATILGDTIQMLNDLTAQVSRLKSEYTTLTEESRELTQEKHDLREEKASLKSDIESLNLQYQQRVRAMYPWGHMDQSVVMHPTSYPYPVPMQMPMPPGSIPMHPSIQPYPFFGSQNPGVVSNPGSAFFPPVQYVSPVVQTSTRSQVSSRQGSRNKSSEQGENGSGKDGDSNDVATELELKTPGSTGDQDGSPCHSKPKKLHRKETSFSDENSASGCASSGTVQASSSTSVIGGANGDHQRSASPP

>AabHLH89

MDPPIINESSFSTANPSSYSLTEIWPFNEGGLGLKIGGGFGDSGESSVTEQSGNGGRNKKRDGNYEDETSLLVSTSSANDLSNLNGKRMKISGLKDESGGLKVDGEGSSGSGSKAAAAEQSSKQSEPPKDYIHVRARRGQATDSHSLAERARREKISERMKILQDLVPGCNKVIGKALVLDEIINYIQSLQHQVEFLSMKLEAVNTRMNSPH

>AabHLH90

MGKVSNTVHDQDHVSDERDRREKLAQQFISLSTLLPNPTKTDMASVLEDACNYIKELESHVKELEESSAGCNSKDVRESNDDEASSSHKTKRGEDIEVQMSGKSVLVQFQCKRDSSSYVNVLGEMLNIGLSIISTNAVSFTSTTLFINVVAEMADDFCMTPADLSKNLQQVL

>AabHLH91

MLPYFQHNSCSEVSNFPFPSTLYQQDLPNPDNQKPAGHKRSCYNLQPQKDHLVIMDNGVDKEKKKKKMVHRETEKKRRQEMSNLSSSLRSLLPLHFIKGTRSMCEHVNQAVNYIKHLEEKTKELSIKRDKLKKMCNVVTDAEVVLKKNDKNERVIVECSQNPITVTVSCSEGGIEILVKSFINENKGLQISRVLKTLVHEGIDVISCNSTKINDSLLIFTIHSKVIDEKTSIQVSMLQQKLAELVNSAS

>AabHLH92

MDLDKLPNPFSLSFSSTPPPQFRTSFKPDSMEAMREMIFQMAAMQPVQIDPEAVRPPKRRNVKISKDPQSVAARHRRERISERIRILQRLVPGGTKMDTASMLEEAVHYMKYLKKQVQSLEQGAAGGMAMVPMNKEVNVGGGYLLHNQYEMSW

>AabHLH93

MDPPIINETSFSTANPSSYTLAGIWPFSSVRMGGRNVSTGYDESMVTEFGAGGRRKREVNFEDECSKIVCTSSGNDLNKVNGKRMKISGSKDELGGSKIDQEGNSGSGSKAAVGQTHKQPEPPKDYIHVRARRGQATDSHSLAERARREKISERMKILQDLVPGCNKVIGKALVLDEIINYIQSLQHQVEFLSMKLEAVNTRMNSPVEGFPTKDLGPPPFDAAGLLFGSQVARQFAHGSQPEWLHMQLDPRIYVMGAKIIC

>AabHLH94

MSSGISANSGGDGNLVVKKYDHNAGERDRRKRVNNLYQCLGSLLPVTGDQKKKQVSIPGIVSRAVKYLPELQKEVEALKLKKENILPFSSPIINSRQEGLAIKNQSGEGAITKRNSWLVSSVNVLGDKEVVIQLTTLTDRMSTNKENCFLSKVLENLENGEYGFVLLNATTMKCSGEGMVLSTLHLQVHIFIYFYECHVTLHTNKVLKKAN

>AabHLH95

MESFGNFFDEEWSSLSTMFHGDDAGSDLFASQGLMSSQYDIGVNIEIPNLFMHSSDESSNSDSFVVDDEHLVNVSNNVNPNFYQFFAQENICSSRASNDTASLSYPCHESFPLYHSNIVPSPSNDVCDQSNEFCMMDEINNLSLPIQVFSDGSLYVRQGVATENVGMENSLVRDKETPLKRKHEVADAIDDEVNNEKTNKNPKKRIRVSRENKNKKNVQPKKNQKIDETEVGNNNNNGERNNGQSSSSCSSNDELNESQDVDATNPNGKTRASRGTATDPQSLYARKRRERINERLRILQNLVPNGTKVDISTMLEEAVEYVKFLKLQIQVVATSKCRDYYDDTGCYRCNDYGNAIAFSILFFYYYLKGYHLNLWRRLGLIELGFNLFELKYDYDNNLILDAKKAAPSGVSGLDTNGGKPNVFSQGCRCVTQQYLSRFKRLCRLYPPIIYRQVAIKKLHSSRQNSADTLCLQTTTGTGYSGFPVGNIRHFDHVTSGALHELPRAPAILDLQSQIIHHLKVVSYSPNSTGGVDNPVAGVLWFDHVINGVFPGECFPLLQQYDDCLTNTNIMLLITVNTILIGGYNVVDLLHAQKLKPMVVRSNRRNMQQALHMDDSDGRRSRNSLPSVTSRKERLEARHPPPPSCKQPTTTKKQLAIHHYKCSPSTTTIINSPPSITITARYPPLPSCKQPTTTIKNSPSSPLP

>AabHLH96

MDPPVMMNGVGGGGGGFRSGNMCNLAEIWPFQMNLTGNGGGGAYVENDDPMVVDNSNNGNSNSKKRRDDDELSKGVSTSSNSMLDSDGKRLKTLALENENSESKPEGERSSGKKAEKPAEPSKQDYIHVRARRGQATDSHSLAERARREKISERMKILQDLVPGCNKVIGKALVLDEIINYIQSLQQQVEFLSMKLEAVTSRSQPSPQGYPSKDYGQQTFEMAGVPFGSQPPREFSRGSSPEWLHMQIGGSFERTT

>AabHLH97

MEPISTLFGEERNFFSGINSNEEADFMSNLFSNFSTKVSNVSTYQDASAFWPHHEQAMNTDEANEVSVSISDNTHATMPLLFQEDSYPESNSIFFPTSSGESSYLSVSCSRSIRKGNSDAPSEYNNLSPNTRKHSCDEADVHHTSWKKPKISRSQSTNTLDSDNDSKNVAMLNTNGKKRASSGSAVDSQSAYAKKRREKINERLRILQNLVPNGTKVDISTMLEEAVQYVKFLKLQIKLLSSDDMWMYAPIAYNGMDLGLDDVKLPSPR

>AabHLH98

MKMAMDWSSSMWLPELEMDDGGFMNQYATMSKPYDTTDSLSYGSFNAESYTGNGCFIDRCYQNPSRFEEKTEMILPGYQKASNISTKTQTPKTPGATISSSNTFTISFGDLKPEDKIMPPFDYSICYEDAGTGKVPTVARTPVQVQDHVLAERKRREKLNRQFISMSALLPNLKKMDKISVLEEATNHIIELHDRVKKLQGLSVVEQKDAKEYTIALKRSRPSDDDNEDSSYQGINFEDDSADVRSKSSAEIEVRISGGSVLVRIYSQKVPSLLGKMLRKMQELGLSVISSSSMPVANTTTLIIILAQIEDDFLMTATDLVNNLQLAL

>AabHLH99

MYPSSNSSTSRDPNNINNNTNGDTNVNQQGIGLARYRSAPVSFLTTTVDSVINGQSQQQSTVGNHMSGGGGTPIRFFSPPDTTSSQLSSVSNNTNTGDRVQTGTSFRLNEFATAFNGMKSTSSQTQNPSPLFRHGSSPAGFLNTLVSSTPTDGRGSRLGSQLSFTGTNSYSRLSEEPDIGNSLMFSSSSSHNKRAKIDVNGLNIMESELNFGLSESALEAAAMEKIMDLPHDSVPCKIRAKRGCATHPRSIAERNSSIPYKSSIPLILDHAEKERRTRISGKLKKLQDLVPNMDKQTSYSDMLDLAVQHIKGLQTHVQNNCLEYLGASDLQSMITLESYITTSSAVLSKLPPEFNNFTIPTNIIHRLSGRFSRWTLGC

>AabHLH100

MNMSGLFPSAHHQFENNMHPSQQHSLLHTVPQAFQGQPTTSTAVTVAHPPSIRPRVRARRGQATDPHSIAERLRRERIAERMRALQELVPSCNKTDKAAMLDEILDYVKFLRLQVKVLSMSRLGGAGAVAQLVSDVPLQSVEGDANENGYNQPAWENWSNDDTEREVAKLMEEDVGAAMQFLQSKALCIMPISLASLIYPTQQPDTSSLVKPEPSAPS

>AabHLH101

MDMSQGWLAELEMEDPGYMSYDQMNILYEDIDNFILDSFYSQIYTEKTTTINQTSQTQKSEVDNPSHQENSSSTINKISTTLEPLIPTNLPSSNTFTISFGDLKPKDEPFQFHDLLGYKAAHNTKVSISLRNPIQAQDHVLAERKRREKLNRNFITLSNVLPNLKKASMLEDATNYIKELEGRVKELEGTLENNKRKNVDQEYVISMKRSRPSSSDDEYYLCDEPMISGESSAPCKTTPEIEACILGNTLTVSIQCHKNHSSFMKALTQMQKLGLSIVSSSSMPFANTHLLITIVAQIADDFSMTTTELVKNLQQSYELKLDNYLSFLQAFKNRQLFSFHAIYLVQREES

>AabHLH102

MDPFENTNWDLIDYNCLINDVASTDLYWPEQSPAVQVDASLASLTPPEDGVEKECPRKRGRSNSCSRAENKACRERQRREKLNERFVELSSTLEPDRPATTDKLAILGDAIRVLNQLKSESQECKEMNEKLLEEIKTLKAEKIELREEKLALKAEKAKMEQQVKAMTNSNLPPPGFMAPHPAAYQAGANKMPVFPGYGYIPMWQYLPQATCDTSHDHELRPPAA

>AabHLH103

MFSLDQNEDLVDHDESCFISFQQQQDRVPNLDEHISKVDGMAIDRSSLNHPESSTKKLGGSRSLNKLNLVRGSGDAGGDEQAQRKMLHREIERQRRQDMAKLHASLREILPIEFVKGNRSISDHMHQAVYYIKQTEENVKRLGMRRDQLKNSLDTEGSLMNHLLNTVSVNYSNGGVEILINSCTIEEGFHLSRVVKALVDESLNVTSCTSTKVNDRFLHSIQSEGNRSISDHMHQAVYYIKQTEENVKRLGMRRDQLKNSLDTEGSLMNHLLNTVSVNYSNGGVEILINSCTIEEGFHLSRVVKALVEESLNVTSCTSTKVNDRFLHSIQSEASDLALLDLSMLQQKLAIDQQHLIKIWCDYKLTVTTEI

>AabHLH104

MISFQQSGELVYHEIPSTISFRNQQDLTVNLHERVTIDGNTNLAGSNKRKRGRDHSSYKANSGIIGHGVRDNKDEHMLRKLVHKEIERERRKNITKLYASLGALLPYEFIKGKRSISDRTLQVVNYIKHMQGKIEAISVKRDQLKKLVGKSFKENTMNKVSISSCNGRIVEIEINSCSIEDGFHLSKVLKALVEEGLHIINYTFTKRNKRLLHSIQSEACDTTLTDLSRLQQRLVSITNTWQIFT

>AabHLH105

MALAKERNPNDHSSHMPGLVHQSYKFYGDSESPEKKGNFTGSSSNSSSLSSPGSATNSSGLLFRASASHQQQSQPEEGHSVISFKPGYYDNNFVQGGSASCFLSFEENDEQPYSSNLSPDQLMNLGSGSFDSIRLLETMSCIQSGSIKENNHHEELESFAWPNPSSSDNYLATQEQPSFHKRTHSGESEQAFKKQCTTNTATKKTKQKATQPKDPQSIAAKNRRERISERLKVLQDLVPNGSKVDLVTMLEKAISYVKFLQLQVKVLATDEFWPVQGGKAPELSQVKDAIDAILSSSQRDRSSSSK

>AabHLH106

MANSNPSDGSADDFFEQILGFPFAGGVGGGGGSNDPNMAGNDQGHVMAGAGVTNNPRMLQLNSGIGVGGGGVGGGGGYHFPLGLSLDASGSGKRFRDDVVDSRVSASGFQGQPMPNTVQTAPNPPAVRPTRVRARRGQATDPHSIAERLRRERIAERIRALQELVPSVNKTDRAAMLDEIVDYVKFLRLQVKVLSMSRLGGASAVAPLVTDIPISSVEDEGGEGGRNQPAWEKWSNDGTERQVAKLMEENVGAAMQFLQSKALCIMPISLASAIYHTQPPDSTSLIKPESEPPL

>AabHLH107

MELLENNINNSLGNFQPNVSLPFMPLLDGTNNNNLSFNISGDHQTRSFYNPMTRLPTTTDDMDKTISGESLGNSNLPNFSSGVTGQNKANRNYSYGGRKRKKNHEAEVEKPREVVHVRARRGEATDSHSLAERLRREKINGKLRSLQELVPGCYKTLGMSVMLDVTVNYIRSLQNQIEFLSMKLSAASMYYDFNSPEMDALDTIKGANGYEAQVMDRMGAEGYGDLLQFQPTWPL

>AabHLH108

MEDLSWERNISFPWSNINHHQHQDIEETFIFGSENIFLNPIQDLRKPDPPTCSNPLPSSNGSASEYWSQTTIAKGKTVIPITTPTHADRVTSLESLNCLFSDKNSNVDTSLDGISVIFADYNNLCNNITNIISDGSSTSVTKDTDDCIVSQSSNERRLKRPRSDPGRPVSSNINFRQTSESDETDSEAIAQMKEMIYRAAAFRPVSFAEDEVAEKRRRKNVKISSDPQTVAARQRRERISDRIRALQKLVPGGNKMDTASMLDEAANYLKFLRSQVKELEHMGQKYDFMSCGTTTSAQSTSQIMQNINPNVTLGVPFPMQTHFLLPHQQLYPNPPHA

>AabHLH109

MDMMTMMMDLDKLPDPFSIPFQDLSDSSIMDLNFSSENSTTNHNGPSSLMYPSQDHVAIPPSHFPRASWLHQKPDSVEAMREMIFRMAALQPVQIDPEAVKPPKRRNVKISKDPQSVAARHRRERISERIRILQRLVPGGTKMDTASMLDEAVHYMKFLKKQVETLEQAAVSGTAACSNVTTTGMNTVVSFSPTNMNHHGGNNNYMIRACGESAGHMVGSM

>AabHLH110

MDIDFLKSFSDDQTEMMMMMQLEKLPDFCGIYGEDHNPSTEFTDQGSSSSTNPNNNPNMSHFLEDSHFANPSTTICFGSPTQTIPTAMQPIHIDPESVKPPKRRNVKISKDPQSVAARHRRERISERIRILQRLVPGGTKMDTASMLDEAIHYVKFLKNQVQTLEKAGENRQPSGSAGIGFPVPMTSGNHIHMGTSSKGYHQQSGQHHFLGA

>AabHLH111

MSHELRNDPSLLERQQARMKWQQQKQQSFFNASDHTMQNMFSTSMPLAQTNETFTGLLSGHNGLDIVKPDPGLMEDWAGFGDHLSYGYMNQNSIMPLGADQSFVHGNSVSVSTSVTVSVSPKKRKADKGQSLEVVTEKEKKLKGCAEEGDSKITHQNSNNSDKATGDNKSSNSKGASTNTSSKDKSKVSEVQKPDYIHVRARRGQATDSHSLAERVRREKISERMKYLQDLVPGCNKITGKAGMLDEIINYVQSLQKQVEFLSMKLATVNPELDFNIDNVFAKEMFQPSTSEFQGLGCTSETPNPAYFQLNSLDQVYCGLDMGINSAEMALRRSLTAPMSIPETFMDSSCFNQILPSAMWDGDLQNLCKMEFEQGTLIPIQSHQYTGSNEGSNLKMEM

>AabHLH112

MMEKREEMCYQFVEDEIESLISPPEGGSSFTALLELPANQVVKLLDSPVGFPVPEGGNNDVNLVKEEPVELRNSEDSDPVVSKSVKRKEREKKVKLPVKKSKNVVNESDGDKEKLPYVHVRARRGQATDSHSLAERARREKINARMKLLQELVPGCNKISGTAMVLDEIINHVQSLQRQVEFLSMRLAAVHPSIDINIDNMFSAESGSLMDCNFHGMVSPPLSLDGQINGHSQQLWQPDGLPPQPLWGAEEVNPNFITPENSLLSYDSSGNSGESQILNWAVWMFVLKLNI

>AabHLH113

MVLCKIRVLTCIVTFNVGHALMALVPSRDSNNHKPAGFNTERTPTARDNKNTPKTAPTQTHPQLPHSVFGVAGMQFSPFGFCPPGYVPNQISGPMLPQAQHQLNSHGFVNSLIGIGQQAGAQQIPHAHLATNSVSSSQQAQPLGQPIYGSQTMQPGDKWFHEGNSQDYDSIDSEYVAAIAAATFAVDSLEEKSSSQHHRREKTREEDSLRTRTSQIDRALSLARPSRPRDPYVNRNLSIRGSGNTNVDTWERNQLLKIQKRYEKNNLTILEWENEKKTRAKHRMEEKKARCRHHEPIEGMVEGQHVIQLNITILINGSGSRLADHGVAEITWENGQPAMHGLGRANETLESIVHQATTCYNQTQYPEIDLQQSQSLPRARNLSSNVASSSRPTYLRKRPRESVIIHDQCVGNLGNASLQEDNVSNSGTVNSKDNDTTMMTWPSFDSPNQSMKSQKTDDDSACQYGSENQEEECRTEGETIRSQSSRRSRAAAIHNQSERRRRERINQKMKALQKLVPNANKMGMPGVIPQPVHHPFMVPQTMLSPAHVGATSQTIHSRPSTSTTVPFNDPHSTFLARQMNMDMYNNMAAFYRQQVNQGKSMSVDSSQLDHVRGE

>AabHLH114

MGSSEGDTIANNNDSEMGYQYRGEMSSGTIFNNKSSSGSGNPFGSSWDPLENFGAYPLMNQYQSGGELVPKIGSFGSGNFSEMVNPFVPNSSPNSGEKRRAQFDPNMNGERNDGSGDNTCENDEKKQRIDSRSKQMGKQVKDNSDSGGAAAKDSYIHVRAKRGQATNSHSLAERVRRERISERMKLLQELVPGCNKITGKAVMLDEIINYVQSLQQQVEFLSMKLATVNPEVNIDIDRLVFKDIHHSRGSSSNPPFGFGPTLSSSHSYPHRSLPGISPTTTPLHPIHPQPVWDSDLHNLLQMGFDANPGINNLGPNAGRAKMDL

>AabHLH115

MGSSEGDTNTNNNDSEMGYQYRGEMSSGTIFNNKSSSGSGNPFGSSWDPLENFGAYPLMNQYQSGGELVPKIGSFGSGNFSEMVNPFVPNSSPNSGEKRRAQFDPNMNGERNDGSGDNTCENDEKKQRIDSRSKQMGKQVKDNSDSGGAAAKDSYIHVRAKRGQATNSHSLAERVRRERISERMKLLQELVPGCNKITGKAVMLDEIINYVQSLQQQVEFLSMKLATVNPEVNIDIDRLVFKDIHHSRGGSSNPPFGFGPTLSSSHSYPHGSLPVISAITTPLHPIHPQMDVAKYDRFHIYVRFNGYLRQVKELPKPVWDNDLHNLLQMGFDANPGINNLGPNGRAKMDL

>AabHLH116

MGSSENDDSEMGYQYRGEMSSGSMFNNKSSSGSGNLFGPGWDPLENFGTYPLMNQYQSGELVPKMMVNPSASPNSNSNNSSREKRRLNPNMNGDNVSENDEKKLRVDSKGKQVSEGSDRKEDYVHMRAKRGQATNSHSLAERVRREKISERMKLLQDLVPGCNKITGKAVMLDEIINYVQSLQQQVEFLSMKLATVNPEVNIDIDRLVFKDVLHSRGSISNPAFGFSHTLNPSHSYSHGSLPGVPATTAQLHAIHPQPVWDNDLHNLLQMGYDVNPGTNNLGPNGREKMDL

>AabHLH117

MNVVSPEMLMSVYERQQARMKLQQQQSFVNENENDHFSALLQDIKPVPCMQNGWPDLSNDQFPSLMVDEKINKRKTHEDHKLEVECKENGVKEKKVKVCSQEESTKTSSKSNVGEDKKQDFIHVRARRGQATDSHSLAERVRREKISERMKYLQELVPGCSKITGKAGMLDEIINYVQSLQKQVEFLSMKLASLHPRFDSDIDNLITKEMFELSAVGYSSEIASSAYFQLNSLLEMGNSPIDMMLRRSMGAPVSIPETSTVPSCYNQIQPSVTWDGDQQILDKMGFQPTIPFQAHKFTGAYEGSNLKMEM

>AabHLH118

MRRKSSVSQRPPAAKEKERRDRMKDLYSTLATLLQLQPHEKMSPPDLLERATEELKQWKERVERLKSRRMELEKDSKGDSSNNIQKLQQFVQVREVVDLQLEANLKILVNNKNVAPFDILRVLEESGTHVTSSNFCIVSHHLFCTIHAEASNARIGFDAEQIESRLLELVY

>AabHLH119

MVDGFTLGISLELLDNNMNTELENFNSAEVNLHGFIPMSGDNFSDYHHRNQRSHPLSFMSLQDGTNAIQNFNFFGDHQPGSVYDHMNHFPITFGAQRDGTETRVESFMNSDPPTFGPALIGETKGRGGKKRKKNDVGDEKPREVVHVRAKRGEATDSHSLAERMRREKINEKLRRLQDLVPGCYKTMGMSVMLDVIINYVRSLQNQIEFLSMKLSAASMFYDFNSPEMDVMETMKGTNGHEAQVMERMVEGYGDPPCFQSTWSL

>AabHLH120

MDDLQFSWYNMIHAHDHQQQTFDFNSDNIFLNQLPHLSPTDWTDQAIENDLICTNGFQEIKNVISSNNNAAASLERLLSGPNSSNDASDDGVSIIFPDGKSSIWCNNVNVTRNNNGISGVSSADSVTDDGVVSETLQQLETDTEAIAQMKEMIYREAAFRPVSFAAETVVEKPKRKNVRISSDPQTAAARQRRERISERIRVLQKLVPGGNKMDTASMLDEAANYLKFLRSQVKALEQLGHNSKTINAHSYASLVAPFTQTPFSMQTQYSFPCENLYPNPPSSVHH

>AabHLH121

MGLVEGSDGGAKGTTLVAVGGGMKRNKRTLVTMNGDMKKESGVEDKKEIKDLQLCGHSKHLWTERQRRKKMQIMFQDLQALLPCHSSPKEHLATIVDEAIAYVKTLEETIQELENQKLEKLCVSSSTAELDTASRSRIAPFCFTRLSPPVFQTWTSPNVSLDVCGADAYITICSSKKSQLITRICFVMEKYKINIISIKIYSDKYRSMFIFHVQANAHDEVLTASCYEELLKKAALEILLLVNSKSS

>AabHLH122

MDDLQFSWYNMIHAHDHQQQTFDFNSDNIFLNQLPHLSPTDWTDQAIENDLNCTNGFQEIKNVISSNNNAAASLECLLSGSNSSNDASDDGVSIILPDCKSSLWRNNVNAARNNNGCGVSSADSVTDDGVVSQTLQQIRQLECSSGKRTRETIEISDSQSNPKRSRSNPSRPTSSNINFQQPGNPETDTEAIAQMKEMIYREAAFRPVNFAAEAVVEKPKRKNVRISSDPQTAAARQRRERISERIRVLQKLVPGGNKMDTASMLDEAANYLKFLRSQVKALEQLGHNSKTINALGTSPIISHASLVTPFTQTPFSMQTQFSFPCENLYPNPPSSIHH

>AabHLH123

MSHIAVERNRRRQMNEHLKVLRSMTPCFYIKRVGFYHGIVCPSLSQLALAYKSVFLKPETASPRVRQDPSNSQVLASYGSETNTIAIIRRSTGDQASIIGGVIEFIKEMQLVLQSLESKKRRRSISPSPGPSPKPLLQPETPQSERSIISHENIKELGASCNSPVADVEAKISGSNVILRTVSRRIPGQVVKIVSLLEKLSLEILHLNISSMEDTVLYSFVIKIGLECQLSVEELAVEVQKSFSLNHT

>AabHLH124

MDRDFFPFENSSQMPSWKVMSQVPEINDRYNSFESNMTSMTSSPVSNSPSNSPKVNWPIMDHFVKENAPNMRNLIPVAPGLPSLVSDPGFAERAAKFSCFGSRSFNGRTSQLGLNSVDPNSQFRSSVPTLIGNSKLPRVSSSPLLKINRSGHDHKSTNSNEEQESSVSEHIEIGLKNDSSSRKRKVASLKGKAKDIASPGVKEEENDEMNTKKPKITEEGTNGETEKPKVPEPPKDYIHVRARRGQATDSHSLAERVRREKISERMKLLQDLVPGCNKVTGKALMLDEIINYVQSLQHQVEFLSMKLATVNPRQDLDMNGHISKDMNQLYPIEPSTSDFYQQNPQQLLFIGSTPMTQSPMDPLPTVHGFSETFTQFAGFEGDDLHSIVKMGFGDSRNQSPEMKIEL

>AabHLH125

MEMMPMMMELDELPEQYPLPFHDLSDSPLVEFTGGASSITNHHTPSQHGHLMYRTPPPVIIPSVHVRALREFPRGSWFQQKPDSVEAMREMIFRMAALQPVQIDPESVKAPKRRNVKISKDPQSVAARHRRERISERIRILQRLVPGGTKMDTASMLDEAAHYMKFLKKQVQTLEQAAAANGGAGAMPTKTSAPVPPPGFNVVTMNNHGTNNHSNLVRACGQPASHMIGSMELLR

>AabHLH126

MDRHFFLNNGMHSYSQFDSVLSSPVSNSTISNDIVKENFPRIHNSMSLSPNLPLMADDPGFAERAAKFSCFGSRSFNSRSDQFGLNSNKGEFPFGSINNVSPVAKFPRVSSVPMLKIDSSPVGFEEKMNLDEVNMKLSRFDGSAANSNEESSVSEQIPVKKDFSSRKRKGTSSAKKEVEANDDSNSKRLKKPEQENGGKTEENSKLPEAPKDYIHVRARRGQATDSHSLAERVRREKISERMKLLQNLVPNCNKVTGKALMLDEIINYVQSLQRQVEFLSMKLATVNPSLDFDTNDLLSQNVNQQNTNLLQPSTFYQQNTQGLYNGSAPMTQPVHEFVEPFPQFSGFSQDDLQSIVRMGFGENLDLDNSFFQTTHDQPSNMKIEL

>AabHLH127

MEKEKYFNNGNGIPQAWNSIFGMGMGMGMGMQTSEGFFNQNCENSVDQRDIFESALSSIVSSPASSHPGIGPGIPIPGGTGGNESIVVRELIGRLGSICNSGDNNGNNSTNNSCYNTPLNSPPKLNLSILDHPIPGNSVPENTILRNPVPGNPSLPLAQFSSDPGFVERAARLSCFSGQSFMGINKLSTQACEESVSVSAETETGMKVQSTNSNVRKRKMVPKGKGKETQSSSTSFSDKDDKVVAEAEKVESDAKRSKSNDEENGNEKDGNGNGNEKQTKENAKAEPPKDYIHVRARRGQATDSHSLAERVRREKISERMKFLQDLVPGCNKVTGKAVMLDEIINYVQSLQRQVEFLSMKLATVNPRMDVSIESLLSKDMFRPRVSMPTNMNPFDASAQPFPYAFQPQNNGIVPDVSENQFSVNPLMAAMHRNSLMKPSHIDGLGEASTFWENDLQSVVKMALGQDQPQGFHGHMKIEL

>AabHLH128

MAGDDEIEGGNRDEEQIHYNSANLSADWPFNGTNELTNTSMSNPMSNIWDHHPTSSQNLGAFCDVNLQNNPTTSSSSLGFRKGNLLVPQRSLDMGWAPPDSAIKRGGMFLPPAASAMLPHSLSQFPADSGFIERAARLSSFSAGNFEDMINPFGNVNDSSLSPYSSRSVQGQAQEGFVGNGFKSASGDGSKDMSLPVDCGANDGSQPKQGGSGFSGNNGTGDAEYSEGGGQDGNSNEGLGSKKRRRSGQDTEYNQAKRSPQTPSDTTKNNTEVQQKADNTPSSLVNKSGGKHGKQAQSSDAQKEEYIHVRARRGQATNSHSLAERVRREKISERMKFLQDLVPGCSKVTGKAVMLDEIINYVQSLQRQVEFLSMKLATVNPRMDFNIEGLLAKDILESRLGPSGPLGFGGDMAMPYTPNQSQMAVMQAGIPGVGTSSDAVRRTINSHLMALGGGYKDHTSQVPSSWDDELHNIVQMGLNPGTPVSSQNLGSTPPGHLKAEP

>AabHLH129

MSKDGFFNTLPQSWNSMFGMELDSQVNEMNLFNHNWENSMDQSDPFESALSSIVSSPVNSHPGTGIRTPVPGCHAGGESVVLKELIGRLGSICNSGEISPESCIHGNNTNNNSTTTSCYTTPLNSPPKLNLSINHRSHHQLPMIPSDPAFVERAARFSCFGAKEGEFQHMVESGKMSRVSSNQSFIKTGGSVSRLSMNLNSDNKEVDHSVEVSSLSEQINGSETGIKGRKRKVISKGKSKETQVDNKAVAESEKEESDAKRSKSDEEGNGIEPEKEKEKAEGNQKQTKENAKLPEPPKDYIHVRARRGQATDSHSLAERVRREKISERMKFLQDLVPGCNKVTGKAVMLDEIINYVQSLQRQVEFLSMKLATVNPRTDVNMEALLSKDIFHSRPSMPNPMNHMEASAQPFYGMVNDGPENSLMAMMHHGSNMKSSQIDGFSEASAFWENDLQSVVQMGFVQNQGPSFHGTMGSGQMKVEL

>AabHLH130

MEGNPSSSCSRVDRRTIEKNRRIHMKALYSKLHSLVAHDSSREMTSLPDQLHEAANYIKKLQIKLEKMNEEKNNLMGIKKLEINNNHKIKCLNMMVGQARAPQIEVRETGSSLEAVLITGVDFQFLFSETIRVIHEEGFDVVNAGFSILNDTVFHTIHAQNSTRFHN

>AabHLH131

MLPYFQHNSCSEVSNFLFPSTLYQQDLPNPDNQKPAGHKRSCYNFQPQEDHLVIMDNGVDKEKKKKKMVHRETEKKRRQEMSNLSSSLRSLLPLHLIKAVNYIKHLEEKTKELSIKRDKLKKMCNVVTDAEVVLKKNDKSERVMVKCSQNPITVTVSCSEGGIEILVKSFINENKGLQISRVLKTLVHEGIDVISCNSTKINDTLLIYTIHSKAHNCRDIKTPIIQFHPCWDALSSSMPNKKTNFQGNLMVPSGGGGQGSYSGGNGSNSGGNGFGGQNRYNYRPRNVNMVASQGTTPDGASTSETSTSGQEDQADTDETRLPRSQEQNF

>AabHLH132

MALSFYTNWSNYDSSVTSLSWPSEASQELPCFHEASTFYDTINPNFDTNYTNNLDFLGLYSSRYPVEPTPNVFMQELQDPNYHTFPYSNTIQHENLLMEYTMGPELPSLVPPFLDSLTYQGNGSVAALPPWYNCGLQGQTQVVSGVKVKKQDANNEERSLTAQSLAARARRRKISEKTQELGKLIPGGQKMNTAEMFQAAFKYVKFLQAQIDVLKHMALLPESEEVLGNGDMQDLVTCASIQEKCIGPNTLQISSNDQH

>AabHLH133

MEGFTSSFHSLKPSFSFIDIEQNMELFNQFSIQYDNSSMSTSQSFMGISNDNFMSQQVSPSLDQQFVQSFQPVFQHEKKNVMVIPEAAPMGPVLNGKRKSMDVSSSSSGNSSSHLVADCEIDGKKYQSSGKGKKAKVSDNGEAPKEVVHVRARRGQATDSHSIAERIRRGKINERLRCLQDIVPGCYKSMGMAVMLDEIINYVQSLQNQVEFLSSKLSEASRFQNFYSESMHIDAFQMGNVIEGLKLQRLEENGPVDQSFGPYPSLPYHRT

>AabHLH134

MSHIAVERNRRRQMNEHLKVLRSMTPCFYIKRVGFYHGIVCPSLCQLALAYKSVFLKPETASPRVRQDPSNSQVLASYGSETNTIAIIRRSTGDQASIIGELGASCNSPVADVEAKISGSNVILRTVSRRIPGQVVKIVSLLEKLSLEILHLNISSMEDTVLYSFVIKIGLECPLSVEELAVEVQKSFSLNLT

>AabHLH135

MEGSIKCSASDLKKPERKIIEKNRRNQMKSLYSNLFSLIPPNIFSKDGDVSDRVDRAIEYIQMSKTNLDMLKNKKEKLSSRKRSHEHTKIIKNVCKPVDIQIHEISHDIDAVMVTGLDNHSSFCDVVWLLNRYSAEVTLATFSSNGHSTFNIRQKKIEGKDICKRLKTLLEGSLNVKELENNHALLTLPQPGLDTGIESGPQRIEESQINLQKELNELEYDSNLSIWDLDFQSNVWITCNELDYESNLSIWDFDFHPNVWGSELEVFQ

>AabHLH136

MYNETQFRMYHELKTFRMYHELKTVGGSIQYELHYIIHCSLDVVDERGIIIKPSYDRKEDGFGNSEPNESNRSNDGMKQEHMVYDPQGPYGLKTFRMYHELKTVGGSIQYELHYIVHCSLDVVDERAIRFRKIQNKIPGIQLFRLICPNVSSNVKLLHTVQSHNRRRSNSCSRAENKACRERQRREKLNERFVELSSTLEPDWSATTDKLAIIGDVIRVLNQLKAESQECKNMNEKILEEIKTFRQQVKTMTNNHLPPPGFMSPHPAAYQAVANKMPIFSGYGYIPMWEYLPQTMCDTSHDQELRPPAA

>AabHLH137

MDFSLRLLRLQDKVVWLEHQGVQNTSFRDTYESYGLSPRIDSYEQPPCLGSRFSEANRKSDLVYQGIVDRAVAMNHKLDEALELCSLKVKKQVASNEERILGAKSFAVRVRRRNISEKTQELGKLIPSDQKMNTPEMFQATFKYIKFMQTQIGVLIPYGLTSGNLQQEAVR

>AabHLH138

MALSFYTNWSNHDSSVTSLFWPSEASQELLCFQEDTTFYDTINPIFDTNYTNNLDFSGLFSSKYPVEPTSNVFKLELKYPNYHTFPYSSTFQHGNLLMEYTMGPELPSLLPPFLDSLTYQGSGSVVALPPWYNCGLQGQTQVEEISSVKVKKQDASNEERILTAQSLAARARRRKISKKTQELGKLIPGGQKMNTAEMFQAAFKYVKFLQAQIGVLKHMALLQESEEVLGNVEMQNLVNSALIQEKLYTAEKCIGPNTLQKPSNHDQH

>AabHLH139

MLHREIERQRRQDMTKLNGSLRNLLPIEFVKGNRSISDHRHQAMQYIKQMEENVKGLSTRRDKLKNNKSSSSMNHLHNTVSVNLCNGGVDILINSCTIEDGFHLSLVLKALDEEGLNVTSCTSTKANDRLLHAIQSEENLASLDLSMLQQRLTFVANTQPNYY

>AabHLH140

MVGNNKCSTSRLKKPERKIIERNRRNQMKFLYSNLFSLIPLNLFSKDGDVSDRVDRAIEYIQMSKTKLDLLKNKREKLSSRKRSHEHTKMINNVCKPVDIQIHEISHDIDAVMVTGLDNHSSFCDVVCLLNRYSAEVTQANFSSYGHSTFHIRKKKVEGQDICKRLKSLLEGSLNVKELENNHALFSVPVTLPQPGLDTGIESGPQRHEESQINLQKELSSQSCNELEYDSNLSIWDLDFQSNVWITCNELDYESN

>AabHLH141

MDASLKCSPSKLKKKPERKIIEKNRRNQMKFLYSHLFSLIPPNYLSKVGDVSDRVDSAIEYIQALKTNLDIIKNKKDKLSSQERSHEHTKMIHNVCNTIDIQIHEMISHDTDAVLVTGLKNYSKFRDVVWFLNQCTTEVTLANFSCTGHSTFHIRQKKVGAEAIRKRVKSLIEGSLNVKELENNYALFCTNVTFPQPRWGRTGSQLGEIEELQMNFQKEPSSVSFCNELDYESNISIWDFESNVTLQ

>AabHLH142

MKRPSGSSSHRPPAAMEKQRRDRMKNLYSKLASLLRLQSYERMPLLGLLEKATDSIKRWKEMVERLTARKKELENELRGAMSNEINLNVVQVSEMDSNLEVNLIIKSSNKRIELLRVLNIIEQGGAEIINYSLSSMGQNTHYTIHAQALYSRFGIDSSLIEYNLKQLVS

>AabHLH143

MQRPSGSSSLRPPAAMEKQRRDQMKDLYSKLASLLRLQSYERITLLGLLENATDSIKQWKETVDRLTARKNELENDLRGAMSKEINLHVVQVSEMDSNLEVNLIIRASNKKIEHSRVLSIIEQGGAEIKNYSLSCIGQNTHYTIHAQALHSRLGIDSSLIEYNLKQLVS

>AabHLH144

MADSWWDSRTTRPCLDSVSVSMNLFQETTTTTTTSGGGSDHGVVGNNPSLQMMELGLSSQPTSQSLDWNQALYRGDQRNEHHQSGYQTLIQDDHGLSSNTSNFQETQWKSHKMYSDSPSEYKQINARGFRLEEPVHYNDESGLNPSFQTLDHSYGSNSTVLQSLFGSDNNTNQDVADSCYDQNQGISYNSYQSSYGGITMSGGGGGGEYPPHPPQEFPVNSPPKVQPPNITPLHFSNNARFWNASAASMNDVRSSFFPSQMQSTSSTIEDKPKNPISEIVKKTISKSSSTKRPRNENPPLPAFKVRKEKMGDRITALQQLVSPFGKTDTASVLTEAIEYIKFLHEQVNVLSTPYMKNGAAPMQQQQQQIPDKPLEGSRQDLRSRGLCLVPISSTFPVTHETTVDFWTPSFGGTFR

>AabHLH145

MDASLKCSPSKLKKKPERKIIEKNRRNQMKFLYSHLFSLIPPNYLPKVGDMSDRVDSAIEYIQTLKTNLDIIKNKKDKLPSRNGSHKHTKMINNVCKPIDIQIHEMSHDTDAVLVTGLKIHSKFRDVVWFLNQCTTEVTLANFSCCGHSIFHIRQKKVGAEAICKRVKSLIEGSLNVKELENNYALFCTNVTLPQPRWGSTGTQPREIEELQMNFQKELSFVSSSNELDYESNISIWDFDFQSNLYHLCDILRYEIHSYPMHSNVYHNVKTFYPKKVYHNVESSIYTRKIIGLCIAQPMDVHNHIDFLSAFE

>AabHLH146

MVVAVVMSYVMFDPTYELSQIRTVIVLRHLSHMNKKTIKMSKKFRGKGEAKEYKREDTRAWEANSWWSKDEHTRXEVCSLKVKKQVASNEERILGAKSFAVRVRRRNISEKTQELGKLIPGGQKMNTPEMFQAAFKYVKFLQTQIGVLIPYGLTSGNLQQEAVR

>AabHLH147

MDASPTHHQHQLLLADQCLSSRNIATANTTSDNNEFIPHSFIELSAHGILDSSENNLQSSNSLMSIHDDKRGRKMPTTNSTSFNHVQTKVRREKISKKMKTLQAIVPGCDKITGKAHMLDEVINYVQSLQNEIQILSLKLASVNPIYDYEADLEAFTVKPHQIMTTNQQQQLSFHEYQMPCMLSQNHEEALWELEEQRQGLDDDLFAIINYNSLYEPYS

>AabHLH148

MACSLSDLVSLDTRFILYNMWVTLMKQLGGEDGGYSGEEGGVDVEKKAAVRGGRPRLEVAGGGVKGFQLEVPDNPKGLPHLSLPELDASRMITTIQAGAEEYKTGNKWAKSVNHVEAESRRRLNQQIYALRSVVPNVSKIDKACILADAIYNINELKGKVECLEYQLHSGKNYLRKMRRVKKQMAGL

>AabHLH149

MYSPRKRASKHQAHCDDELADLVLKLQALLPTSSSKGDGREKMATSKIIQETCNYIKRLQKEVNIIGERLSQLLDSMENDDLDMDILRNLLQQ

>AabHLH150

MGDQMSFDTLLQFINSLSGQIDLDTVLRDAEALCLCASEDGAASIPPGTPPSLPVEDEESIYQQEDEVCAQKEVLIRNQMFFMPKYPDLMIHHYHFFEETQVKDTLKYQCKSCFVFVLQQKLFGLSMAALSFYSNWSTFPQNRYETVVPQPPELSPFQDNLALFESNNNHFFTNNLYPCQNTIKYPTLSSSYNPISHQHYSHPLAVPSVFDHEEVFPMDYQMEPECYLNPLVDSNVLFDNEVFSPTEGMQPEYYSCNSYHSYPLVSEVCEQMQPELPPLPEIYQFGGGSSDVMPMSHDFDNGCNVVQGESTLQVKDNGDGGRKLSAQSMAARVRRRKISEKTMELGKLVPGGHRMTTAEMFQAAFKYIKFLQAQASVLQHMDSSPALGEELQAFVTNPSIQEKLYTAEKCIVTEKLGKTLTGDYEGTNN

>AabHLH151

MGAEKSPTCLQPSKKSPGKVPKRIHKAEREKLKREHLNELFLELAGALELSEQNSGKASILGETTRVVKDMVDQIKSLRKENAALLSESQYVTIERNELQDEASTLQNQISVLKSMIKEHTVQTNLDLNAPAIETQEPQLPQYFPQDIIRLPSGDPVLNPVFVIPPCQNIQINPQPVSNVSKPNPRYPTPSDSWPFQLLEKPSQEVGEIQHRERV

>AabHLH152

MIDLEAWWKKLLLFAGDENLSDETMLLETHENMVHEPKGNTLGEETSLGANLVTGEIMVRKPSENTLGEEKSLVPSIATNEKYAKKLDMGLIGGHDSGPNEVDDEAVREHPNEVDDGSVRARKKGKFVVTENDGTGQRDHRASKKAIHVKAVRKHRKLLNGLFKNLRVLLPQLPTKISTETLVEEAVSSIKSLEETRDSLEKHKLERLSTDTRMAPPAPSQTRVIENADSMMNKDVILGPTSHFSKNWCSSNISLSVFGANALVNICTVRNANFYSSISYILKKHNVDVLATSIHSDQAKTMYMMALRVNAPTEIAHMFLYEDLFKLAMNEIDYAYRQI

>AabHLH153

MASLSFCSNWPTLSQLRHETIVPQPLEISAFHDNFNLFEPNNNHFSTFNNNITNFPIQNTVKYPTLSASYNPISQQHYSNPLSPNVFDHEEFFPMEQMQPEYYSNPLFIPNVFDNQEFSPMEQMQIQPEYCSYNSFDSYPLVPDVCEQMHPEHCLYNSYDSYPIVPNVCEQMQPELLPLPEIYQNGSGSEDIMAMSYDTGHGCNVVNLDESLQVAKNGEGEGRKLSSQSMAARVRRRKISEKTMELGKLVPGGHRMTTAEMFQATFKYIKFLQAQVAVLQHMGSSPGLGEELQALITNSTVQEKLYTAGKCIVTEELGKFLADNHQGTKNNINN

>AabHLH154

MMSFKIDYFHLIVAAGKGDPMVVMADCEFKVEPYLSQTLRIKKQEVEKMNEMMFFRFARGFRKLVILLLDKEEGNDYHKEKLQEPSKDYIHVRARRGQATDSHCLAERVRREKISERMKLLQNLAPNCNKVTGKVLMLYEIINYMQSLQRQVEFLSMKLATVNPSLSTLTPMTYSRKIVHWFQEVPNGMYHYFSKVVPTIYTNIRGRTIQSNQFSVTEHYKSPEVGRQSLPGVFFFYDLSPIKVTFTETYASFLHFMTNVCGIVGGADLHCFKNRIELKWEDPVCAPGGKWTMTFPKSKSDTCCLYTLLPMIGEQFDHGDEICGAVANVRSRQEKIALWTKNASNEAPQTSIGKQWKELLDYNDTIGFIFHEDAKKLDRGAKNKYTACDIVYF

>AabHLH155

MIRNKTKENAKAEPPKDYIHVRARRGQATDSHSLAERVRREKISERMKFLQDLVPSCNKVTGKAFMLDESYVQSLQRQVEFLSMKLGTVNPRMDVSIESLLSKDMFLPRVSMPTNMNPFDASAQPFPYSFQPQNNGIVPDVSENQFSMNPLMAAMHRNSLMKPSHVDGLGECSQDGIRTGSLLNSSFYSFVGNPGLCIDYVLNNCDANLTRNLRRCTGASSKKLKGLSKFQTGMVALGTSAFLFAIVVGTGFFILQCRQREKHDTEEMYNDKREEDDILFQKVMEATEDLNDRYIIGRGAHGTVYKASLGSQDGVYAVKKLMFGGSNKEGSTSMIREIETVGKVYAYMVLSKDLVGLVR

>AabHLH156

MSSGCGHQQRKRRSVGKGHAGHNPAIENIRARNGGTEVSKFSVIWDFHQVKTSAHECYRNQHGGGDPGMCPIGVKEHLAPYLPSHPVTQKVVAKTKRNVNSKKQEATNTNNGETIYVVEEKKPEEGTYQIQSFDTGFWVVLYASIATSRLSFKYTRDMPLARSIHSYIQLESPDTGWIGFFPIVIRVEEVSSVKATKQDASNEERSLSAQILAARVRRRKISEKTQELAKLILGGQKMNTAEIFHYAFKYVKFLQDQIGVLKHMALLPDYFDSQQVNATNQEPGMKKMESSLRTSKAYGSLRVFISENMTVGLNDPIVRTEVAVMMKAHEGIRMDNGDISERTLDLAERKIRSKVDLSNDKSHEGHHLAARLIFLDSPLMLSSKDMNFDGYTYKNIEIVNYYQVPRMGDQCYLERFNDSSQHISICQLYNDLHKLGCRNAVILAIESMDQGSQRLLEHLSMKIRLVPCFGSHFFTVLPALDIHTTIFASSFTMEYELKGFKVWSTDLYFVHVKTQKAQLQLDAQLQKDARIIEPCFISIYMCFKKAKFTAFAQLGKAHGTVLGREQRQHGGNEDSTEGAVLILVLKGTEAKNVVDEFLFGLVEDLNMWNQFPWGSYVWPTLYSNLKDTAIKRSELHFAEGRDPNNLPKYMLNGFIWAFKVRRPPPRLRPDEVELLSEWWVHSKALFDGNPLPPPVRQPQVNSLMDDEIPPVILQRFKVYDAMLERHQLELKKLAELNNQAIHKETSFVGDEPGFSYFRVTQDTQAGPSFAPDMAVDTRVNY

>AabHLH157

MTTVECRANAVEPKHEFDSALALDIDRMMLTCEGSSKKPERKTLEKNRRIHMKGLCYKLNSLIPSLSSQPFKLTTQENQFDQATAYIKQLRKRIELLKEKRDQALRLVNNGRNNDASCSIESKQKAVSSWLPTVEVKEFEGALQVFLTSNFERKFSLPQVVRIVEDGGGEVVKGGYTIVGDKVIYTIHAKARVTRIGVDVTGVHKELQELINGSSCRSRLRFQGSGSWEVAGFF

>AabHLH158

MVVAVVMSYVMFDPTYELSQIRTVIVLRHLSQQYIMVEEVCSLKVKKQVASNEERILGAKSFAVRVRRRNISEKTQELGKLIPGGQKMNTPEMFQAAFKYVKFLQTQIGVLIPYGLTSGNLQQEAIQYDNIKSGRYHTLITYAFSHVSLLHMVANMVGLYSFGKSLGRIITPGSLLRLYIAGALGGAFAYLVDCSYRASEPAPSSEGSKPHPTKRIAVGASGAVTAMVCLDILLFPREKVLLYFIIPVPSLLAGIYMIWLDANRVKSSSNNAEPIMPQINTQIPNTSSFTNRSNTLTHRGNISTYTTSPQAFSNPIPGNRLLTKGNIGSKLFVGHEFRPLRHQFRRHFSIYSKRWVSKIQFSSFKSHTMHKLFSLKLITKNPSKFTPKPFKSNPVSHPVTNNVTHNYHHHHGFYSLPQAFQGTPTNCLIKVLSNPVRFNGVLAKFKGFVGQGGFGSLRDQFRRHGFQFNQPVSYQQTWLAQFRRRLTTDGVVIGLIVTNVAVFLLWRVADRRFMVQNFMIQLDNFKSGRFHTMITSAFSHNDVGHIVSNMIGLYFFGKSIGHQFGPEFLLMLYLAGAFVGSAFYLVHRAFLVPSSKDRRLFEPDPSKVPGLGASGAVNAIMLLDIFLNPTKTIYLEFIIPVPAILLGIFLVGHDMMRILEDRRLFEPDPSKVPGLGASGAVNAIMLLDIFLNPTKTIYLEFIIPVPAILLGIFLVGHDMMRILEGDSKISGSAHLGGAVVAAVAWARLRKVRILAKGLVQVFQQIIDQIGRVHQLVMLDD

>AabHLH159

MDPNLYLIWDEDEDDAKVSGDGDSSETVTTRNPKTKRRSGGGVKGDRTKSLISERKRRSGMKENLYALRTLVPNISKVNHFLPQIILMVTTPQMAFRDGSIWGQQKVVTKG

>AabHLH160

MADNWWDSSSRTRPSLDSLSVSNSINLFQDSETATAATTTTTAATSMGGGTGASTSLHMMGLGLSSPSLPQSLDWNQALVRGDQKGDGGFRNLLEDQDHSLSSSTDNFPLENNQWRQQKMYSASSQDSSSDFKQINVRGFQLDQPMHDSDNESIITCQGLNSSFQSMDLYGSPSTIMQSLFGSDNNQQQLGSRLDQNQGMSYSLYQSSYGGINMPGGGNGGGGGGGAELSTSNWSKFPPQPQEFVVNSPSKVQLADMSGSQLHFANNARFWNPSAGGVNDIRPPFFPSLQMQLPTSTFEDKPKIAPKVVKESTSESSSSSTKRPRTENQSPLPGAFKVKKEKMGDRITALQQLVSPFGKTDTASVLSEAIEYIKFLHEQVSVLSTPYMKNGAAMMQQQAATDKPPDGPRQDLRSRGLCLVPVSSTFPVTHETTVDFWTPTFGGTFR

>AabHLH161

MSKDGFFNTLPQSWNSMFGMELDSQVNEMNLFNHNWENSMDQSDPFESALSSIVSSPVNSHPGTGIRTPVPGCHAGAARFSCFGAKEGEFQHMVESGKMSRVSSNQSFIKTGGSVSRLSMNLNSDNKEVDHSVEVSSLSEQINGSETGIKGRKRKVISKGKSKETQVDNKVRREKISERMKFLQDLVPGCNKVTGKAVMLDEIINYVQSLQRQVEFLSMKLATVNPRTDVNMEALLSKDIFHSRPSMPNPMNHMEASAQPFYGMVNDGPENSLMAMMHHGSNMKSSQIDGFSEASAFWENDLQSVVQMGFVQNQGPSFHGTMGSGQMKVEL

>AabHLH162

MESANFHQQQHQEHQPVLDSSCYGRSWSQNPSLNNTSNTNSRENIHLVPCHNASPPMLGLPWNTTTSSNVTSNPIENFMTHELQRLARIKDEFSASESYPRFSEMINTSPTSSVEDLHLNPSPGYHNNQDFFLRTFSNECQIKGSLVMDQVPDDTQNNLYQNCSRGTFSQIFPTINVSNLNQSRAASVSSNSFDMNLPALDLFGSPRFNRNFSHPSSFNPHQLGSFFKDTCLSYGLDQMHQSNNRPAIFPSKISSAFNTSCTEAKRPATNYMDTKAPQATVPKKSKVEPRASCAPFKVRKEKLGDRIAALQQMVAPFGKTDTASVLMEAIGYIKFLQNQVETLSVPYMKSTQKNNRLPTQGVSLEEGNEEPKRDLRSRGLCLVPLSCLSYVTDGGGGVWPGP

>AabHLH163

MEGSDENVDLAKKKSVGRSSSSKKKDGVKVPRKIRKAEREKLKRDHLNDLFLDLTNALEPATQNIGKSSALTDTMRILRDLIAQVDSLKKENSTLLAESQYIAVERDELKEENSAIEAHIKKLQSQIDERMNPQSSWSSECNPVVGPVFMLPLQNDPKLYAEPKIPEFVTKSLGPNVSKPHARYPSASDSWPLNILSEQSRAA

>AabHLH164

MYGVNNSSDAISRDMNSILYSSTFKHPADTEFAKIKQLISLDNNNNSYENPSTHPQHQENSENPPLVSYRSTPSSFFSNLLNENENDAFQDHEPEEIYFMDQQQHKKKSDQSEAYNNMKREKQEMGSKNIEVLGYGYSKQSDLDCGSSFRSDLVRQSSSPAGFLSSLTGENAFAKDLRNGSSSSSFNSHISFSLGSSSSSSRFLPQIAENENELNDSTFHSLKRSRDGSLKMSQNGETVNHTPNLVHHVSLPKTSSEMAAVDNFLHFQQDSSVPWKTRAKRGFATHPRSIAERVRRTRISERIKRLQELFPDMDKVKFSSLKRKSCIELLKTRNQMIHFIYVCSKNCKHLSEMRFK

>AabHLH165

MFHQLPDLTSFENLNYPPSLPTFLSQPRKIPTSSAREKQQQKRRTLSEKTRALQKILPWDKKMDMGTLLEETYKYIKFLQAQVKVLELMPVDSSTTGSNFGCCVYSTEQLVMFKDLAEKNYASMLGILGMVKAKGEVKGGLVILEIATVVVNESIDIVISQNLGTLLKLTSTRWQVLYNRSIKLYVIYSEKILYILCVNLKGNLAKDSRFMYQYKGQPGKDFSFQNRRYHKFTSLTISYINQQRHGIQGDQQCTYIIHEHEPLILCALQSLNVNKIIKLHTVKTQCIPSSNIISSTKQDALLEIPK

>AabHLH166

MHRRTQLGVNDSWGKLRQTARDHLVMEIGDGNKASMWFDLAQIKTVNLDNGKSDVLKWKNRKGKLGKFTVSHGYNDLREDETDANWYKLVWFSQNIPQHAFVLWLAVQNKLTTQDVIKRWGSYDMMLKMGIREINLSWEGIVNKMADWGRRNLGWGHRNLGWVGSGVGRRLFAGEVQEYEEDELSTARTHRNDLKDTSDSCSSRLVKSRLALSSFSHKSSGSSERKREKMRKMVNTIREIVPSGKQMNSVAVIDEAVKYLKSLKVELQEVGVGI

>AabHLH167

MAKEMIDLEAWWKKLLLFAGDENLSDETMLLETHENMVHEPKGNTLGEETSLGANLVTDEIMVRKPSENTLGEEKSLVPSIATKEKYAEIIDLGLIGGHDSGPNEVDDGSVRARRKGKSVITEDDGTGRRDRRTSKKVIHLEAVREHPNEVDDGSVRARKKGKSIVTENDGTGRRDRQTSKKVIHLEAVREHPNEVDDGSVRTRKKGKFVVTKNDGTGQIDHRTSKKAIHVKAVRKHRKLLTGLFKNLRVLLPQLPTKISRETLVEEAVSSIKSLEETRDSLEKHKLERLSTDTRMAPPAPSKTRVFENADSMMNKDEILGPTSHFSKNWCSSNISLSVFGANALVNICTVRNANFYSSISYILKKHNVDVLATSIHSDQAKTMYMMTLRVNAPTEIAHMFPYEDLFKLAMNEIDYAYRQIFKAPKEDNMM

>AabHLH168

MIMFHQLPDLTSFENLNYPPSLPTFLSQPRKIPTSSAREKQQQKRGTLSEKTRALQKILPWDKKMDMGTLLEETYKYIKFLQAQVKVLELMPVDSSTTGSNFGSNEDDNVGYAAYGYGTYGYGCYGNGYGNNVYGGLGRLNRQQLLEVIVNSPVAQANLCSRGCCVYSTEQLVMFKDLAEKNYASMYY

>AabHLH169

MLAVIETKRVEPATKMMDHKRSPVSIEQGSLTSLTPKRQKAGLSMSSKERKEKVGERVAALQQLVSPYGKTDTASVLLEAMEYIHFLHDQVKVLSAPYLQSDPTDQYQELDSYNLRSKGLCLVPTSYTMGVASSNGADIWAPIKTQSPT

>AabHLH170

MAEEFQAAEVSWWNSPRTNFNGSPFMSTYAGGCWQNDFMNIKTRSTDESCGGYTATISPDSAFQITDSGSGSSPSTNTTWHQSLLVNGRNEESYNQTLPEIILNNLPVSGQEISSNFAMNQQETSNFMTNSGDCTENLVTTSYGYPSSLLQTLFESASPPTAAAPQQQALYDFEANLNNFNSVPSMPNGFSSIVKPKQQVLGGLHLANKTPYWNSSVLDLNDNRGGYIASTQPRFGSSTYEEKNSYPNMKSQNEEIRDLGSSVKKSSGEPTFKRPRLETPSPLPTFKVRKEKLGDRVTALQQLVSPFGKTDTASVLHEAIEYIKLLHDQVNVLSTPYMKNGATMQRQQIHDKVKDDTEGAKQDLRSRGLCLVPVSSTFPVTTETAQDYWTSSFGSTFK

>AabHLH171

MPQNEATTIDPISGGTFSRLLFSDADVDDAVTLLNNNNTFTFSSNDQKPPKMLCFGQDKYVTQNNPTTNDHDHDKSLLPSSTTTSLSSSSNMKRNMGCDYYQPVVLSTSVVTTTTGATVAPSGSNRRSNKKMKAENTPPAGHAKVKKEKLGERIAALQQIVSPYGKTDTASVLHEALGYIKFLQEQVQVLCSPYMKLIPATHNEGQEDGGGQRKKDLQSRGLCLVPVECTLHVAESNGADMWSAAMVNHHGSSALR

>AabHLH172

MSFIAFVTGSGIEGNVWWLKVNKRRRGFSMLRELIPHGGQKRDKASFLLEVYHERHVYFVHPKLTYRHIAKPKVLVCQIIEYIQFLQKKVHKFEDSCRGWNNEQPAMTAWVITCHK

>AabHLH173

MINSNRPTTLNKLSCLYIPNTPPPSPNQHKFQSLMYKFIMAEQCNQNSSSTAKWWPDVHASSLCSWTGGANYASNNLCNNSQTPNSNCSNGEEDVSISTSFTTNASNNSGLSMESSRRLVEKASTNDPYGEAVSDNHHLWNQVLLGVGTTGELQNTSSQMFEPACDYLKKIDNGWDFLSSTHPNQFQKNFNGVNNGLYQNKISQDVVISPQPDQYNGQFTAIKSERMESDVHREGIFRRGLSCHASEYQAGTNDVVLEDSNKYYDNGMSSDMECTNGRAFLDLVGYGSFLNKPVLDLESNVFNKPMMSTMNLPDRMKQGIHNSFQPIRPRGNTPTKSNERGNGIANEGKRKKSEDQSGSLKKPKLETSTVSSTKAQLPKAKLGEKITALQQIVSPFGKTDTASVLGEAICYIKCLQEQVQLLSNPYMKTNIIKDPWVRLETKDRGDMNLDLKTRGLCLVPLSRTPQVYHENNGSDYWTPAYRGYIYR

>AabHLH174

MNRGNVLQSSPVQQMIAGGPNGWWSMMATTRPSTPPPPQQPFFASSAQPHFFPHQYAPPLTIPSSQAPWHNNQENPDSLSQLLMSGLVSEEDKSALSHMQQVKKLENWEQQLLLRDHHRQQQQHNSLNAQSVEESTIKQENSLNYGSSYGQGNGEFHGTKPSNWSNQMIPVSSPTSCVSSVSNSMLDFSTTTSNKADGRHPPPDRSSECNSTATGGALKKAKIQPSSSQSTFKVRKEKLGDRITALHQLVSPFGKTDTASVLLEAIGYIRFLQSQIEALSLPYLGGGSAGNMRNHQHSVQQGERNCLFPEDPGQLVNDSCMKMKGAALDQDSHEEPKKDLRSRGLCLVPVSCTMQVGSDNGADYWAPALNGGFR

>AabHLH175

MDDLNEHVPQNDSDLQPYTSTLPETFGYVPRLAENTFYQQHIVQAKDCDIYSVPQWIGEYLAGNGLSNMQGQIPYDSTGIGCSSFTEVPFLSMNGHLAEPRDINSHVNFLSQQHEEVPVNNFDTTQYGIGYQKQGPNTGLNNAAQHLENMSVSTSGSGTKSGKKRVKYPETNCRRKTRLTAALDALENVLPRSREVNKETIVDDCEDYIKSLQLHMKELSQNRLRCEPTSNHLQYLEGYGHYLVHENTATGPLDDILGKLLKENPTAATKLLESKGLFMTTSTPN

>AabHLH176

MVCQSAGQTRFRALKHENGIDGSATIIVKVIACFQPLQDCQHFESWFQNQQSDPNLSSALFSFQHRRNIPYLGNTLSPNTININLPMFAFSASNPEEPRDWFNGLAPMAKSIPKQQLPEPQASKPQEPCDWFNGLTPMVKTIPIQQLPEPQASKPQESCDWFNGLTRMVNSIPKQQLPERQVTHGLQKKFLVFDQSNYRTTLIYSSTPAQYQIPKPQLFFNVKKESSVIENHLDTHFSIPFPDDGNTTASEMQEDSEELKALLYSDDESDYSEDEEEQSTGHSPDLIPGLDRHECKDDNLEEVASSTGCRKRPKHELEDTASSGKSGLNCSGDADLSSCGNNSGVRNGVSDVESETFPPVKRARKEKLWETINILQNLVPSGKSDNKDAMVILDEAIDYLRILKVKAIALGLDSL

>AabHLH177

MSPSINPTDHFKNQFATRFVHALNNINATKSSPHDDHKNMFQRSRRVKIAAYTAMASVSGSERAWSRALLWKIRNRSRNRGLLLRNKKRADHAKVSSKKRNPNPKRQDVNALRYSGQELKLRKIVPGAETMDSRSLMSETADYIKCLVAQVEVMKSLVDLCYTKV

>AabHLH178

MELPPQIGALKKLKLFDLEGTELMYLPKEIGKLETLECLRVSFSTYADDQKDRSGVEHIIPRMTISKLTKLKELSISVNADNEWWEVELLEGIMGDLIFLPDLKTIKLYLPTAKALQEFLSLERYKVPIYSNLWNFRFMIGRCEELPCSVQLDIEENFLKLEKCVKFMNGDGYTDETAELVRNARALYLRRHWTIGKLPIFDMKRVKYCLLMECNEMQTLFDQEDVYAHLDKATNDEDASLASLQYLGVHFMKKLQRLSKGPISSTSLSHLRILALHSCPEMSSIFTGSLLQNMQGLTELVVEDCPKFNCLVNLEDGTPCSSGPFLQSLRRVSLIDLPELVSISGGVSIAPQLDSLLVFNCLKLDYLSIMELTRDVKEIKGEIEWWDALKYGKMTWNNVFVPLKRDGNLLDLLAQDTNSLQHFLELFTAPSHAGSILQVDQNSSRDHVDQLQIEHDVLLSNETQKMSSQDIFNSNKATQSLDAGTAVRMWEPQYPVIGKLDNEDMDCDFDVPNYKPASIKNKRRSISFITSKHQVKVNKGIEVEKALDDGYTWRKYGQKEILNAKYPRAYYRCSFGSTHGCCAKKNVQRSTEDPSVFEVMYIGYHTCPTILMTATESSCRILRTKDSSTATESDSTNTYSRFEHPKRPQMLESNKISKSGRGARLTQHVGVSPKTAGFETPPDDGYKWRRYGQREILGAKYSSSYYRCTSCSTKKRAQRSDDDPSIWEITYKGTHTCLGPSIARNFASTTESEISIIKNSDSSTITKSASVITENSVGLEFTKNMNLQSSTKELQNSKRRPSPFSKARKEKLGDRITALQVLVSPFGKTDTASVLFETCAYIRFLHEQVSVLMTPYTTKGAPLELQQKVTKRDLRNQGLCLVPVSTCEHLAGCYSNYSDFAISTSAKNPNESLAHEQLFQM

>AabHLH179

MGMEAVAGDGDNDDEEIILSWGEKCGNVPFLPDYGPLSVTDWGLLNSSGMSYGVQPFFSSQFYNFGNMNEASSSSTFPEELEDMIQEEKVTFYTNDFRLEPELYNYYQQIDHRAVDVPLQGPMIDYQSSGVNNNHGAILQQPFTPDMKHSDWRYEQNSRVMENPKKRMNNVRDQKVVNKKHARHNKMQGIEGVTERVPMRRSQKLADKITALQKLVSPYGKTDTASVLQEAHISINLLHGQIQKLLQSTQTPAINIGPIQNRNNKEAESSLRDKGLCLVPVSTLQVNSIYHGEQNFISGNY

>AabHLH180

MHNSSQLKKEFIKNWMKGLHICYSSNKQMKLLERKKKIKISADIALACAKNATTSWSKALIAEAKKDEQNKILIDNLACPESEIKGFHQKVMTCHKRIRCKKILKRSHGVVKRTKKLKPRRSDLAIRVAKRLVKKRTQVLKGLVPGGESMDEFSIIKEALDYILSLKVQVDVMKNLVNAANVLN

>AabHLH181

MDNNGGFTKEKSSLRTERKVRRTSRRINTTVLRKVKKLQKLIPGGKGLNADRLFDHTASYIMHLKLQVDVLQALSDVYRP

>AabHLH182

MMASSSDPISNNIERVRDSSKRRKKKKIKSNNDIAKNKDEITQWKSETQQQVYSSKLLQALRHVRISSGVALKSAPHRGRAVREAADRVLAVTAKGRTRWSRAILTNKLKLKFMKKNRRQKGSIVTPTSNSRIKKPRVSILRLKTKNLPAVQRKTRVLGRLVPGCRKQSLPVVLEEASDYIAALEMQVKAMAALAQLLSGGSGSNSGATGDMISSHRQPPPSL

>AabHLH183

MSSSTVSQIPLRDSSKRRKRKKLQQLQSHNNDQITKQNNKIQWKSDAQQQIYSSKLLQALRHVRRSTGTSTGTSSTTAGAGTSARKHAVRETADRVLAVTAKGRTRWSRAILTNKLKIKNLKRSRRERGLIVSSAGSANSRLKKPRVSILRLKTKNLPAVQRKTRVLGGLVPGCKKQSLPVVLEEATDYIPALEMQVKALAALVELLSGGSSSGLSSGSGAGDGGGGLNLLSYRPQGRL

>AabHLH184

MSPPHQPTSINPNSLKIQLAYRFLHNLNNINTKRSNLDHTQINRKSHRVKVAAYASMAFVTGSRRAWSRSILRKIRNRGVLARIKKRVDHKASRVRVHHHLNARSTKRRNPSCSNPNGDYIDPSGNLGLEVKLRKLVPGAVMMDACGLLDETADYIKCLATQVEVMRTLVDLYSTI

>AabHLH185

MPRKNSKMVSRENKKAALHDKLQLLRSVTNSHAKEDSSIIIDASKYIQELKQKIDMLNQDVAQSSSYQNAWPMVTVENLDKGIQVNVYSERSCPGLVVFVLKVFEDLNLNVLEARVSCTGSFQLEALGIEVIYVFLYVIALLIASMISIASILEKKKQKYNLKWFNKLLKIKALKHI

>AabHLH186

MPRKNSKMVSRENKKAALHDKLQLLRSVTNSHAKEDSSIIIDASKYIQELKQKIDMLNQDVAQSSSYQNAWPMVTVENLDKGIQVNVYSERSCPGLVVFVLKVFEDLNLNVLEARVSCTGSFQLEALGIENDENGECIDSHLVKQAVLQAIEEWSESNDQEC

>AabHLH187

MHTSSKLKKEFFKKWMQGLQICCSSKKQMNIMERKKKIKLSSDIAMASAKMTPTSWSNALISNAKKCEQDTVLVDKLIGPQSQLKLQKTSNKMISFHNRVQCKKVLKRSCNYAAKKTKRMGDSRKNLATIIAKRLVKKRTKVLKRLVPGGESLDEFSLIKEALDYILSLKVQVDVMRSVVNATEVVLNGDNLMKSV

>AabHLH188

MSTTKKKMSRQYSCVRQQAMQNISNNNESTSWKSKYQQELYRSKLVQALRQIQLSSSPLSGHVVHEAANQVLAMTAKGHTRWSQAILTNKVIRKARRVVVPTMVAKRRFKKRRVGILRLKSKNLSAVQRKARDLRHLVPGCRKQPLSVVLDEVTDYICAVEMQVKAMATLADLFSSINQPPSSS

>AabHLH189

MESANFYHHQQQDNQLVDTSFHWSQNPILNGANNNINSRNLDTSINSMVEDMGFPYNGIGYPIDNLMTQELQRLARIKEEFSVAESYPKFLELLNGSPTTSSIEDLRSQPYSTTYMKTDHHDQQISYSNYNQDFLLTNLLNGCQIKGGQLVNDQRSSNGTLSQIFPTISISSLKQSTTSSSSSSSSAISSSSFDMNCLPALDHFGSPRFDASFSRPSSLNANNLGGFPYGLDRMNQPNHKPAVCPSKISSVSTTGSCTYQSAKRPASKYIDEKVTQPIAQKKSKSETRAPCAPFQVRKEKLGDRIAAIQQMVAPFGKTDTASVLMEAIGYIKFLHTQVETLSVPYMKSTNKICGISSQGGRVEDGVTTKAKRDLQSRGLCLVPLSCLSYVTDGCEGIWPPH

>AabHLH190

MTLFLFQKVGVIHSNHKCASNRVGRDSERLDLSVLRTEVSGSNPNALADHHQHENQDFPVSWSQLLMTGLANDQEQHNLVAGHVGEVKHGYLDNQQSRQPFYSHKSDHQDQNNSIEDDQQGCSSTWSSQLNPYDDTSTSSGASKKPRFQSSSTQASPVVRKEKLGDRISALHQLVSPFGKTDTASVLFEATAQIRHLEGQIETLTSPYMNLANNVSGATRHQHSSDGNALQRNLTSRGLCLVPVNCIDHIDTSNMTNNGTEFWTPALGGGL

>AabHLH191

MYTKLSMLNFFVVGNIKMHTSSKQLKKDFIKKWVKGLEICCSSNKKMDVMERKKKIKLCADIALASAKNATTSWSNALIYNAKKDDENAILVDNLLGPESRFNSQRTAHQMITFHKRVRSKRILKKSCTVSQRMKKASPPASNLATCIAKRLVKKRTQVLKKLVPGGEAMDEYSLIKEALDYILSLRVQVDVMRSLANATEVLD

>AaHLH1

MELSQPRPRGEPGAKPTHDFLSLYSPAHQDPSPTIPGSYLKTHNFLQPLEQVGKTVCEEVEYIKKSFPPSPPTVGEHILPGGMGTYSISHIPHINQTQKVSKPEGIVISAAQSSSSNNNDENSNCSSYTGSGFTLWEESNVNKGKTRKENNIAANRHTMRDGGMKFGVPWMTSIEQPSKSSSINNHPSTTRSSAPKSQYFVDMKSGRNFQEVEDINEGFSMLRGIIPHGDQKRDKASFLLEVIEYIQFLQEKVHKYEDSSQGWTNEPPKTIPINDFIHQPQIPTNQNLLDSNMISHDATNETSQHPQSTNKPSASAAASYEQISTPDTATTASQAQELIIEGGTISISTIYSQGLASALTQALNSSGVDLSHANISVQIDLGKRSNANTNTLESSPHNLKENETRSNDQSIAHSRLVSTWEDEKDQGFKRLKTSRN

>AaHLH2

MRRFQILRDLIPNSDQKRDTASFLLEVIEYVQYLQERVQKHEGSYQGYSAEPTKLMPWRNSHWRVPNFGHPPVLRNDSGSAPSLPVRFDETVSTINTPPQIPARSDPSGDLNRNLIDSQPDLQANIPVPDEYVQYLQERVQKHEGSYQGYSAEPTKLMPWRNSHWRVPNFGHPPVLRNDSGSAPSLPVRFDETVSTINTPPQIPARSDPSGDLNRNLIDSQPDLQANIPVPDGTLSHSTLGGSVCHVNRQSTDIPAAGDGNQQDELTVEGGTINISSVYSEG

>AaHLH3

MDIASKYEVKAMPTFLLIKEGVVVGRLVGANPEEIKKRIETHLQSNTHLIDHFPVPGGELPSLEPGFHWSANSFPGSTSVVRFQELNEILDPGRSAKTDKTVILADAIRMITHLRNEATNLKDSSQDLLVKINELKVEKNELRDEKQKLKTDKERLEQHLKSTFCGPPTAFYPPAHPVMPVPCPGPTPVGGNKFMPYMGFQGVPMWQFAPPAAVDTSKDHVHRSPLA

>AaHLH4

MTTSKFVELALILEPGKPPKIDKAAILVDAVRKLAQLRNEVQKLIDSNTEIQENIK

>AaHLH5

MHQQQPTPPLVPMLADYGVAEITWENGQPAMHGLGRANETLESIVHQATTCYNQTQYPEIDLQQSQSLPRARNLSSNVASSSRPTYLRKRPRESVIIHDQCVGNLGNASLQEDNVSNSGTVNSKDNDTTMMTWPSFDSPNQSMKSQKTDDDSACQYGSENQEEECRTEGETIRSQSSRRSRAAAIHNQSERTDKASLLDEVIDYLKKLQSQVQLMKNMPFTPQQMMMSMPLQLQQQQHQHQQQQQLQMSMLARMGMGFGLQMGMPGVIPQPVHNPFMVPQTMLSPAHVGTTSQTIHSRPSTNTPVPFNDPHSTFLAQQMNMDMYNNMAAFYRQQVNQGKSMSVDSSQLDHVRGE

>AaHLH6

MKEVEVKEVDTEEEGKRKKERAAKVDIEPEAVPGIGFGKDSGTEVMSLVAKSDSAYEGGPPADYYYSKSYFASRFVELGSTLEPDQSATTDRLAIIGDVIRVLNQLKSEFQECKEMNEKLLEEIKTFRAEMVELRKE

>AaHLH7

MGSSIMFSECLNFEDCGVSLLRFVELSSTLEPGRSVTTDKLAIIGDVIRVLNQLKSESQECKEMNEKLLEEIKTFRIIEFSSVPVTVSSDAAEKQSIPLVALKCILELLFE

>AaHLH8

MAFVSFKFVELGSTLEPDRSATTDKLAIIGDVIRVLNQLKSEIQECKEMNEKLLEEIKIFRGEDELIMKEGASNDQNVHYGNCFAFCVDKAEVVGYFHLYDLLAMWENKLGRPPTKKLLSRKAPHQKYSTCTTTTEFLGTDDGHEEVLAAVNATIKPGGCALSSPFWRLMDPLFGFVFDAGVAYVKQEVMAY

>AaHLH9

MKSFYSNLFSLIPPNVFSKEGDVSDRVDRAIEYIQMSKTHLDMLKNKKEKLSASRKRSHEHTKMINYVCKQVDVQIHEMSHDIDAVLVTGFENHSSFCDVLWLLNRYSAEVTLAMFSSNGYSTFHIRKKKIEAQDICKRLMSLLEGSLNVKELENNHALFCVPVTLPQPAGWDTRMESGPQRIEESHINLQNELNSLSCNELEYDSNLSIWDLDFQSNVWITCNELDYESNLSMYGDLS

>AaHLH10

MTAGAPKFYPLTPPPSSLPSRINEELCATTMPSPTGALLDVASVLGDAIKYLKELLQKINDLNHELKETP

>AaHLH11

MTTSRFVELASILEPEKPPKIDKAAIFVDVVRKLAQLRNEVQKLIDSNTEIQENIRDEGNPIAMFFLYYTSITNEKTDLRDEKQRLKAKKEDLEQKVKSMNTQPNFMIPPPGIQAANSFTRHISSFVAPLLACDIAKPTERFGVCWIFTSIFYDRKEVFAITSSLYHSWFYDARKKCNKSANHVKNGASSSGATKNKNAKSAKQANTCDEHGIIYQVVPRF

>AaMyc-bHLH1

MTMNIWNSDDNAMMDAFMSSDMFSIWGTPATPAGAPAGPGLVVPPASSSASTSAAIGSEWNQDTLQQRLQGLIDNARESWTYAIFWQSSGVDYTAPSVLGWGDGYYKGEVNKPKTVESVTSLAEQEHRKKVLRELNSLISGSHSQENETIDEEVTDTEWFFLISMTQSFVNGHGLPGQAMFSNQPVWITGRDRLSVSHCERARQGQVFGIQTIVCLPSSNGVVELGSTELIFQSLDLINQVKVLFNFGNSPPDLAPLDADDQDQNTDPSSMWLNDPPPVSSGTTVNTVEMKDTGDVRAVVPPKETSVVPLNNSVHTENTTKSVVNNSNHQGLSGSRELNFSSGFGCKPESVELVNFGENNKKKKAPALRGINEGGMMSFSSGMVVPPSDTVKSGGVMSAADFDQSDIEASIGREVESRLVVEPEKKPRKRGRKPANGREEPLNHVEAERQRREKLNQKFYALRAVVPNVSKMDKASLLGDAISYINDLKSKLDSTTTDKEELKDQLDAMKKELLSKDSHQSSSSTVSPPEDLMTPNPSNPILNDLDIDVKIIGWDAMLRIQSGKKNHPSARLMAALKDLDLEVHHASVSVVNELMMQQATVKMGSRFYTEDQLRIALTNRMSDPR

>AaMyc-bHLH2

MSIESFNDEDKAIVASVLGTKAYDYLISSSVTNESLLTSLASNDDNLQNKLSDLVENVSLGNFSWNYAIFWQISRSKTGELVLVWGDGCCREPREGEEFDIARILSIRLEDENQQRVKKRVLQKLHVLFGGLDEDNYAFGLDRVTDTEMFFLISMYFSFPQGQGGPGKCFSSGKHFWYSDALKSSSDYCFRSNLAKSAGIQTVVLVPTDGGVVEVGSIRSIPENMDLLHSVRSSFSLKPNNGLVAAAPLMGNAQLSSALIGERKNENGHGGHFLDLGLVDHQLKASKVVRQDMGLSLRQPQFREKLAVRKAEEPRSPWEGYPVTNSRLLASNTRNRITGSNWGQFTSPQEEFQLNSFRPQKSPTEMQIDFTGAVSRPSVVSRPVSGDSEASDVEASGRDERAVLTGLTDDKRPRKRGRKPANGREEPLNHVEAERQRREKLNQRFYALRAVVPNISKMDKASLLGDAITYITDLQKKLKEMESERSGSHGSTSMETPNNSNNGSSLEKIEIEADKDQVTVRVSCPVDTHPISKVIQAFKEAQIRVVDSKMAAANDKVFHIFVIKSQGPEQLTKEKLMAVFSKESSSSLNSLP

>AaMyc-bHLH3

MTEYRMNHWTPDENASMLDAFDMSSIWANNSNQTQTVPPTSSSASTSTVHQEFNQDTLQQRLQGLIDTARESWTYAIFWQSSVLEYSGPPILGWGDGYYKGEPNKPKTVMSATSLAEQQYRKKVLRELNSMISGTQAPENDAVDEEVTDTEWFFLISMTQSFVNGVGLPGQAMVTNQPVWVAGRERLMTSHCERARQGQGFGLQTIVCIPSADGVIELGSTELMYQSSDIMKKVRGSFNFNQARDPMQDTVGVIGSEMMTSVIPSVNKSHVPKQLPFENPNTLSQNPRSGHNFGSRELNFSEFRSMEGGSGGKNANSSYTKPEPGKLLNFGESKRSITNNGALSPTSCGSNEDGMLSFVSGTAKSGAGPFTGADSDHSDLDASMIKEVESSRVVEPEKKPRKRGRKPANGREEPLNHVEAERQRREKLNQRFYALRAVVPNVSKMDKASLLGDAILYIKELKSKVDNTQCDKEELRNQLEALKKELLSKDSRQSSSSAISLPDEMKMSTHSLIADLDVDVKVIGWDAMIRIQCNKKNHPAARLMAVFKELDFEVNHASVSIVNDLMIQQATVKMGSRLYSQDQLRVALTNGFSDPL

>AaMyc-bHLH4

MKSEASMVIGEENLSEEDKSIISSVLGSKAFNYLLSASFANECSFTSLANDENLQHKLSHLVDNPNSARFSWNYAIFWQVSRSKSGELVLGWGDGSCREPKEGEEFDIGRILRFRLENDEKQQMMKKNVLQKVHGLFGGLDEDNYAYGLDKVTDTEMFFLLSMYFLFRNGEGGPGRCFGQGRDVWISDALSSGSDYCFRSNLAKAAGIQTVVMVPTDIGVLEVGSVRSIPENPGVLQSIRASFSGNCVANGYGHPDRVISTIKMSKIFGQDFSSGMNQPQFREKIAVRKPEEPARVPFSNWAKIPGPQLQIDFTGITESKVSDECLVRDDREMVTTIIDEKRPRKRGRKPANGREEPLNHVEAERQRREKLNQRFYALRAVVPNISKMDKASLLGDAITYITDLQKKLKEMESERQSPNYKVSLEKIEVQATENEVLVRVTSPLEAHPVSKVIQTFEESKIKVVESKMNAINDLVFHTFVVKSQGPEQLTKESLISAFSRETSSS

>AaMyc-bHLH5

MGDKFRLEEEVKGMLDSVLGREAREFFVWSASNKAPDEFRSKTSDLGVQEGLHKILEGSDWNYVVFWQVSNSKSGKSALIWGDGHRKESKEHEEYNNNRDETVKRMRVLQTLHSCFNGSHEGNIASKMDSVSDLDMLYLTSMYYLFPFDKPSSPSQSFNTSRSVWASDAKSCEEHYHSRSFLAKLARVQTLVLVPVKRGVLEIGSFKSIPEDQTFVSTVKTLFNGYHPPKVLPKIFGQELSLGAKSAPKSGPISISFSPKVEDDMEFGSESYEIQPSFGGNSSNGNQSMVLNSQALVSGLDQSNQDLLTDRKPRKRGRKPANGREEPLNHVEAERQRREKLNQRFYALRAVVPNISKMDKASLLGDAISYITDLQSKIRILEAEKEVTGAPEVDFMARKDDAVLRVSCPLDEHPVARVIKTFREHQIVTQDTNVTVEDGKVIHTFSFQGLAGGAAEELKEKLDTVLLD

>AaMyc-bHLH6

MKCTIFLTSLFILHHLLLYIISIRCNKIQTIRYELIEMDDLIVSSSSSSSIVSIPSTTINPNQCDTLQQKLQTLLQNQPQPWAYAIFWQTFNDESNGSVSLSWGDGHFLSSNDILPDSFLPDSDLDCRKSVVREIKALLGPDNREDVEWFYVISLTRSFMPGDGSVPGTALGSNSMIWLSGVDQLQSFSCERAKEAQVHGLETMVCIPTCNGVVEMGSYHFIEETWNLAYQAQSLFGFGGGSTKFNELNDGHHNIISFADMVLMTSGLHDEQDEGIKVLGFEANTPDDEMSKNGGKLRTNMNNVVITNTYIETGSEHSDSDCQLVLATSEKRSQRKKGKNTRGRCPPVNHVEAERQRREKLNQRFYALRSVVPNVSRMDKASLLADAVCYISELKRKVECLESQLQHRNNQGKIKKVKTELPHTTDNTCNLYVSTKPILKNNNKANLTMNKMTSGFGEIEVKIVGEDAMIRVQSGNADLPTAKLMDALREMKGQIKHASMSCVNDIMLQDVVVRIPGATDSDELKSDLVRILDL

>AaMyc-bHLH7

MVRVLVWGDGYYNGSIKTRKTVQSIEVSTEEAALGRSEQLRELYDSLVAGDHLVTENPQATTIRRPSMALSPEDLTESEWFYLMCVSFSFPPGVGLVGEAYTKQQHLWLTGANQVDSKVFTRAILAKSANIQTVLCIPLLNGVIELGTTDKVEEAIELVQHDKTGMDAVMEANELLPLDMSEDIRFGSLNDDPNHLDSHCNLLAMIHDDSYRAESIPKWSDNLEFNEPTSIQLQVSGELSQGEDTHYSHTVLTLLNNQQLTQRSNFSTPSCQNSIQSVFATWTPNHLFPAKTTKTSQRVLKYMLCTVPYLHSTASPGDTSTVVGAAARHDEVSMNHVMAERRRREKLNERFVTLRSLVPLVTKMDKASILGDTIEYVKHLRKKVAELEARGCHAPQGKLPEKRKIRVVESGVTAVEVSIIESDALVEIECVHREGLLLDVMKKLREFGVEIVTVQSCVDGGICTAEMRAKVKMKGIRGKKISIMQVKKAINQIISP

>AaMyc-bHLH8

MGTRSPLRSFLQSLCENSCWNYAVFWKLQQQNQMILTWEDGCFGNMEAQDSIENMFAETSFQGLEETSSCNTYRGTSGGEAVELAVAYMSNFQYALGDGAVGDVAYTGNSQWVSAGSKTSGEINTTSISEHADEWVFQFAAGVKTILLVPVIPHGVLQLGSLDVVPEDAKMANYIKSEFVAHQDLTAYSDAFATNQQFSSQSPSSLMSMKSLDELLYDDVATIDNVNWSNHKHLTNGIEIPTCDNWYASHLAGIDMHNPSAGGQMGAIELTEPLHQLTTLINPDVIESSNSGCLEPLVCDVDALLSFPKECELHKALVPPFMGKTDDYSHHLSIGDDMYTSSHIFSEDPVDSLPKKTNGYLSCKEDVRNPLGNVVTSLHLGENSSIQTSGQSSIINSHGQYSSMAKRKNMHDKSAFEGESSLINNHVAPALCYNTAKNYNDSPSPSAISYEDVVDELAVEEERKNRYDGLHHNEGSKPSIASKRRGKPGAKQKARPRDRQLIQDRLKDLRELVPNGEKCSIDGLLDRTVKHMLFFERVGVRAIKLRQCLQSEGMGMGPKNNRTTEDKGSQNGASWAYELGGDINVCPILVEDLQYPGHMIIEMLCNESICFLEIAEVIHGLDLTILNGAMEMRSGDTWARYIVEAPRGFHRLDIFWPLMKLLQQPKQHSPISSKI

>AaMyc-bHLH9

MMGGGAQLRQLLKHLCVNTDWKYAVFWKLEHQDRMMLTCEDSYCVNNEKNNRLESNWFDNMGDNLKDGFYAKDLIGLAIAKMSYRVYSVGEGIVGQVADTGKHLWINGHQLVNRVCSLEEPLDGWKTQFRAGIRTVVVVDVVPYGVIQLGSLKTVTEDLKLVNNIREIFFELQNSLMSSTDCSPCVTDVSTRSDNRTTVEMINYHQERENPILDFIQSTLCNEQNRDLFMPGTSNDQINSNYHHDVGTETNESFVFPSGCELYEALGTAFCKQPYNFDWETATTETLKVDRIPEETSSSVLTQVSGSENLLEAVVARVCCSDSDDKRPMSFGQPVTCYSNERSLQGFSSAGVSKCSEQLDRSREPPKVGKKRARPGESRKPRPRDRQLIQDRLKELRQLVPNGSKCSIDSLLEQTIRHMVFMQSATKHAAKMDRYAEFKLLGKETGIQGSSWAMEVGTEIKVCPIIVENIGTDGQMLVEMMCDEYVHFLEITEAIRNLGLTILKGATEACGDKNWMCFVVEGDNNKSIHRVDILWSLIQILELKTKT

>AaMyc-bHLH10

MLLLNSTTMAQSSLQTRLKFILQNRPERWLYTIFWQASKKTDDHLVLEWADGYFPETNYVFGLDDVSDTQWLIMSSLGMCFPAGHDVVGKCFGSRSCVWLAGDIELGKYDSKRCEEVRVHGIKSLVCIPTNNGVVELGCCDVVQQDCGLIELTKSVFDPNSLPNINFYNLVDESEFPNQGLQNQVSEGQEEVMSTKKMRMSSSDSDPLEINSSSSVTTKNTCTPKRKGRRVKGTIAQPEVLAPGYHVEAERQRREKLNHRFYALRSVVPYVSKMDKASLLADAVTYINELKSKIQTLENKKGSESSLIRPRNENQLNVNQCNHDHDKRTIGHSTVNQLEVEVKLLESEAMIRVQSAEVNHPACKLMDALRSLNLKVHYASVSCVKDMMLQDVIVNLPNGFASEEDTLRLAILNKMCLD

>AaMyc-bHLH11

MEGSALHQALKTLCLNTDWNYAVFWKPDPHARMMLTCEDAYYDQNDPSGSKPFNKGMDNLPDQQYAQDILDLAVAKMCSRRYSLGEGIVGQVAITDKHMWIFGDQYVNNPDLSFEDYDELKIQFATGITTIAVVGVVPHGVVQLGSSNHIPEDLKMVNHIRDIILDLQNSLMGCTPSNTSSYNDTITSLNYGANTISDLDRNVDLCMPGSSNVQSETDSMNTLKTSFKFPAGYELYEALGPAFYTQNDNHSYQKLEEMPGTSSSNLLTPNPGSEHLLEAVVANVSQSDSEFSNSVKWVETHFNDMQTSTSGCYSFESSLGFSSASHSRCSDQRVMSQESPANVTKKRAKPGESSRPRPRDRQLIQDRIKELRELVPNGSKCSIDSLLERTIKHMLFMQSVTKHADKIDKYAESKLLSKETGIRGTSSHEQGSSWAMEVGNDMKVCPVTVENIGTNGQMLVEMMCEEGVHFLEIADAIRSLGLTILKGVAEPDGDKMWMCFVVEGQNNRNVHRMDILWLLVQILQSKAKS

>AaMyc-bHLH12

MAESSLQTRLKFILQNRPERWLYAIFWQASKKTDDHLVLEWADGYFPETNYVFGLDDVSDTQWLIISSLGMCFTAGHDVVGQCFGSRSCIWLAGDIELGKYDSKRCEEVRVHGIKSLGCIPTNNGVVELGCCDVVQQDCGLIELTKSVFDPNSSSNINFHNLVDECEFPNQGEGQEEVMSRKKMKMSSSDSDQLEINSSPSLRTKNTCTPKRKGRRVKGTIAQPEVLAHGYHVDAERQRREKLNRRFYALRSVVPYVSKMDKASLLADAVTYINELKSKIQTLENNKGSESSLIRSRNDNQLIINQCNHVHDQRTTGHSTASQVEVEVKLLDSEAMIRVQSAEVDHPACKLMDALRSLNLKDLMLQDVLVKVPNGFTIEEDTLRLAILNKIACEEVPGDLYPSLGYNTWEKSVPNL

>AaMyc-bHLH13

MGYLLKEALKTLCGVNNQWSYAIFWKFGCQNPKLLIWEECYYEPVIYSTGEHGIQATNNIHLLVTKMMKDNYVNLLGEGLVGRVAFTRNLQWITSDNKMLDVHPPEVSNELCTQFSAGIQTIAVIPVYPHGVVQLGSSSIIMENMGFVNDVKSLISQLGCVPGALLSENFMKSEGTSDSSPSQRESICQMNSVSAHSSGKSKVTDYNQQGHSPQASGSSGHTSFSLNRHIGANAVASGSTRVISDEDQCQAKVIHIMKQNPSFKTNQQIGITGVKSITGASLYNQNSKKEQQNAFGSCLLKYDPLTLMEQDNLSDAFLGDHANDTMMSQIAQTSIPDAHFSCTHVDKENLDNELFQALSVSPEEACLKPSTRDDLFDIVGMDFKSILFGGTRTNFVNNGVEASPLNLKRNDSVVLEPFSVYEGESDSGIYSSTPSDHLLDAVVSKVYSSSGQIADDNMSCSSLTKTNSSSSSMWANKGINLMQKELTGVPKSLKNEGLTNSCSFKSVIFEDGGNFSDTSSIISSQISSWNEKLRKDSGGASQYGKRADETSKSNRKRLKPGENPRPRPKDRQMIQDRVKELREIIPNGAKCSIDTLLERTIKHMLFLQSVTKHADKLKQTGESKIASKDGGLLLRDNFEGGATWAYEVGSQSMVCPIIVEDLNSPRQMLVEMLCEERGSFLEIADVVKGLGLTILKGVMESRNGKIWAHFTVEANRDVTRMEIFISLISLFDPAAKNDASAAGCDANGNISVHEQSFNQLATNPATVGPHNIIQ

>AaMyc-bHLH14

MIYQNLVRSIYSPSRISIIFASHTFCFHNQTIRITMDDEILILSPSSCSSIIHQNTHNSHHKLQFLLQTQPYPWAFAIFWKTTYNHDYGRPWTLTWADGYFLQNPNKPAFKEIQTLVGPDNTDGAEWFYVVSLARSFGIGDESAPSNSFTSNSVIWLTGAHSLISFNCERAKEAYIHGLETLVYIPTTNGVVELGSYHVINHTESDLAHRVKSLFSASSSSSSSFLSPSSNLINQPNDYLISFGEMVSRLPQDEESKDIFDLGTTTFGQQSKKLGKVVENTNNKGQKKSQKKKGHDPPLNHVEAERKRLTNLNQRFYALRSVVPYVSKMDKTSLLEDAVCYINELKNKVEELEAQLQAMNNQPKLKKIKVKAPDVTFVRNSQGANIYNNGNRTKTNGTLEVEVRMVGDNAMIRIQSGNADWPSAKLMDALREMEAKVHQASMSCINDVTLQDVVARVQELMEGIKQYPLHSYGLINSPKILADIVESLVGAVYVDTNLSVDATWEIGIKLEYKNLWVESGEIEIYNGNELIGKGNYKKKKTTMKNKAAADAYVNLVKQLGLKDDAQFDEFLH

>AaMyc-bHLH15

MDTATTHKDSAVKNVLKNLCTSYGWSYAVFWNFDQPNSILLTMQDAYFEVEIQGLISDLLQQTPMLGGGLIGQAALTKKHIWMSSEDNYIGQSSSGSIWDMFQDDSKFFHQFSSGVKTIAAIPVEPQGVVQFGSIEKILEKTEFIHQTKRMFGEIVNGNVPAISSCTSNGLFASLISSDDSYFGGAAQYSVNQTSLDLPPQSFAFGSQSQPMFLDNIQSTAHLQPNTVPWSTSSSLTSFNEPLQSTNTHQDLFDLPMDFGILDEMFQTGDFNISQLLPQSPGQSNVTGLLPISSNEKFNSLTISGVDVDLIGSTGDLGDIVTPVVKENQFGFDSYKSQNIDNDSAPKKGLFSNLGIKELFEGISGTSSSCIEDQVSKRRKTGSSFSEMSSLQPGVYNKSFGQKSEPILKSEPWMGSAYSMDGSSTVLQAKTQVEPAKPTKKKAKPGTRPRPKDRQMILDRMAELRELIPNGEKMSIDCLLDRTIKHMLFLQSVTKHADRIKQADEPKHNGVIPNNLSNDPNNNGVTWACEVGNQSMICPLIVEDLSTPGQMLIEMICEEHGFFLEIVDIIRGFGLTILKGIMESREEKIWARFIVEAEAKRHVTRHEIFAALVQLLQTMGSNDKHLEKKIMQTGNSFHNGFQHSGIQLPVSLADTVYGMN

>AaMyc-bHLH16

MEVLQEMMDRLRSIVGPESWDYCVLWKPCKEQRVIEWIDCCCSGSNAARHGIGNDDQQQQLVFQCKDVAFHHPTTDTCNLLSLLPSSMPFDSGLYGQTMISNQPRWLNFSNSSNSGYSEENLGTKVLIPVPIGLVELFVSKQISEDQSIIDFVTTMFNMSLEQPMLNPNNNIDSSFSVNMDSLDDGESKDYIAQVLDDQKDQNNHFQPPISPATMLENLNLSPNNISDNHMHPMNFLQQFNYGESRNTNNIFMEGTSEPMMNHDGPFDPNSEDNVGFDHEIDMALQGQMMSENMGKAHLMEPLENTPKKQGNDMNRSDSVSDCSDQNDEDDDPKCRRKNGKGQSKNLMAERKRRKKLNDRLYTLRSLVPKITKLDRASILKDAIEYVMELKRQVEDLQNELEENSDDEGTTNNQSTIVEQEVIHGNGSNSKRRYNHGPGLFVNGPQLEAYSGVGNIEVSKQNQDVEGASEKGQQMEPQVEVASLDGNEFFVKVFSEHKPGGFVRLMEAFNALGLEVTNVNVTSFRCLVLNVFKVERKDSEMVQADHVRESLLEITRNPYKGWPENSMKAQENGHGMAKVLLESFNGLRNKPNPGNSSSPPYDLCFFSHPSVILQQSLLHHQDIQMTRSPFAQQKKRRIQAAMIAATGNFNIITTMVRRFMLGCLIINHLIESKTLRRKYMVSLSSRRDNMWSMVYQSDIASVVNIRMNILAFSKLCKLLETRGGLHSSKHMLVDEQLVLDAICRLHKEFYKTPVRVPDNETDERWKWFKGCLGALDGTYIKVRVPTCDRKPYRTRKGEICTNVLGVCTRDLMFSYVLAGWEGSAADSRVLRDAISRPNGLKITRVTYMYHEETMSPFRVVLSSTA

>AaMyc-bHLH17

MGKSLLGPDNSDDAEWFYVVSLTRSFSAGDGSAPANSFASNSVIWLTGAHSLLSFDCVRAKEAHIHGLETLVYIPTTNGVVEMGSFHVINHTESDLAHRAKSLFEMGARLPQEEESMNIIDFGTITFDQQPKKLGKIVKNMNMKGNEVSETGSEESDSDCQLVVATSKKVGQKKKGRDPPINHVEAERQRREKLNQRFYTLRSVVPNVSKMDKASLLADAVCYINELKGKVEELEAQLQATNNQPKLKKIKIEIPDVTIVPKSNGAKIYNKKTQTKMIENLEVEVKMVGEDAMIRVQSGNGDWPAAKLMDALREMEVKVHHASMSCVNDIMLQDVVARITGSTEDEVKSHLLARLNQ

>AaMyc-bHLH18

MGTLQQDSIVKNVIKNLCCSYGWSYGAFWSYDQPNSILLTLQDAYFEEKCGSLIDNLVRQVPLGGGIIGQAAYNNKHTWMSSEDHYNEQNFSGSIWDMFLDDYEFRRQFSGGMKTIAVIPVEPRGVVQFGSIDKILETVEFVNQTKIMFQEILNLGGSEVGLTSLDGQTCYQNEAFASLISPQESFFTDFGIPDEFFQNGTFPPLNPIDSDGKHESLTISGTDVDLLRNSGDLGHILAPFIDGSHSGFHSYSSECMSMSKSAQRVDCTTRGPKERLFSKLGIEELLEGVSGISNADSLSCIDGQISAKRRKTGNSKWEEVMPISQPCLHMVDGYSVSDSSTVMQAKKQVEPLKPIKKKAKPGTRPRPKDRQQILDRMAELRLLIPNGEKMDANVAAEVSADVPLVNTSVETPESADVSATEPSSHHSIYIPGRRAKRMARMKVSSSSVHREVDLDAAESSFXSIC

>AaMyc-bHLH19

MGTLQQDSIVKNVIKNLCCSYGWSYGAFWSYDQPNSILLTLQDAYFEEKCGSLIDNLVRQVPLGGGIIGQAAYNNKHTWMSSEDHYNEQNFSGSIWDMFLDDYEFRRQFSGGMKTIAVIPVEPQGVVQFGSIDKILETVEFVNQTKIMFQEISNLGGSEVGLTSLDGQTCYQNEAFASLISPQESFFTDFGIPDEFFQNGTFPPLNPIDSDGKHESLTISGTDVDLLRNSGDLGHILAPFIDGSHSGFHSYSSECMSMSKSAQGVDCTTRVPKERLFSKLGIEELLEGVSGISNADSLSCIDGQISAKRRKTGNSKWEEVMPISQPCLHMVDGYSVSDSSTVMQAKKQVEPLKPIKKKAKPGTRPRPKDRQQILDRMAELRLLIPNGEKMSIDCLLDRTIKHMIFMQNLAKQADKIKQAEERKHNRIDSNDTSTNGVTWACELGNQTMVCPLMVEDLDEPGQMLIEMICEEQGFFLEIVDIIRRFGLIILKGVMETRGDKIWARFIVEPEVNKHITRHEIFSALVKFLQENAHIVDEKCTRQGNSLLGDFQQAGIQNIGIQNLVNLADMQYFVNL

>AaMyc-bHLH20

MAQSSHQTRLKFILQNRPERWLYAIFWIASKKTDDHLVLEWADGYFPETNYVFGLDDVSDTQWLIMSSLGMCFTAGHDVVGQCFGSRSCVWLAGDIELGKYDSKRCEEVRVHGIKSLFCLTKARSFLGLQNQVGDGQEEVMSTKKMKMSSSDSDPLEMNSSSSLTTKKTCTPKRKGRRVKGTIAQPEVLVPGYHVEAERQRREKLNHRFYALRSVVPYVSKMDKASLLADAVTYINELKSKIQRLENNKESESSLIRPRNGNQLNINQCNHVHDQRTTGHSMSNKVEVEVKLLESEAIIRVQSAEVNHPACKLMDALRSLNLKVNYASVSCVKDLMLQDVIVKVPNGFTSEEDTLRLAILNKMCLD

>AaMyc-bHLH21

MSALTERLRPLVETKSWDYCIVWKFGDDPSRYIEWFGCCCNGSSNQDVCGNVKKEIEETKPRSSQVCRDTFVEHGLGTKACEKLADMPFYLPLYSGVHGEVAMSGQPSWSHDTIGTQVLFPVNGGLLELYISKQVPRDEEMIETLTAQFNALSKDEWFCDTKTAAQHMVYLKTEGSPNGEGLWSENSSLVSAGSAQVSPTQSIDNPINVLGDMKSRQKNGKEQYQSKNLVTERKRRNRIKENLYILRSVVPKISKMDKASILGDAIEYIKELQNNVQELQDELKRSEEDEIKSHEEEVEVCKPKRKRAYEHSPTKGHSLVSTSPDKKIEVTVEVHQIGAKDFWLKLVCGQERGVFKRIMETLDSLELQVIDVNVTTCYGHVLTNLKVEAKGKEVVAAKSLKDLLLNCWMPGIHDENQRRG

>AaMyc-bHLH22

MALLNTLDLLRPLISTKSWDYCIVWKFTNDPLMCIEWVGSCCSGSQGVCGNVKCENEAYLCKDSCVKHLIRTDACVKLAMVPSSLPFYPGIHGEVAVSKQPFWQTTDLSGSQLVVPVDGGLIELYRSKHVPTDERTIEALIARLSNIVDPKLDEEDSKTRLNSYLFDHIGPNLQLLIPLPELISPLPELVSHPTTPGLTSGNDNEVMVAKQKKVKKQFKSKNLEAERNRRKRIKDNLLILRSLVPKVSKMDTVSIVGDAIKYIQELQENAKELEDELKALEEQDCMVNGHEVKDVEVEVHKIGARKYLLKVFCSHKPDGFSRLIEAVQSLGLQVINVSMTTCIGLVLNTLAVEAKEEIDAKSLKDSLLSSWTSSELDQKL

>AaMyc-HLH1

MENLRQRLAMAVKSIQWSYAIFWSTSSTEQGVLTWCDGYYNGDIKTRKTIQAEGMNEDDDDGQVGLQRTEQLKQLYESLSAAETHHYEPQARRPSAALSPEDLTDTEWYFLVCMTFEFGYGQGLPGRTLAKNTTSWLSDAHLADSKVFTRSLLAKSASIQTVVCIPYLEGIVEFGITERVLEEQNIIQRIKSLIFIAPPQKIHEIPLESCSAMLDHDLIHNNLSTSRAGDLWSDDDSRYQCVLSKIFKNTQRSAMGPDYRNSDSEKSAFVSWKNYDGMEWKGSSSQMLLKNVLYEVPKMHENHLSRYYDKNGNLDRMQEVAVDDVNDVNHRFSVLSSIVPSRGKVDKVSLLDDTINYLKTLERKVEVLQSRKKSHDVRERTSDNYANKRKASCALEDIQEECSSDCITVSAIEKDVTIEIRCKWRENMMVQVFDAMSSLNLESHSVCSSTVDGILTLSIETKLKKFTTSTAKMIRQALQRVIGRY

>AaMyc-HLH2

MNEDDDDGQVGLQRTEQLKQLYESLSAAETHHYEPQARRPSAALSPEDLTDTEWYFLVCMTFEFGYGQGLPGRTLAKNTTSWLSDAHLADSKVFTRSLLAKSASIQTVVCIPYLEGIVEFGITERVLEEQNIIQRIKSLIFIAPPQKIHEIPLESCSAMLDHDLIHNNLSSMLEYDQPLVRKPHNSPKNSVGAFQPHEQSWQFVDDDGDDDEDEEEEEGEVSYYHNNSMGSSDCVSQNLASRAGDLWSDDDSRYQCVLSKIFKNTQRSAMGPDYRNSDSEKSAFVSWKNYDGMEWKGSSSQMLLKNVLYEVPKMHENHLSRYYDKNGNLDRMQEVAVDDVNDVNHRFSVLSSIVPSRGKVDKVSLLDDTINYLKTLERKVEVLQSRKKSHDVRERTSDNYANKRKASCALEDIQEECSSDCITVSAIEKDVTIEIRCKWRENMMVQVFDAMSSLNLESHSVCSSTVDGILTLSIETKLKNFTTSTAKMIRQALQRVIGRY
